# Modelling of ‘sub-atomic’ contrast resulting from back-bonding on Si(111)-7×7

**DOI:** 10.3762/bjnano.7.85

**Published:** 2016-06-29

**Authors:** Adam Sweetman, Samuel P Jarvis, Mohammad A Rashid

**Affiliations:** 1 The School of Physics and Astronomy, The University of Nottingham, Nottingham, NG7 2RD, United Kingdom

**Keywords:** NC-AFM, qPlus, sub-atomic, sub-molecular

## Abstract

It has recently been shown that ‘sub-atomic’ contrast can be observed during NC-AFM imaging of the Si(111)-7×7 substrate with a passivated tip, resulting in triangular shaped atoms [Sweetman et al. *Nano Lett. ***2014**, 14, 2265]. The symmetry of the features, and the well-established nature of the dangling bond structure of the silicon adatom means that in this instance the contrast cannot arise from the orbital structure of the atoms, and it was suggested by simple symmetry arguments that the contrast could only arise from the backbonding symmetry of the surface adatoms. However, no modelling of the system has been performed in order to understand the precise origin of the contrast. In this paper we provide a detailed explanation for ‘sub-atomic’ contrast observed on Si(111)-7×7 using a simple model based on Lennard-Jones potentials, coupled with a flexible tip, as proposed by Hapala et al. [*Phys. Rev. B ***2014**, 90, 085421] in the context of interpreting sub-molecular contrast. Our results show a striking similarity to experimental results, and demonstrate how ‘sub-atomic’ contrast can arise from a flexible tip exploring an asymmetric potential created due to the positioning of the surrounding surface atoms.

## Introduction

Recent developments in low temperature scanning probe instrumentation [1], coupled with specific experimental techniques utilising the in situ functionalisation of scanning probe tips with single molecules [2], and operation in the constant-height imaging mode, have resulted in an explosion of interest in high-resolution imaging and force mapping of atomic and molecular structures using non-contact atomic force microscopy (NC-AFM). In particular, suppressing the chemical bonding between tip and sample enables the stable exploration of the repulsive part of the tip–sample force regime, which has allowed outstanding resolution to be obtained during imaging of planar organic molecules [3–4]. An important development in the interpretation of sub-molecular resolution imaging has been the explicit consideration of deflection (i.e., mechanical deformation) in the tip–sample junction [5–7], which can result in contrast enhancement [6], but also unwanted distortions and potential artefacts [5,7–10]. Modelling using computationally inexpensive empirical potentials has produced a surprisingly good agreement with experimental data, and also allows for simulated images to be computed with a comparable size and resolution to experiment, which is essential for meaningful qualitative comparisons.

In this paper we explore the application of a simple Lennard-Jones model with a flexible tip probe [5,7–8] to a case of ‘sub-atomic’ imaging on the Si(111)-7×7 surface [11–12]. We show that the triangular features observed experimentally arise naturally from the exploration of an asymmetric potential by a flexible tip and do not require consideration of the detailed electronic structure of the surface. By constructing artificial surface slabs utilising different elements of the full Si(111)-7×7 unit cell, we are able to examine the relative influence of the different parts of the surface on the contrast. Our simulations show the influence of the backbonding atoms (that is, the atoms directly behind the topmost adatoms, via which they are bonded to the surface), and also the influence of the rest atoms in the unit cell, on the triangular adatom contrast. We also highlight the limitations of the model when chemical interactions become important at close approach, and explore the qualitative variation in contrast observed between force and Δ*f* images depending on oscillation amplitude.

## Simulation Methods

To simulate constant height force images, we used the method proposed by Hapala et al. [7,13] to model the interaction between a functionalized probe and the Si(111)-7×7 unit cell, using simple Lennard-Jones (L-J) potentials. In this model the functionalized tip is assumed to consist of a tip base, representing the end of the bulk tip material, and a single passivated probe particle. In order to simulate the mechanical deformation in the tip–sample junction, the probe particle is allowed to move around the tip base, and acts as a flexible end of the model tip. To model the Si(111)-7×7 surface we imported a relaxed geometry from previous density functional theory simulations performed in our group, details of which are described elsewhere [14]. During the force field calculations the positions of all the atoms in the surface slab were kept fixed. We note that more sophisticated versions of the probe-particle model also incorporate the effect of electrostatics via introduction of the Hartree potential, which has been shown to have important consequences for the imaging of certain classes of molecules [15]. In our simulations the effect of the Hartree potential is not included, primarily as electrostatic forces are not expected to result in significant differences in contrast due to the small variation in electrostatic force over the different atoms of the Si(111)-7×7 unit cell [16].

In the simulations the probe particle is subject to forces from three sources: 1) a L-J-like interaction due to the tip base, 2) a sum of all pairwise forces due to interactions with the atoms in the sample slab, and 3) a lateral harmonic restoring force from the tip base. We used the same L-J parameters as described by Hapala et al. [7], i.e., a tip base with parameters *r*_α_= 2 Å, and ε_α_ = 1000 meV (artificially large to keep the probe particle attached), and a probe particle with parameters *r*_α_ = 1.66 Å and ε_α_ = 9.106 meV, and a lateral stiffness of 0.5 N/m.

In the L-J model, the interaction between atoms α and β are written as:

[1]
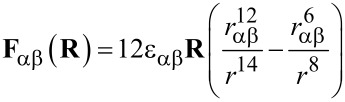


[2]
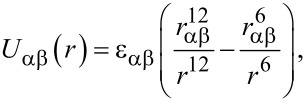


where *r* = |**R**| is the distance between atoms α and β, 
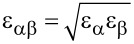
 is the pair binding energy and *r*_αβ_ = *r*_α_ + *r*_β_ is the equilibrium separation of the two atoms with ε_α_ and *r*_α_ being the atomic parameters. In our calculations the L-J parameters for the silicon atoms were set to ε_α_ = 25.489 meV and *r*_α_ = 1.9 Å. We acquired the simulation data by scanning the sample laterally with a step of Δ*x*, Δ*y* = 0.1 Å. At each lateral position we placed the tip base at an initial separation *z*_0_ = 15 Å from the surface molecule and approached the sample in steps of Δ*z* = 0.05 Å, allowing the probe position to be relaxed at each step due to the combined force of the sample and tip base. After calculation of the 3D force field, a complementary Δ*f* grid was calculated using the method proposed by Giessibl et al. [17], assuming cantilever parameters of *k*_cant_ = 1800 N/m and *f*_0_ = 30 kHz.

It is important to stress that there are a number of differences between the systems normally modelled using this approach and the experimental system to which we compare our results. Typically, in sub-molecular resolution imaging experiments, well defined atoms (such as Xe or Cl), or molecules (such as CO) are picked up from metal surfaces onto metal-coated tips by STM protocols [3]. In our experimental data the initial tip termination is likely silicon due to prior STM treatment of the tip on the clean Si(111)-7×7 surface (although the tip bulk material is tungsten). In addition, the identity of the passivating end group, which was picked up spontaneously during NC-AFM imaging of the clean surface, is not known. Although CO is a common vacuum contaminant, our tip termination could also easily be a number of other common contaminants (for example H, OH or O), which would also suppress the chemical reactivity of the tip apex. Therefore our modelling, using CO parameters, is only intended to represent a ‘generic’ passivated tip. In particular, the chemical interaction between the passivating end group and a silicon-terminated tip is likely to be different to the interaction between CO and a metal terminated tip, which may explain the differences between experiment and simulation which we observe at close approach (see later discussion).

## Results

### Origin of triangular contrast in simulated images

**Figure 1:**
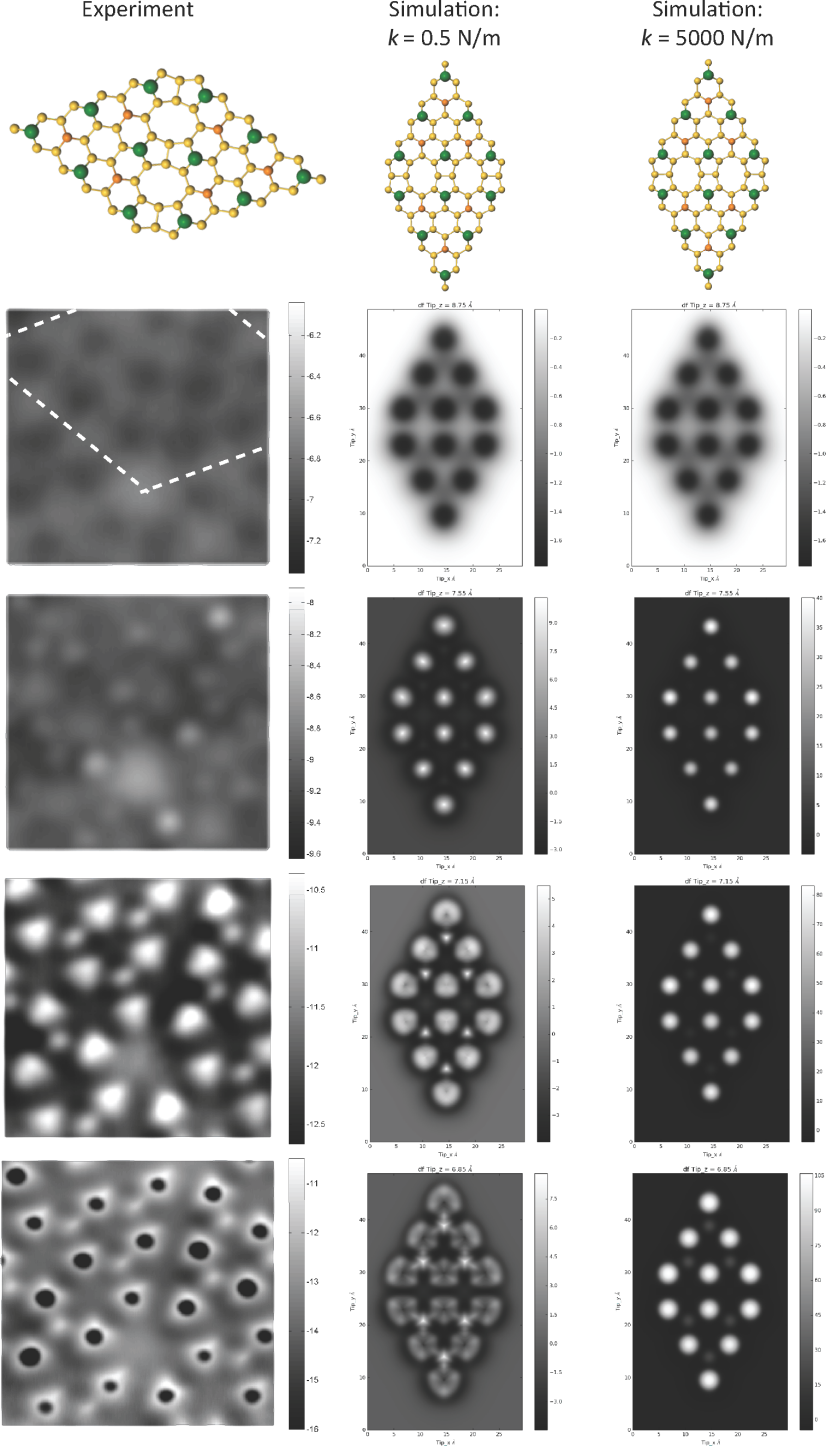
Left column: Experimental constant height Δ*f* images at decreasing tip–sample separation. Note a 10 point gaussian filter has been applied to all images to remove high frequency noise. Experimental parameters: *A*_0_ = 110 pm, *V*_gap_ = 0 V. Experimental tip heights relative to Δ*f* feedback setpoint (top to bottom): +0.186 nm, +0.104 nm, +0.032 nm, 0 nm. Image size 3.6 nm × 3.6 nm. Data acquired at 77 K. Middle column: simulated constant height force images over a Si(111)-7×7 unit cell using a flexible tip with stiffness *k*_xy_ = 0.5 N/m. At close approach triangular adatoms and rest adatoms become apparent, strikingly similar to the experimental images. At very close approach an inversion occurs directly over the adatoms resulting in a dark hole in the centre of the atom. Right column: simulated constant height force images using a tip with stiffness *k*_xy_ = 5000 N/m. The atomic features remain spherically symmetric at all tip–sample separations. Simulated tip heights: (top to bottom) 0.875 nm, 0.755 nm, 0.715 nm, 0.685 nm.

Figure 1 shows a comparison between experimental constant height Δ*f* images (acquired during the same experimental run as [11]), and simulated constant height Δ*f* images using a flexible, and very rigid, tip apex. In both cases the images have been selected from full datasets in order to best illustrate the evolution of the contrast as the tip–sample distance is decreased, full datasets are available in Supporting Information File 1–Supporting Information File 2.

Far from the surface (top row Figure 1) the adatoms of the surface appear as attractive features (i.e., dark depressions resulting from more negative frequency shifts). Likewise, both of the simulated sequences show attractive contrast at large tip–surface separations, as expected for a L-J interaction. Closer to the surface (second row from top Figure 1) the adatoms and rest atoms begin to image as repulsive features (bright regions corresponding to more positive frequency shifts). At this height the triangular shape of the atoms is already visible. In the simulated images the adatoms and rest atoms are visible as repulsive features, but are only slightly non-spherical. Further into the repulsive regime (second row from bottom Figure 1) triangular adatoms and rest atoms are clearly observed experimentally, and these features are reproduced well in the simulations using the flexible tip. At very close approach dark depressions are observed in the center of the adatoms experimentally (bottom row Figure 1), which corresponds to the onset of a strong attractive interaction. It is possible that these features correspond to some reversible change in the tip state due to the strong repulsive tip–sample forces – e.g., either a change in the position of the passivating end group, or some modification of the chemical reactivity of the tip due to mechanical deformation. Interestingly, somewhat similar features are reproduced in simulation using the flexible tip, with an inversion of contrast directly over the adatoms. This results from the deflection of the tip, and is the origin of the contrast inversion during intramolecular imaging described previously by Hapala et al. [7]. However, it is important to note that the simulations do not reproduce the dramatic drop in Δ*f* observed experimentally, as the simple Lennard-Jones potential used does not take into account chemical interactions, or changes in the chemical reactivity of the tip. This evolution in contrast is not reproduced in the simulations using a very stiff tip (right column), where the atoms of the Si(111)-7×7 surface remain spherical throughout. This highlights the essential requirement for considering the flexibility of the tip apex in interpreting contrast obtained in the repulsive regime using passivated tips. There are also some additional minor differences between the simulation and experimental results. Most notably, a difference is observed experimentally in the contrast over the different adatoms of the Si(111)-7×7 unit cell, corresponding to the known differences in chemical reactivity at these sites [18]. As each of the atoms in the simulations has identical properties, this variation is not reproduced in the simulated images. We also note the non-physical asymmetric distortions in the atoms at the edge of the unit cell in the simulations, due to the finite unit cell size. Therefore in comparisons to the experimental data we focus on the appearance of the atoms in the centre of the unit cell, which experience a uniform attractive background.

**Figure 2:**
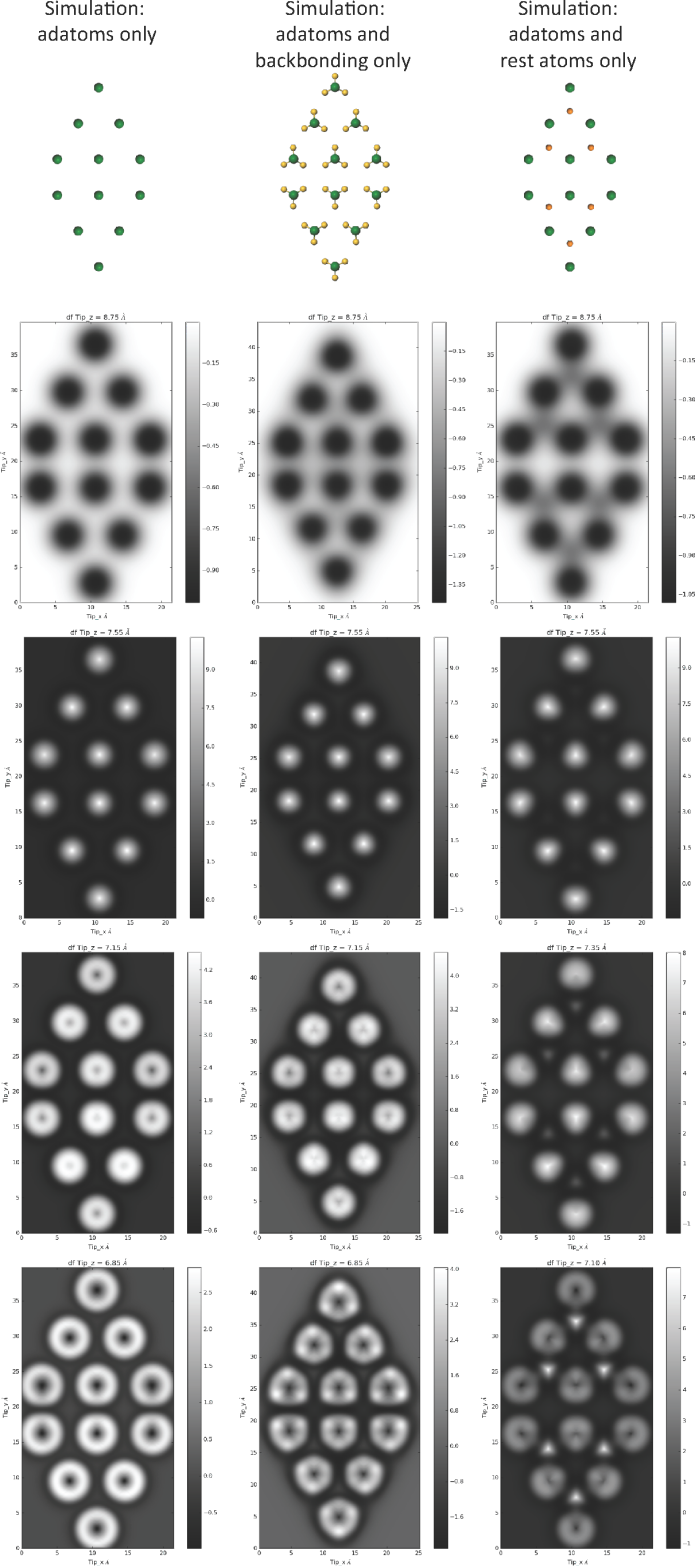
Left column: simulated constant height force images at decreasing tip–sample separation, over a Si(111)-7×7 unit cell containing only the upper adatoms. All atoms image as uniform spheres, at close approach an inversion occurs in the centre of the atoms. Middle column: simulated constant height force images at decreasing tip–sample separation, over a Si(111)-7×7 unit cell containing only the adatoms and their backbonding atoms. At close approach the adatoms appear as triangles with a orientation matching the backbonding atoms, with an inversion in the centre of the atoms. Right column: simulated constant height force images at decreasing tip–sample separation, over a Si(111)-7×7 unit cell containing the adatoms and rest atoms (no backbonding atoms). The rest atoms image as triangles as a result of being positioned in the asymmetric potential of the surrounding adatoms. Also note the weak triangular shape of the adatoms as a result of the potential arising from the position of the rest atoms, in addition to the inversion in the centre of the atom. Simulated tip heights, left and middle column: (top to bottom) 0.875 nm, 0.755 nm, 0.715 nm, 0.685 nm, right column (top to bottom): 0.875 nm, 0.755 nm, 0.735 nm, 0.710 nm. Tip stiffness *k*_xy_ = 0.5 N/m for all simulations.

Although these simulations reveal the key role that relaxation in the tip–sample junction plays in explaining the image contrast, they do not necessarily reveal the origin of the features. Beyond the computational simplicity of the L-J model, an additional advantage compared to more sophisticated modelling techniques is that it is easy to construct “toy” systems that allow us to explore how different parts of the surface contribute to the image contrast. A selection of simulated image sequences using this approach is shown in Figure 2, which helps elucidate the origin of the contrast in the simulations shown in Figure 1. The first column shows a control model, where only the adatoms of the Si(111)-7×7 unit cell have been used as the surface slab. In this sequence the adatoms image as uniform spheres at all heights, with the exception of the previously mentioned contrast inversion in the centre at close approach. When the atoms in the backbonding positions are included (middle column), the evolution of the adatoms goes from spherical to triangular contrast with close approach of the tip. Consequently, it appears that the presence of the backbonding atoms creates an asymmetry in the repulsive part of the potential, producing a complementary asymmetry in the deflection of the probe particle as it explores the tip–sample interaction. Interestingly, when only the adatoms and rest atoms are included in the surface slab (right hand column) the rest atoms appear clearly triangular, and the adatoms also have a triangular symmetry, despite the lack of atoms in the backbonding positions. This separation of the effect of the different elements of the surface slab illustrates how the appearance of the atoms is shaped by the potential created by the entire surface. In the case of the rest atoms, their position within the asymmetric attractive potential of the surrounding adatoms means that a saddle in the potential is created, resulting in an asymmetric deflection of the probe particle – an effect that is enhanced by their lower topographic location relative to the adatoms. The complementary influence of the rest atoms on the appearance of the adatoms is somewhat reduced (due to their lower height), but is still sufficient to produce a noticeable change in their appearance. Consequently, the simulations suggest that the experimentally observed triangular shape of the atoms results from the potential that results from a combination of the effect of the backbonding atoms, and presence of the rest atoms.

### Comparison of Δ*f* and force, and effect of oscillation amplitude

**Figure 3:**
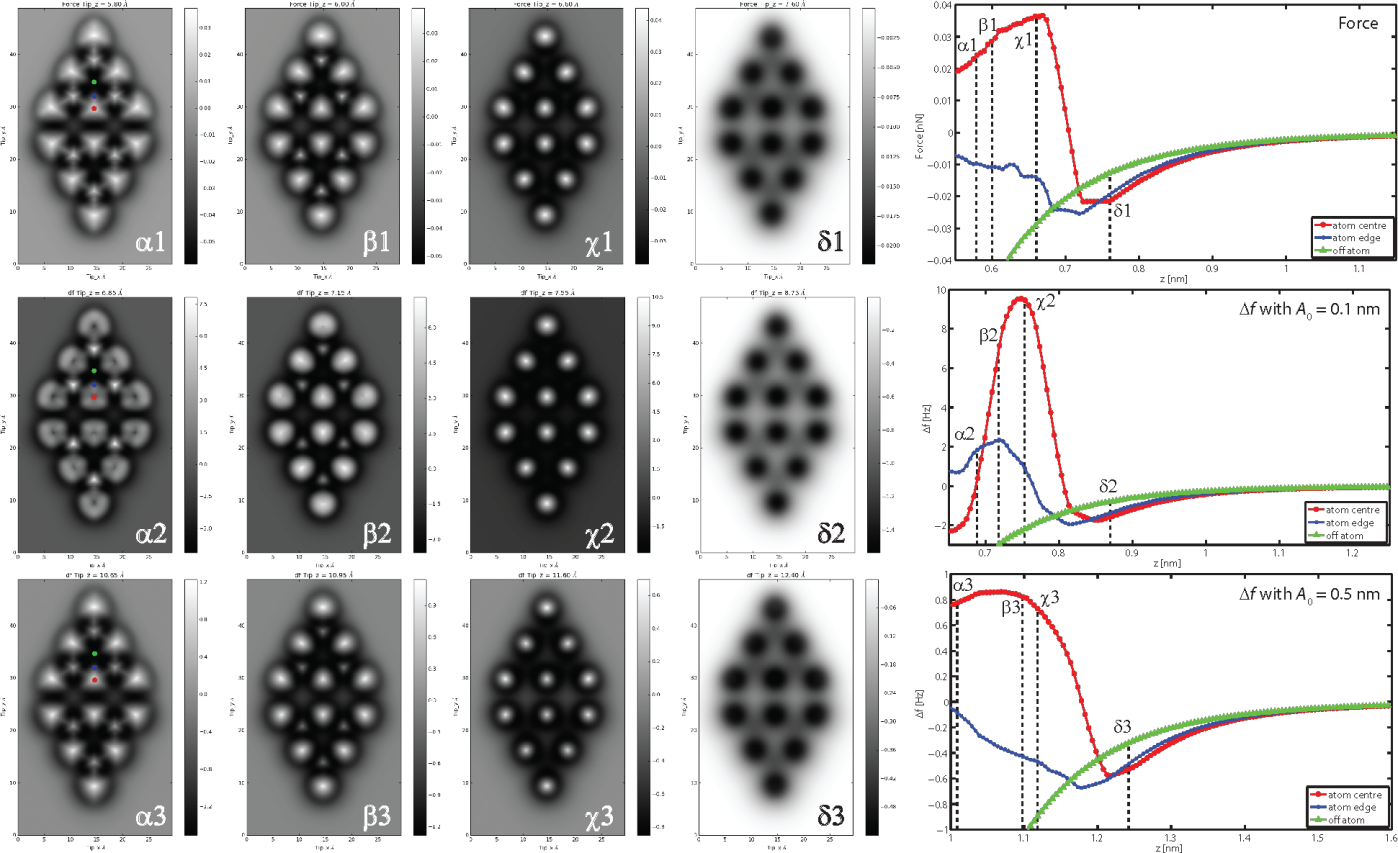
Comparison of the evolution in force (top row) and frequency shift (lower two rows). The evolution in Δ*f* is shown for oscillation amplitudes of 0.1 nm (middle row), and 0.5 nm (lower row). The position of single *F*(*z*) and Δ*f*(*z*) curves are marked on the *xy* images, and the heights of each image is marked on the graphs with the corresponding Greek letter. The Δ*f* contrast and evolution in *z* is qualitatively similar for the force and 0.5 nm oscillation amplitude simulations. The simulations with an oscillation amplitude of 0.1 nm show an dark region in the centre of the adatoms, which is reflected in the inversion observed in the Δ*f*(*z*) curves.

In the limit of small oscillation amplitudes, the frequency shift tends towards the force gradient between tip and sample [19], however, it is less trivial to determine how the frequency shift relates to the force with finite oscillation amplitudes [20]. In particular, there has been little consideration of how the use of finite oscillation amplitudes effects the contrast in the Δ*f* images acquired during intramolecular imaging with passivated tips [21], where it is often assumed that the Δ*f* images closely reflect the force and/or charge density associated with the molecule. In Figure 3 we compare constant height force, and Δ*f* images acquired with different oscillation amplitudes, at different tip–sample separations. Intriguingly, the triangular shape of the adatoms is more pronounced in the force images (top row), and importantly, lacks the inversion observed in the Δ*f* images acquired with *A*_0_ = 0.1 nm. The Δ*f* images simulated with *A*_0_ = 0.5 nm (lower row) show a striking qualitative similarity to the force images, being more triangular, and also lacking the contrast inversion over the adatoms. Intuitively, these results may be understood on the basis that at smaller amplitudes the Δ*f* begins to resemble the force gradient, whereas at larger amplitudes the Δ*f* is more strongly dominated by the interaction at the point of closest approach.

### Effect of *k*_xy_ on simulated images

**Figure 4:**
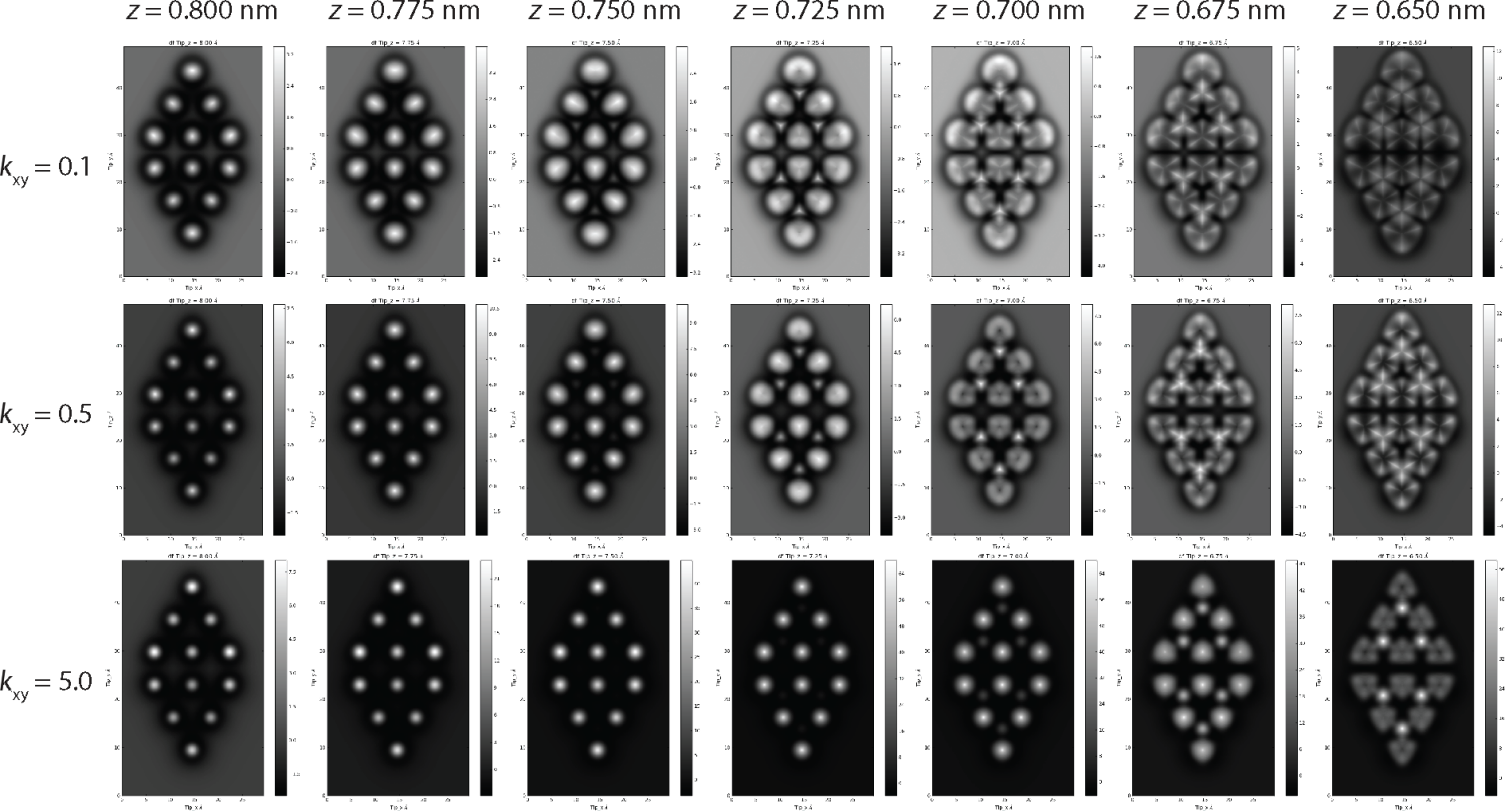
Simulated constant height images at decreasing tip–sample separation for three different probe lateral stiffness (*k*_xy_). The triangular appearance of the adatoms, and the subsequent contrast inversion, occurs at larger tip separations for lower stiffness probes. For the lowest stiffness probe the ‘hole’ produced by the contrast inversion is almost reduced to a point due the the extreme sharpening of the features caused by the deflection of the probe.

As noted above, the tip used to take the experimental images in Figure 1 was not intentionally functionalised, and, more importantly, the identity of the passivating group at the apex of the tip is not known. In the majority of the simulations performed in the previous sections, we have assumed a lateral stiffness *k*_xy_ = 0.5 N/m, in line with previous work modelling CO terminated tips. However, a priori, we have no knowledge of the actual stiffness of our probe, and it is important to consider what a modification of the lateral stiffness may have on our simulated results. While for small modifications of *k*_xy_ we find that the contrast is qualitatively similar as previously reported [7], we find that for larger changes in *k*_xy_ we observe qualitative changes in the appearance of the simulated images. These results are summarised in Figure 4, where we compare the *k*_xy_ = 0.5 N/m simulations with a very low stiffness tip (*k*_xy_ = 0.1 N/m), and a relatively rigid tip (*k*_xy_ = 5 N/m). Although for all stiffnesses we observe triangular shaped adatoms at close approach, the extent and shape of the contrast inversion in the centre of the adatoms is directly affected by the change in lateral stiffness. The choice of *k*_xy_ = 0.5 N/m therefore appears to be justified empirically for two primary reasons. First, simulations with stiffness’s around this value best reproduce the experimental contrast. Second, tips producing similar contrast over the adatoms also produced very similar contrast during intramolecular imaging experiments, [12], including the characteristic sharpening of the bond features typically associated with the tilting of the CO molecule [6].

## Conclusion

We have modelled an example of ‘sub-atomic’ contrast on the Si(111)-7×7 substrate with a passivated tip, using simple L-J potentials and a flexible tip model. Despite lacking information on the electronic or chemical nature of the surface, the model well reproduces the contrast observed over the adatoms and rest atoms. By decomposing the contributions of different parts of the substrate, we are able to show that ‘sub-atomic’ contrast can in principle arise as the result of a flexible tip exploring an asymmetric potential around an atom unrelated to its electronic orbital configuration. Our simulations show that the local atomic environment (i.e., the position of the other atoms on the surface) can provide such a potential. A distinction must therefore be drawn between what might be termed ‘orbital’ imaging, which explicitly images the orbitals of single atoms, and ‘sub-atomic’ imaging, which can arise from a number of multi-atom effects. Therefore, we suggest that interpretation of ‘sub-atomic’ features that share a symmetry with either the direct backbonding atoms, or even nearby atoms that are not directly bonded to the target atom, must therefore be carried out with the utmost care. We do stress that a simple L-J model cannot reproduce the onset of a repulsive ‘halo’ that occurs before repulsion over the centre of the atom, as was reported recently [22], and interpretation of features of this type requires full ab-initio modelling of the combined tip–sample system, with full consideration of the combined charge density, and the relaxation of the atomic positions, in the tip–sample junction.

## Supporting Information

File 1 Datasets A0=1A adatoms_backbonds k=0.5.

File 2 Datasets A0=1A k=5000.

File 3 Datasets A0=1A adatoms_only k=0.5.

File 4 Datasets A0=1A adatoms_rest_atoms k=0.5

File 5 Datasets A0=1A adatoms_rest_atoms_backbonds k=0.5.

File 6 Datasets A0=1A k=0.5.

File 7 Datasets A0=1A k=0.5_extendedrange.

File 8 Datasets A0=1A k=5.0.

File 9 Datasets A0=5A k=0.5.
