# Supplementary figures and images for: Modelling of ‘sub-atomic’ contrast resulting from back-bonding on Si(111)-7×7 (part 1 of 18)
Source: Beilstein J Nanotechnol. 2016 Jun 29;7:937–45. doi: 10.3762/bjnano.7.85 (PMC4979881; doi:10.3762/bjnano.7.85)

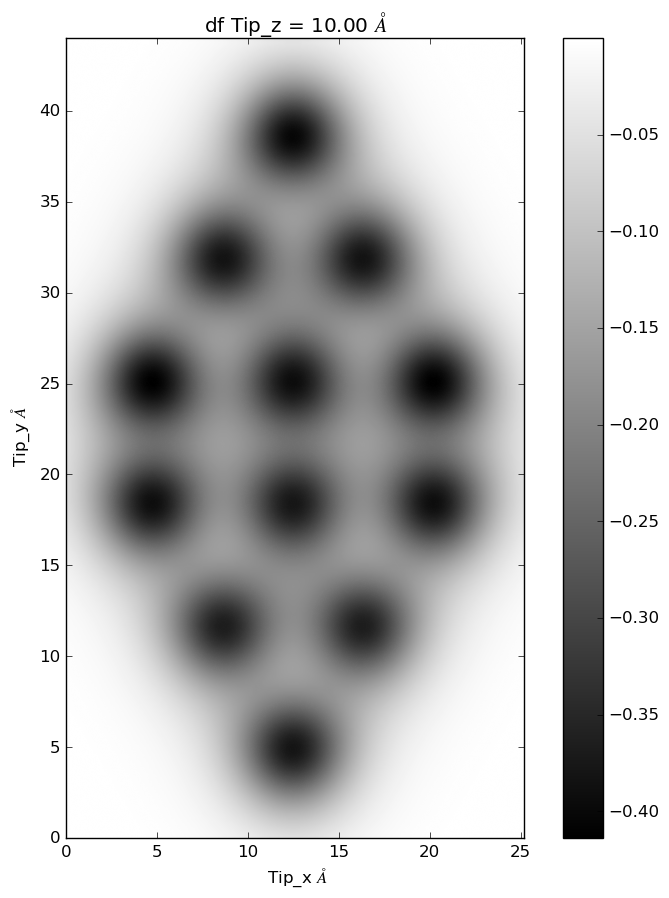

Supplement: File 1 — Datasets A0=1A adatoms_backbonds k=0.5. [file Beilstein_J_Nanotechnol-07-937-s001.zip › S1/A0=1A/adatoms_backbonds/k=0.5/results/df_0000.png]

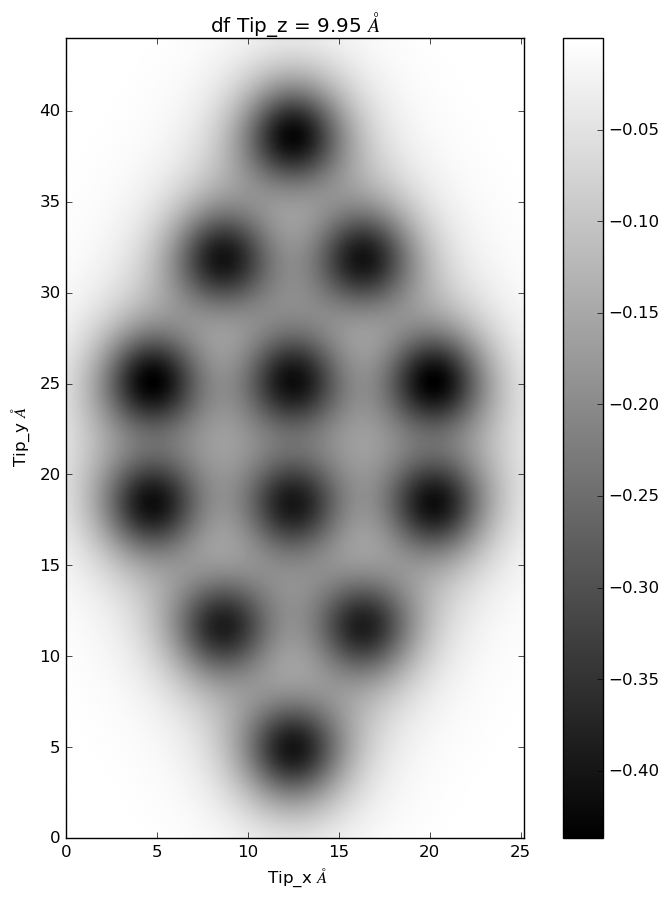

Supplement: File 1 — Datasets A0=1A adatoms_backbonds k=0.5. [file Beilstein_J_Nanotechnol-07-937-s001.zip › S1/A0=1A/adatoms_backbonds/k=0.5/results/df_0001.png]

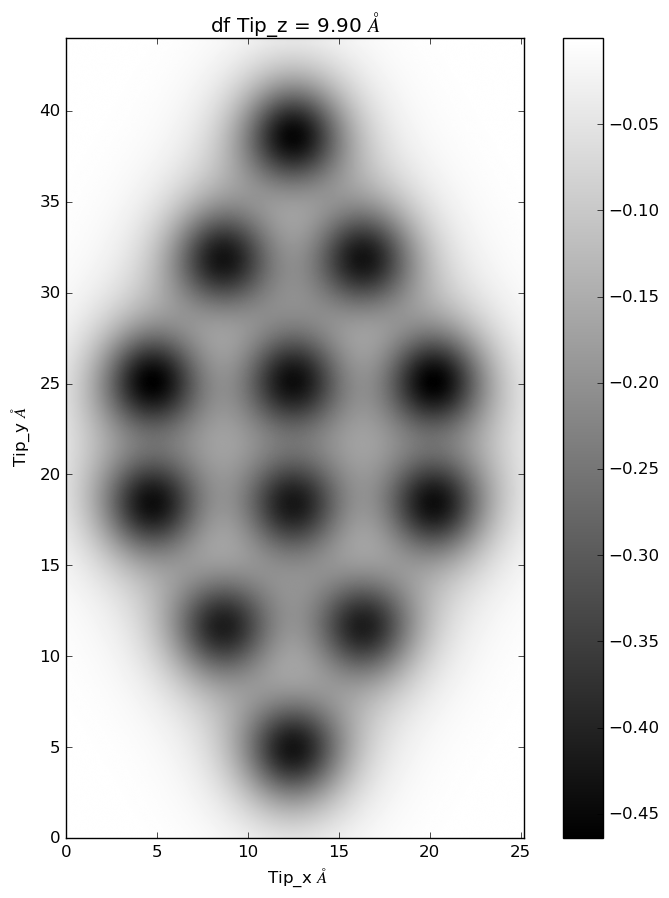

Supplement: File 1 — Datasets A0=1A adatoms_backbonds k=0.5. [file Beilstein_J_Nanotechnol-07-937-s001.zip › S1/A0=1A/adatoms_backbonds/k=0.5/results/df_0002.png]

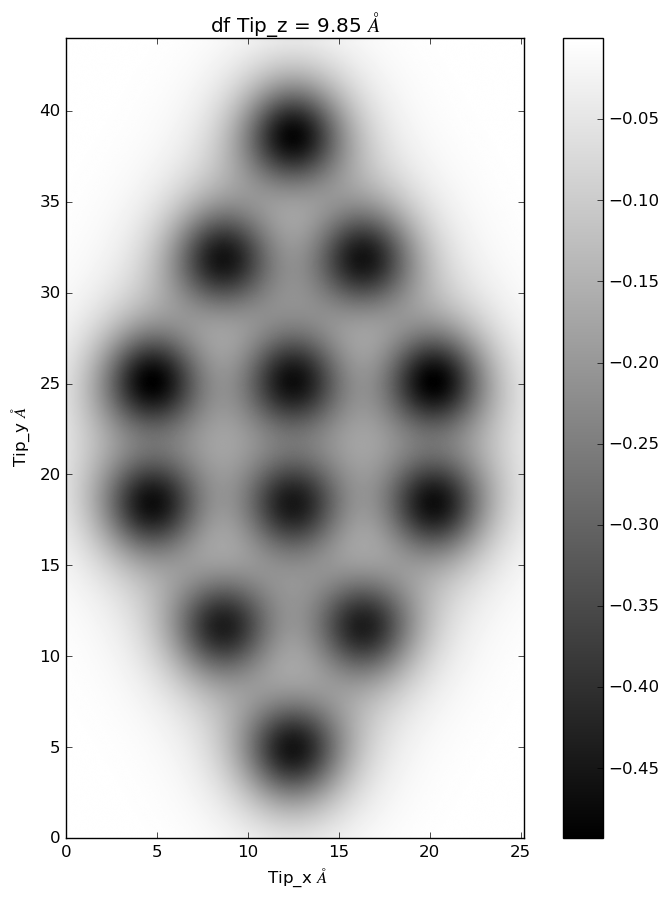

Supplement: File 1 — Datasets A0=1A adatoms_backbonds k=0.5. [file Beilstein_J_Nanotechnol-07-937-s001.zip › S1/A0=1A/adatoms_backbonds/k=0.5/results/df_0003.png]

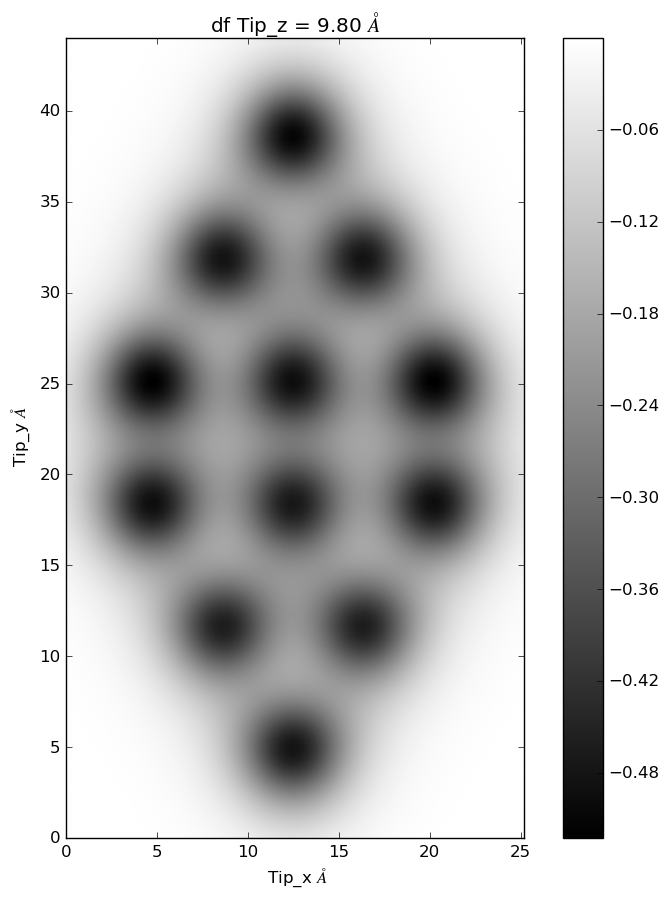

Supplement: File 1 — Datasets A0=1A adatoms_backbonds k=0.5. [file Beilstein_J_Nanotechnol-07-937-s001.zip › S1/A0=1A/adatoms_backbonds/k=0.5/results/df_0004.png]

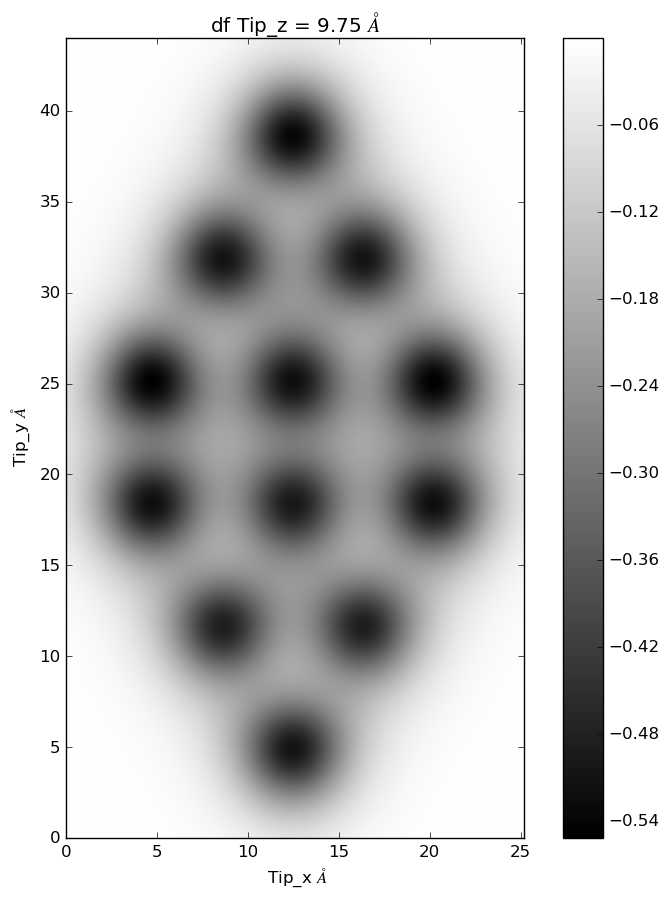

Supplement: File 1 — Datasets A0=1A adatoms_backbonds k=0.5. [file Beilstein_J_Nanotechnol-07-937-s001.zip › S1/A0=1A/adatoms_backbonds/k=0.5/results/df_0005.png]

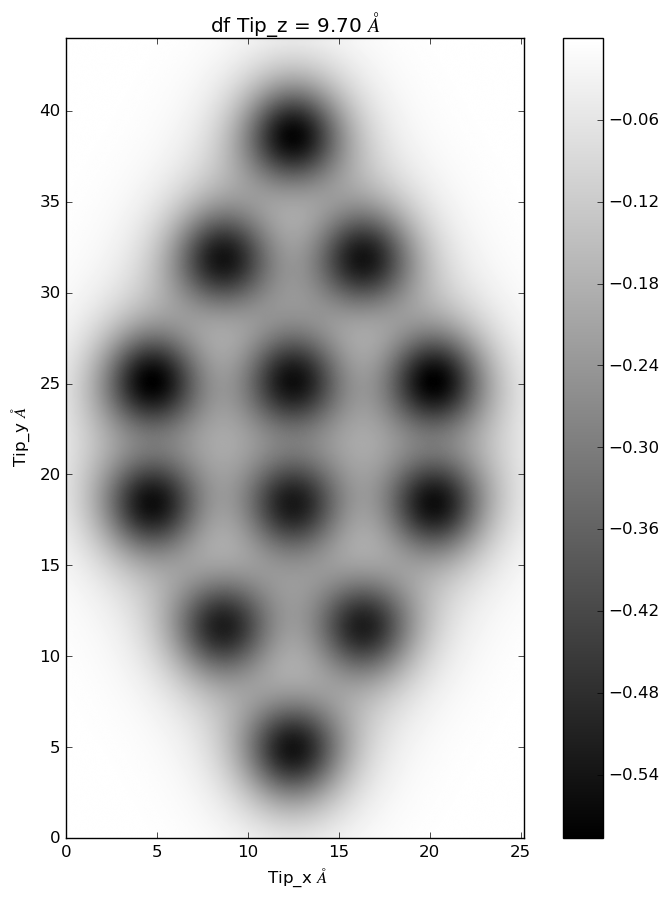

Supplement: File 1 — Datasets A0=1A adatoms_backbonds k=0.5. [file Beilstein_J_Nanotechnol-07-937-s001.zip › S1/A0=1A/adatoms_backbonds/k=0.5/results/df_0006.png]

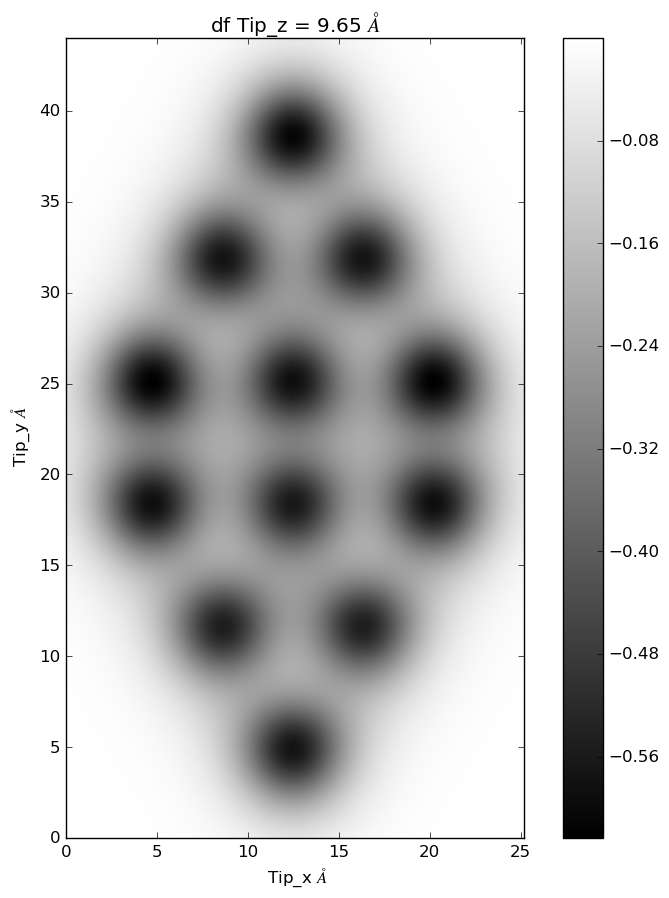

Supplement: File 1 — Datasets A0=1A adatoms_backbonds k=0.5. [file Beilstein_J_Nanotechnol-07-937-s001.zip › S1/A0=1A/adatoms_backbonds/k=0.5/results/df_0007.png]

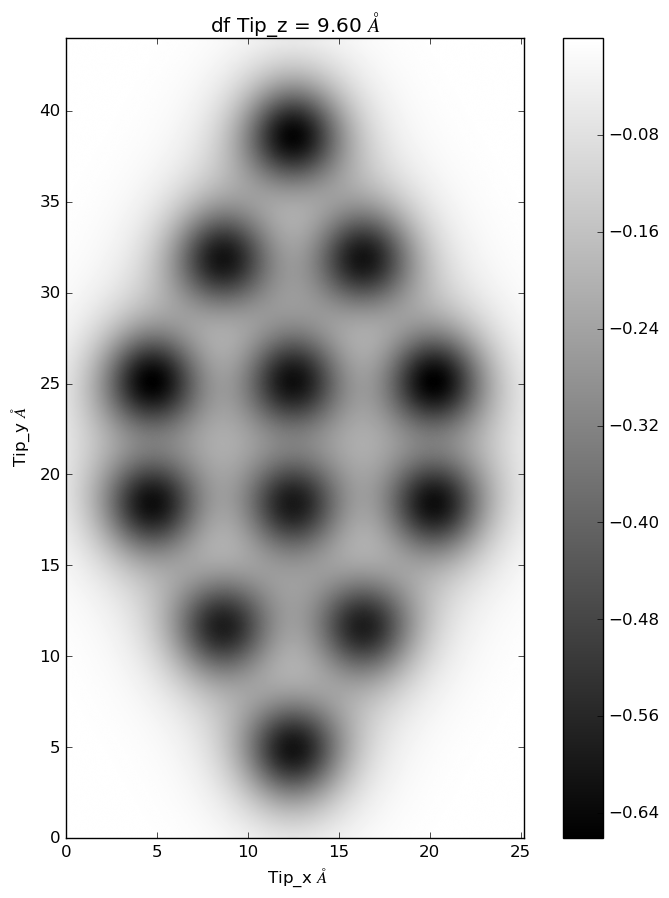

Supplement: File 1 — Datasets A0=1A adatoms_backbonds k=0.5. [file Beilstein_J_Nanotechnol-07-937-s001.zip › S1/A0=1A/adatoms_backbonds/k=0.5/results/df_0008.png]

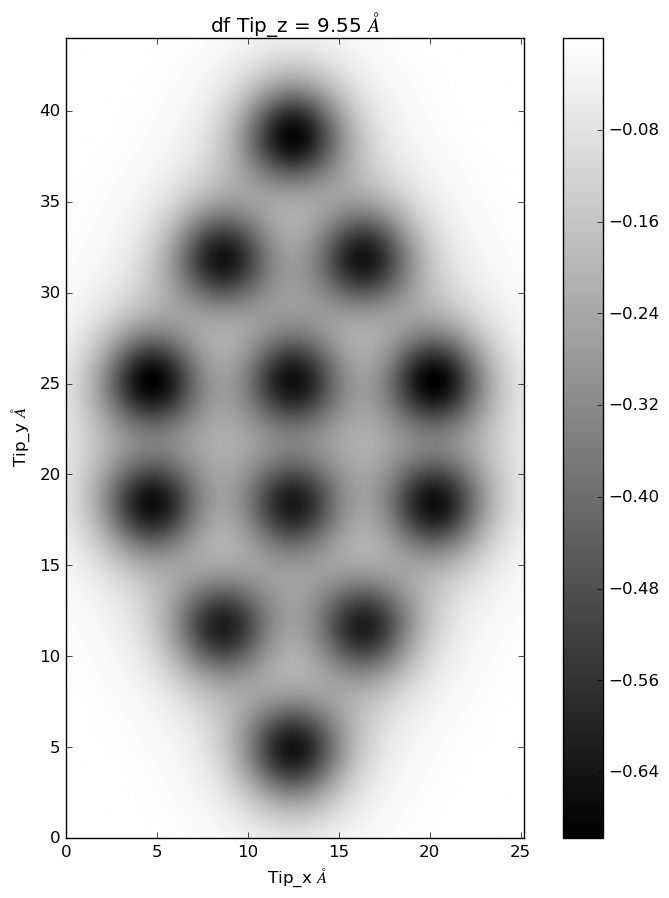

Supplement: File 1 — Datasets A0=1A adatoms_backbonds k=0.5. [file Beilstein_J_Nanotechnol-07-937-s001.zip › S1/A0=1A/adatoms_backbonds/k=0.5/results/df_0009.png]

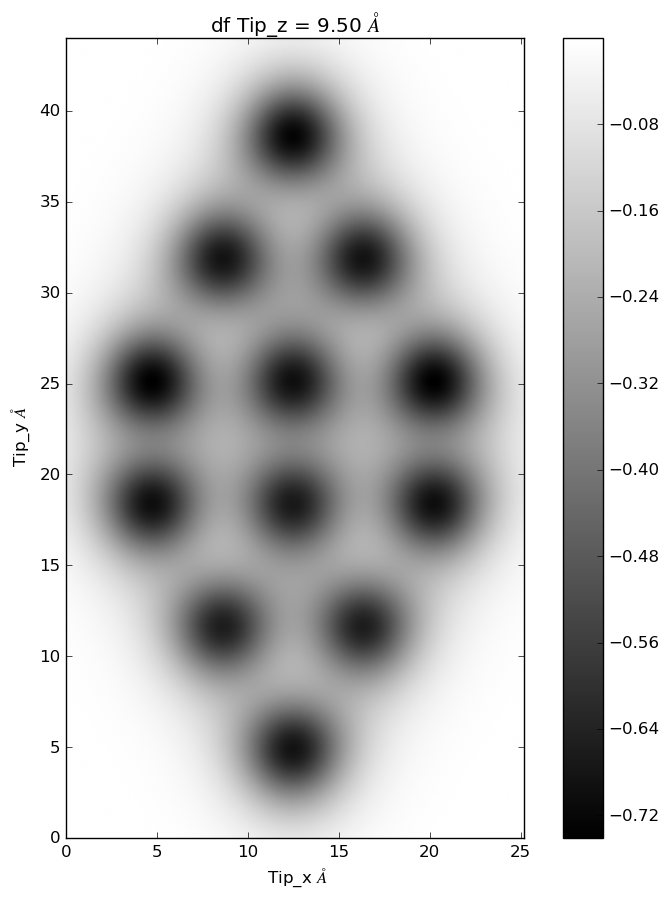

Supplement: File 1 — Datasets A0=1A adatoms_backbonds k=0.5. [file Beilstein_J_Nanotechnol-07-937-s001.zip › S1/A0=1A/adatoms_backbonds/k=0.5/results/df_0010.png]

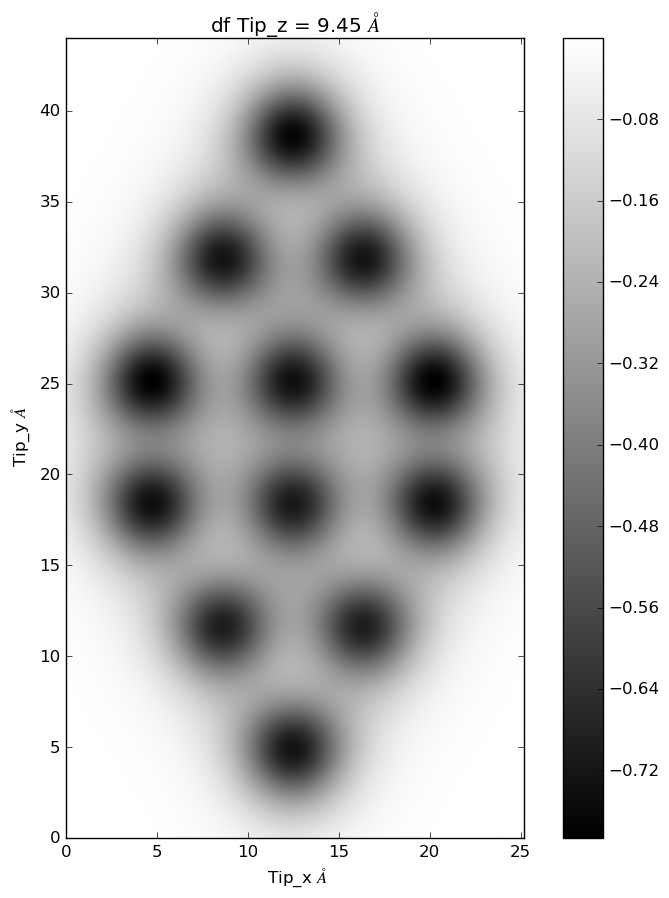

Supplement: File 1 — Datasets A0=1A adatoms_backbonds k=0.5. [file Beilstein_J_Nanotechnol-07-937-s001.zip › S1/A0=1A/adatoms_backbonds/k=0.5/results/df_0011.png]

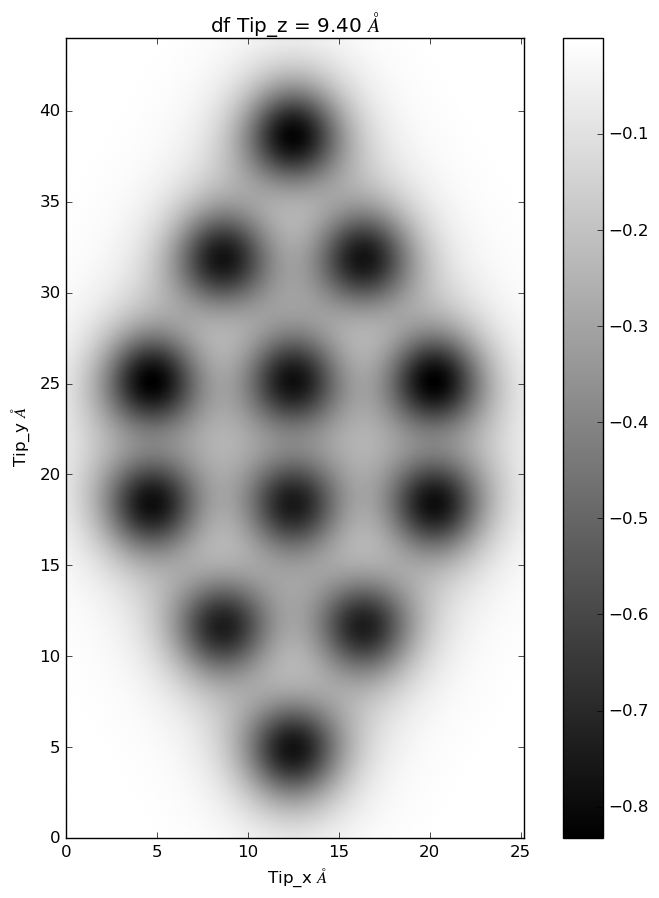

Supplement: File 1 — Datasets A0=1A adatoms_backbonds k=0.5. [file Beilstein_J_Nanotechnol-07-937-s001.zip › S1/A0=1A/adatoms_backbonds/k=0.5/results/df_0012.png]

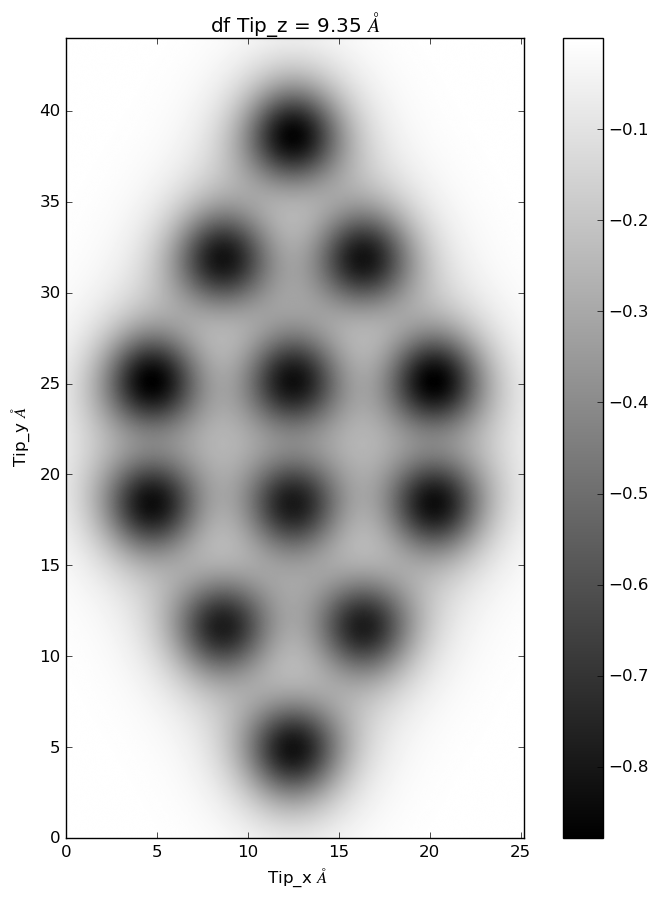

Supplement: File 1 — Datasets A0=1A adatoms_backbonds k=0.5. [file Beilstein_J_Nanotechnol-07-937-s001.zip › S1/A0=1A/adatoms_backbonds/k=0.5/results/df_0013.png]

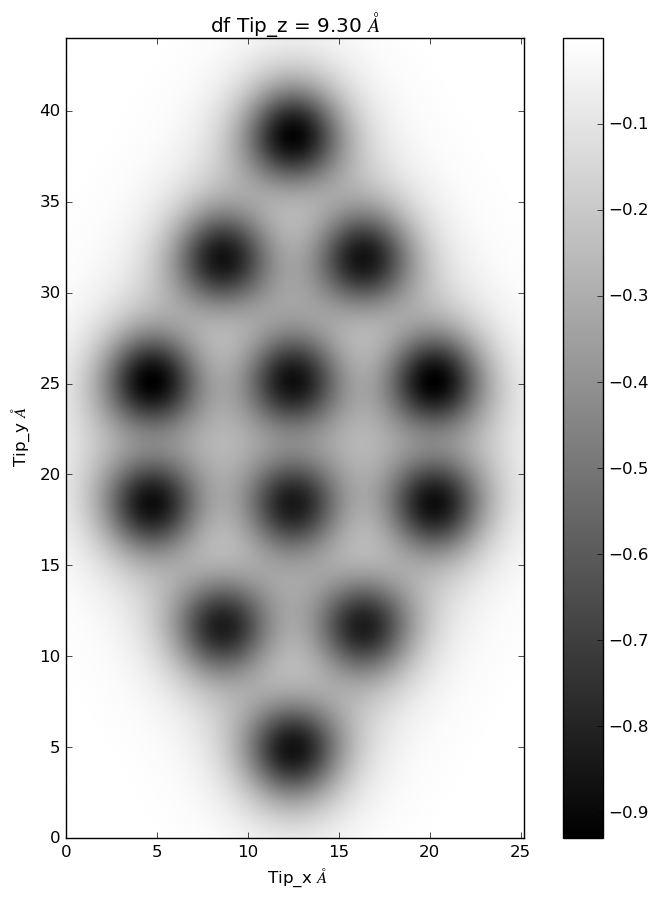

Supplement: File 1 — Datasets A0=1A adatoms_backbonds k=0.5. [file Beilstein_J_Nanotechnol-07-937-s001.zip › S1/A0=1A/adatoms_backbonds/k=0.5/results/df_0014.png]

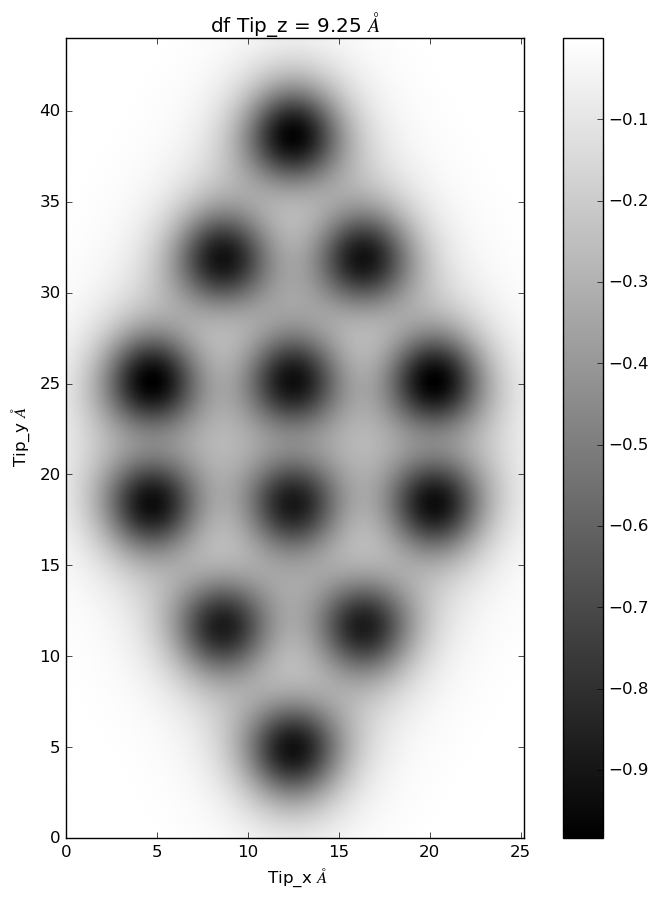

Supplement: File 1 — Datasets A0=1A adatoms_backbonds k=0.5. [file Beilstein_J_Nanotechnol-07-937-s001.zip › S1/A0=1A/adatoms_backbonds/k=0.5/results/df_0015.png]

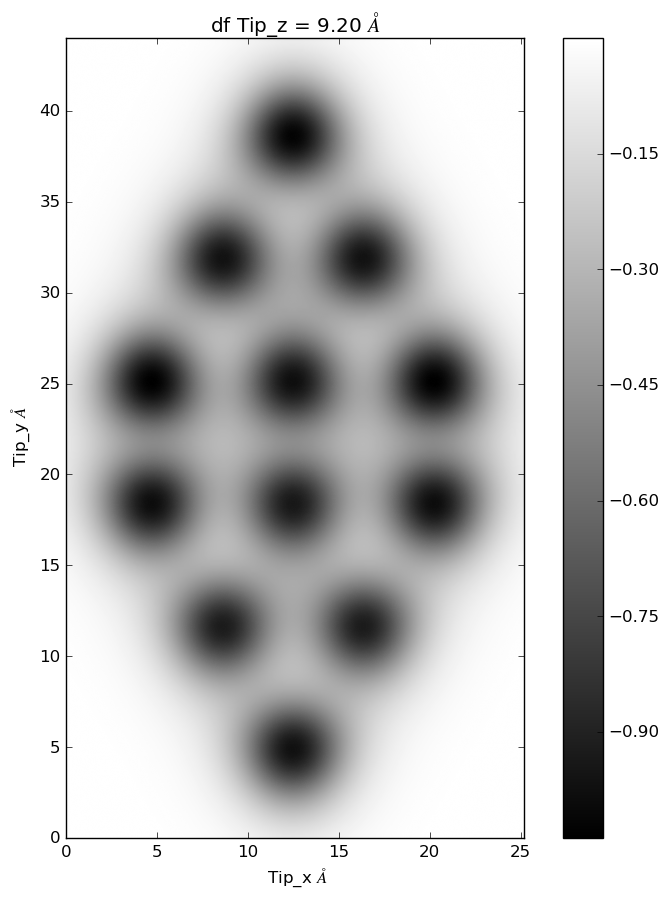

Supplement: File 1 — Datasets A0=1A adatoms_backbonds k=0.5. [file Beilstein_J_Nanotechnol-07-937-s001.zip › S1/A0=1A/adatoms_backbonds/k=0.5/results/df_0016.png]

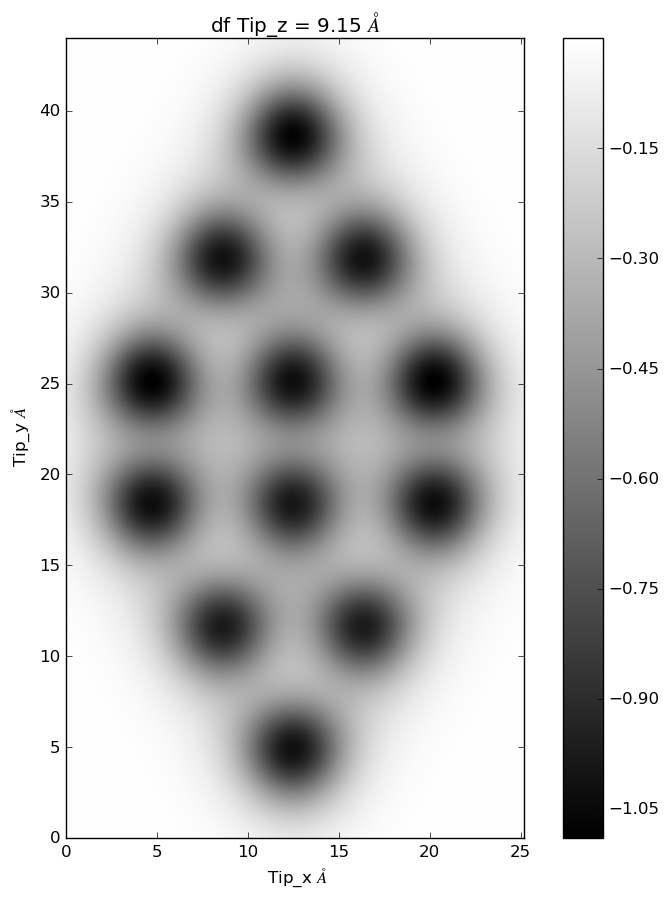

Supplement: File 1 — Datasets A0=1A adatoms_backbonds k=0.5. [file Beilstein_J_Nanotechnol-07-937-s001.zip › S1/A0=1A/adatoms_backbonds/k=0.5/results/df_0017.png]

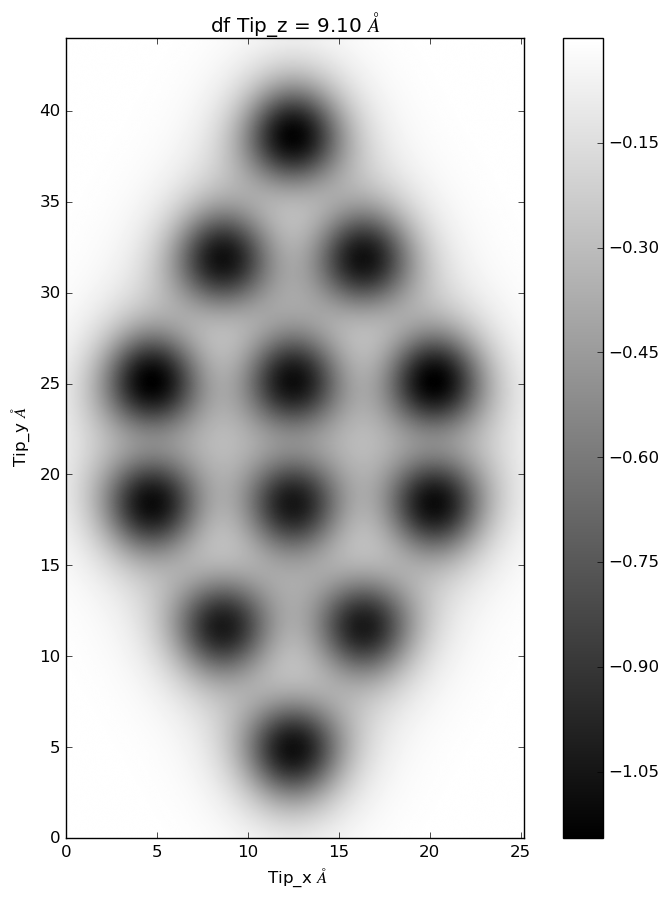

Supplement: File 1 — Datasets A0=1A adatoms_backbonds k=0.5. [file Beilstein_J_Nanotechnol-07-937-s001.zip › S1/A0=1A/adatoms_backbonds/k=0.5/results/df_0018.png]

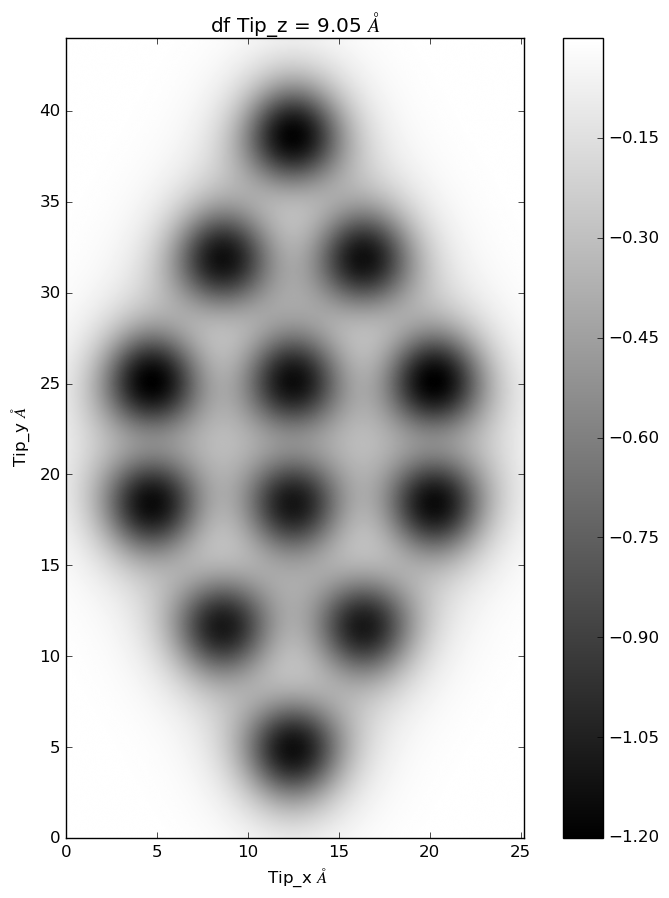

Supplement: File 1 — Datasets A0=1A adatoms_backbonds k=0.5. [file Beilstein_J_Nanotechnol-07-937-s001.zip › S1/A0=1A/adatoms_backbonds/k=0.5/results/df_0019.png]

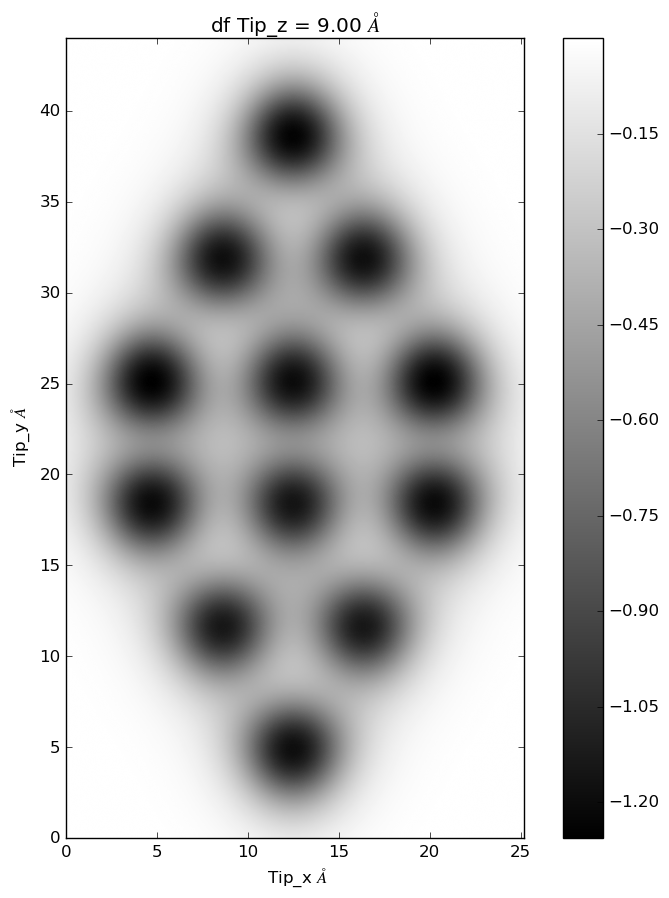

Supplement: File 1 — Datasets A0=1A adatoms_backbonds k=0.5. [file Beilstein_J_Nanotechnol-07-937-s001.zip › S1/A0=1A/adatoms_backbonds/k=0.5/results/df_0020.png]

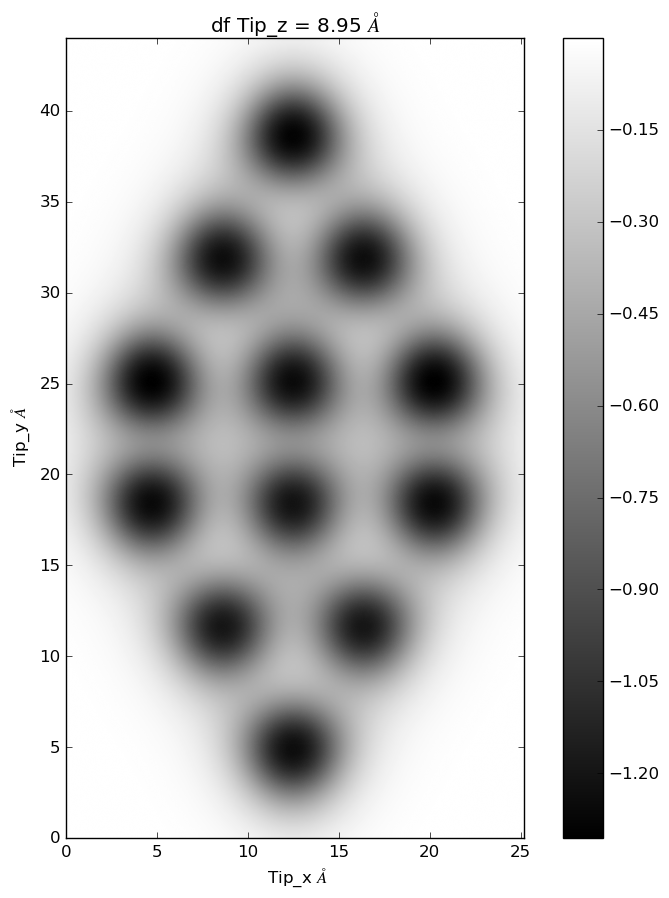

Supplement: File 1 — Datasets A0=1A adatoms_backbonds k=0.5. [file Beilstein_J_Nanotechnol-07-937-s001.zip › S1/A0=1A/adatoms_backbonds/k=0.5/results/df_0021.png]

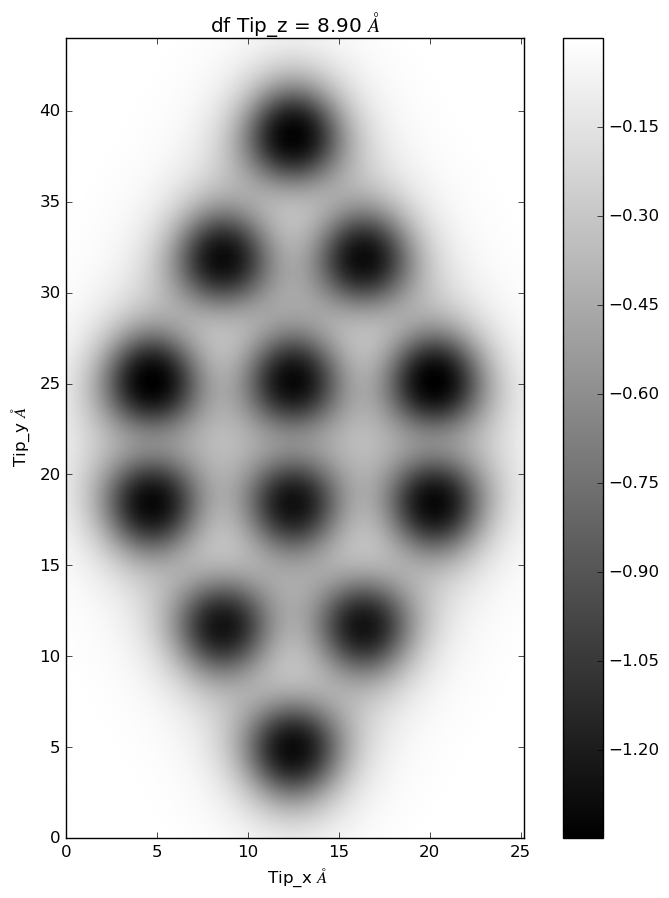

Supplement: File 1 — Datasets A0=1A adatoms_backbonds k=0.5. [file Beilstein_J_Nanotechnol-07-937-s001.zip › S1/A0=1A/adatoms_backbonds/k=0.5/results/df_0022.png]

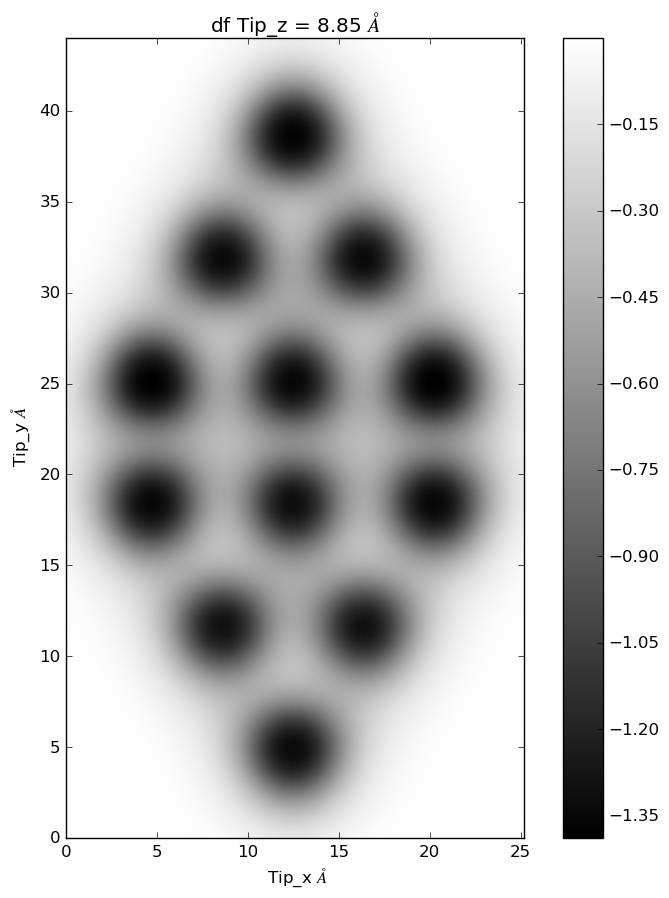

Supplement: File 1 — Datasets A0=1A adatoms_backbonds k=0.5. [file Beilstein_J_Nanotechnol-07-937-s001.zip › S1/A0=1A/adatoms_backbonds/k=0.5/results/df_0023.png]

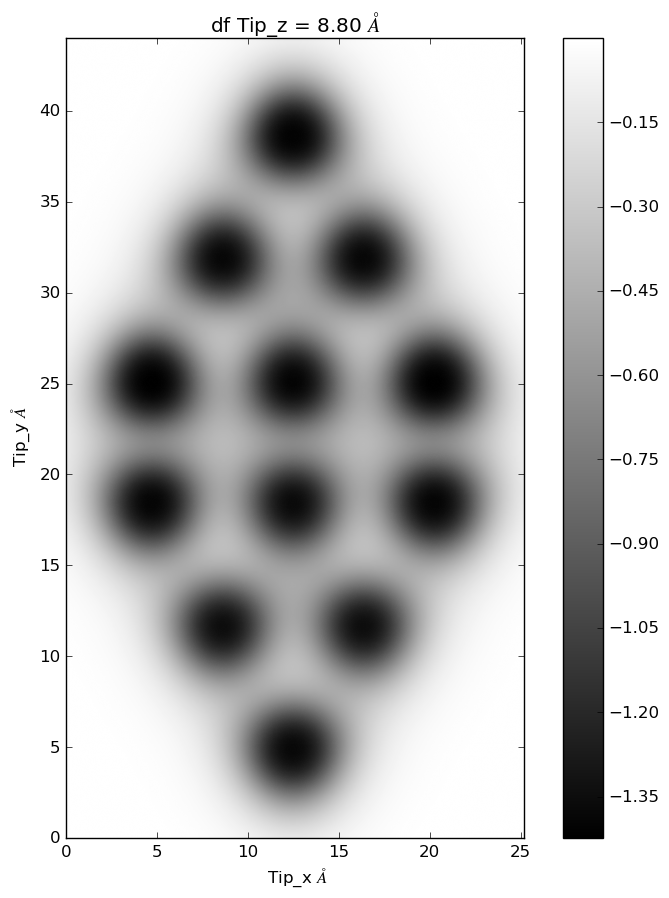

Supplement: File 1 — Datasets A0=1A adatoms_backbonds k=0.5. [file Beilstein_J_Nanotechnol-07-937-s001.zip › S1/A0=1A/adatoms_backbonds/k=0.5/results/df_0024.png]

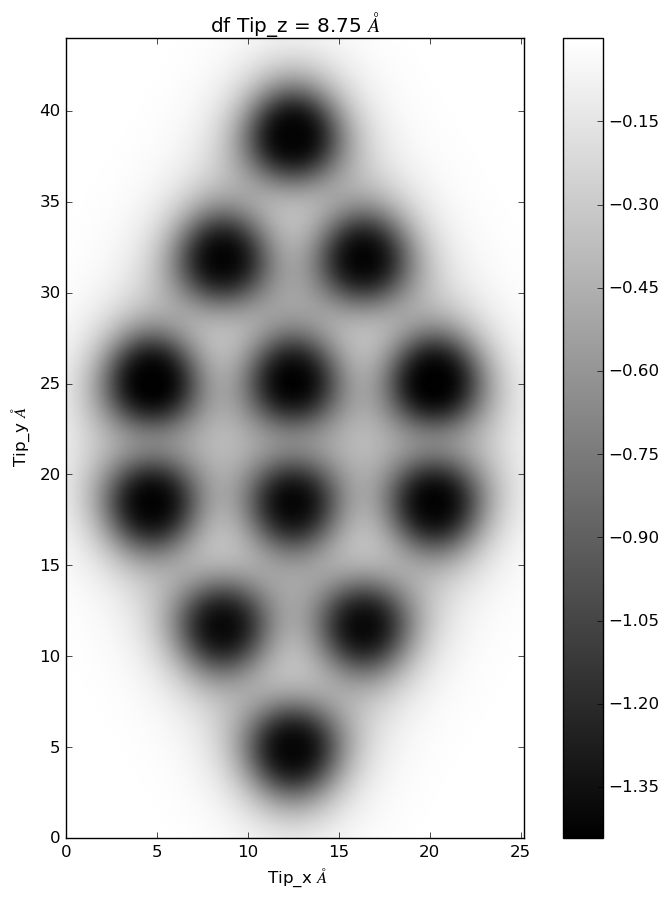

Supplement: File 1 — Datasets A0=1A adatoms_backbonds k=0.5. [file Beilstein_J_Nanotechnol-07-937-s001.zip › S1/A0=1A/adatoms_backbonds/k=0.5/results/df_0025.png]

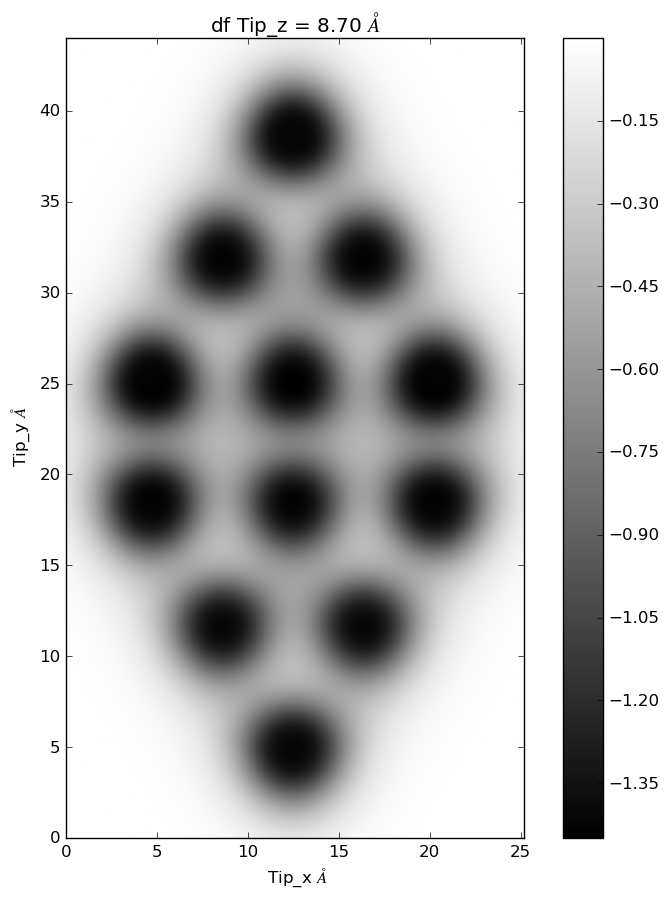

Supplement: File 1 — Datasets A0=1A adatoms_backbonds k=0.5. [file Beilstein_J_Nanotechnol-07-937-s001.zip › S1/A0=1A/adatoms_backbonds/k=0.5/results/df_0026.png]

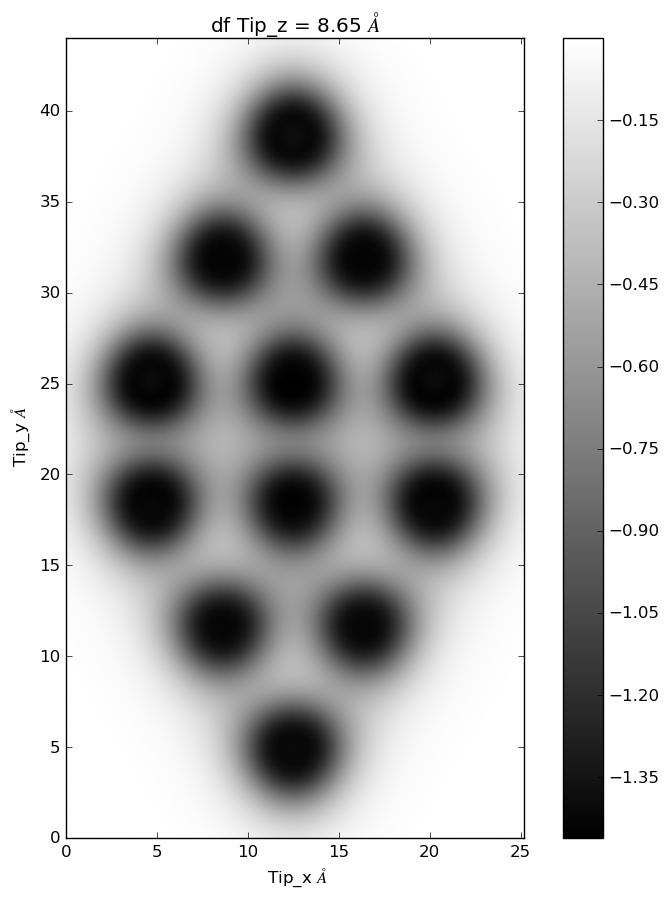

Supplement: File 1 — Datasets A0=1A adatoms_backbonds k=0.5. [file Beilstein_J_Nanotechnol-07-937-s001.zip › S1/A0=1A/adatoms_backbonds/k=0.5/results/df_0027.png]

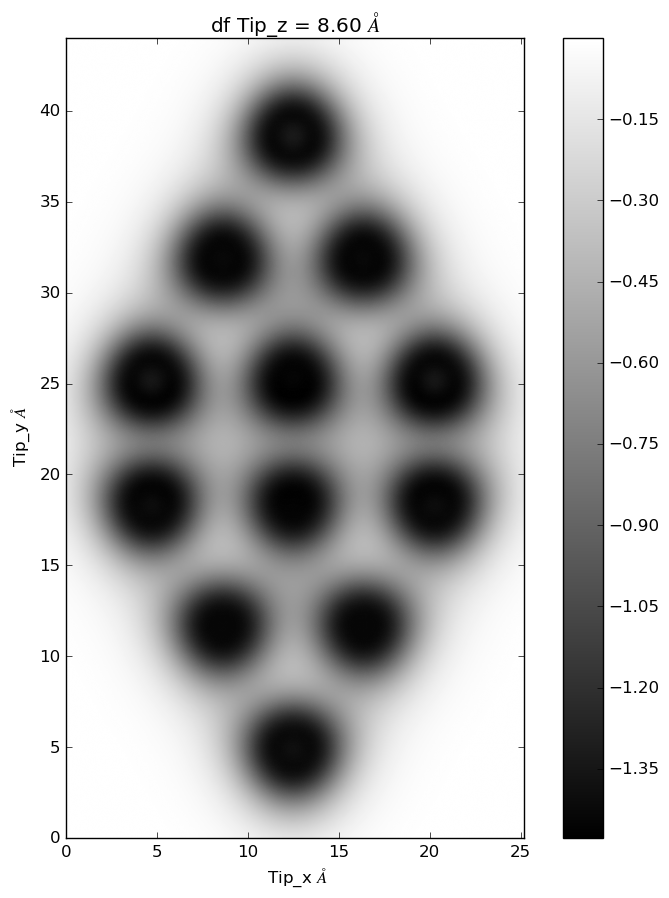

Supplement: File 1 — Datasets A0=1A adatoms_backbonds k=0.5. [file Beilstein_J_Nanotechnol-07-937-s001.zip › S1/A0=1A/adatoms_backbonds/k=0.5/results/df_0028.png]

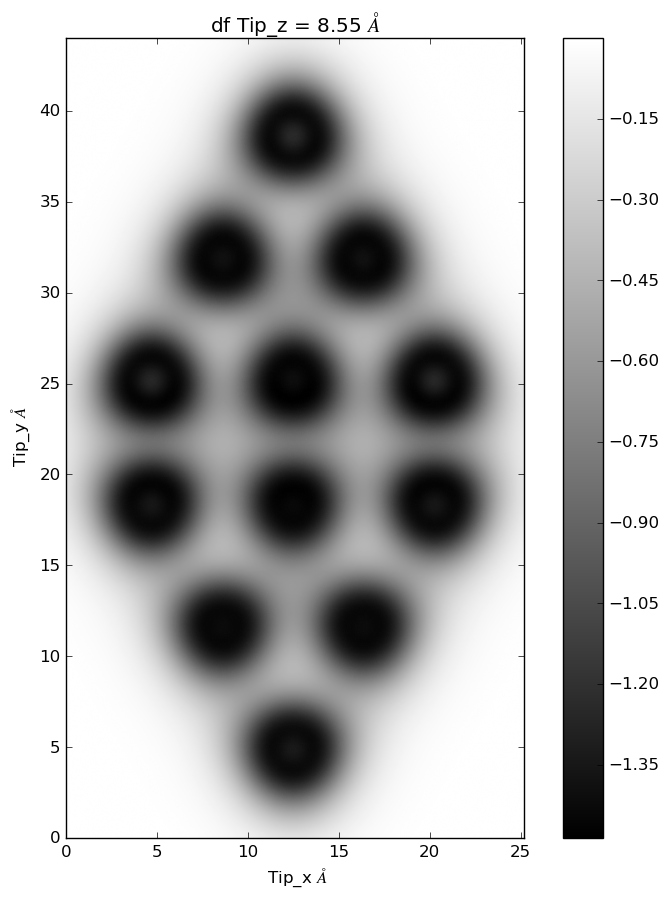

Supplement: File 1 — Datasets A0=1A adatoms_backbonds k=0.5. [file Beilstein_J_Nanotechnol-07-937-s001.zip › S1/A0=1A/adatoms_backbonds/k=0.5/results/df_0029.png]

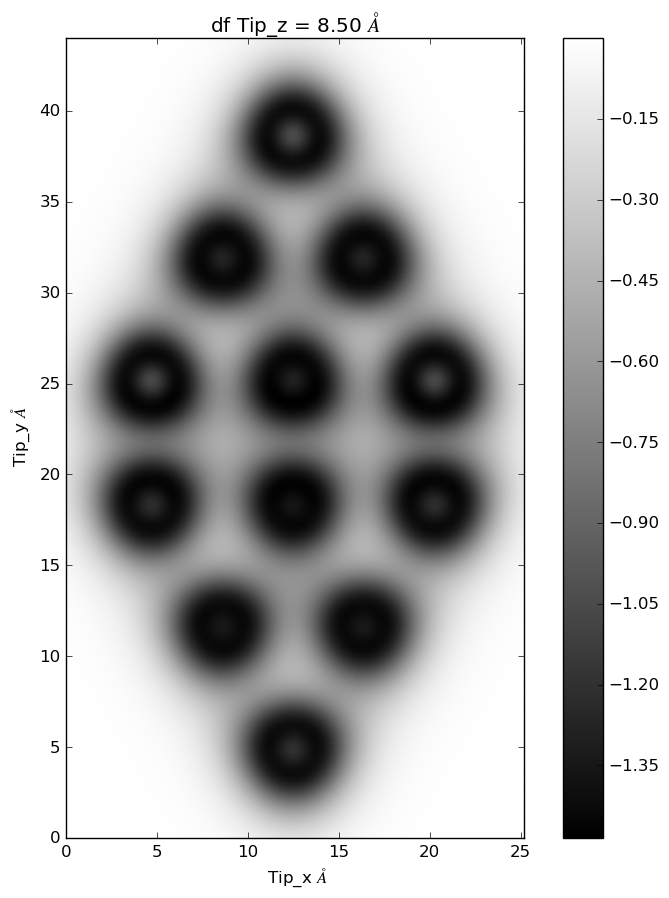

Supplement: File 1 — Datasets A0=1A adatoms_backbonds k=0.5. [file Beilstein_J_Nanotechnol-07-937-s001.zip › S1/A0=1A/adatoms_backbonds/k=0.5/results/df_0030.png]

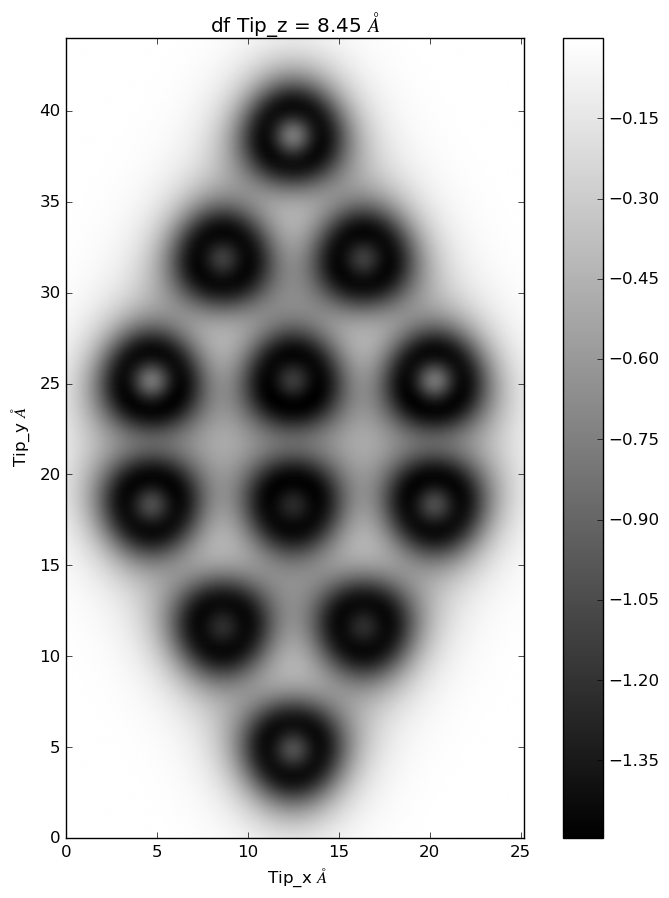

Supplement: File 1 — Datasets A0=1A adatoms_backbonds k=0.5. [file Beilstein_J_Nanotechnol-07-937-s001.zip › S1/A0=1A/adatoms_backbonds/k=0.5/results/df_0031.png]

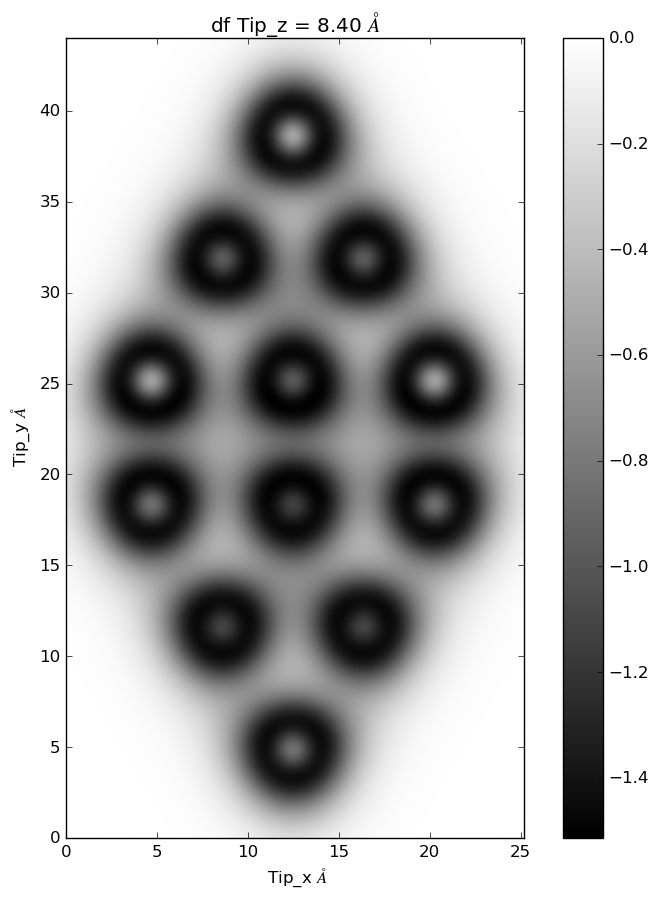

Supplement: File 1 — Datasets A0=1A adatoms_backbonds k=0.5. [file Beilstein_J_Nanotechnol-07-937-s001.zip › S1/A0=1A/adatoms_backbonds/k=0.5/results/df_0032.png]

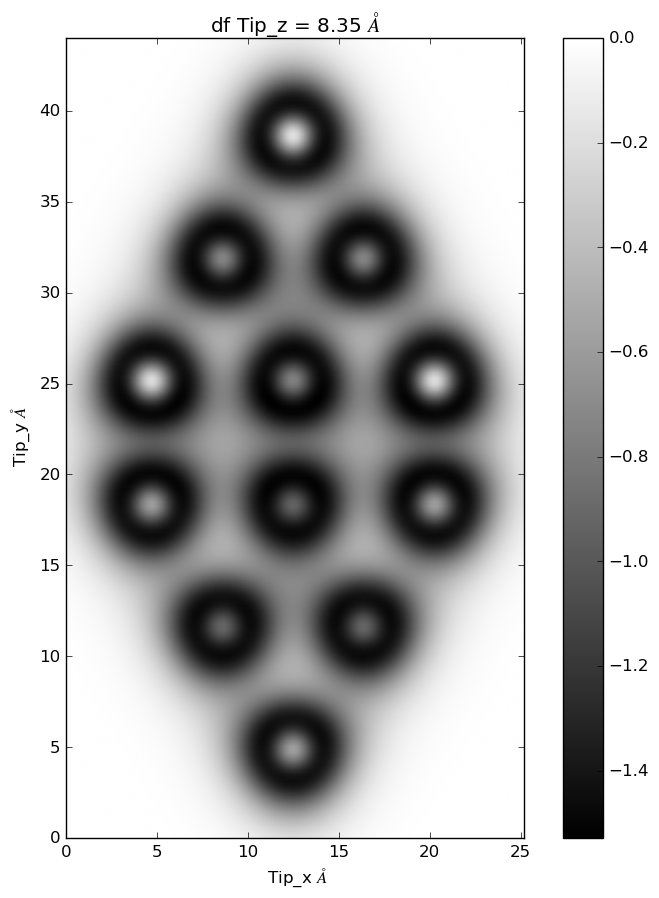

Supplement: File 1 — Datasets A0=1A adatoms_backbonds k=0.5. [file Beilstein_J_Nanotechnol-07-937-s001.zip › S1/A0=1A/adatoms_backbonds/k=0.5/results/df_0033.png]

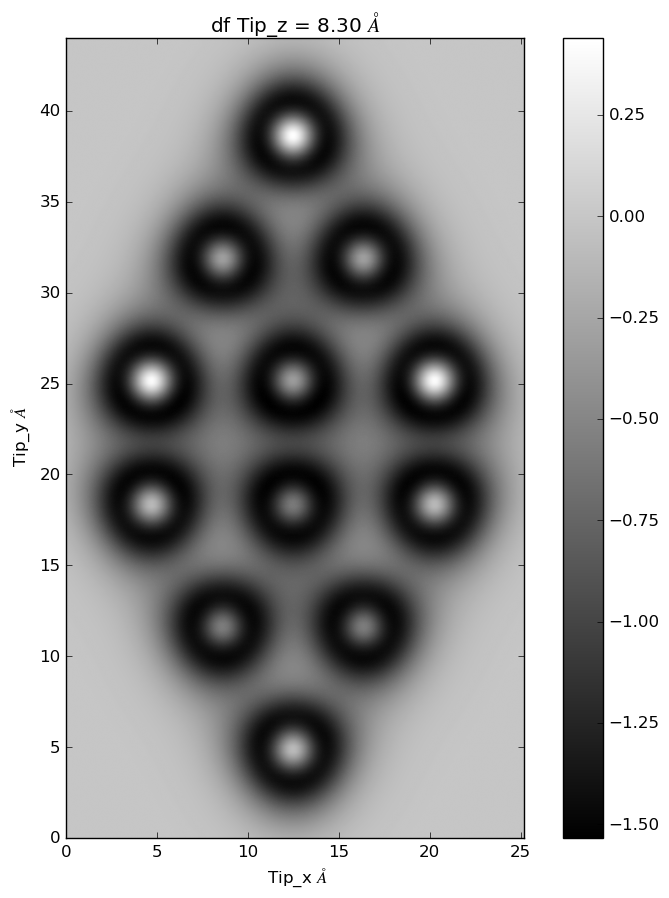

Supplement: File 1 — Datasets A0=1A adatoms_backbonds k=0.5. [file Beilstein_J_Nanotechnol-07-937-s001.zip › S1/A0=1A/adatoms_backbonds/k=0.5/results/df_0034.png]

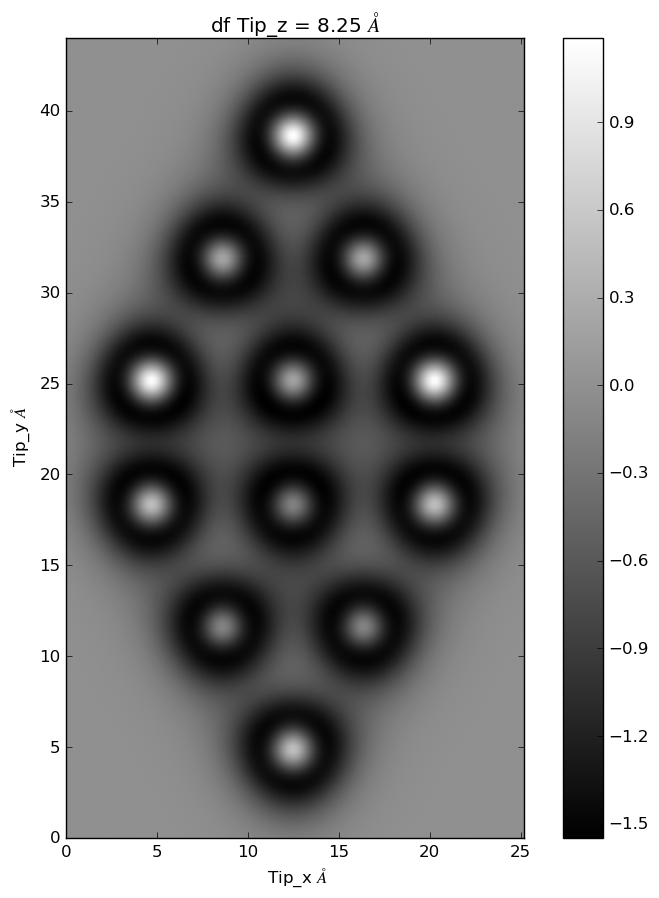

Supplement: File 1 — Datasets A0=1A adatoms_backbonds k=0.5. [file Beilstein_J_Nanotechnol-07-937-s001.zip › S1/A0=1A/adatoms_backbonds/k=0.5/results/df_0035.png]

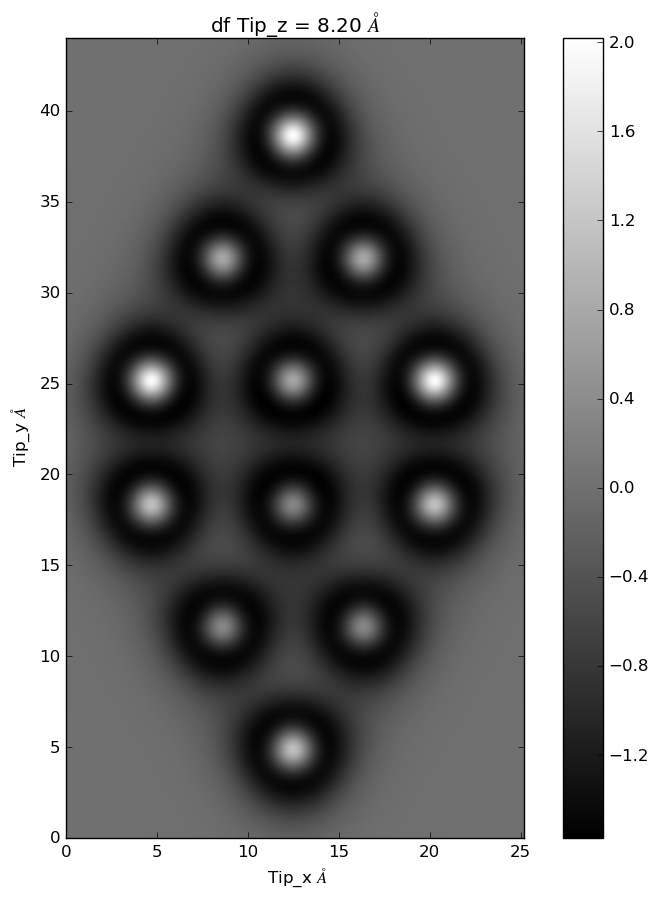

Supplement: File 1 — Datasets A0=1A adatoms_backbonds k=0.5. [file Beilstein_J_Nanotechnol-07-937-s001.zip › S1/A0=1A/adatoms_backbonds/k=0.5/results/df_0036.png]

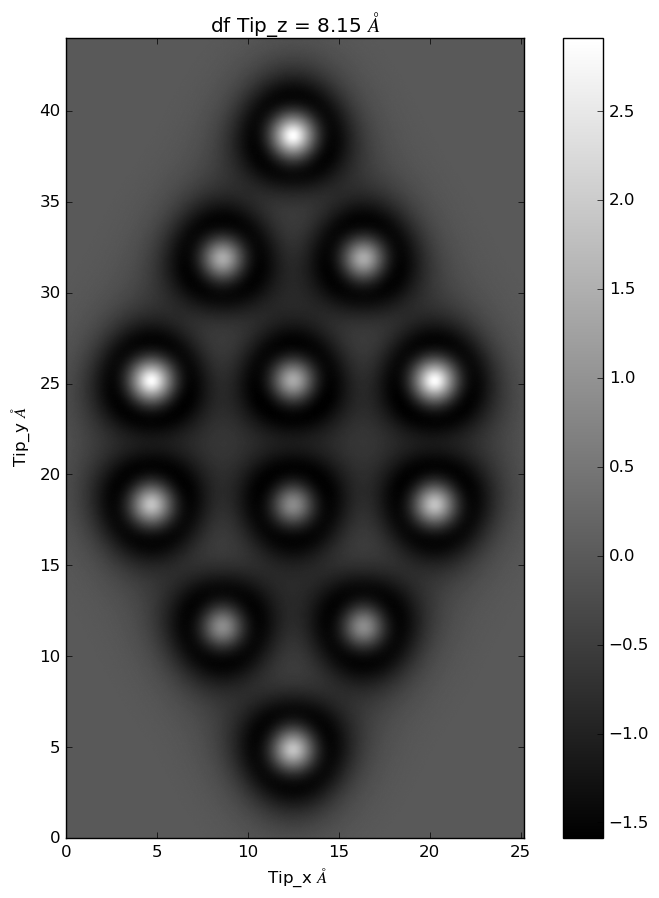

Supplement: File 1 — Datasets A0=1A adatoms_backbonds k=0.5. [file Beilstein_J_Nanotechnol-07-937-s001.zip › S1/A0=1A/adatoms_backbonds/k=0.5/results/df_0037.png]

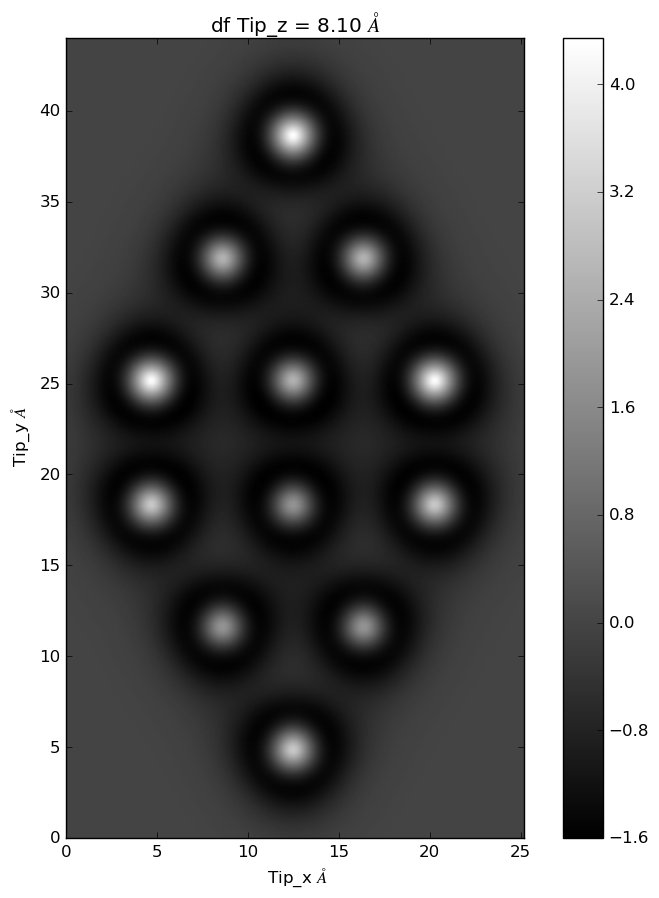

Supplement: File 1 — Datasets A0=1A adatoms_backbonds k=0.5. [file Beilstein_J_Nanotechnol-07-937-s001.zip › S1/A0=1A/adatoms_backbonds/k=0.5/results/df_0038.png]

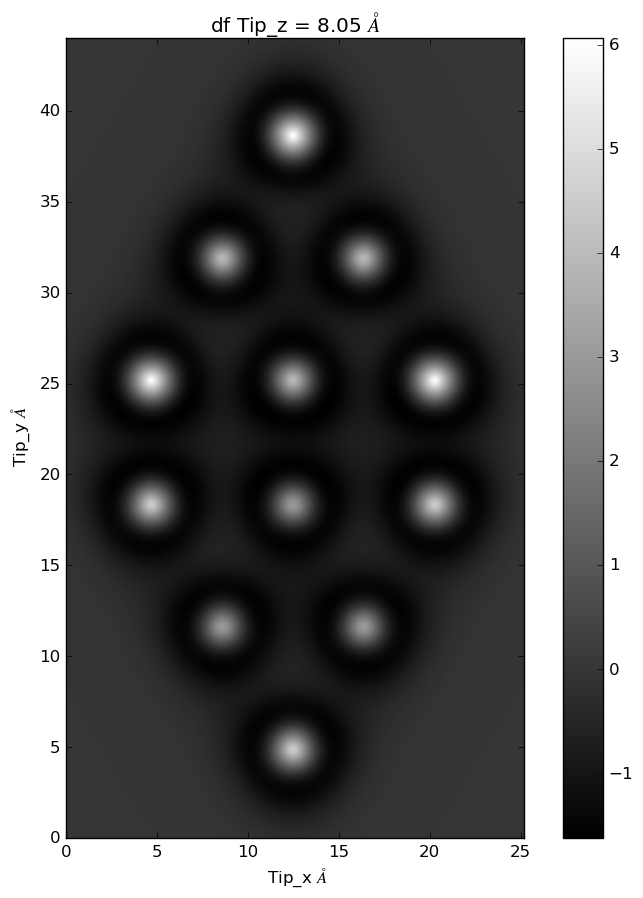

Supplement: File 1 — Datasets A0=1A adatoms_backbonds k=0.5. [file Beilstein_J_Nanotechnol-07-937-s001.zip › S1/A0=1A/adatoms_backbonds/k=0.5/results/df_0039.png]

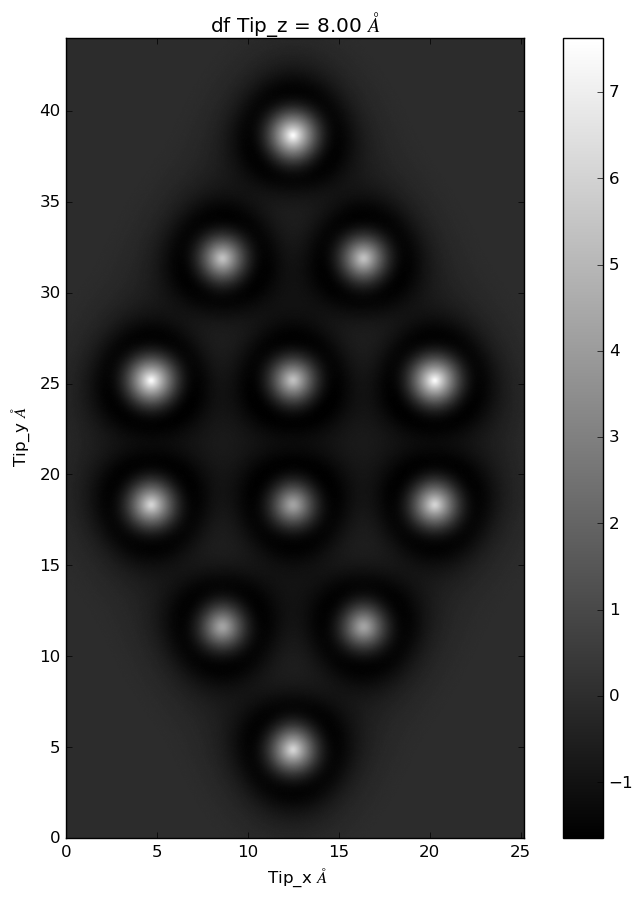

Supplement: File 1 — Datasets A0=1A adatoms_backbonds k=0.5. [file Beilstein_J_Nanotechnol-07-937-s001.zip › S1/A0=1A/adatoms_backbonds/k=0.5/results/df_0040.png]

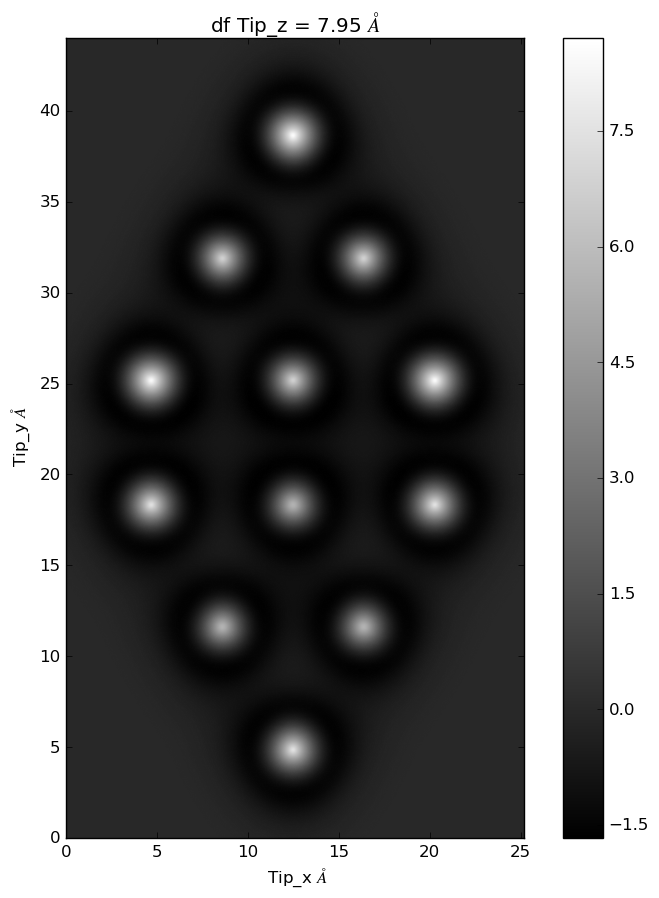

Supplement: File 1 — Datasets A0=1A adatoms_backbonds k=0.5. [file Beilstein_J_Nanotechnol-07-937-s001.zip › S1/A0=1A/adatoms_backbonds/k=0.5/results/df_0041.png]

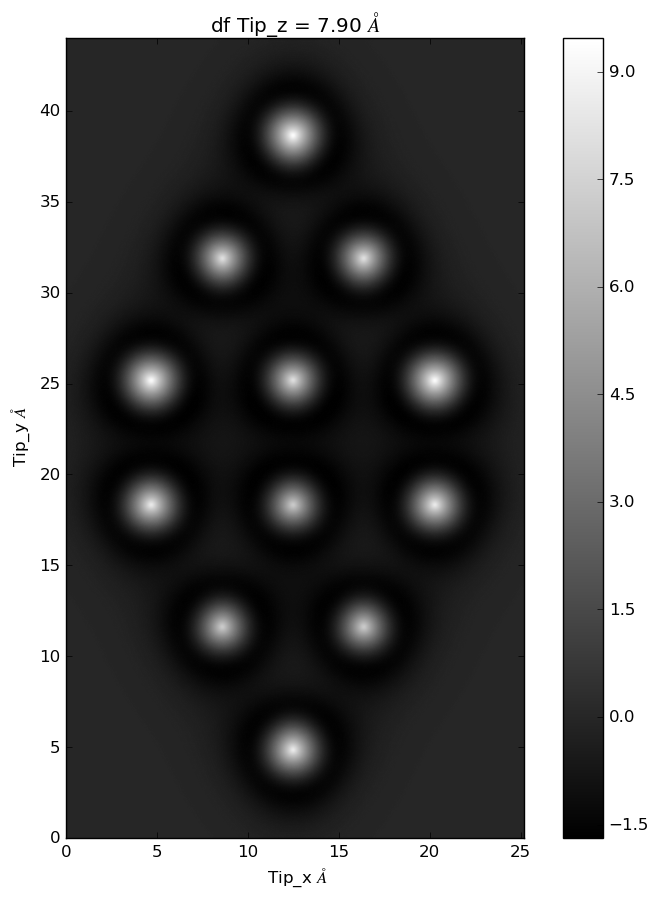

Supplement: File 1 — Datasets A0=1A adatoms_backbonds k=0.5. [file Beilstein_J_Nanotechnol-07-937-s001.zip › S1/A0=1A/adatoms_backbonds/k=0.5/results/df_0042.png]

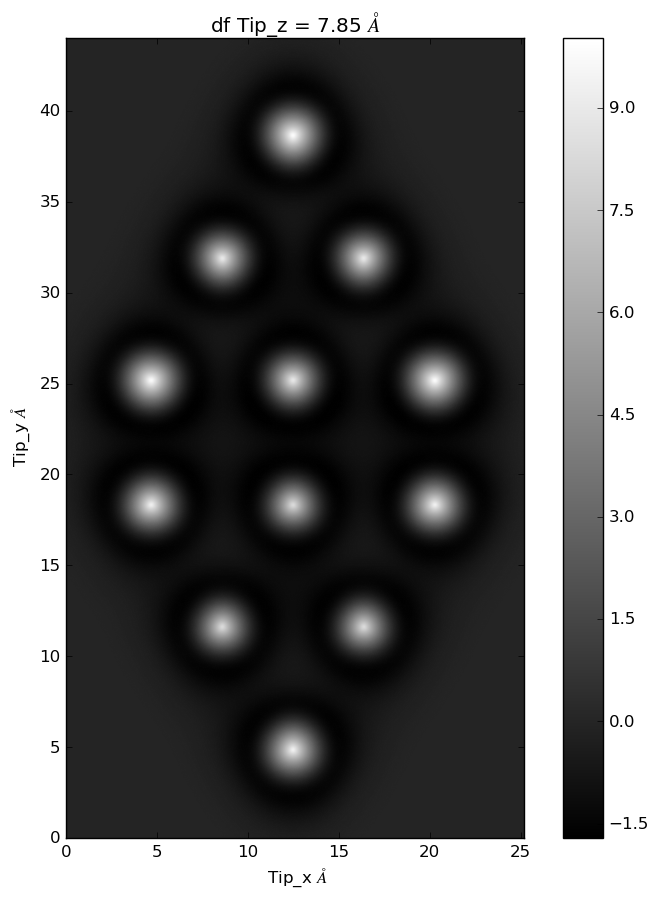

Supplement: File 1 — Datasets A0=1A adatoms_backbonds k=0.5. [file Beilstein_J_Nanotechnol-07-937-s001.zip › S1/A0=1A/adatoms_backbonds/k=0.5/results/df_0043.png]

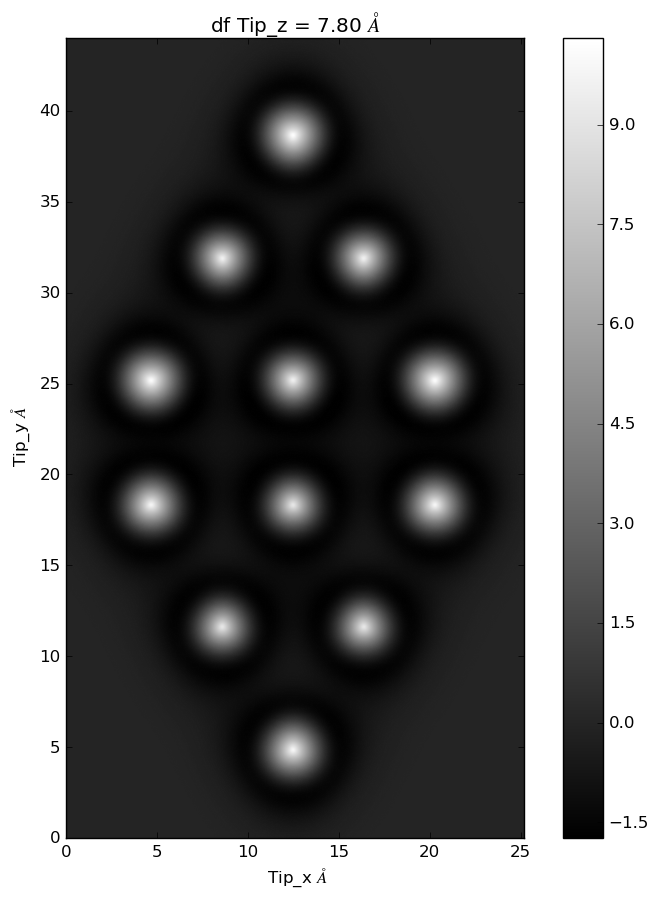

Supplement: File 1 — Datasets A0=1A adatoms_backbonds k=0.5. [file Beilstein_J_Nanotechnol-07-937-s001.zip › S1/A0=1A/adatoms_backbonds/k=0.5/results/df_0044.png]

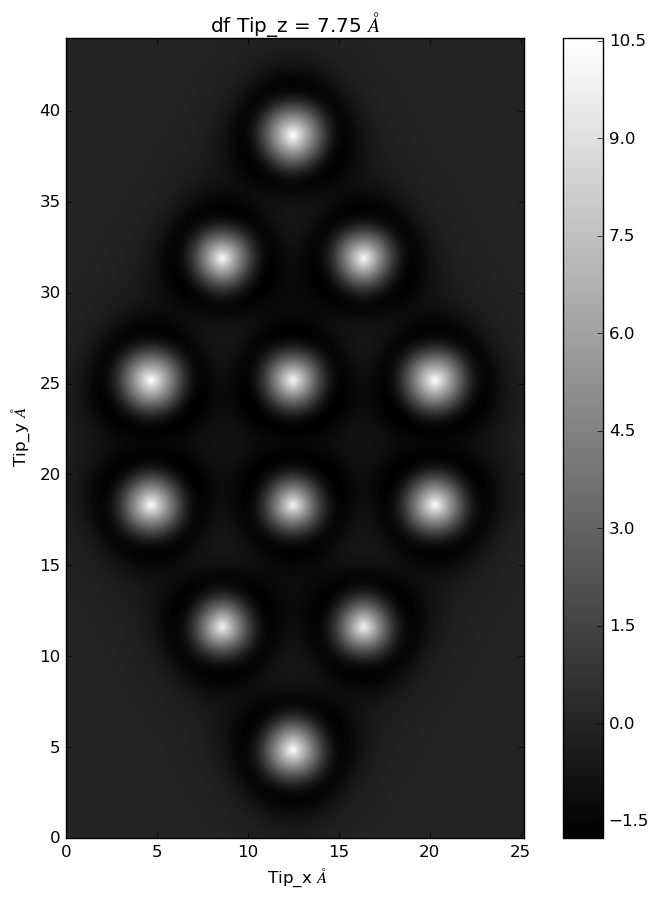

Supplement: File 1 — Datasets A0=1A adatoms_backbonds k=0.5. [file Beilstein_J_Nanotechnol-07-937-s001.zip › S1/A0=1A/adatoms_backbonds/k=0.5/results/df_0045.png]

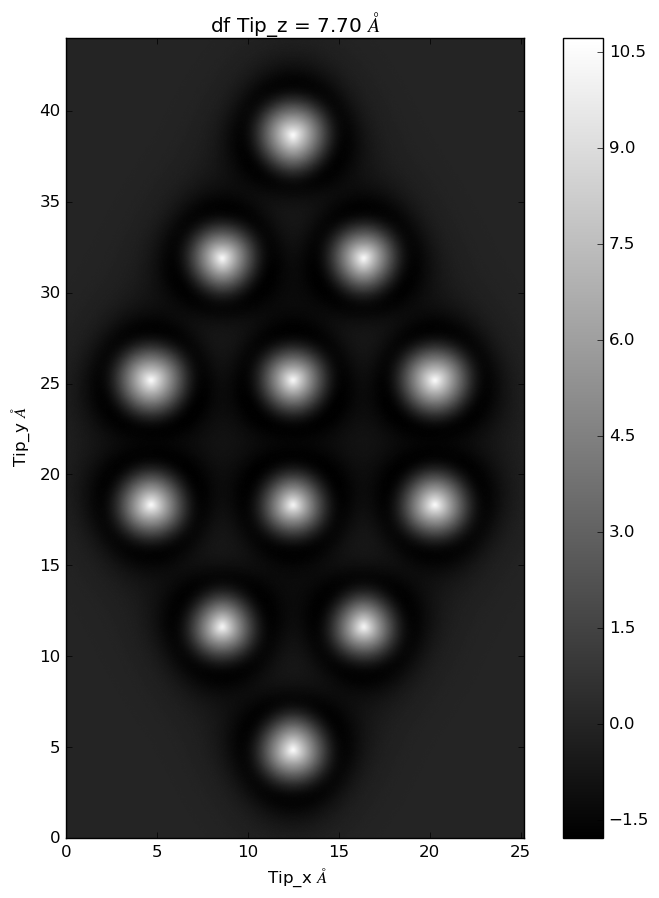

Supplement: File 1 — Datasets A0=1A adatoms_backbonds k=0.5. [file Beilstein_J_Nanotechnol-07-937-s001.zip › S1/A0=1A/adatoms_backbonds/k=0.5/results/df_0046.png]

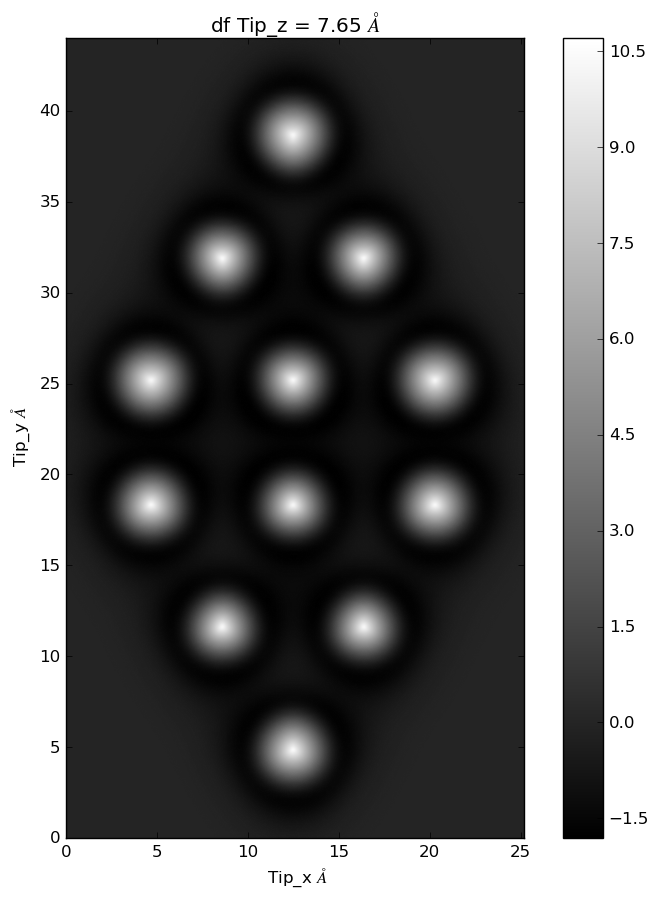

Supplement: File 1 — Datasets A0=1A adatoms_backbonds k=0.5. [file Beilstein_J_Nanotechnol-07-937-s001.zip › S1/A0=1A/adatoms_backbonds/k=0.5/results/df_0047.png]

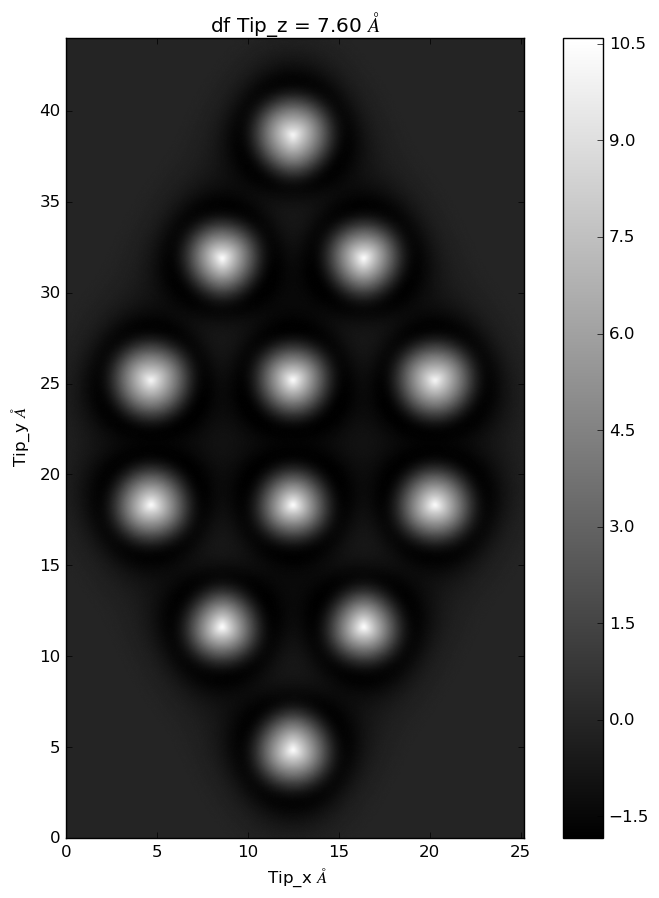

Supplement: File 1 — Datasets A0=1A adatoms_backbonds k=0.5. [file Beilstein_J_Nanotechnol-07-937-s001.zip › S1/A0=1A/adatoms_backbonds/k=0.5/results/df_0048.png]

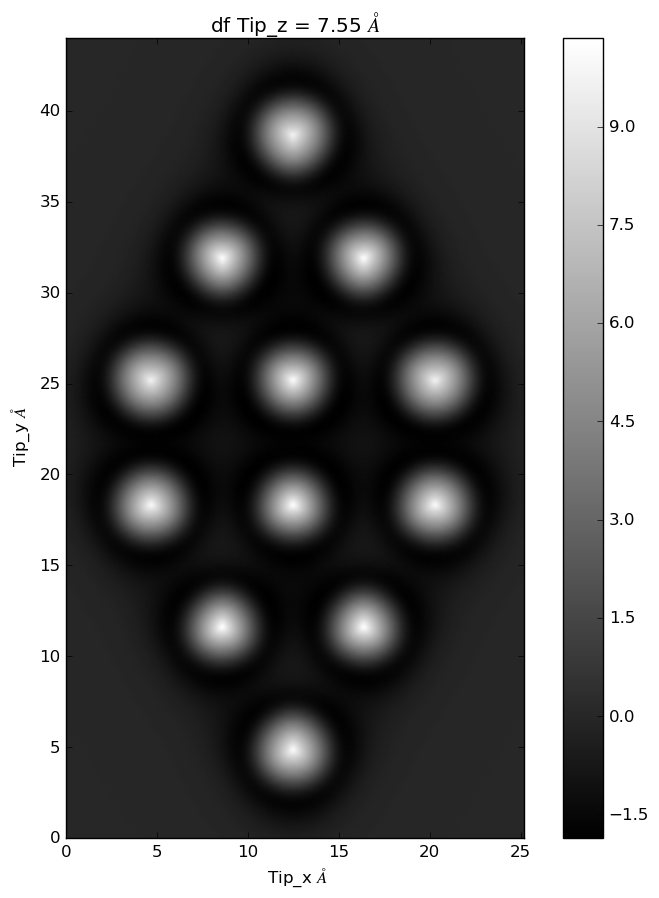

Supplement: File 1 — Datasets A0=1A adatoms_backbonds k=0.5. [file Beilstein_J_Nanotechnol-07-937-s001.zip › S1/A0=1A/adatoms_backbonds/k=0.5/results/df_0049.png]

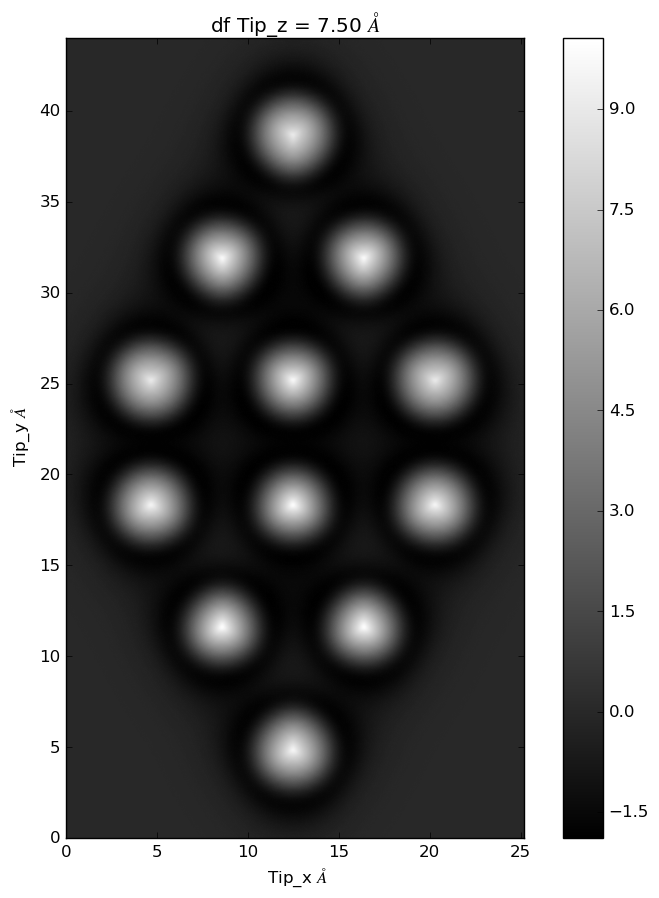

Supplement: File 1 — Datasets A0=1A adatoms_backbonds k=0.5. [file Beilstein_J_Nanotechnol-07-937-s001.zip › S1/A0=1A/adatoms_backbonds/k=0.5/results/df_0050.png]

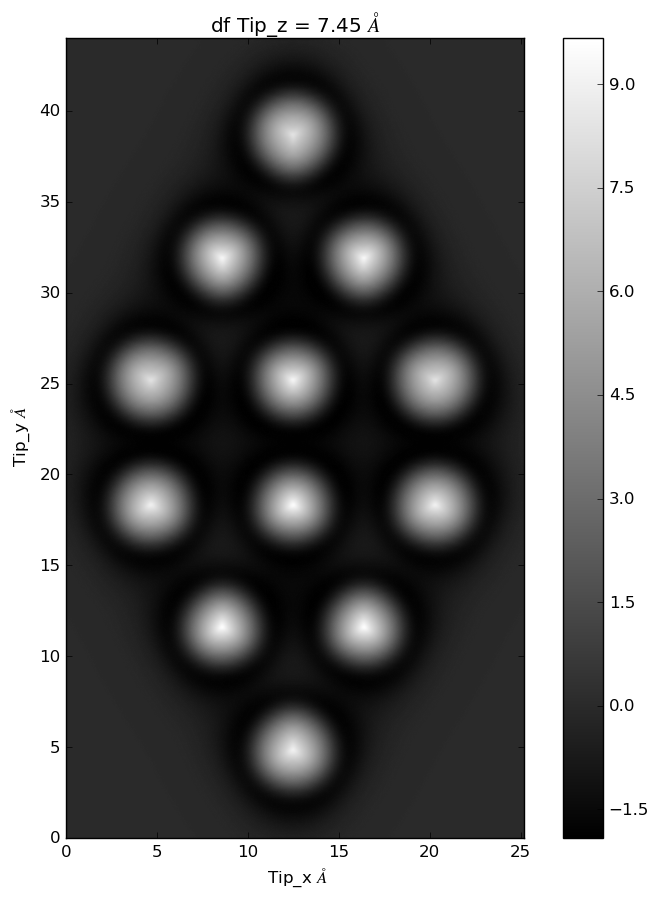

Supplement: File 1 — Datasets A0=1A adatoms_backbonds k=0.5. [file Beilstein_J_Nanotechnol-07-937-s001.zip › S1/A0=1A/adatoms_backbonds/k=0.5/results/df_0051.png]

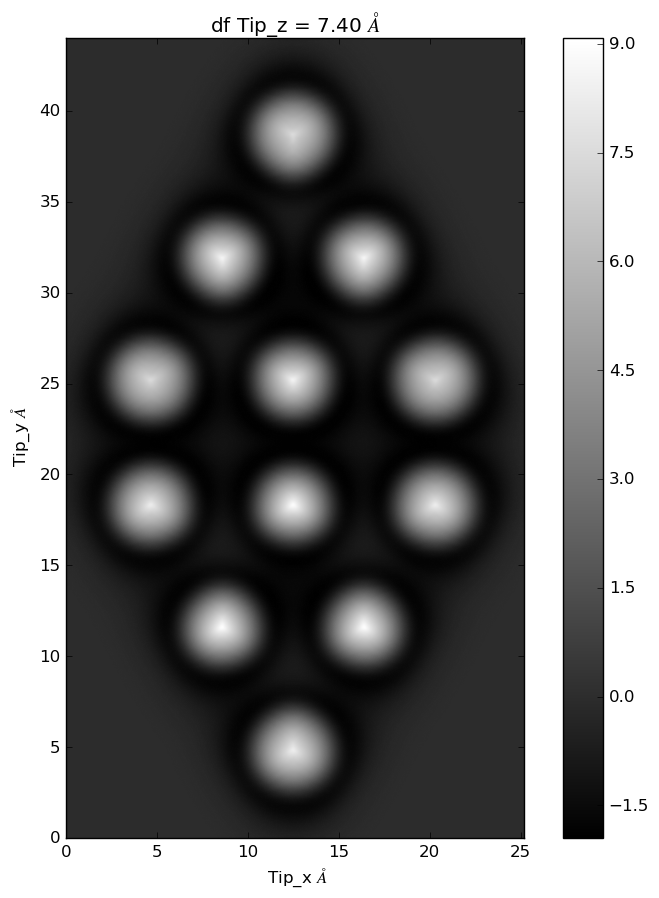

Supplement: File 1 — Datasets A0=1A adatoms_backbonds k=0.5. [file Beilstein_J_Nanotechnol-07-937-s001.zip › S1/A0=1A/adatoms_backbonds/k=0.5/results/df_0052.png]

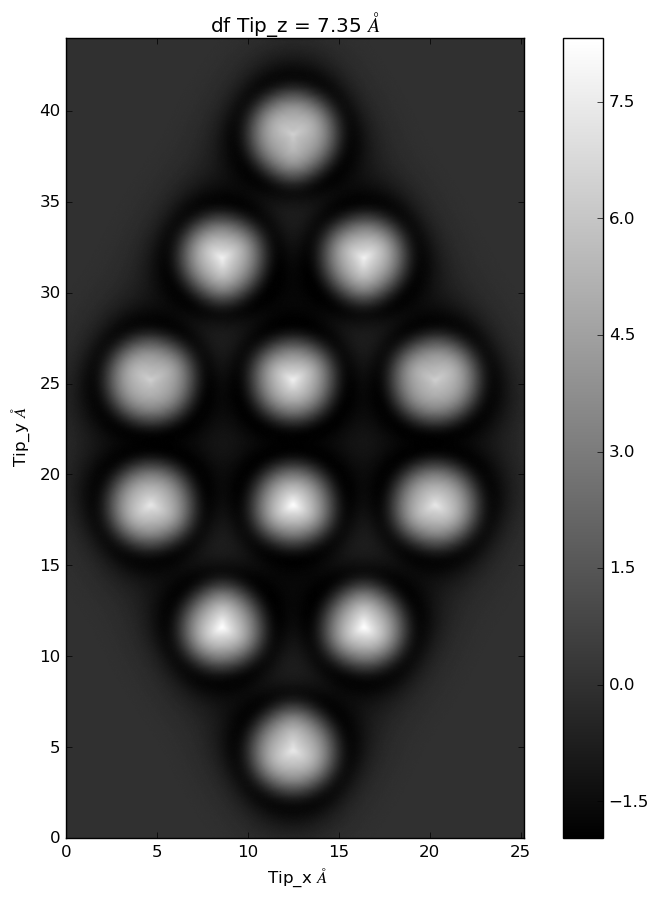

Supplement: File 1 — Datasets A0=1A adatoms_backbonds k=0.5. [file Beilstein_J_Nanotechnol-07-937-s001.zip › S1/A0=1A/adatoms_backbonds/k=0.5/results/df_0053.png]

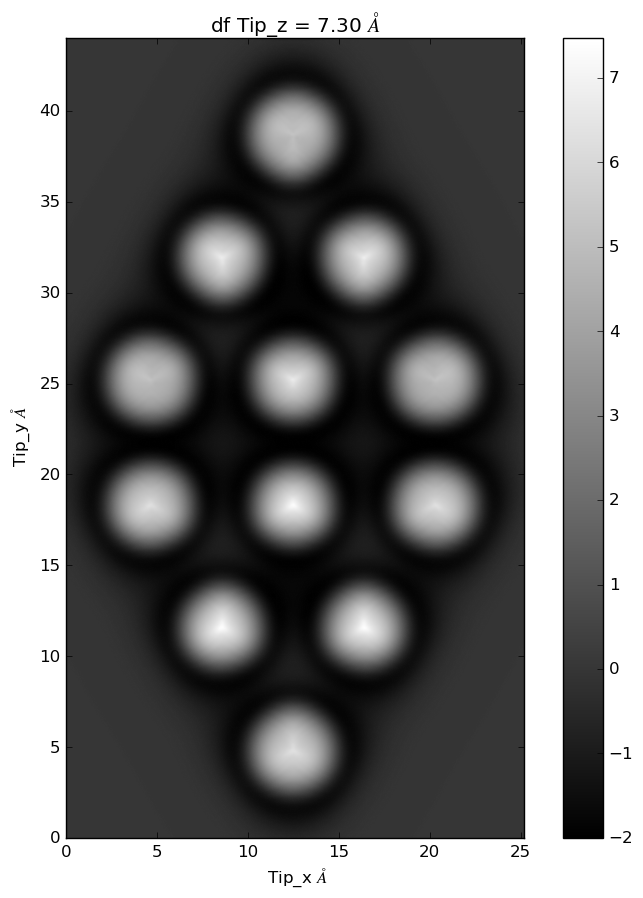

Supplement: File 1 — Datasets A0=1A adatoms_backbonds k=0.5. [file Beilstein_J_Nanotechnol-07-937-s001.zip › S1/A0=1A/adatoms_backbonds/k=0.5/results/df_0054.png]

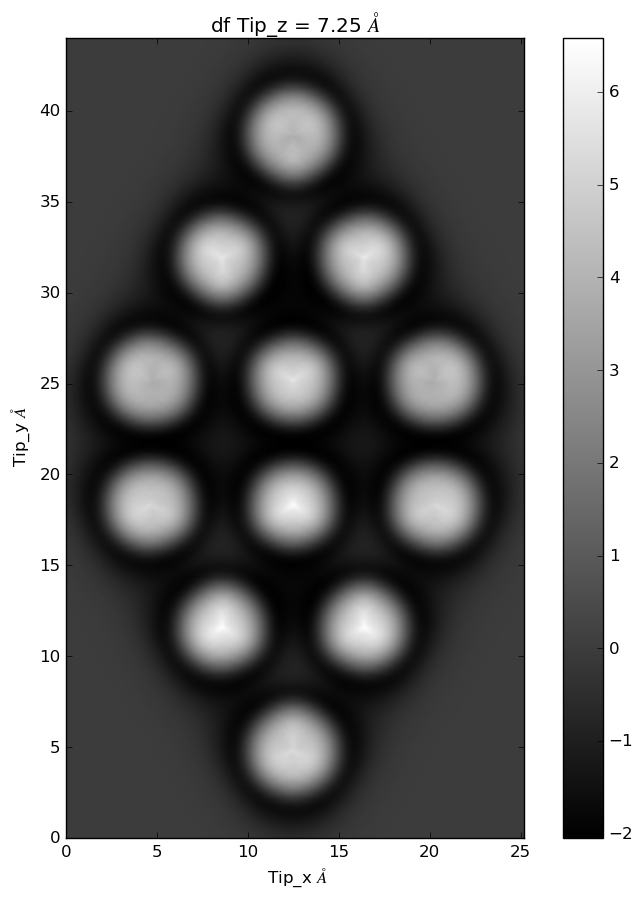

Supplement: File 1 — Datasets A0=1A adatoms_backbonds k=0.5. [file Beilstein_J_Nanotechnol-07-937-s001.zip › S1/A0=1A/adatoms_backbonds/k=0.5/results/df_0055.png]

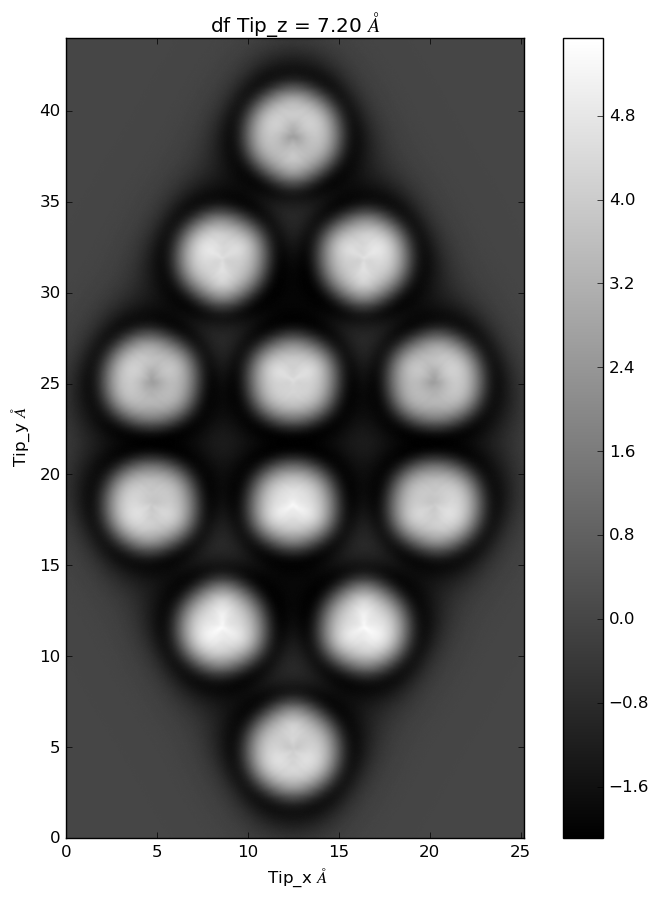

Supplement: File 1 — Datasets A0=1A adatoms_backbonds k=0.5. [file Beilstein_J_Nanotechnol-07-937-s001.zip › S1/A0=1A/adatoms_backbonds/k=0.5/results/df_0056.png]

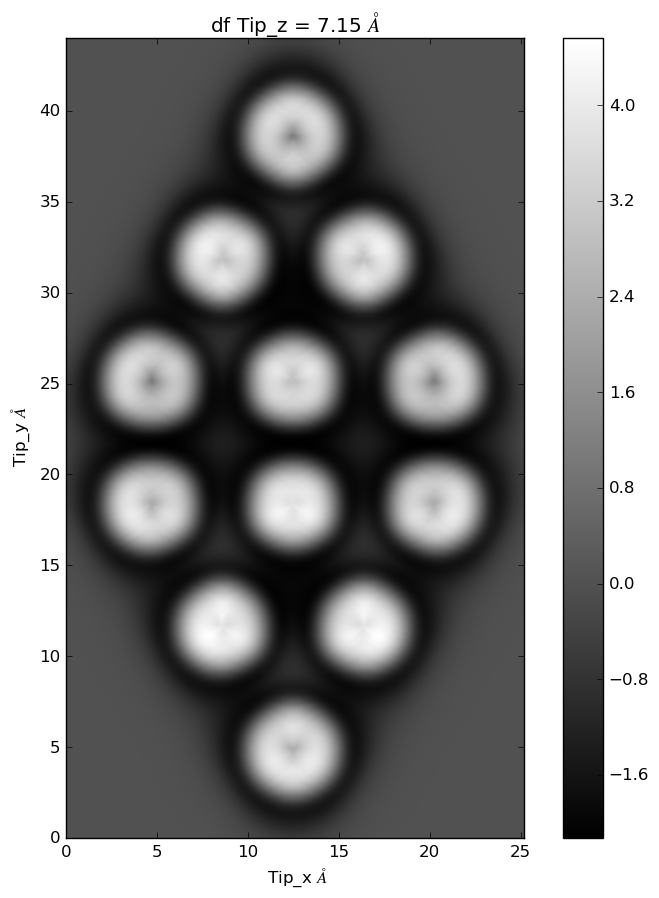

Supplement: File 1 — Datasets A0=1A adatoms_backbonds k=0.5. [file Beilstein_J_Nanotechnol-07-937-s001.zip › S1/A0=1A/adatoms_backbonds/k=0.5/results/df_0057.png]

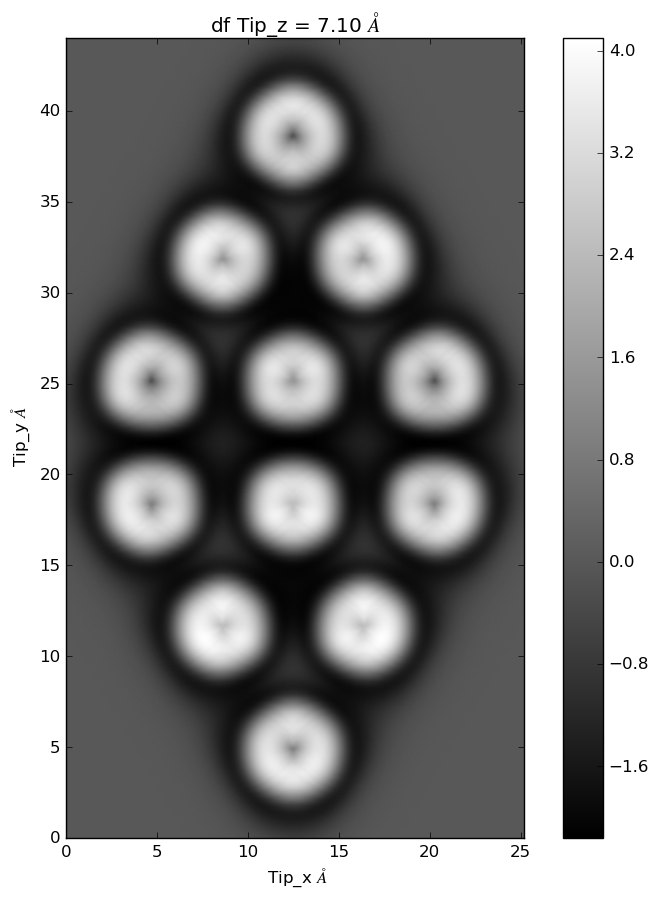

Supplement: File 1 — Datasets A0=1A adatoms_backbonds k=0.5. [file Beilstein_J_Nanotechnol-07-937-s001.zip › S1/A0=1A/adatoms_backbonds/k=0.5/results/df_0058.png]

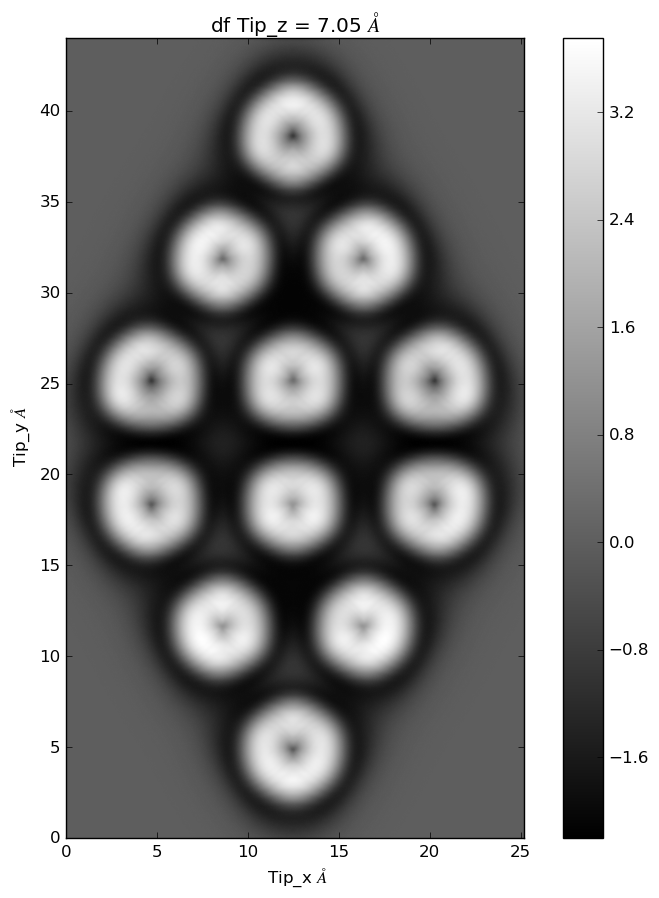

Supplement: File 1 — Datasets A0=1A adatoms_backbonds k=0.5. [file Beilstein_J_Nanotechnol-07-937-s001.zip › S1/A0=1A/adatoms_backbonds/k=0.5/results/df_0059.png]

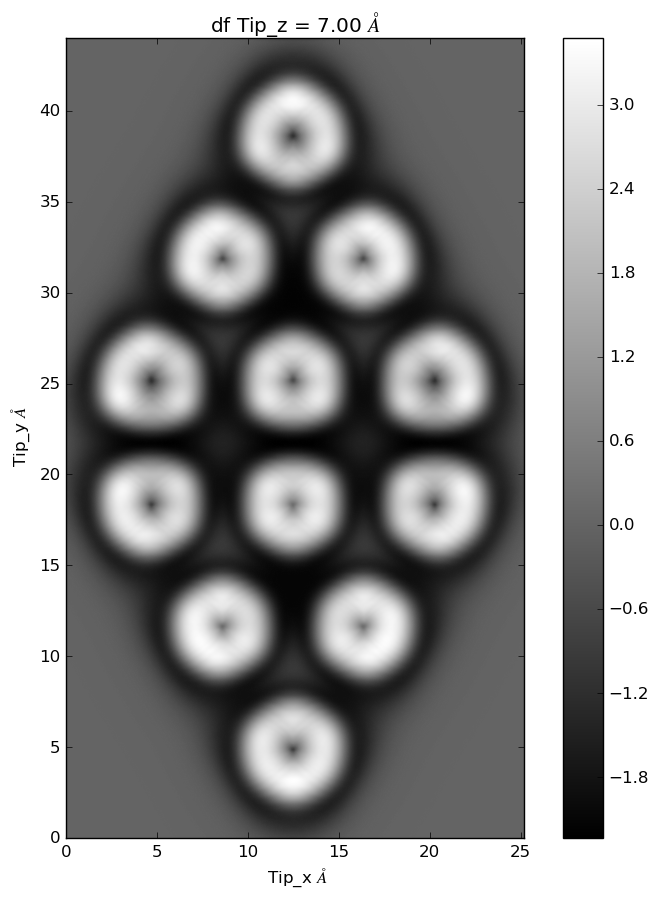

Supplement: File 1 — Datasets A0=1A adatoms_backbonds k=0.5. [file Beilstein_J_Nanotechnol-07-937-s001.zip › S1/A0=1A/adatoms_backbonds/k=0.5/results/df_0060.png]

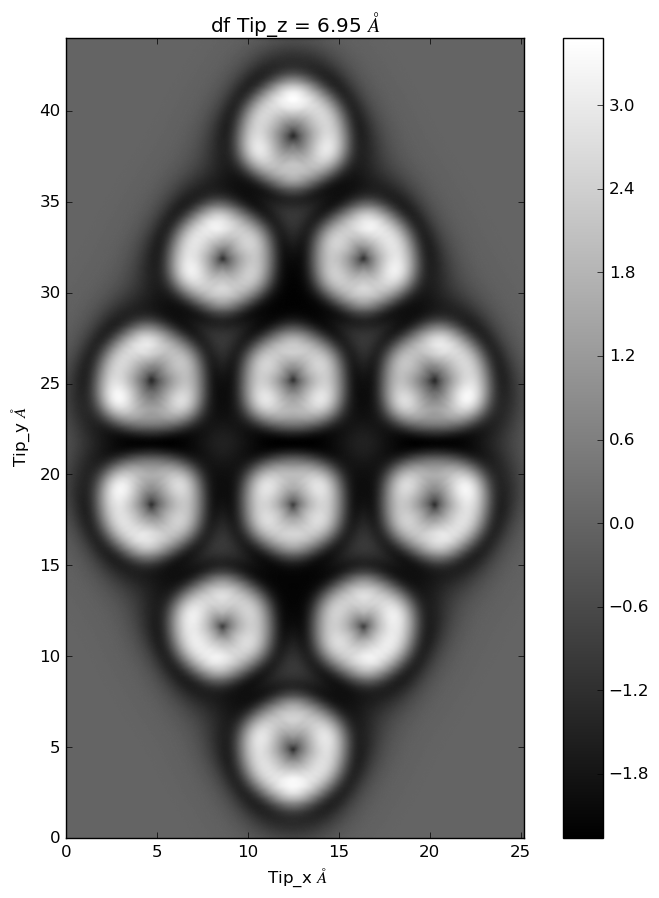

Supplement: File 1 — Datasets A0=1A adatoms_backbonds k=0.5. [file Beilstein_J_Nanotechnol-07-937-s001.zip › S1/A0=1A/adatoms_backbonds/k=0.5/results/df_0061.png]

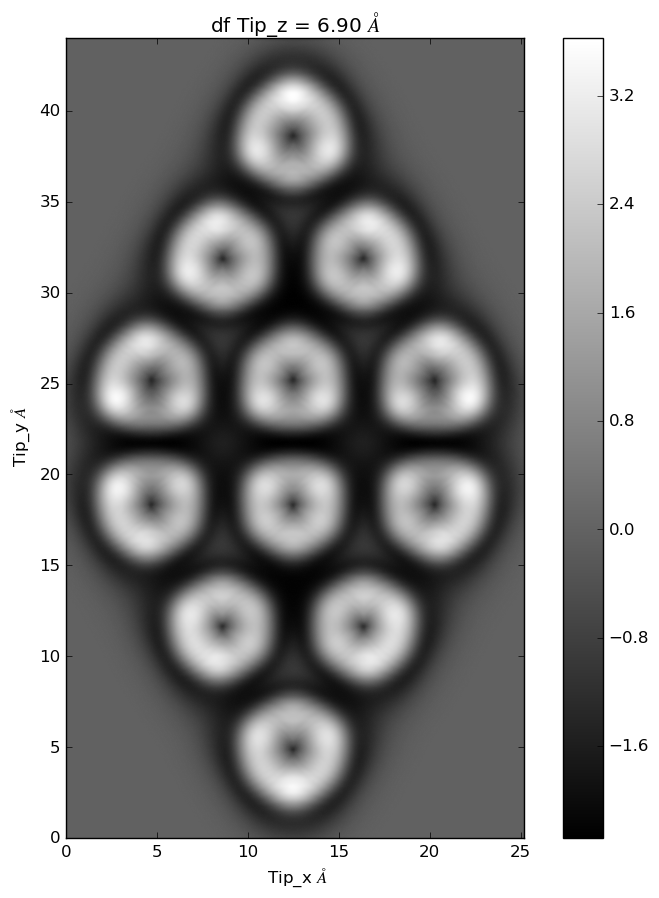

Supplement: File 1 — Datasets A0=1A adatoms_backbonds k=0.5. [file Beilstein_J_Nanotechnol-07-937-s001.zip › S1/A0=1A/adatoms_backbonds/k=0.5/results/df_0062.png]

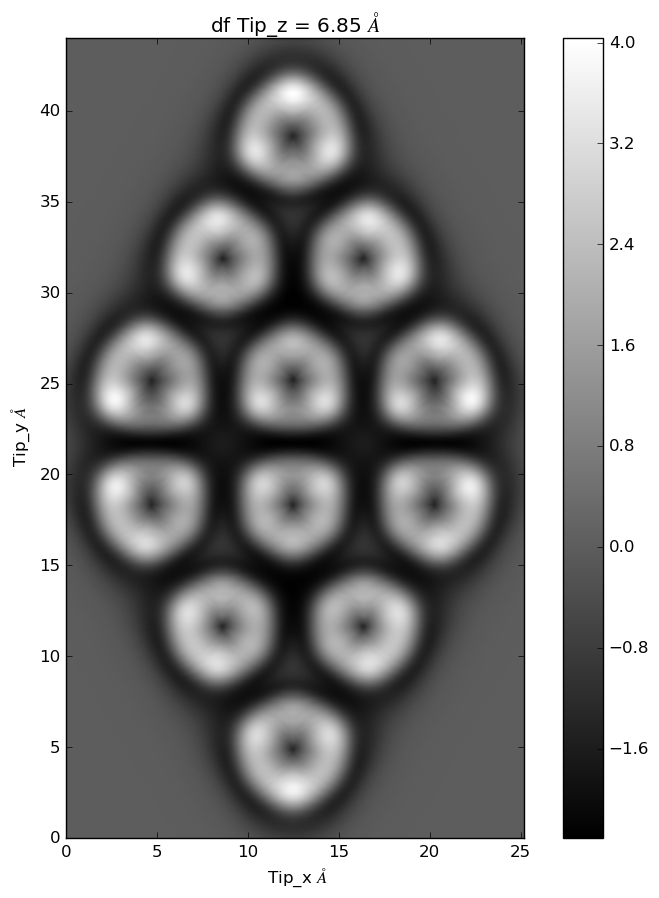

Supplement: File 1 — Datasets A0=1A adatoms_backbonds k=0.5. [file Beilstein_J_Nanotechnol-07-937-s001.zip › S1/A0=1A/adatoms_backbonds/k=0.5/results/df_0063.png]

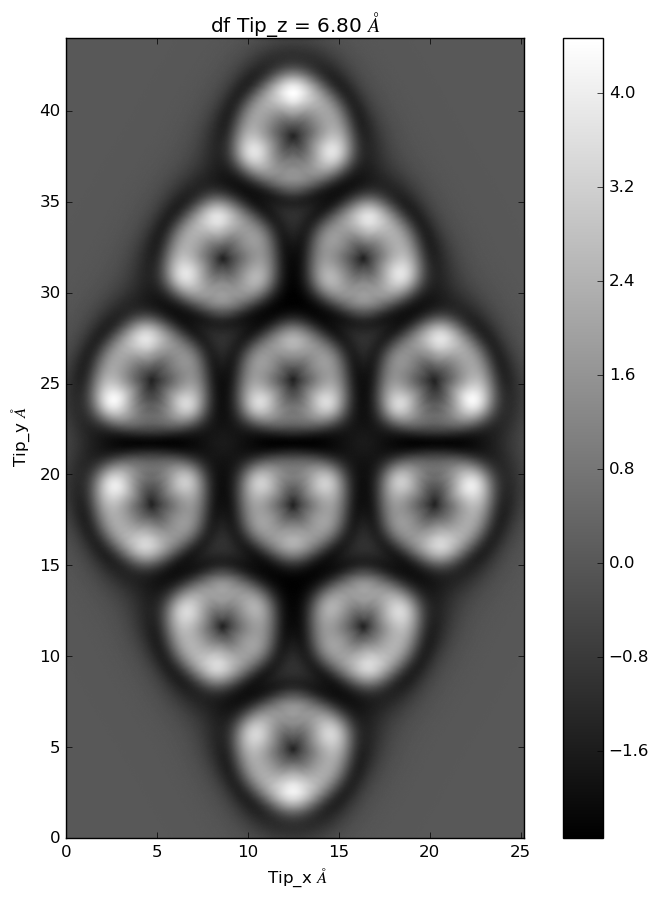

Supplement: File 1 — Datasets A0=1A adatoms_backbonds k=0.5. [file Beilstein_J_Nanotechnol-07-937-s001.zip › S1/A0=1A/adatoms_backbonds/k=0.5/results/df_0064.png]

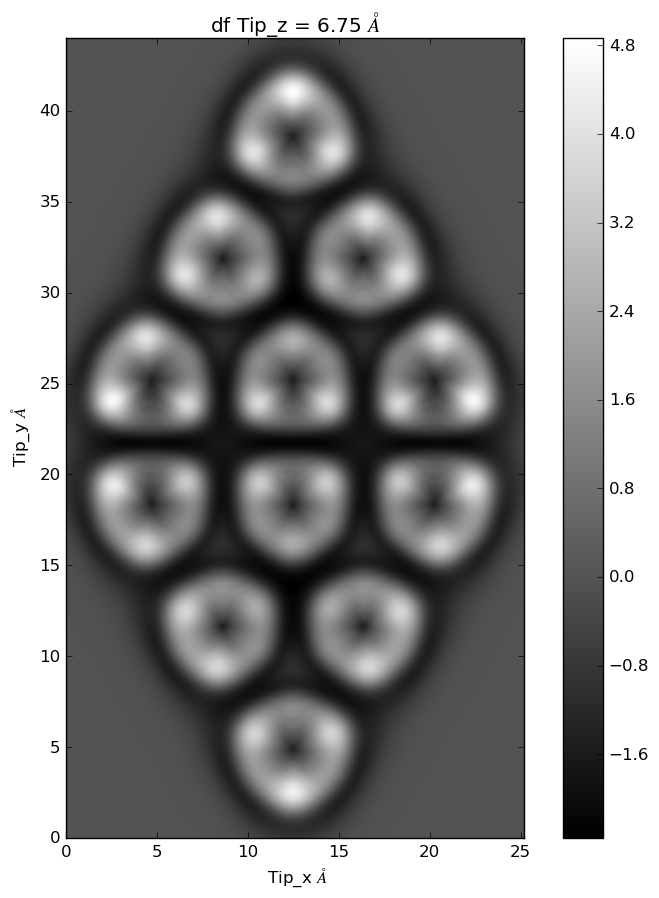

Supplement: File 1 — Datasets A0=1A adatoms_backbonds k=0.5. [file Beilstein_J_Nanotechnol-07-937-s001.zip › S1/A0=1A/adatoms_backbonds/k=0.5/results/df_0065.png]

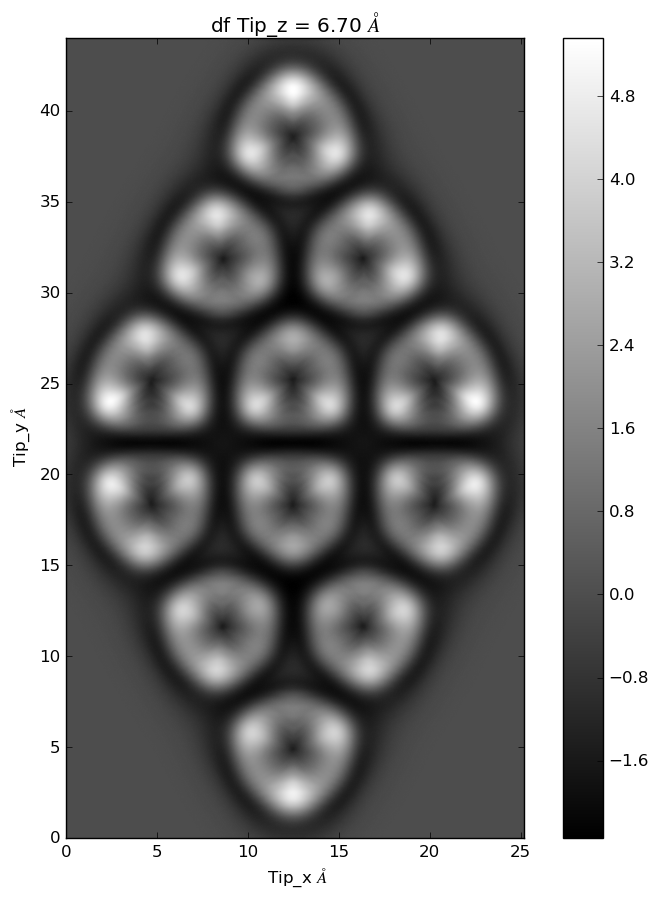

Supplement: File 1 — Datasets A0=1A adatoms_backbonds k=0.5. [file Beilstein_J_Nanotechnol-07-937-s001.zip › S1/A0=1A/adatoms_backbonds/k=0.5/results/df_0066.png]

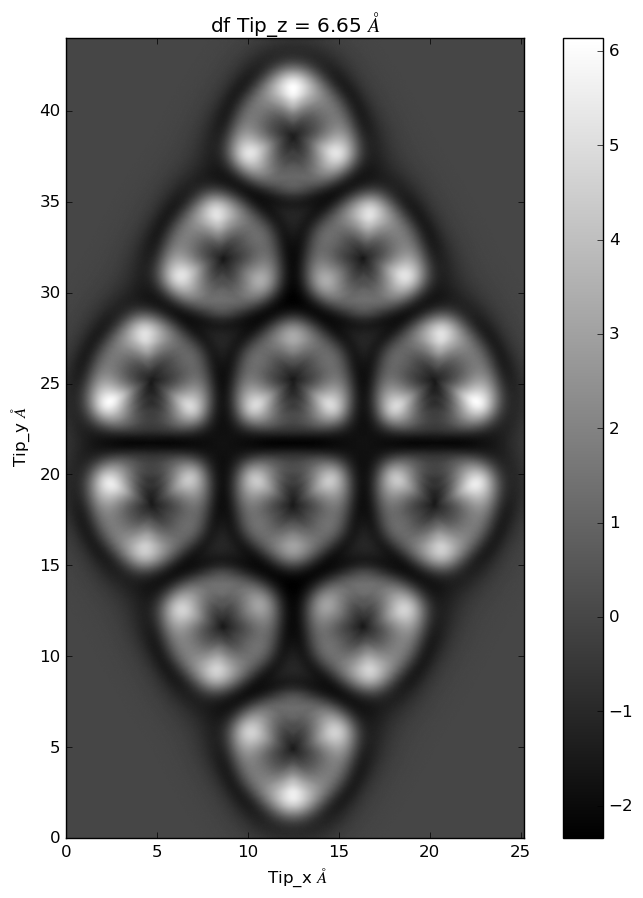

Supplement: File 1 — Datasets A0=1A adatoms_backbonds k=0.5. [file Beilstein_J_Nanotechnol-07-937-s001.zip › S1/A0=1A/adatoms_backbonds/k=0.5/results/df_0067.png]

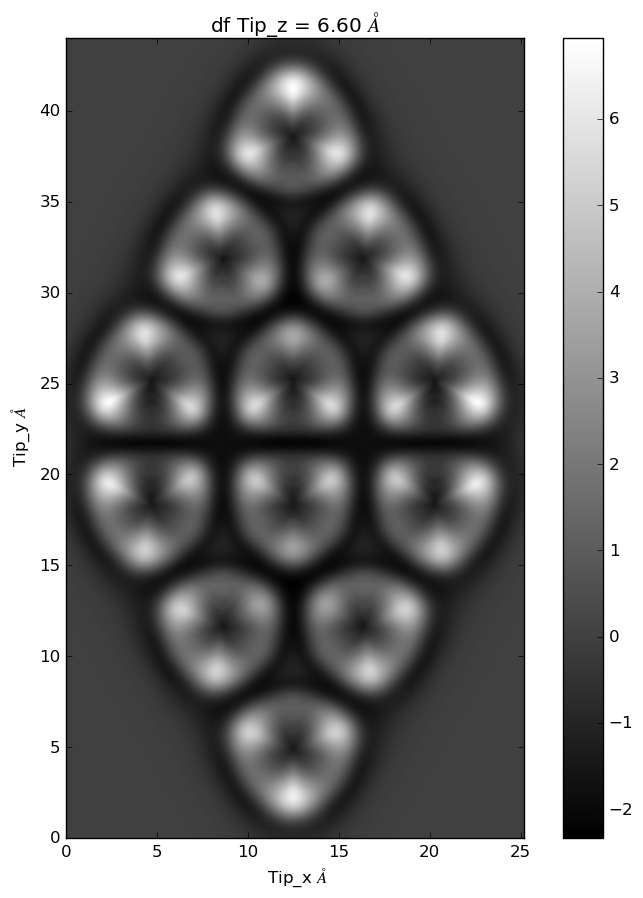

Supplement: File 1 — Datasets A0=1A adatoms_backbonds k=0.5. [file Beilstein_J_Nanotechnol-07-937-s001.zip › S1/A0=1A/adatoms_backbonds/k=0.5/results/df_0068.png]

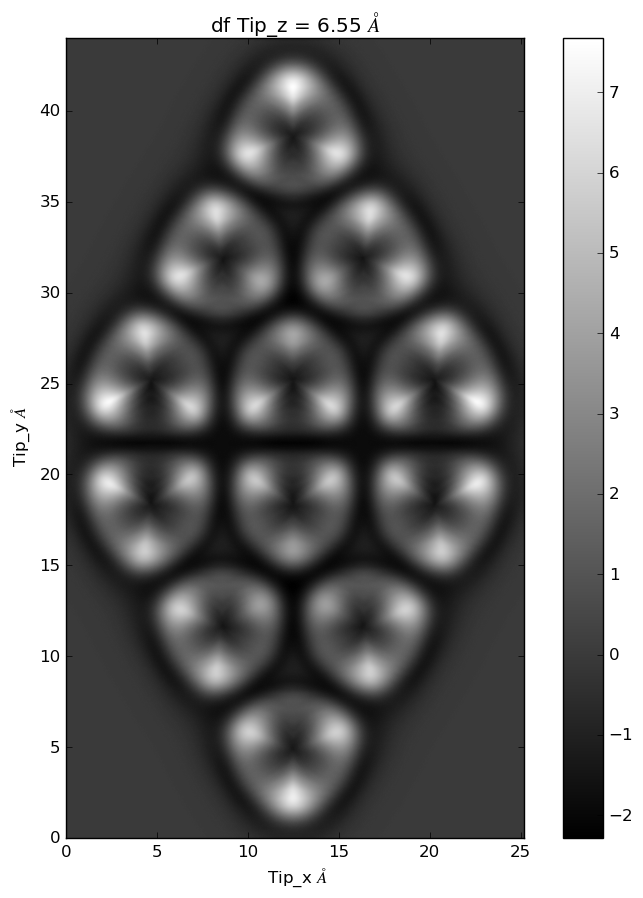

Supplement: File 1 — Datasets A0=1A adatoms_backbonds k=0.5. [file Beilstein_J_Nanotechnol-07-937-s001.zip › S1/A0=1A/adatoms_backbonds/k=0.5/results/df_0069.png]

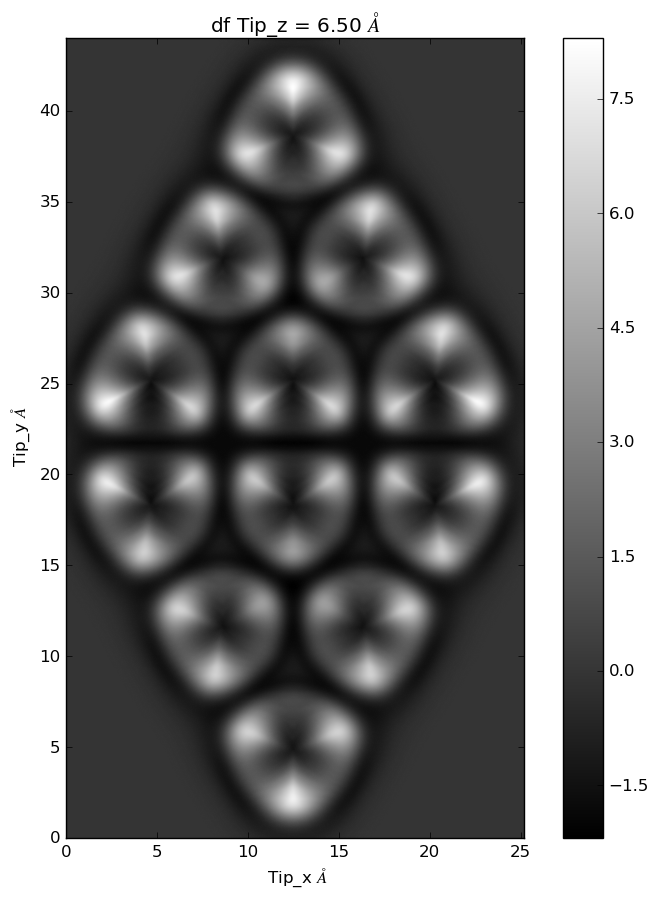

Supplement: File 1 — Datasets A0=1A adatoms_backbonds k=0.5. [file Beilstein_J_Nanotechnol-07-937-s001.zip › S1/A0=1A/adatoms_backbonds/k=0.5/results/df_0070.png]

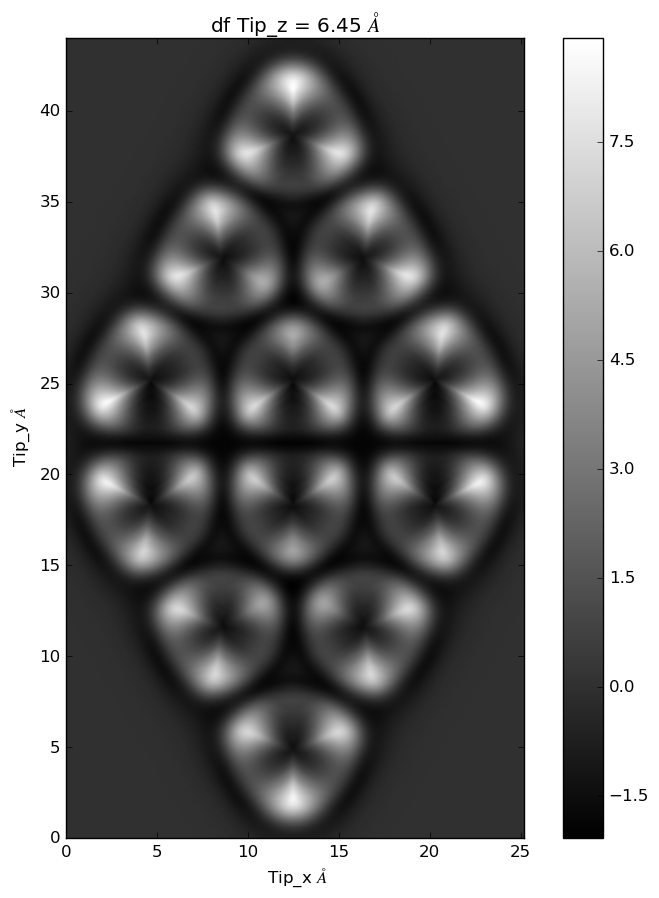

Supplement: File 1 — Datasets A0=1A adatoms_backbonds k=0.5. [file Beilstein_J_Nanotechnol-07-937-s001.zip › S1/A0=1A/adatoms_backbonds/k=0.5/results/df_0071.png]

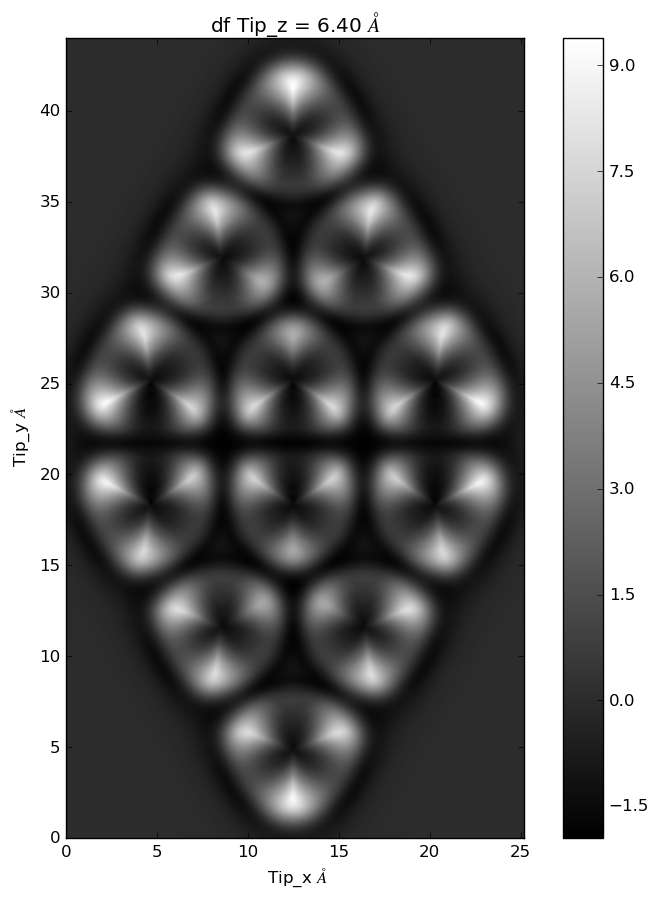

Supplement: File 1 — Datasets A0=1A adatoms_backbonds k=0.5. [file Beilstein_J_Nanotechnol-07-937-s001.zip › S1/A0=1A/adatoms_backbonds/k=0.5/results/df_0072.png]

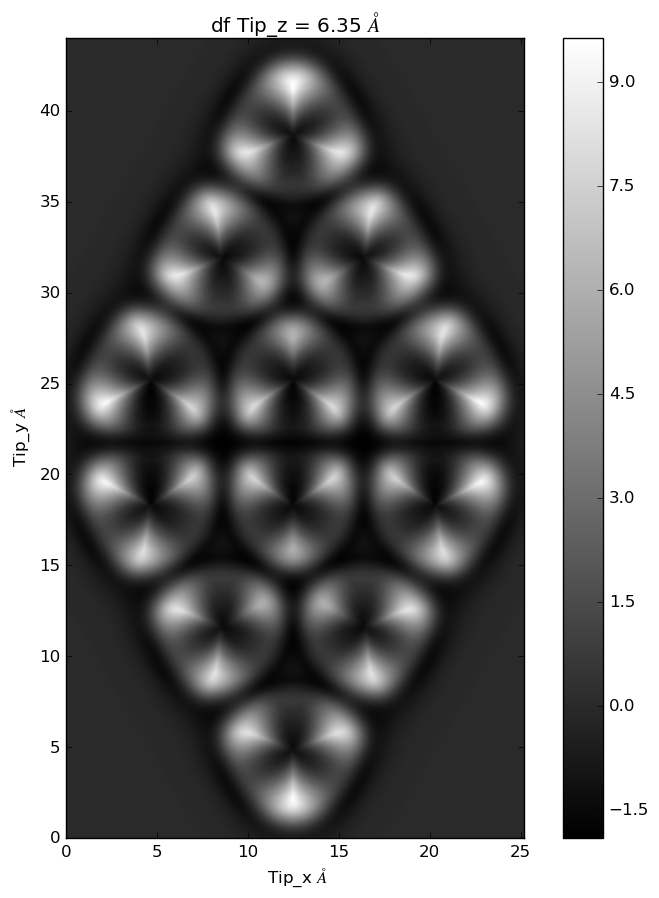

Supplement: File 1 — Datasets A0=1A adatoms_backbonds k=0.5. [file Beilstein_J_Nanotechnol-07-937-s001.zip › S1/A0=1A/adatoms_backbonds/k=0.5/results/df_0073.png]

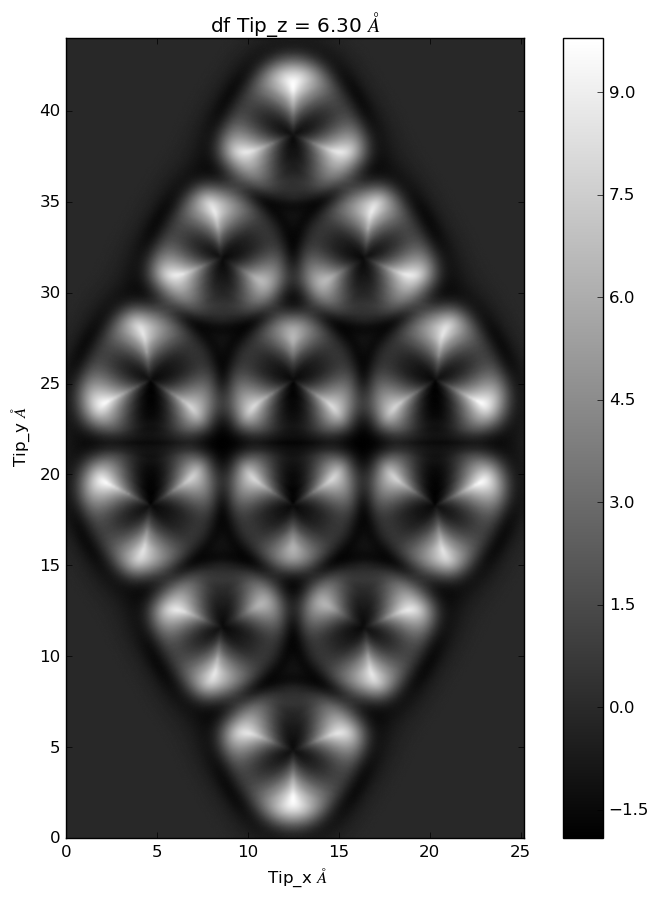

Supplement: File 1 — Datasets A0=1A adatoms_backbonds k=0.5. [file Beilstein_J_Nanotechnol-07-937-s001.zip › S1/A0=1A/adatoms_backbonds/k=0.5/results/df_0074.png]

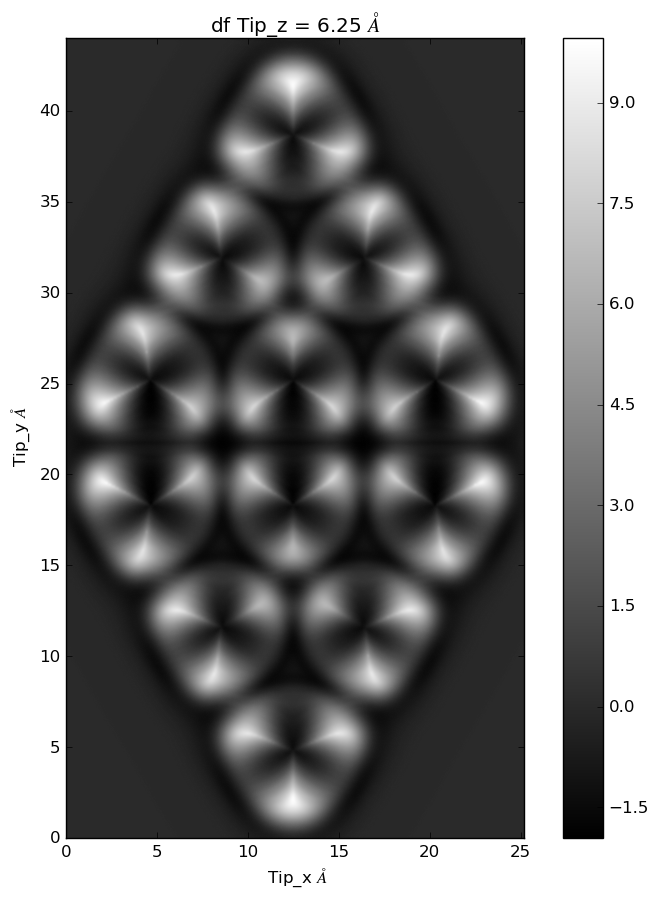

Supplement: File 1 — Datasets A0=1A adatoms_backbonds k=0.5. [file Beilstein_J_Nanotechnol-07-937-s001.zip › S1/A0=1A/adatoms_backbonds/k=0.5/results/df_0075.png]

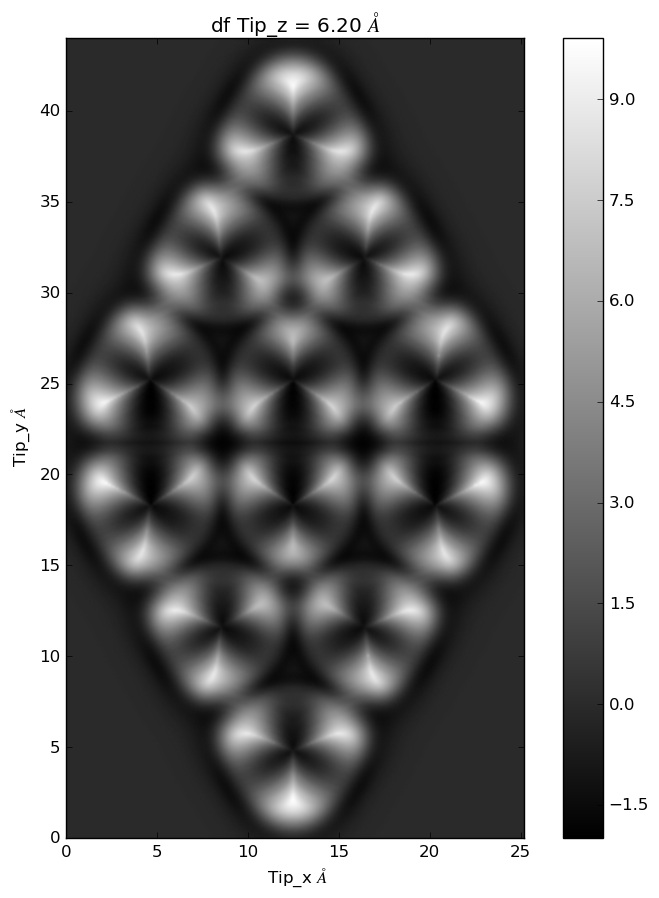

Supplement: File 1 — Datasets A0=1A adatoms_backbonds k=0.5. [file Beilstein_J_Nanotechnol-07-937-s001.zip › S1/A0=1A/adatoms_backbonds/k=0.5/results/df_0076.png]

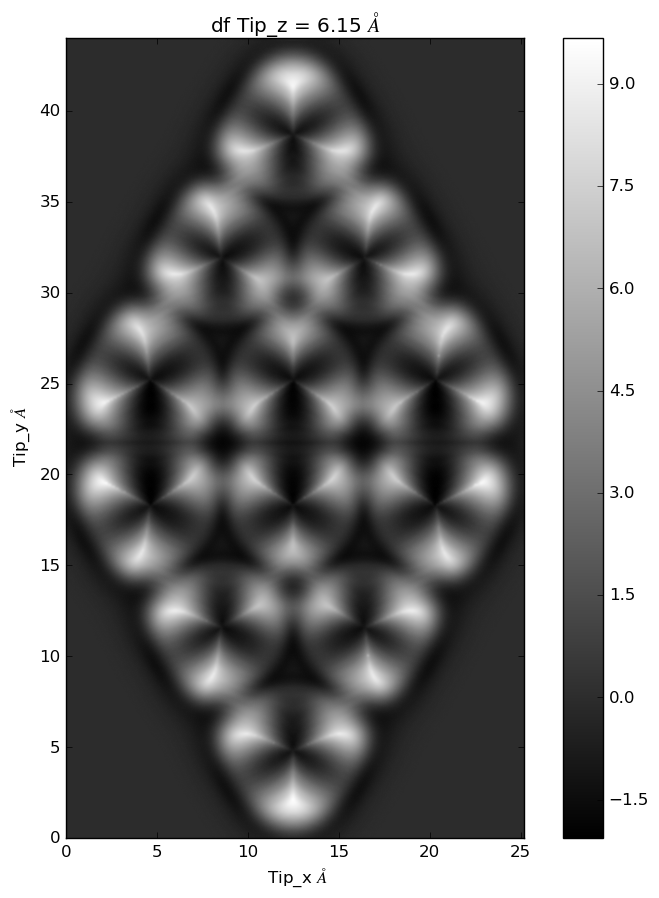

Supplement: File 1 — Datasets A0=1A adatoms_backbonds k=0.5. [file Beilstein_J_Nanotechnol-07-937-s001.zip › S1/A0=1A/adatoms_backbonds/k=0.5/results/df_0077.png]

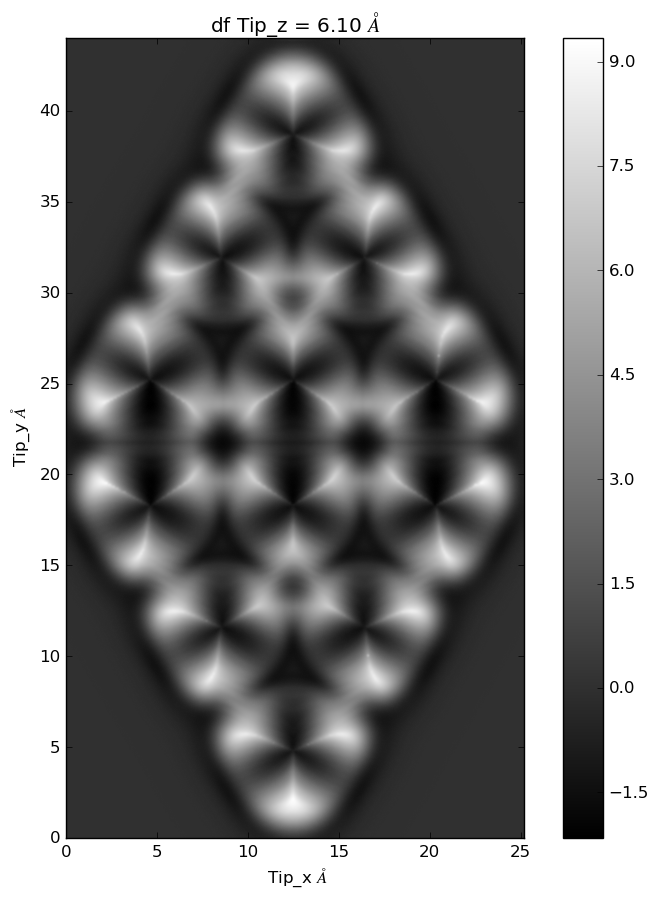

Supplement: File 1 — Datasets A0=1A adatoms_backbonds k=0.5. [file Beilstein_J_Nanotechnol-07-937-s001.zip › S1/A0=1A/adatoms_backbonds/k=0.5/results/df_0078.png]

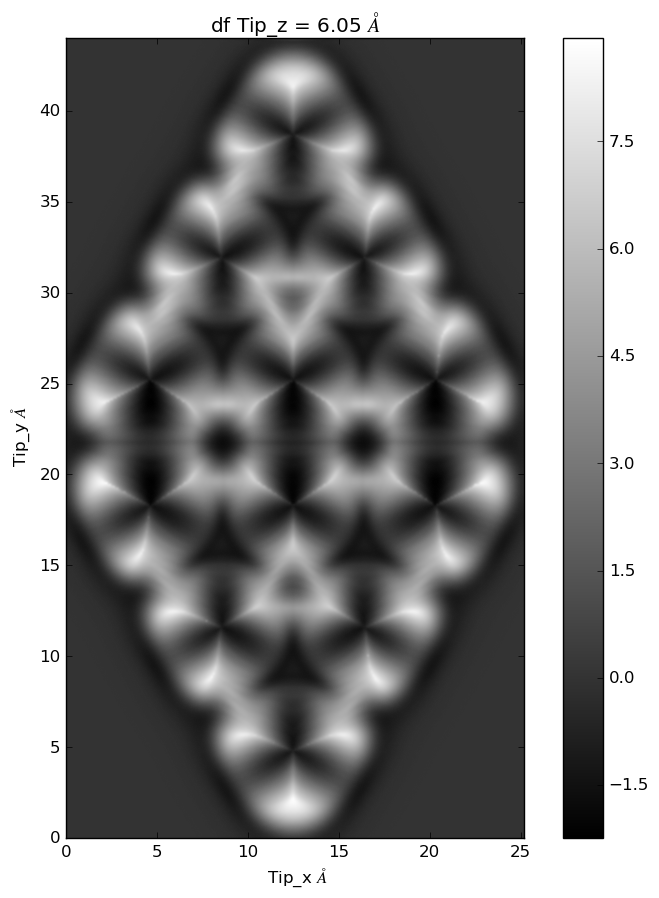

Supplement: File 1 — Datasets A0=1A adatoms_backbonds k=0.5. [file Beilstein_J_Nanotechnol-07-937-s001.zip › S1/A0=1A/adatoms_backbonds/k=0.5/results/df_0079.png]

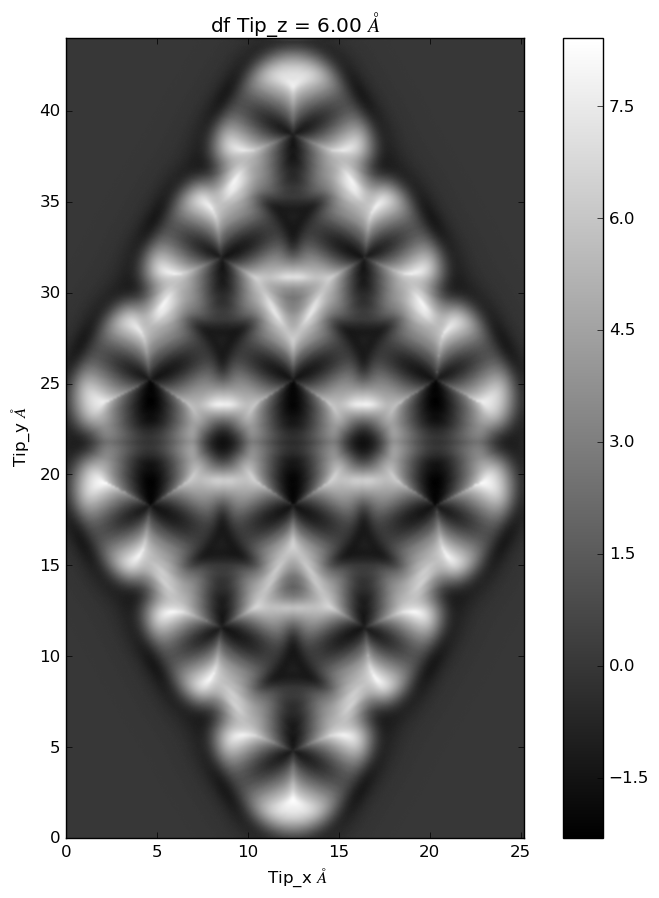

Supplement: File 1 — Datasets A0=1A adatoms_backbonds k=0.5. [file Beilstein_J_Nanotechnol-07-937-s001.zip › S1/A0=1A/adatoms_backbonds/k=0.5/results/df_0080.png]

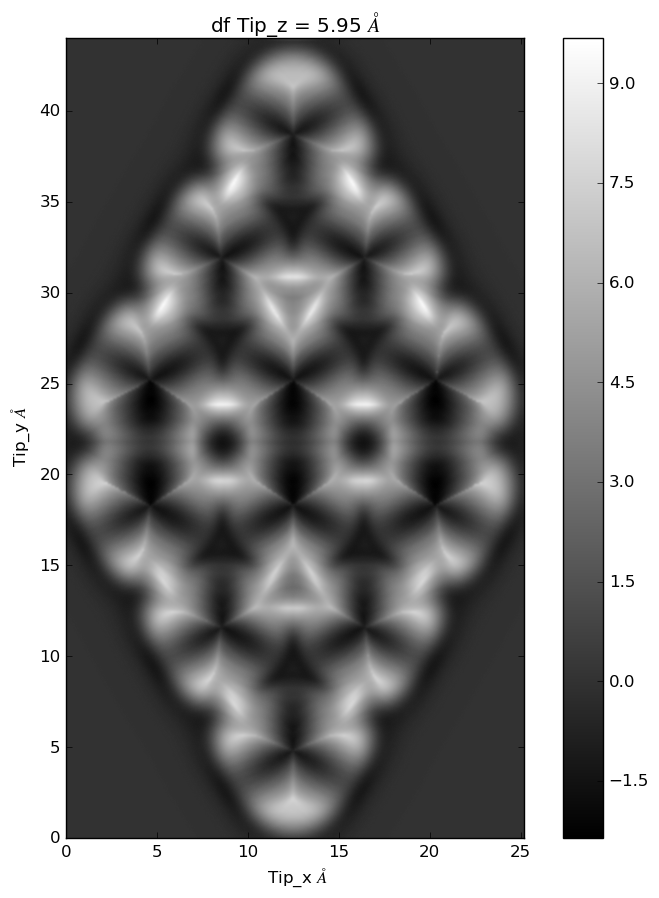

Supplement: File 1 — Datasets A0=1A adatoms_backbonds k=0.5. [file Beilstein_J_Nanotechnol-07-937-s001.zip › S1/A0=1A/adatoms_backbonds/k=0.5/results/df_0081.png]

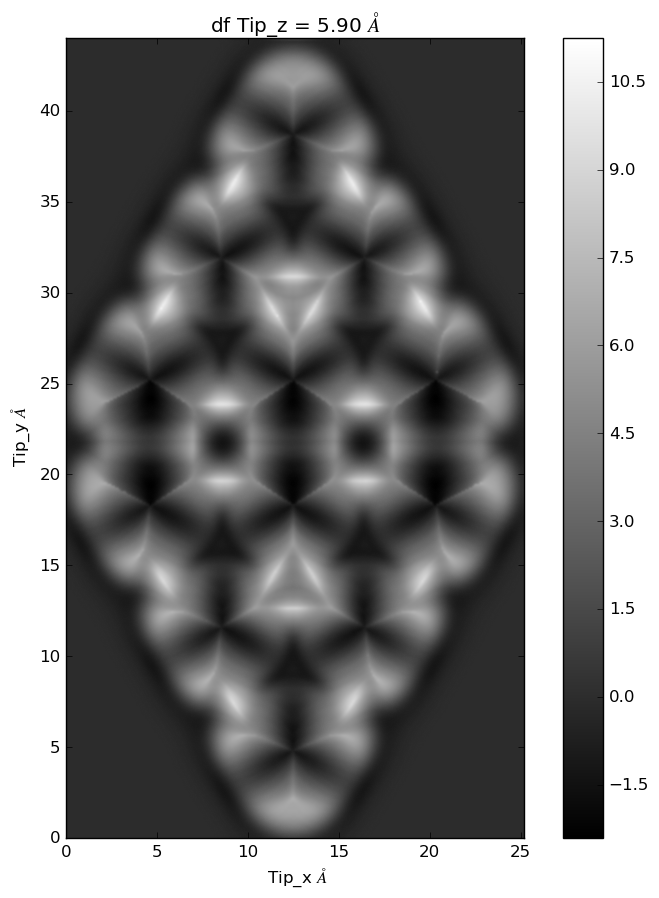

Supplement: File 1 — Datasets A0=1A adatoms_backbonds k=0.5. [file Beilstein_J_Nanotechnol-07-937-s001.zip › S1/A0=1A/adatoms_backbonds/k=0.5/results/df_0082.png]

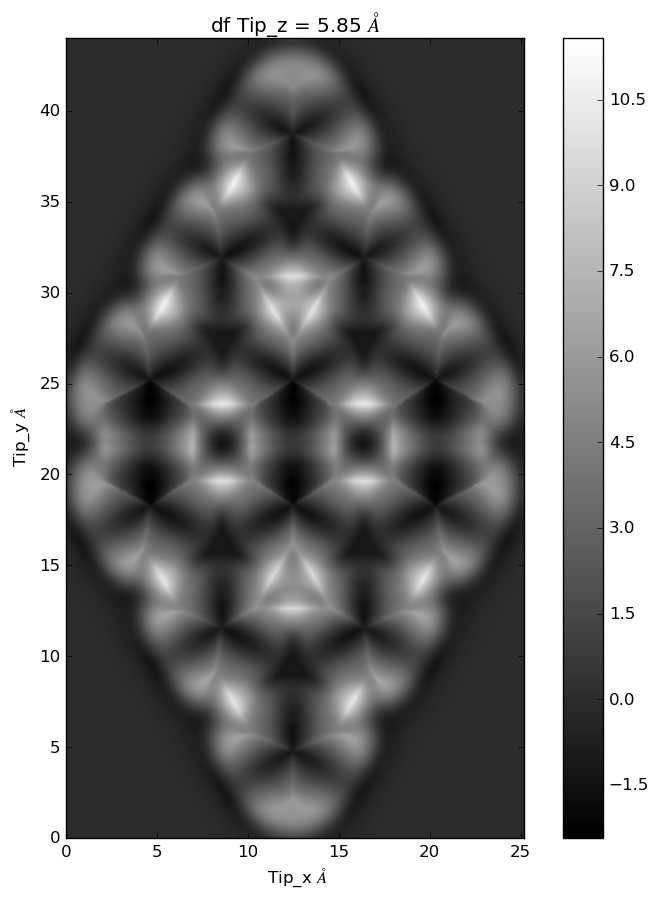

Supplement: File 1 — Datasets A0=1A adatoms_backbonds k=0.5. [file Beilstein_J_Nanotechnol-07-937-s001.zip › S1/A0=1A/adatoms_backbonds/k=0.5/results/df_0083.png]

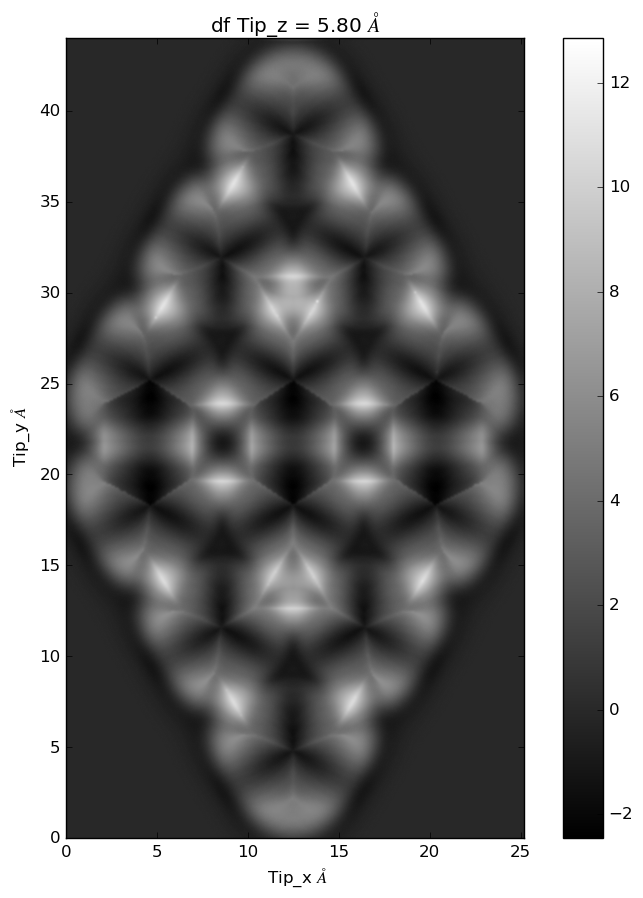

Supplement: File 1 — Datasets A0=1A adatoms_backbonds k=0.5. [file Beilstein_J_Nanotechnol-07-937-s001.zip › S1/A0=1A/adatoms_backbonds/k=0.5/results/df_0084.png]

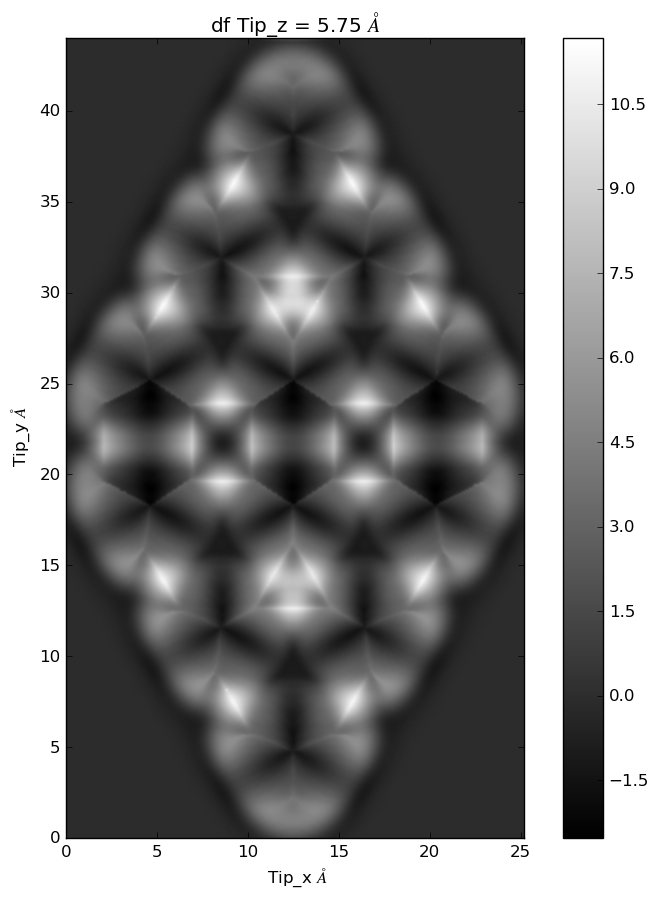

Supplement: File 1 — Datasets A0=1A adatoms_backbonds k=0.5. [file Beilstein_J_Nanotechnol-07-937-s001.zip › S1/A0=1A/adatoms_backbonds/k=0.5/results/df_0085.png]

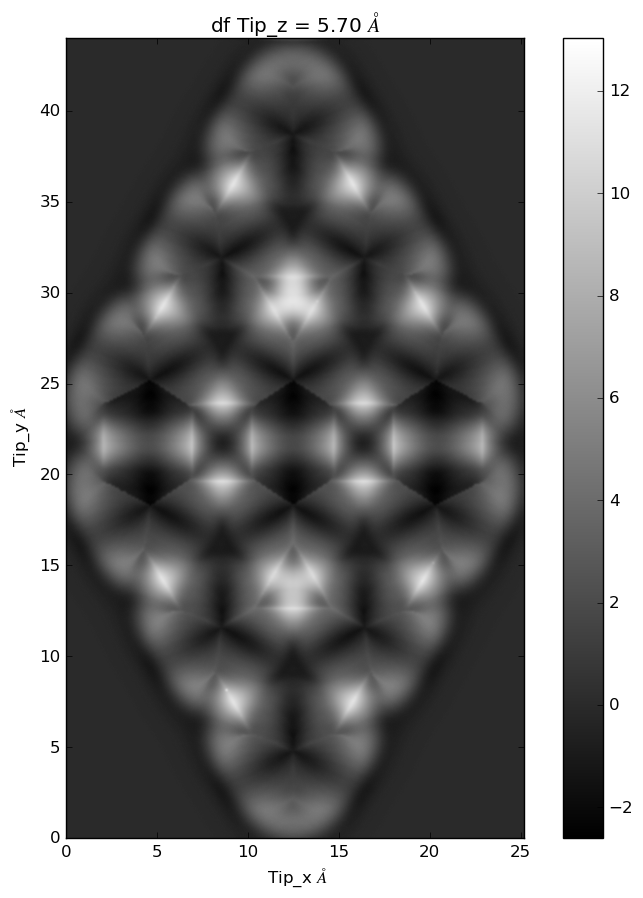

Supplement: File 1 — Datasets A0=1A adatoms_backbonds k=0.5. [file Beilstein_J_Nanotechnol-07-937-s001.zip › S1/A0=1A/adatoms_backbonds/k=0.5/results/df_0086.png]

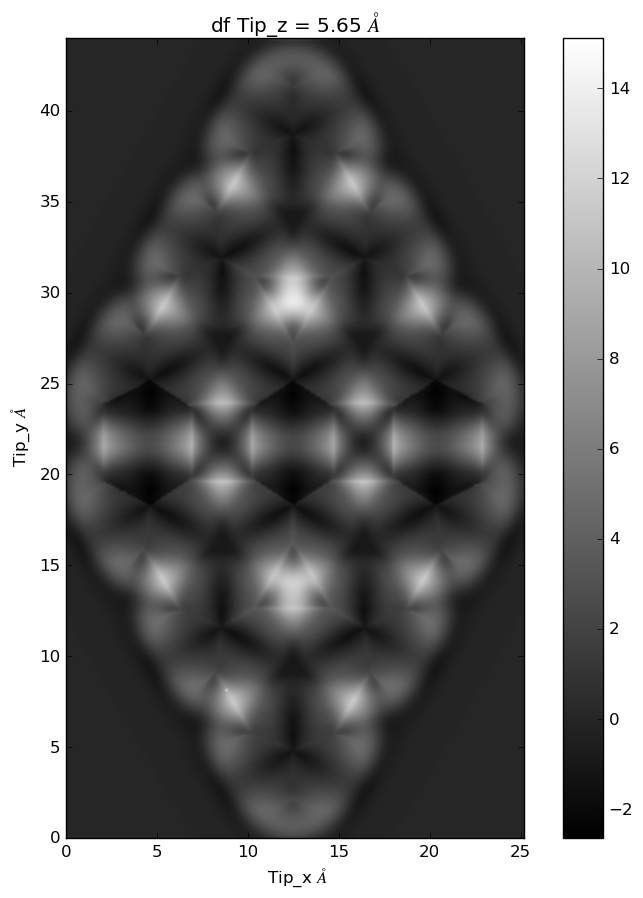

Supplement: File 1 — Datasets A0=1A adatoms_backbonds k=0.5. [file Beilstein_J_Nanotechnol-07-937-s001.zip › S1/A0=1A/adatoms_backbonds/k=0.5/results/df_0087.png]

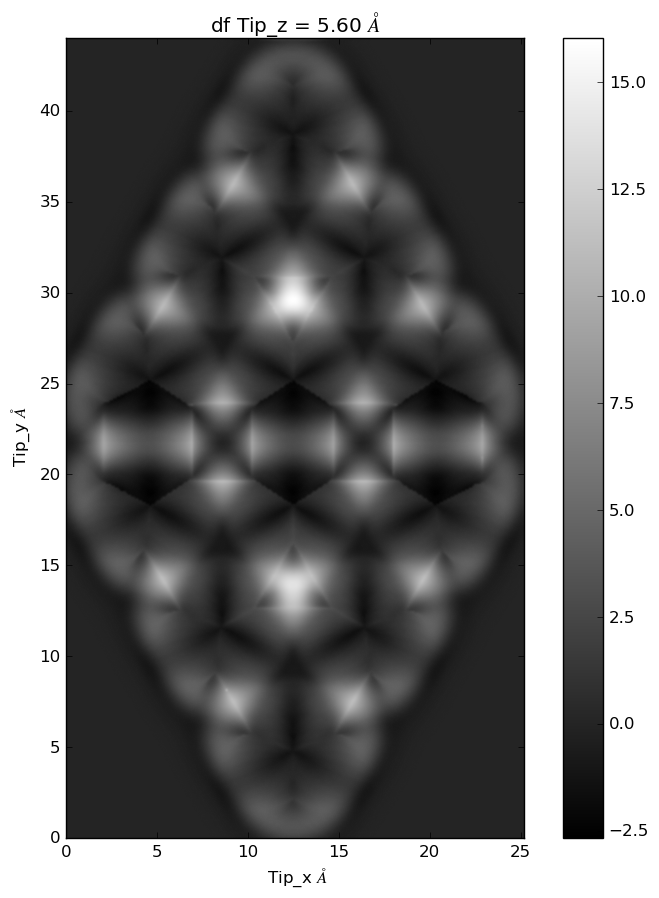

Supplement: File 1 — Datasets A0=1A adatoms_backbonds k=0.5. [file Beilstein_J_Nanotechnol-07-937-s001.zip › S1/A0=1A/adatoms_backbonds/k=0.5/results/df_0088.png]

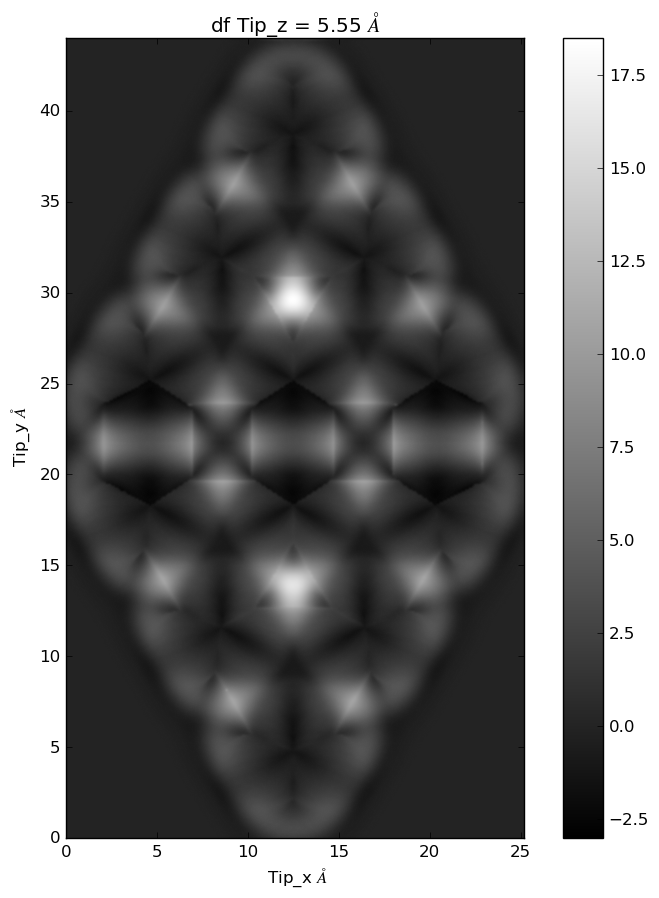

Supplement: File 1 — Datasets A0=1A adatoms_backbonds k=0.5. [file Beilstein_J_Nanotechnol-07-937-s001.zip › S1/A0=1A/adatoms_backbonds/k=0.5/results/df_0089.png]

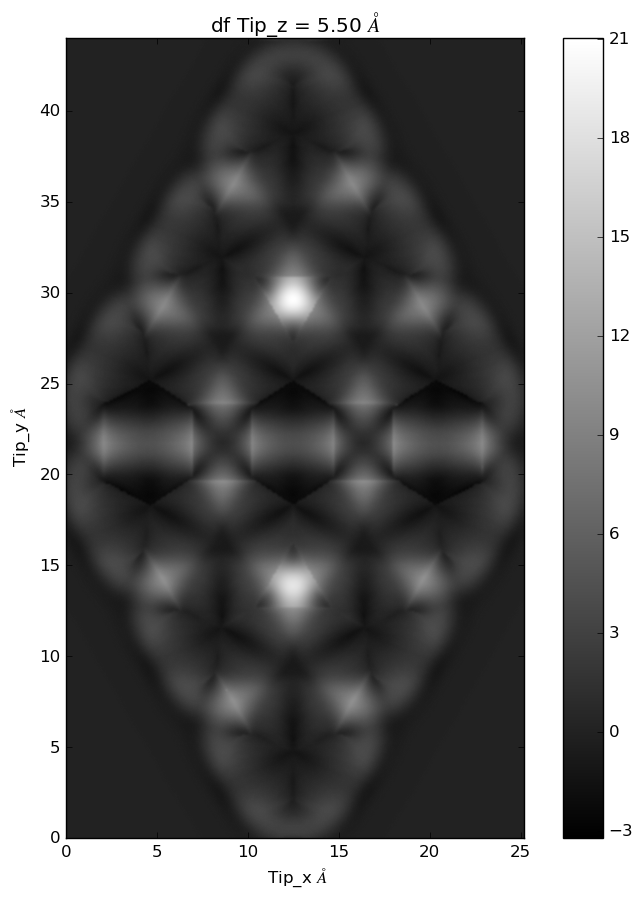

Supplement: File 1 — Datasets A0=1A adatoms_backbonds k=0.5. [file Beilstein_J_Nanotechnol-07-937-s001.zip › S1/A0=1A/adatoms_backbonds/k=0.5/results/df_0090.png]

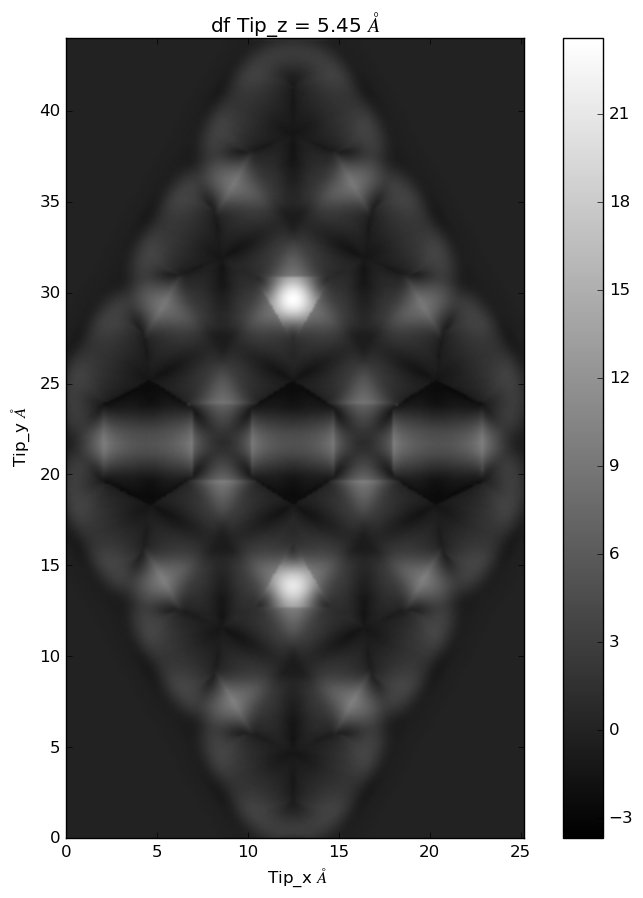

Supplement: File 1 — Datasets A0=1A adatoms_backbonds k=0.5. [file Beilstein_J_Nanotechnol-07-937-s001.zip › S1/A0=1A/adatoms_backbonds/k=0.5/results/df_0091.png]

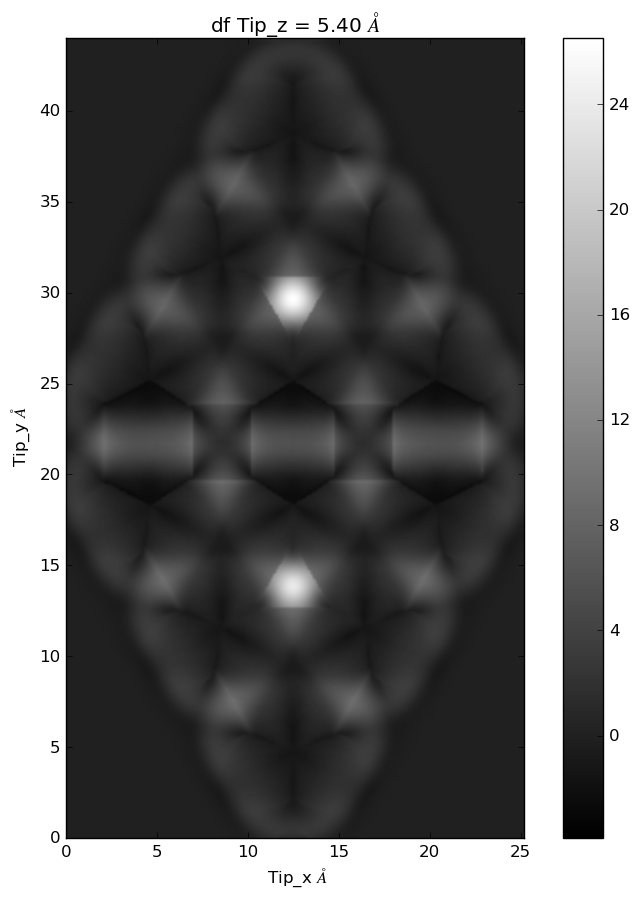

Supplement: File 1 — Datasets A0=1A adatoms_backbonds k=0.5. [file Beilstein_J_Nanotechnol-07-937-s001.zip › S1/A0=1A/adatoms_backbonds/k=0.5/results/df_0092.png]

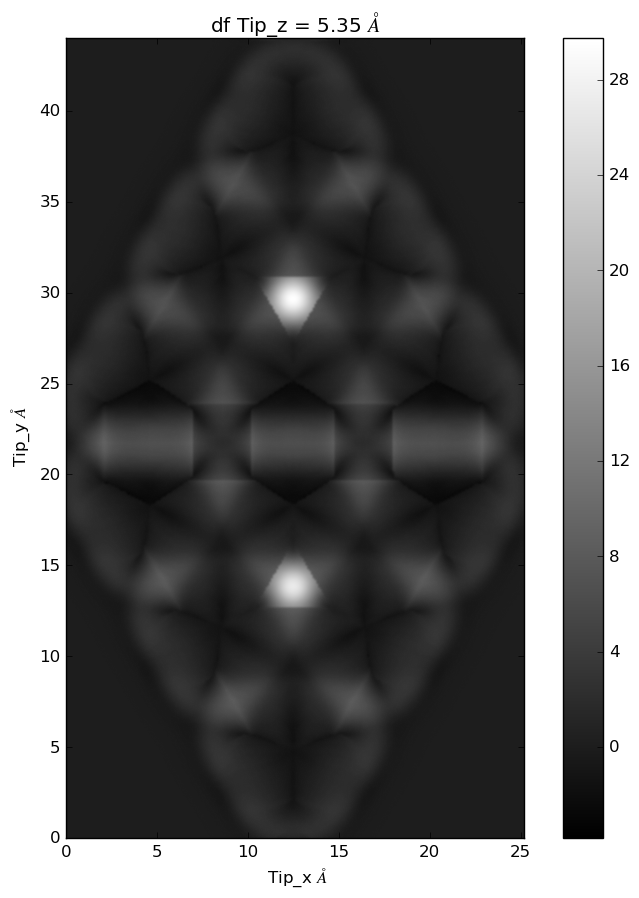

Supplement: File 1 — Datasets A0=1A adatoms_backbonds k=0.5. [file Beilstein_J_Nanotechnol-07-937-s001.zip › S1/A0=1A/adatoms_backbonds/k=0.5/results/df_0093.png]

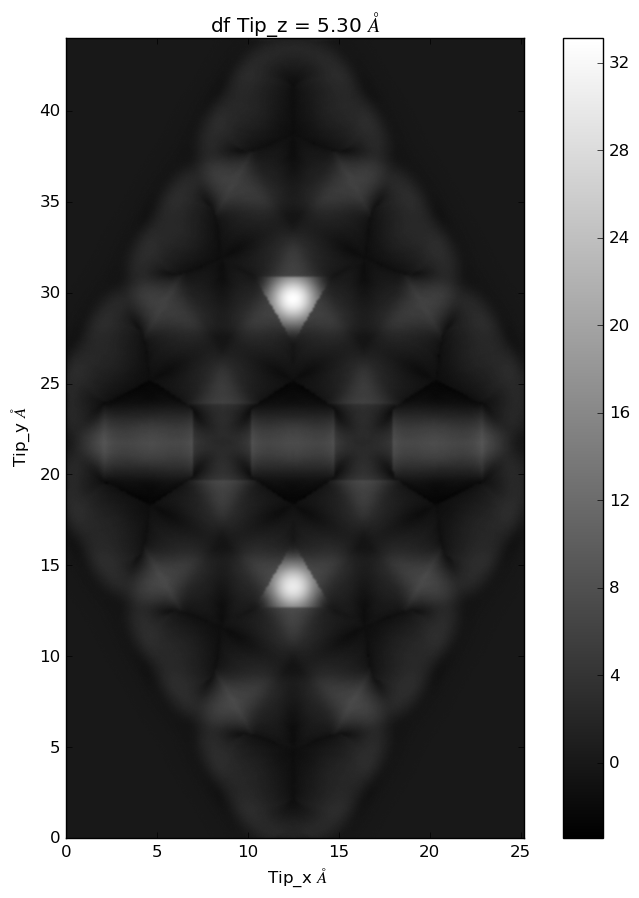

Supplement: File 1 — Datasets A0=1A adatoms_backbonds k=0.5. [file Beilstein_J_Nanotechnol-07-937-s001.zip › S1/A0=1A/adatoms_backbonds/k=0.5/results/df_0094.png]

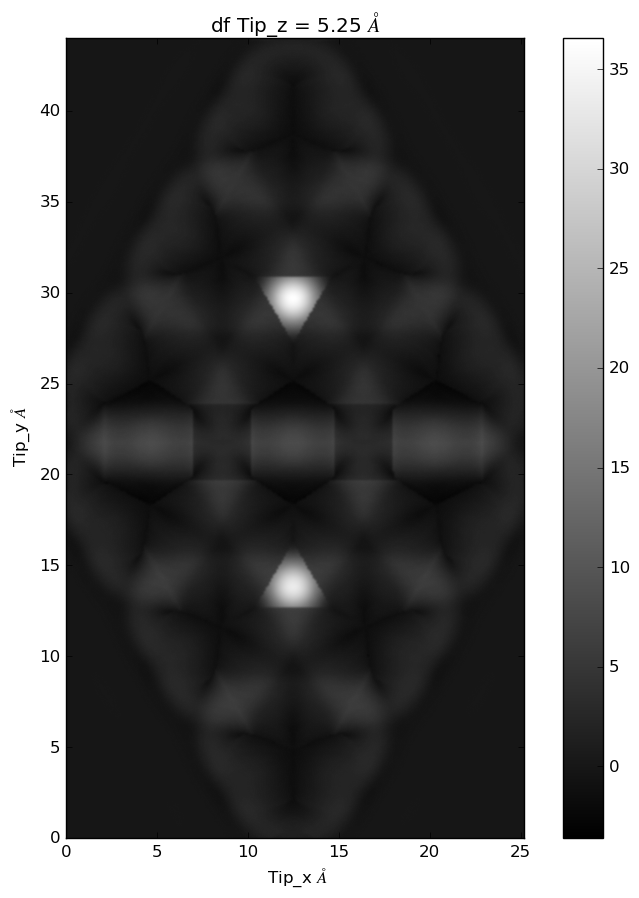

Supplement: File 1 — Datasets A0=1A adatoms_backbonds k=0.5. [file Beilstein_J_Nanotechnol-07-937-s001.zip › S1/A0=1A/adatoms_backbonds/k=0.5/results/df_0095.png]

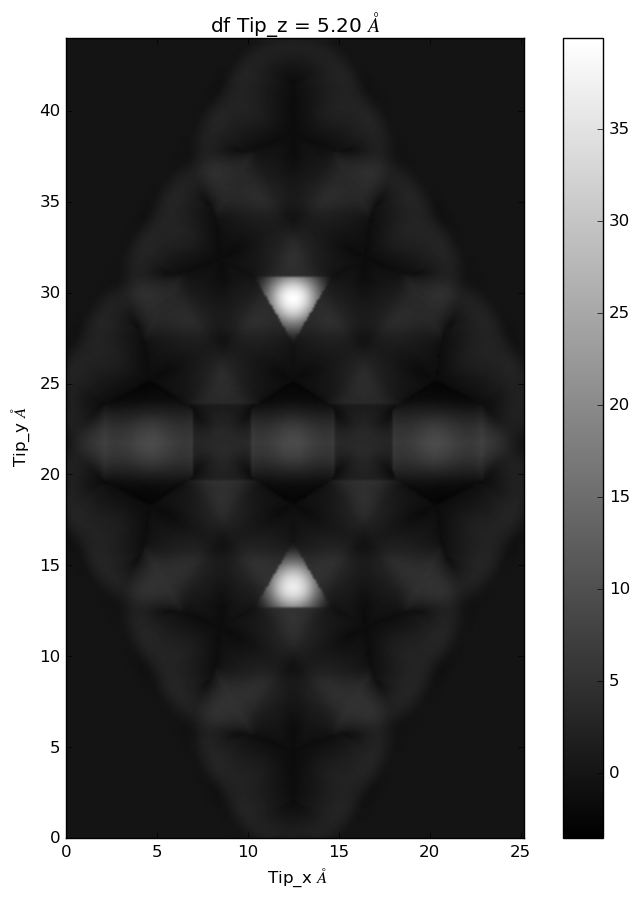

Supplement: File 1 — Datasets A0=1A adatoms_backbonds k=0.5. [file Beilstein_J_Nanotechnol-07-937-s001.zip › S1/A0=1A/adatoms_backbonds/k=0.5/results/df_0096.png]

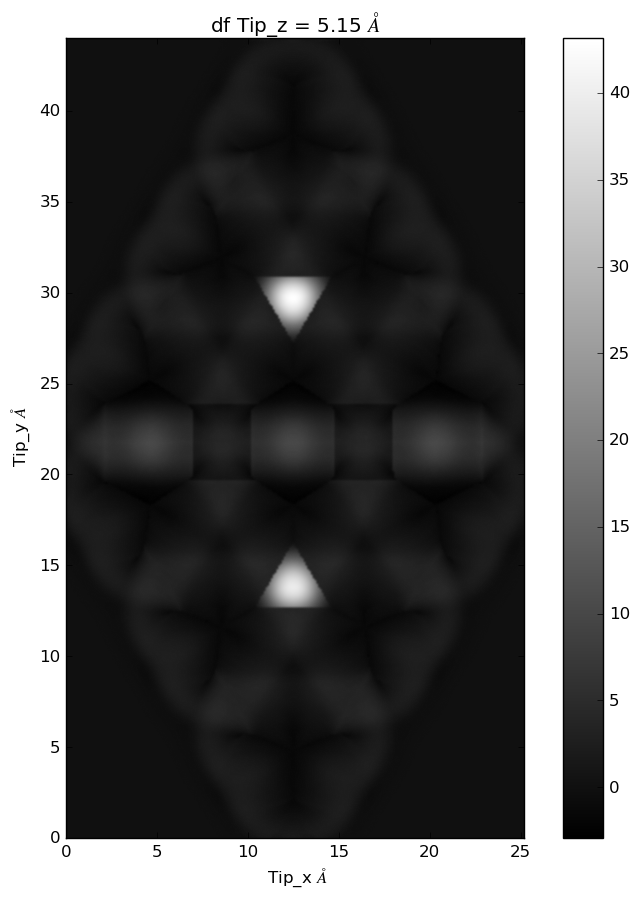

Supplement: File 1 — Datasets A0=1A adatoms_backbonds k=0.5. [file Beilstein_J_Nanotechnol-07-937-s001.zip › S1/A0=1A/adatoms_backbonds/k=0.5/results/df_0097.png]

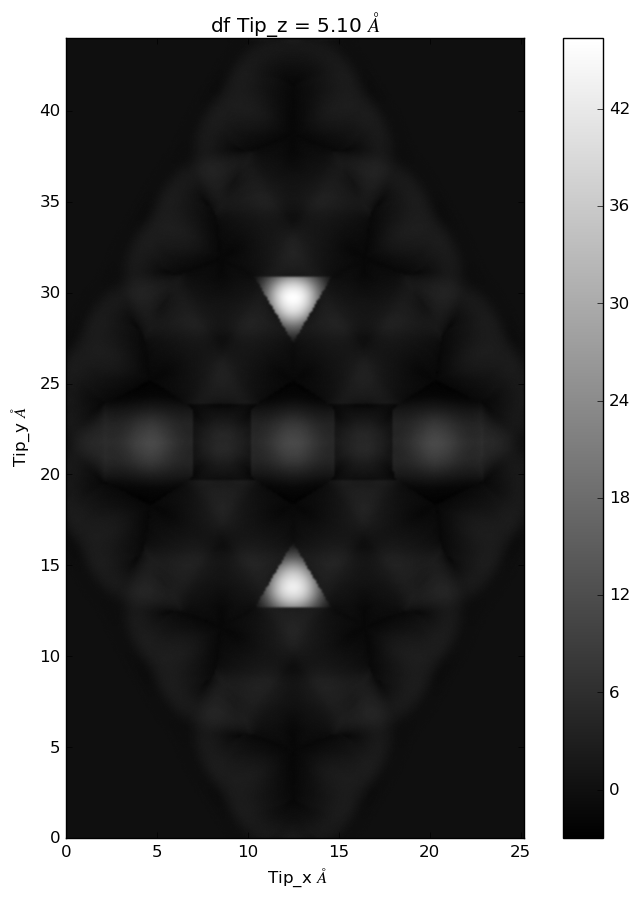

Supplement: File 1 — Datasets A0=1A adatoms_backbonds k=0.5. [file Beilstein_J_Nanotechnol-07-937-s001.zip › S1/A0=1A/adatoms_backbonds/k=0.5/results/df_0098.png]

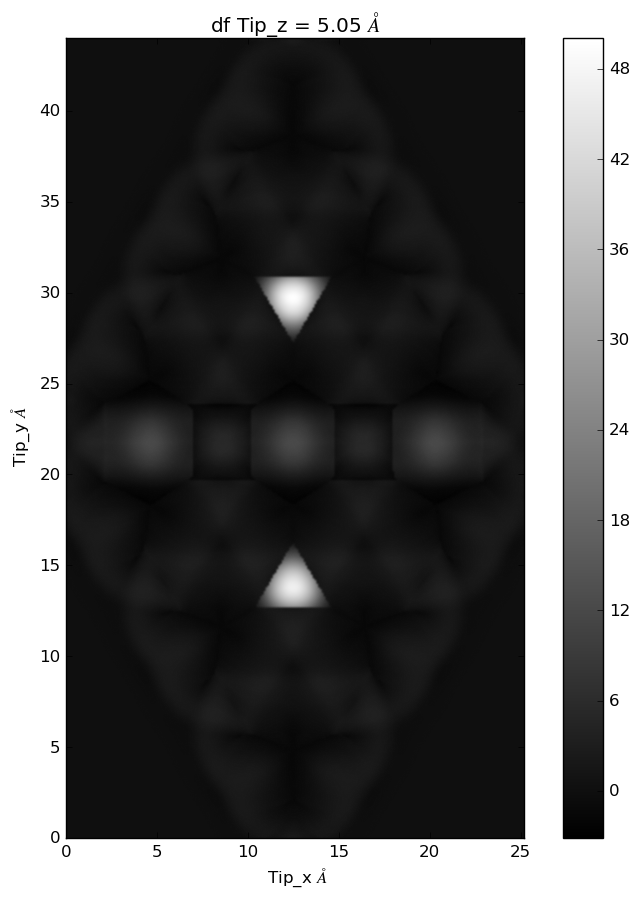

Supplement: File 1 — Datasets A0=1A adatoms_backbonds k=0.5. [file Beilstein_J_Nanotechnol-07-937-s001.zip › S1/A0=1A/adatoms_backbonds/k=0.5/results/df_0099.png]
