# Supplementary figures and images for: Modelling of ‘sub-atomic’ contrast resulting from back-bonding on Si(111)-7×7 (part 6 of 18)
Source: Beilstein J Nanotechnol. 2016 Jun 29;7:937–45. doi: 10.3762/bjnano.7.85 (PMC4979881; doi:10.3762/bjnano.7.85)

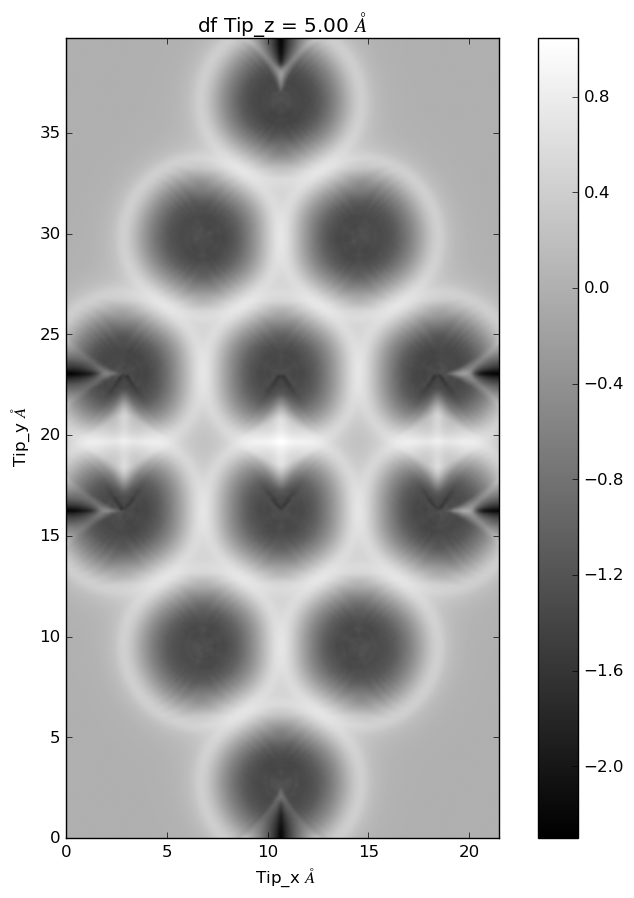

Supplement: File 3 — Datasets A0=1A adatoms_only k=0.5. [file Beilstein_J_Nanotechnol-07-937-s003.zip › S3/A0=1A/adatoms_only/k=0.5/results/df_0100.png]

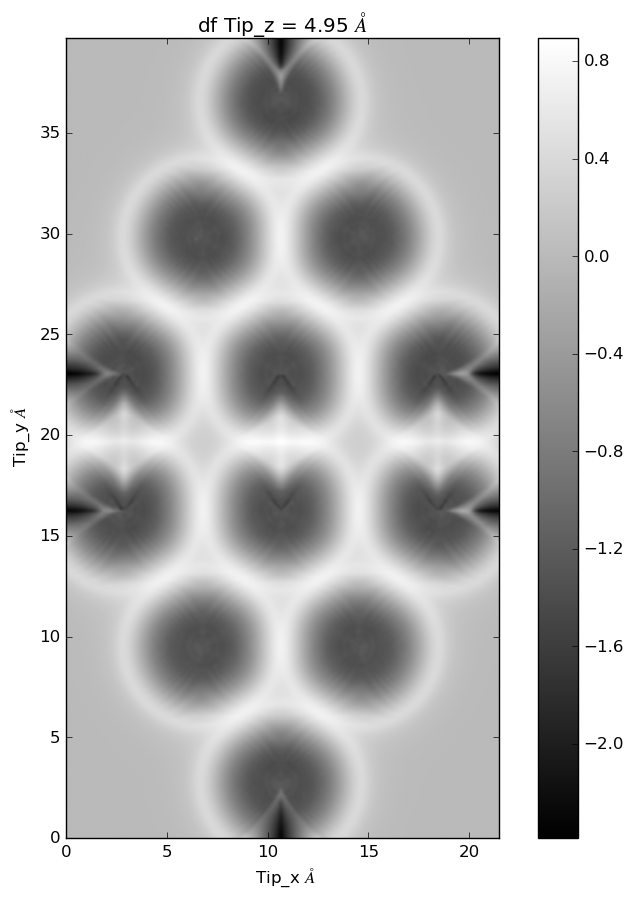

Supplement: File 3 — Datasets A0=1A adatoms_only k=0.5. [file Beilstein_J_Nanotechnol-07-937-s003.zip › S3/A0=1A/adatoms_only/k=0.5/results/df_0101.png]

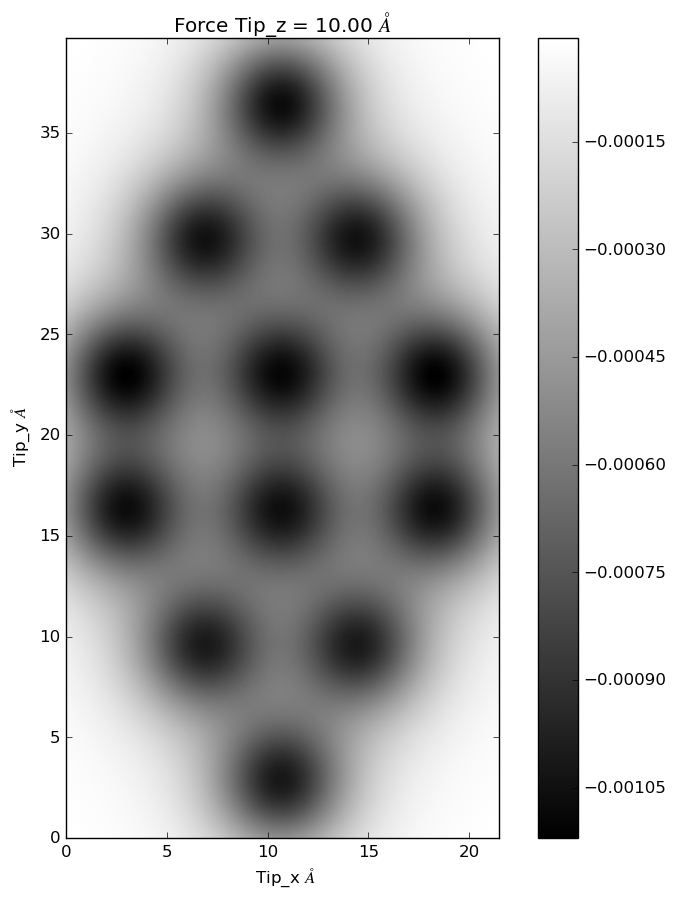

Supplement: File 3 — Datasets A0=1A adatoms_only k=0.5. [file Beilstein_J_Nanotechnol-07-937-s003.zip › S3/A0=1A/adatoms_only/k=0.5/results/Force_0000.png]

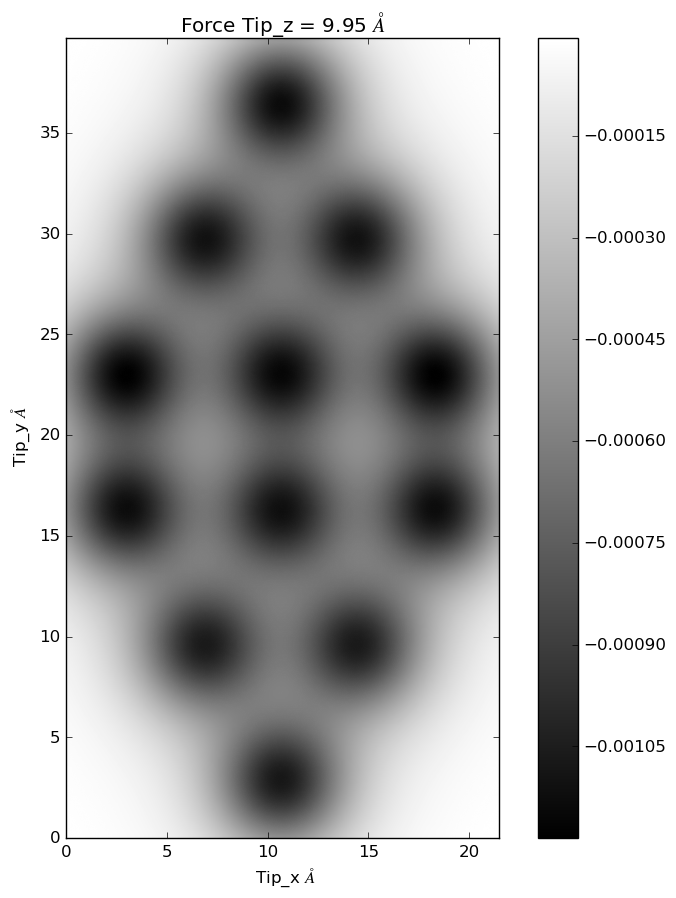

Supplement: File 3 — Datasets A0=1A adatoms_only k=0.5. [file Beilstein_J_Nanotechnol-07-937-s003.zip › S3/A0=1A/adatoms_only/k=0.5/results/Force_0001.png]

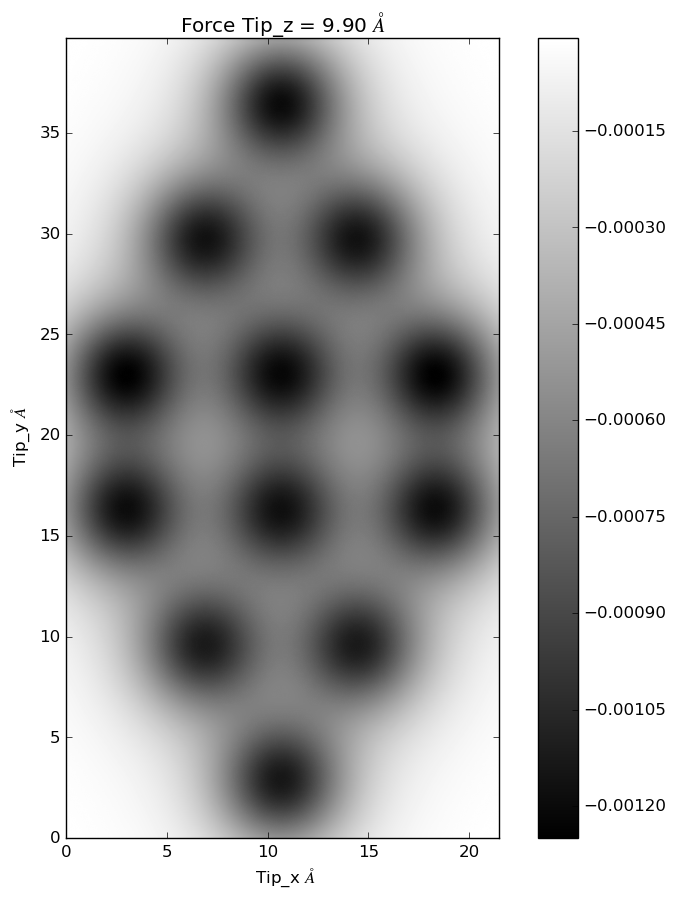

Supplement: File 3 — Datasets A0=1A adatoms_only k=0.5. [file Beilstein_J_Nanotechnol-07-937-s003.zip › S3/A0=1A/adatoms_only/k=0.5/results/Force_0002.png]

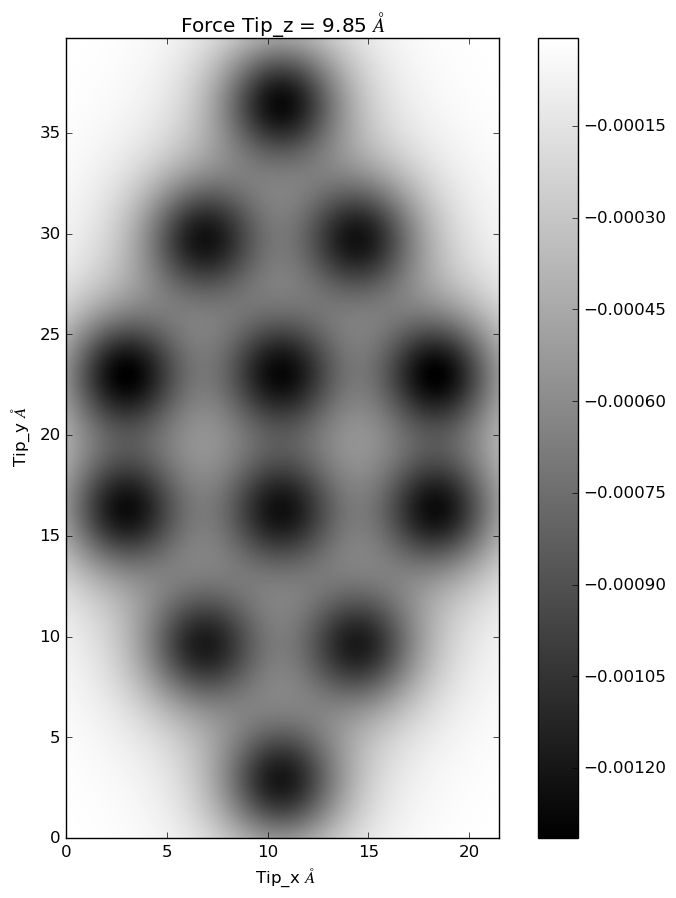

Supplement: File 3 — Datasets A0=1A adatoms_only k=0.5. [file Beilstein_J_Nanotechnol-07-937-s003.zip › S3/A0=1A/adatoms_only/k=0.5/results/Force_0003.png]

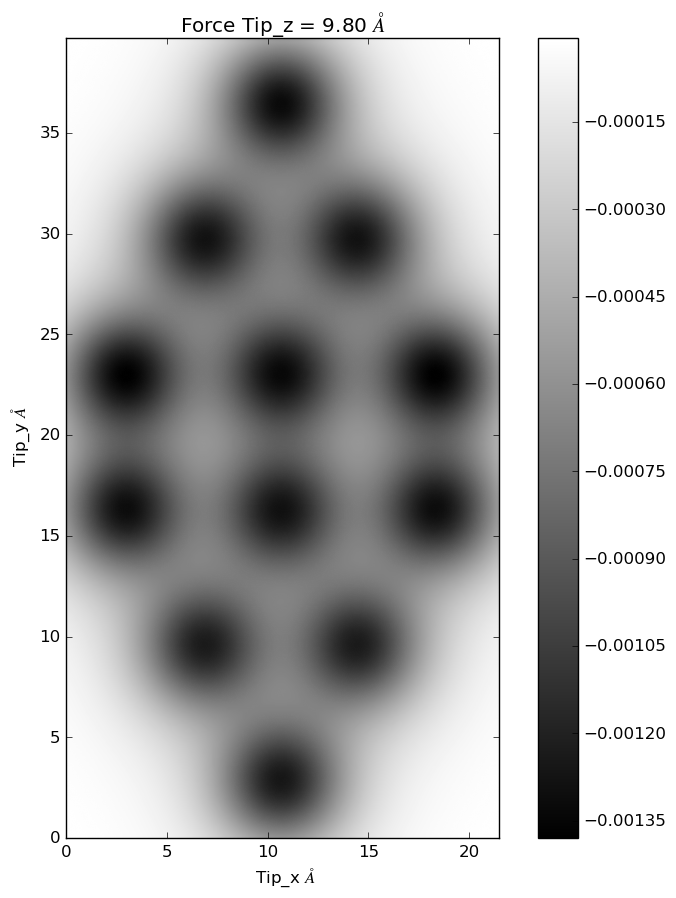

Supplement: File 3 — Datasets A0=1A adatoms_only k=0.5. [file Beilstein_J_Nanotechnol-07-937-s003.zip › S3/A0=1A/adatoms_only/k=0.5/results/Force_0004.png]

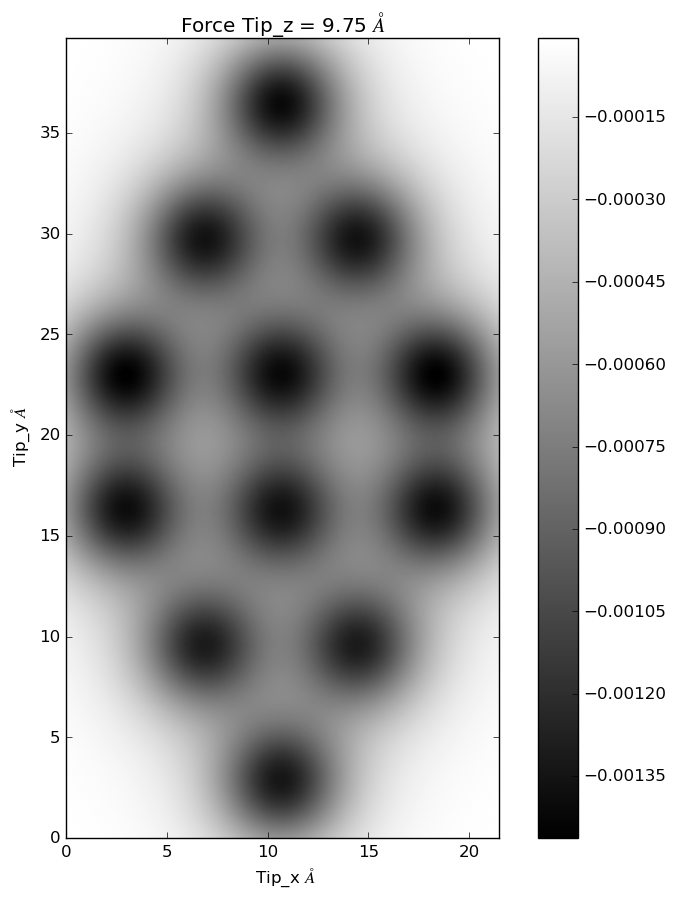

Supplement: File 3 — Datasets A0=1A adatoms_only k=0.5. [file Beilstein_J_Nanotechnol-07-937-s003.zip › S3/A0=1A/adatoms_only/k=0.5/results/Force_0005.png]

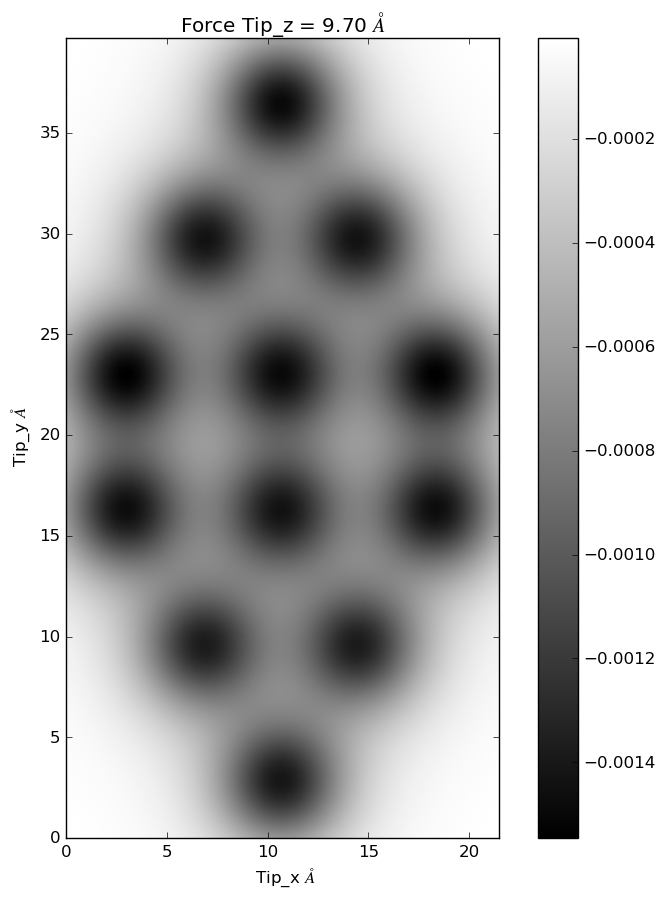

Supplement: File 3 — Datasets A0=1A adatoms_only k=0.5. [file Beilstein_J_Nanotechnol-07-937-s003.zip › S3/A0=1A/adatoms_only/k=0.5/results/Force_0006.png]

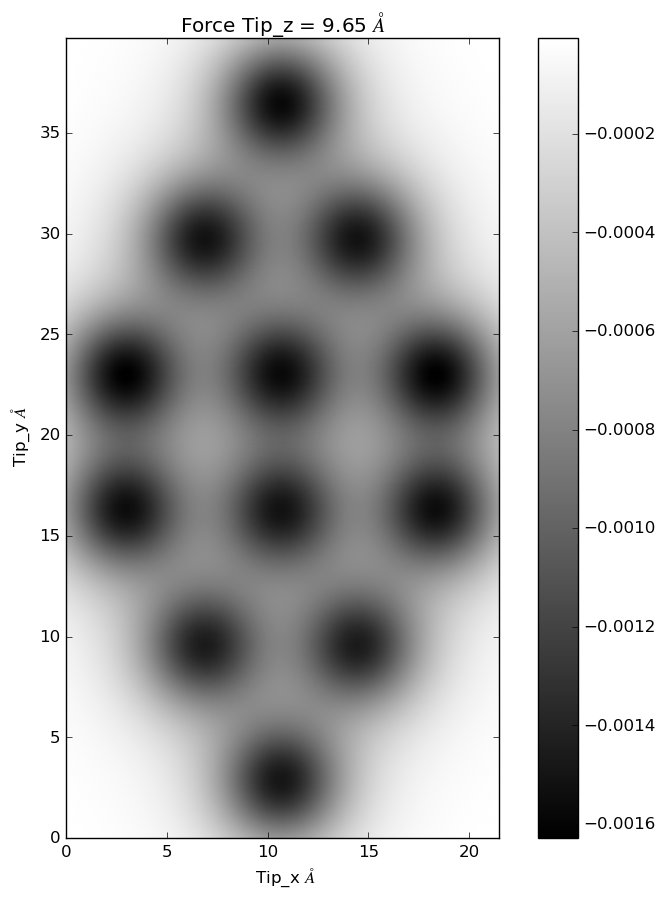

Supplement: File 3 — Datasets A0=1A adatoms_only k=0.5. [file Beilstein_J_Nanotechnol-07-937-s003.zip › S3/A0=1A/adatoms_only/k=0.5/results/Force_0007.png]

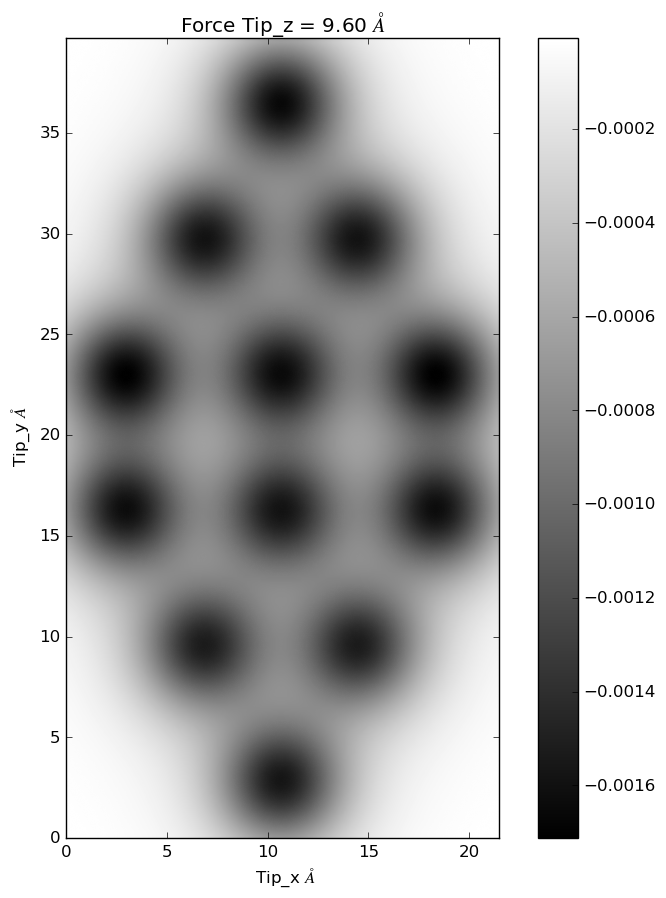

Supplement: File 3 — Datasets A0=1A adatoms_only k=0.5. [file Beilstein_J_Nanotechnol-07-937-s003.zip › S3/A0=1A/adatoms_only/k=0.5/results/Force_0008.png]

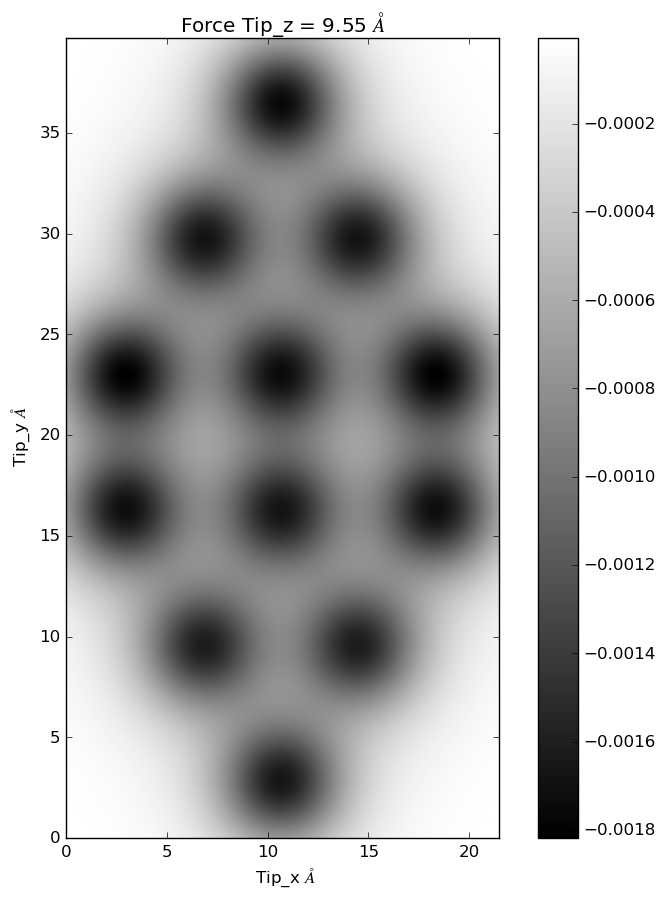

Supplement: File 3 — Datasets A0=1A adatoms_only k=0.5. [file Beilstein_J_Nanotechnol-07-937-s003.zip › S3/A0=1A/adatoms_only/k=0.5/results/Force_0009.png]

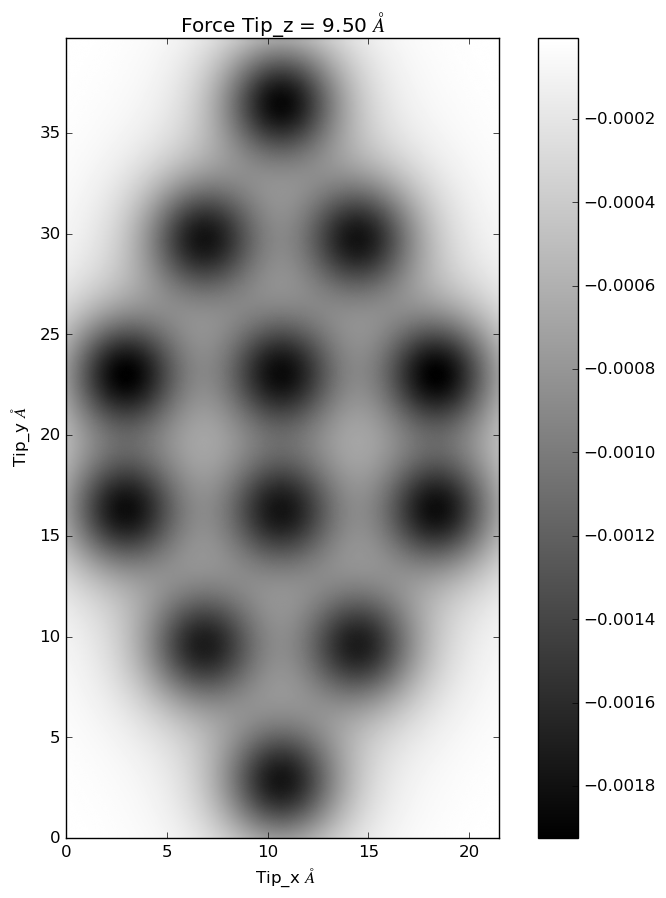

Supplement: File 3 — Datasets A0=1A adatoms_only k=0.5. [file Beilstein_J_Nanotechnol-07-937-s003.zip › S3/A0=1A/adatoms_only/k=0.5/results/Force_0010.png]

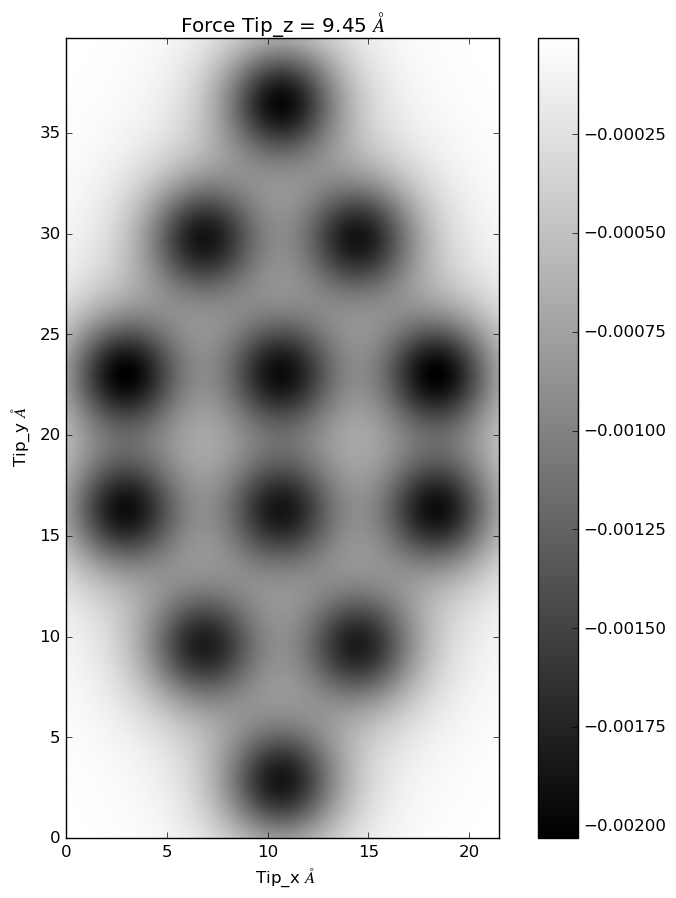

Supplement: File 3 — Datasets A0=1A adatoms_only k=0.5. [file Beilstein_J_Nanotechnol-07-937-s003.zip › S3/A0=1A/adatoms_only/k=0.5/results/Force_0011.png]

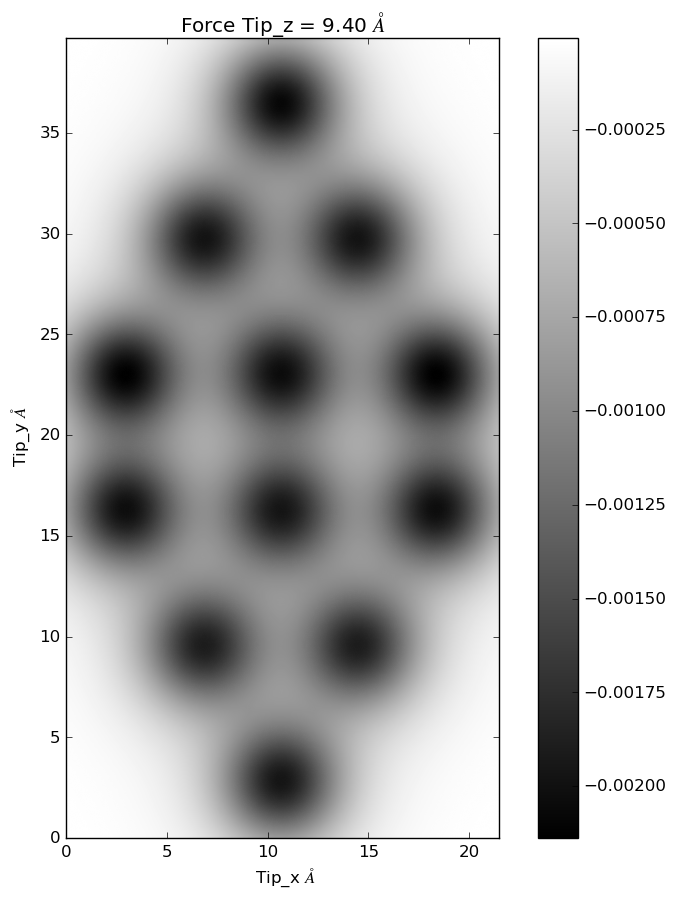

Supplement: File 3 — Datasets A0=1A adatoms_only k=0.5. [file Beilstein_J_Nanotechnol-07-937-s003.zip › S3/A0=1A/adatoms_only/k=0.5/results/Force_0012.png]

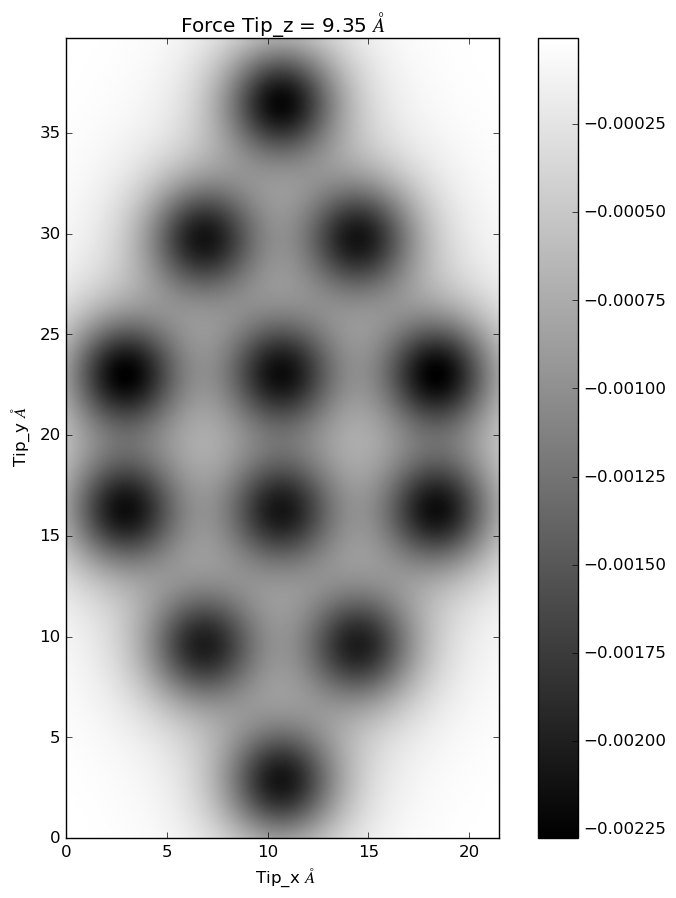

Supplement: File 3 — Datasets A0=1A adatoms_only k=0.5. [file Beilstein_J_Nanotechnol-07-937-s003.zip › S3/A0=1A/adatoms_only/k=0.5/results/Force_0013.png]

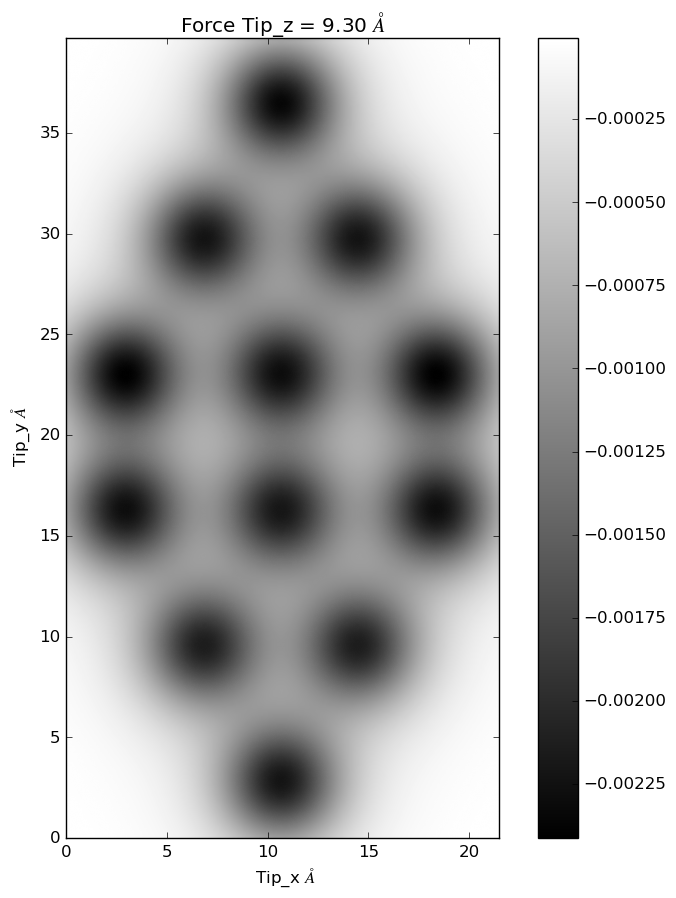

Supplement: File 3 — Datasets A0=1A adatoms_only k=0.5. [file Beilstein_J_Nanotechnol-07-937-s003.zip › S3/A0=1A/adatoms_only/k=0.5/results/Force_0014.png]

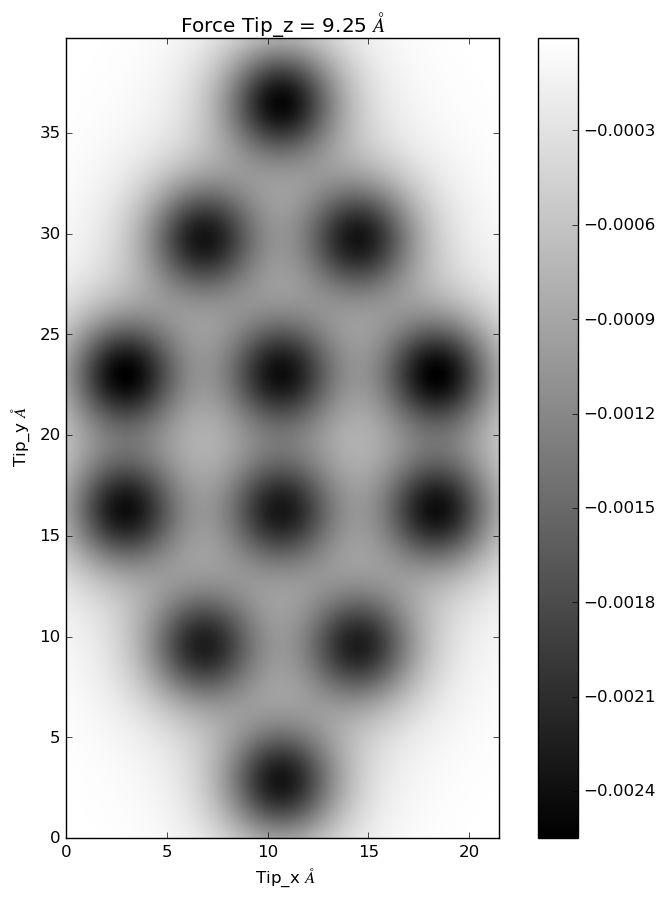

Supplement: File 3 — Datasets A0=1A adatoms_only k=0.5. [file Beilstein_J_Nanotechnol-07-937-s003.zip › S3/A0=1A/adatoms_only/k=0.5/results/Force_0015.png]

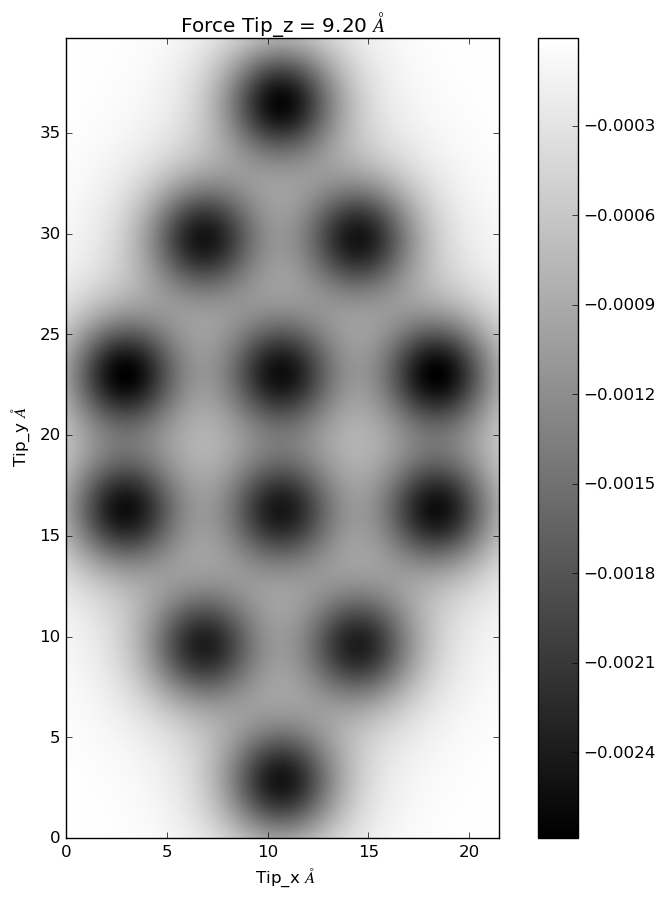

Supplement: File 3 — Datasets A0=1A adatoms_only k=0.5. [file Beilstein_J_Nanotechnol-07-937-s003.zip › S3/A0=1A/adatoms_only/k=0.5/results/Force_0016.png]

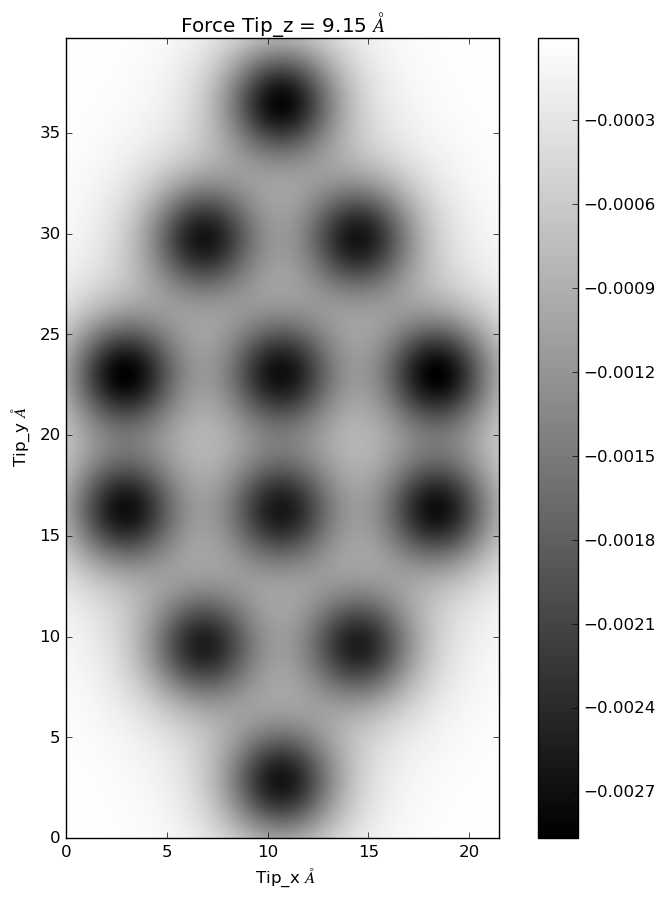

Supplement: File 3 — Datasets A0=1A adatoms_only k=0.5. [file Beilstein_J_Nanotechnol-07-937-s003.zip › S3/A0=1A/adatoms_only/k=0.5/results/Force_0017.png]

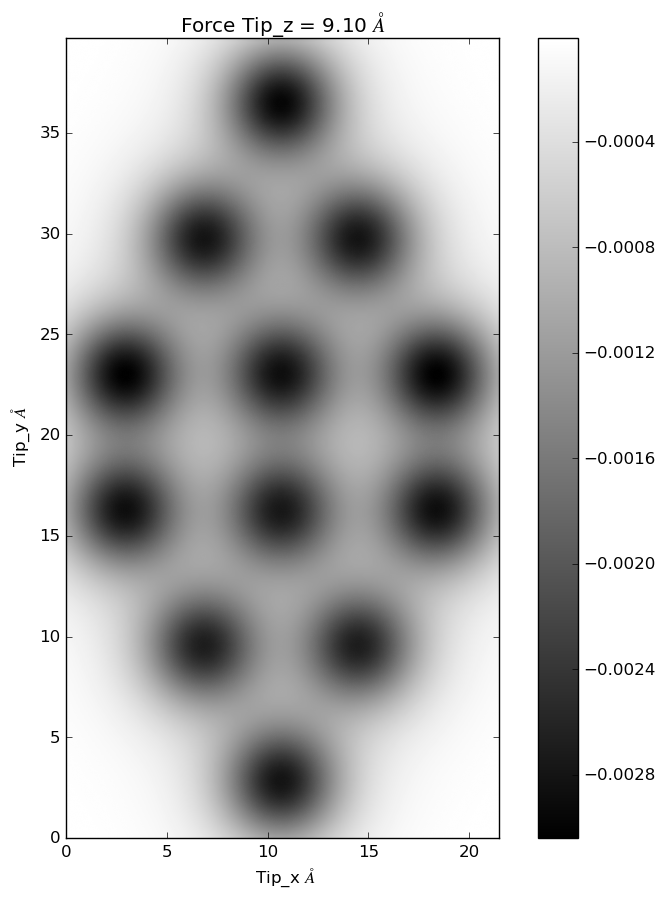

Supplement: File 3 — Datasets A0=1A adatoms_only k=0.5. [file Beilstein_J_Nanotechnol-07-937-s003.zip › S3/A0=1A/adatoms_only/k=0.5/results/Force_0018.png]

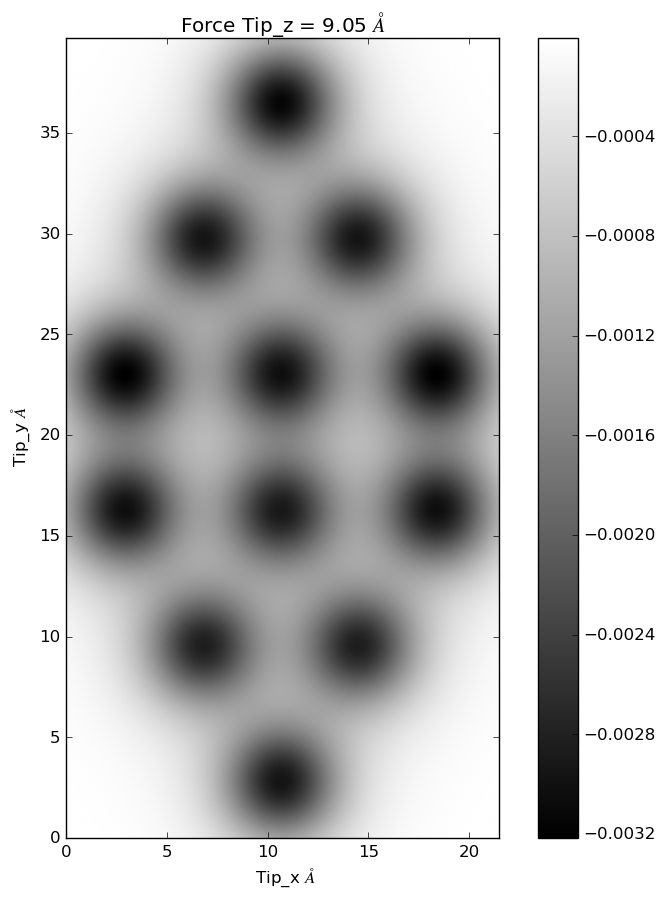

Supplement: File 3 — Datasets A0=1A adatoms_only k=0.5. [file Beilstein_J_Nanotechnol-07-937-s003.zip › S3/A0=1A/adatoms_only/k=0.5/results/Force_0019.png]

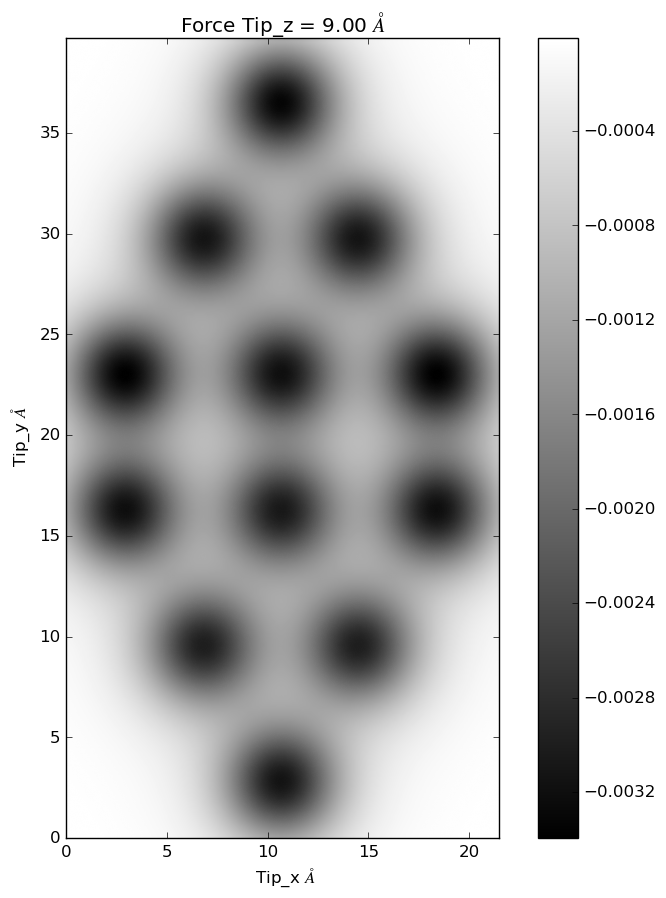

Supplement: File 3 — Datasets A0=1A adatoms_only k=0.5. [file Beilstein_J_Nanotechnol-07-937-s003.zip › S3/A0=1A/adatoms_only/k=0.5/results/Force_0020.png]

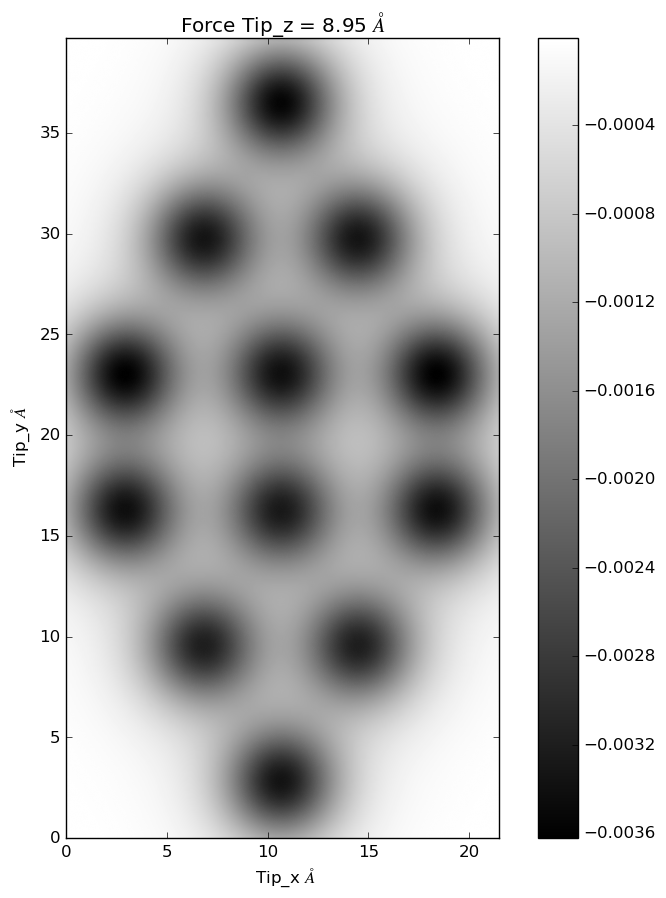

Supplement: File 3 — Datasets A0=1A adatoms_only k=0.5. [file Beilstein_J_Nanotechnol-07-937-s003.zip › S3/A0=1A/adatoms_only/k=0.5/results/Force_0021.png]

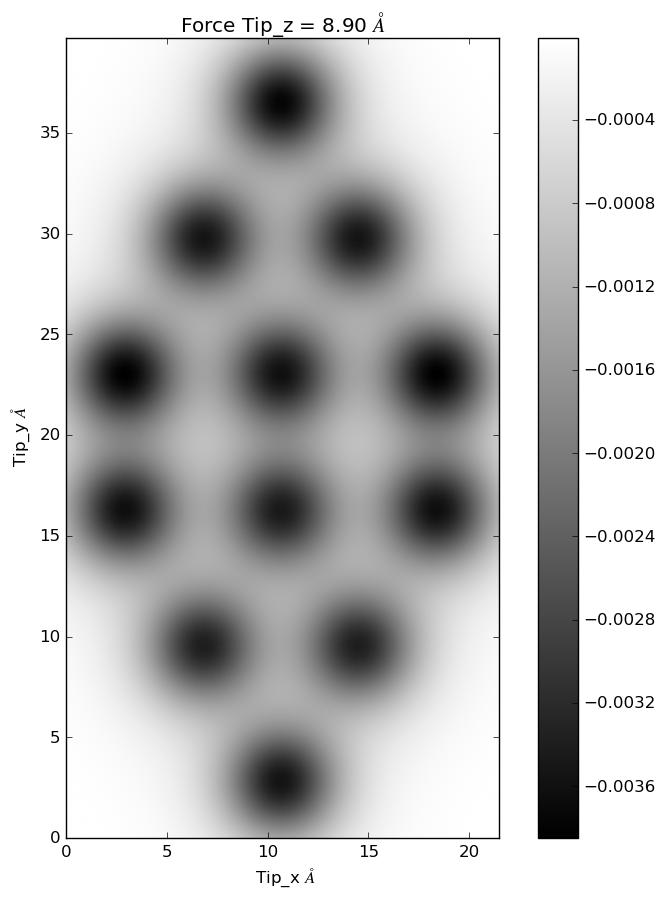

Supplement: File 3 — Datasets A0=1A adatoms_only k=0.5. [file Beilstein_J_Nanotechnol-07-937-s003.zip › S3/A0=1A/adatoms_only/k=0.5/results/Force_0022.png]

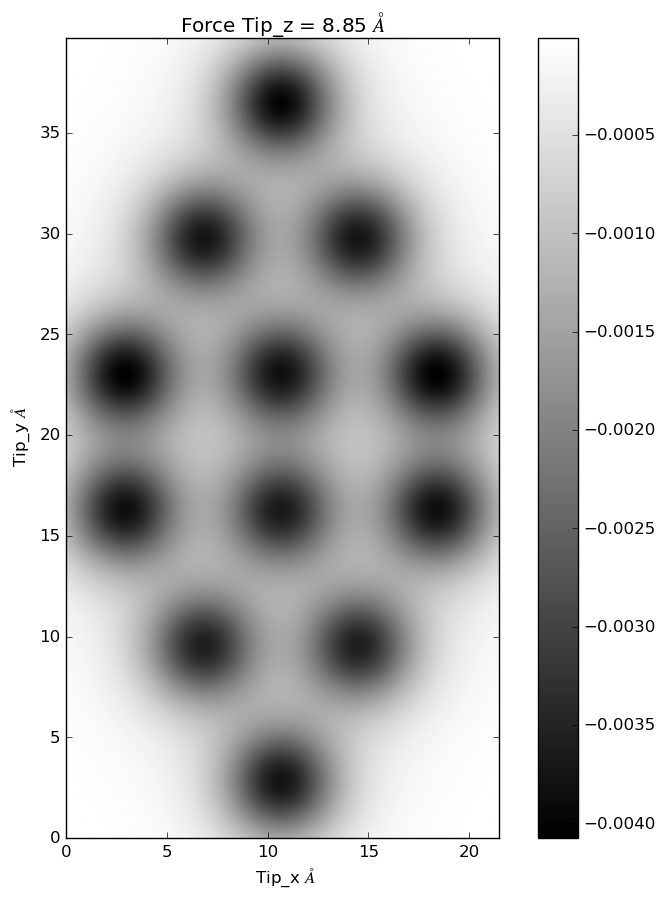

Supplement: File 3 — Datasets A0=1A adatoms_only k=0.5. [file Beilstein_J_Nanotechnol-07-937-s003.zip › S3/A0=1A/adatoms_only/k=0.5/results/Force_0023.png]

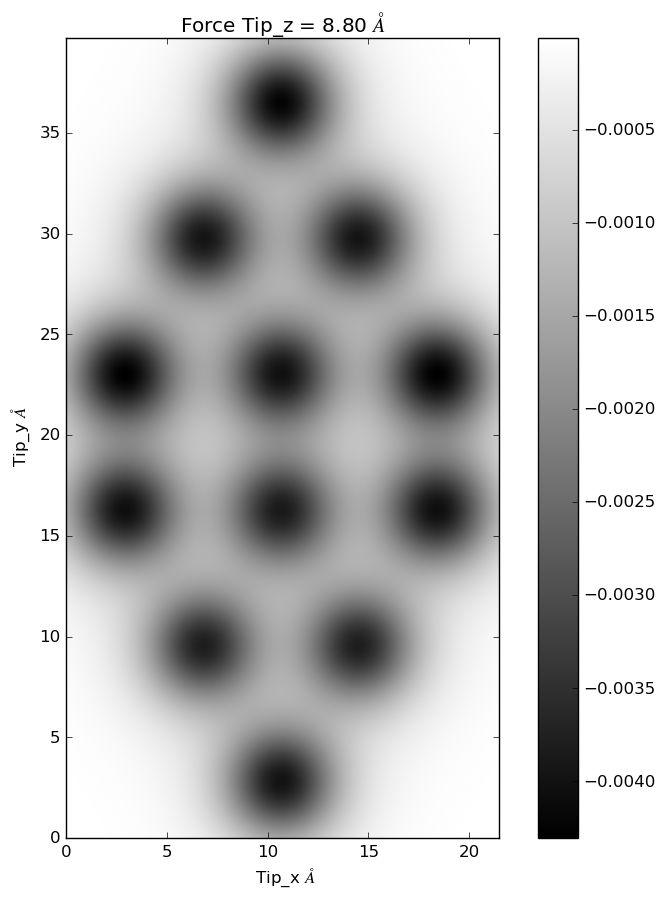

Supplement: File 3 — Datasets A0=1A adatoms_only k=0.5. [file Beilstein_J_Nanotechnol-07-937-s003.zip › S3/A0=1A/adatoms_only/k=0.5/results/Force_0024.png]

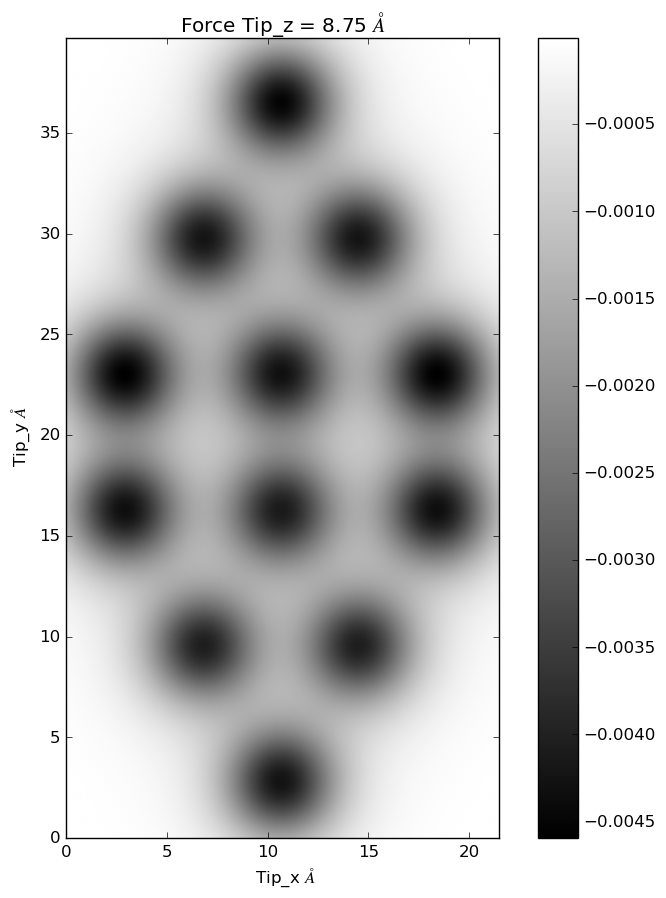

Supplement: File 3 — Datasets A0=1A adatoms_only k=0.5. [file Beilstein_J_Nanotechnol-07-937-s003.zip › S3/A0=1A/adatoms_only/k=0.5/results/Force_0025.png]

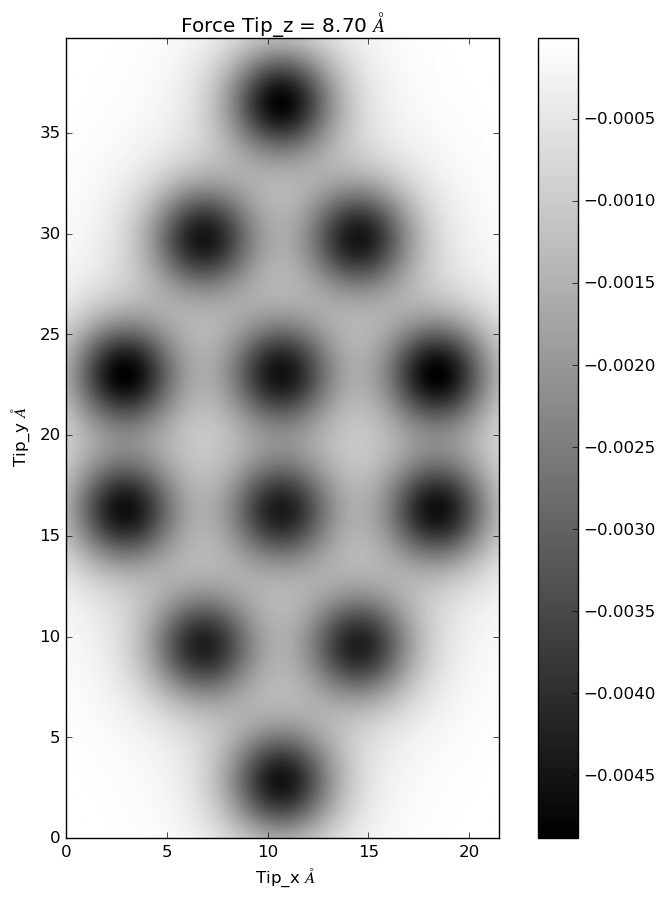

Supplement: File 3 — Datasets A0=1A adatoms_only k=0.5. [file Beilstein_J_Nanotechnol-07-937-s003.zip › S3/A0=1A/adatoms_only/k=0.5/results/Force_0026.png]

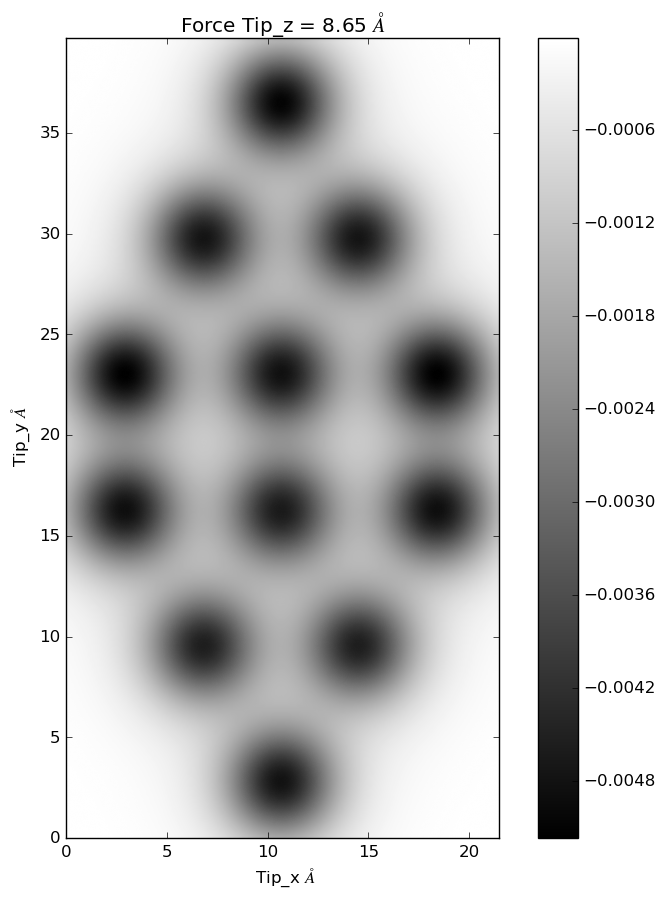

Supplement: File 3 — Datasets A0=1A adatoms_only k=0.5. [file Beilstein_J_Nanotechnol-07-937-s003.zip › S3/A0=1A/adatoms_only/k=0.5/results/Force_0027.png]

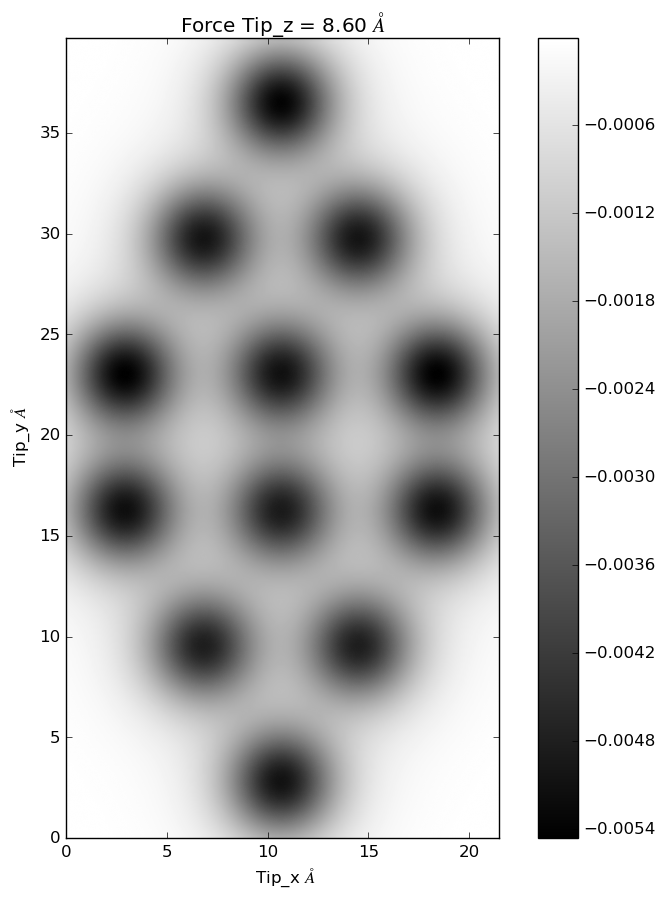

Supplement: File 3 — Datasets A0=1A adatoms_only k=0.5. [file Beilstein_J_Nanotechnol-07-937-s003.zip › S3/A0=1A/adatoms_only/k=0.5/results/Force_0028.png]

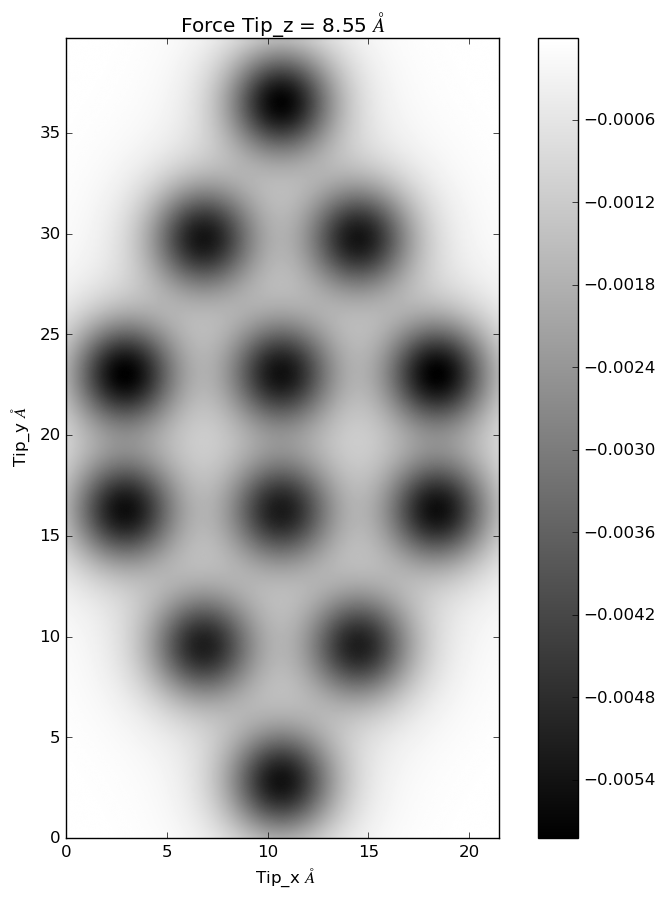

Supplement: File 3 — Datasets A0=1A adatoms_only k=0.5. [file Beilstein_J_Nanotechnol-07-937-s003.zip › S3/A0=1A/adatoms_only/k=0.5/results/Force_0029.png]

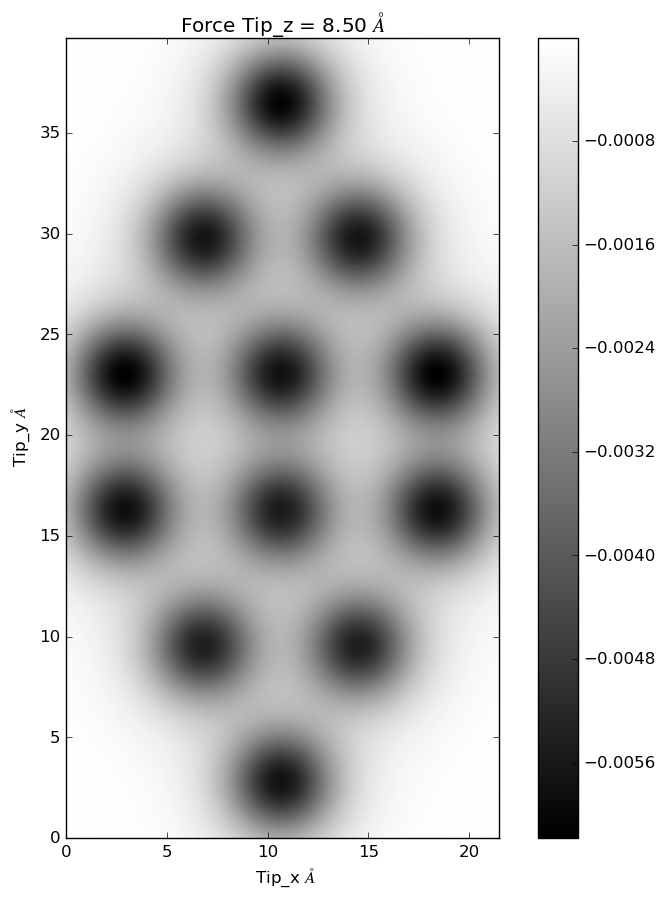

Supplement: File 3 — Datasets A0=1A adatoms_only k=0.5. [file Beilstein_J_Nanotechnol-07-937-s003.zip › S3/A0=1A/adatoms_only/k=0.5/results/Force_0030.png]

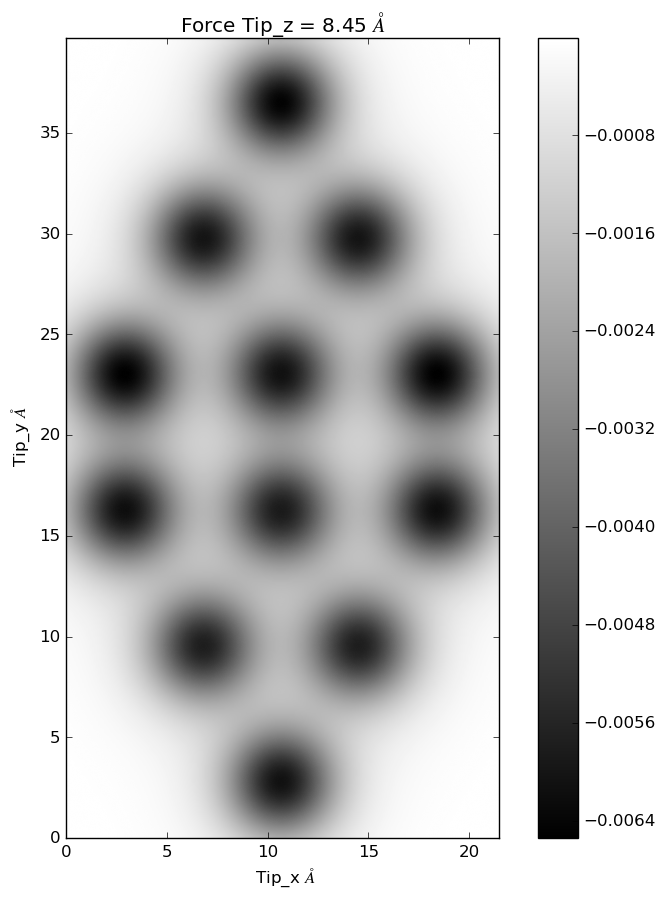

Supplement: File 3 — Datasets A0=1A adatoms_only k=0.5. [file Beilstein_J_Nanotechnol-07-937-s003.zip › S3/A0=1A/adatoms_only/k=0.5/results/Force_0031.png]

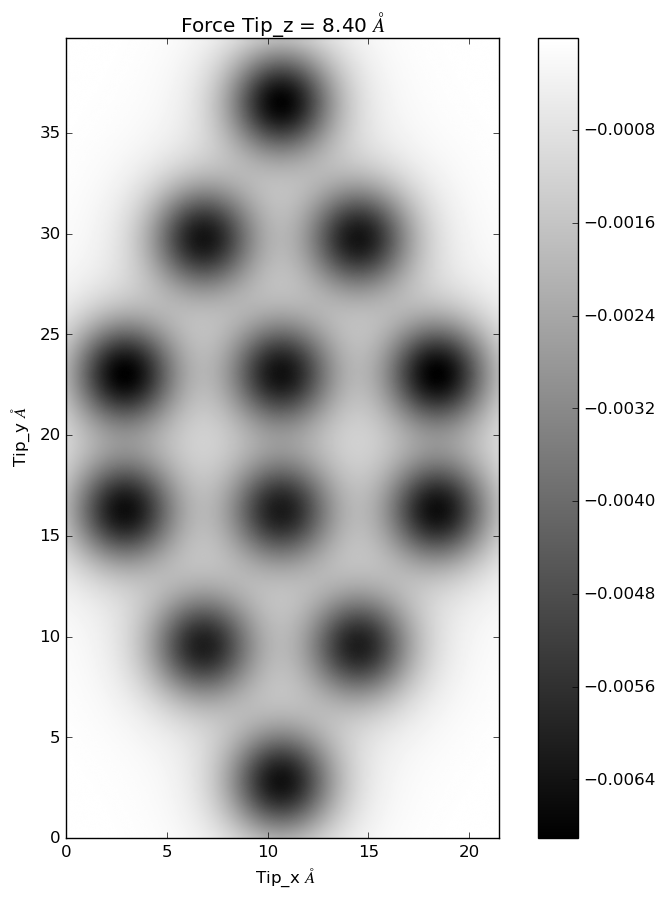

Supplement: File 3 — Datasets A0=1A adatoms_only k=0.5. [file Beilstein_J_Nanotechnol-07-937-s003.zip › S3/A0=1A/adatoms_only/k=0.5/results/Force_0032.png]

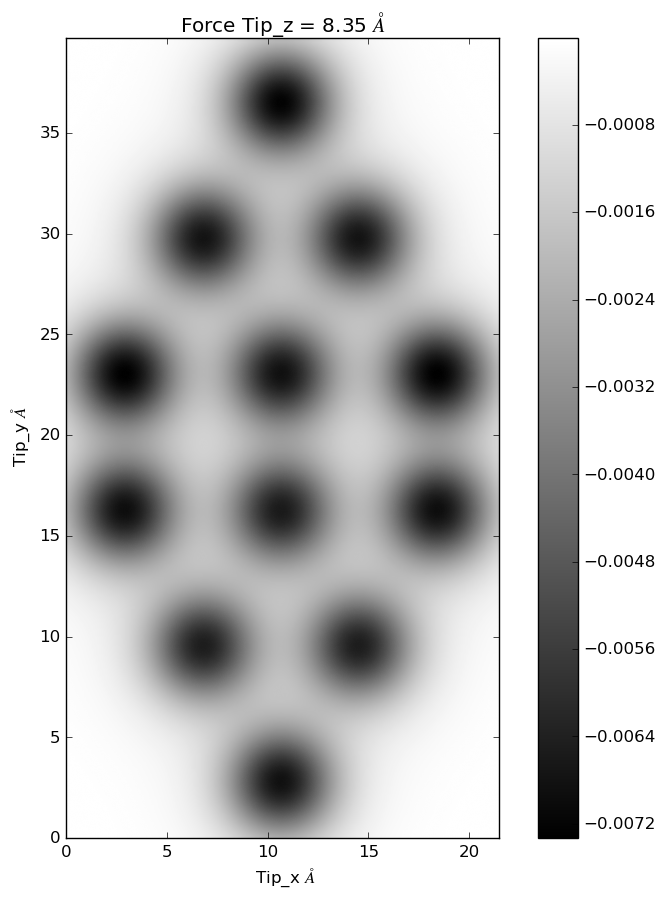

Supplement: File 3 — Datasets A0=1A adatoms_only k=0.5. [file Beilstein_J_Nanotechnol-07-937-s003.zip › S3/A0=1A/adatoms_only/k=0.5/results/Force_0033.png]

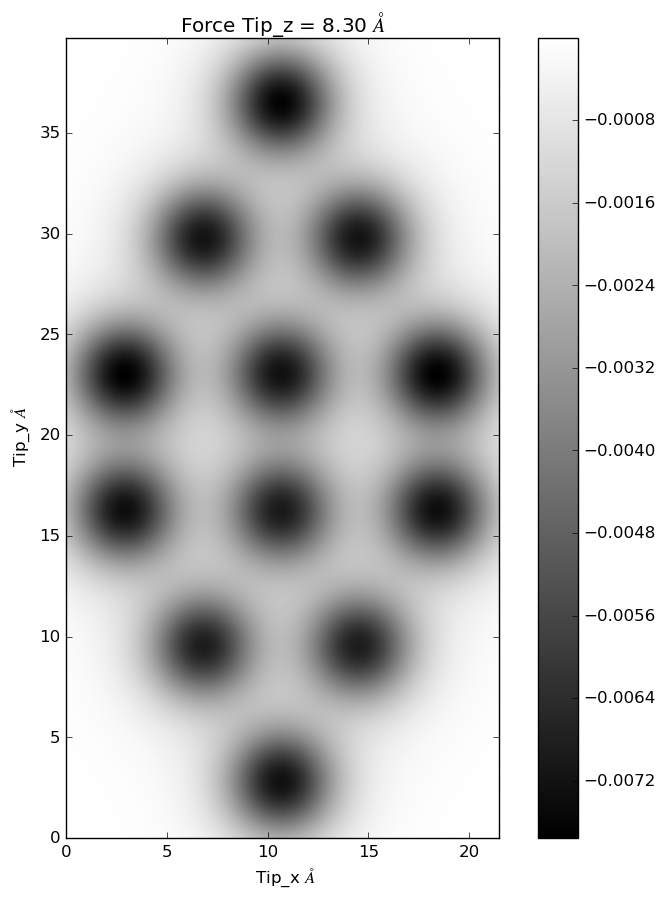

Supplement: File 3 — Datasets A0=1A adatoms_only k=0.5. [file Beilstein_J_Nanotechnol-07-937-s003.zip › S3/A0=1A/adatoms_only/k=0.5/results/Force_0034.png]

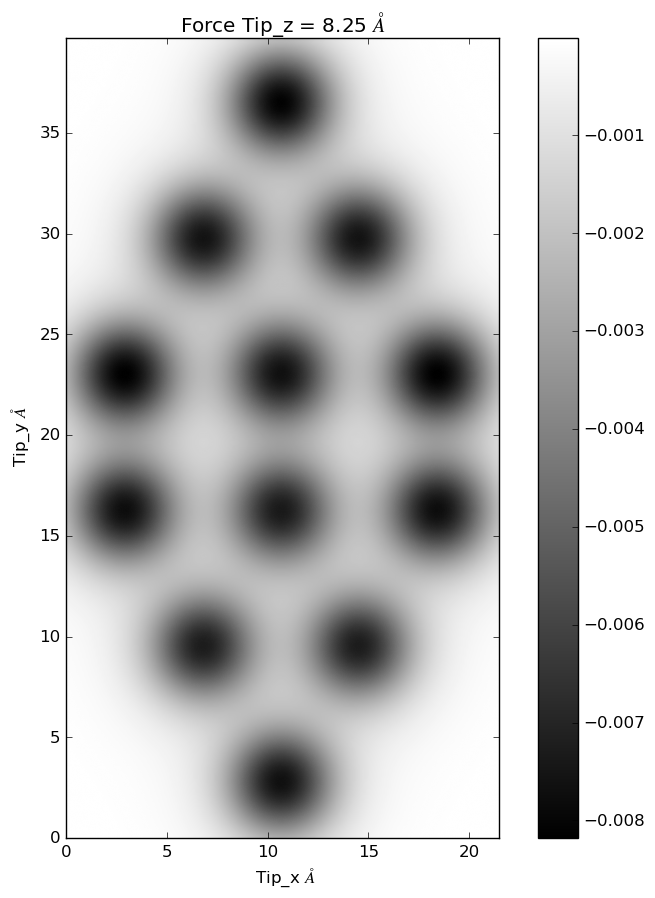

Supplement: File 3 — Datasets A0=1A adatoms_only k=0.5. [file Beilstein_J_Nanotechnol-07-937-s003.zip › S3/A0=1A/adatoms_only/k=0.5/results/Force_0035.png]

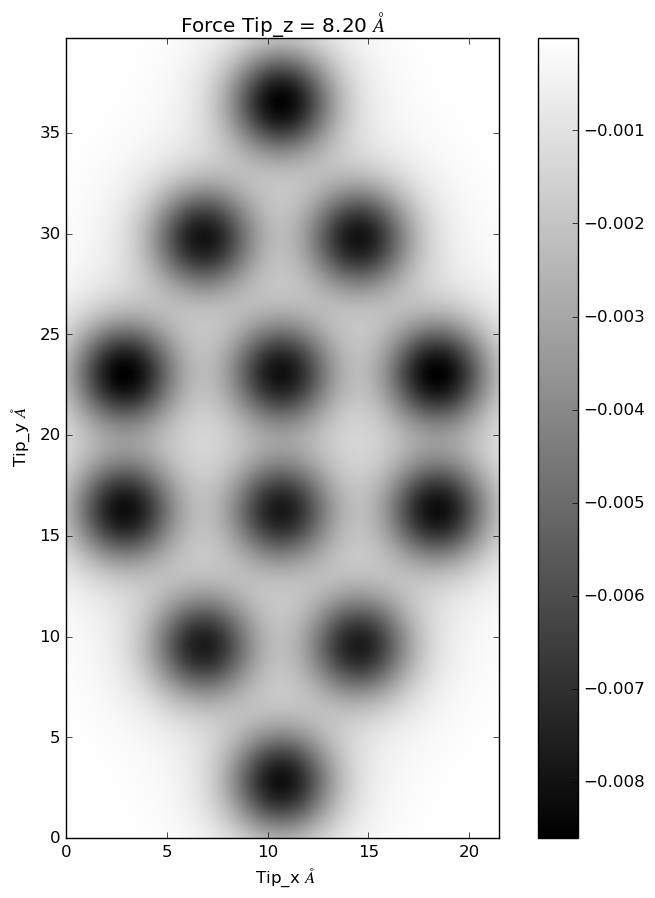

Supplement: File 3 — Datasets A0=1A adatoms_only k=0.5. [file Beilstein_J_Nanotechnol-07-937-s003.zip › S3/A0=1A/adatoms_only/k=0.5/results/Force_0036.png]

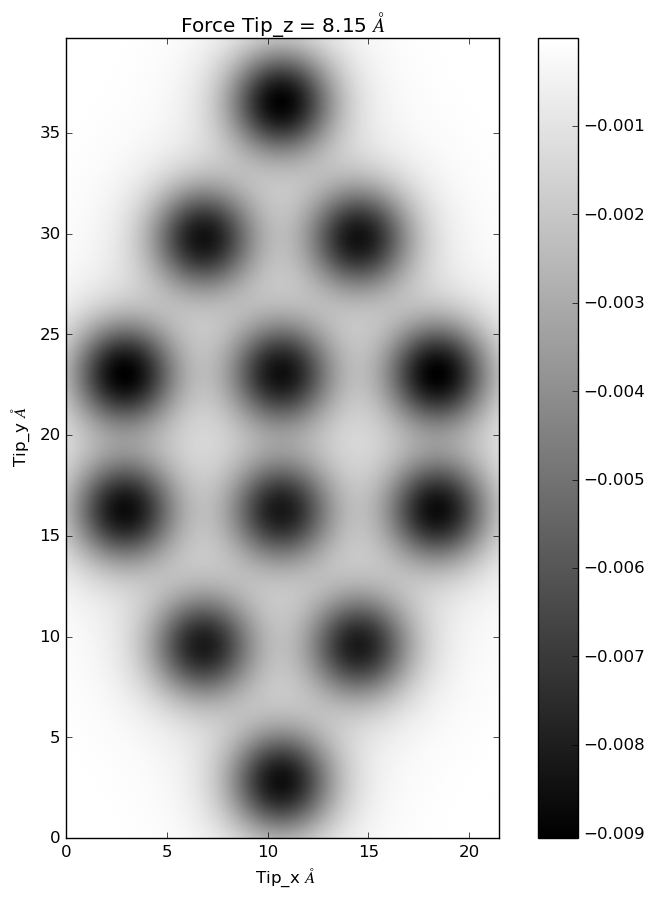

Supplement: File 3 — Datasets A0=1A adatoms_only k=0.5. [file Beilstein_J_Nanotechnol-07-937-s003.zip › S3/A0=1A/adatoms_only/k=0.5/results/Force_0037.png]

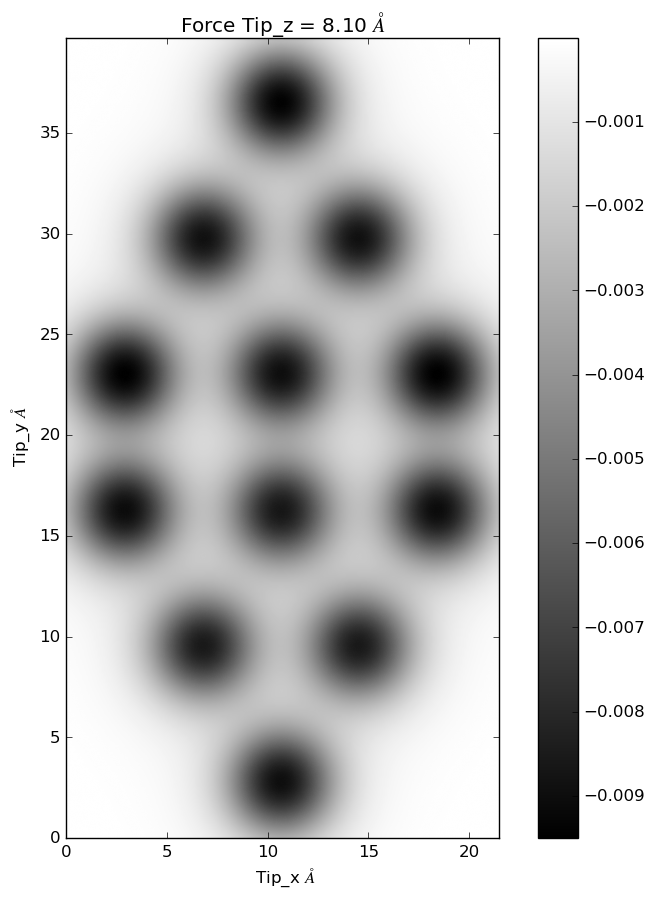

Supplement: File 3 — Datasets A0=1A adatoms_only k=0.5. [file Beilstein_J_Nanotechnol-07-937-s003.zip › S3/A0=1A/adatoms_only/k=0.5/results/Force_0038.png]

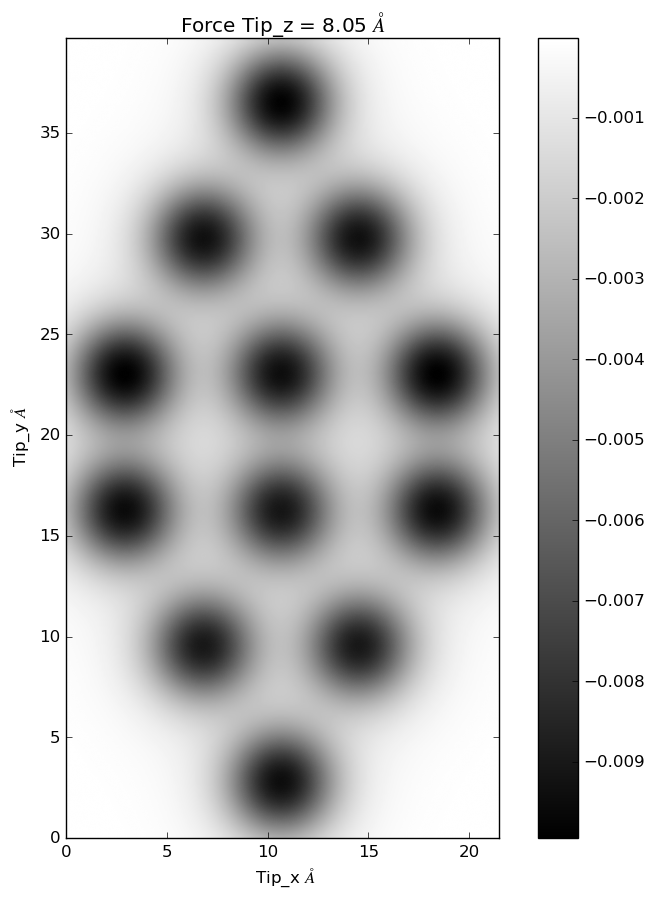

Supplement: File 3 — Datasets A0=1A adatoms_only k=0.5. [file Beilstein_J_Nanotechnol-07-937-s003.zip › S3/A0=1A/adatoms_only/k=0.5/results/Force_0039.png]

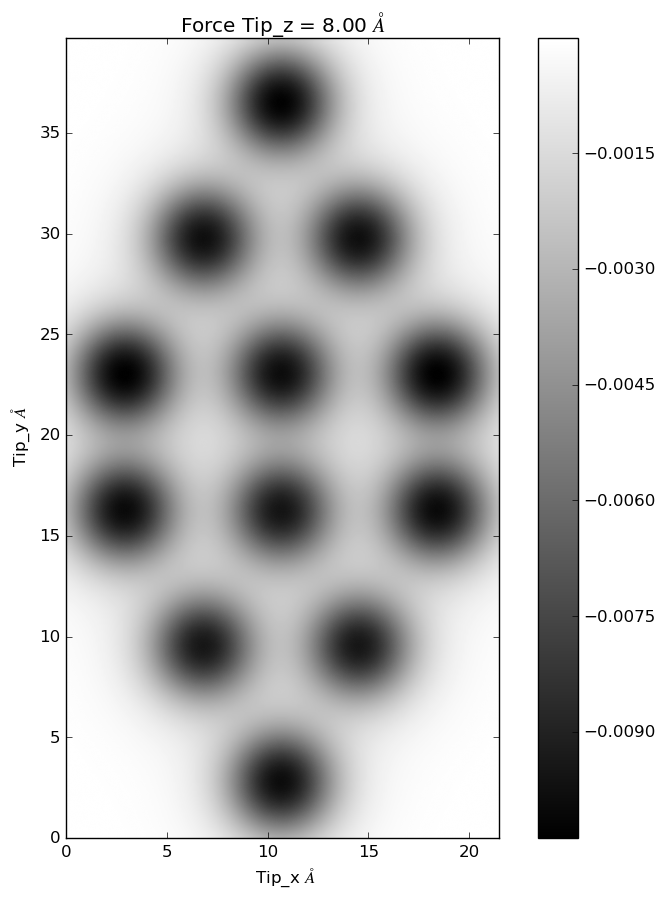

Supplement: File 3 — Datasets A0=1A adatoms_only k=0.5. [file Beilstein_J_Nanotechnol-07-937-s003.zip › S3/A0=1A/adatoms_only/k=0.5/results/Force_0040.png]

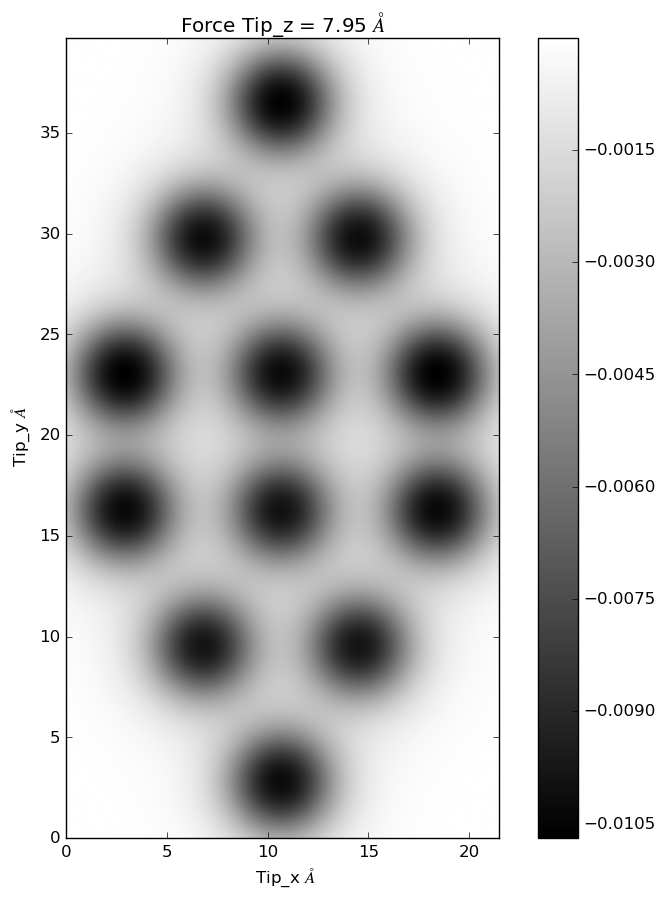

Supplement: File 3 — Datasets A0=1A adatoms_only k=0.5. [file Beilstein_J_Nanotechnol-07-937-s003.zip › S3/A0=1A/adatoms_only/k=0.5/results/Force_0041.png]

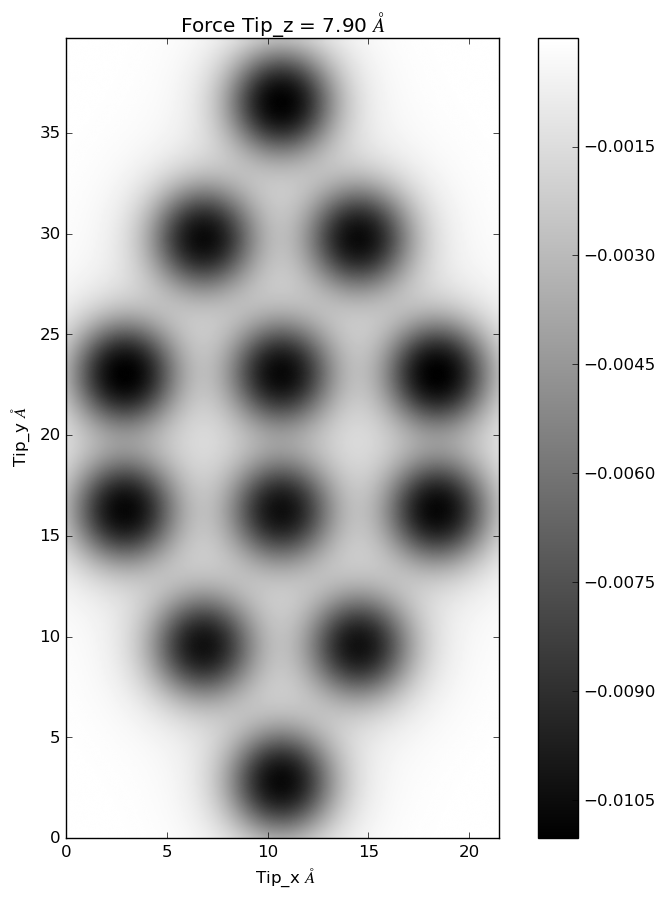

Supplement: File 3 — Datasets A0=1A adatoms_only k=0.5. [file Beilstein_J_Nanotechnol-07-937-s003.zip › S3/A0=1A/adatoms_only/k=0.5/results/Force_0042.png]

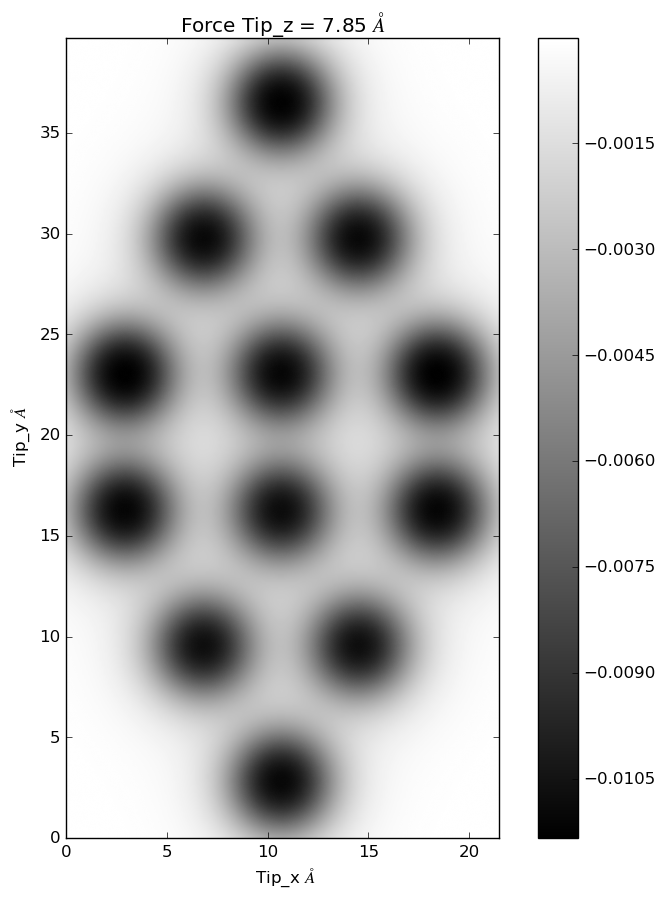

Supplement: File 3 — Datasets A0=1A adatoms_only k=0.5. [file Beilstein_J_Nanotechnol-07-937-s003.zip › S3/A0=1A/adatoms_only/k=0.5/results/Force_0043.png]

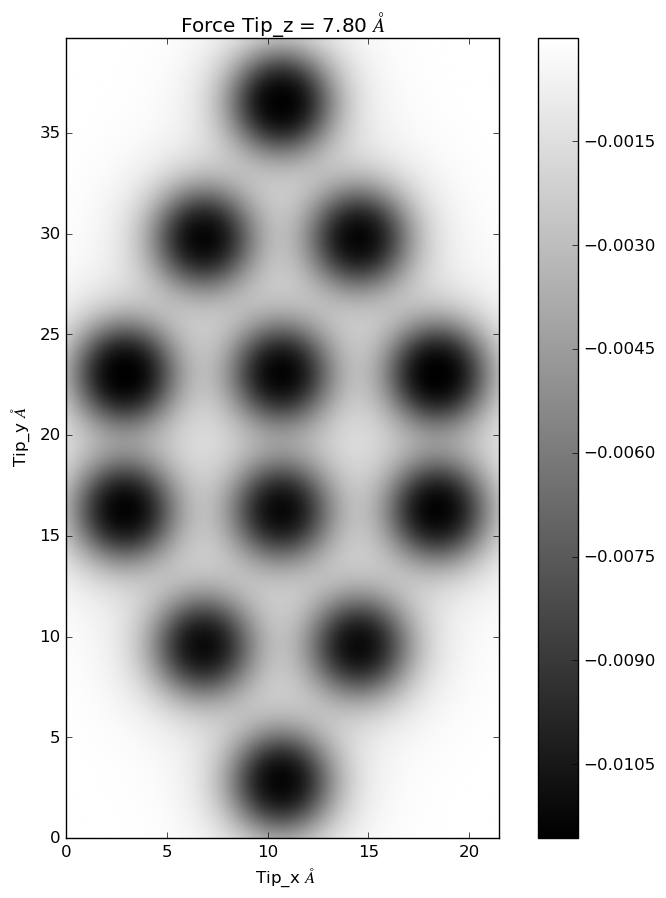

Supplement: File 3 — Datasets A0=1A adatoms_only k=0.5. [file Beilstein_J_Nanotechnol-07-937-s003.zip › S3/A0=1A/adatoms_only/k=0.5/results/Force_0044.png]

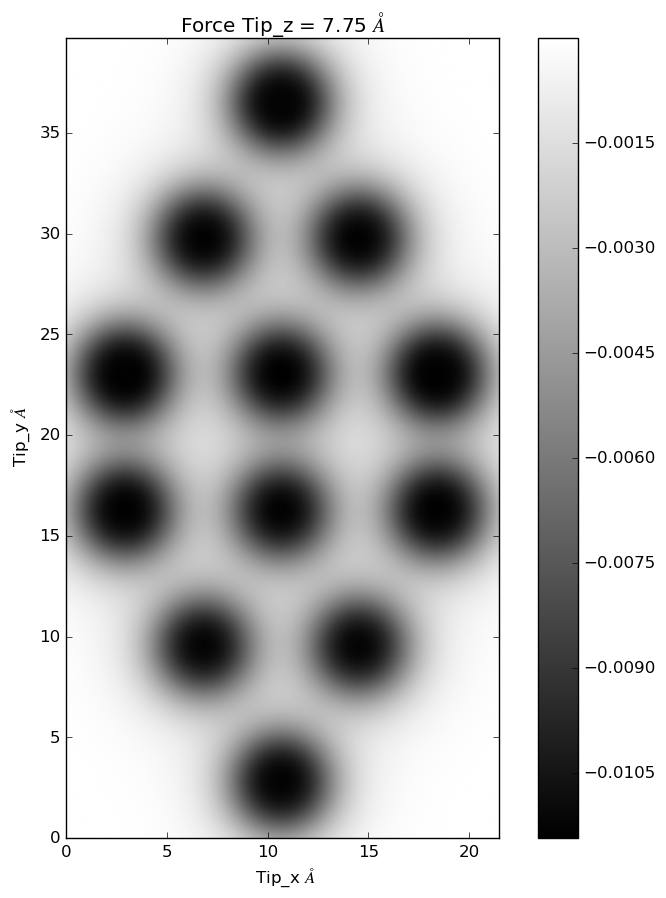

Supplement: File 3 — Datasets A0=1A adatoms_only k=0.5. [file Beilstein_J_Nanotechnol-07-937-s003.zip › S3/A0=1A/adatoms_only/k=0.5/results/Force_0045.png]

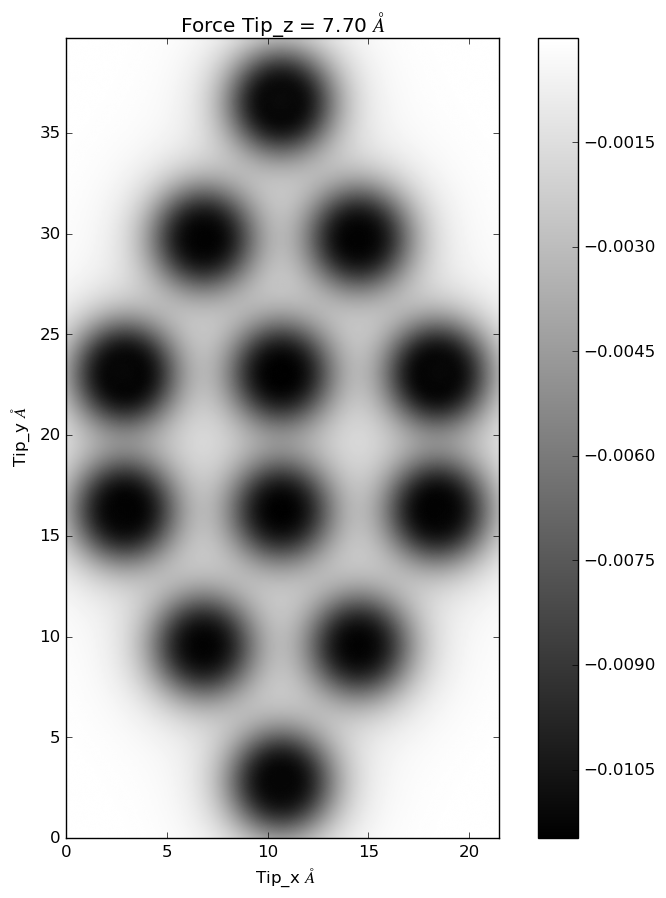

Supplement: File 3 — Datasets A0=1A adatoms_only k=0.5. [file Beilstein_J_Nanotechnol-07-937-s003.zip › S3/A0=1A/adatoms_only/k=0.5/results/Force_0046.png]

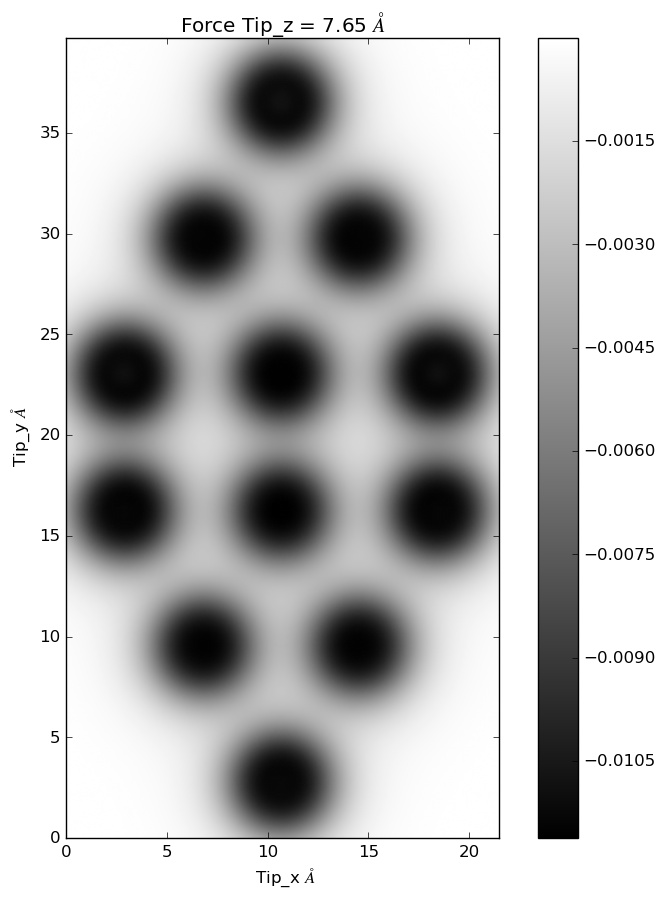

Supplement: File 3 — Datasets A0=1A adatoms_only k=0.5. [file Beilstein_J_Nanotechnol-07-937-s003.zip › S3/A0=1A/adatoms_only/k=0.5/results/Force_0047.png]

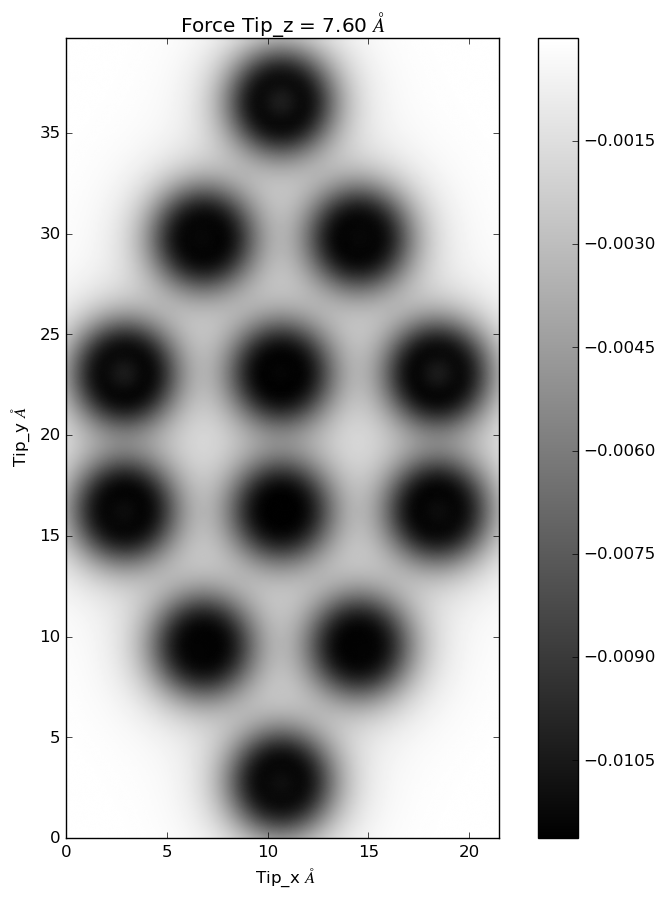

Supplement: File 3 — Datasets A0=1A adatoms_only k=0.5. [file Beilstein_J_Nanotechnol-07-937-s003.zip › S3/A0=1A/adatoms_only/k=0.5/results/Force_0048.png]

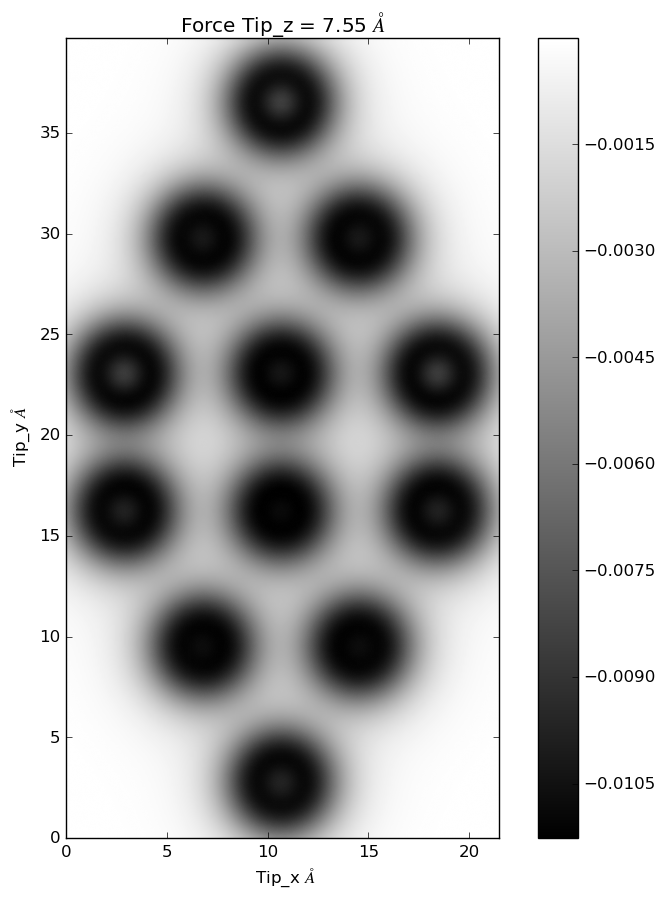

Supplement: File 3 — Datasets A0=1A adatoms_only k=0.5. [file Beilstein_J_Nanotechnol-07-937-s003.zip › S3/A0=1A/adatoms_only/k=0.5/results/Force_0049.png]

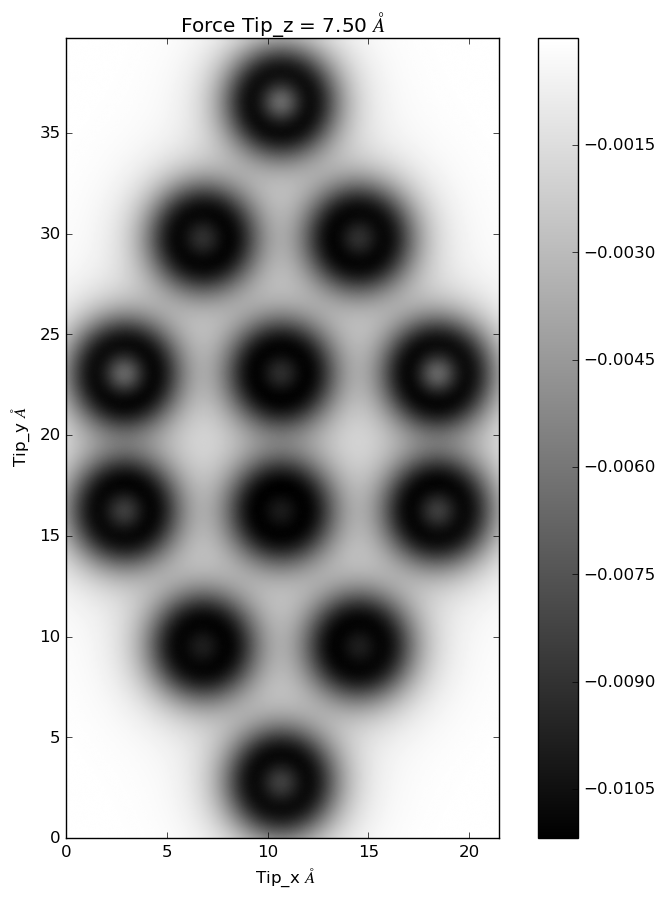

Supplement: File 3 — Datasets A0=1A adatoms_only k=0.5. [file Beilstein_J_Nanotechnol-07-937-s003.zip › S3/A0=1A/adatoms_only/k=0.5/results/Force_0050.png]

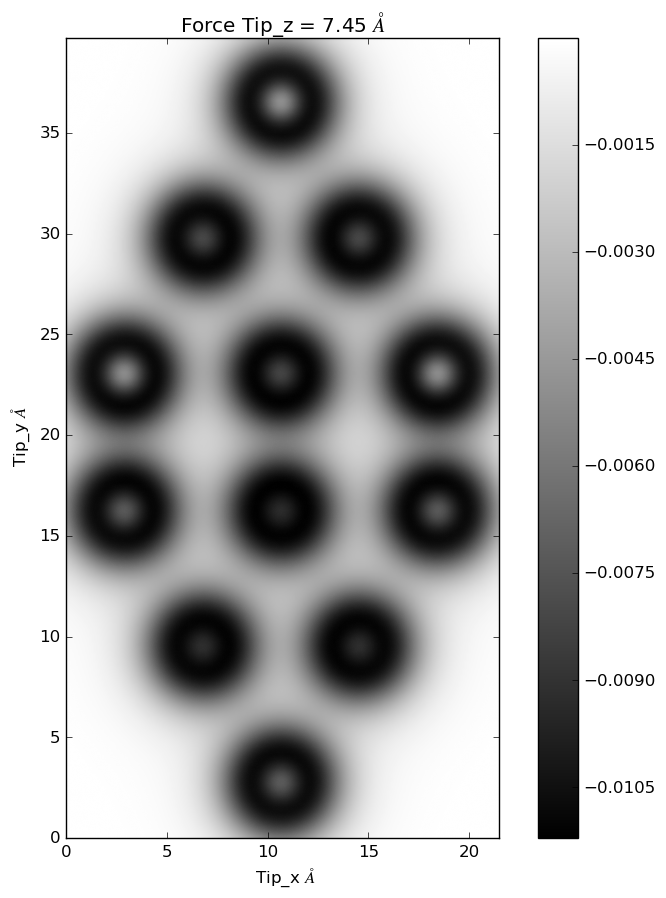

Supplement: File 3 — Datasets A0=1A adatoms_only k=0.5. [file Beilstein_J_Nanotechnol-07-937-s003.zip › S3/A0=1A/adatoms_only/k=0.5/results/Force_0051.png]

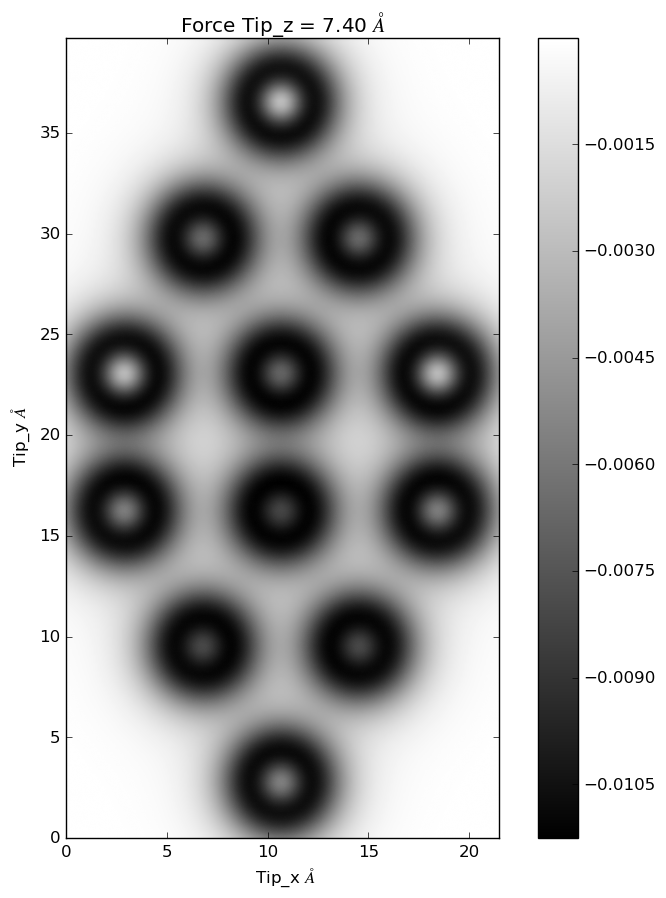

Supplement: File 3 — Datasets A0=1A adatoms_only k=0.5. [file Beilstein_J_Nanotechnol-07-937-s003.zip › S3/A0=1A/adatoms_only/k=0.5/results/Force_0052.png]

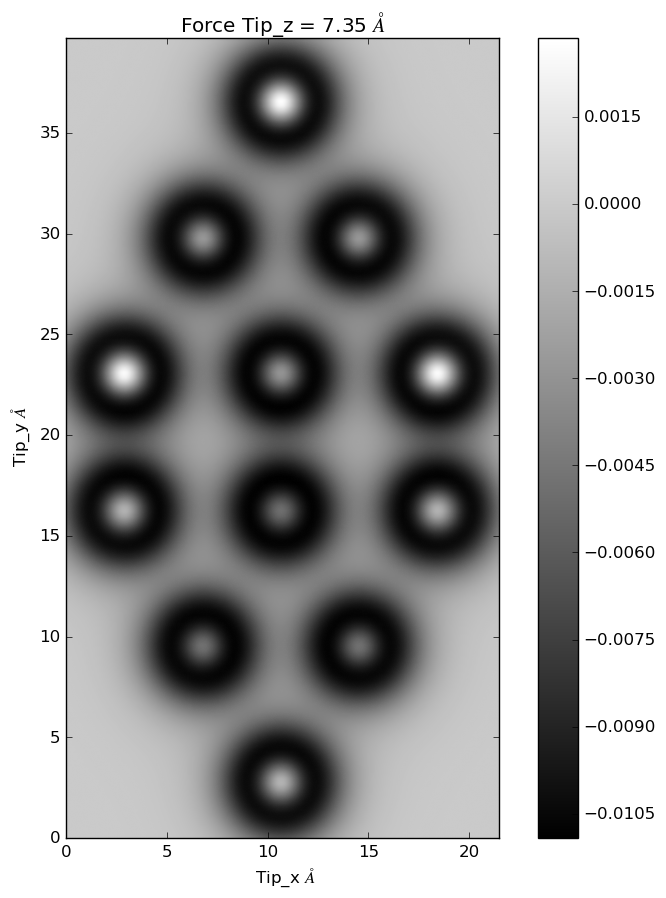

Supplement: File 3 — Datasets A0=1A adatoms_only k=0.5. [file Beilstein_J_Nanotechnol-07-937-s003.zip › S3/A0=1A/adatoms_only/k=0.5/results/Force_0053.png]

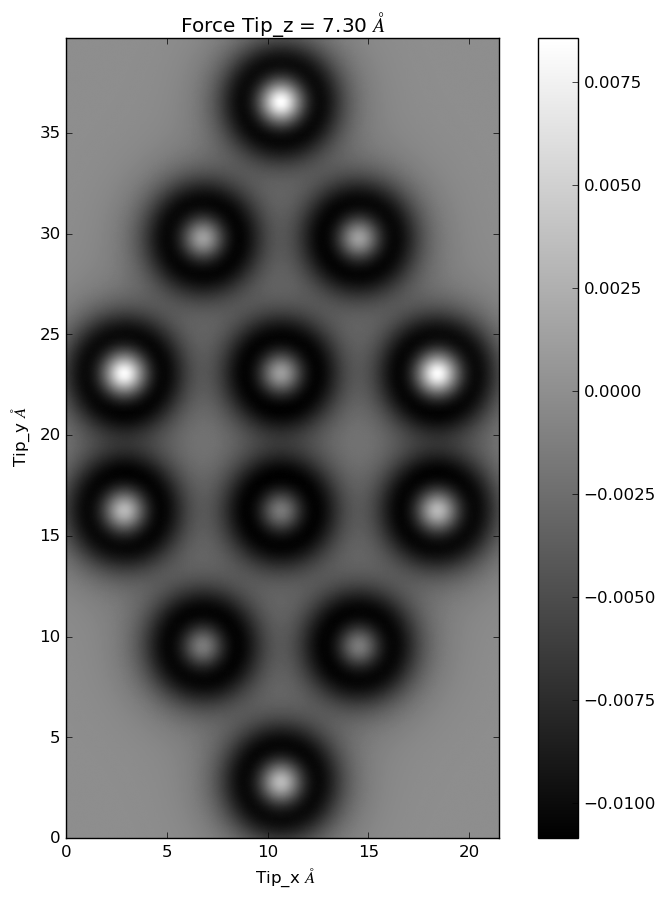

Supplement: File 3 — Datasets A0=1A adatoms_only k=0.5. [file Beilstein_J_Nanotechnol-07-937-s003.zip › S3/A0=1A/adatoms_only/k=0.5/results/Force_0054.png]

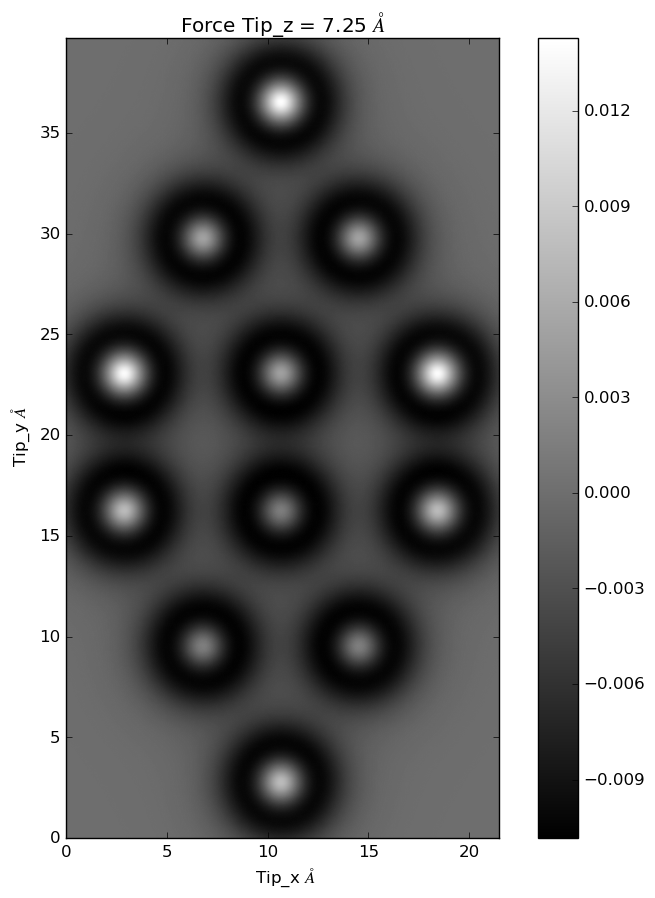

Supplement: File 3 — Datasets A0=1A adatoms_only k=0.5. [file Beilstein_J_Nanotechnol-07-937-s003.zip › S3/A0=1A/adatoms_only/k=0.5/results/Force_0055.png]

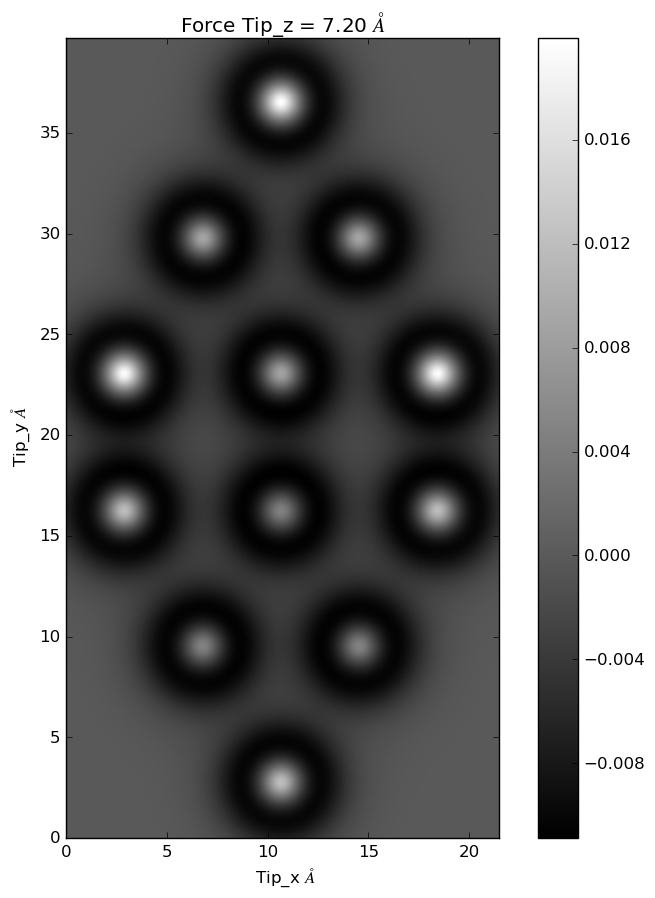

Supplement: File 3 — Datasets A0=1A adatoms_only k=0.5. [file Beilstein_J_Nanotechnol-07-937-s003.zip › S3/A0=1A/adatoms_only/k=0.5/results/Force_0056.png]

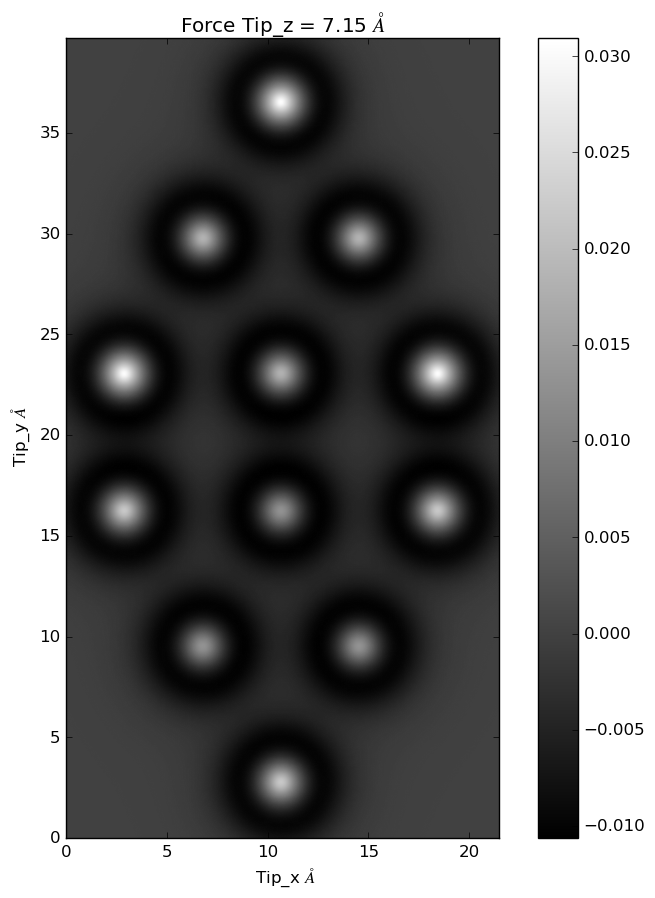

Supplement: File 3 — Datasets A0=1A adatoms_only k=0.5. [file Beilstein_J_Nanotechnol-07-937-s003.zip › S3/A0=1A/adatoms_only/k=0.5/results/Force_0057.png]

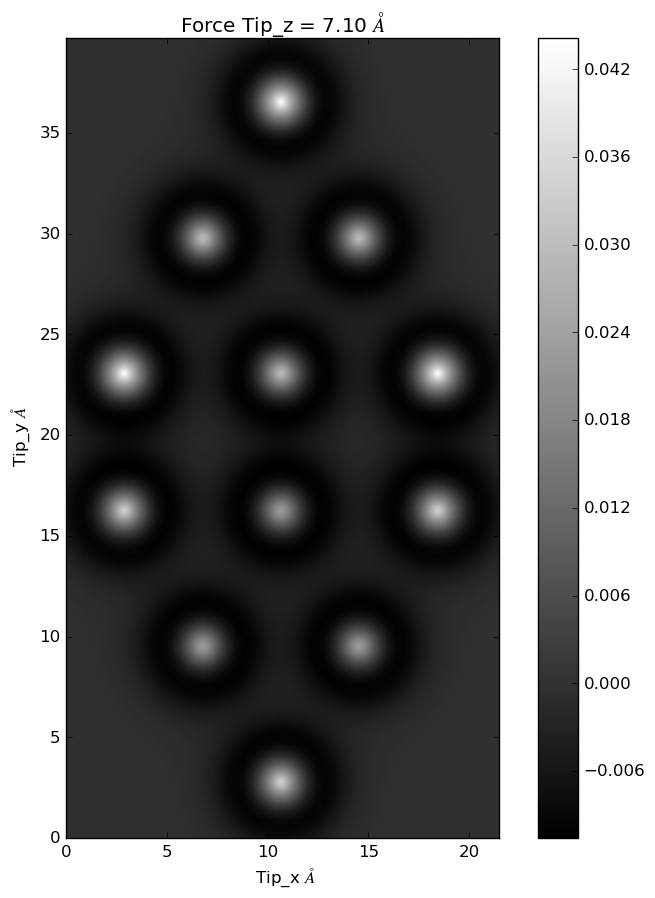

Supplement: File 3 — Datasets A0=1A adatoms_only k=0.5. [file Beilstein_J_Nanotechnol-07-937-s003.zip › S3/A0=1A/adatoms_only/k=0.5/results/Force_0058.png]

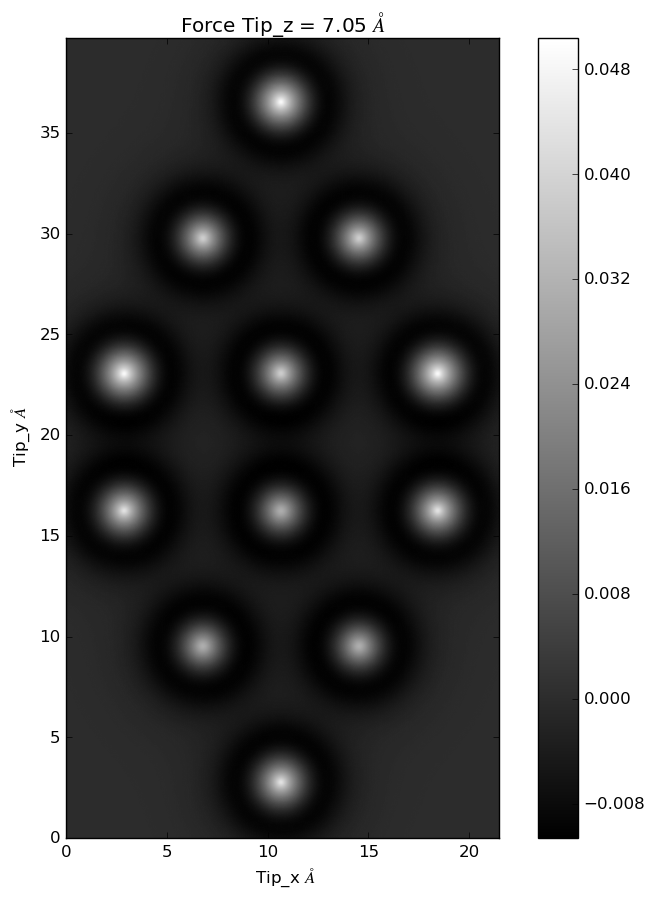

Supplement: File 3 — Datasets A0=1A adatoms_only k=0.5. [file Beilstein_J_Nanotechnol-07-937-s003.zip › S3/A0=1A/adatoms_only/k=0.5/results/Force_0059.png]

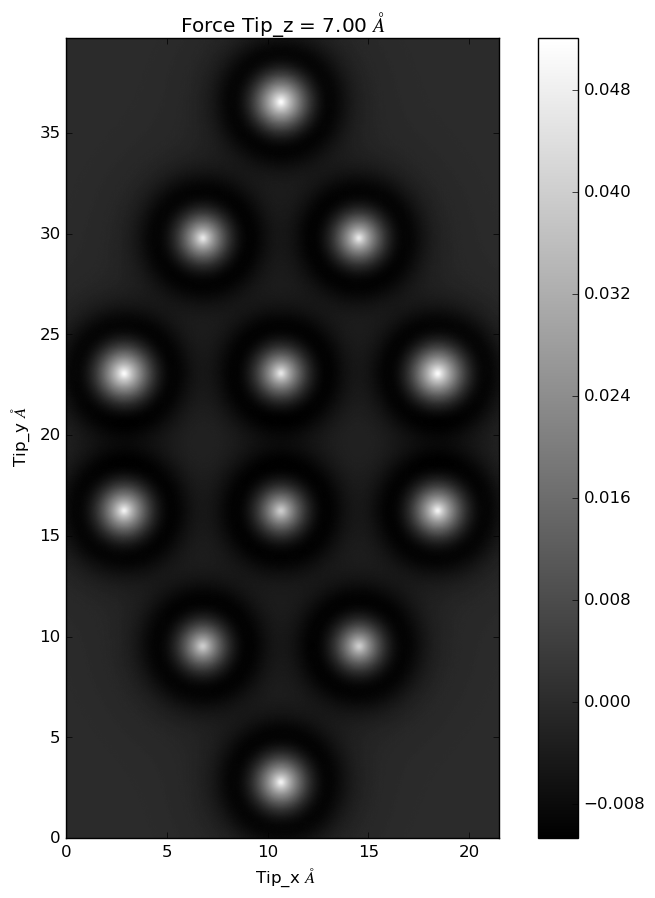

Supplement: File 3 — Datasets A0=1A adatoms_only k=0.5. [file Beilstein_J_Nanotechnol-07-937-s003.zip › S3/A0=1A/adatoms_only/k=0.5/results/Force_0060.png]

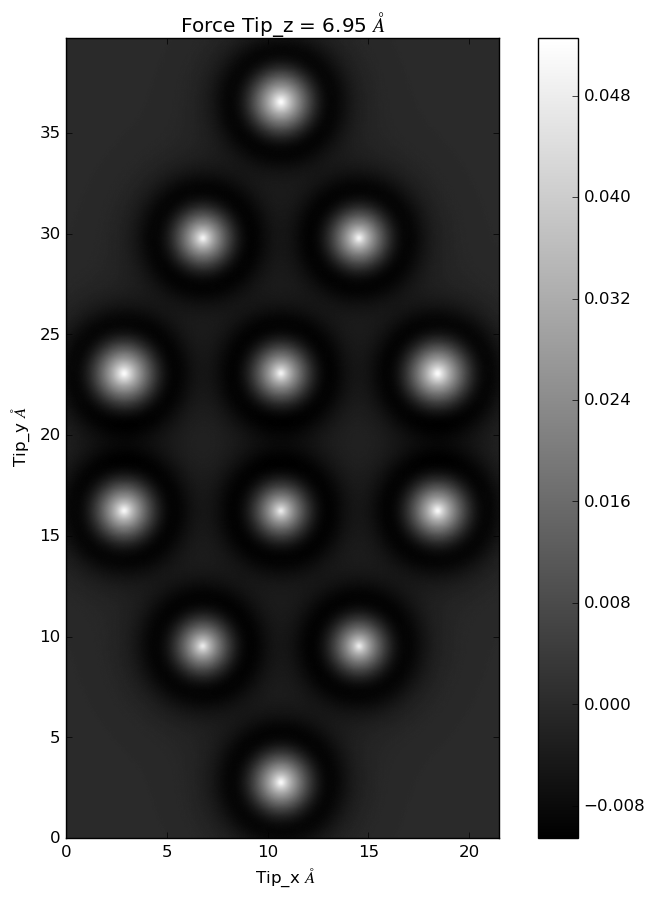

Supplement: File 3 — Datasets A0=1A adatoms_only k=0.5. [file Beilstein_J_Nanotechnol-07-937-s003.zip › S3/A0=1A/adatoms_only/k=0.5/results/Force_0061.png]

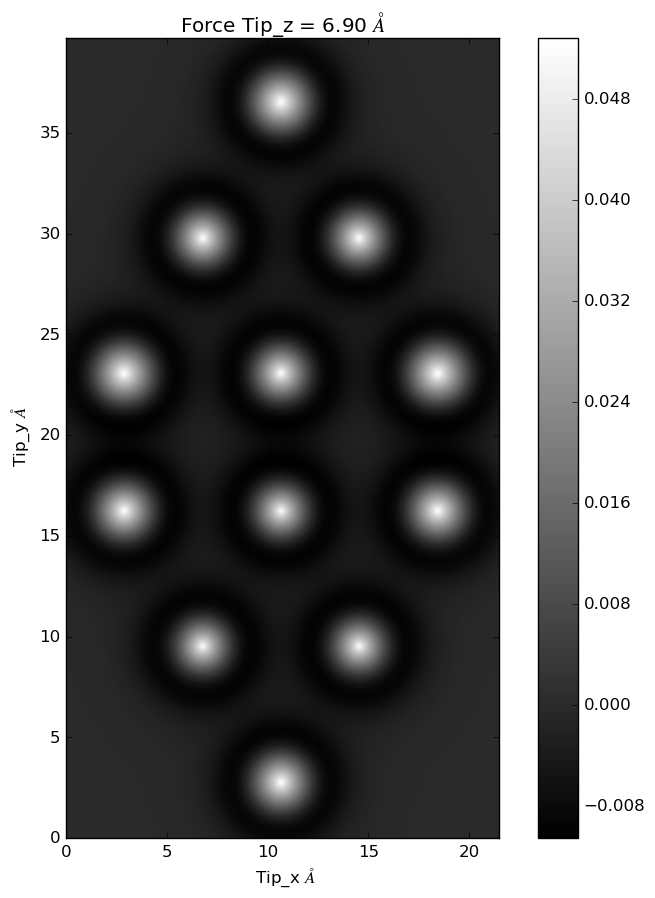

Supplement: File 3 — Datasets A0=1A adatoms_only k=0.5. [file Beilstein_J_Nanotechnol-07-937-s003.zip › S3/A0=1A/adatoms_only/k=0.5/results/Force_0062.png]

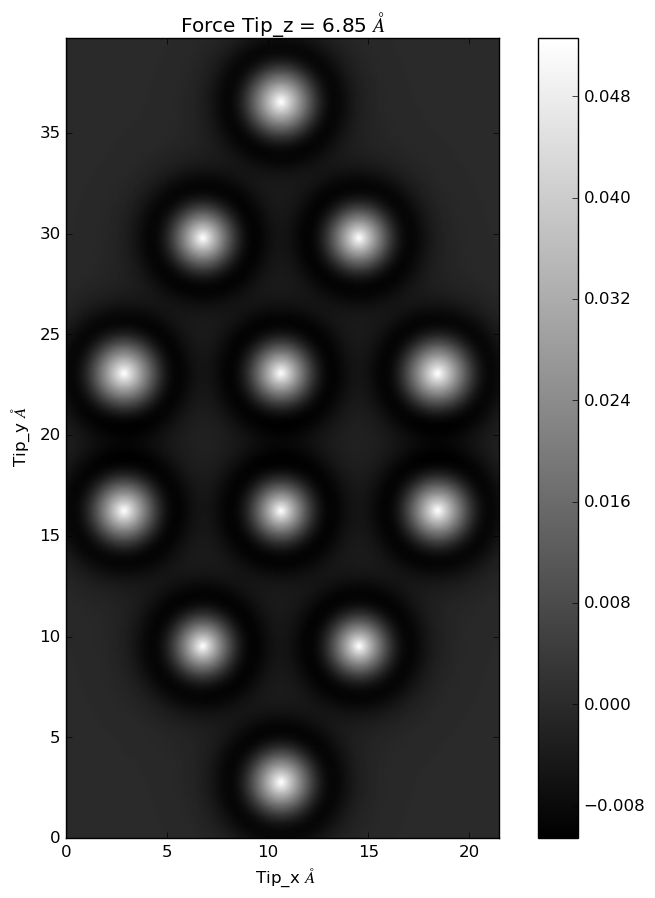

Supplement: File 3 — Datasets A0=1A adatoms_only k=0.5. [file Beilstein_J_Nanotechnol-07-937-s003.zip › S3/A0=1A/adatoms_only/k=0.5/results/Force_0063.png]

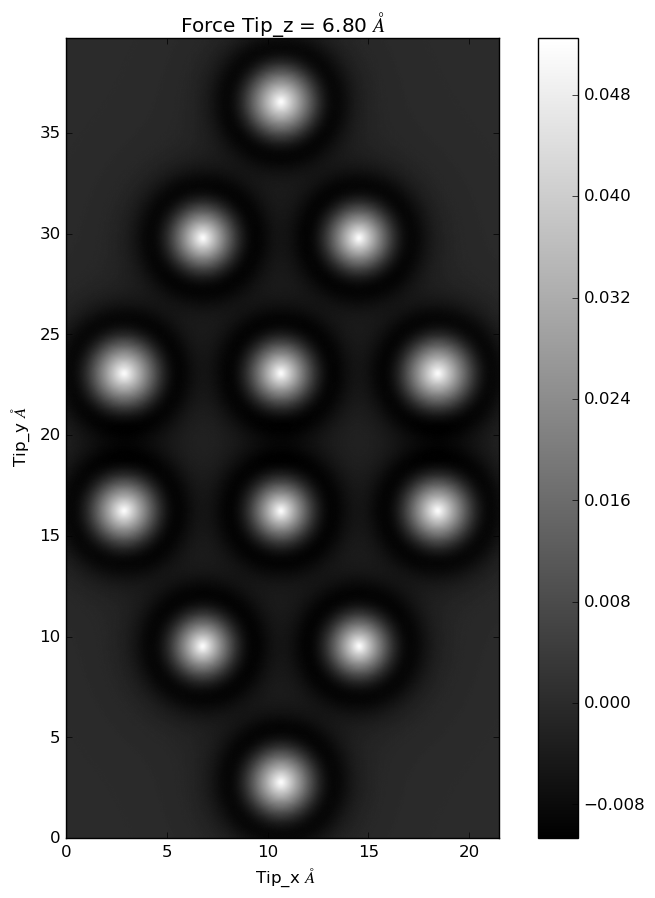

Supplement: File 3 — Datasets A0=1A adatoms_only k=0.5. [file Beilstein_J_Nanotechnol-07-937-s003.zip › S3/A0=1A/adatoms_only/k=0.5/results/Force_0064.png]

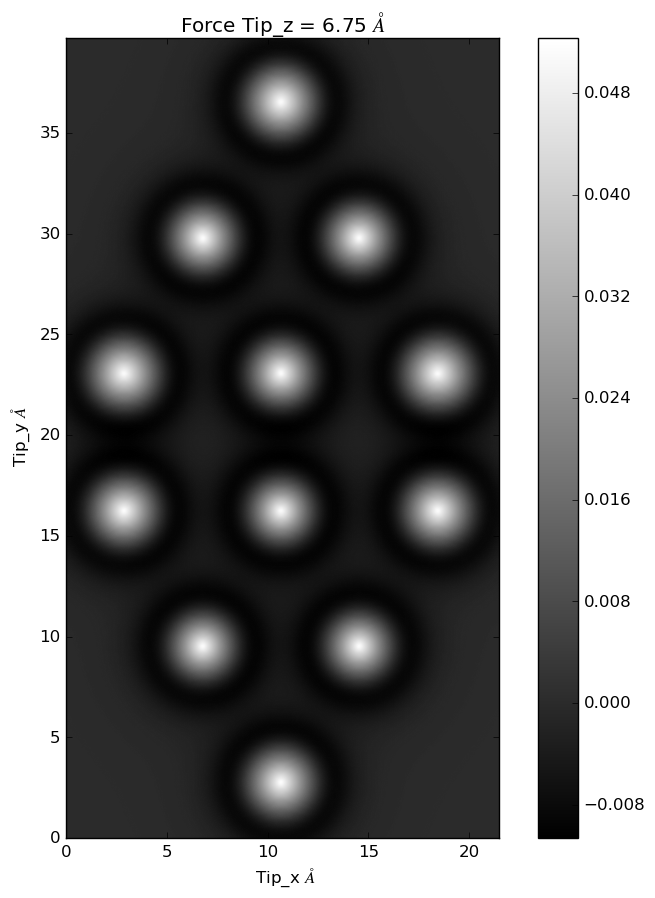

Supplement: File 3 — Datasets A0=1A adatoms_only k=0.5. [file Beilstein_J_Nanotechnol-07-937-s003.zip › S3/A0=1A/adatoms_only/k=0.5/results/Force_0065.png]

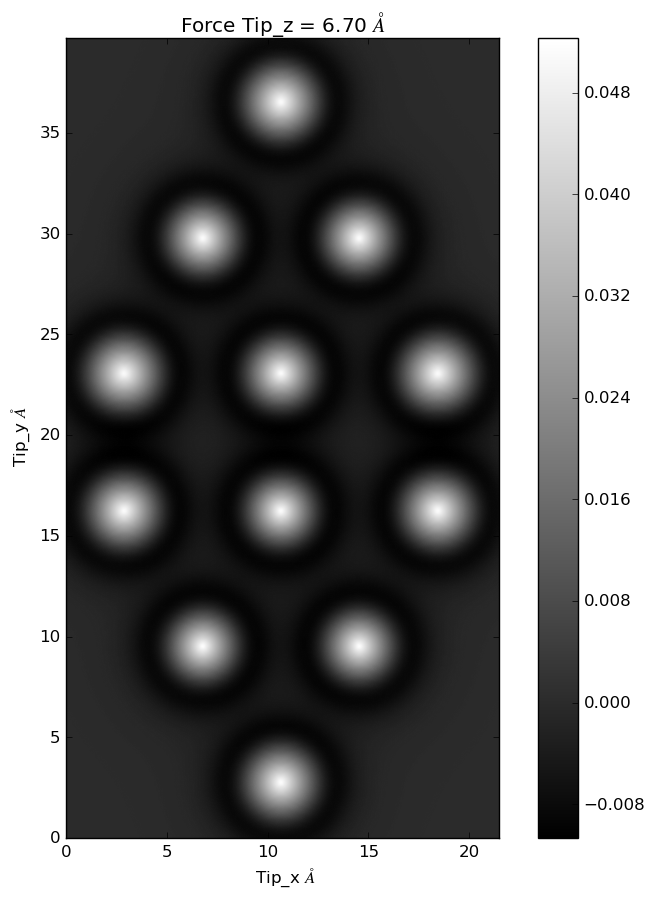

Supplement: File 3 — Datasets A0=1A adatoms_only k=0.5. [file Beilstein_J_Nanotechnol-07-937-s003.zip › S3/A0=1A/adatoms_only/k=0.5/results/Force_0066.png]

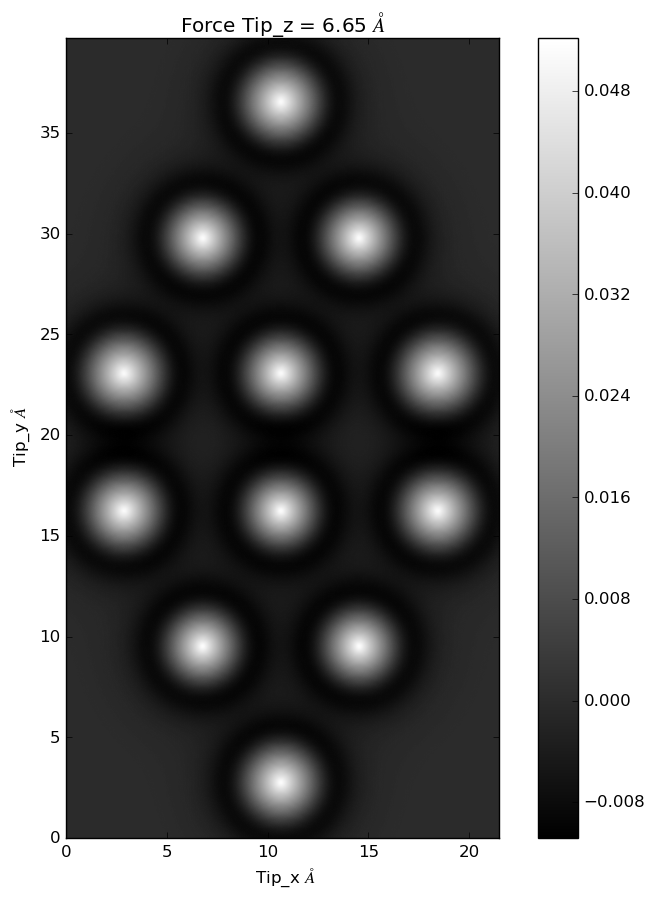

Supplement: File 3 — Datasets A0=1A adatoms_only k=0.5. [file Beilstein_J_Nanotechnol-07-937-s003.zip › S3/A0=1A/adatoms_only/k=0.5/results/Force_0067.png]

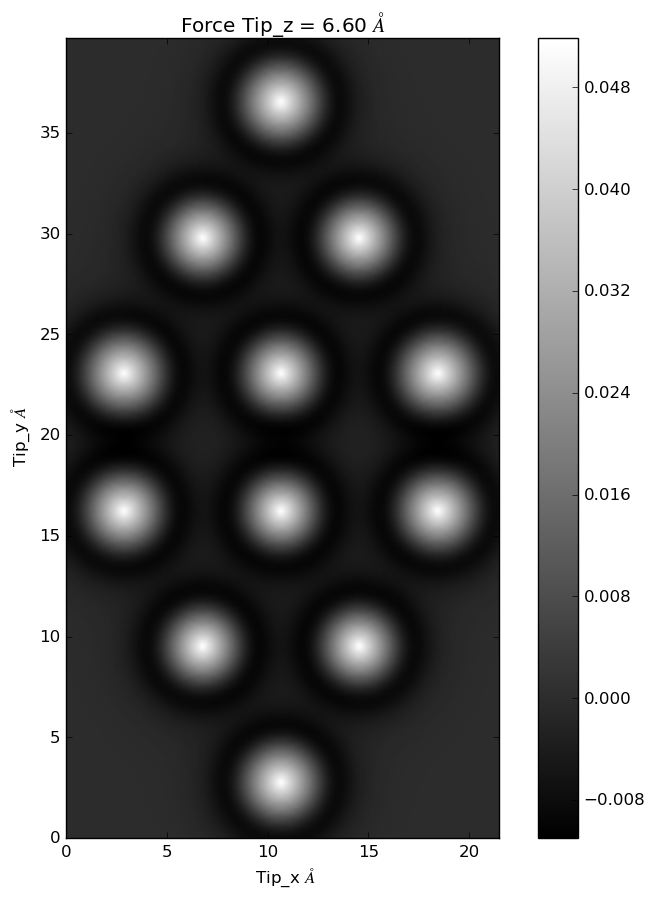

Supplement: File 3 — Datasets A0=1A adatoms_only k=0.5. [file Beilstein_J_Nanotechnol-07-937-s003.zip › S3/A0=1A/adatoms_only/k=0.5/results/Force_0068.png]

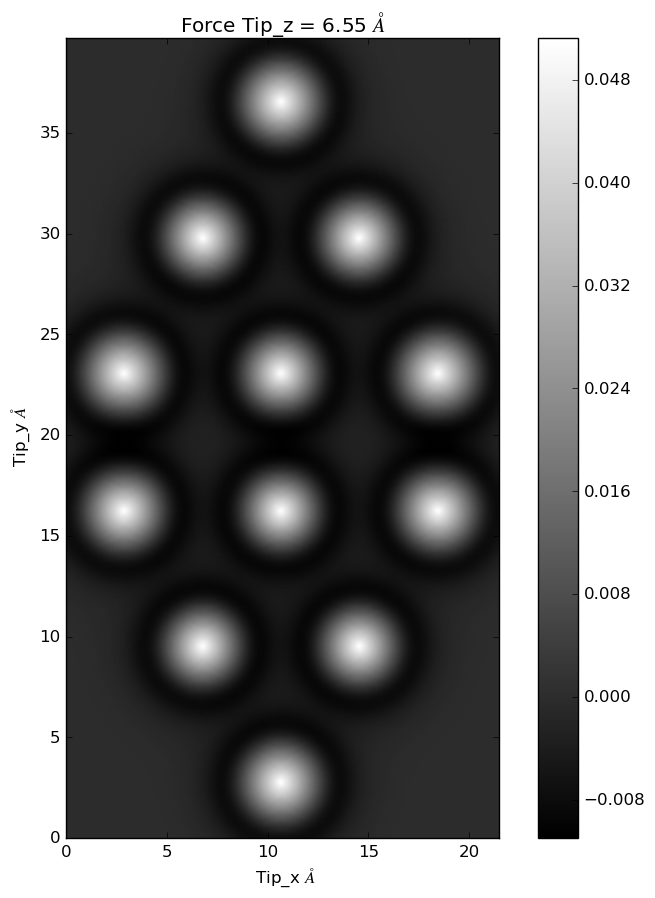

Supplement: File 3 — Datasets A0=1A adatoms_only k=0.5. [file Beilstein_J_Nanotechnol-07-937-s003.zip › S3/A0=1A/adatoms_only/k=0.5/results/Force_0069.png]

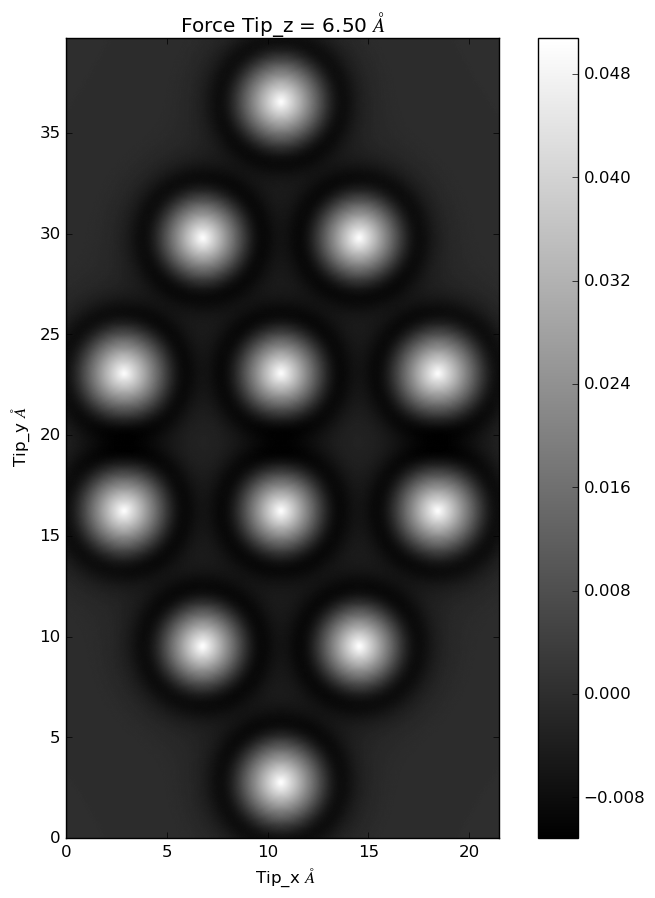

Supplement: File 3 — Datasets A0=1A adatoms_only k=0.5. [file Beilstein_J_Nanotechnol-07-937-s003.zip › S3/A0=1A/adatoms_only/k=0.5/results/Force_0070.png]

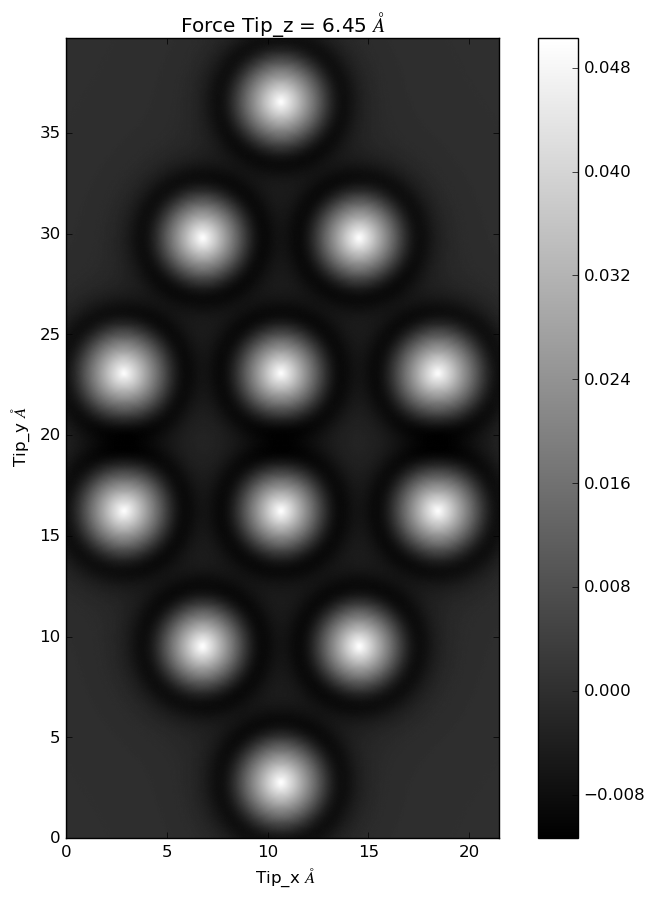

Supplement: File 3 — Datasets A0=1A adatoms_only k=0.5. [file Beilstein_J_Nanotechnol-07-937-s003.zip › S3/A0=1A/adatoms_only/k=0.5/results/Force_0071.png]

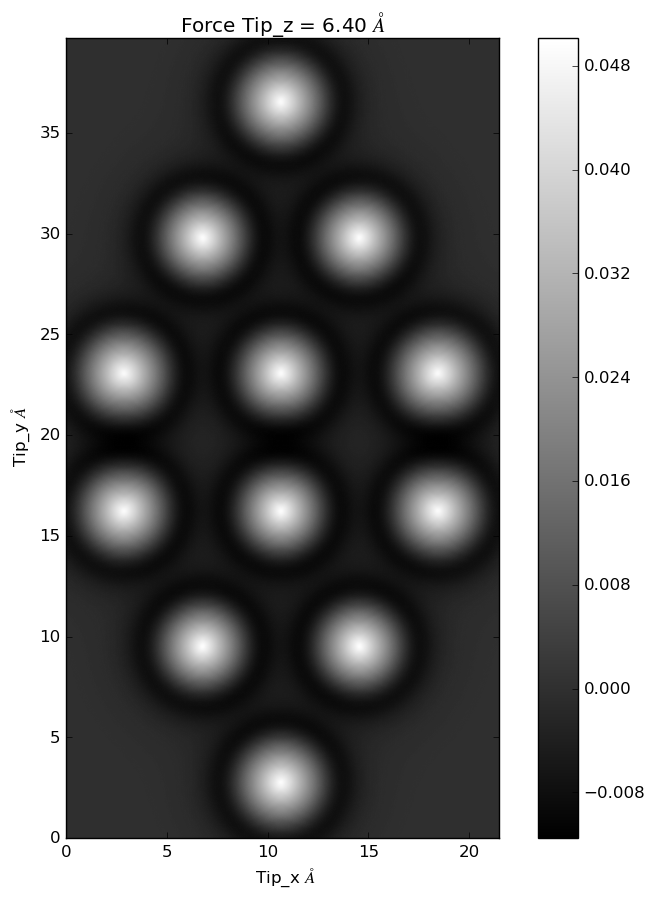

Supplement: File 3 — Datasets A0=1A adatoms_only k=0.5. [file Beilstein_J_Nanotechnol-07-937-s003.zip › S3/A0=1A/adatoms_only/k=0.5/results/Force_0072.png]

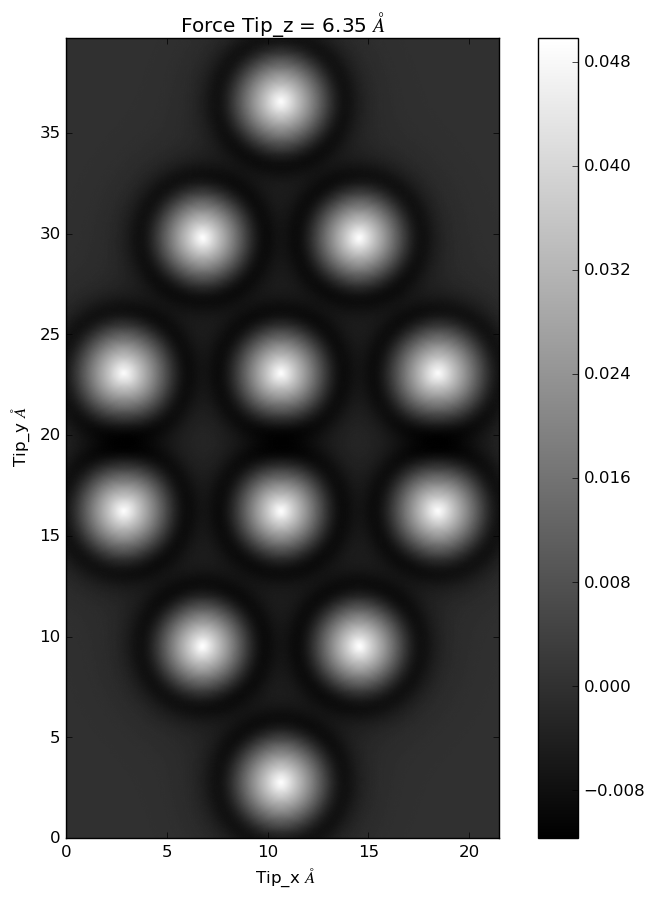

Supplement: File 3 — Datasets A0=1A adatoms_only k=0.5. [file Beilstein_J_Nanotechnol-07-937-s003.zip › S3/A0=1A/adatoms_only/k=0.5/results/Force_0073.png]

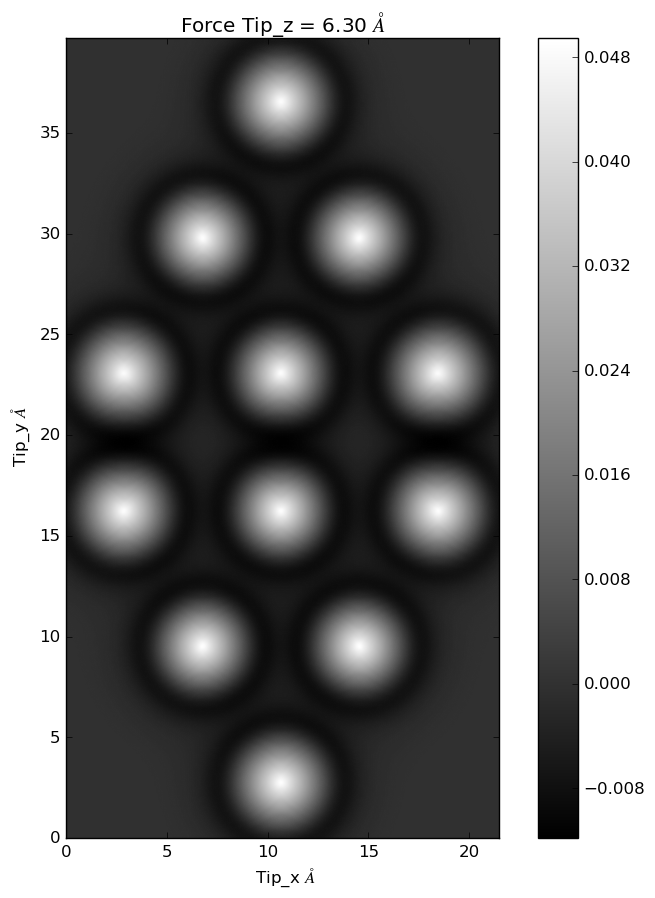

Supplement: File 3 — Datasets A0=1A adatoms_only k=0.5. [file Beilstein_J_Nanotechnol-07-937-s003.zip › S3/A0=1A/adatoms_only/k=0.5/results/Force_0074.png]

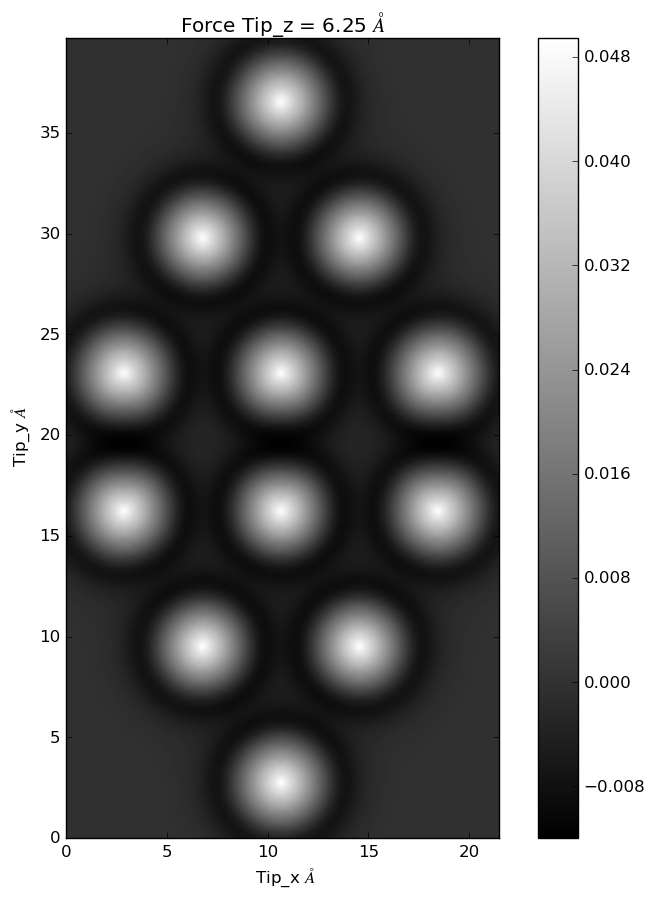

Supplement: File 3 — Datasets A0=1A adatoms_only k=0.5. [file Beilstein_J_Nanotechnol-07-937-s003.zip › S3/A0=1A/adatoms_only/k=0.5/results/Force_0075.png]

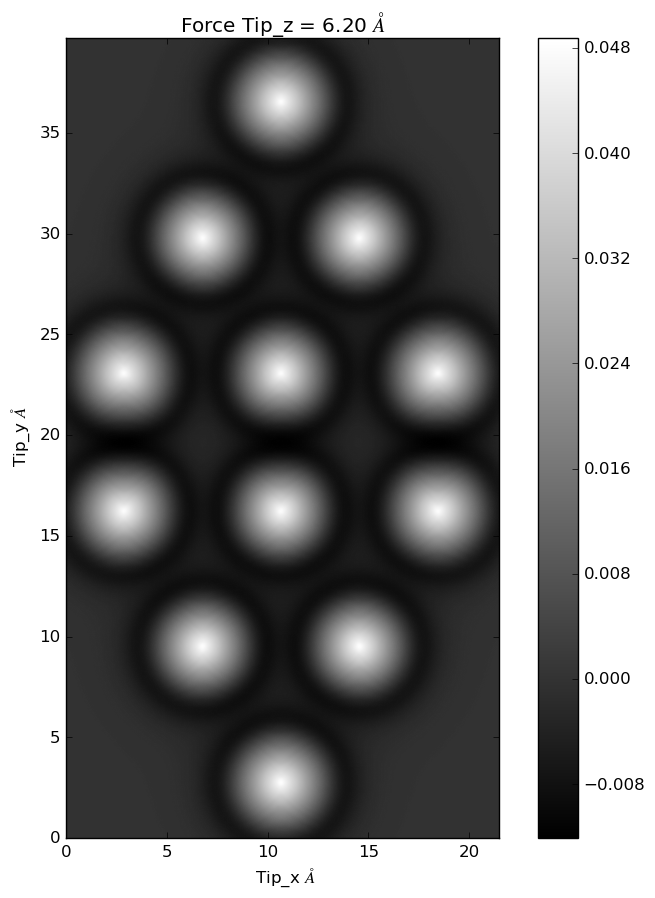

Supplement: File 3 — Datasets A0=1A adatoms_only k=0.5. [file Beilstein_J_Nanotechnol-07-937-s003.zip › S3/A0=1A/adatoms_only/k=0.5/results/Force_0076.png]

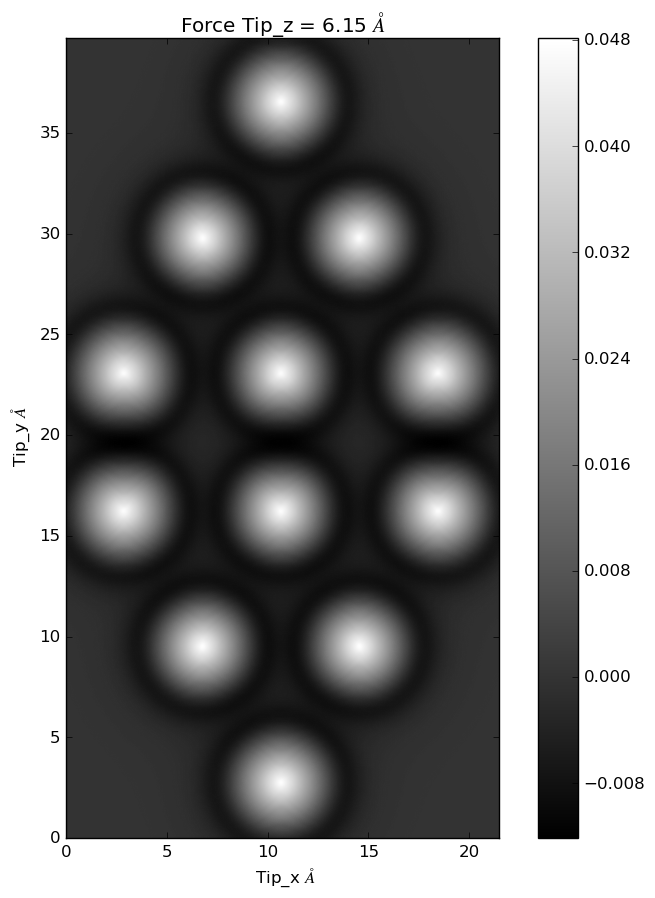

Supplement: File 3 — Datasets A0=1A adatoms_only k=0.5. [file Beilstein_J_Nanotechnol-07-937-s003.zip › S3/A0=1A/adatoms_only/k=0.5/results/Force_0077.png]

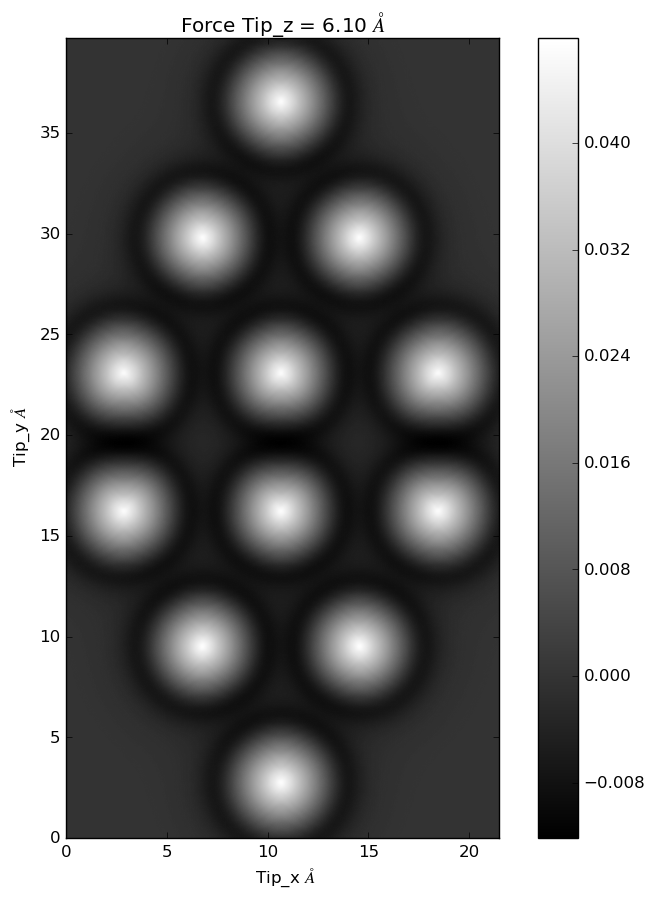

Supplement: File 3 — Datasets A0=1A adatoms_only k=0.5. [file Beilstein_J_Nanotechnol-07-937-s003.zip › S3/A0=1A/adatoms_only/k=0.5/results/Force_0078.png]

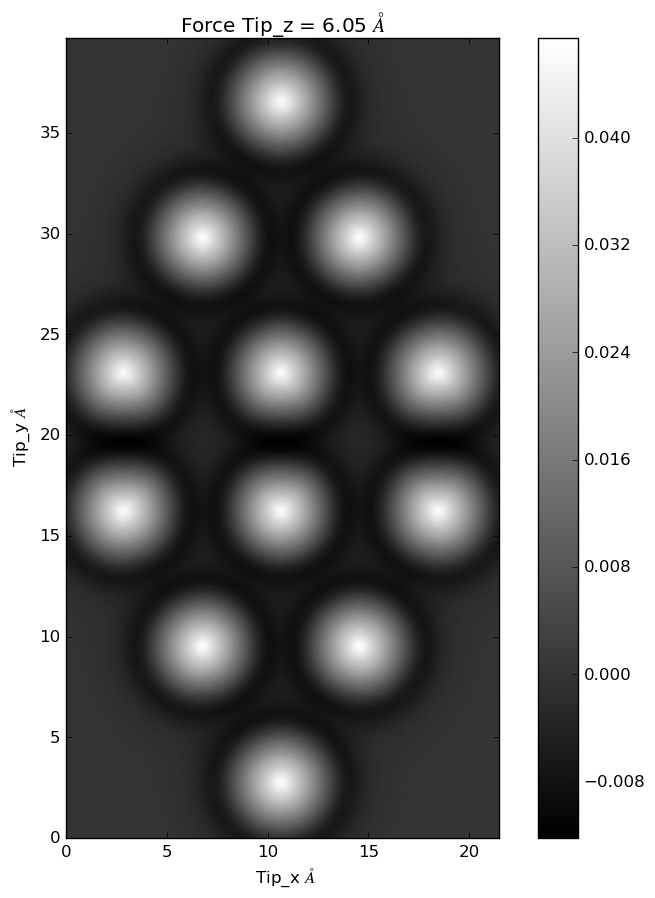

Supplement: File 3 — Datasets A0=1A adatoms_only k=0.5. [file Beilstein_J_Nanotechnol-07-937-s003.zip › S3/A0=1A/adatoms_only/k=0.5/results/Force_0079.png]

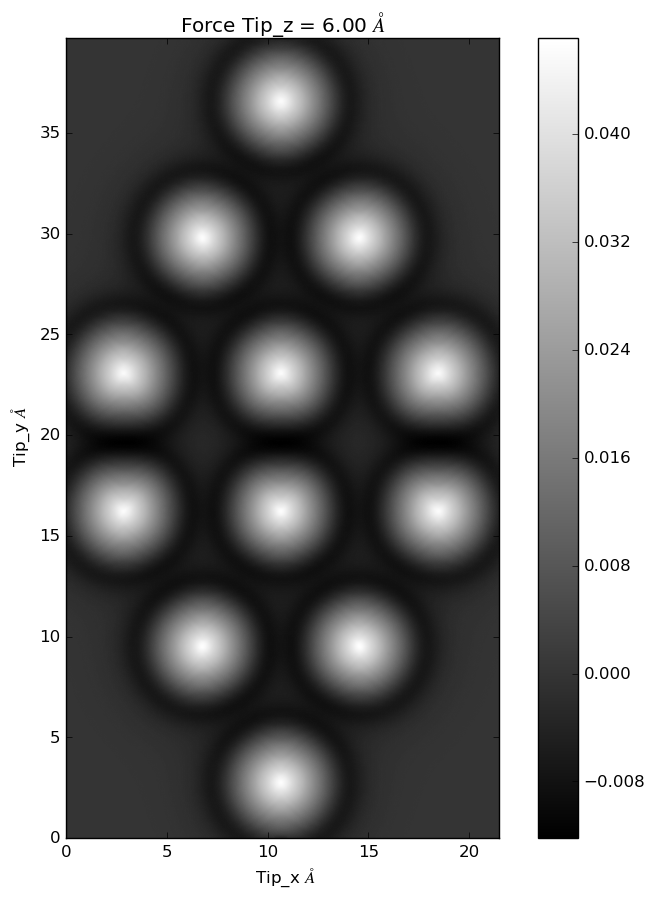

Supplement: File 3 — Datasets A0=1A adatoms_only k=0.5. [file Beilstein_J_Nanotechnol-07-937-s003.zip › S3/A0=1A/adatoms_only/k=0.5/results/Force_0080.png]

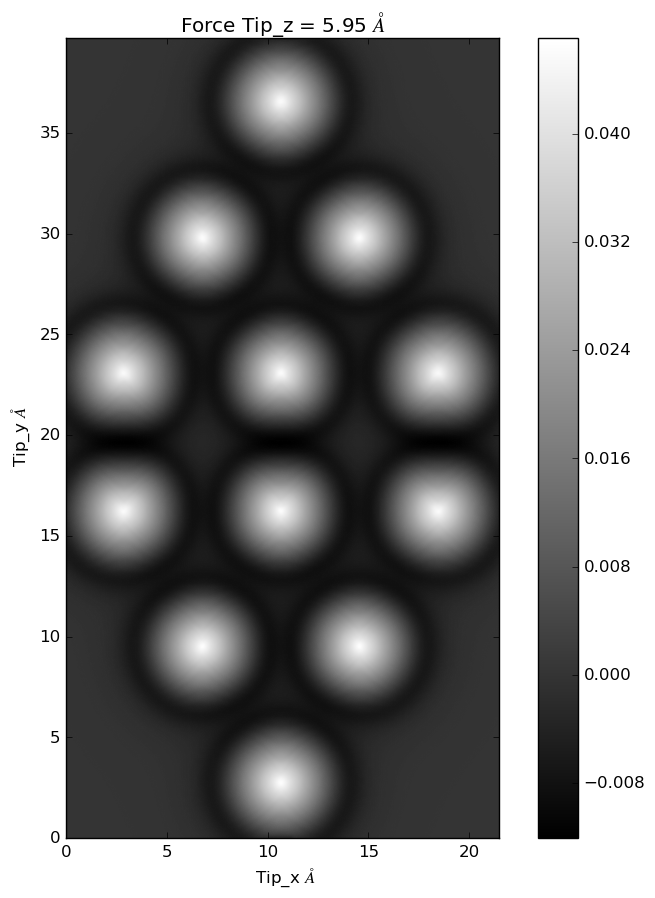

Supplement: File 3 — Datasets A0=1A adatoms_only k=0.5. [file Beilstein_J_Nanotechnol-07-937-s003.zip › S3/A0=1A/adatoms_only/k=0.5/results/Force_0081.png]

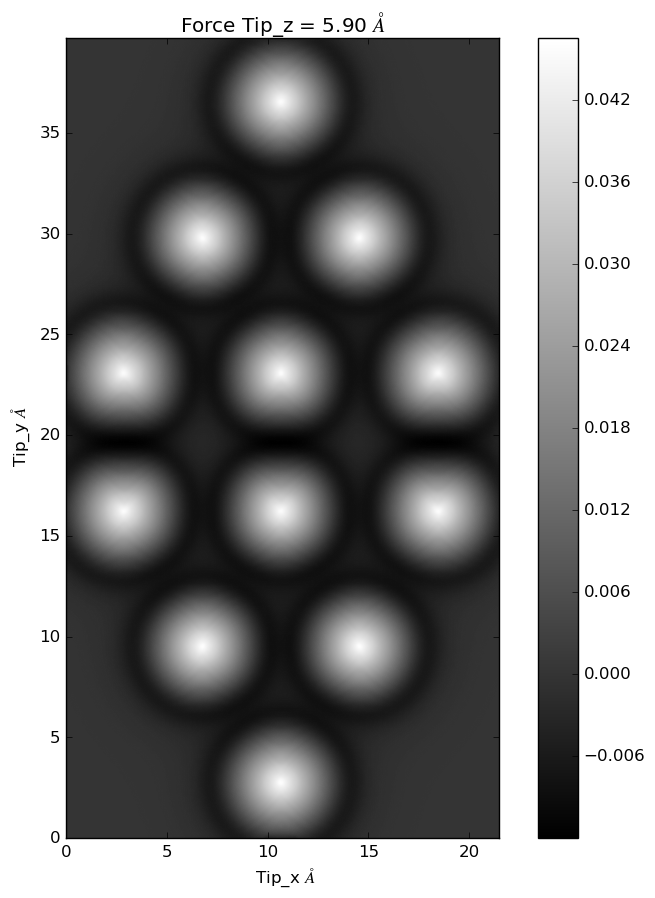

Supplement: File 3 — Datasets A0=1A adatoms_only k=0.5. [file Beilstein_J_Nanotechnol-07-937-s003.zip › S3/A0=1A/adatoms_only/k=0.5/results/Force_0082.png]

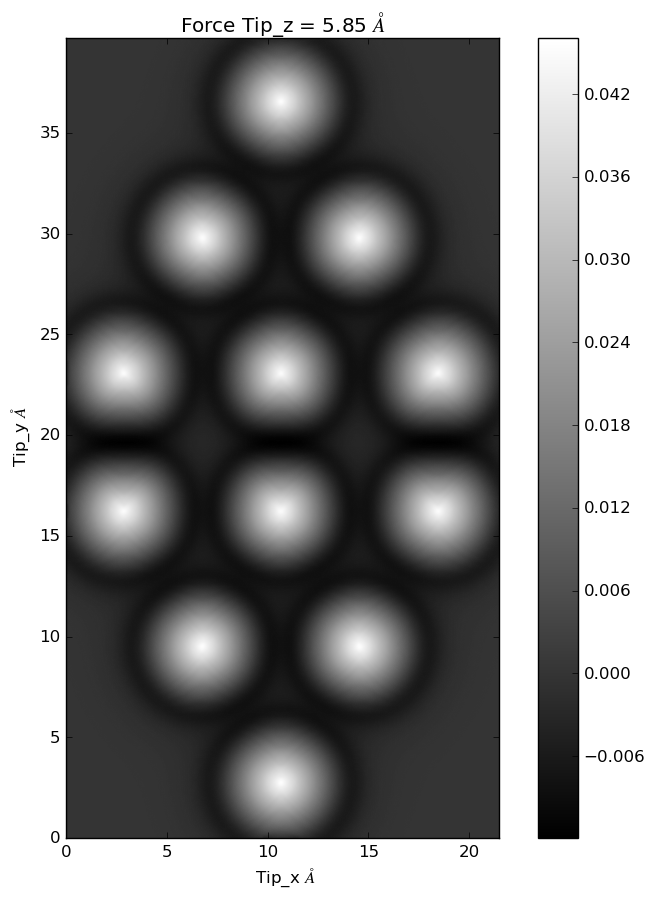

Supplement: File 3 — Datasets A0=1A adatoms_only k=0.5. [file Beilstein_J_Nanotechnol-07-937-s003.zip › S3/A0=1A/adatoms_only/k=0.5/results/Force_0083.png]

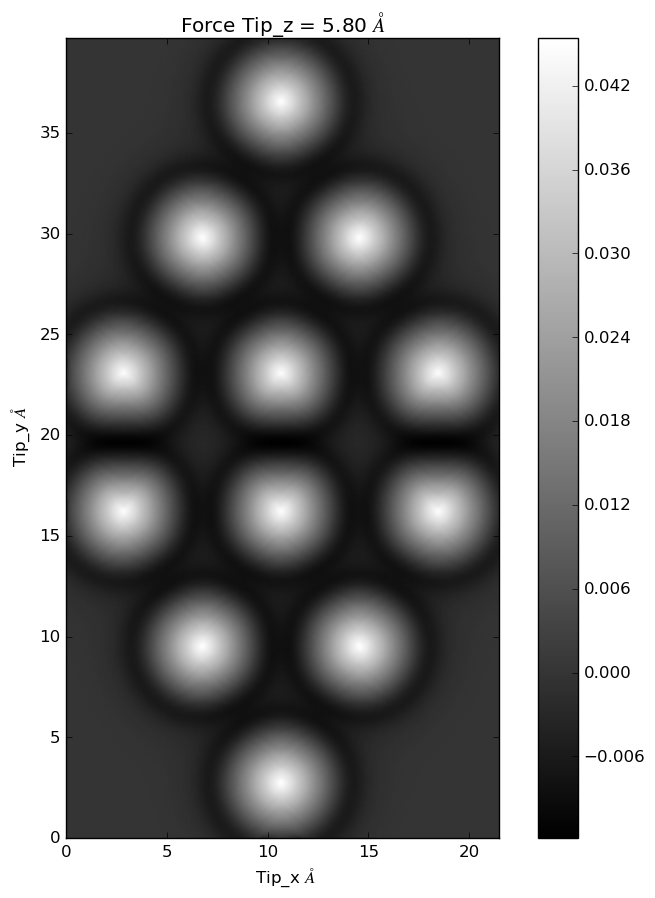

Supplement: File 3 — Datasets A0=1A adatoms_only k=0.5. [file Beilstein_J_Nanotechnol-07-937-s003.zip › S3/A0=1A/adatoms_only/k=0.5/results/Force_0084.png]

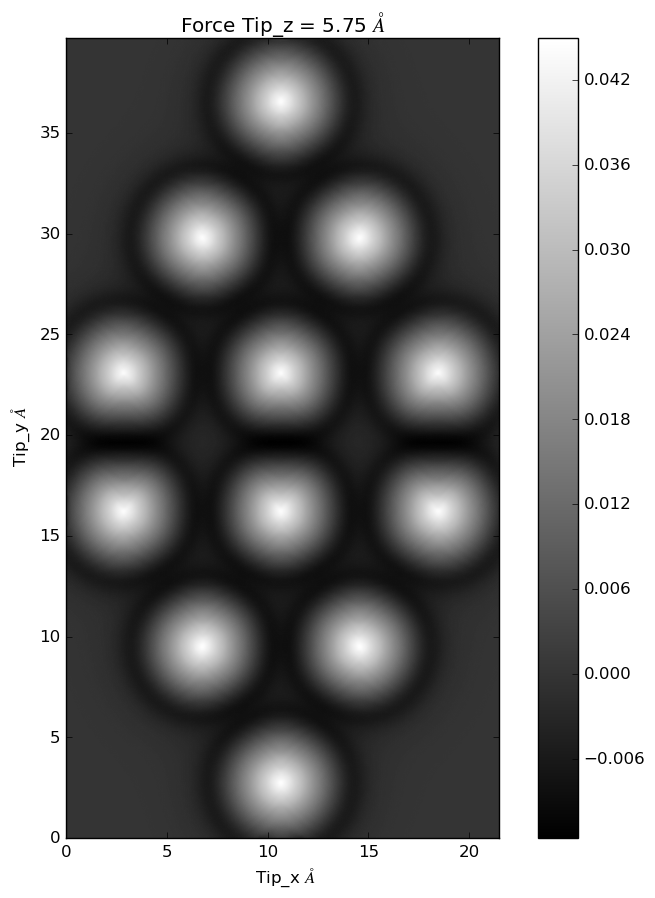

Supplement: File 3 — Datasets A0=1A adatoms_only k=0.5. [file Beilstein_J_Nanotechnol-07-937-s003.zip › S3/A0=1A/adatoms_only/k=0.5/results/Force_0085.png]

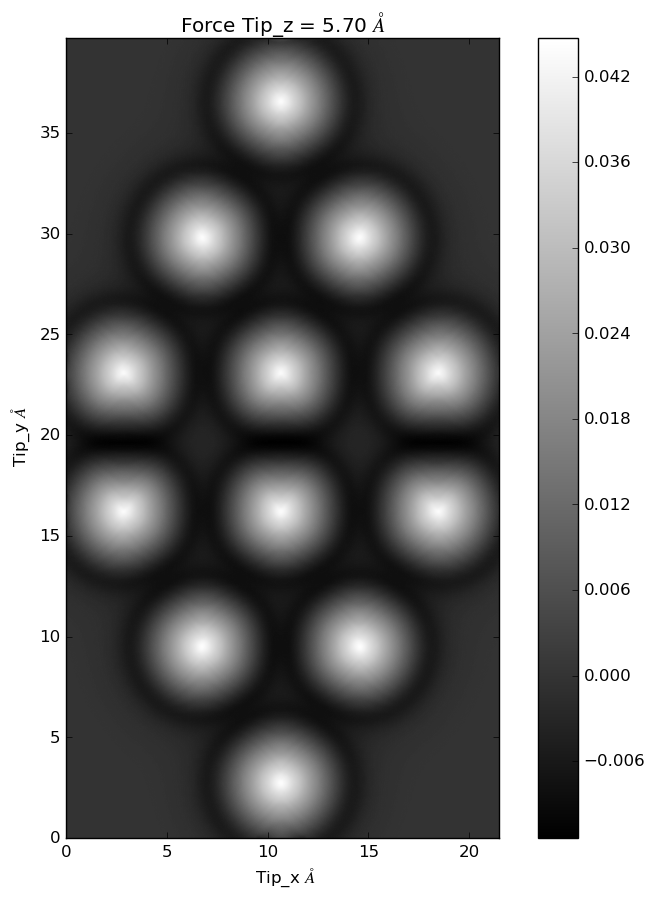

Supplement: File 3 — Datasets A0=1A adatoms_only k=0.5. [file Beilstein_J_Nanotechnol-07-937-s003.zip › S3/A0=1A/adatoms_only/k=0.5/results/Force_0086.png]

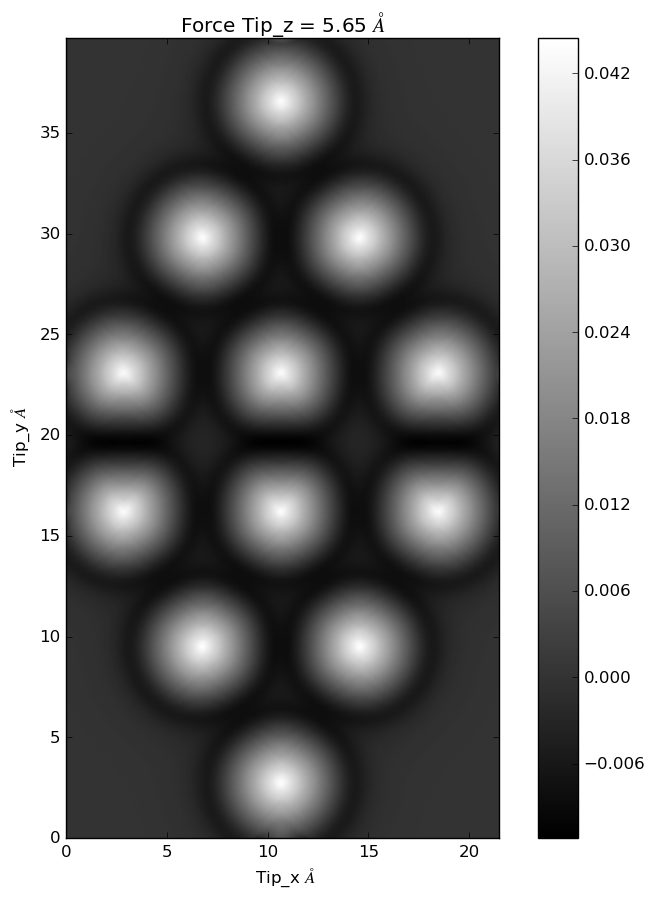

Supplement: File 3 — Datasets A0=1A adatoms_only k=0.5. [file Beilstein_J_Nanotechnol-07-937-s003.zip › S3/A0=1A/adatoms_only/k=0.5/results/Force_0087.png]

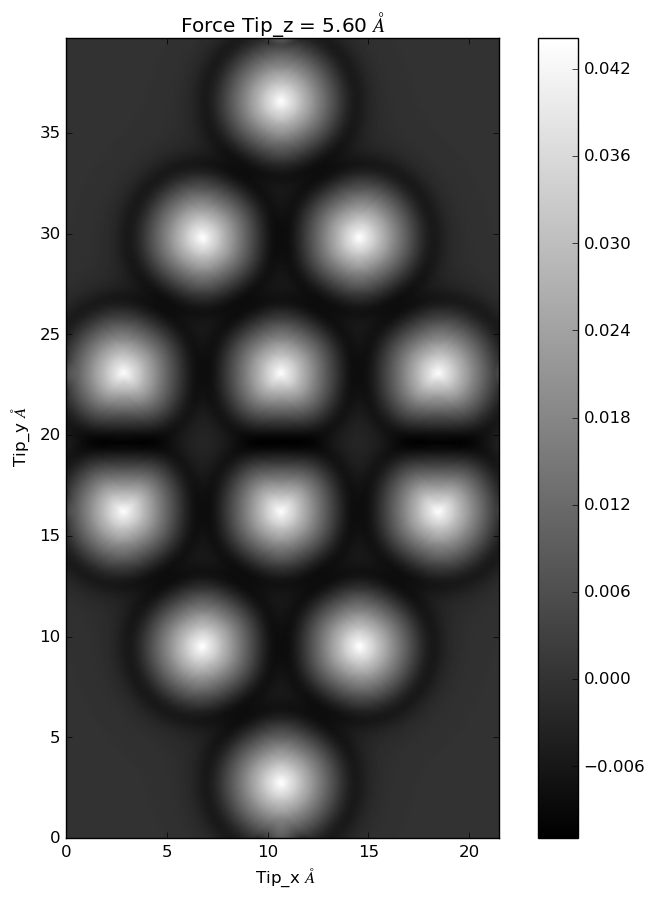

Supplement: File 3 — Datasets A0=1A adatoms_only k=0.5. [file Beilstein_J_Nanotechnol-07-937-s003.zip › S3/A0=1A/adatoms_only/k=0.5/results/Force_0088.png]

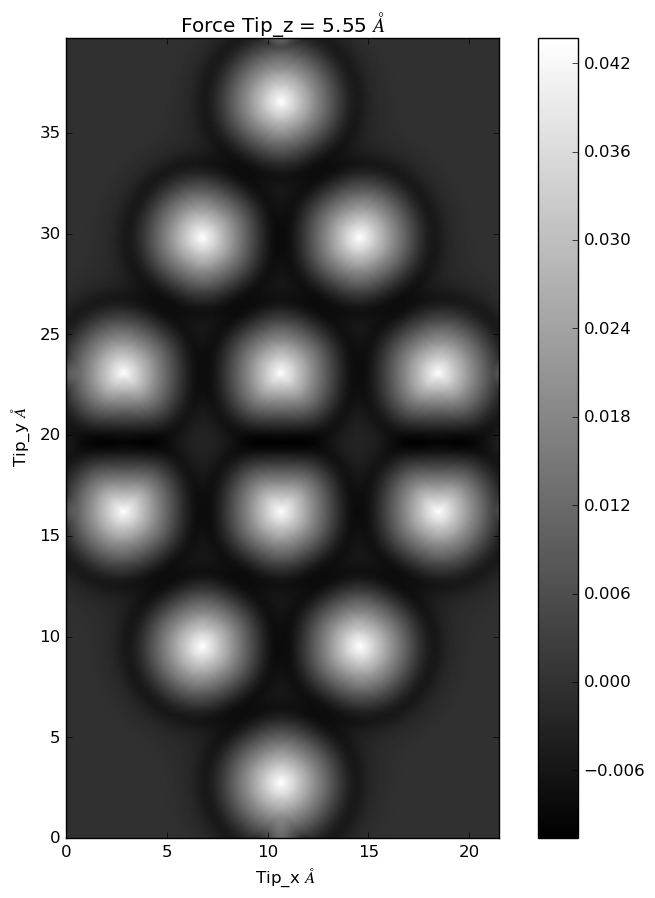

Supplement: File 3 — Datasets A0=1A adatoms_only k=0.5. [file Beilstein_J_Nanotechnol-07-937-s003.zip › S3/A0=1A/adatoms_only/k=0.5/results/Force_0089.png]

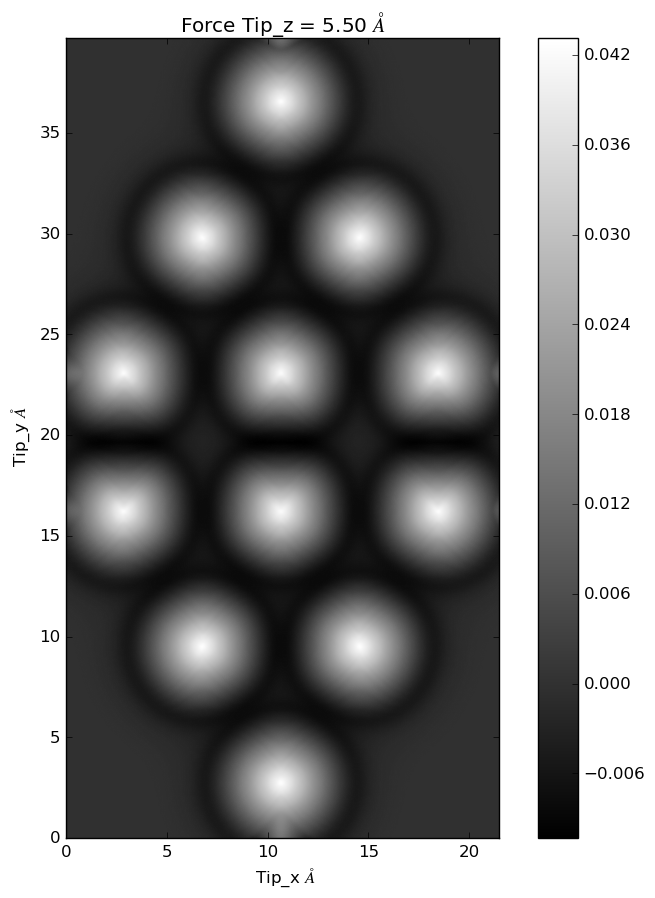

Supplement: File 3 — Datasets A0=1A adatoms_only k=0.5. [file Beilstein_J_Nanotechnol-07-937-s003.zip › S3/A0=1A/adatoms_only/k=0.5/results/Force_0090.png]

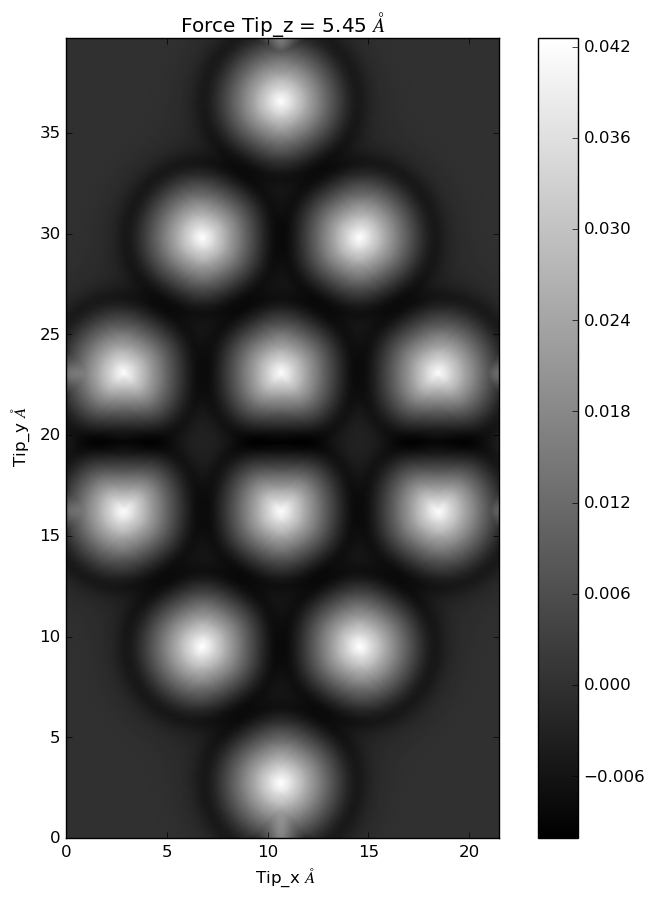

Supplement: File 3 — Datasets A0=1A adatoms_only k=0.5. [file Beilstein_J_Nanotechnol-07-937-s003.zip › S3/A0=1A/adatoms_only/k=0.5/results/Force_0091.png]

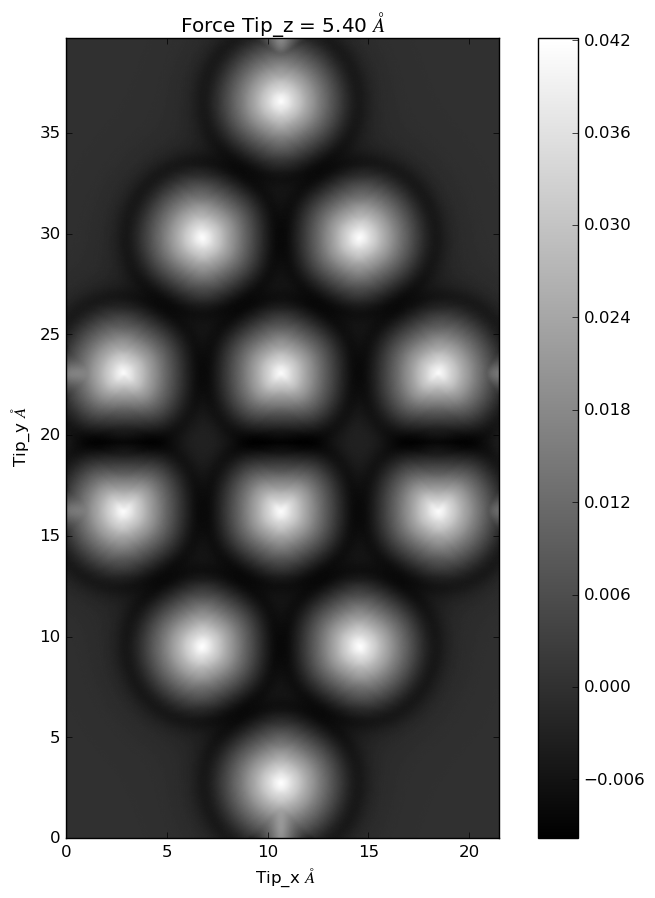

Supplement: File 3 — Datasets A0=1A adatoms_only k=0.5. [file Beilstein_J_Nanotechnol-07-937-s003.zip › S3/A0=1A/adatoms_only/k=0.5/results/Force_0092.png]

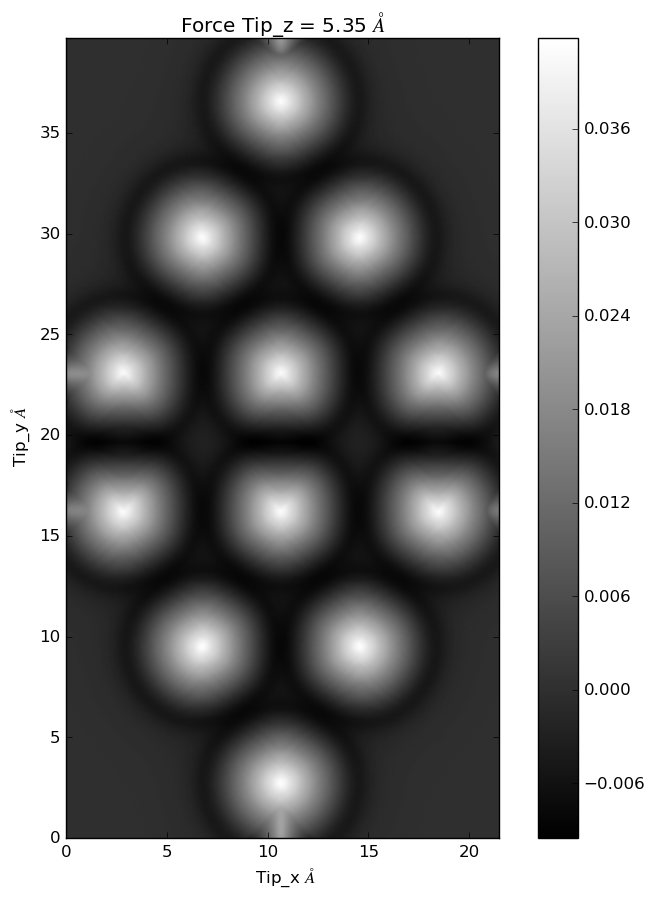

Supplement: File 3 — Datasets A0=1A adatoms_only k=0.5. [file Beilstein_J_Nanotechnol-07-937-s003.zip › S3/A0=1A/adatoms_only/k=0.5/results/Force_0093.png]

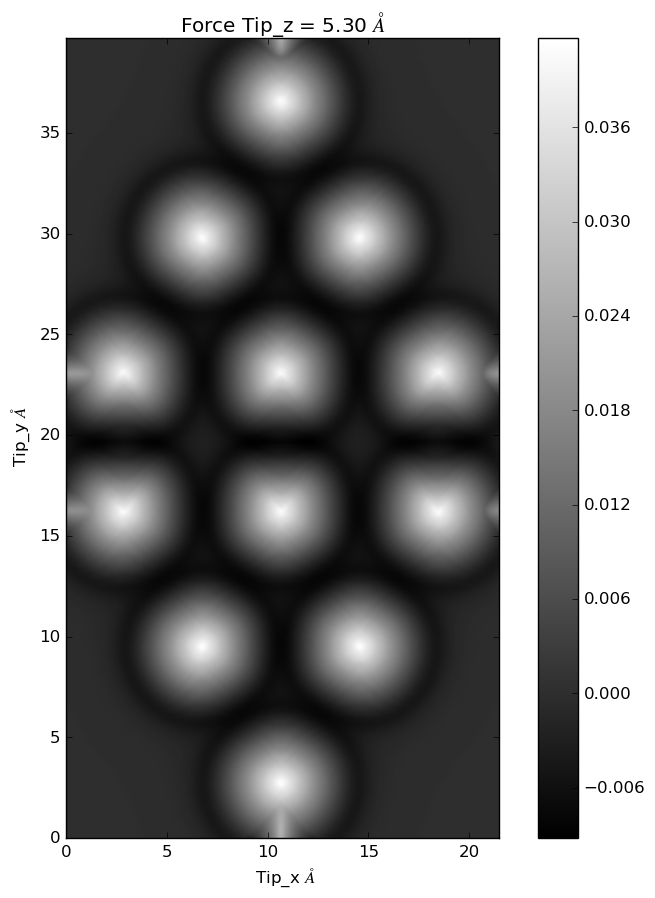

Supplement: File 3 — Datasets A0=1A adatoms_only k=0.5. [file Beilstein_J_Nanotechnol-07-937-s003.zip › S3/A0=1A/adatoms_only/k=0.5/results/Force_0094.png]

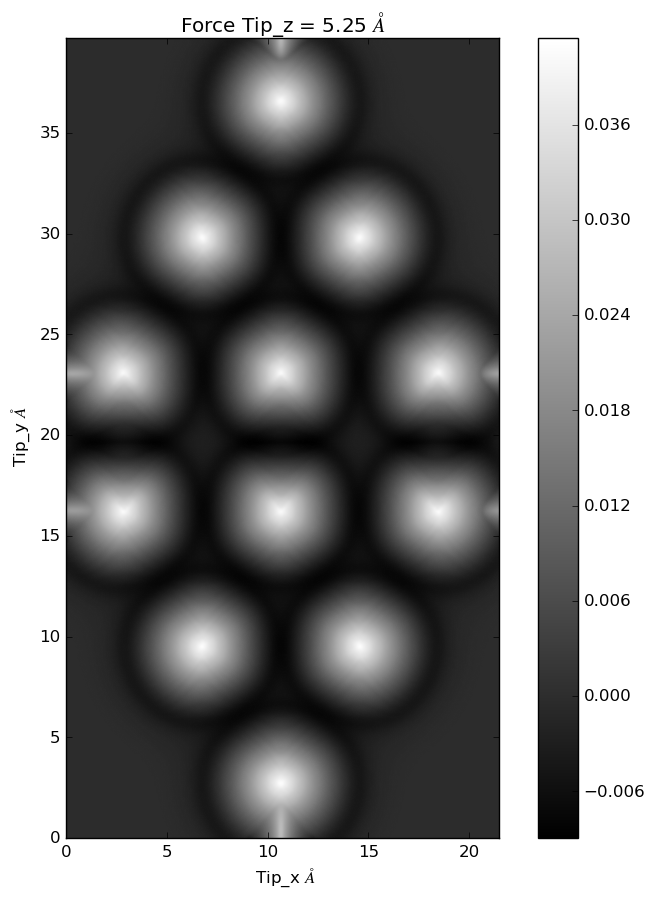

Supplement: File 3 — Datasets A0=1A adatoms_only k=0.5. [file Beilstein_J_Nanotechnol-07-937-s003.zip › S3/A0=1A/adatoms_only/k=0.5/results/Force_0095.png]

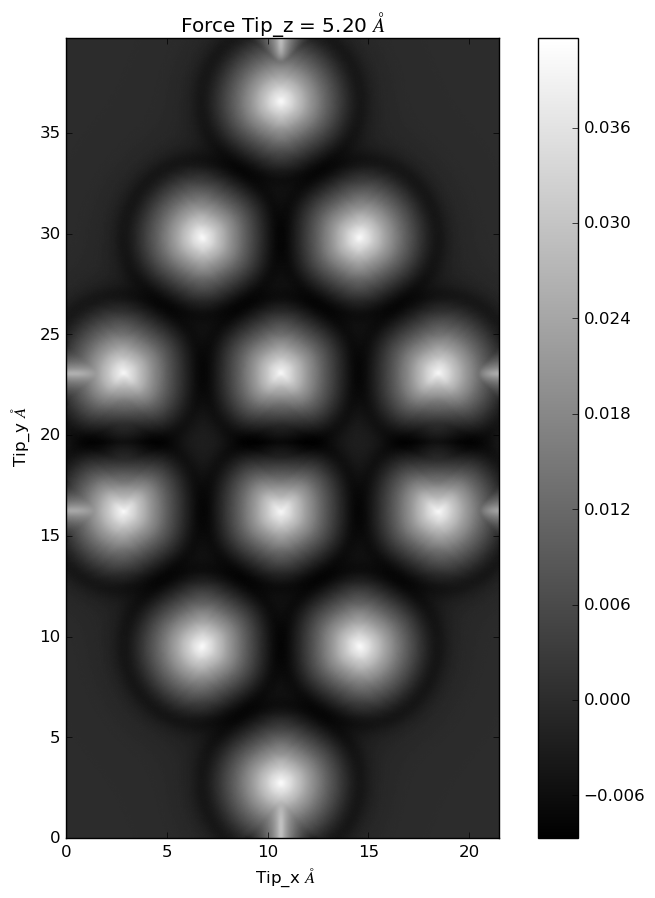

Supplement: File 3 — Datasets A0=1A adatoms_only k=0.5. [file Beilstein_J_Nanotechnol-07-937-s003.zip › S3/A0=1A/adatoms_only/k=0.5/results/Force_0096.png]

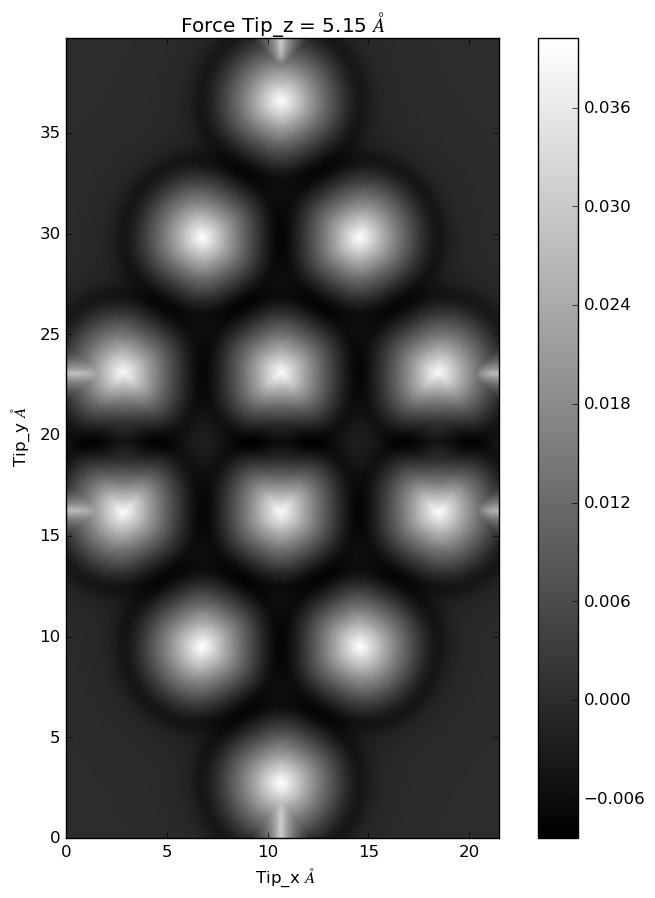

Supplement: File 3 — Datasets A0=1A adatoms_only k=0.5. [file Beilstein_J_Nanotechnol-07-937-s003.zip › S3/A0=1A/adatoms_only/k=0.5/results/Force_0097.png]
