# Supplementary figures and images for: Modelling of ‘sub-atomic’ contrast resulting from back-bonding on Si(111)-7×7 (part 14 of 18)
Source: Beilstein J Nanotechnol. 2016 Jun 29;7:937–45. doi: 10.3762/bjnano.7.85 (PMC4979881; doi:10.3762/bjnano.7.85)

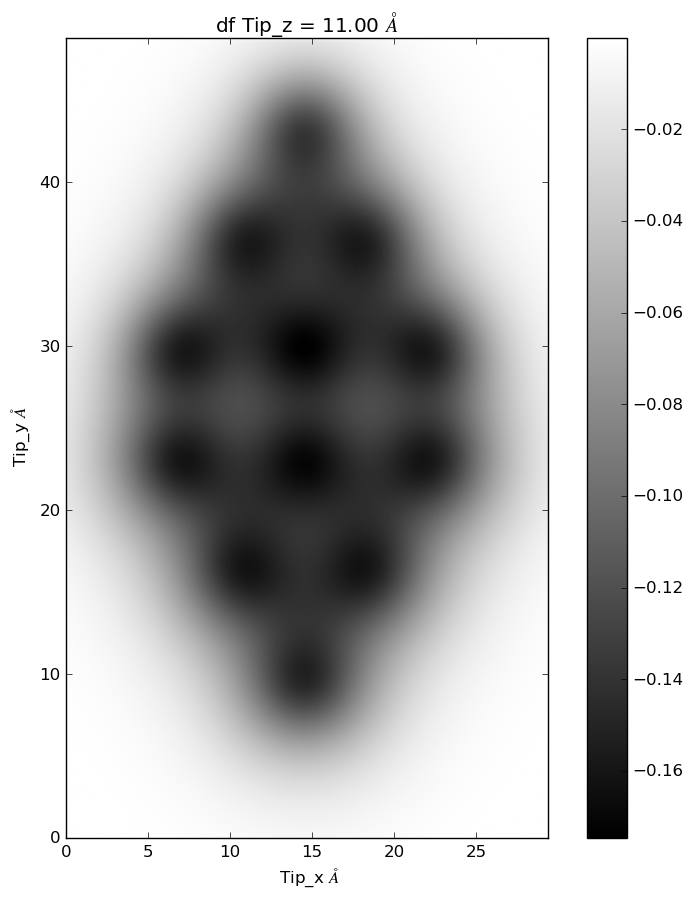

Supplement: File 7 — Datasets A0=1A k=0.5_extendedrange. [file Beilstein_J_Nanotechnol-07-937-s007.zip › S7/A0=1A/k=0.5_extendedrange/results/df_0100.png]

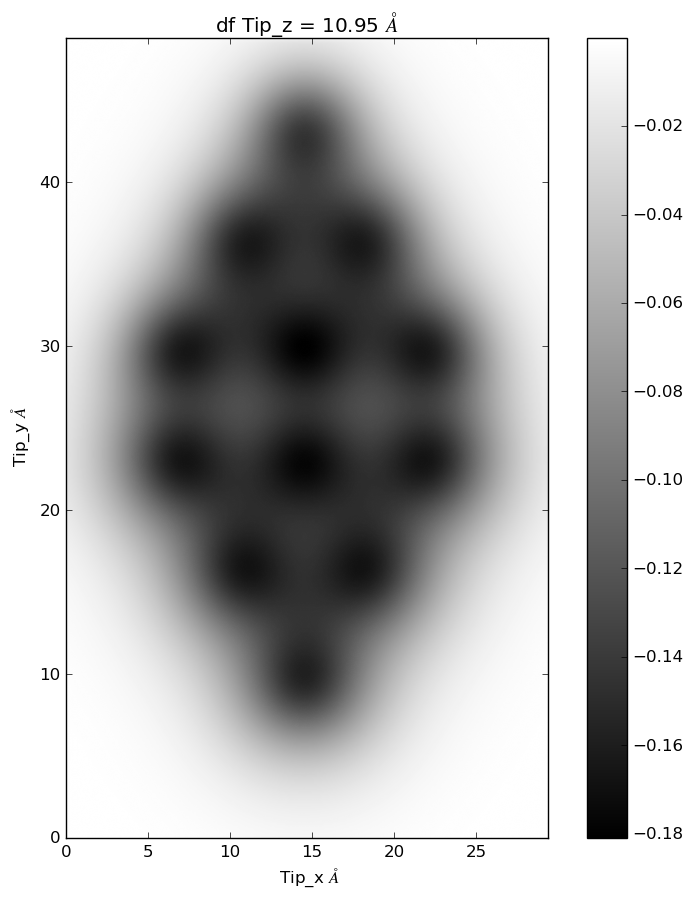

Supplement: File 7 — Datasets A0=1A k=0.5_extendedrange. [file Beilstein_J_Nanotechnol-07-937-s007.zip › S7/A0=1A/k=0.5_extendedrange/results/df_0101.png]

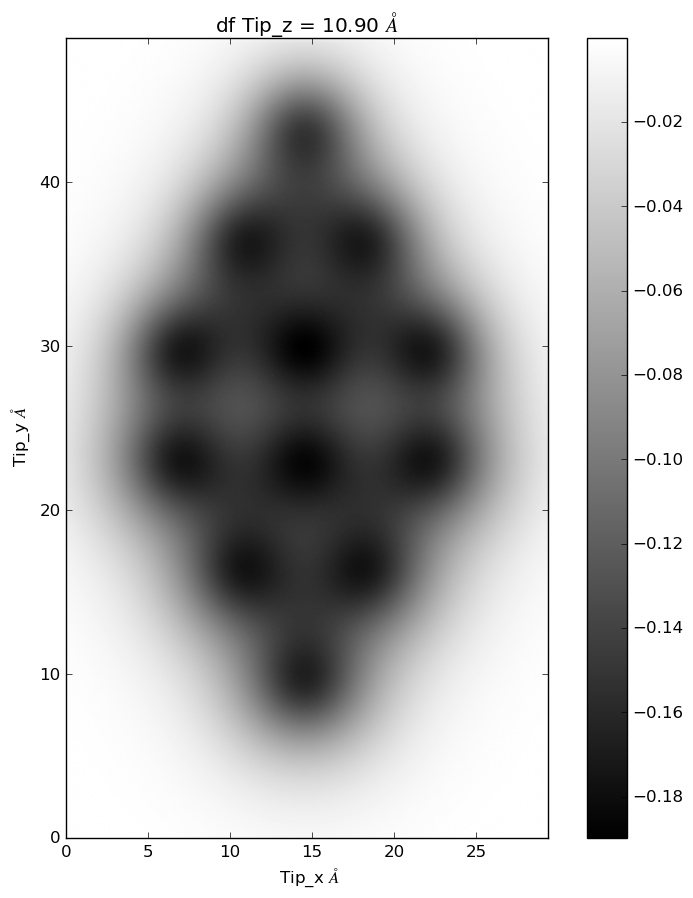

Supplement: File 7 — Datasets A0=1A k=0.5_extendedrange. [file Beilstein_J_Nanotechnol-07-937-s007.zip › S7/A0=1A/k=0.5_extendedrange/results/df_0102.png]

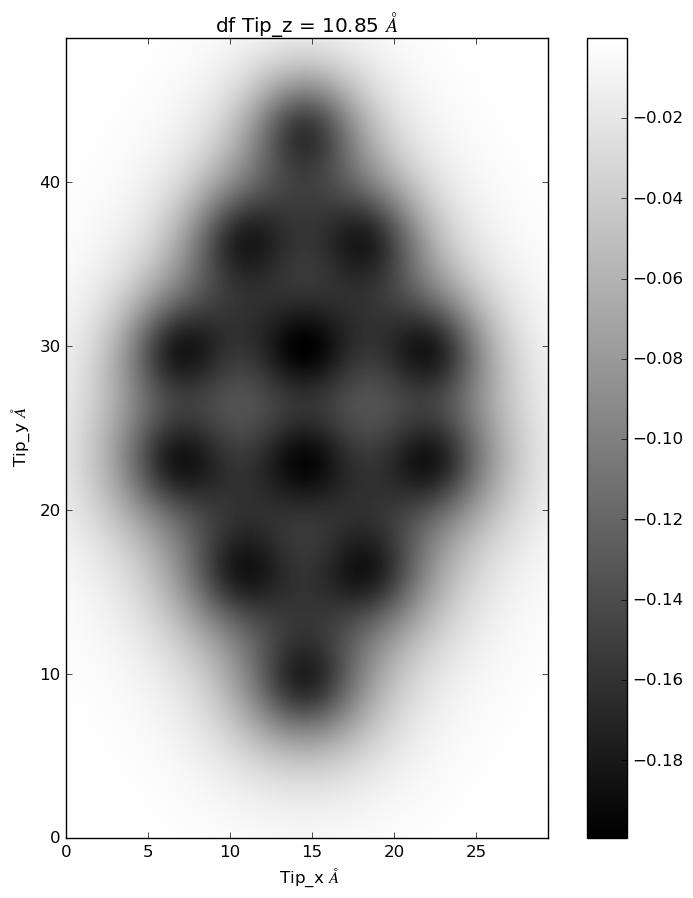

Supplement: File 7 — Datasets A0=1A k=0.5_extendedrange. [file Beilstein_J_Nanotechnol-07-937-s007.zip › S7/A0=1A/k=0.5_extendedrange/results/df_0103.png]

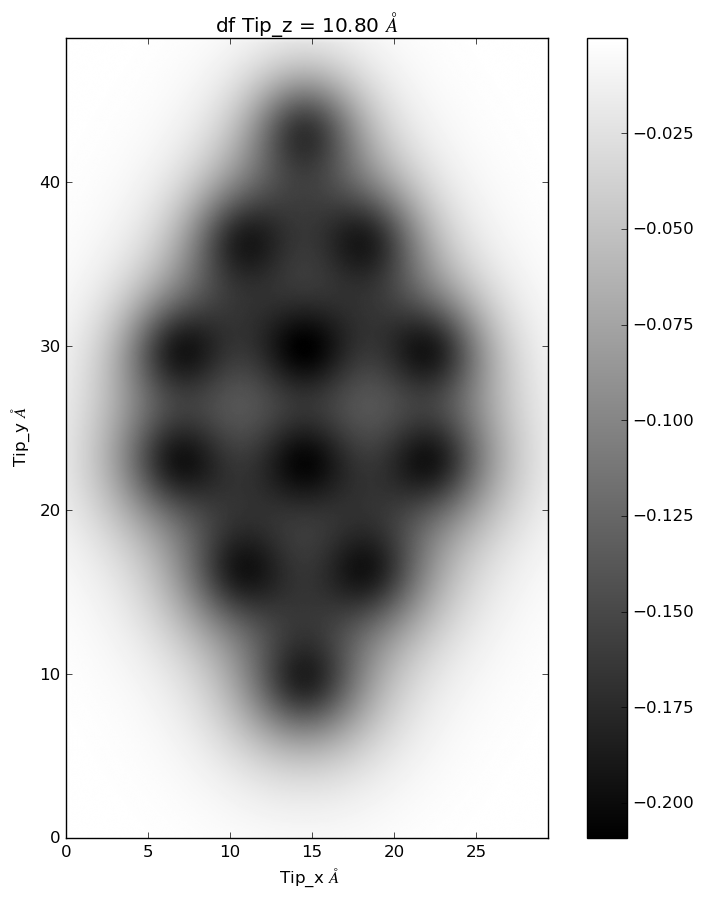

Supplement: File 7 — Datasets A0=1A k=0.5_extendedrange. [file Beilstein_J_Nanotechnol-07-937-s007.zip › S7/A0=1A/k=0.5_extendedrange/results/df_0104.png]

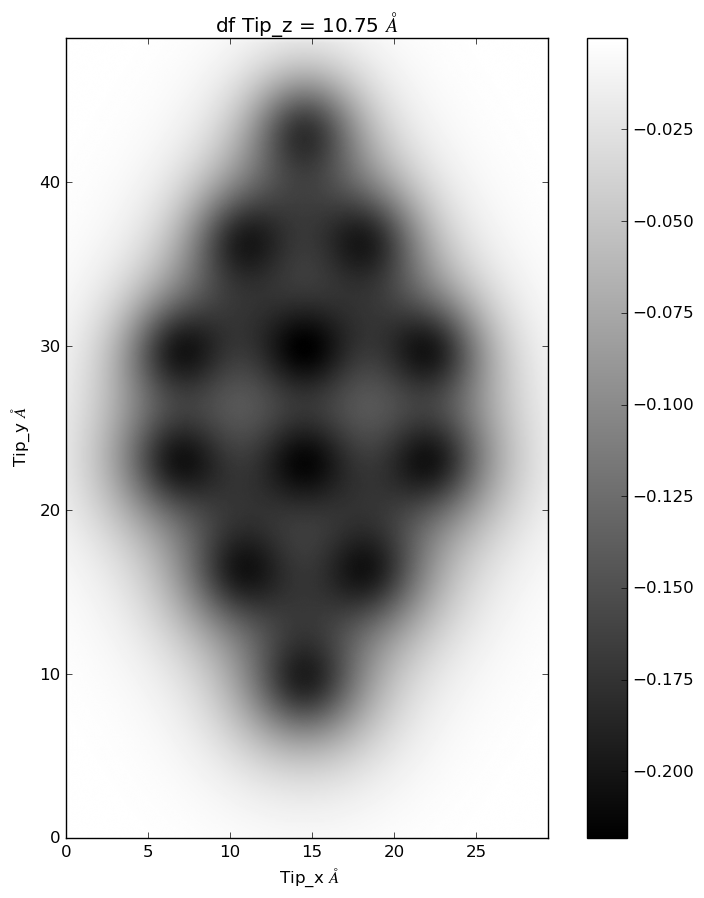

Supplement: File 7 — Datasets A0=1A k=0.5_extendedrange. [file Beilstein_J_Nanotechnol-07-937-s007.zip › S7/A0=1A/k=0.5_extendedrange/results/df_0105.png]

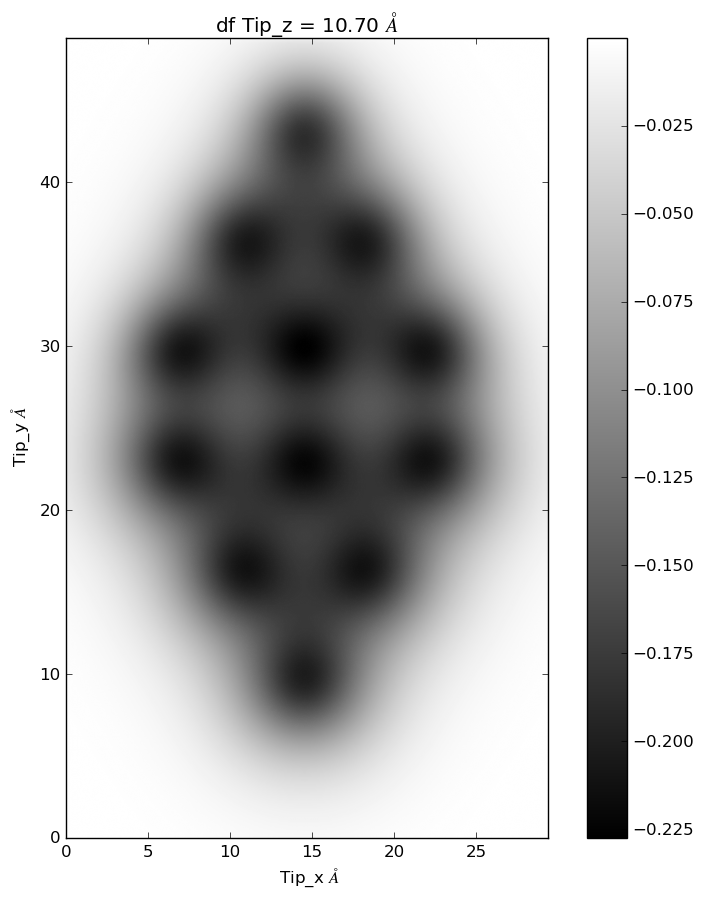

Supplement: File 7 — Datasets A0=1A k=0.5_extendedrange. [file Beilstein_J_Nanotechnol-07-937-s007.zip › S7/A0=1A/k=0.5_extendedrange/results/df_0106.png]

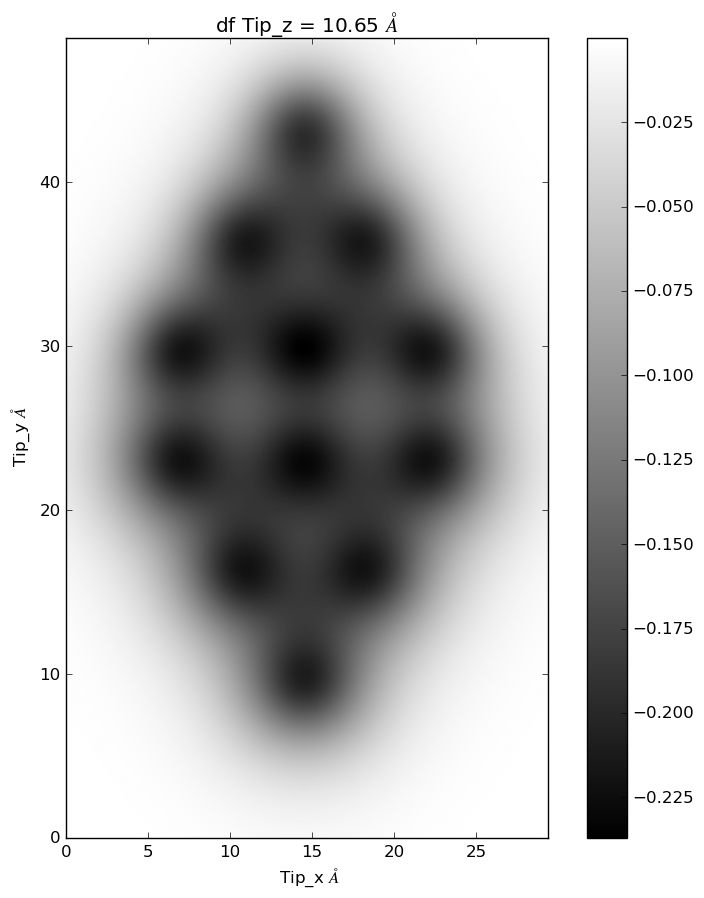

Supplement: File 7 — Datasets A0=1A k=0.5_extendedrange. [file Beilstein_J_Nanotechnol-07-937-s007.zip › S7/A0=1A/k=0.5_extendedrange/results/df_0107.png]

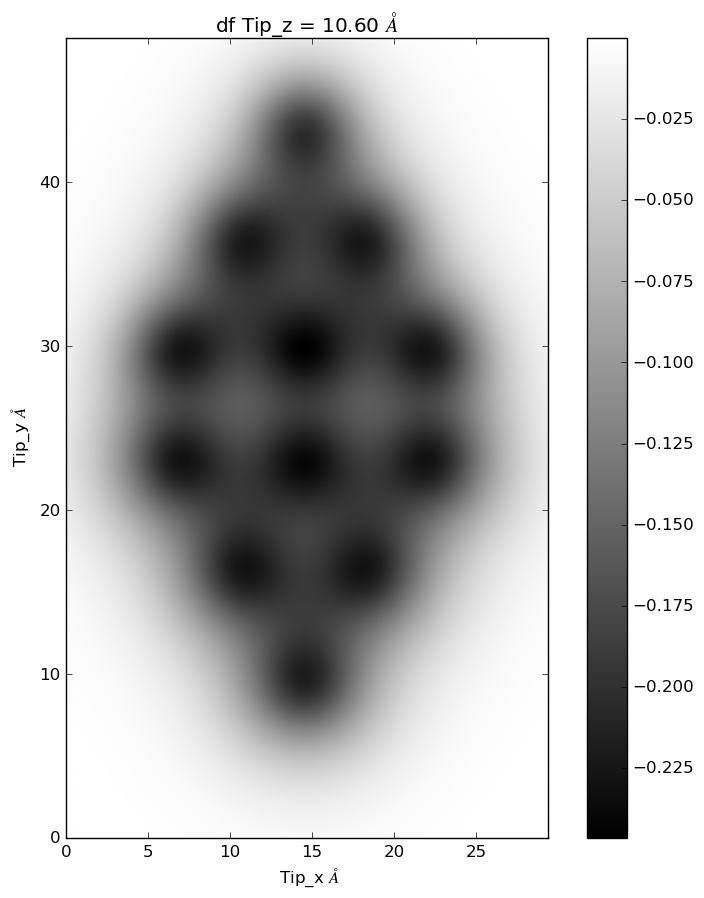

Supplement: File 7 — Datasets A0=1A k=0.5_extendedrange. [file Beilstein_J_Nanotechnol-07-937-s007.zip › S7/A0=1A/k=0.5_extendedrange/results/df_0108.png]

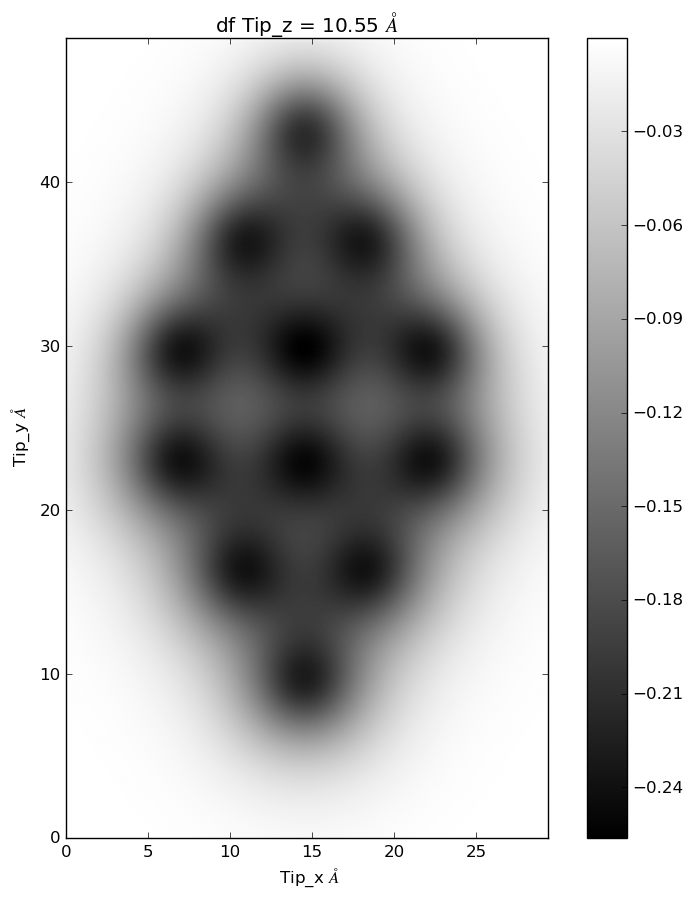

Supplement: File 7 — Datasets A0=1A k=0.5_extendedrange. [file Beilstein_J_Nanotechnol-07-937-s007.zip › S7/A0=1A/k=0.5_extendedrange/results/df_0109.png]

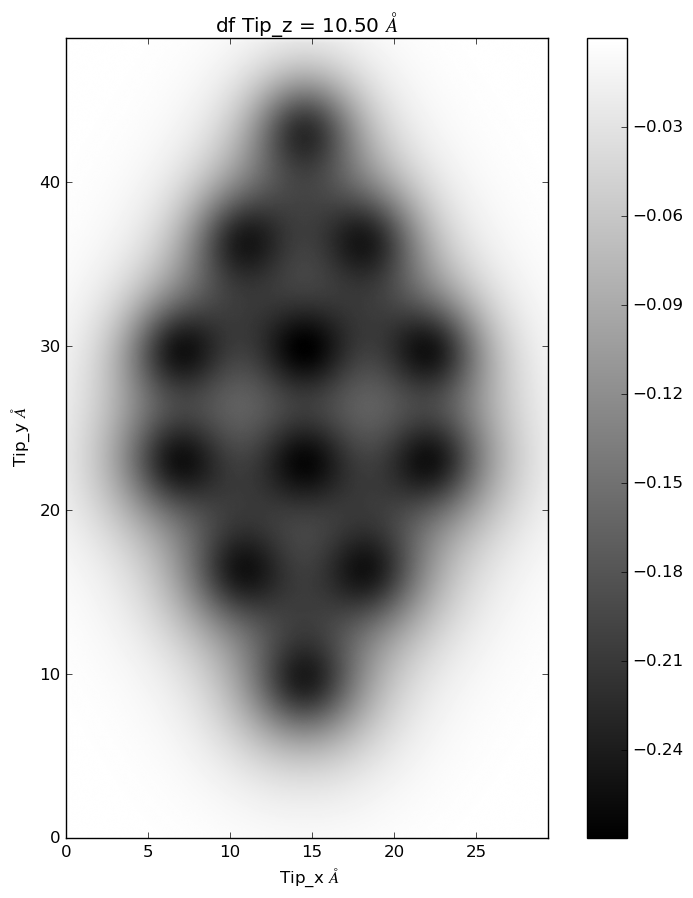

Supplement: File 7 — Datasets A0=1A k=0.5_extendedrange. [file Beilstein_J_Nanotechnol-07-937-s007.zip › S7/A0=1A/k=0.5_extendedrange/results/df_0110.png]

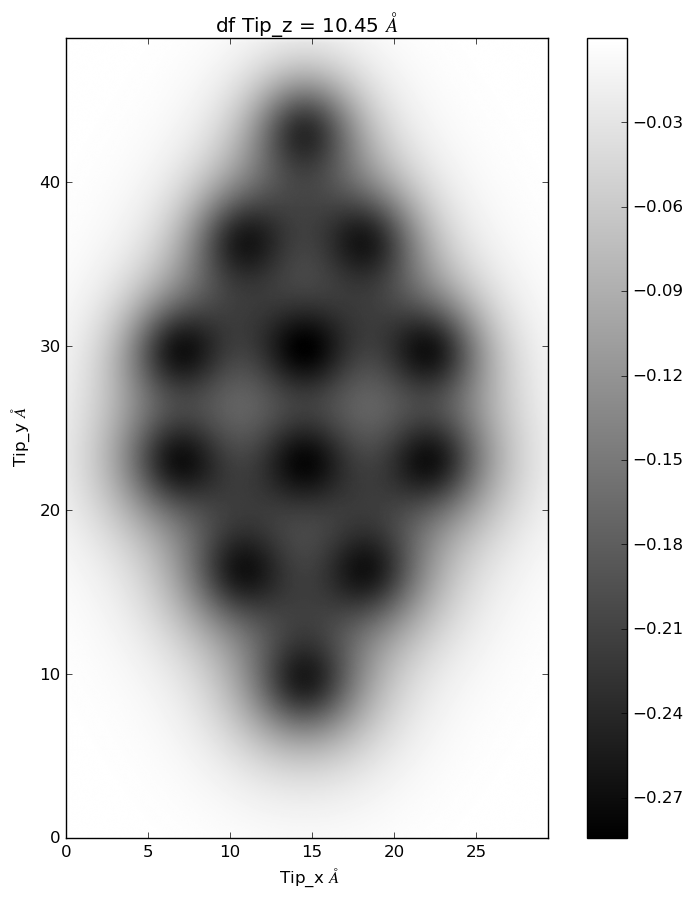

Supplement: File 7 — Datasets A0=1A k=0.5_extendedrange. [file Beilstein_J_Nanotechnol-07-937-s007.zip › S7/A0=1A/k=0.5_extendedrange/results/df_0111.png]

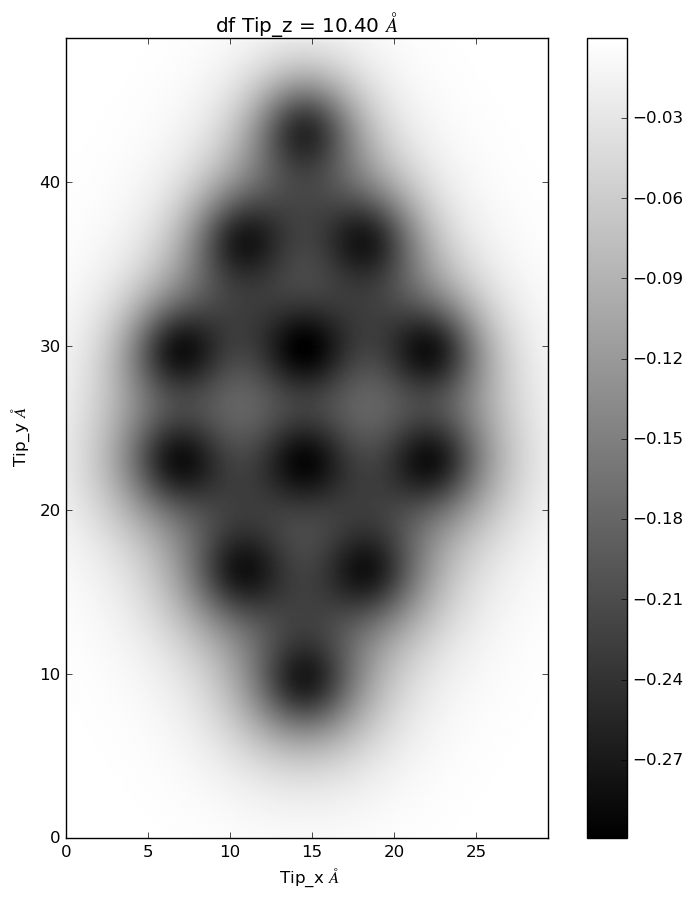

Supplement: File 7 — Datasets A0=1A k=0.5_extendedrange. [file Beilstein_J_Nanotechnol-07-937-s007.zip › S7/A0=1A/k=0.5_extendedrange/results/df_0112.png]

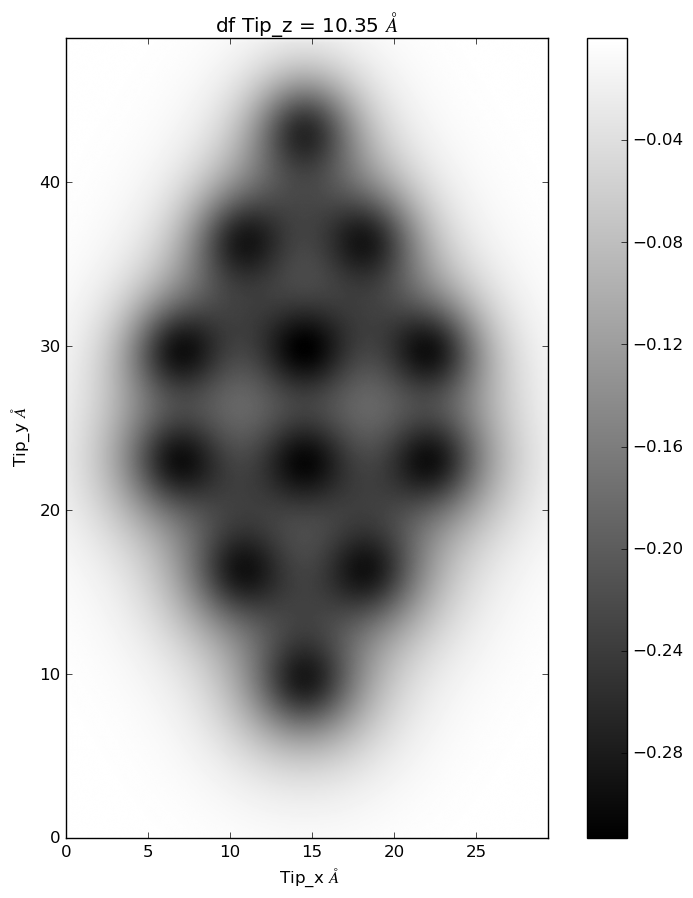

Supplement: File 7 — Datasets A0=1A k=0.5_extendedrange. [file Beilstein_J_Nanotechnol-07-937-s007.zip › S7/A0=1A/k=0.5_extendedrange/results/df_0113.png]

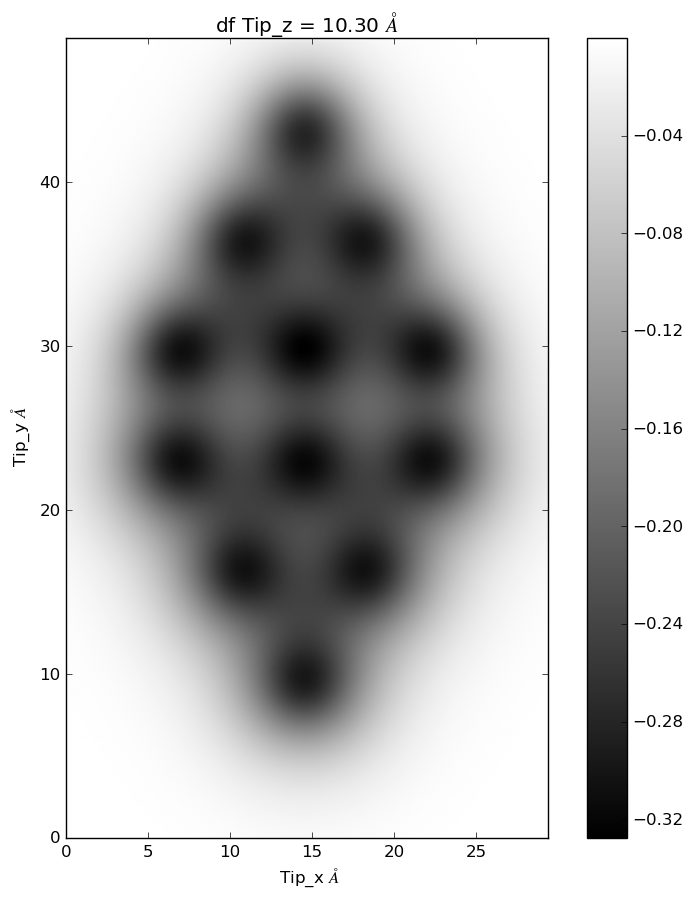

Supplement: File 7 — Datasets A0=1A k=0.5_extendedrange. [file Beilstein_J_Nanotechnol-07-937-s007.zip › S7/A0=1A/k=0.5_extendedrange/results/df_0114.png]

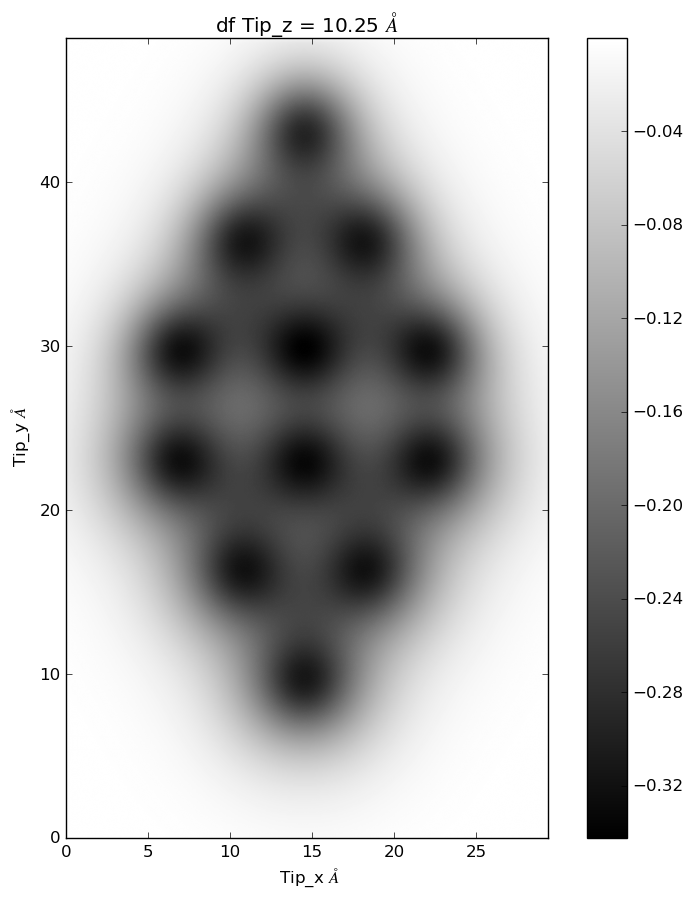

Supplement: File 7 — Datasets A0=1A k=0.5_extendedrange. [file Beilstein_J_Nanotechnol-07-937-s007.zip › S7/A0=1A/k=0.5_extendedrange/results/df_0115.png]

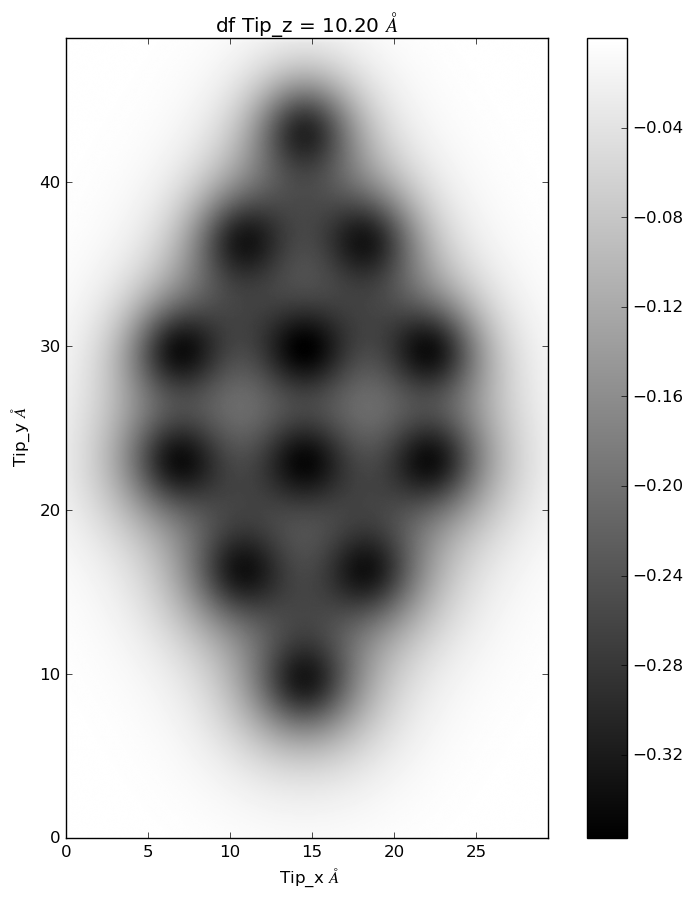

Supplement: File 7 — Datasets A0=1A k=0.5_extendedrange. [file Beilstein_J_Nanotechnol-07-937-s007.zip › S7/A0=1A/k=0.5_extendedrange/results/df_0116.png]

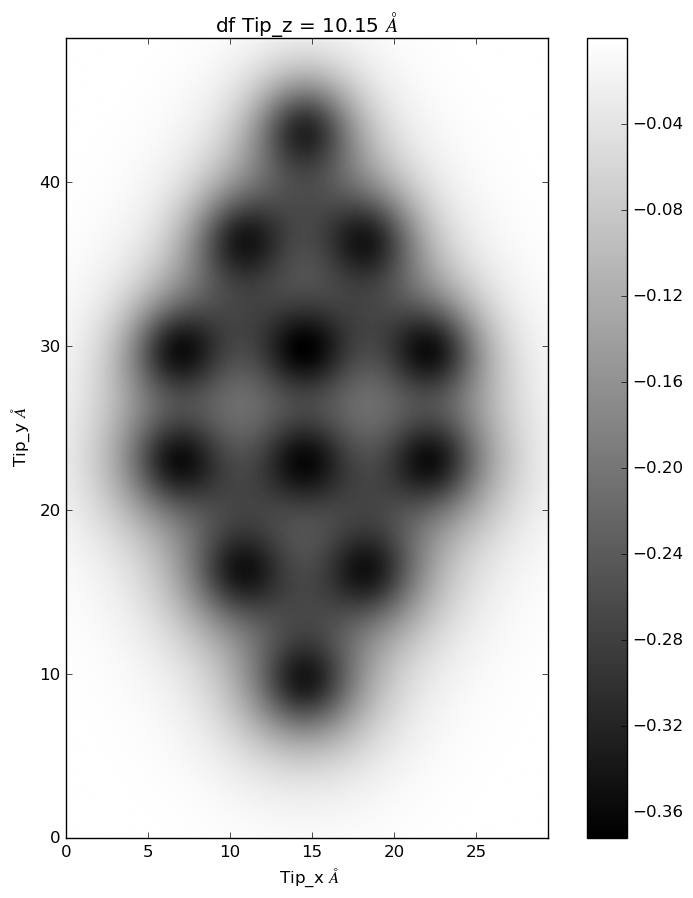

Supplement: File 7 — Datasets A0=1A k=0.5_extendedrange. [file Beilstein_J_Nanotechnol-07-937-s007.zip › S7/A0=1A/k=0.5_extendedrange/results/df_0117.png]

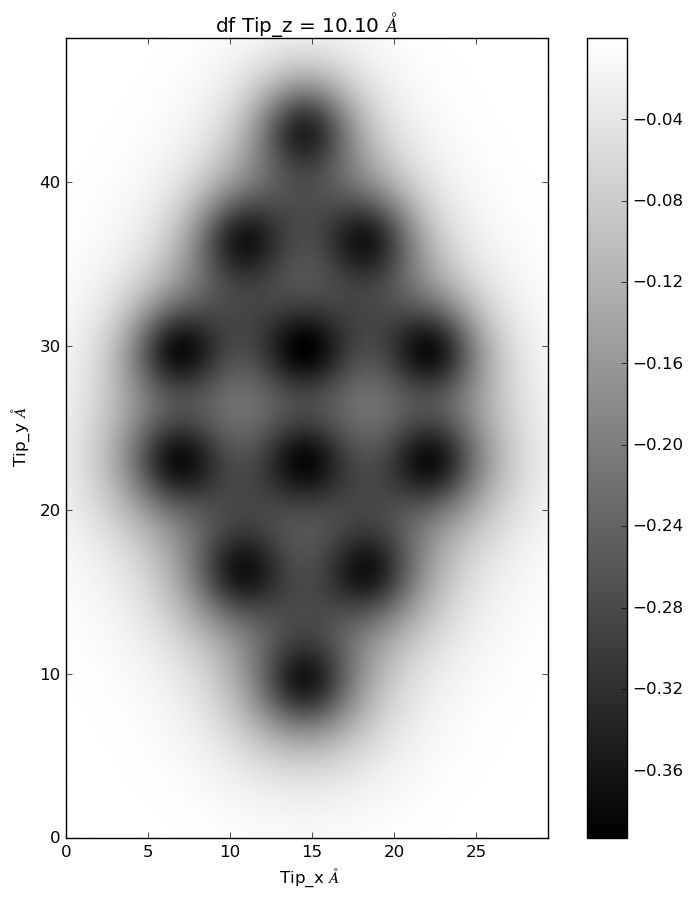

Supplement: File 7 — Datasets A0=1A k=0.5_extendedrange. [file Beilstein_J_Nanotechnol-07-937-s007.zip › S7/A0=1A/k=0.5_extendedrange/results/df_0118.png]

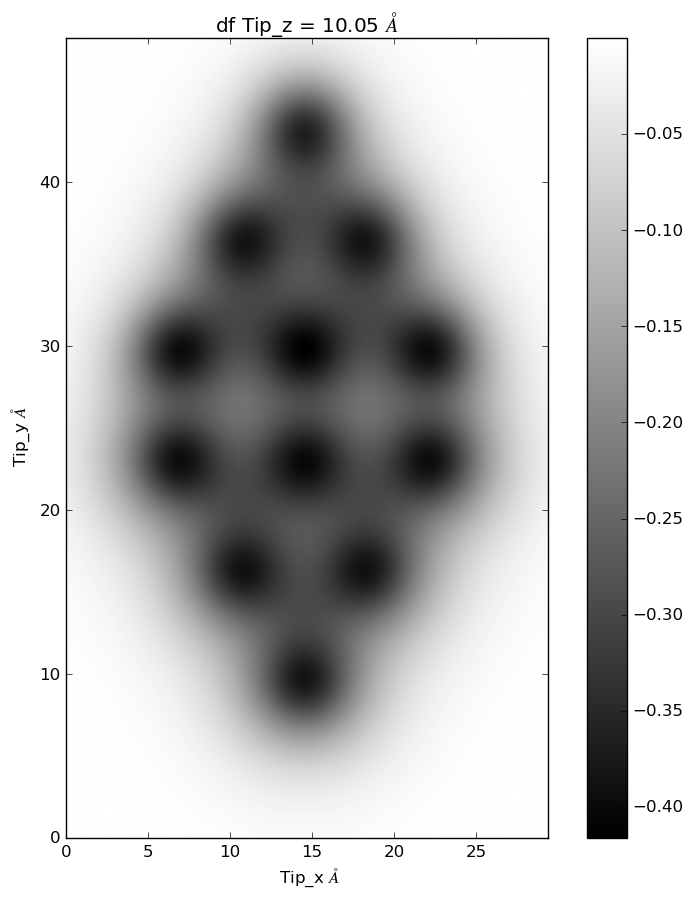

Supplement: File 7 — Datasets A0=1A k=0.5_extendedrange. [file Beilstein_J_Nanotechnol-07-937-s007.zip › S7/A0=1A/k=0.5_extendedrange/results/df_0119.png]

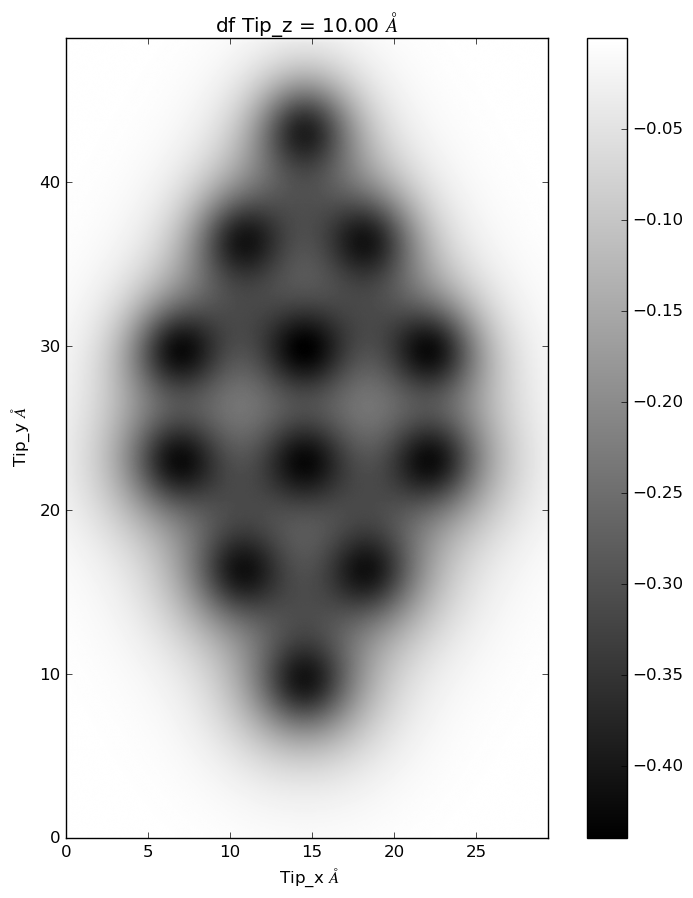

Supplement: File 7 — Datasets A0=1A k=0.5_extendedrange. [file Beilstein_J_Nanotechnol-07-937-s007.zip › S7/A0=1A/k=0.5_extendedrange/results/df_0120.png]

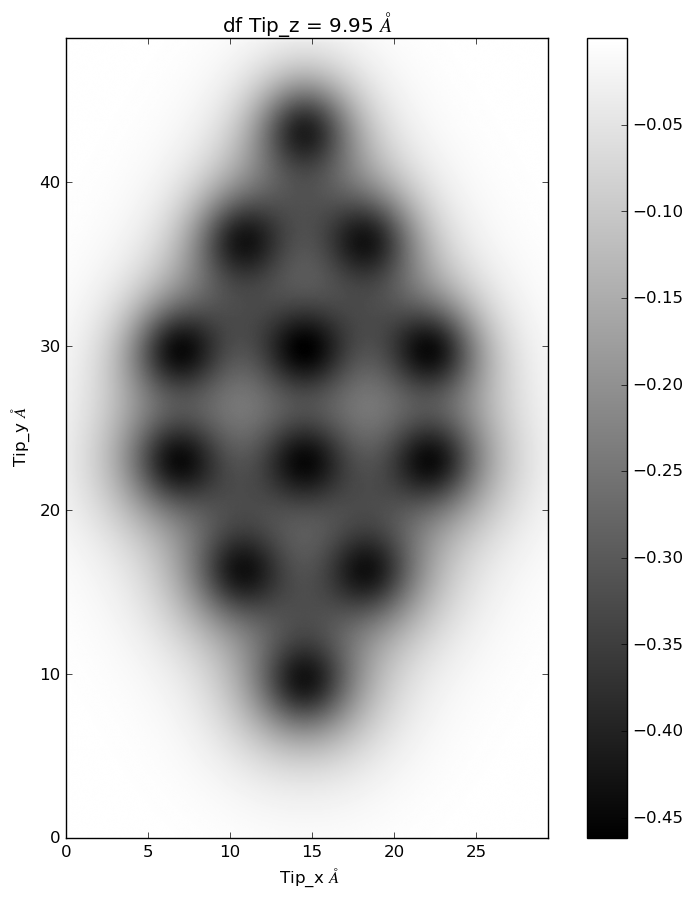

Supplement: File 7 — Datasets A0=1A k=0.5_extendedrange. [file Beilstein_J_Nanotechnol-07-937-s007.zip › S7/A0=1A/k=0.5_extendedrange/results/df_0121.png]

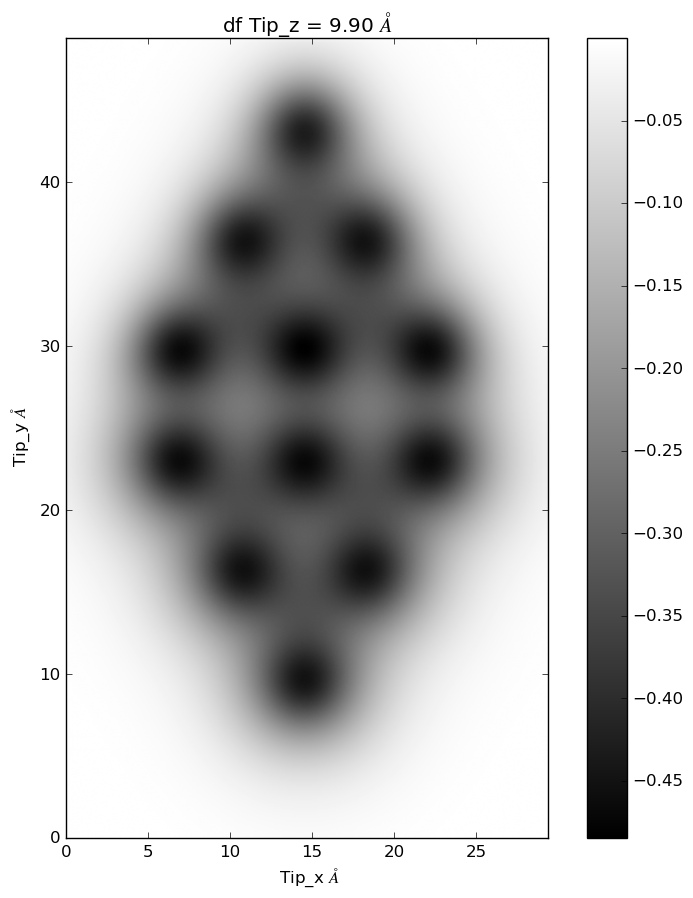

Supplement: File 7 — Datasets A0=1A k=0.5_extendedrange. [file Beilstein_J_Nanotechnol-07-937-s007.zip › S7/A0=1A/k=0.5_extendedrange/results/df_0122.png]

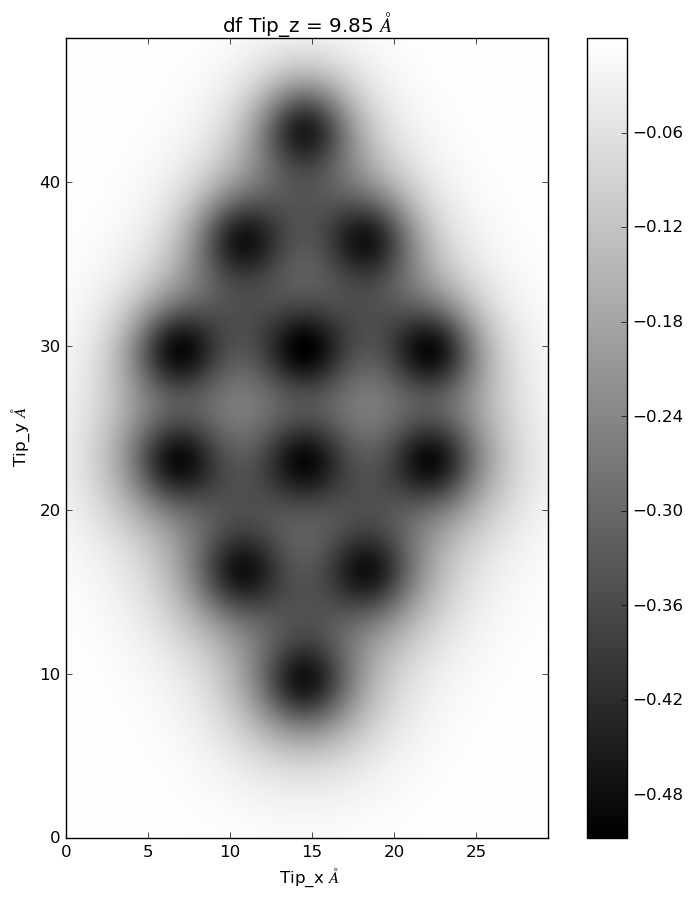

Supplement: File 7 — Datasets A0=1A k=0.5_extendedrange. [file Beilstein_J_Nanotechnol-07-937-s007.zip › S7/A0=1A/k=0.5_extendedrange/results/df_0123.png]

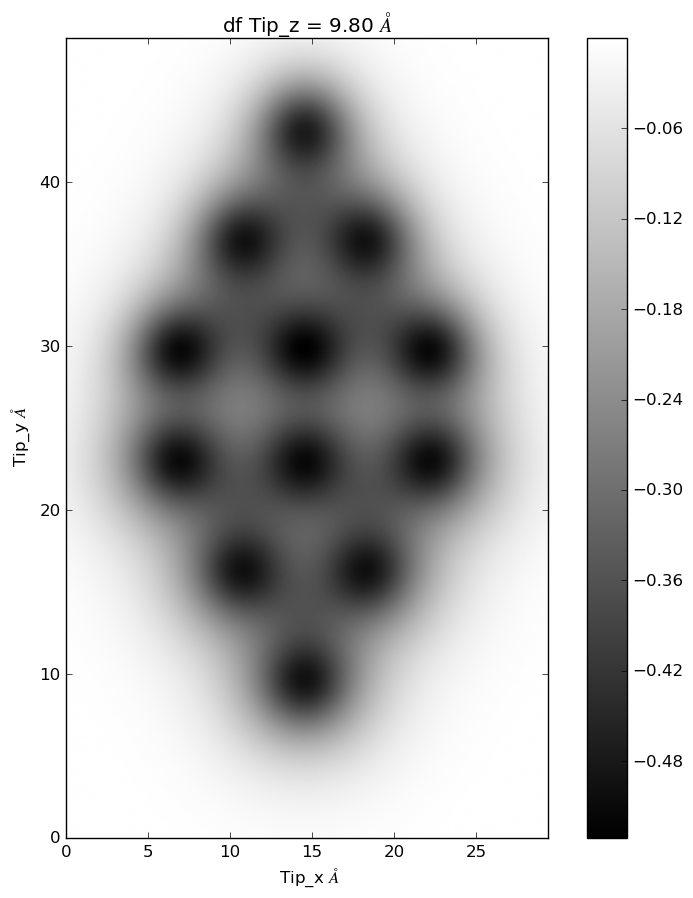

Supplement: File 7 — Datasets A0=1A k=0.5_extendedrange. [file Beilstein_J_Nanotechnol-07-937-s007.zip › S7/A0=1A/k=0.5_extendedrange/results/df_0124.png]

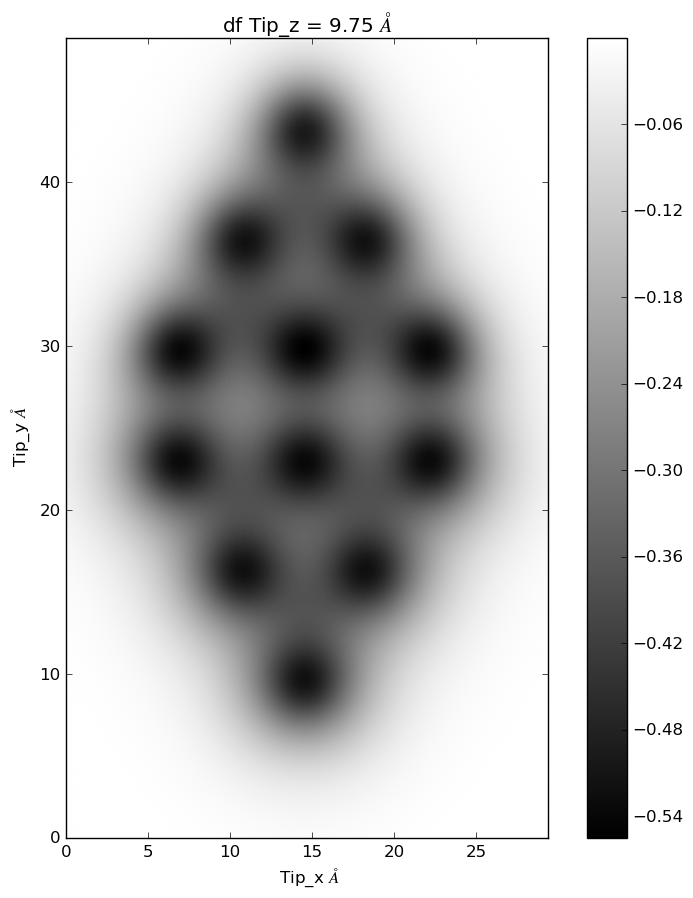

Supplement: File 7 — Datasets A0=1A k=0.5_extendedrange. [file Beilstein_J_Nanotechnol-07-937-s007.zip › S7/A0=1A/k=0.5_extendedrange/results/df_0125.png]

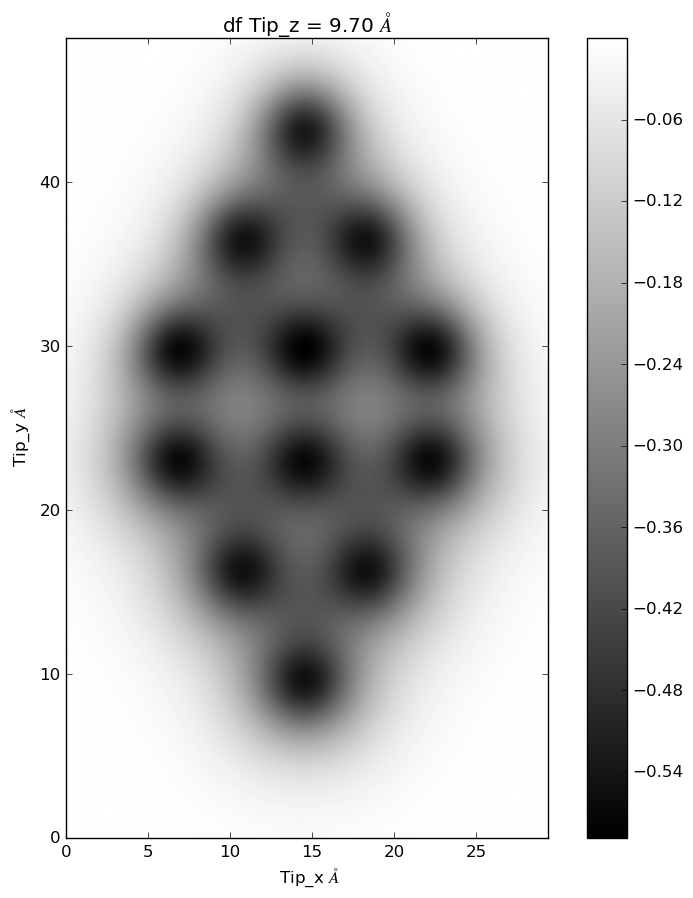

Supplement: File 7 — Datasets A0=1A k=0.5_extendedrange. [file Beilstein_J_Nanotechnol-07-937-s007.zip › S7/A0=1A/k=0.5_extendedrange/results/df_0126.png]

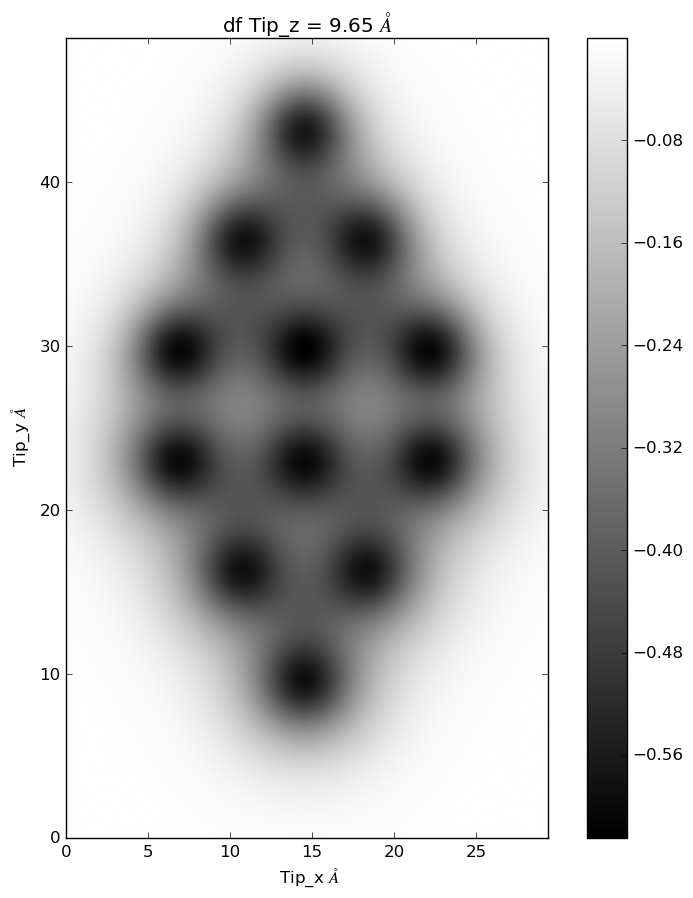

Supplement: File 7 — Datasets A0=1A k=0.5_extendedrange. [file Beilstein_J_Nanotechnol-07-937-s007.zip › S7/A0=1A/k=0.5_extendedrange/results/df_0127.png]

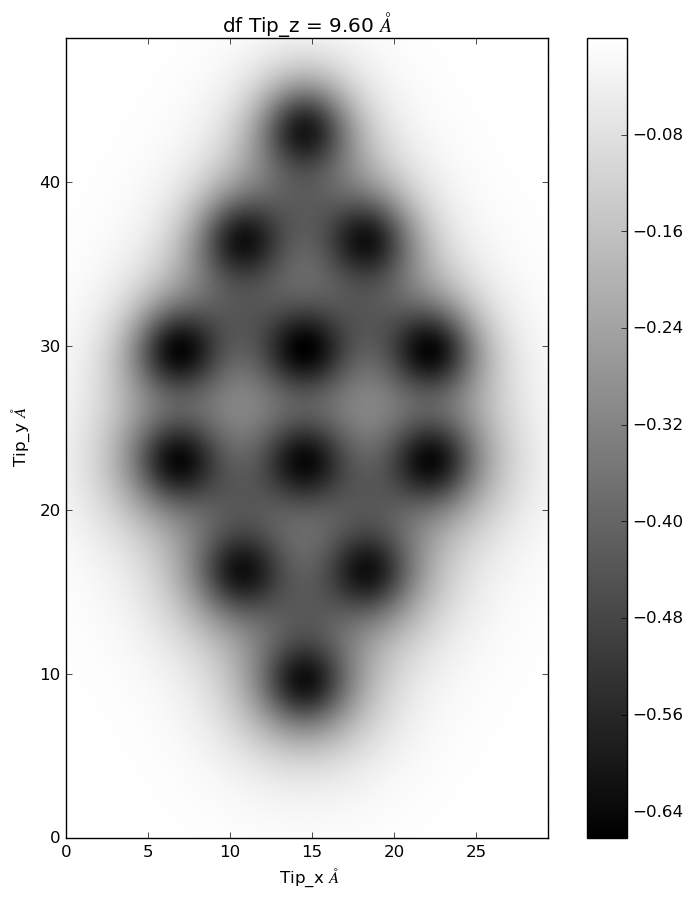

Supplement: File 7 — Datasets A0=1A k=0.5_extendedrange. [file Beilstein_J_Nanotechnol-07-937-s007.zip › S7/A0=1A/k=0.5_extendedrange/results/df_0128.png]

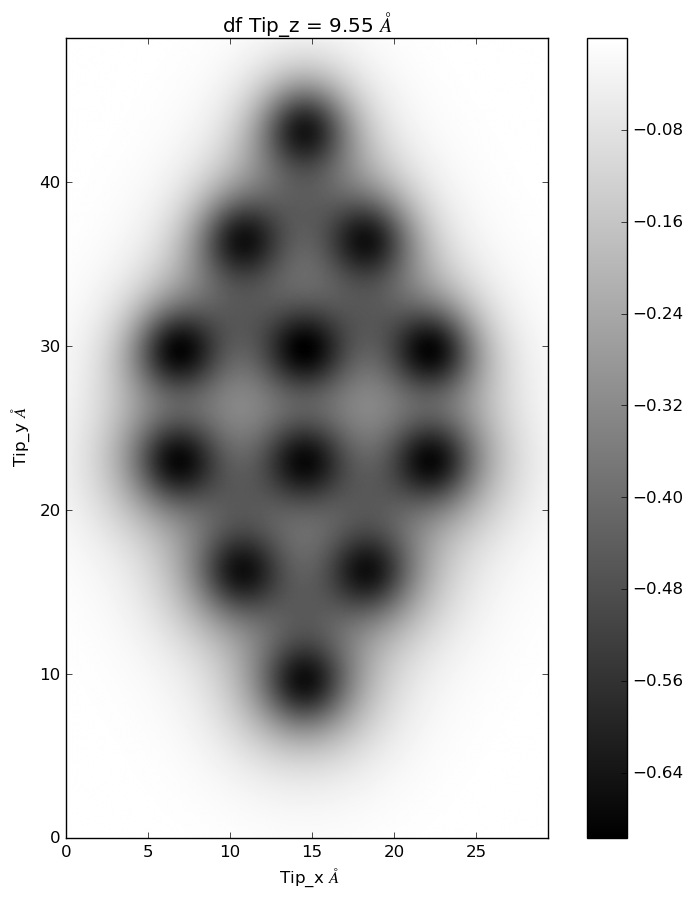

Supplement: File 7 — Datasets A0=1A k=0.5_extendedrange. [file Beilstein_J_Nanotechnol-07-937-s007.zip › S7/A0=1A/k=0.5_extendedrange/results/df_0129.png]

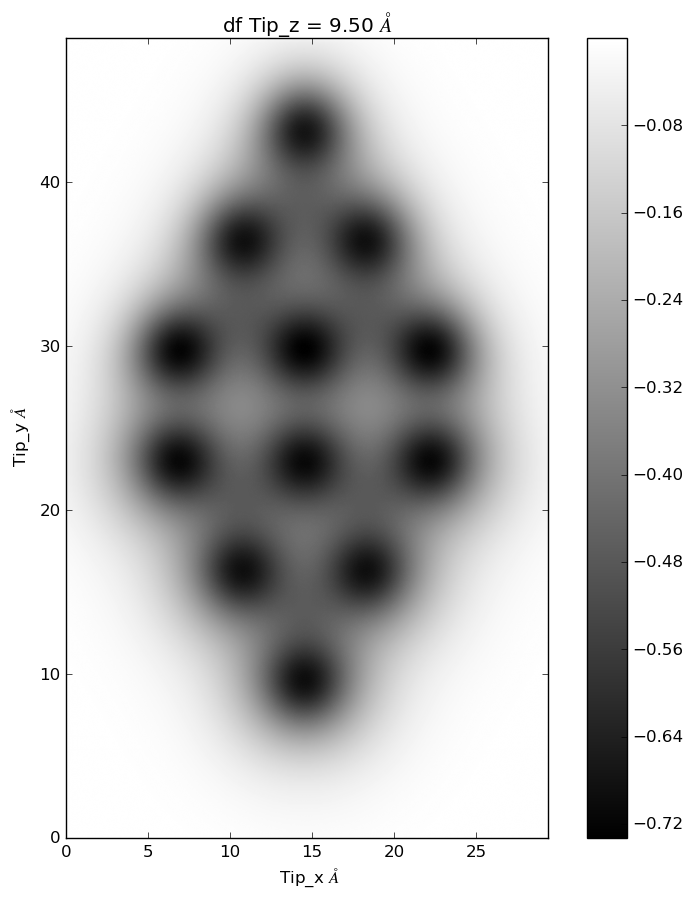

Supplement: File 7 — Datasets A0=1A k=0.5_extendedrange. [file Beilstein_J_Nanotechnol-07-937-s007.zip › S7/A0=1A/k=0.5_extendedrange/results/df_0130.png]

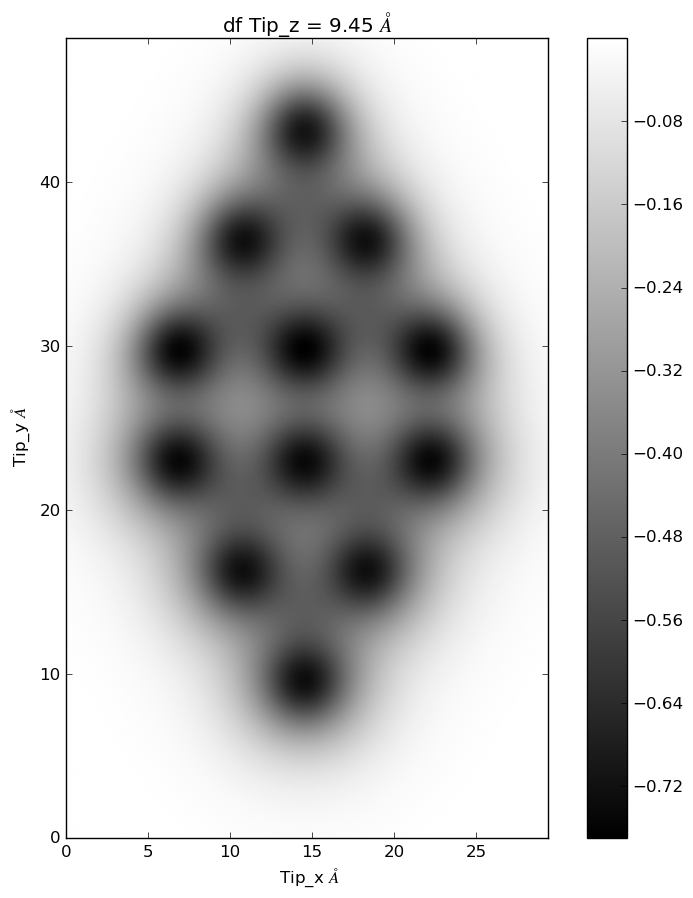

Supplement: File 7 — Datasets A0=1A k=0.5_extendedrange. [file Beilstein_J_Nanotechnol-07-937-s007.zip › S7/A0=1A/k=0.5_extendedrange/results/df_0131.png]

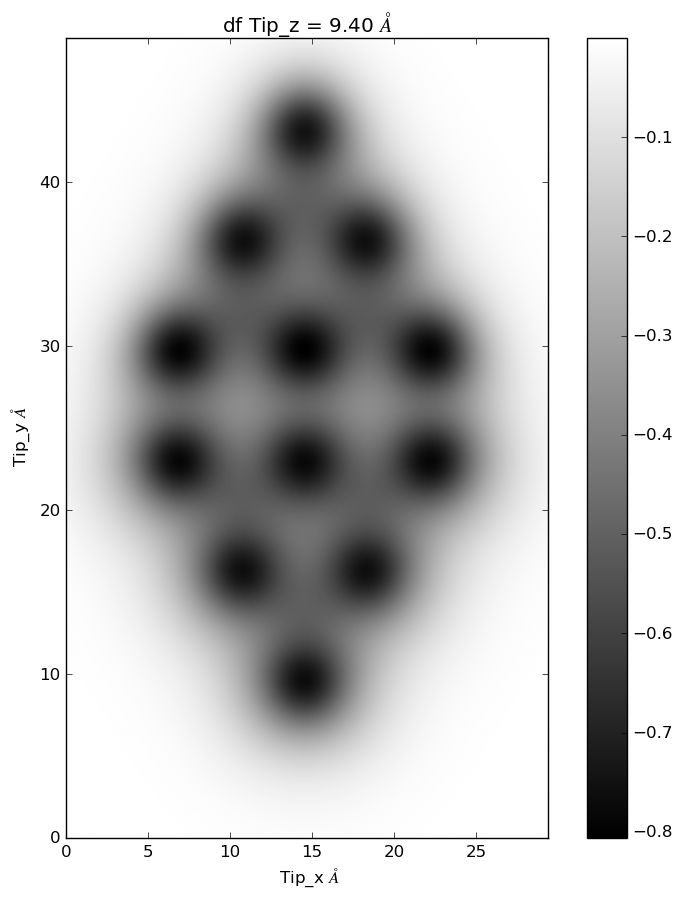

Supplement: File 7 — Datasets A0=1A k=0.5_extendedrange. [file Beilstein_J_Nanotechnol-07-937-s007.zip › S7/A0=1A/k=0.5_extendedrange/results/df_0132.png]

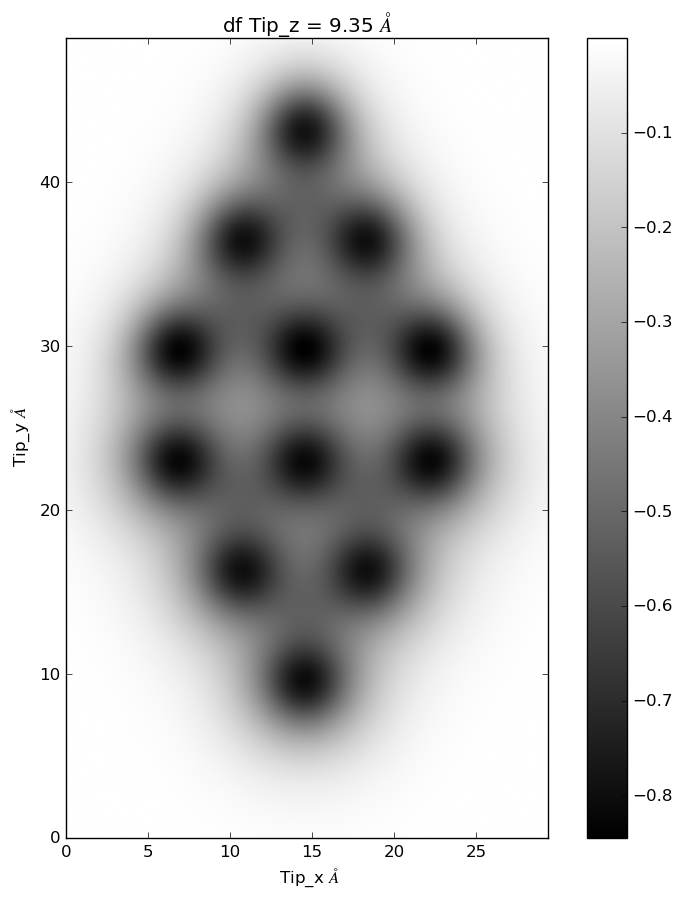

Supplement: File 7 — Datasets A0=1A k=0.5_extendedrange. [file Beilstein_J_Nanotechnol-07-937-s007.zip › S7/A0=1A/k=0.5_extendedrange/results/df_0133.png]

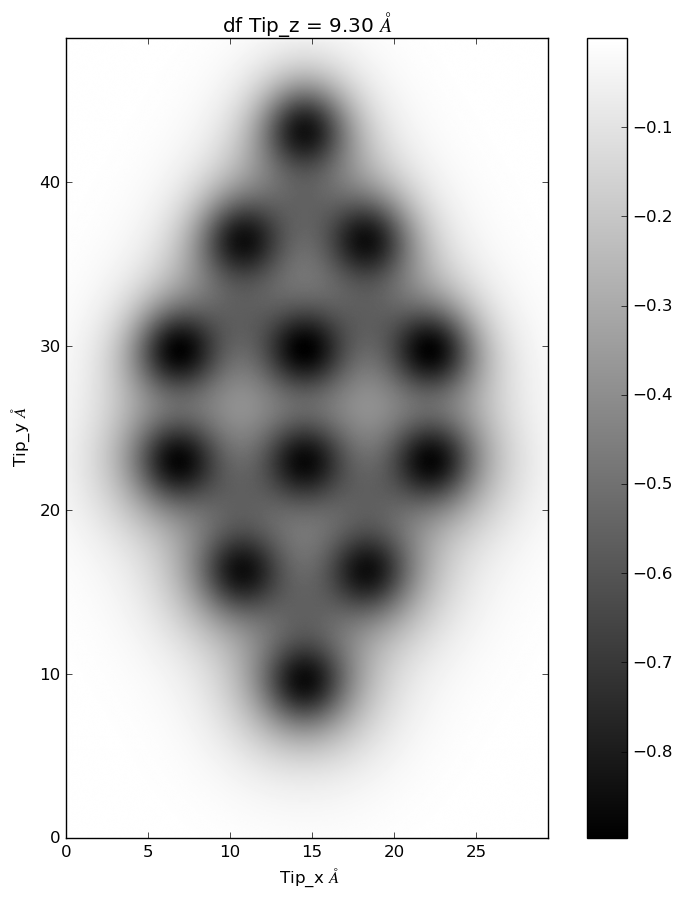

Supplement: File 7 — Datasets A0=1A k=0.5_extendedrange. [file Beilstein_J_Nanotechnol-07-937-s007.zip › S7/A0=1A/k=0.5_extendedrange/results/df_0134.png]

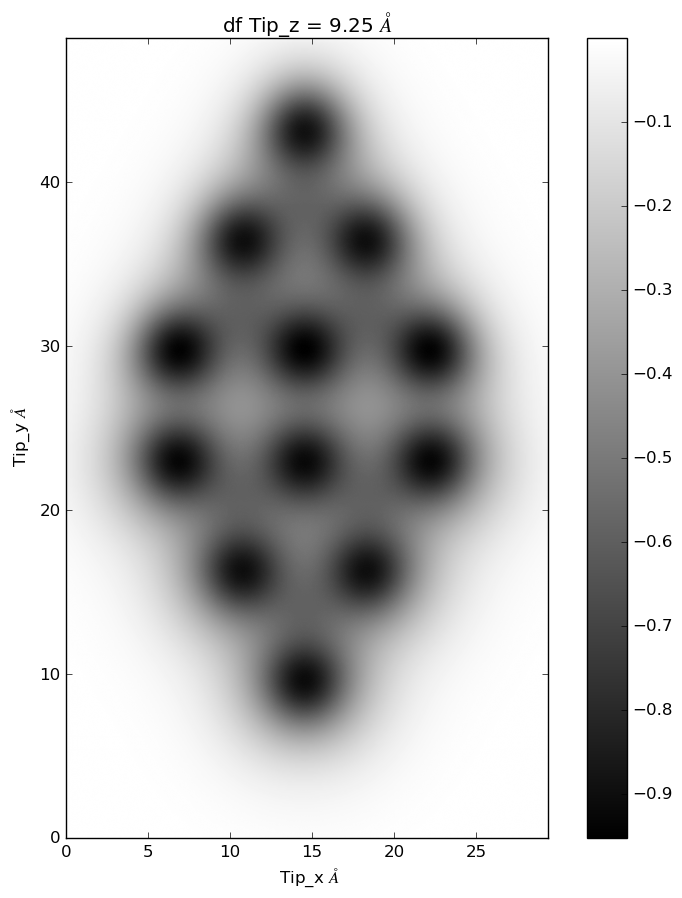

Supplement: File 7 — Datasets A0=1A k=0.5_extendedrange. [file Beilstein_J_Nanotechnol-07-937-s007.zip › S7/A0=1A/k=0.5_extendedrange/results/df_0135.png]

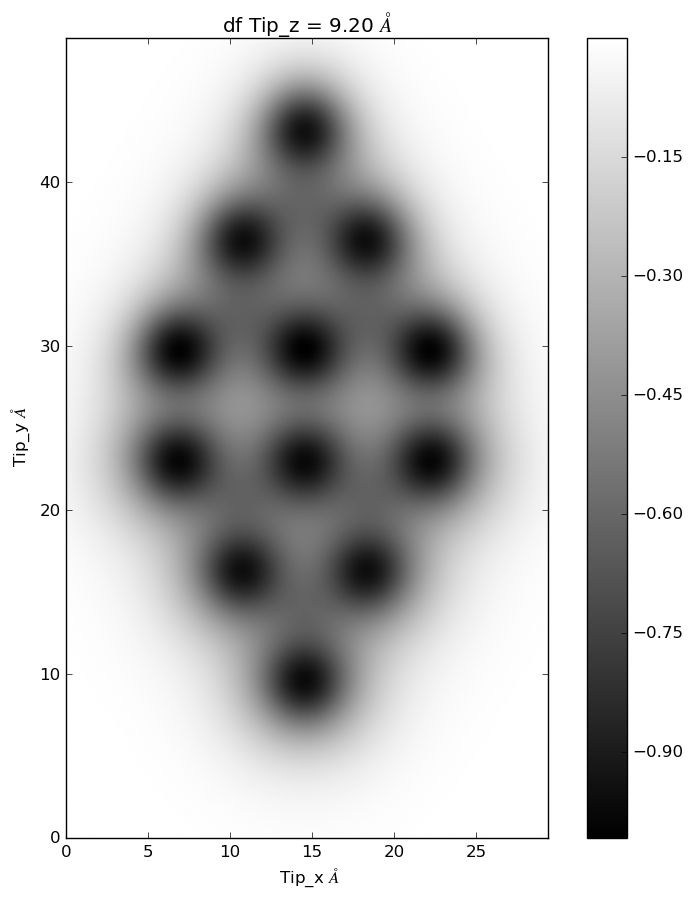

Supplement: File 7 — Datasets A0=1A k=0.5_extendedrange. [file Beilstein_J_Nanotechnol-07-937-s007.zip › S7/A0=1A/k=0.5_extendedrange/results/df_0136.png]

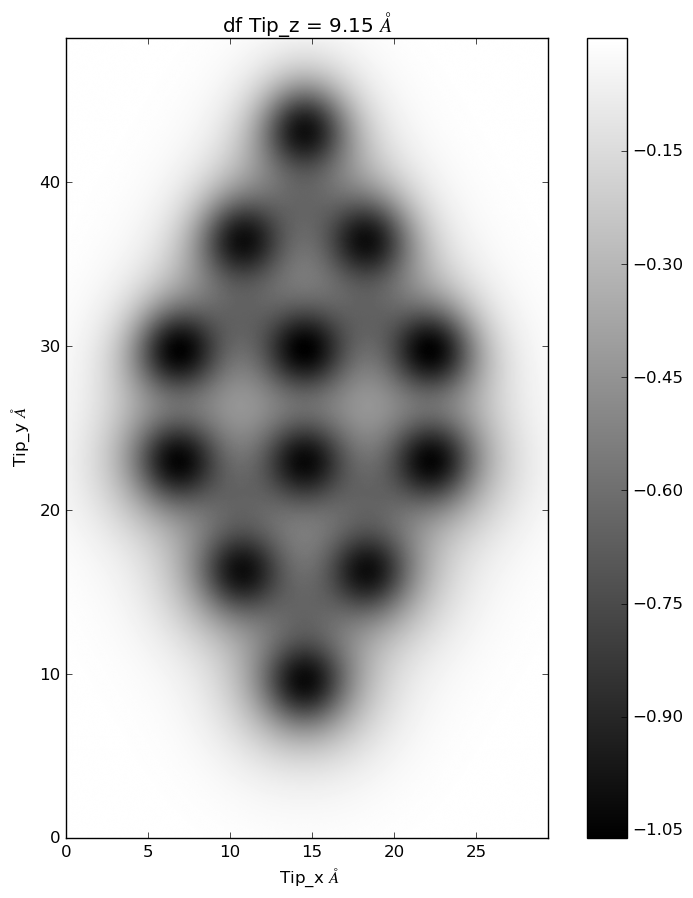

Supplement: File 7 — Datasets A0=1A k=0.5_extendedrange. [file Beilstein_J_Nanotechnol-07-937-s007.zip › S7/A0=1A/k=0.5_extendedrange/results/df_0137.png]

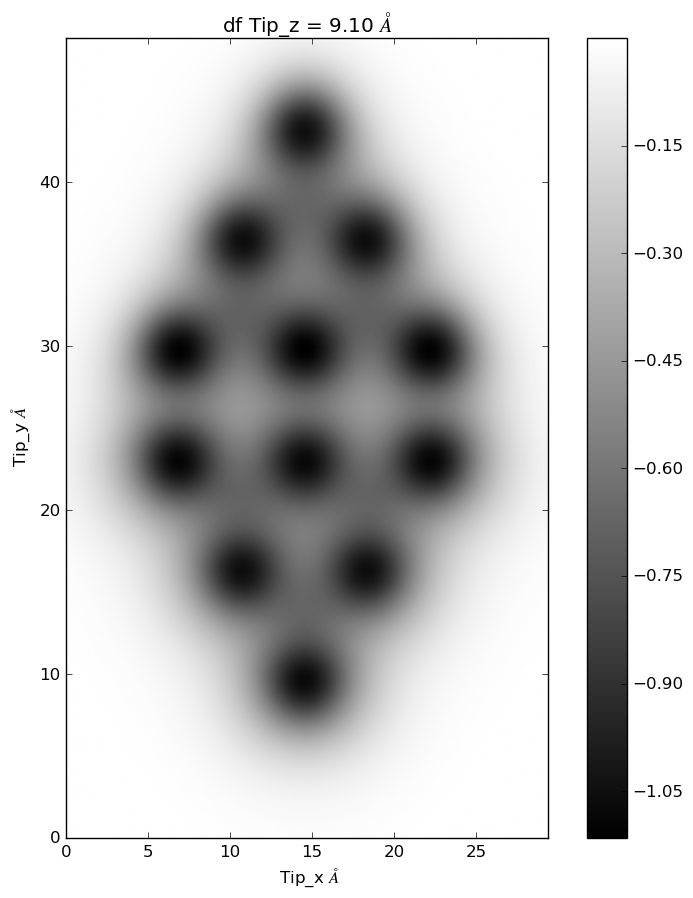

Supplement: File 7 — Datasets A0=1A k=0.5_extendedrange. [file Beilstein_J_Nanotechnol-07-937-s007.zip › S7/A0=1A/k=0.5_extendedrange/results/df_0138.png]

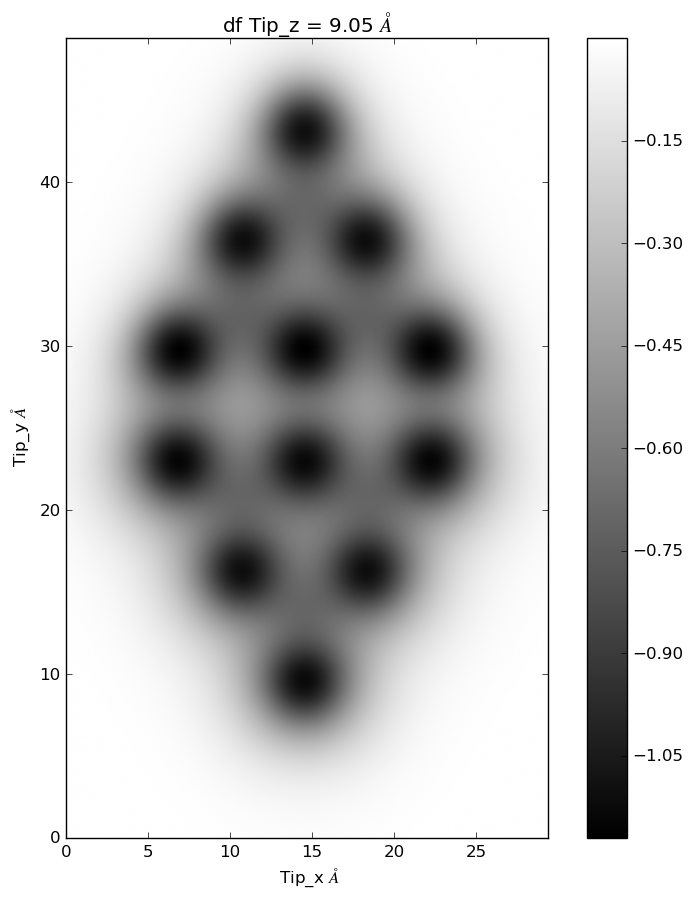

Supplement: File 7 — Datasets A0=1A k=0.5_extendedrange. [file Beilstein_J_Nanotechnol-07-937-s007.zip › S7/A0=1A/k=0.5_extendedrange/results/df_0139.png]

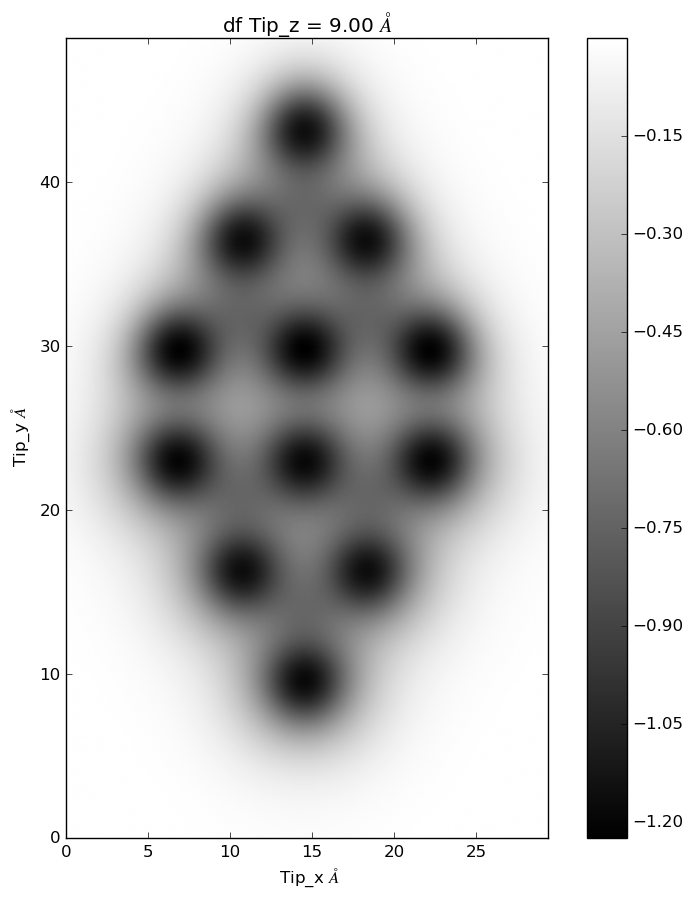

Supplement: File 7 — Datasets A0=1A k=0.5_extendedrange. [file Beilstein_J_Nanotechnol-07-937-s007.zip › S7/A0=1A/k=0.5_extendedrange/results/df_0140.png]

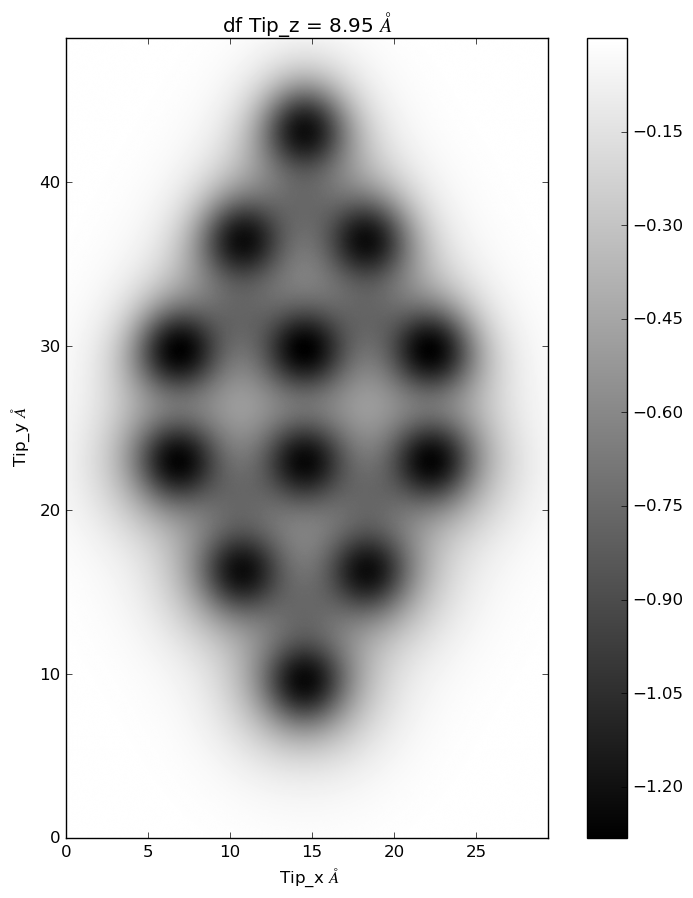

Supplement: File 7 — Datasets A0=1A k=0.5_extendedrange. [file Beilstein_J_Nanotechnol-07-937-s007.zip › S7/A0=1A/k=0.5_extendedrange/results/df_0141.png]

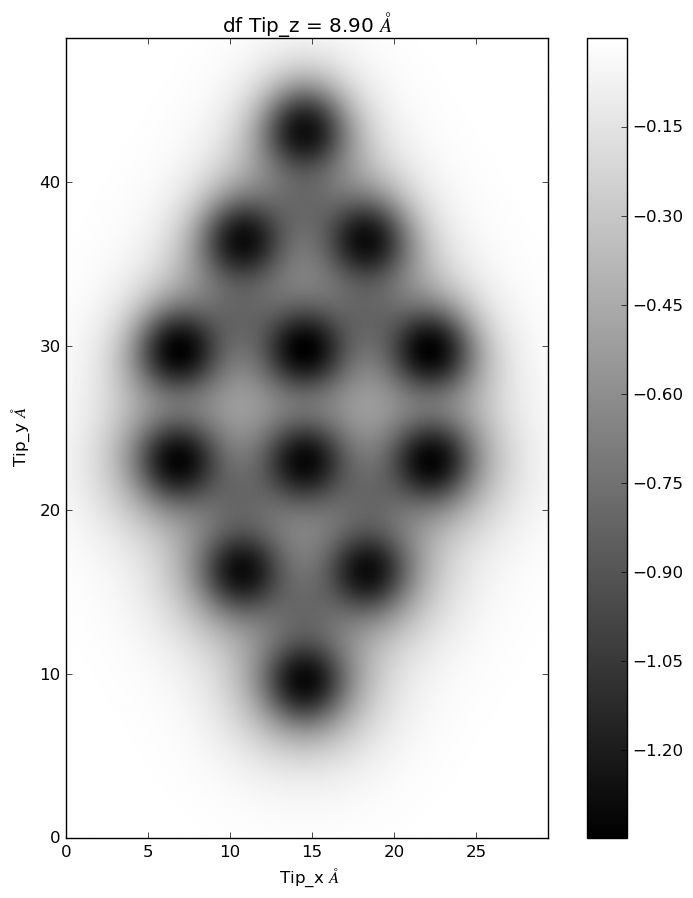

Supplement: File 7 — Datasets A0=1A k=0.5_extendedrange. [file Beilstein_J_Nanotechnol-07-937-s007.zip › S7/A0=1A/k=0.5_extendedrange/results/df_0142.png]

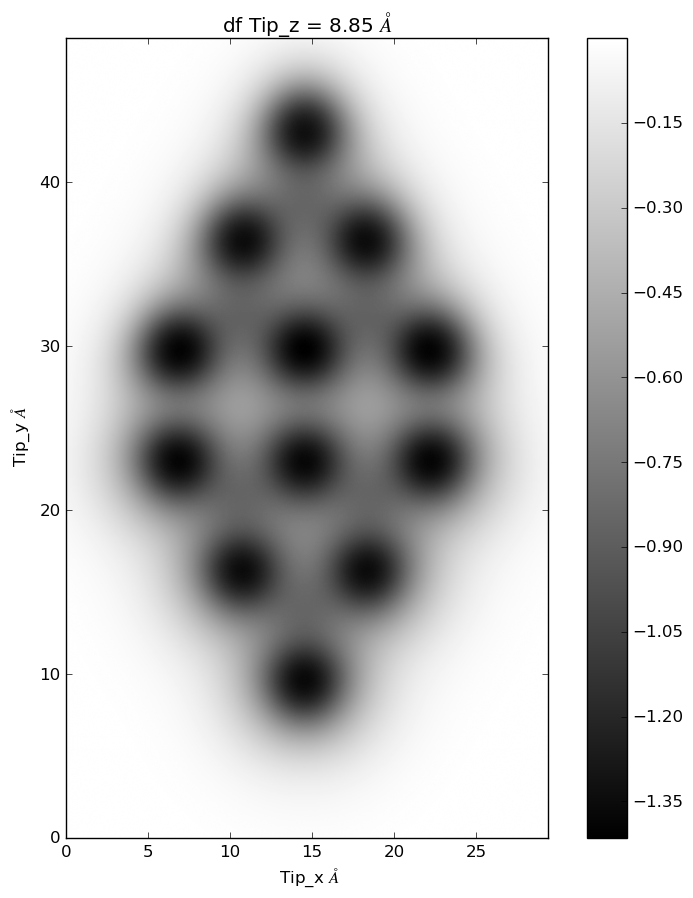

Supplement: File 7 — Datasets A0=1A k=0.5_extendedrange. [file Beilstein_J_Nanotechnol-07-937-s007.zip › S7/A0=1A/k=0.5_extendedrange/results/df_0143.png]

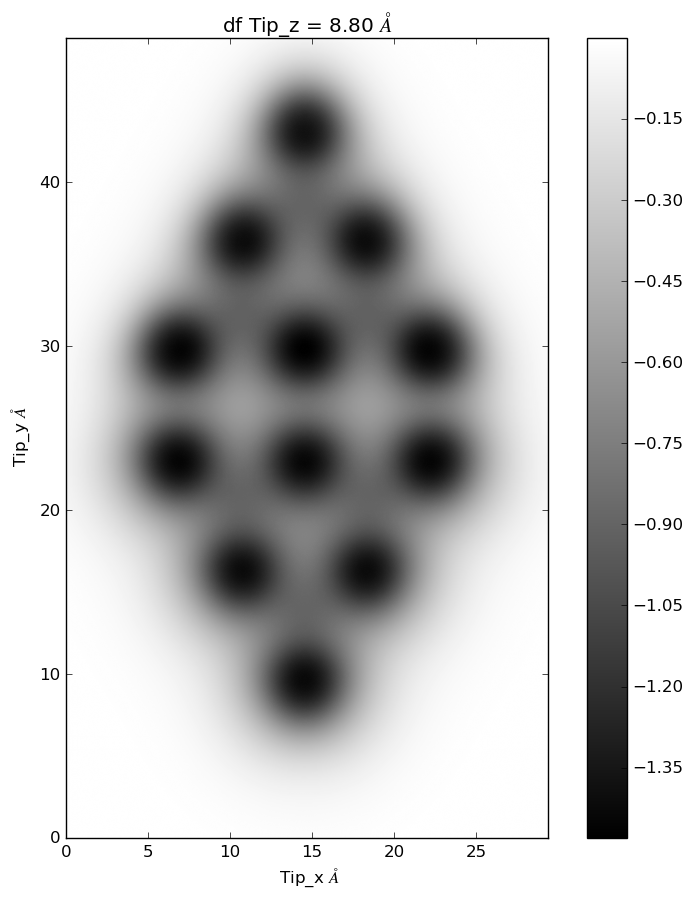

Supplement: File 7 — Datasets A0=1A k=0.5_extendedrange. [file Beilstein_J_Nanotechnol-07-937-s007.zip › S7/A0=1A/k=0.5_extendedrange/results/df_0144.png]

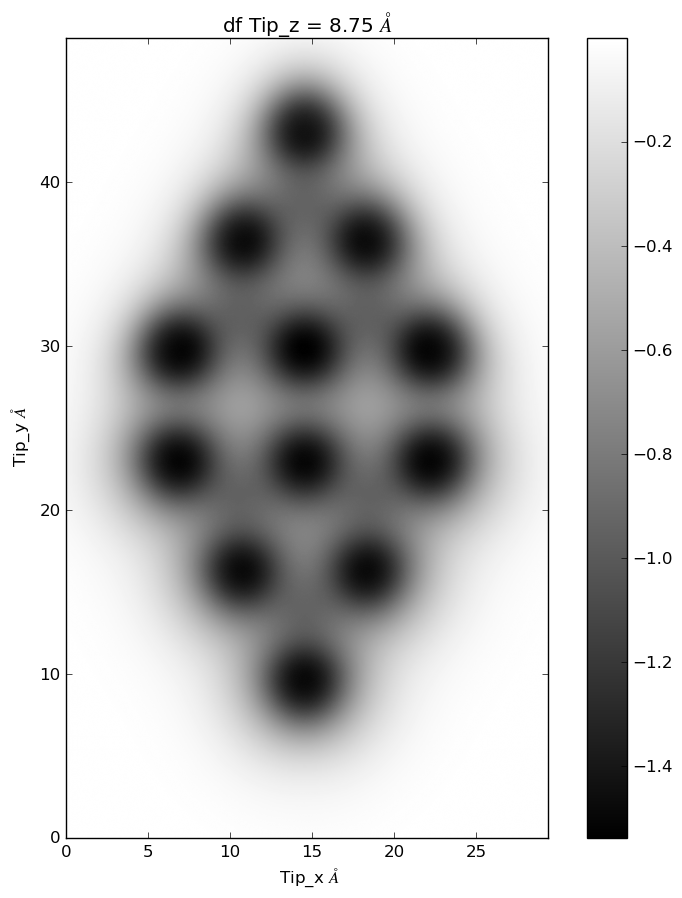

Supplement: File 7 — Datasets A0=1A k=0.5_extendedrange. [file Beilstein_J_Nanotechnol-07-937-s007.zip › S7/A0=1A/k=0.5_extendedrange/results/df_0145.png]

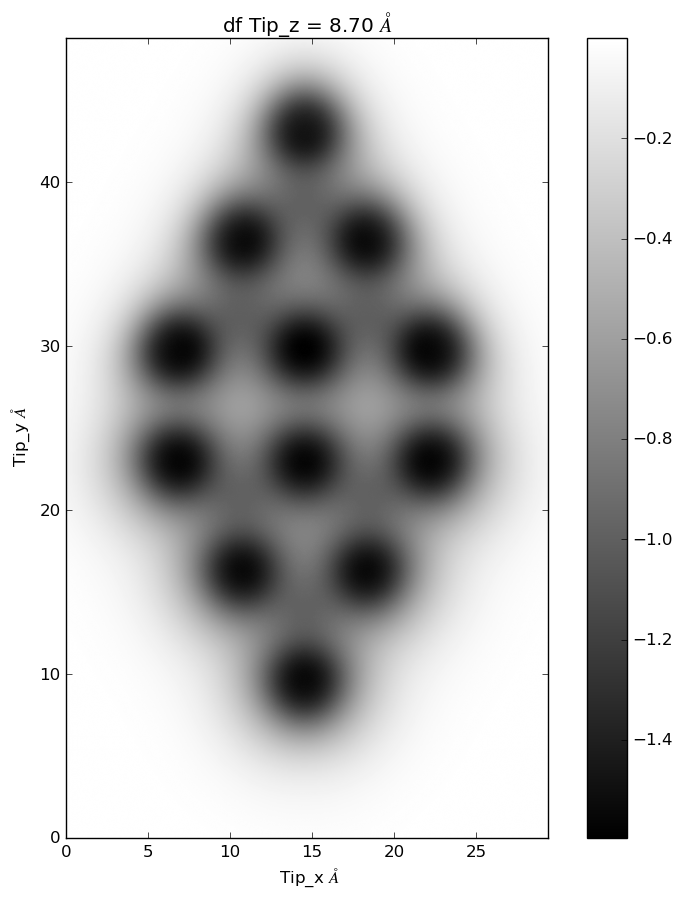

Supplement: File 7 — Datasets A0=1A k=0.5_extendedrange. [file Beilstein_J_Nanotechnol-07-937-s007.zip › S7/A0=1A/k=0.5_extendedrange/results/df_0146.png]

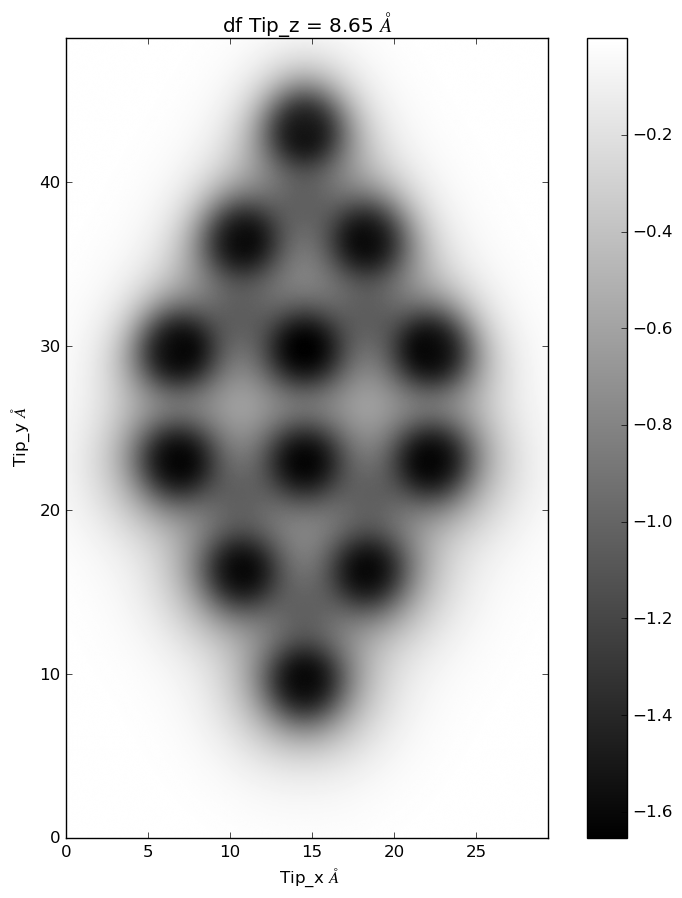

Supplement: File 7 — Datasets A0=1A k=0.5_extendedrange. [file Beilstein_J_Nanotechnol-07-937-s007.zip › S7/A0=1A/k=0.5_extendedrange/results/df_0147.png]

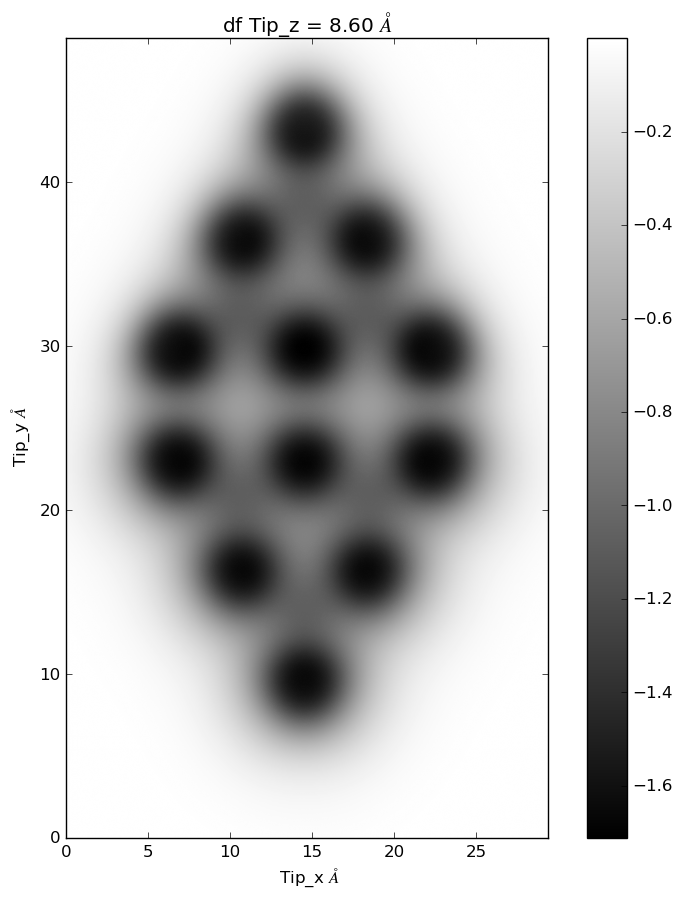

Supplement: File 7 — Datasets A0=1A k=0.5_extendedrange. [file Beilstein_J_Nanotechnol-07-937-s007.zip › S7/A0=1A/k=0.5_extendedrange/results/df_0148.png]

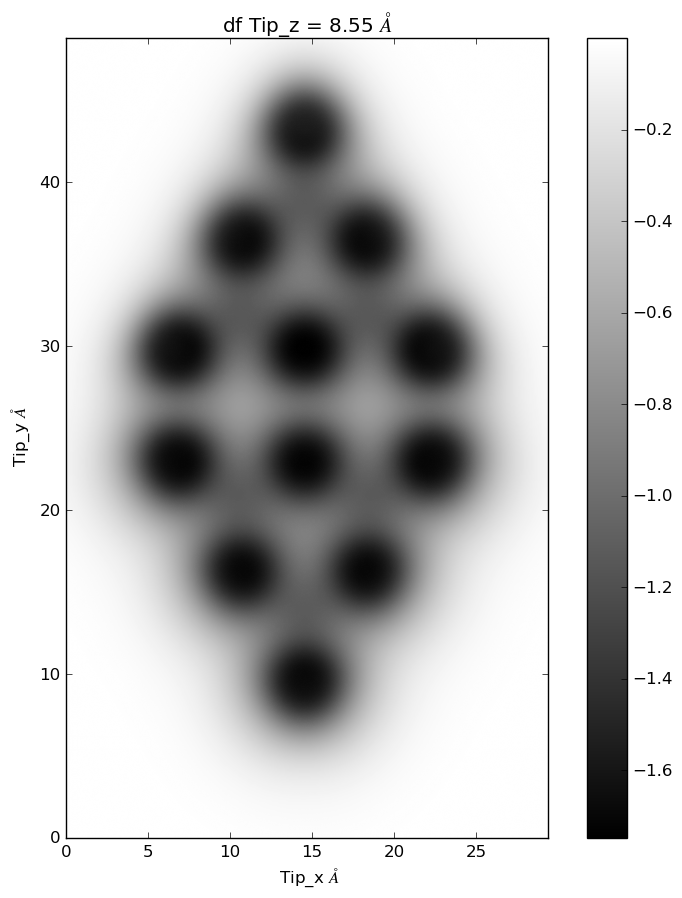

Supplement: File 7 — Datasets A0=1A k=0.5_extendedrange. [file Beilstein_J_Nanotechnol-07-937-s007.zip › S7/A0=1A/k=0.5_extendedrange/results/df_0149.png]

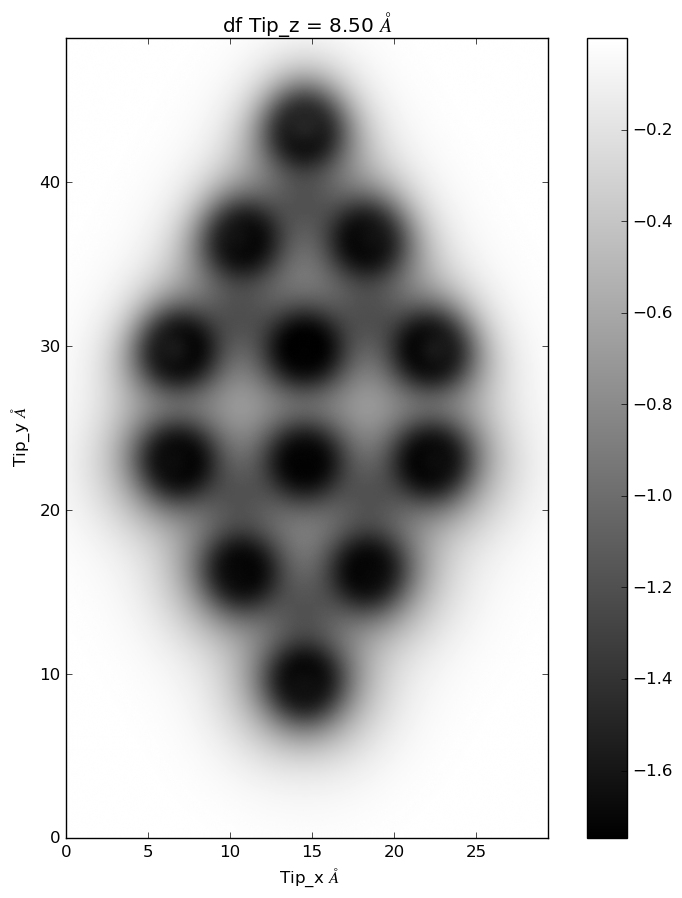

Supplement: File 7 — Datasets A0=1A k=0.5_extendedrange. [file Beilstein_J_Nanotechnol-07-937-s007.zip › S7/A0=1A/k=0.5_extendedrange/results/df_0150.png]

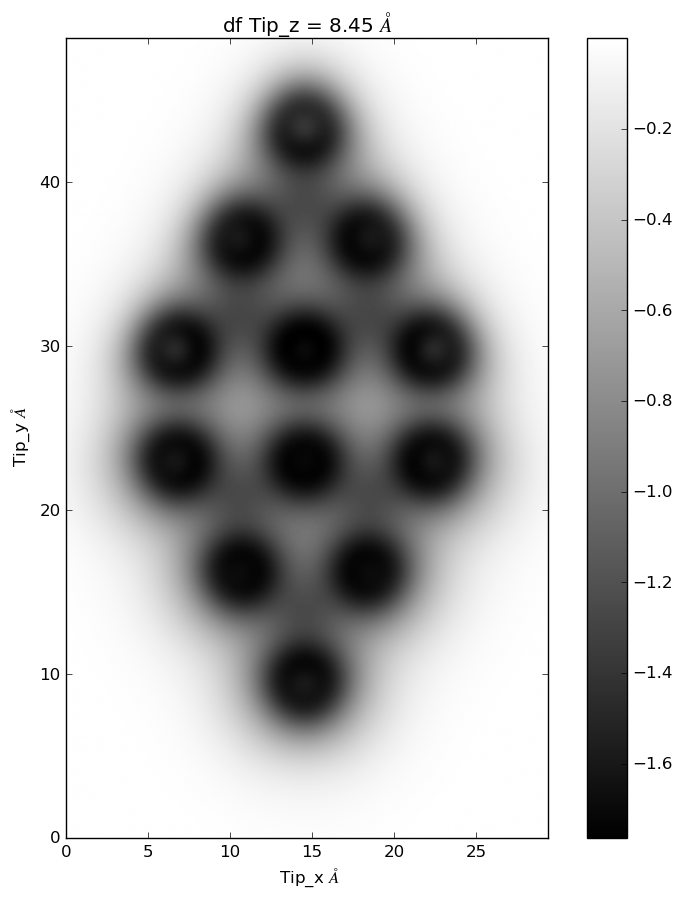

Supplement: File 7 — Datasets A0=1A k=0.5_extendedrange. [file Beilstein_J_Nanotechnol-07-937-s007.zip › S7/A0=1A/k=0.5_extendedrange/results/df_0151.png]

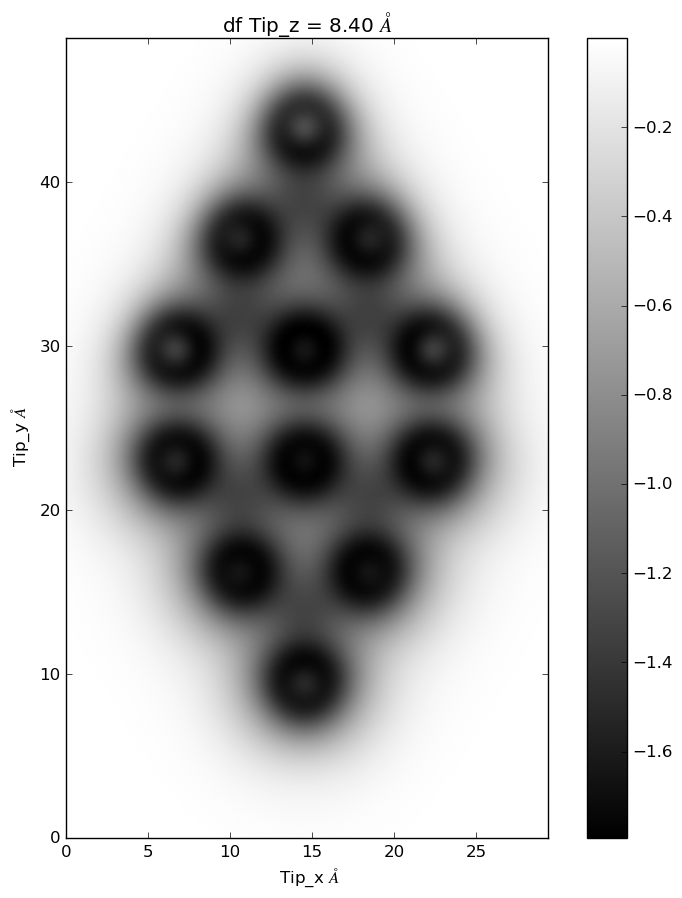

Supplement: File 7 — Datasets A0=1A k=0.5_extendedrange. [file Beilstein_J_Nanotechnol-07-937-s007.zip › S7/A0=1A/k=0.5_extendedrange/results/df_0152.png]

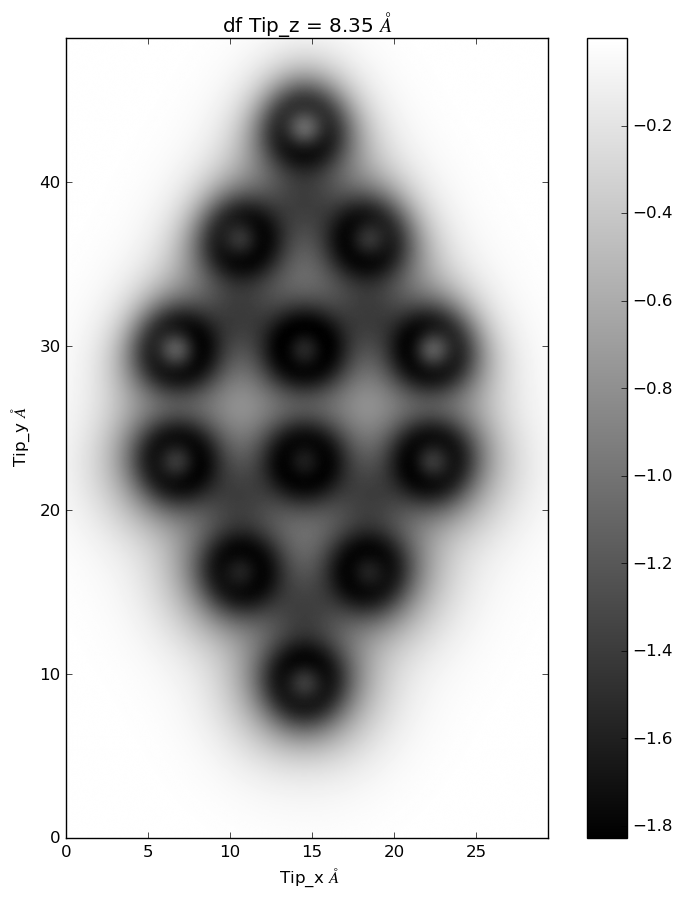

Supplement: File 7 — Datasets A0=1A k=0.5_extendedrange. [file Beilstein_J_Nanotechnol-07-937-s007.zip › S7/A0=1A/k=0.5_extendedrange/results/df_0153.png]

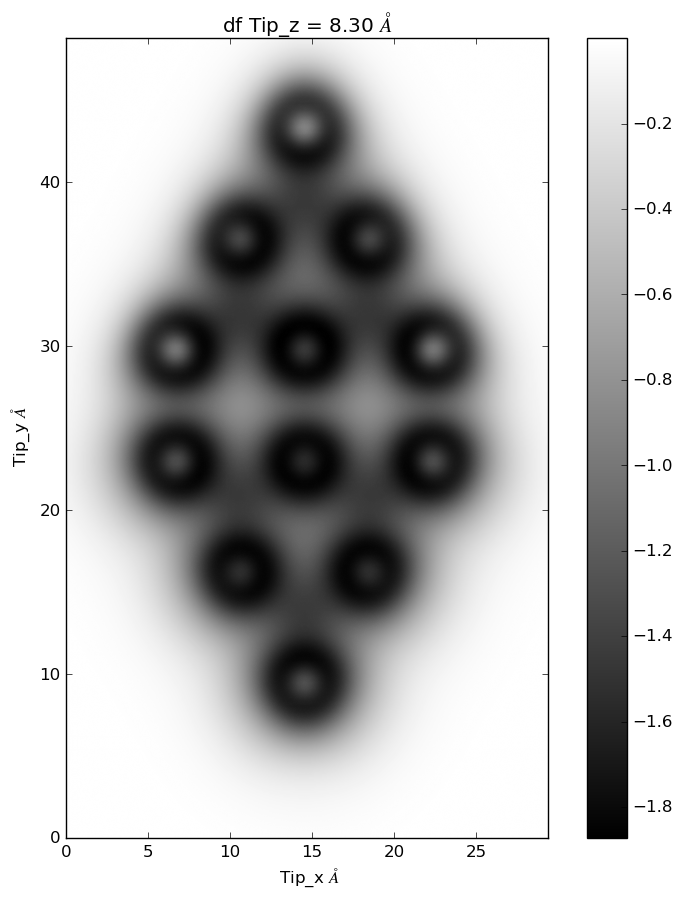

Supplement: File 7 — Datasets A0=1A k=0.5_extendedrange. [file Beilstein_J_Nanotechnol-07-937-s007.zip › S7/A0=1A/k=0.5_extendedrange/results/df_0154.png]

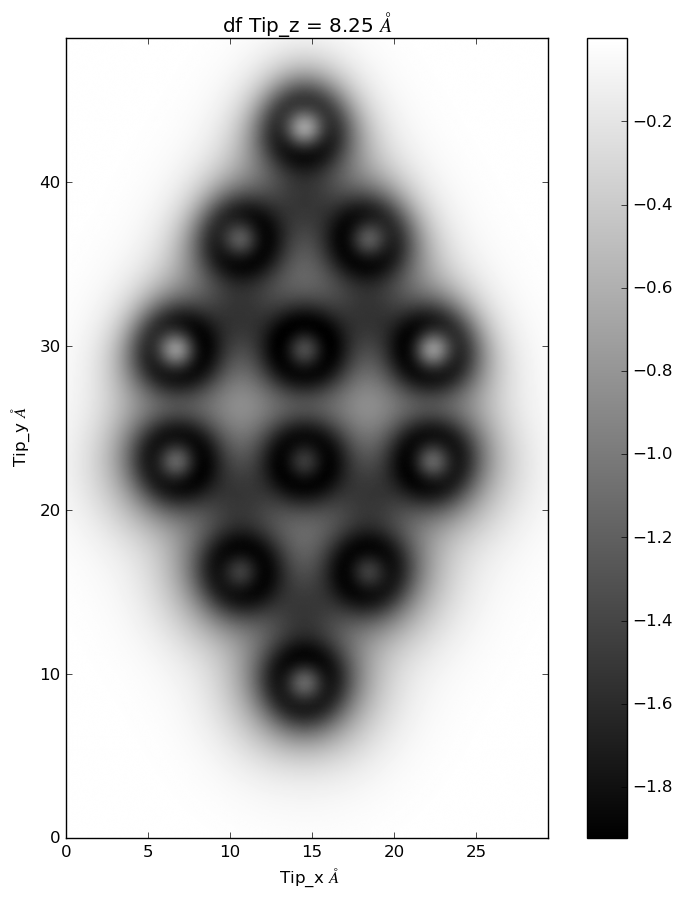

Supplement: File 7 — Datasets A0=1A k=0.5_extendedrange. [file Beilstein_J_Nanotechnol-07-937-s007.zip › S7/A0=1A/k=0.5_extendedrange/results/df_0155.png]

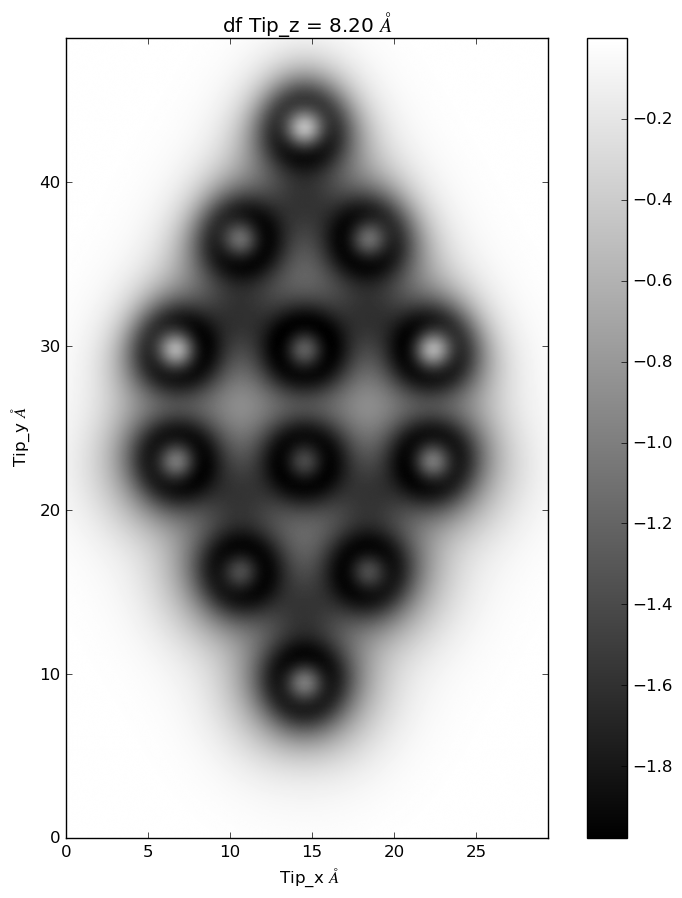

Supplement: File 7 — Datasets A0=1A k=0.5_extendedrange. [file Beilstein_J_Nanotechnol-07-937-s007.zip › S7/A0=1A/k=0.5_extendedrange/results/df_0156.png]

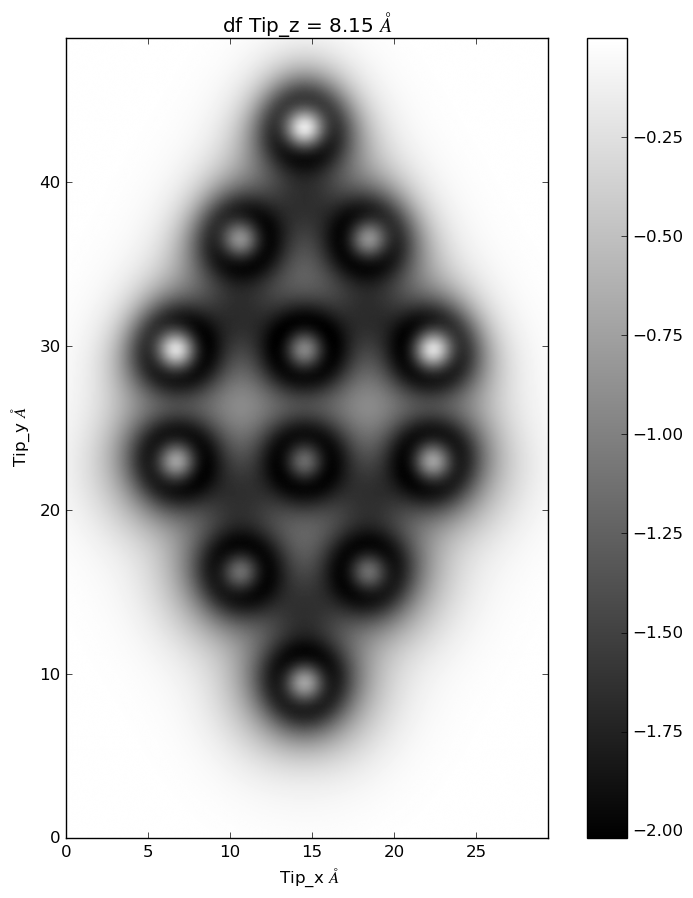

Supplement: File 7 — Datasets A0=1A k=0.5_extendedrange. [file Beilstein_J_Nanotechnol-07-937-s007.zip › S7/A0=1A/k=0.5_extendedrange/results/df_0157.png]

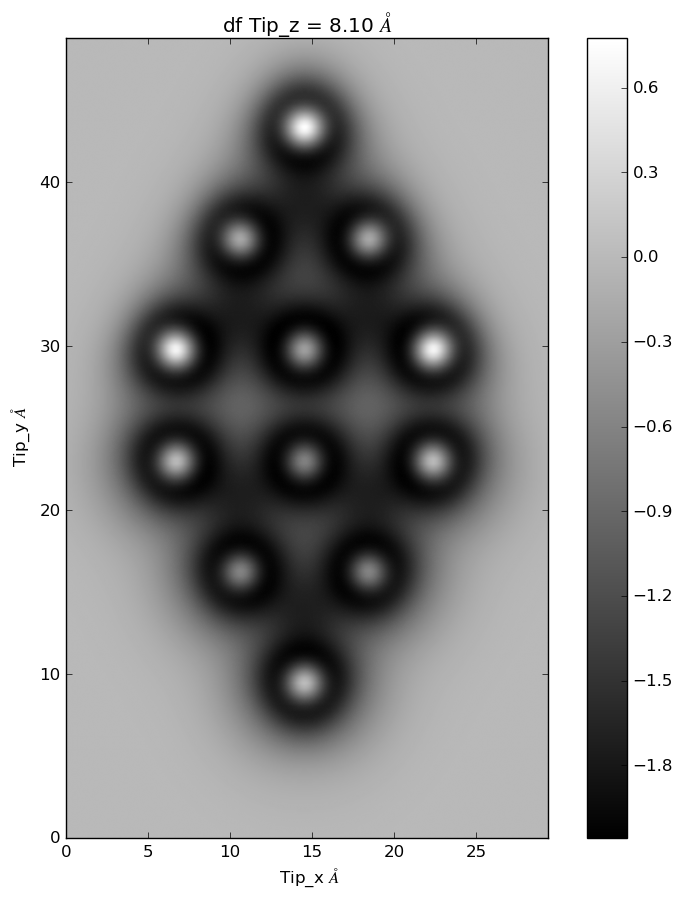

Supplement: File 7 — Datasets A0=1A k=0.5_extendedrange. [file Beilstein_J_Nanotechnol-07-937-s007.zip › S7/A0=1A/k=0.5_extendedrange/results/df_0158.png]

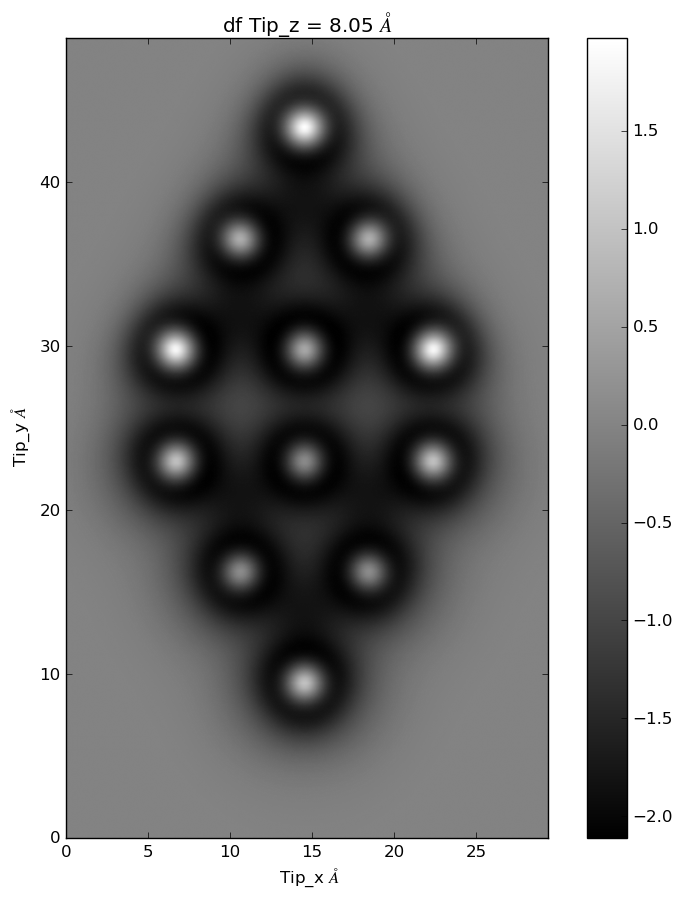

Supplement: File 7 — Datasets A0=1A k=0.5_extendedrange. [file Beilstein_J_Nanotechnol-07-937-s007.zip › S7/A0=1A/k=0.5_extendedrange/results/df_0159.png]

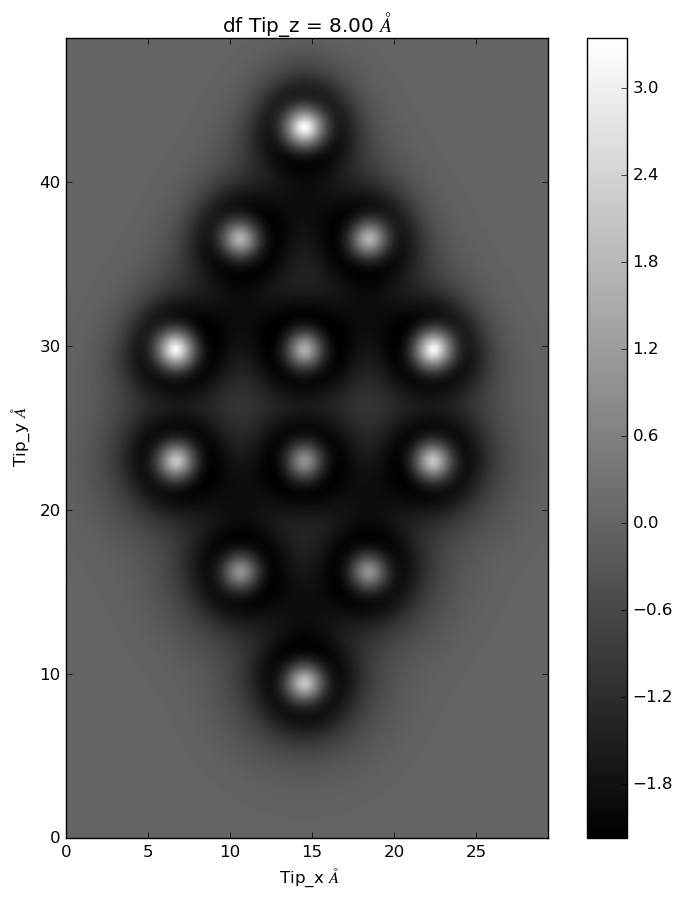

Supplement: File 7 — Datasets A0=1A k=0.5_extendedrange. [file Beilstein_J_Nanotechnol-07-937-s007.zip › S7/A0=1A/k=0.5_extendedrange/results/df_0160.png]

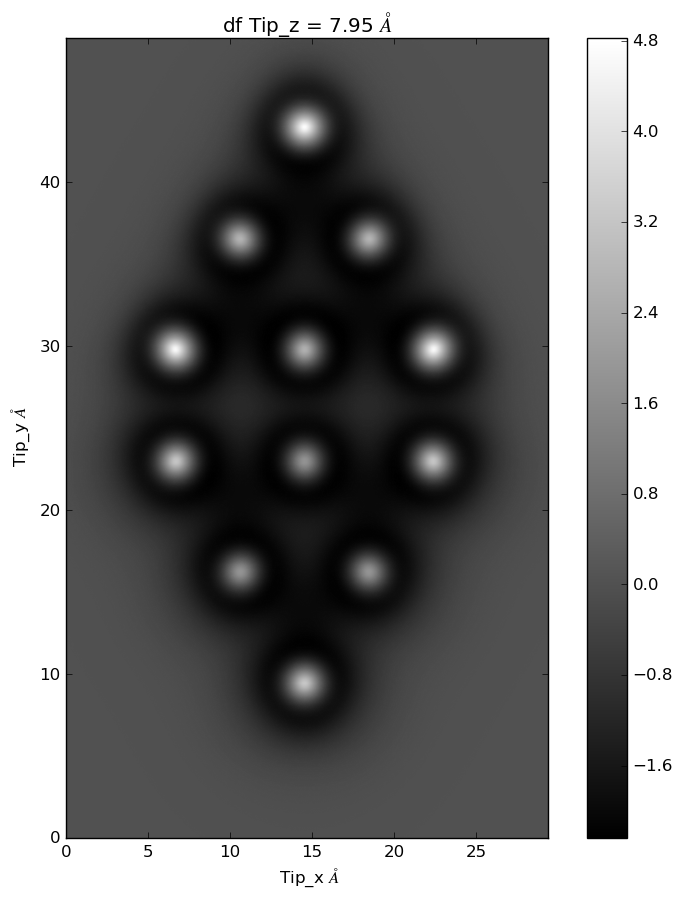

Supplement: File 7 — Datasets A0=1A k=0.5_extendedrange. [file Beilstein_J_Nanotechnol-07-937-s007.zip › S7/A0=1A/k=0.5_extendedrange/results/df_0161.png]

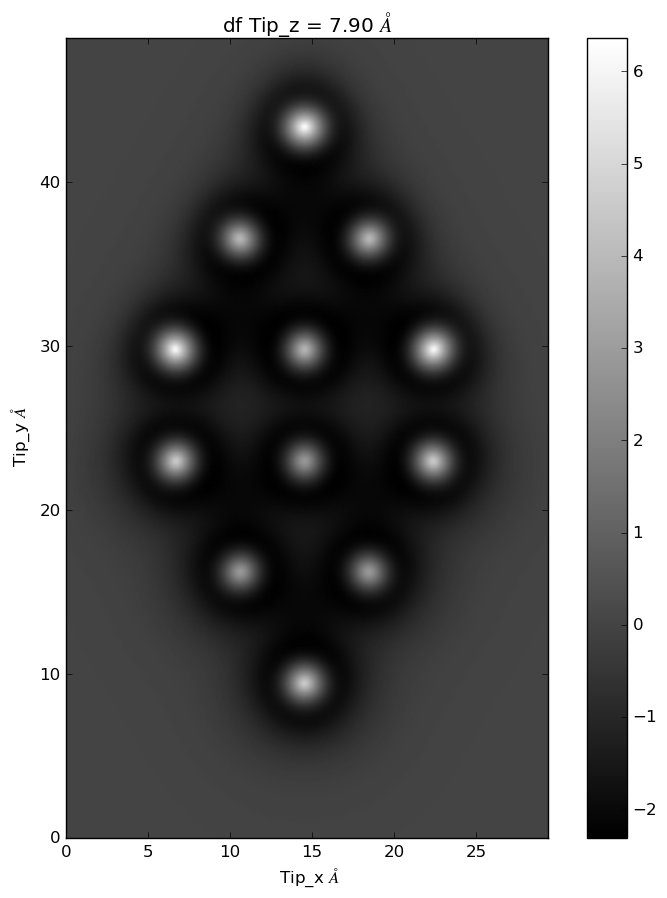

Supplement: File 7 — Datasets A0=1A k=0.5_extendedrange. [file Beilstein_J_Nanotechnol-07-937-s007.zip › S7/A0=1A/k=0.5_extendedrange/results/df_0162.png]

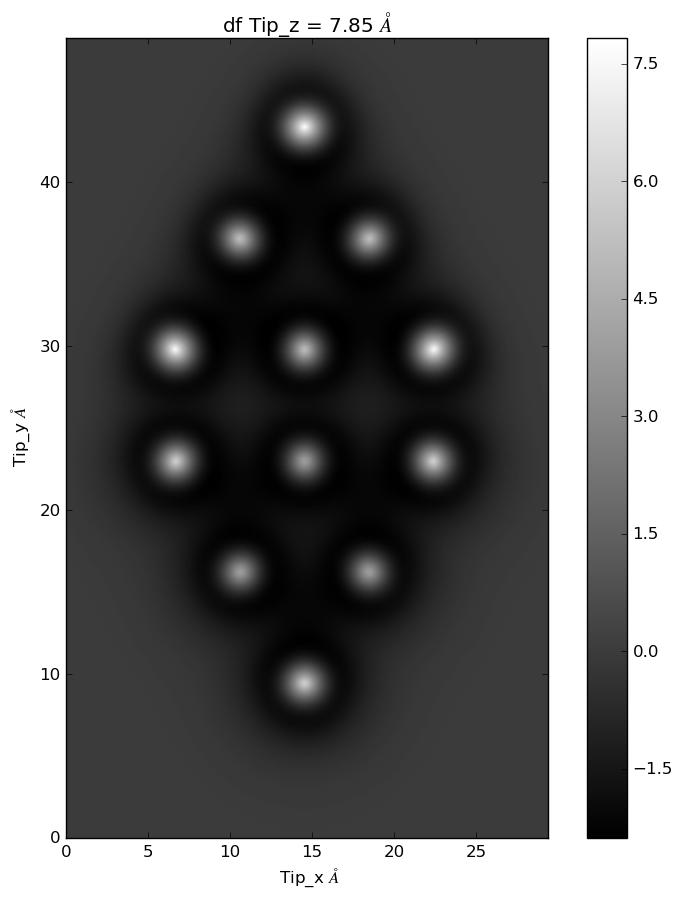

Supplement: File 7 — Datasets A0=1A k=0.5_extendedrange. [file Beilstein_J_Nanotechnol-07-937-s007.zip › S7/A0=1A/k=0.5_extendedrange/results/df_0163.png]

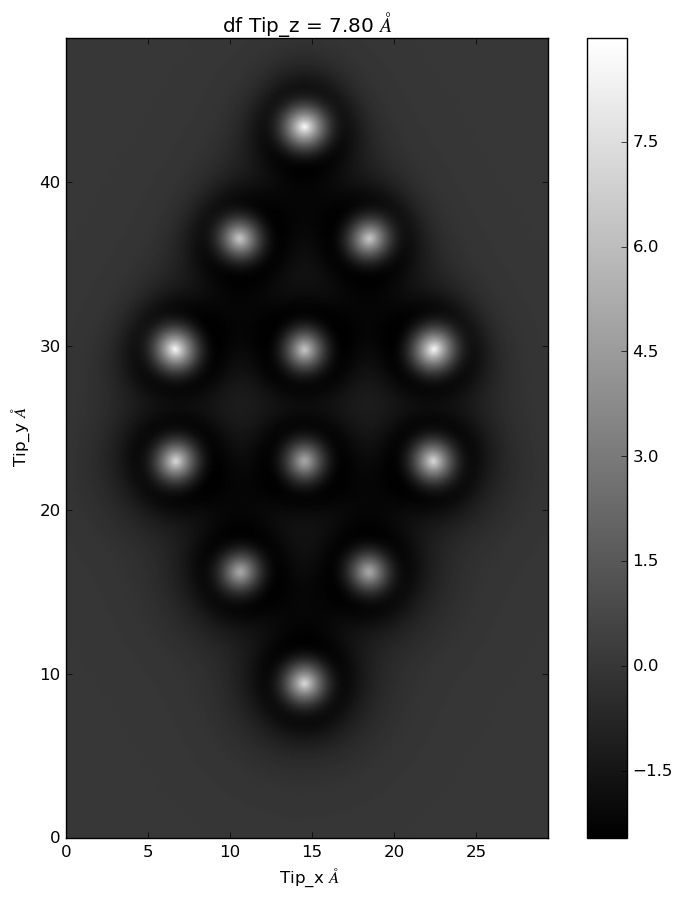

Supplement: File 7 — Datasets A0=1A k=0.5_extendedrange. [file Beilstein_J_Nanotechnol-07-937-s007.zip › S7/A0=1A/k=0.5_extendedrange/results/df_0164.png]

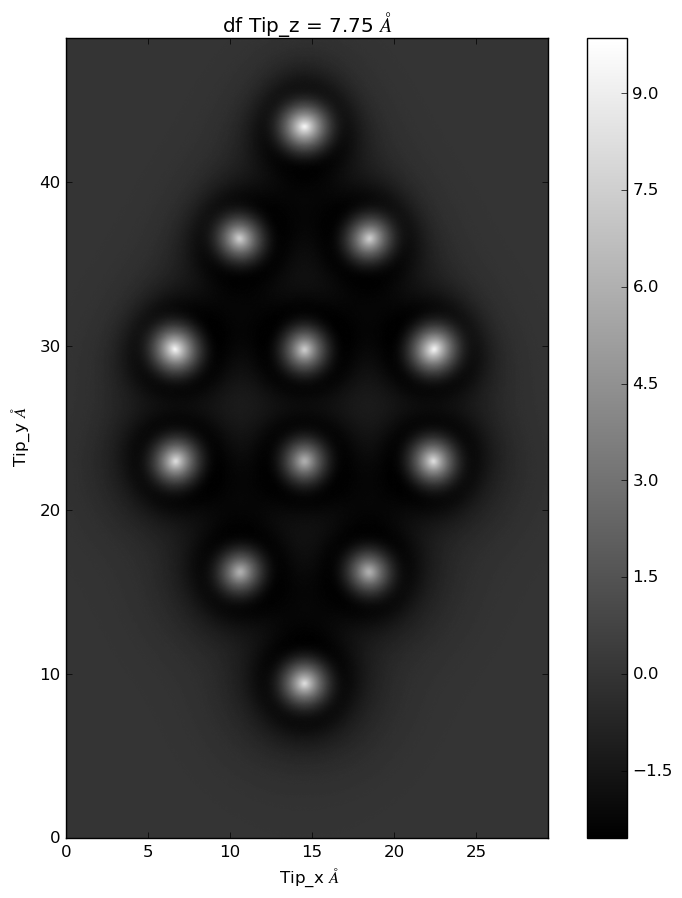

Supplement: File 7 — Datasets A0=1A k=0.5_extendedrange. [file Beilstein_J_Nanotechnol-07-937-s007.zip › S7/A0=1A/k=0.5_extendedrange/results/df_0165.png]

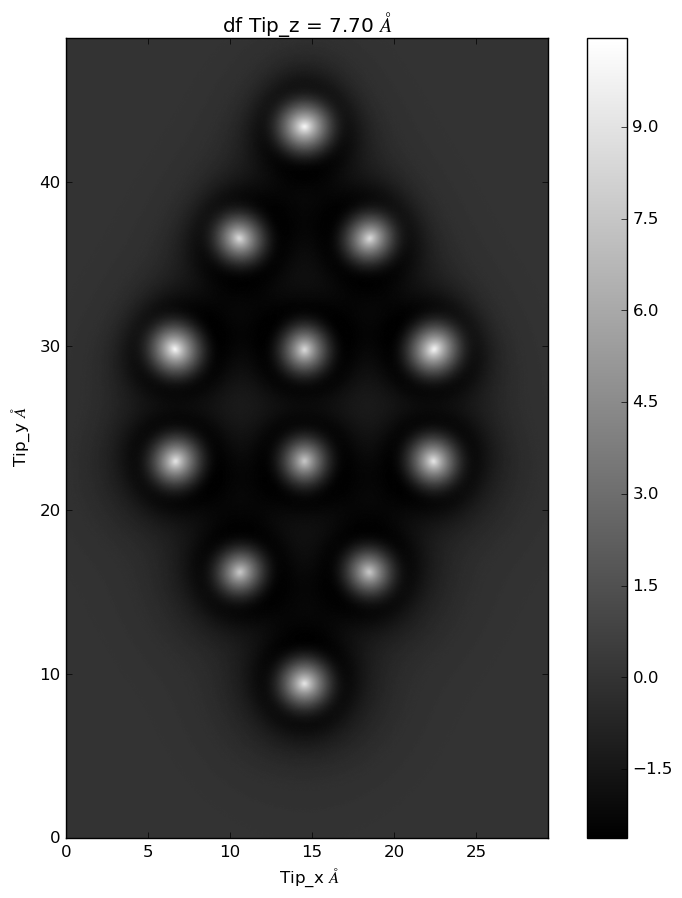

Supplement: File 7 — Datasets A0=1A k=0.5_extendedrange. [file Beilstein_J_Nanotechnol-07-937-s007.zip › S7/A0=1A/k=0.5_extendedrange/results/df_0166.png]

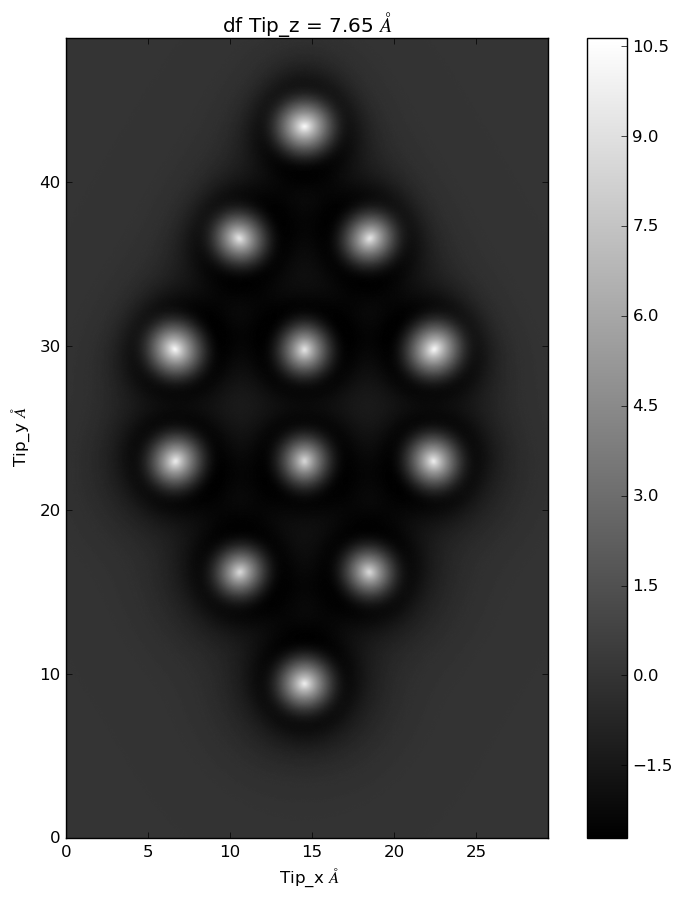

Supplement: File 7 — Datasets A0=1A k=0.5_extendedrange. [file Beilstein_J_Nanotechnol-07-937-s007.zip › S7/A0=1A/k=0.5_extendedrange/results/df_0167.png]

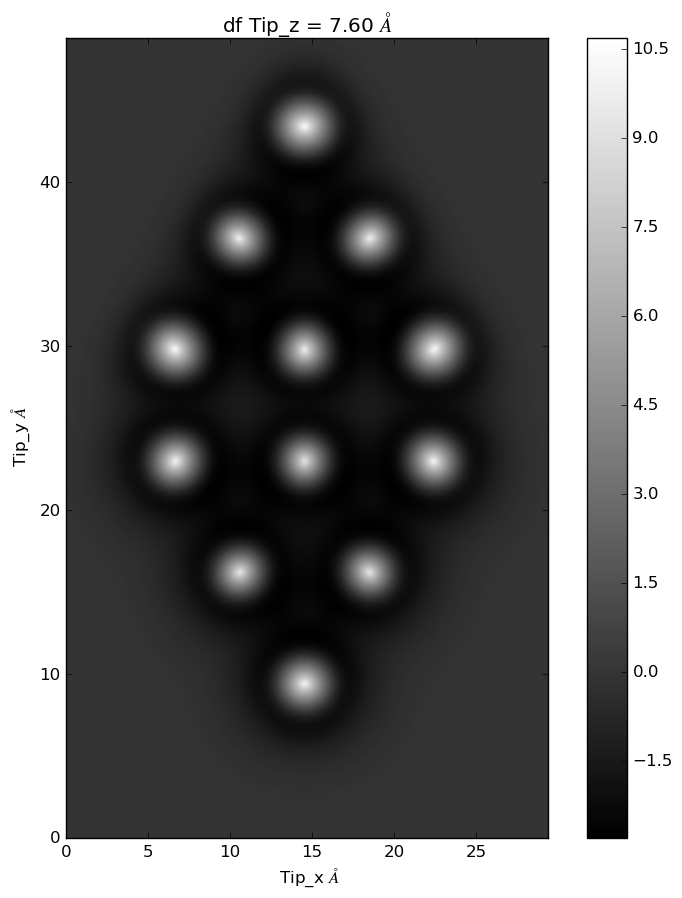

Supplement: File 7 — Datasets A0=1A k=0.5_extendedrange. [file Beilstein_J_Nanotechnol-07-937-s007.zip › S7/A0=1A/k=0.5_extendedrange/results/df_0168.png]

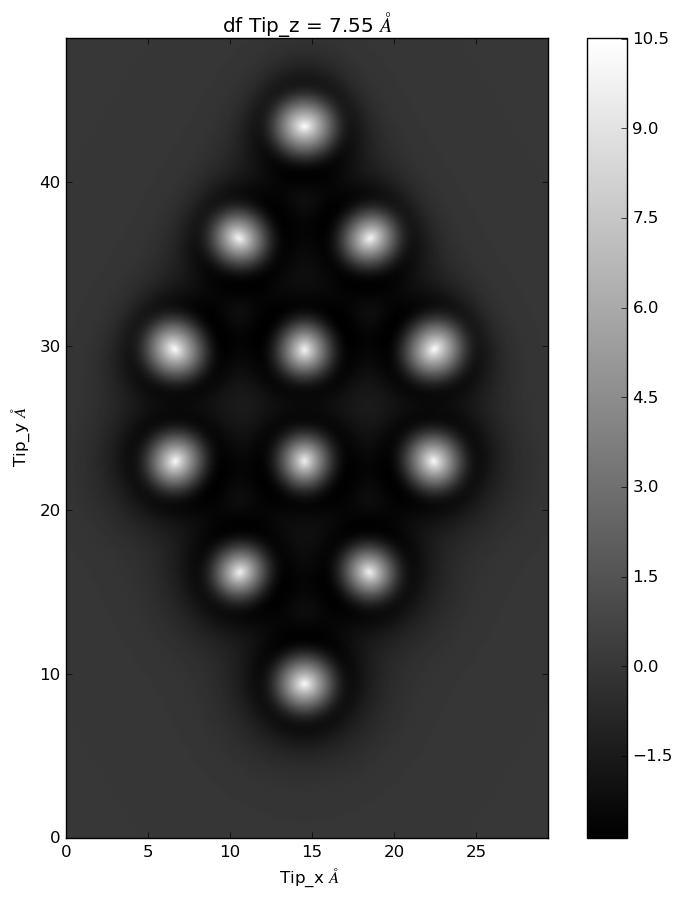

Supplement: File 7 — Datasets A0=1A k=0.5_extendedrange. [file Beilstein_J_Nanotechnol-07-937-s007.zip › S7/A0=1A/k=0.5_extendedrange/results/df_0169.png]

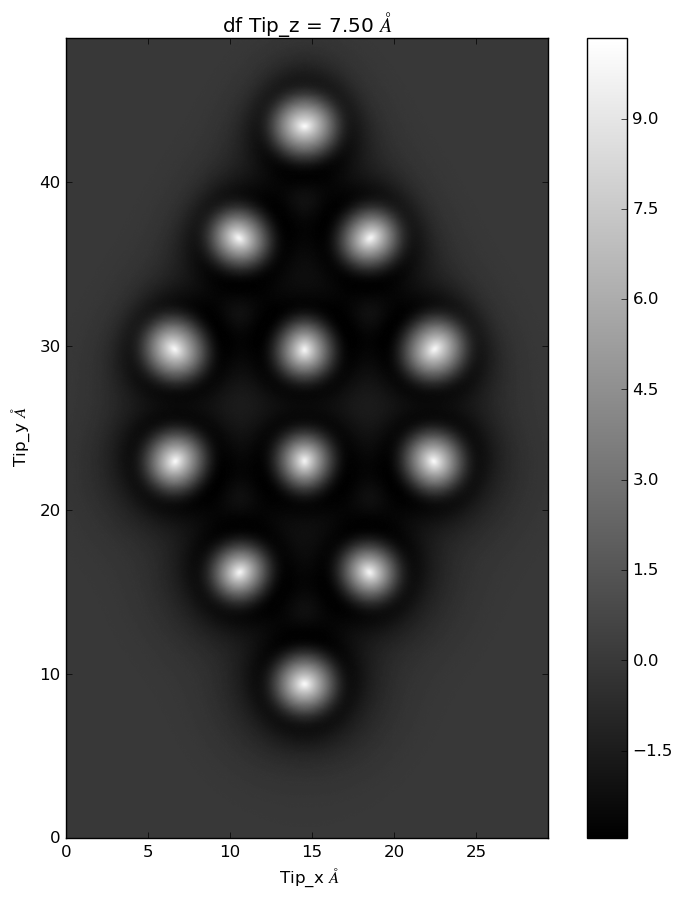

Supplement: File 7 — Datasets A0=1A k=0.5_extendedrange. [file Beilstein_J_Nanotechnol-07-937-s007.zip › S7/A0=1A/k=0.5_extendedrange/results/df_0170.png]

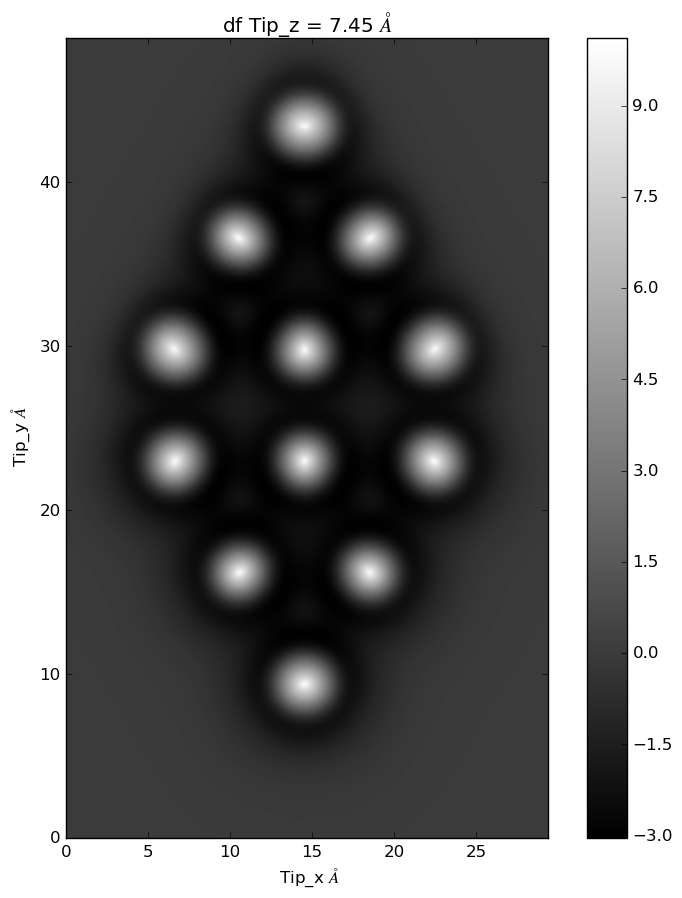

Supplement: File 7 — Datasets A0=1A k=0.5_extendedrange. [file Beilstein_J_Nanotechnol-07-937-s007.zip › S7/A0=1A/k=0.5_extendedrange/results/df_0171.png]

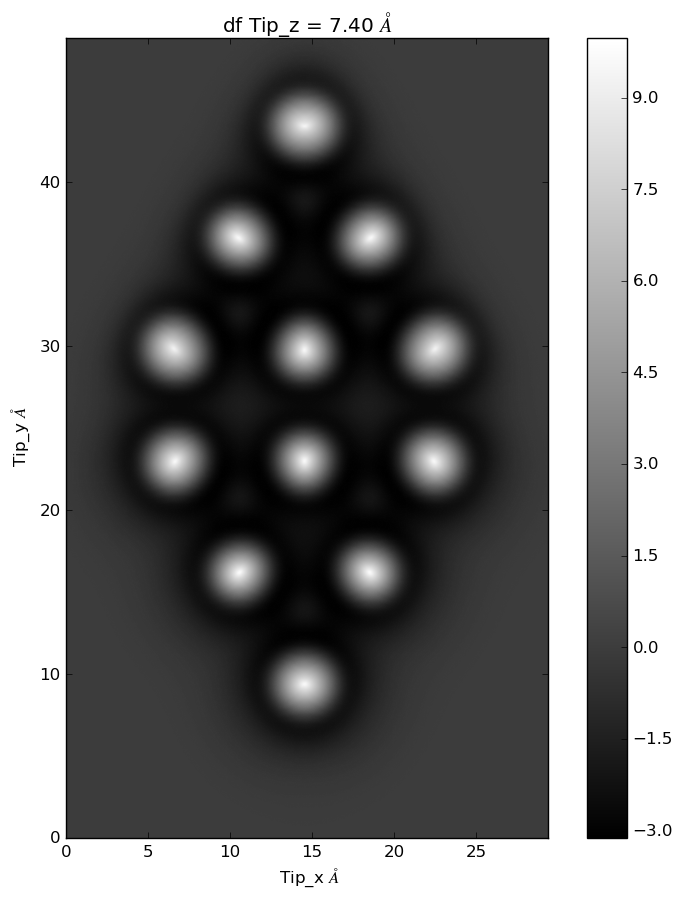

Supplement: File 7 — Datasets A0=1A k=0.5_extendedrange. [file Beilstein_J_Nanotechnol-07-937-s007.zip › S7/A0=1A/k=0.5_extendedrange/results/df_0172.png]

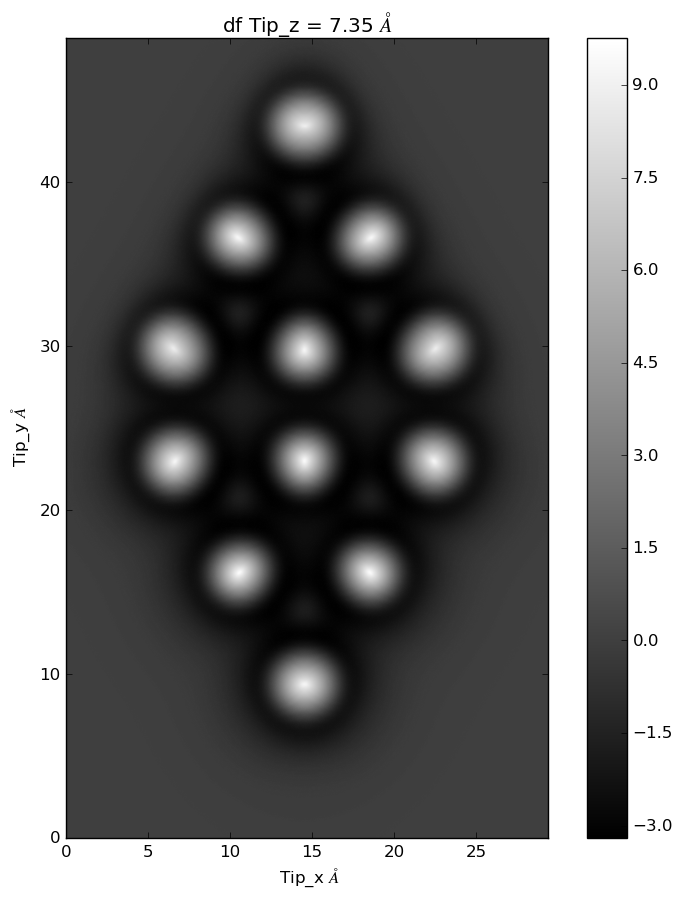

Supplement: File 7 — Datasets A0=1A k=0.5_extendedrange. [file Beilstein_J_Nanotechnol-07-937-s007.zip › S7/A0=1A/k=0.5_extendedrange/results/df_0173.png]

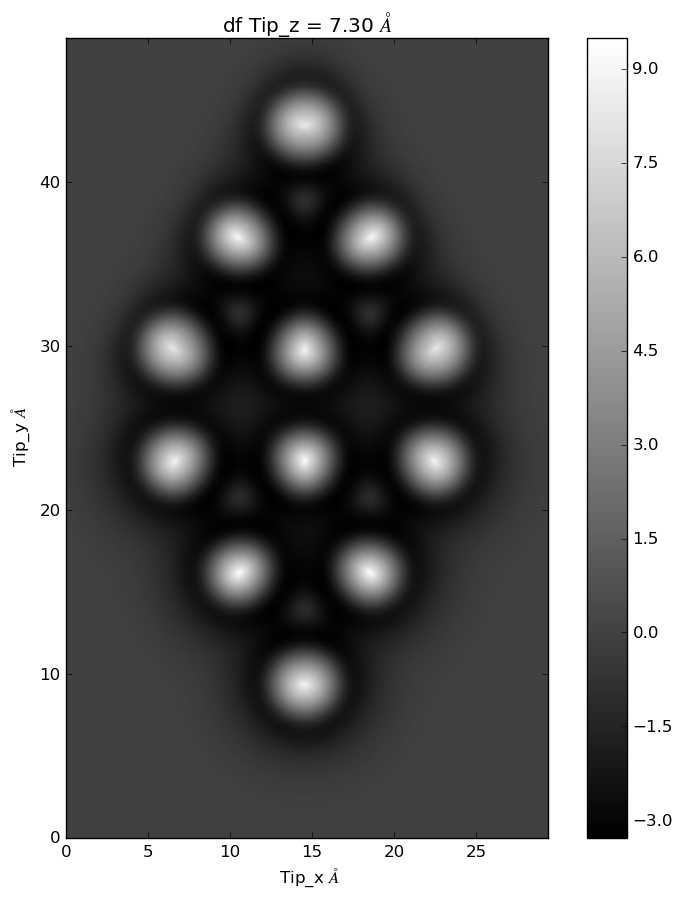

Supplement: File 7 — Datasets A0=1A k=0.5_extendedrange. [file Beilstein_J_Nanotechnol-07-937-s007.zip › S7/A0=1A/k=0.5_extendedrange/results/df_0174.png]

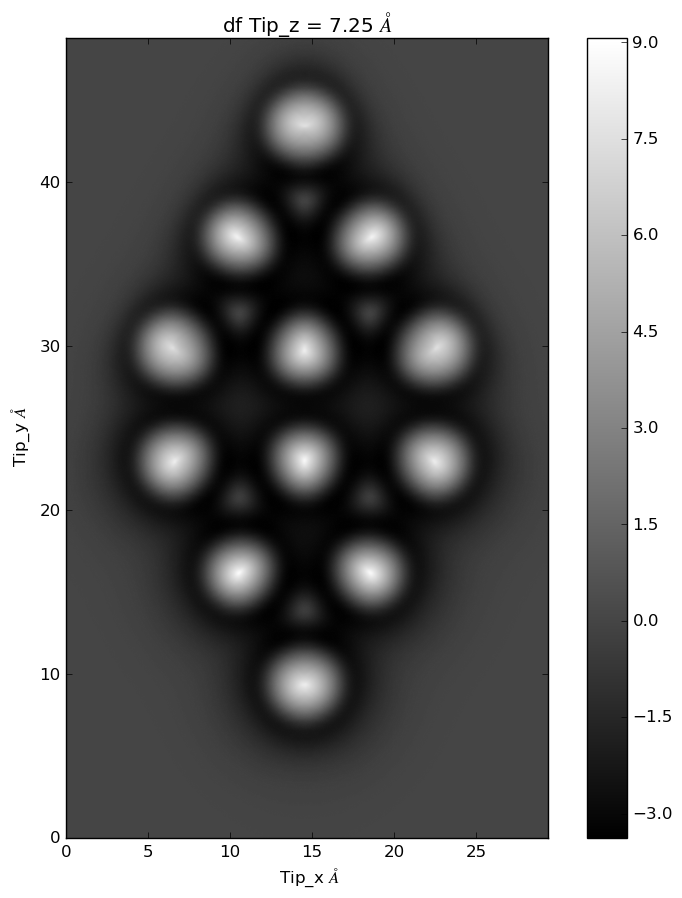

Supplement: File 7 — Datasets A0=1A k=0.5_extendedrange. [file Beilstein_J_Nanotechnol-07-937-s007.zip › S7/A0=1A/k=0.5_extendedrange/results/df_0175.png]

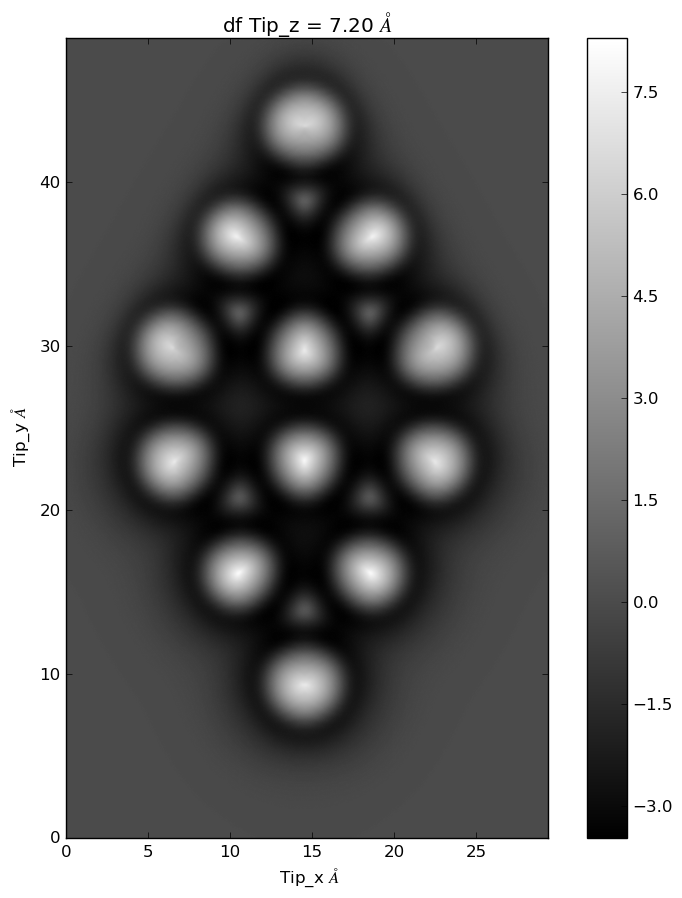

Supplement: File 7 — Datasets A0=1A k=0.5_extendedrange. [file Beilstein_J_Nanotechnol-07-937-s007.zip › S7/A0=1A/k=0.5_extendedrange/results/df_0176.png]

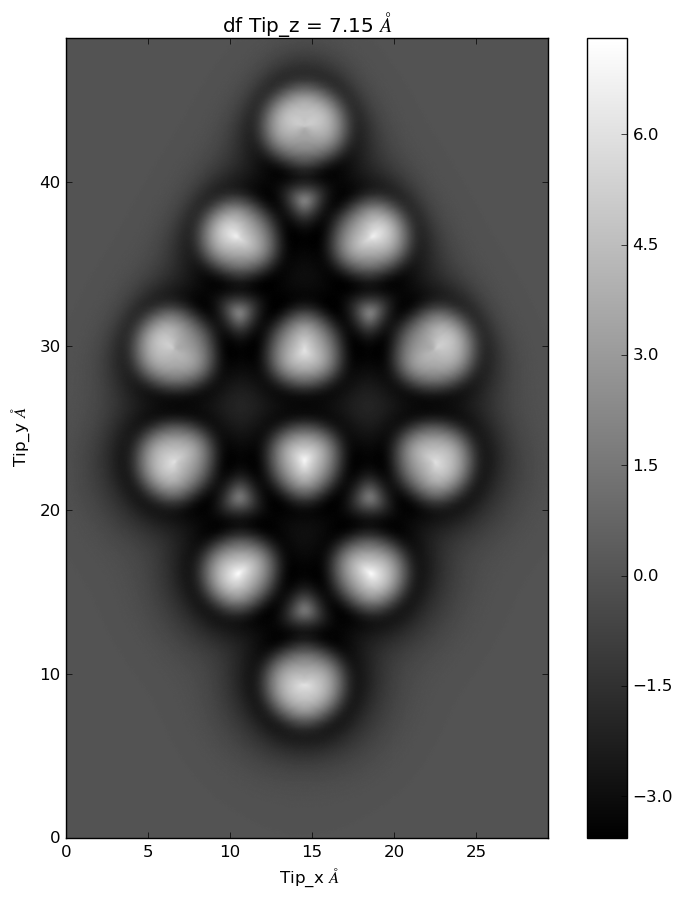

Supplement: File 7 — Datasets A0=1A k=0.5_extendedrange. [file Beilstein_J_Nanotechnol-07-937-s007.zip › S7/A0=1A/k=0.5_extendedrange/results/df_0177.png]

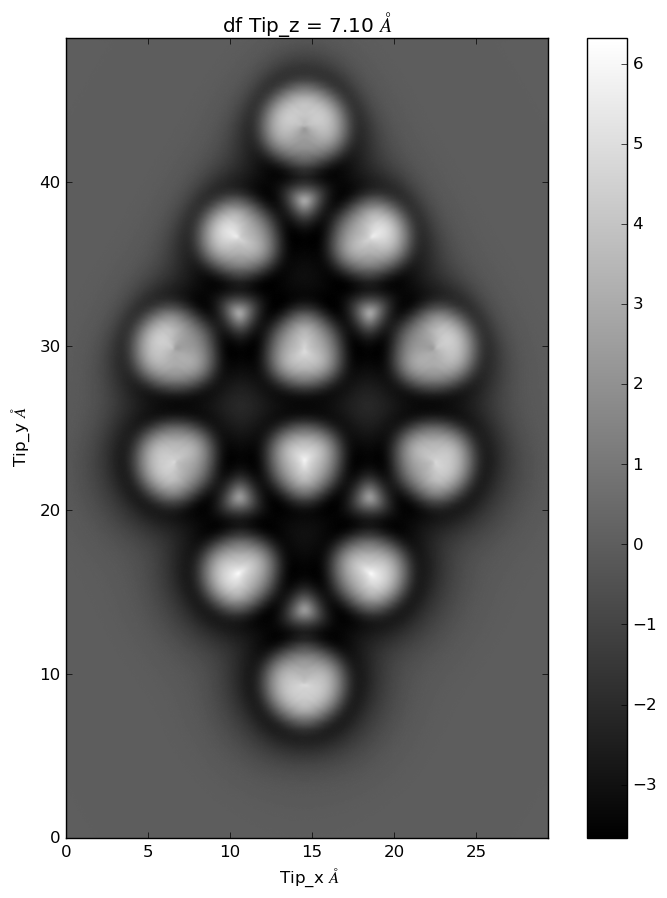

Supplement: File 7 — Datasets A0=1A k=0.5_extendedrange. [file Beilstein_J_Nanotechnol-07-937-s007.zip › S7/A0=1A/k=0.5_extendedrange/results/df_0178.png]

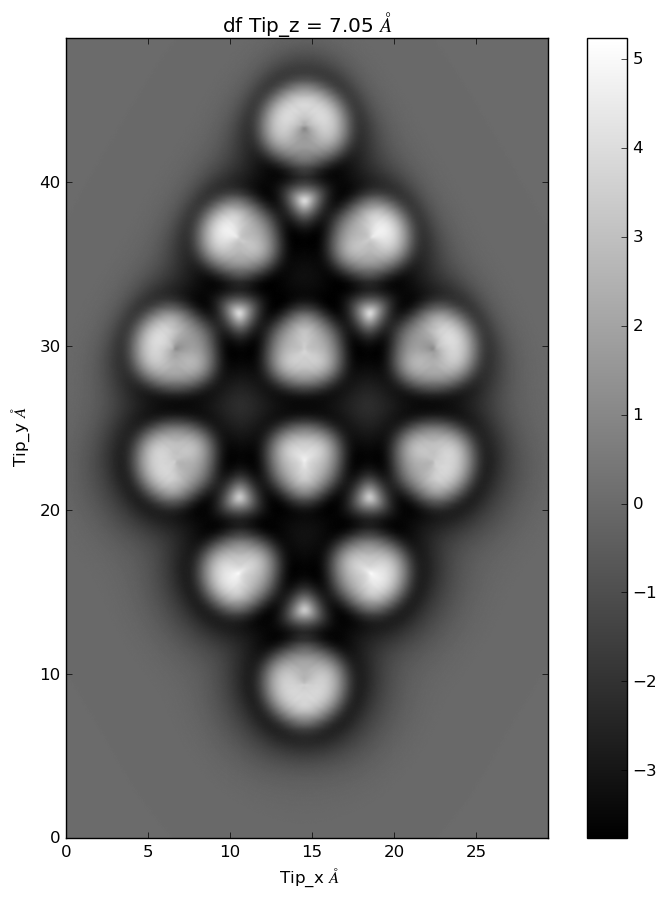

Supplement: File 7 — Datasets A0=1A k=0.5_extendedrange. [file Beilstein_J_Nanotechnol-07-937-s007.zip › S7/A0=1A/k=0.5_extendedrange/results/df_0179.png]

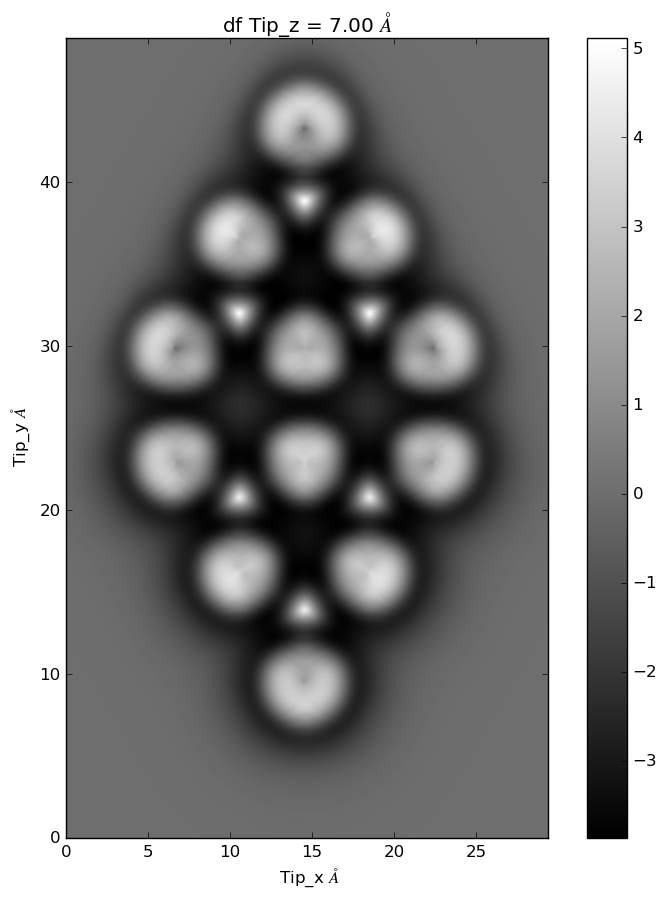

Supplement: File 7 — Datasets A0=1A k=0.5_extendedrange. [file Beilstein_J_Nanotechnol-07-937-s007.zip › S7/A0=1A/k=0.5_extendedrange/results/df_0180.png]

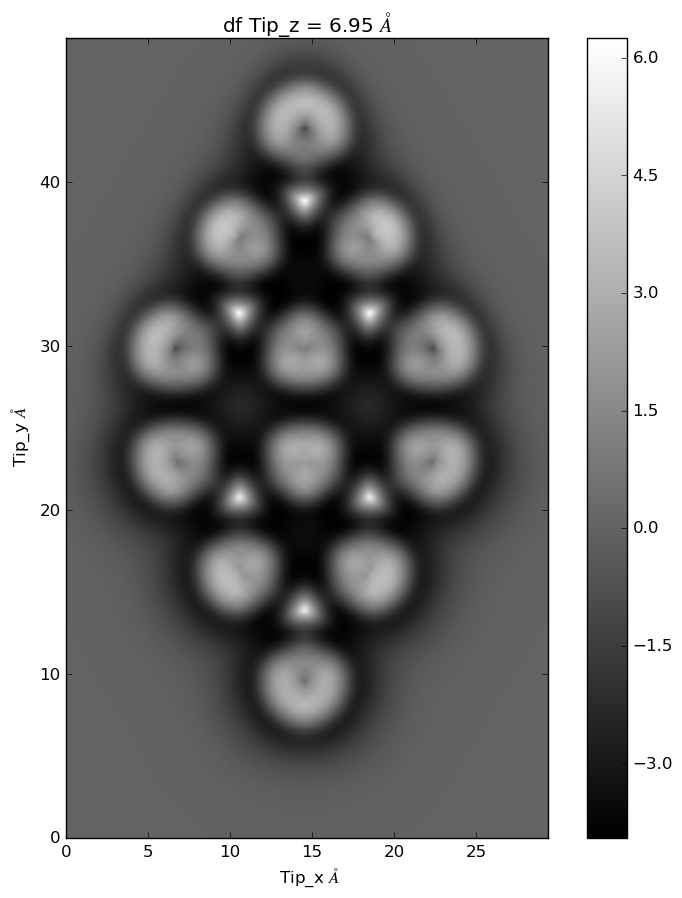

Supplement: File 7 — Datasets A0=1A k=0.5_extendedrange. [file Beilstein_J_Nanotechnol-07-937-s007.zip › S7/A0=1A/k=0.5_extendedrange/results/df_0181.png]

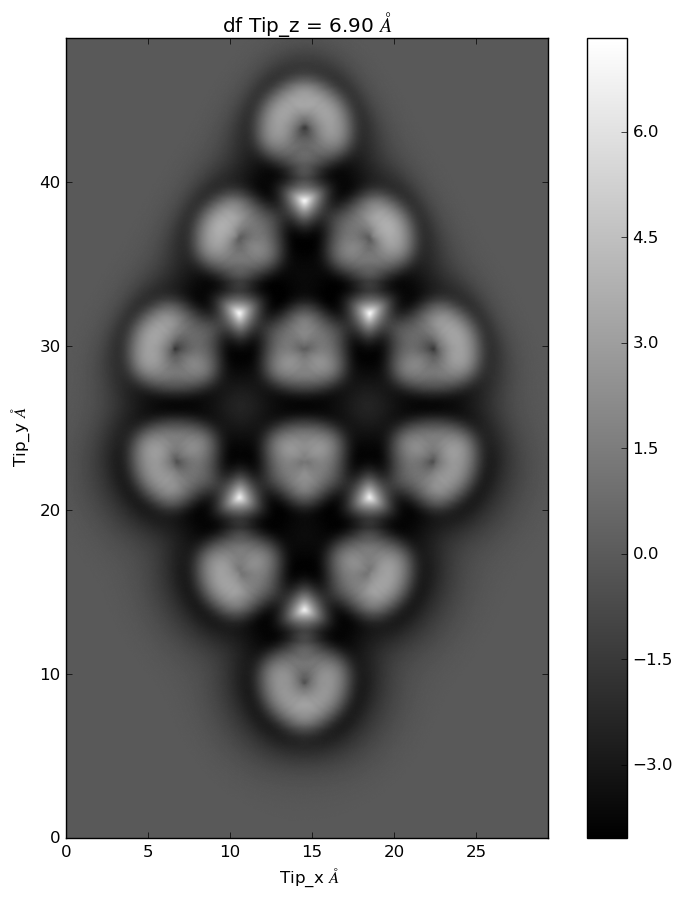

Supplement: File 7 — Datasets A0=1A k=0.5_extendedrange. [file Beilstein_J_Nanotechnol-07-937-s007.zip › S7/A0=1A/k=0.5_extendedrange/results/df_0182.png]

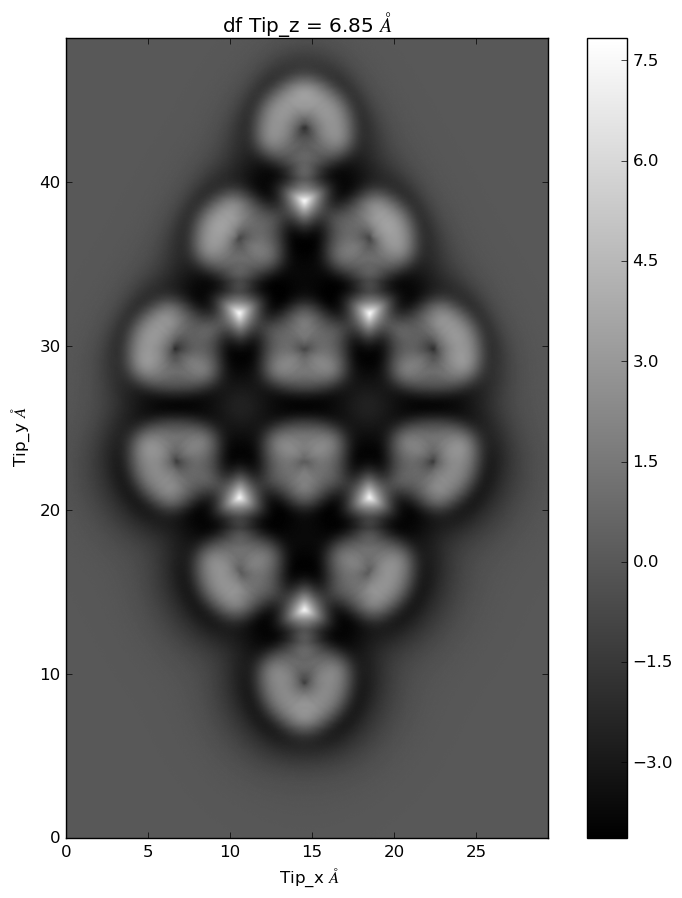

Supplement: File 7 — Datasets A0=1A k=0.5_extendedrange. [file Beilstein_J_Nanotechnol-07-937-s007.zip › S7/A0=1A/k=0.5_extendedrange/results/df_0183.png]

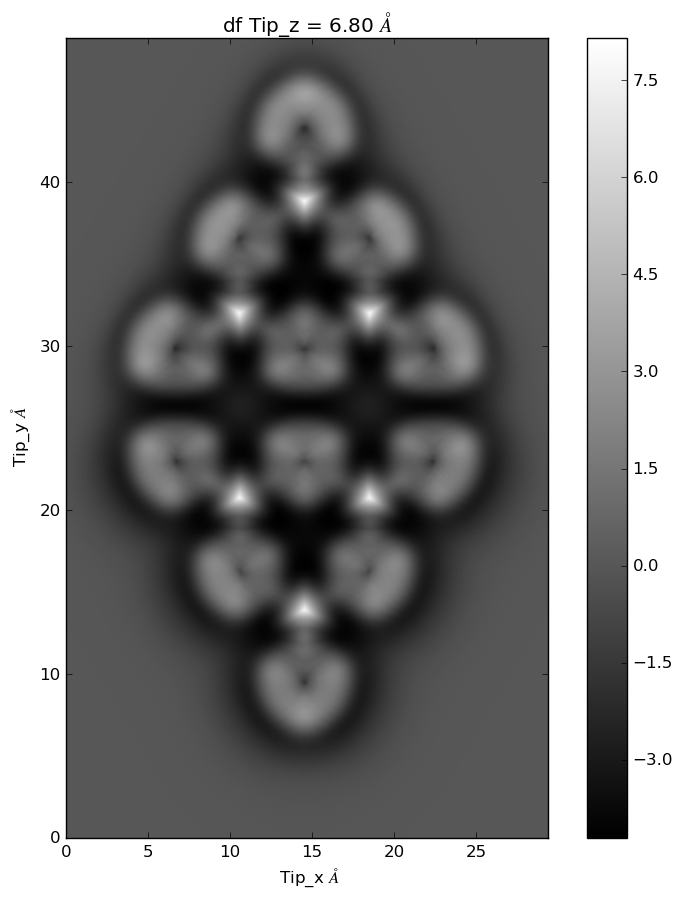

Supplement: File 7 — Datasets A0=1A k=0.5_extendedrange. [file Beilstein_J_Nanotechnol-07-937-s007.zip › S7/A0=1A/k=0.5_extendedrange/results/df_0184.png]

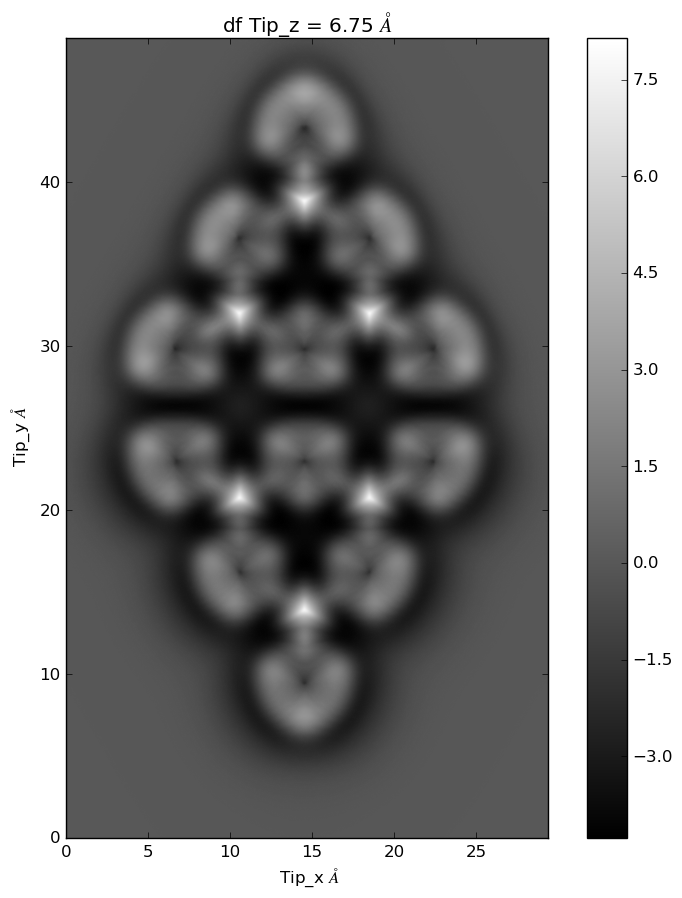

Supplement: File 7 — Datasets A0=1A k=0.5_extendedrange. [file Beilstein_J_Nanotechnol-07-937-s007.zip › S7/A0=1A/k=0.5_extendedrange/results/df_0185.png]

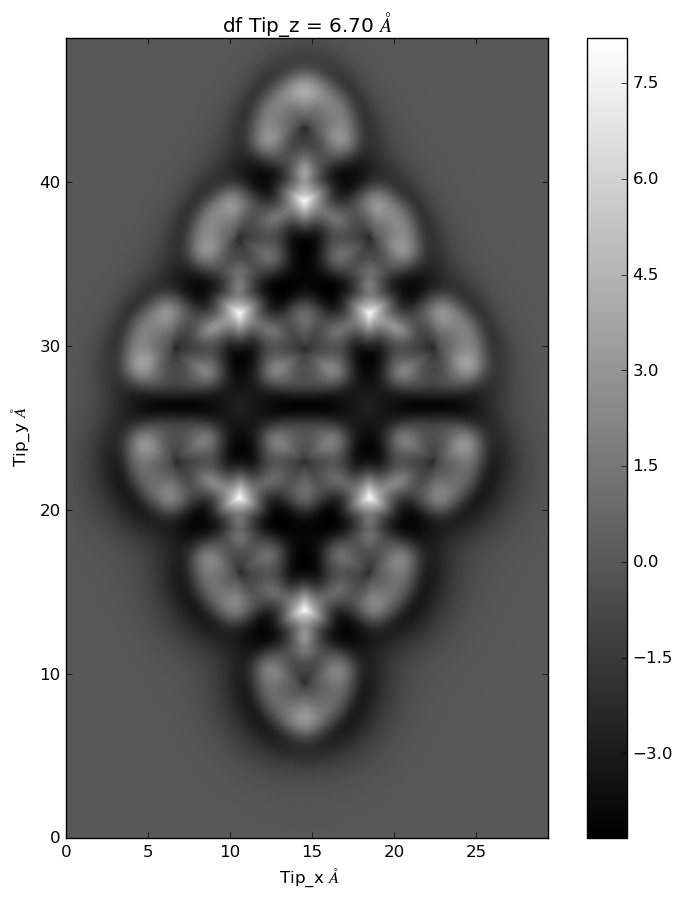

Supplement: File 7 — Datasets A0=1A k=0.5_extendedrange. [file Beilstein_J_Nanotechnol-07-937-s007.zip › S7/A0=1A/k=0.5_extendedrange/results/df_0186.png]

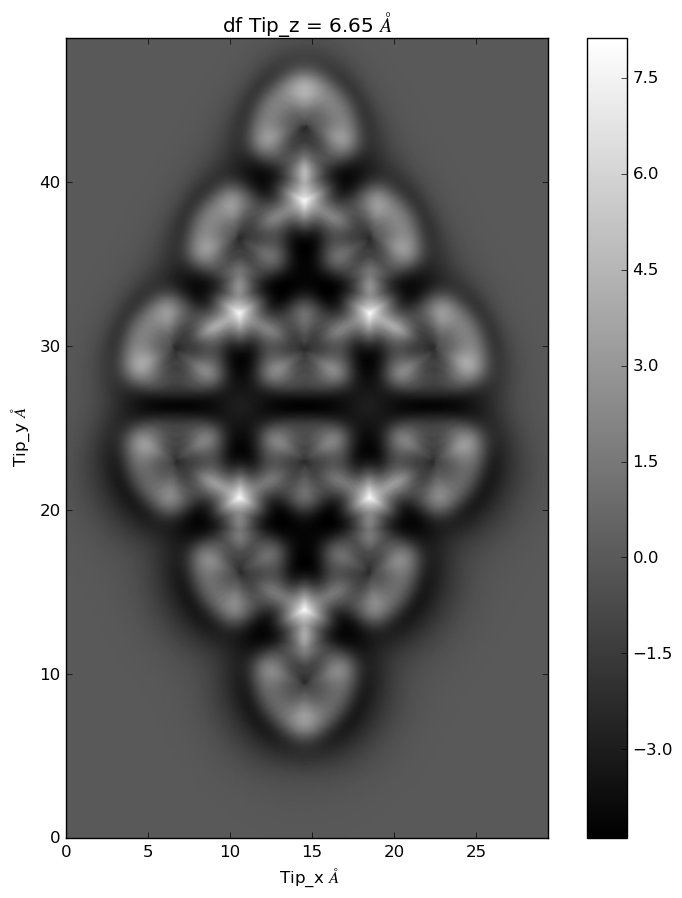

Supplement: File 7 — Datasets A0=1A k=0.5_extendedrange. [file Beilstein_J_Nanotechnol-07-937-s007.zip › S7/A0=1A/k=0.5_extendedrange/results/df_0187.png]

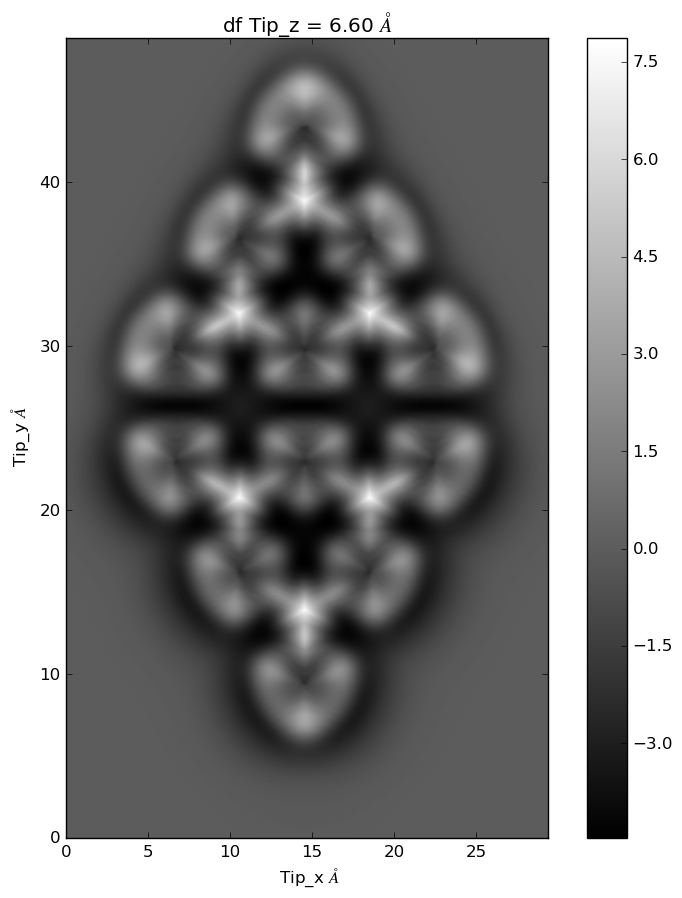

Supplement: File 7 — Datasets A0=1A k=0.5_extendedrange. [file Beilstein_J_Nanotechnol-07-937-s007.zip › S7/A0=1A/k=0.5_extendedrange/results/df_0188.png]

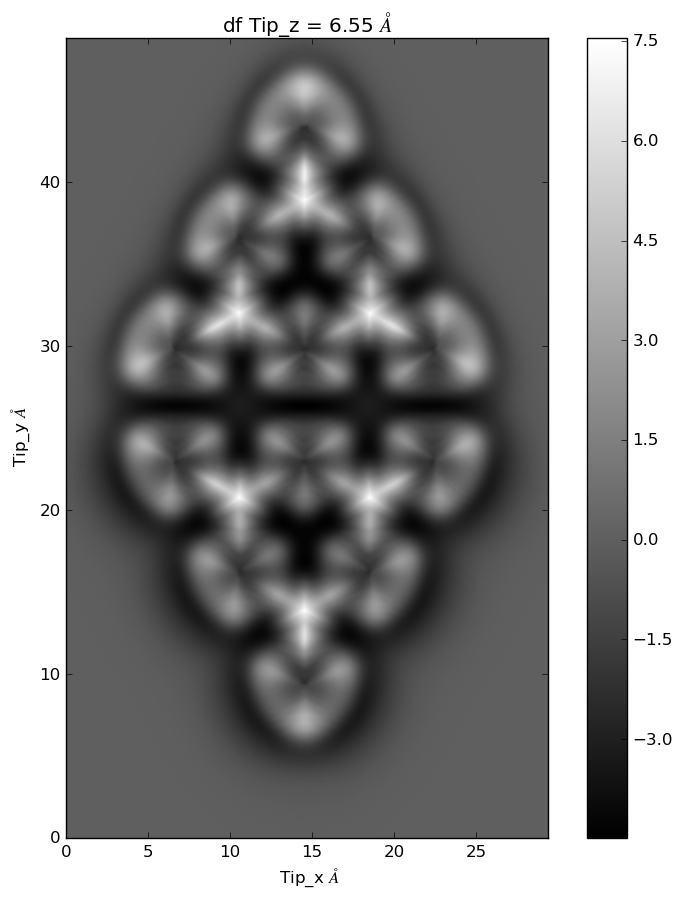

Supplement: File 7 — Datasets A0=1A k=0.5_extendedrange. [file Beilstein_J_Nanotechnol-07-937-s007.zip › S7/A0=1A/k=0.5_extendedrange/results/df_0189.png]

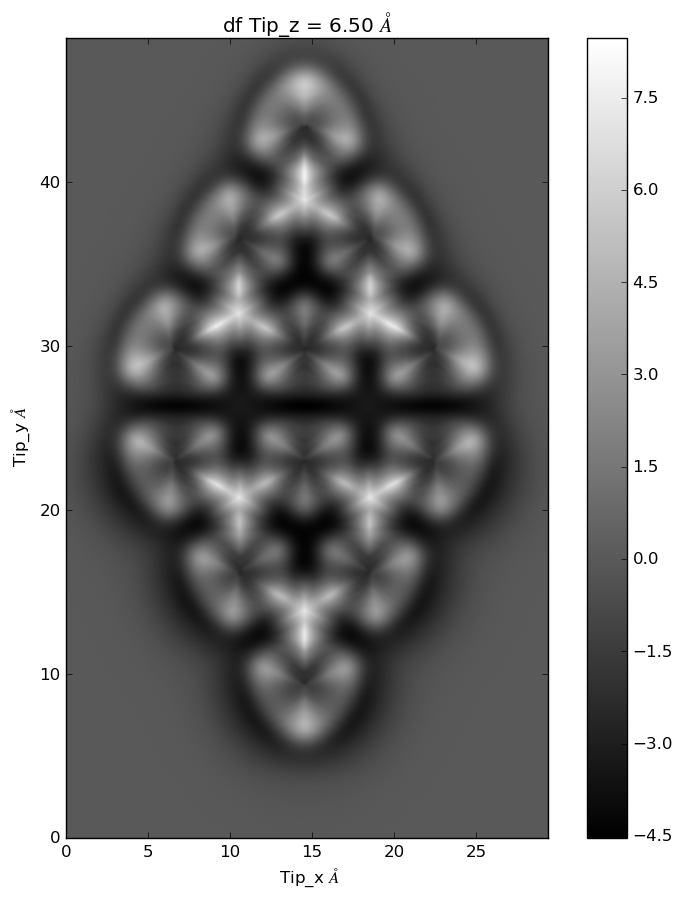

Supplement: File 7 — Datasets A0=1A k=0.5_extendedrange. [file Beilstein_J_Nanotechnol-07-937-s007.zip › S7/A0=1A/k=0.5_extendedrange/results/df_0190.png]

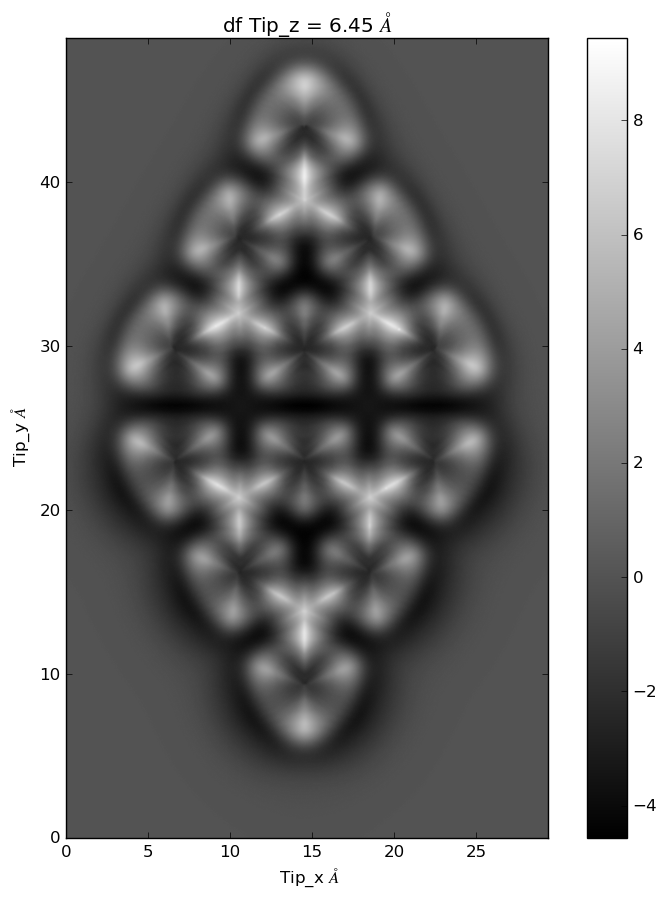

Supplement: File 7 — Datasets A0=1A k=0.5_extendedrange. [file Beilstein_J_Nanotechnol-07-937-s007.zip › S7/A0=1A/k=0.5_extendedrange/results/df_0191.png]

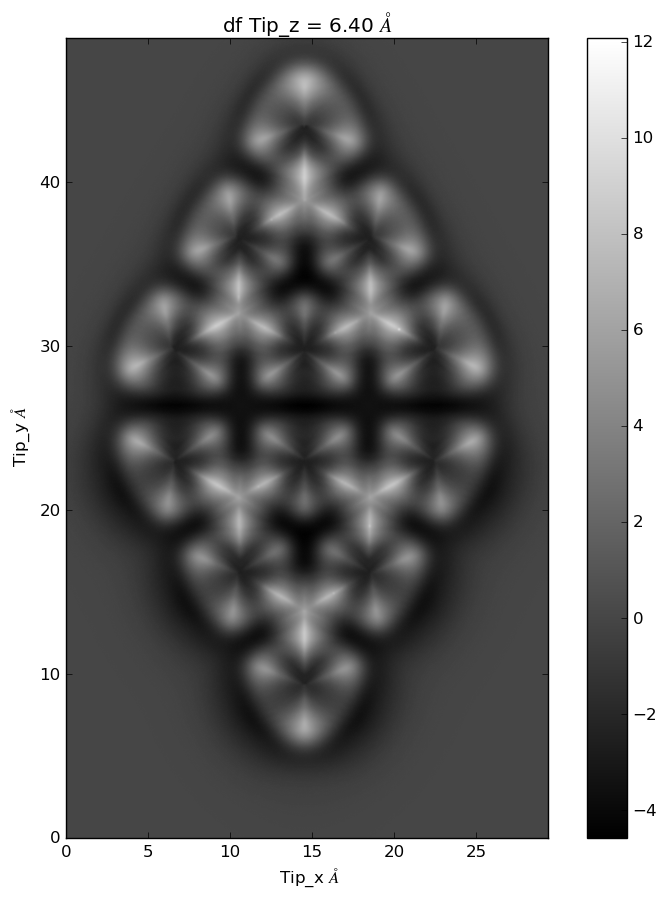

Supplement: File 7 — Datasets A0=1A k=0.5_extendedrange. [file Beilstein_J_Nanotechnol-07-937-s007.zip › S7/A0=1A/k=0.5_extendedrange/results/df_0192.png]

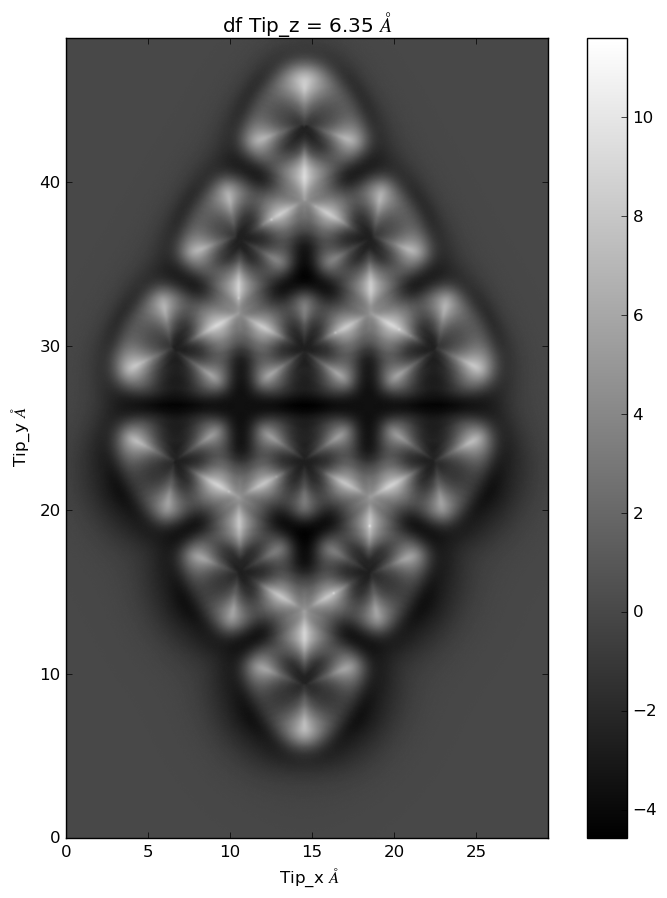

Supplement: File 7 — Datasets A0=1A k=0.5_extendedrange. [file Beilstein_J_Nanotechnol-07-937-s007.zip › S7/A0=1A/k=0.5_extendedrange/results/df_0193.png]

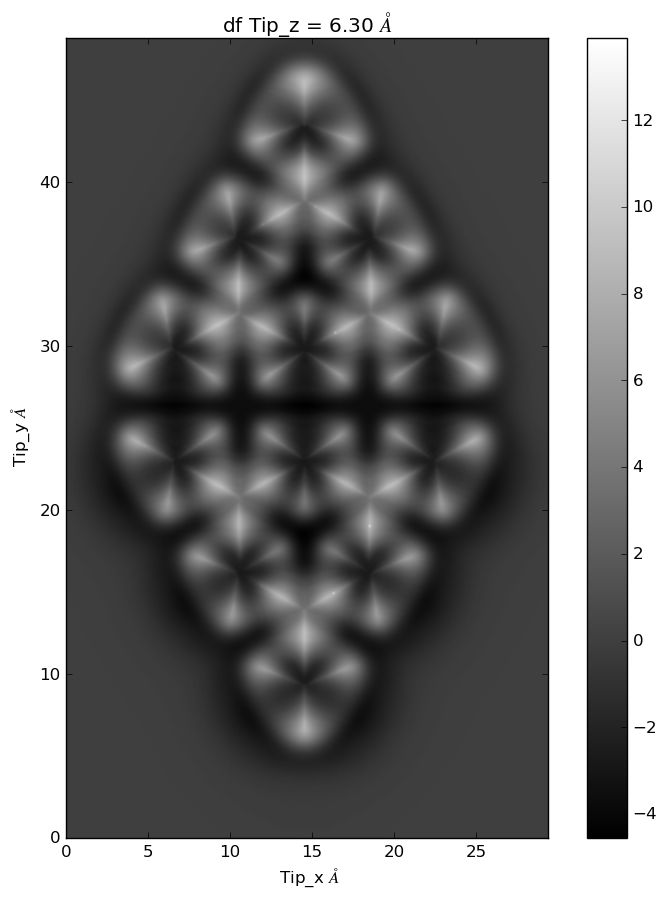

Supplement: File 7 — Datasets A0=1A k=0.5_extendedrange. [file Beilstein_J_Nanotechnol-07-937-s007.zip › S7/A0=1A/k=0.5_extendedrange/results/df_0194.png]

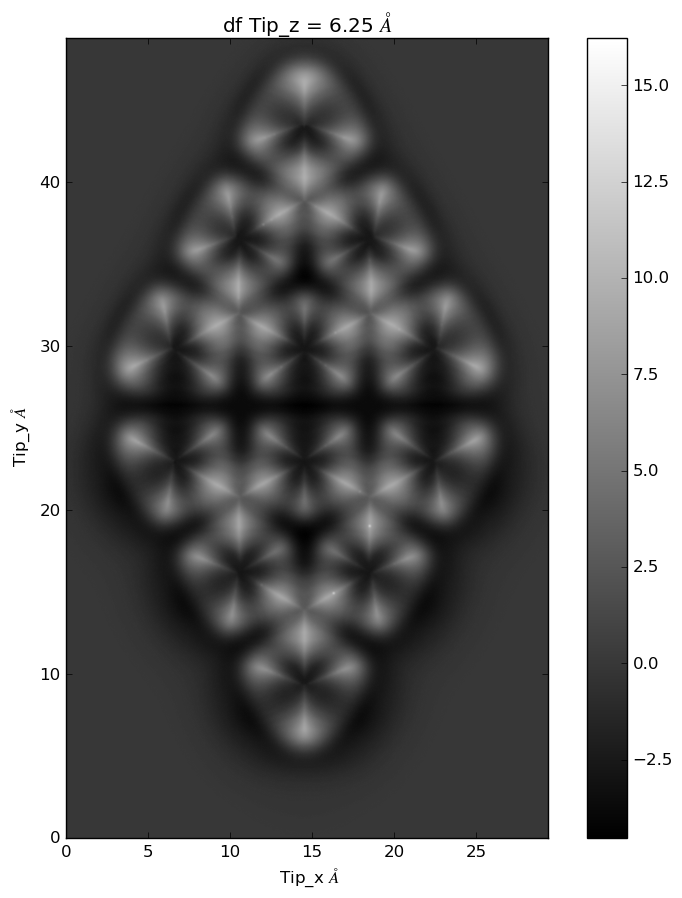

Supplement: File 7 — Datasets A0=1A k=0.5_extendedrange. [file Beilstein_J_Nanotechnol-07-937-s007.zip › S7/A0=1A/k=0.5_extendedrange/results/df_0195.png]

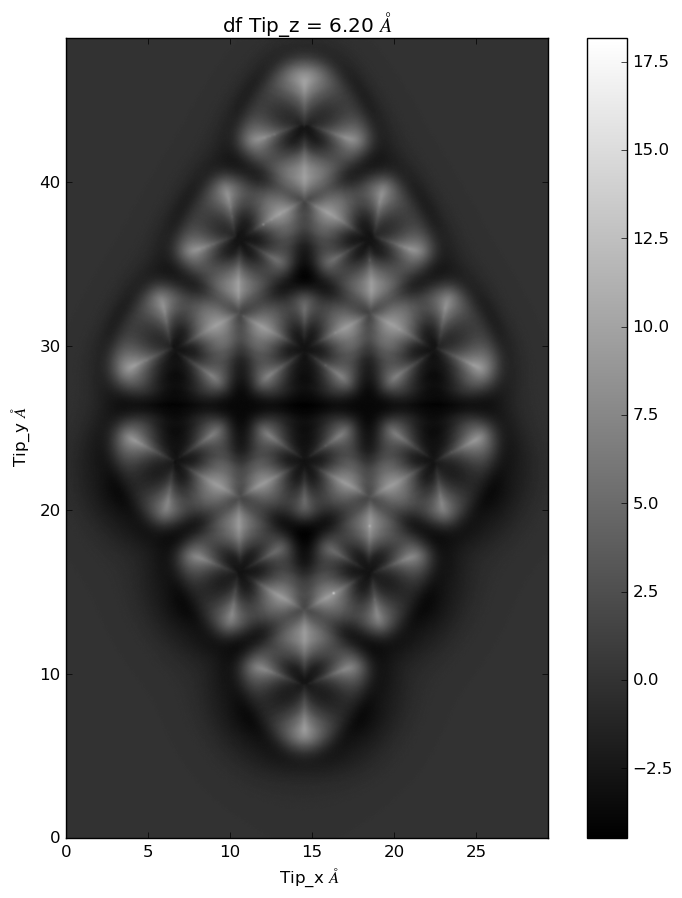

Supplement: File 7 — Datasets A0=1A k=0.5_extendedrange. [file Beilstein_J_Nanotechnol-07-937-s007.zip › S7/A0=1A/k=0.5_extendedrange/results/df_0196.png]

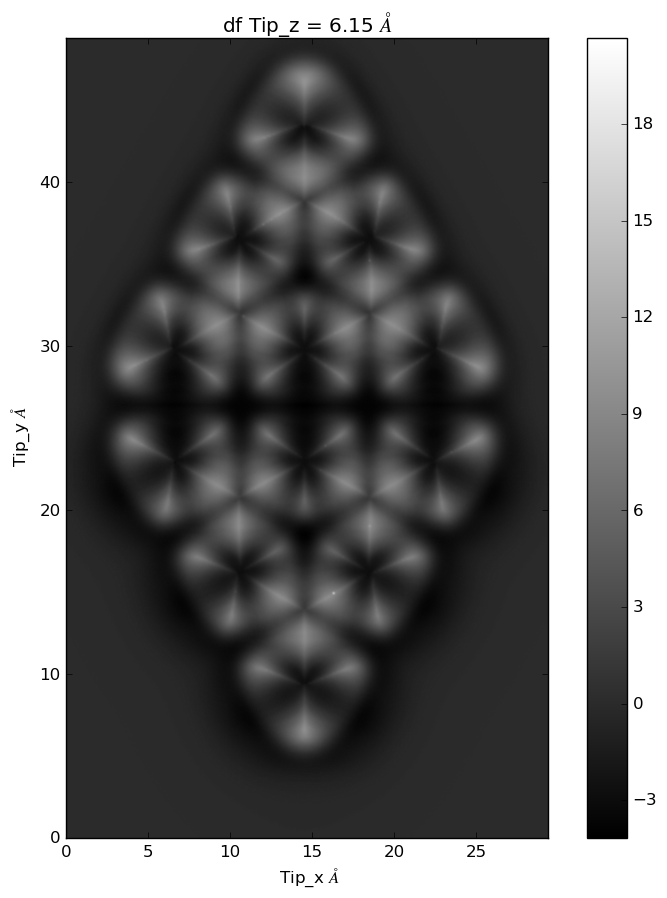

Supplement: File 7 — Datasets A0=1A k=0.5_extendedrange. [file Beilstein_J_Nanotechnol-07-937-s007.zip › S7/A0=1A/k=0.5_extendedrange/results/df_0197.png]

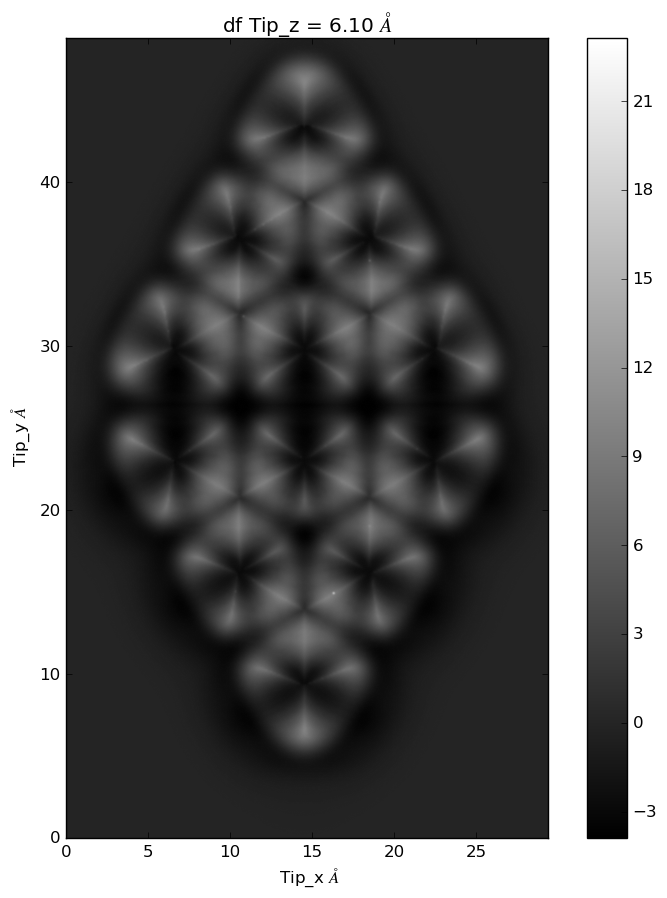

Supplement: File 7 — Datasets A0=1A k=0.5_extendedrange. [file Beilstein_J_Nanotechnol-07-937-s007.zip › S7/A0=1A/k=0.5_extendedrange/results/df_0198.png]

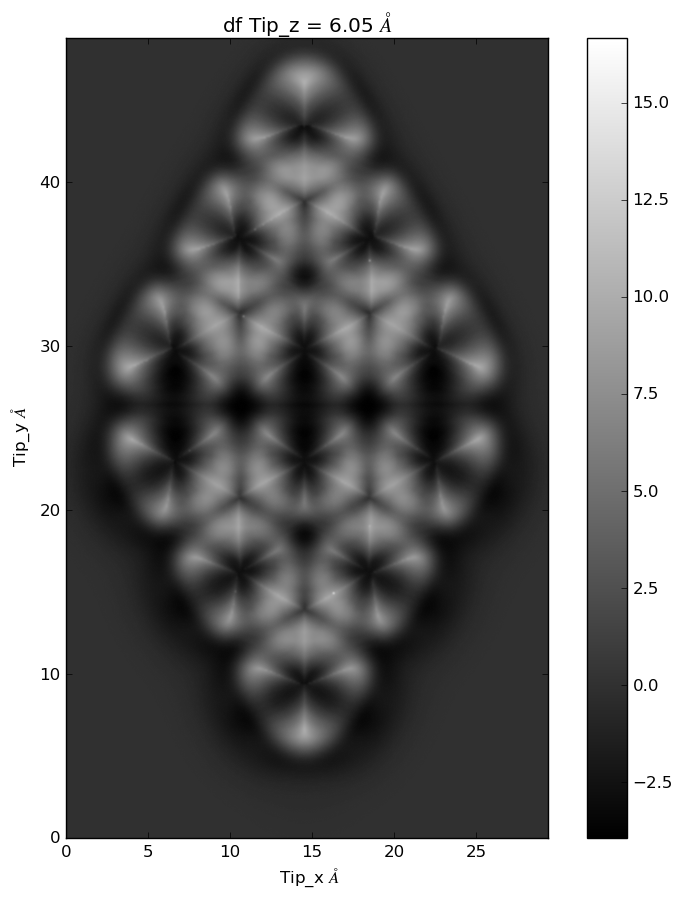

Supplement: File 7 — Datasets A0=1A k=0.5_extendedrange. [file Beilstein_J_Nanotechnol-07-937-s007.zip › S7/A0=1A/k=0.5_extendedrange/results/df_0199.png]
