# Supplementary figures and images for: Modelling of ‘sub-atomic’ contrast resulting from back-bonding on Si(111)-7×7 (part 18 of 18)
Source: Beilstein J Nanotechnol. 2016 Jun 29;7:937–45. doi: 10.3762/bjnano.7.85 (PMC4979881; doi:10.3762/bjnano.7.85)

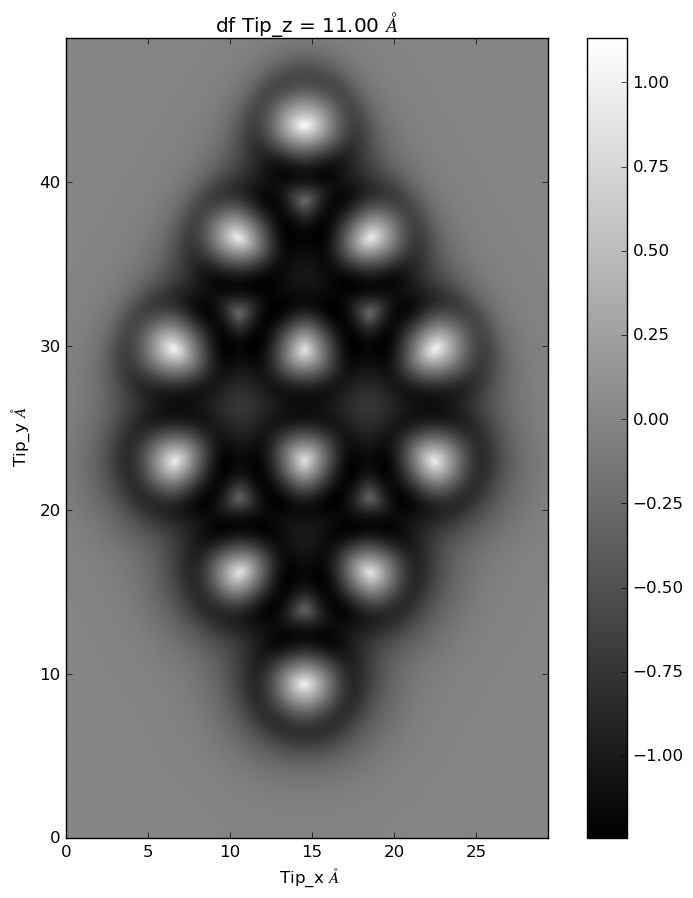

Supplement: File 9 — Datasets A0=5A k=0.5. [file Beilstein_J_Nanotechnol-07-937-s009.zip › S9/A0=5A/k=0.5/results/df_0100.png]

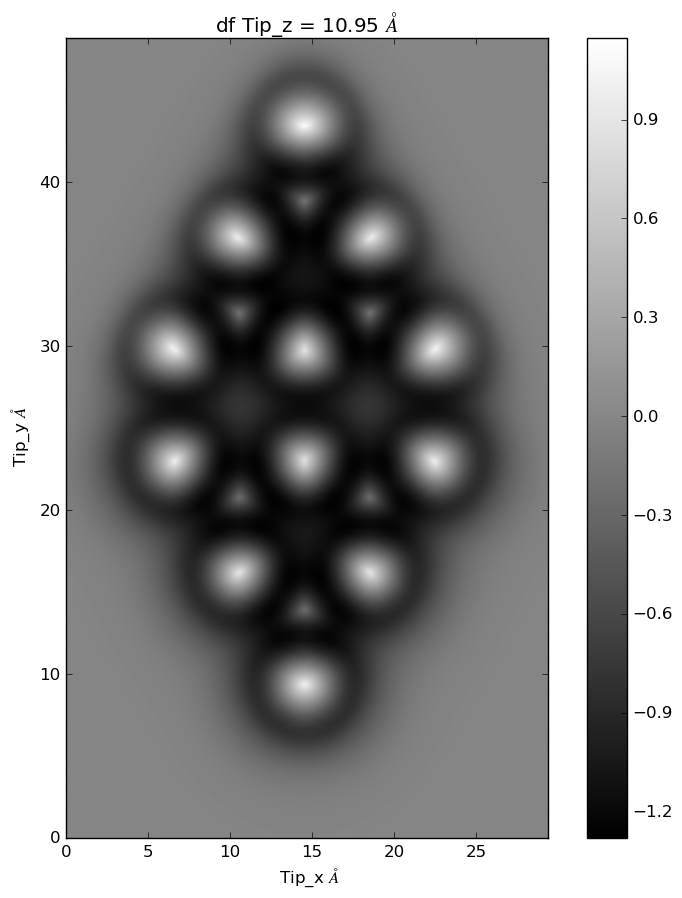

Supplement: File 9 — Datasets A0=5A k=0.5. [file Beilstein_J_Nanotechnol-07-937-s009.zip › S9/A0=5A/k=0.5/results/df_0101.png]

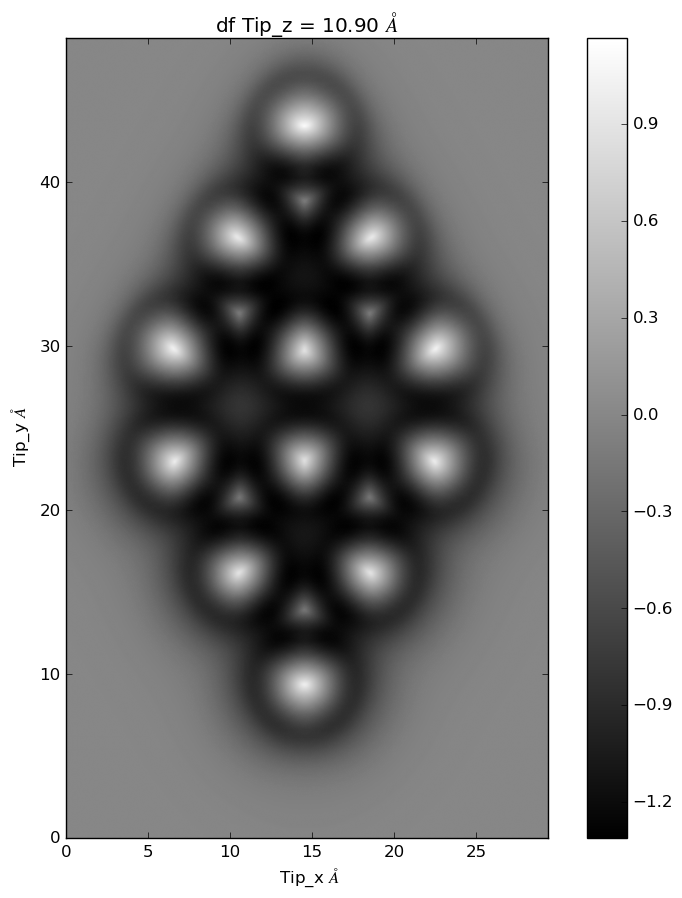

Supplement: File 9 — Datasets A0=5A k=0.5. [file Beilstein_J_Nanotechnol-07-937-s009.zip › S9/A0=5A/k=0.5/results/df_0102.png]

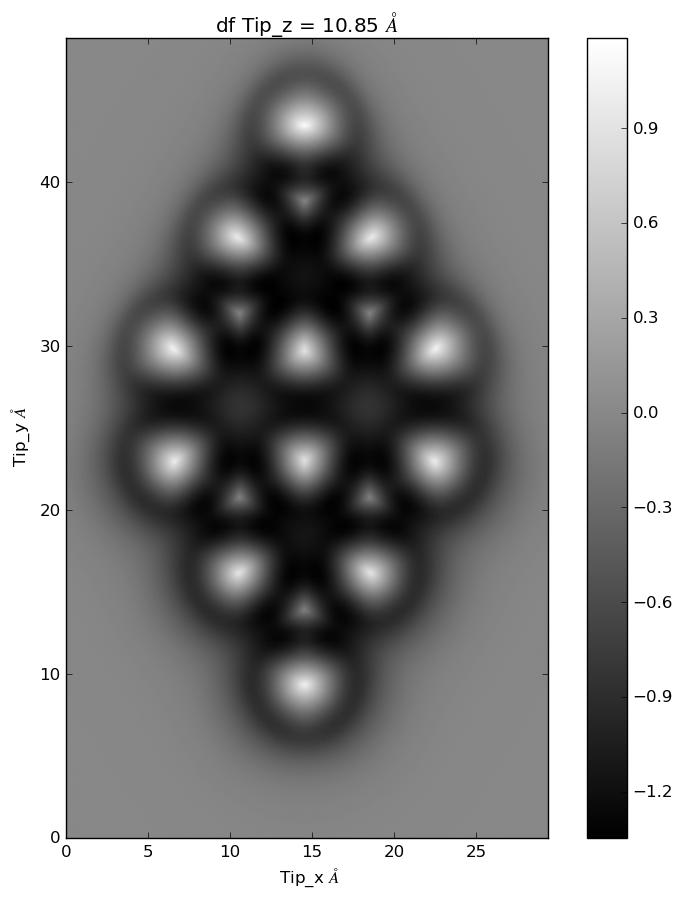

Supplement: File 9 — Datasets A0=5A k=0.5. [file Beilstein_J_Nanotechnol-07-937-s009.zip › S9/A0=5A/k=0.5/results/df_0103.png]

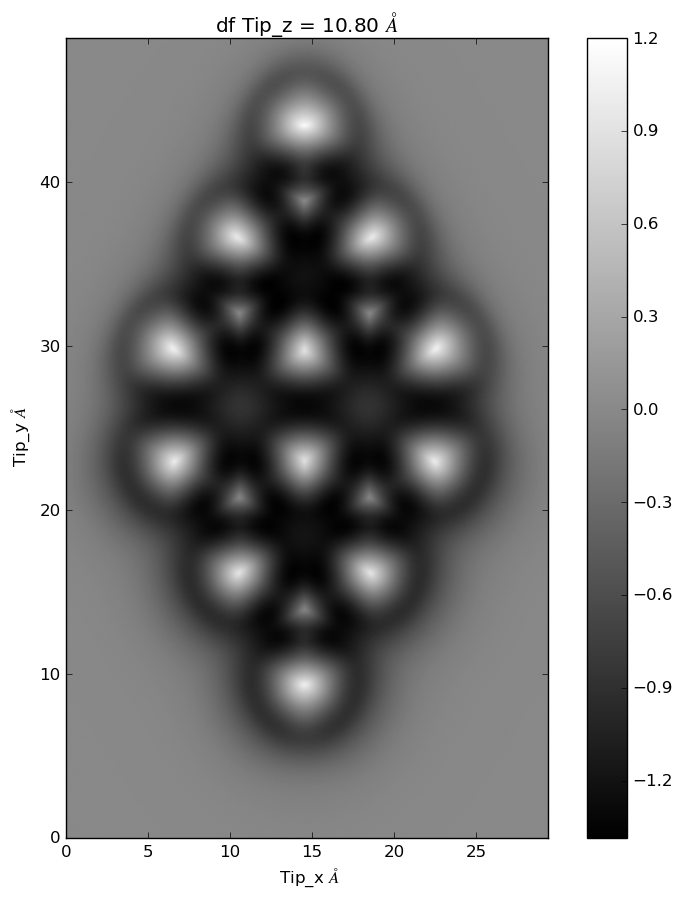

Supplement: File 9 — Datasets A0=5A k=0.5. [file Beilstein_J_Nanotechnol-07-937-s009.zip › S9/A0=5A/k=0.5/results/df_0104.png]

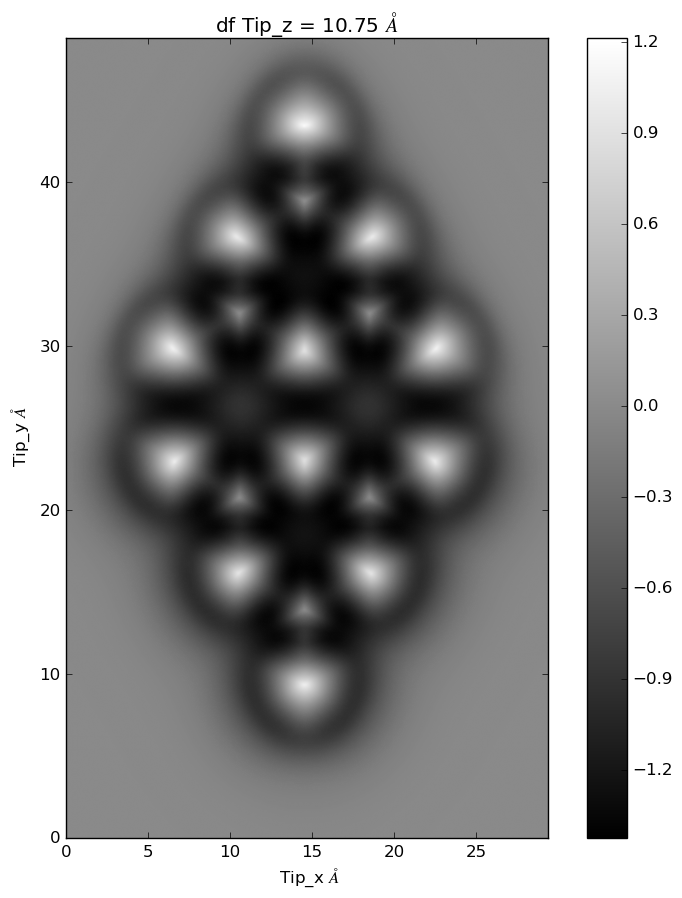

Supplement: File 9 — Datasets A0=5A k=0.5. [file Beilstein_J_Nanotechnol-07-937-s009.zip › S9/A0=5A/k=0.5/results/df_0105.png]

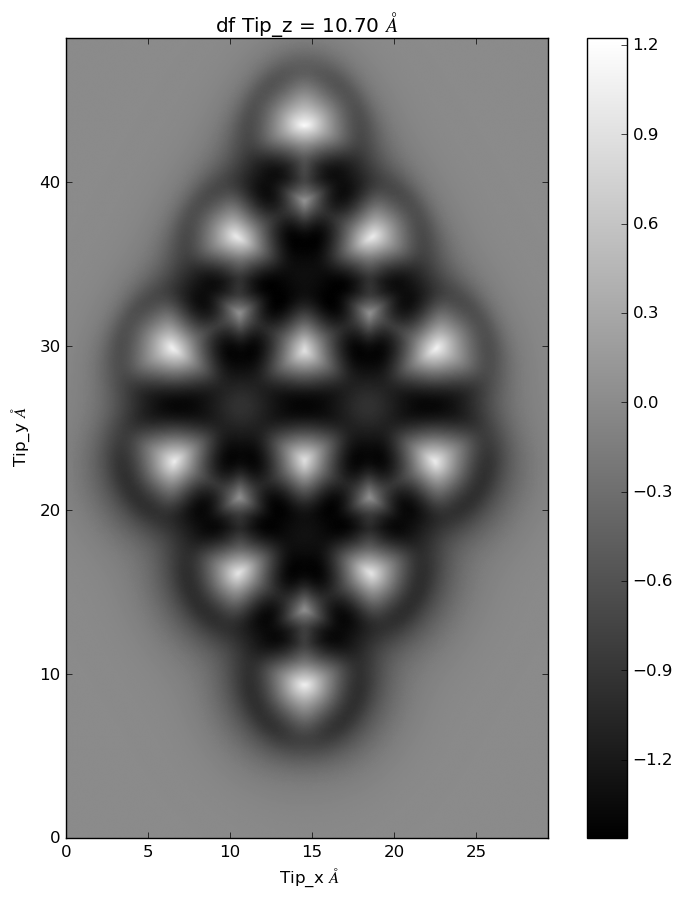

Supplement: File 9 — Datasets A0=5A k=0.5. [file Beilstein_J_Nanotechnol-07-937-s009.zip › S9/A0=5A/k=0.5/results/df_0106.png]

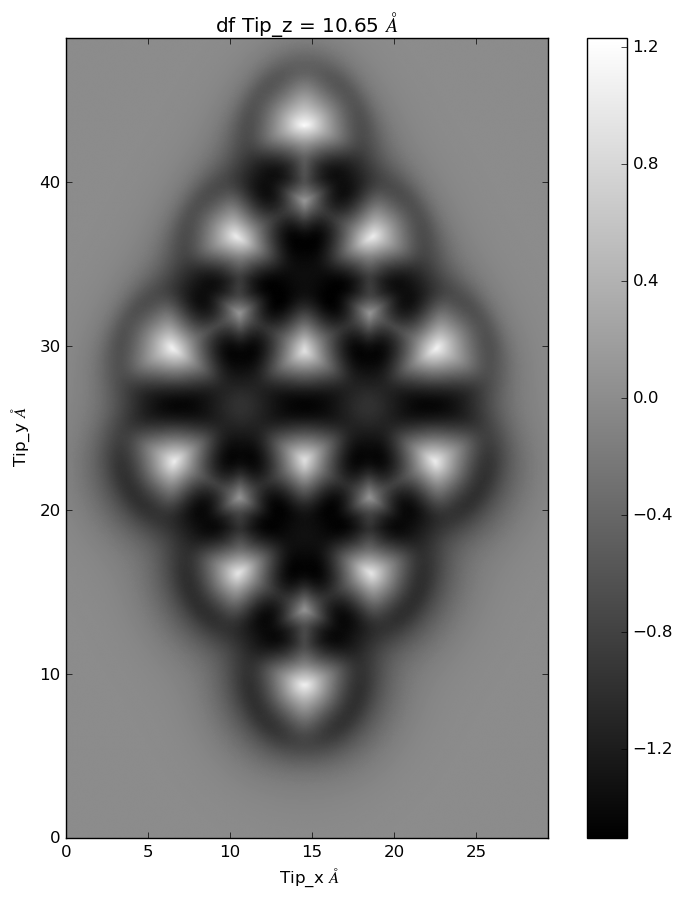

Supplement: File 9 — Datasets A0=5A k=0.5. [file Beilstein_J_Nanotechnol-07-937-s009.zip › S9/A0=5A/k=0.5/results/df_0107.png]

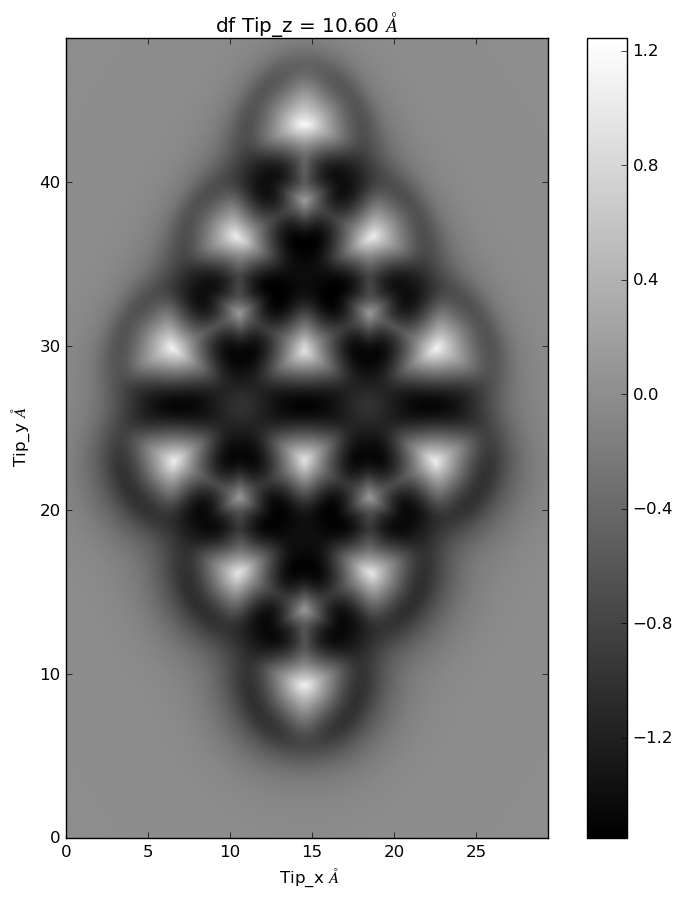

Supplement: File 9 — Datasets A0=5A k=0.5. [file Beilstein_J_Nanotechnol-07-937-s009.zip › S9/A0=5A/k=0.5/results/df_0108.png]

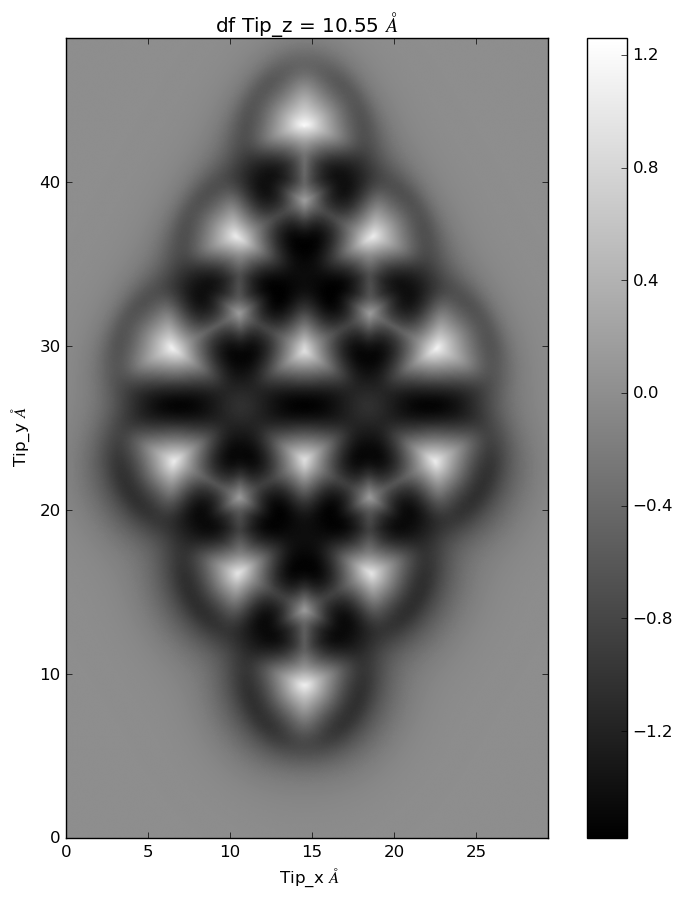

Supplement: File 9 — Datasets A0=5A k=0.5. [file Beilstein_J_Nanotechnol-07-937-s009.zip › S9/A0=5A/k=0.5/results/df_0109.png]

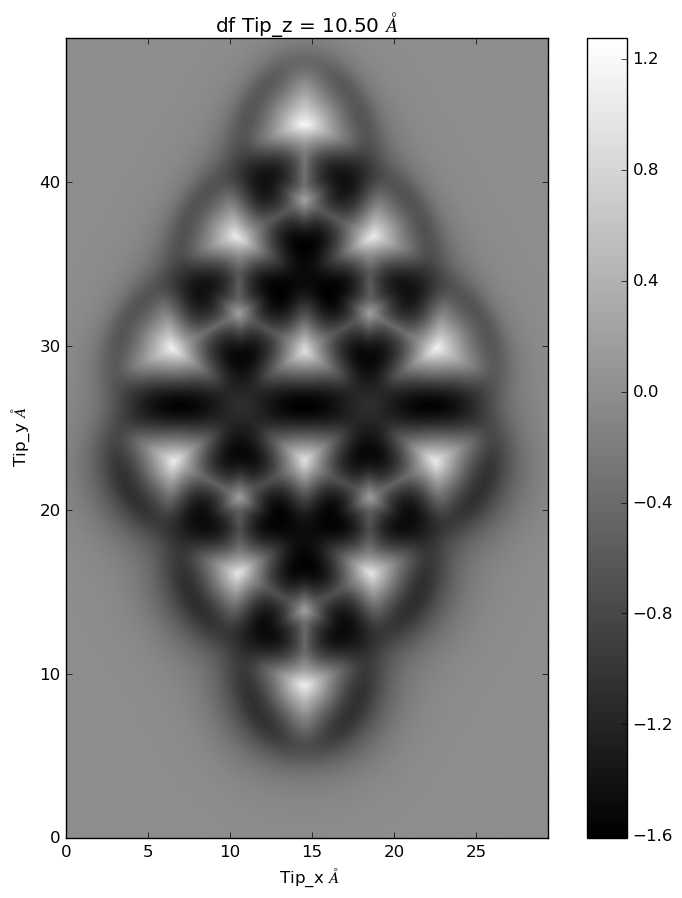

Supplement: File 9 — Datasets A0=5A k=0.5. [file Beilstein_J_Nanotechnol-07-937-s009.zip › S9/A0=5A/k=0.5/results/df_0110.png]

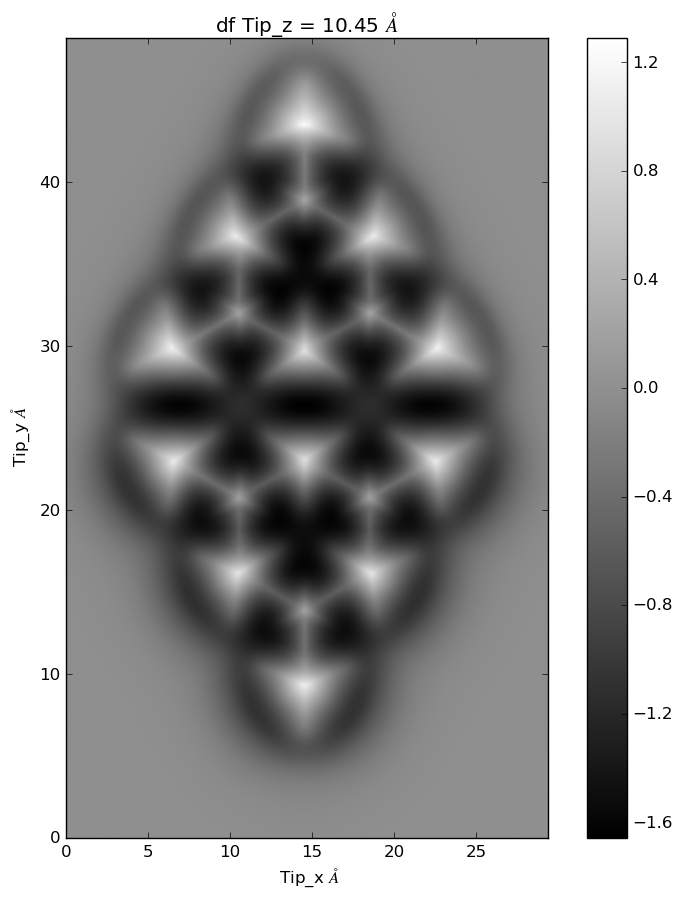

Supplement: File 9 — Datasets A0=5A k=0.5. [file Beilstein_J_Nanotechnol-07-937-s009.zip › S9/A0=5A/k=0.5/results/df_0111.png]

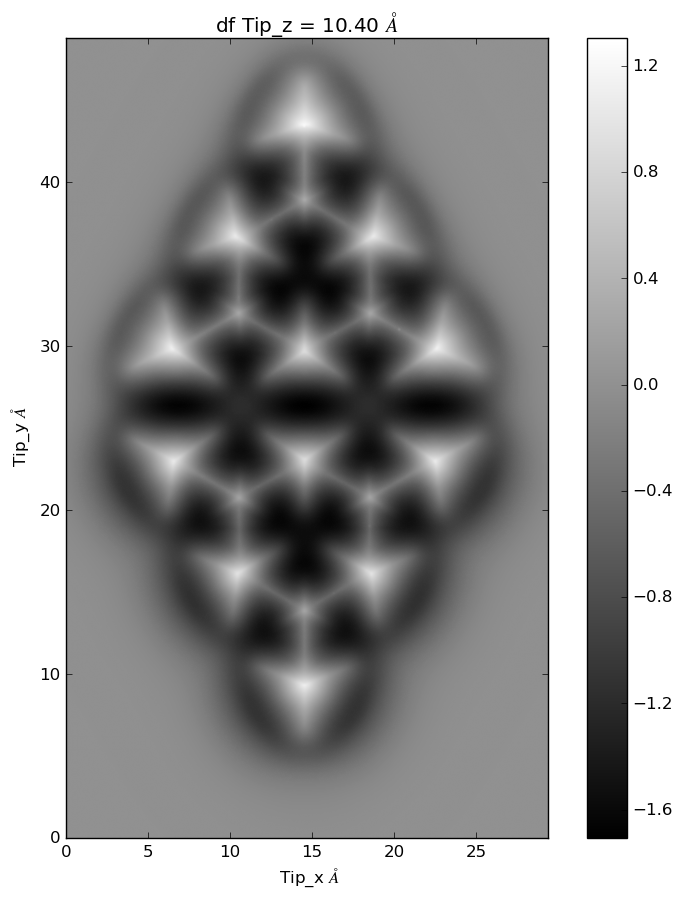

Supplement: File 9 — Datasets A0=5A k=0.5. [file Beilstein_J_Nanotechnol-07-937-s009.zip › S9/A0=5A/k=0.5/results/df_0112.png]

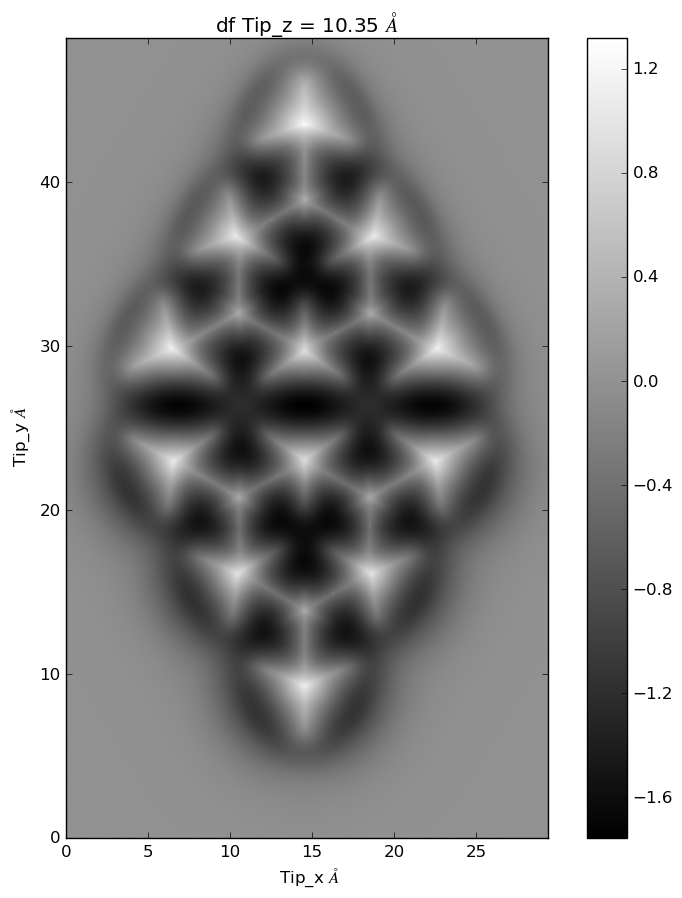

Supplement: File 9 — Datasets A0=5A k=0.5. [file Beilstein_J_Nanotechnol-07-937-s009.zip › S9/A0=5A/k=0.5/results/df_0113.png]

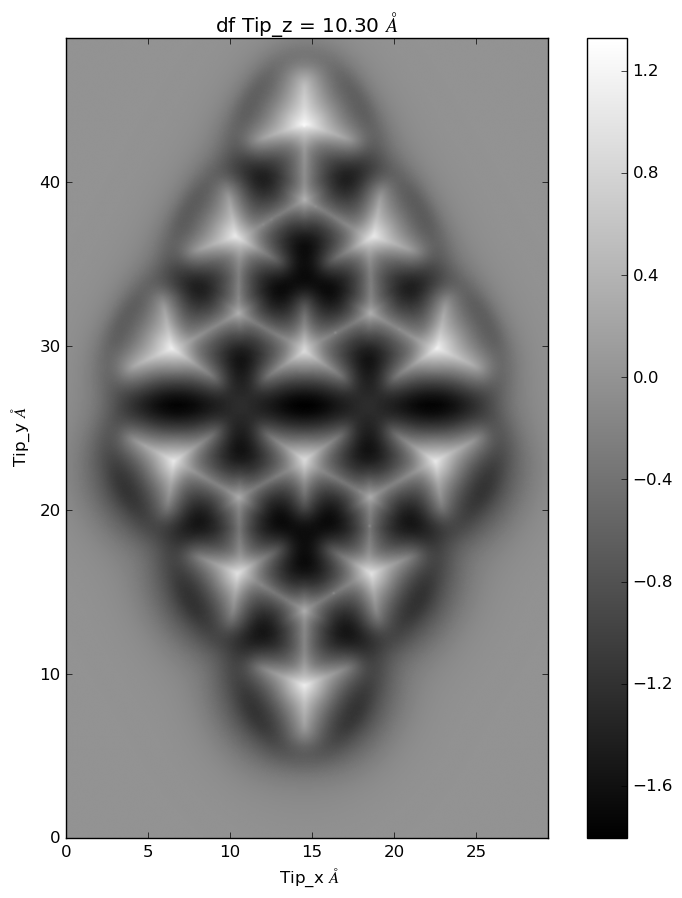

Supplement: File 9 — Datasets A0=5A k=0.5. [file Beilstein_J_Nanotechnol-07-937-s009.zip › S9/A0=5A/k=0.5/results/df_0114.png]

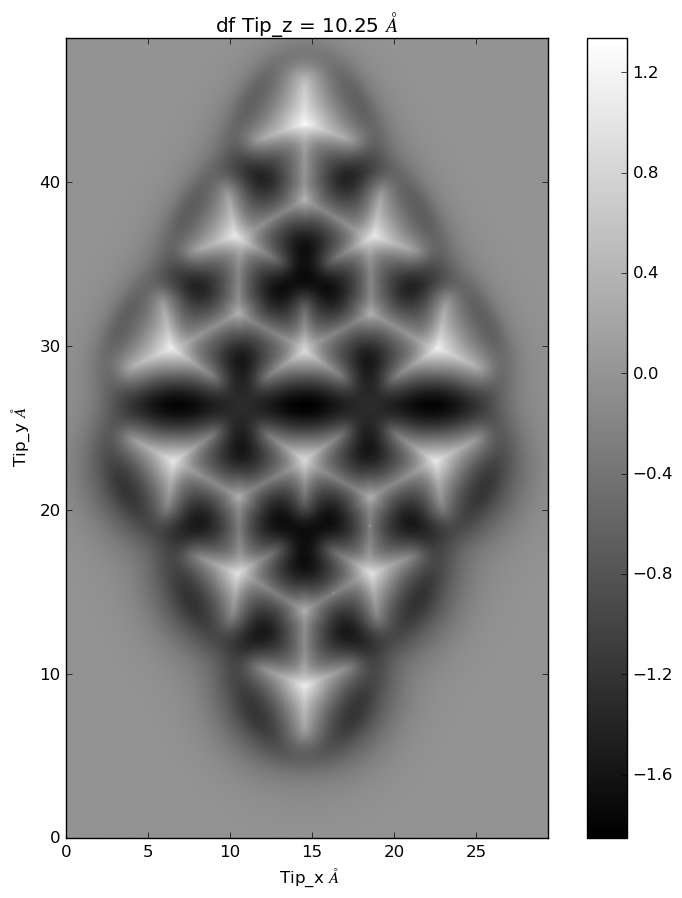

Supplement: File 9 — Datasets A0=5A k=0.5. [file Beilstein_J_Nanotechnol-07-937-s009.zip › S9/A0=5A/k=0.5/results/df_0115.png]

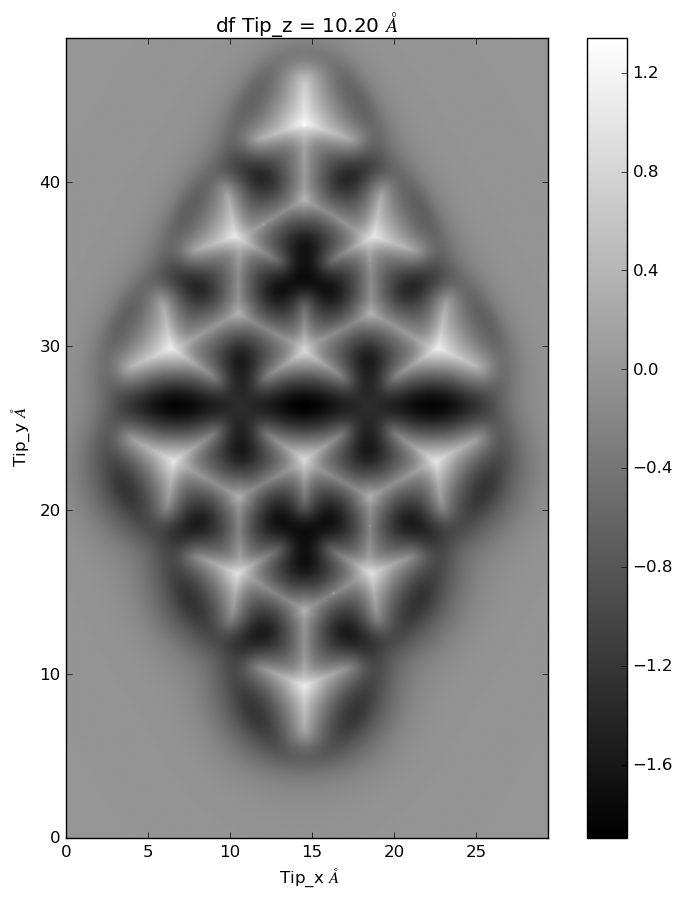

Supplement: File 9 — Datasets A0=5A k=0.5. [file Beilstein_J_Nanotechnol-07-937-s009.zip › S9/A0=5A/k=0.5/results/df_0116.png]

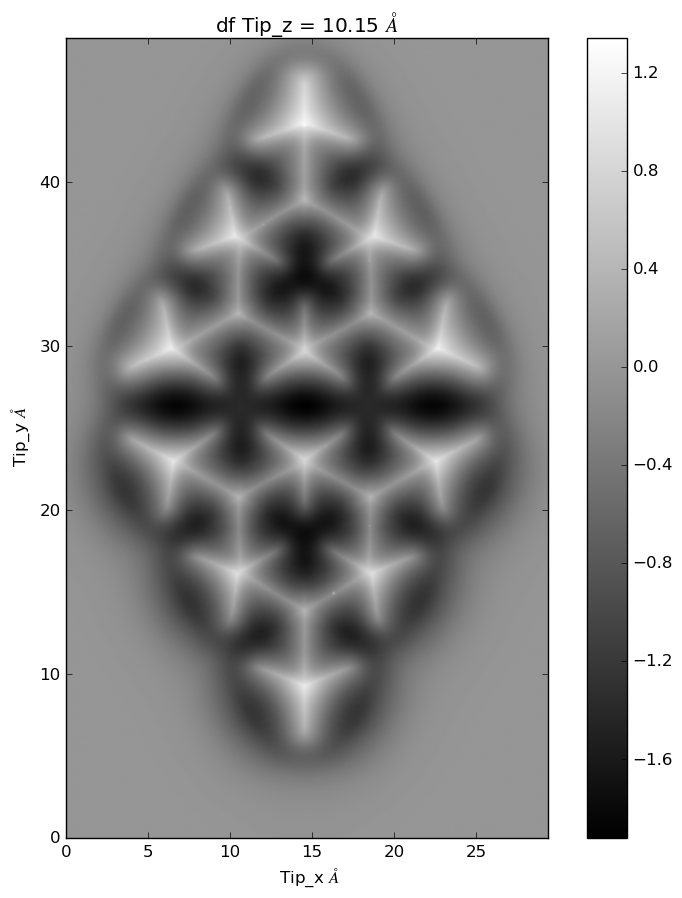

Supplement: File 9 — Datasets A0=5A k=0.5. [file Beilstein_J_Nanotechnol-07-937-s009.zip › S9/A0=5A/k=0.5/results/df_0117.png]

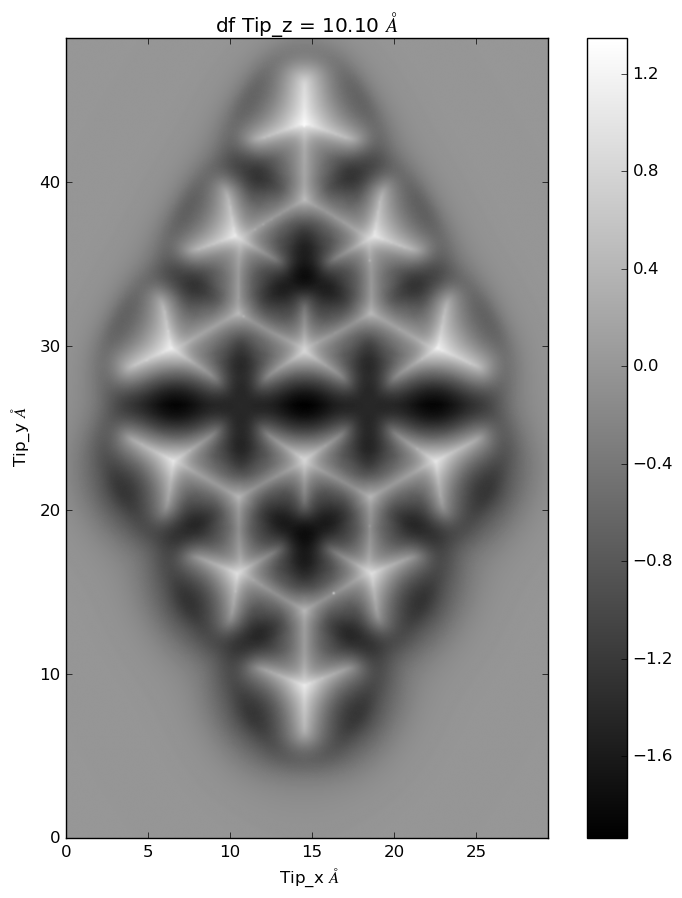

Supplement: File 9 — Datasets A0=5A k=0.5. [file Beilstein_J_Nanotechnol-07-937-s009.zip › S9/A0=5A/k=0.5/results/df_0118.png]

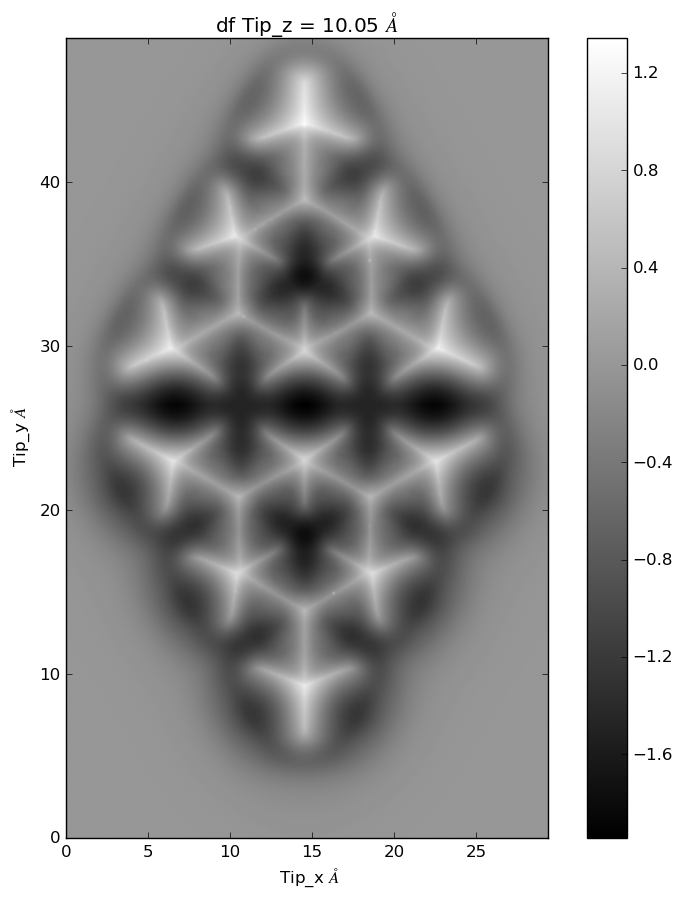

Supplement: File 9 — Datasets A0=5A k=0.5. [file Beilstein_J_Nanotechnol-07-937-s009.zip › S9/A0=5A/k=0.5/results/df_0119.png]

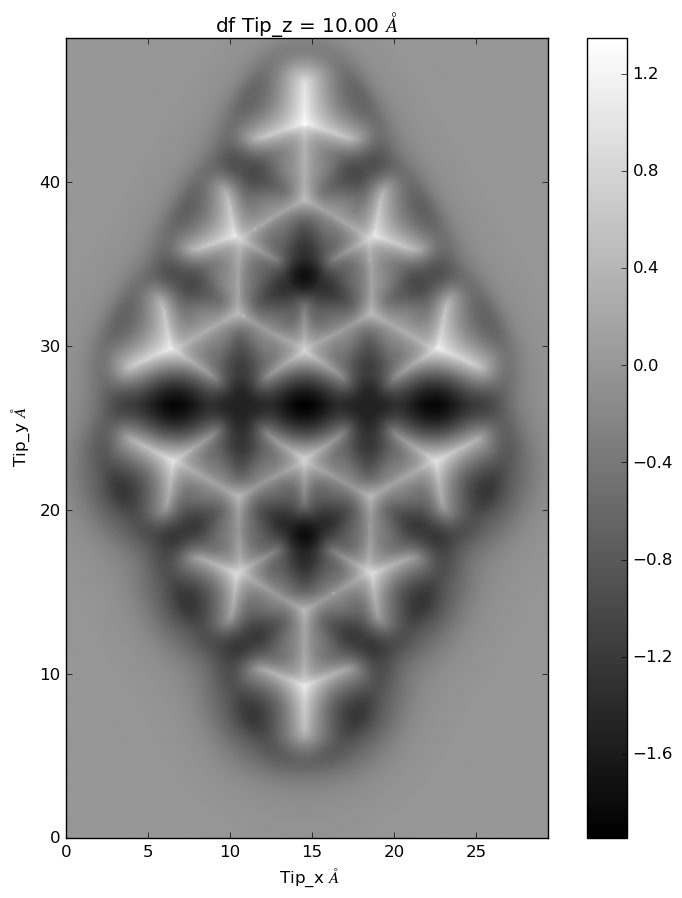

Supplement: File 9 — Datasets A0=5A k=0.5. [file Beilstein_J_Nanotechnol-07-937-s009.zip › S9/A0=5A/k=0.5/results/df_0120.png]

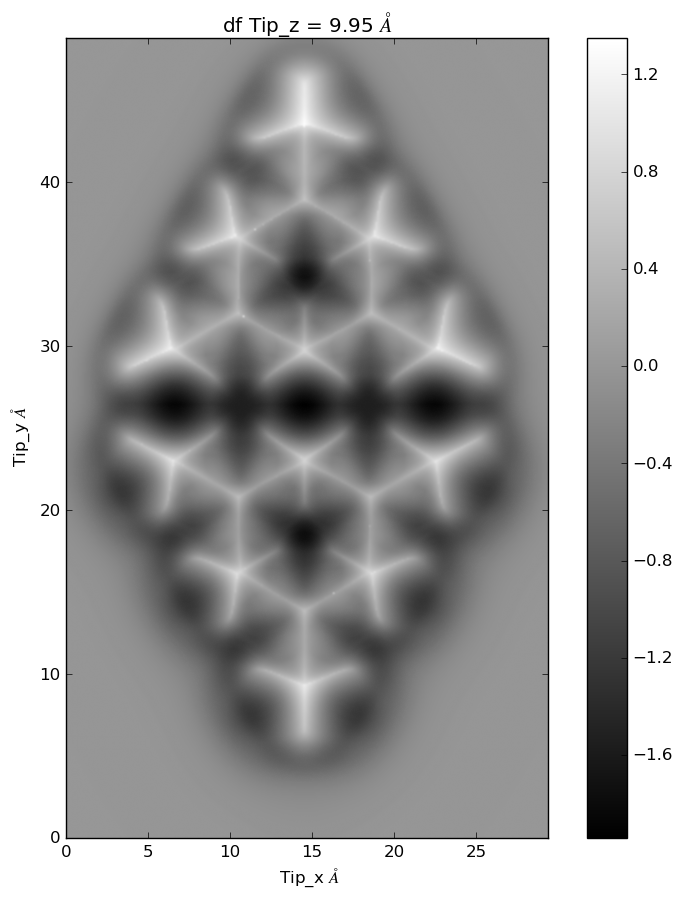

Supplement: File 9 — Datasets A0=5A k=0.5. [file Beilstein_J_Nanotechnol-07-937-s009.zip › S9/A0=5A/k=0.5/results/df_0121.png]

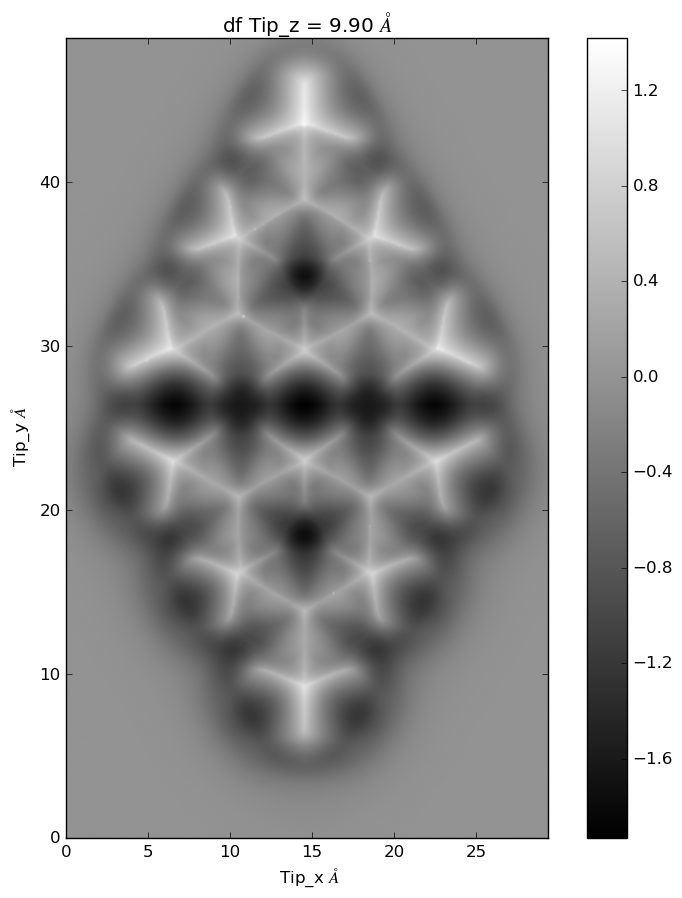

Supplement: File 9 — Datasets A0=5A k=0.5. [file Beilstein_J_Nanotechnol-07-937-s009.zip › S9/A0=5A/k=0.5/results/df_0122.png]

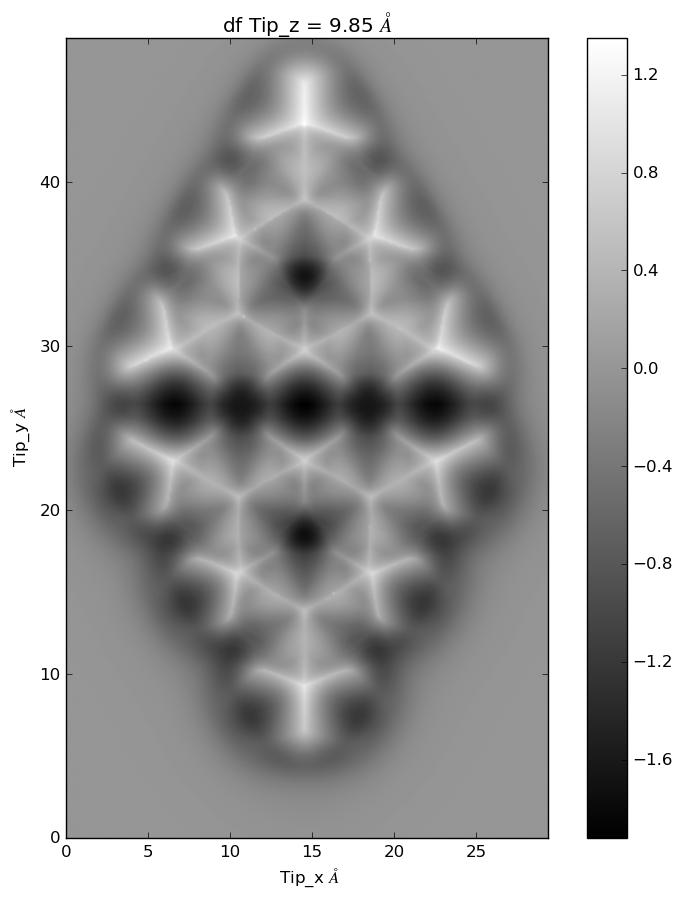

Supplement: File 9 — Datasets A0=5A k=0.5. [file Beilstein_J_Nanotechnol-07-937-s009.zip › S9/A0=5A/k=0.5/results/df_0123.png]

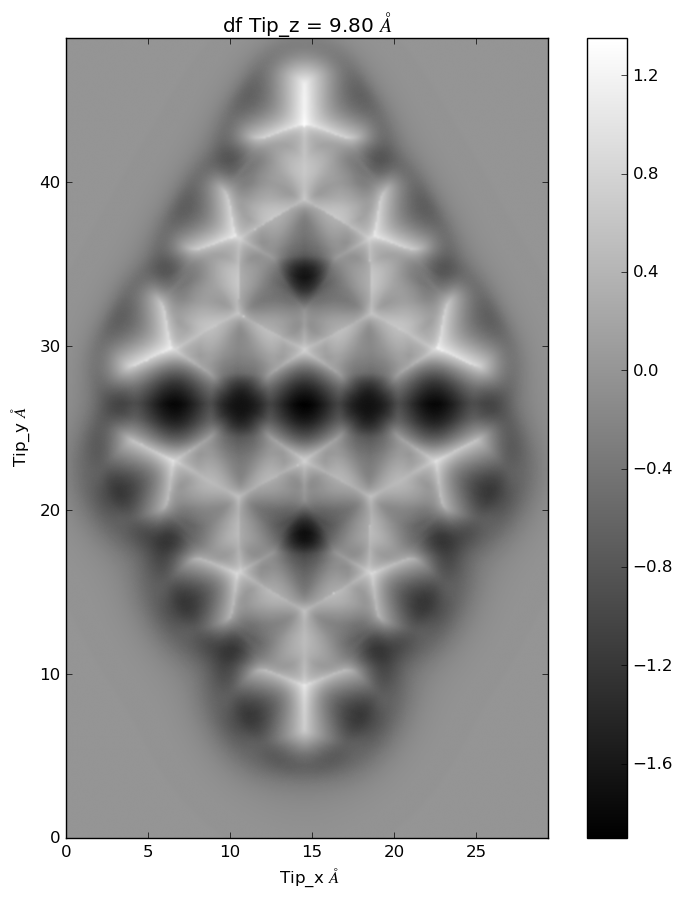

Supplement: File 9 — Datasets A0=5A k=0.5. [file Beilstein_J_Nanotechnol-07-937-s009.zip › S9/A0=5A/k=0.5/results/df_0124.png]

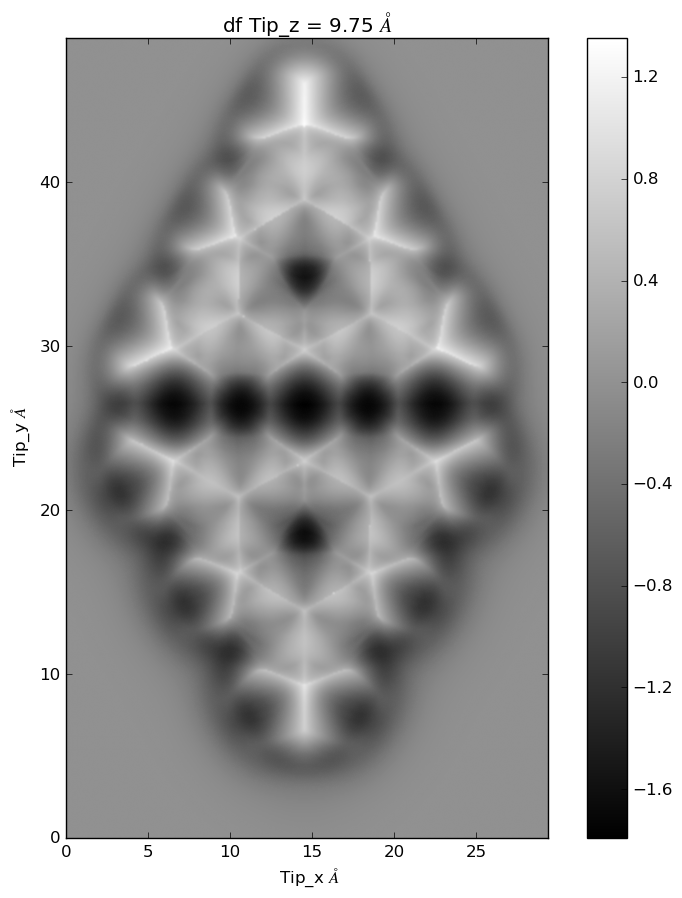

Supplement: File 9 — Datasets A0=5A k=0.5. [file Beilstein_J_Nanotechnol-07-937-s009.zip › S9/A0=5A/k=0.5/results/df_0125.png]

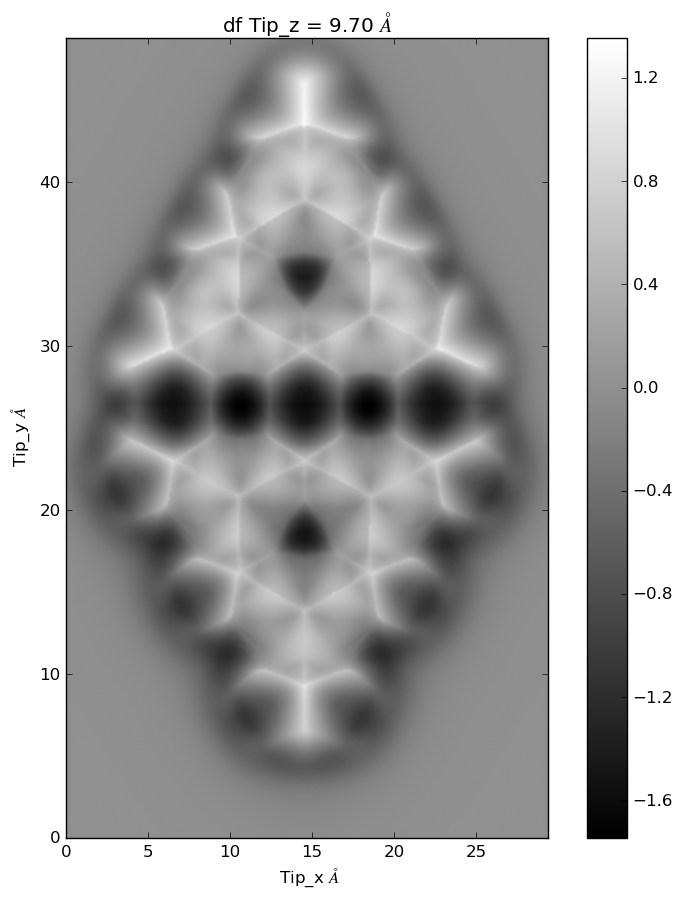

Supplement: File 9 — Datasets A0=5A k=0.5. [file Beilstein_J_Nanotechnol-07-937-s009.zip › S9/A0=5A/k=0.5/results/df_0126.png]

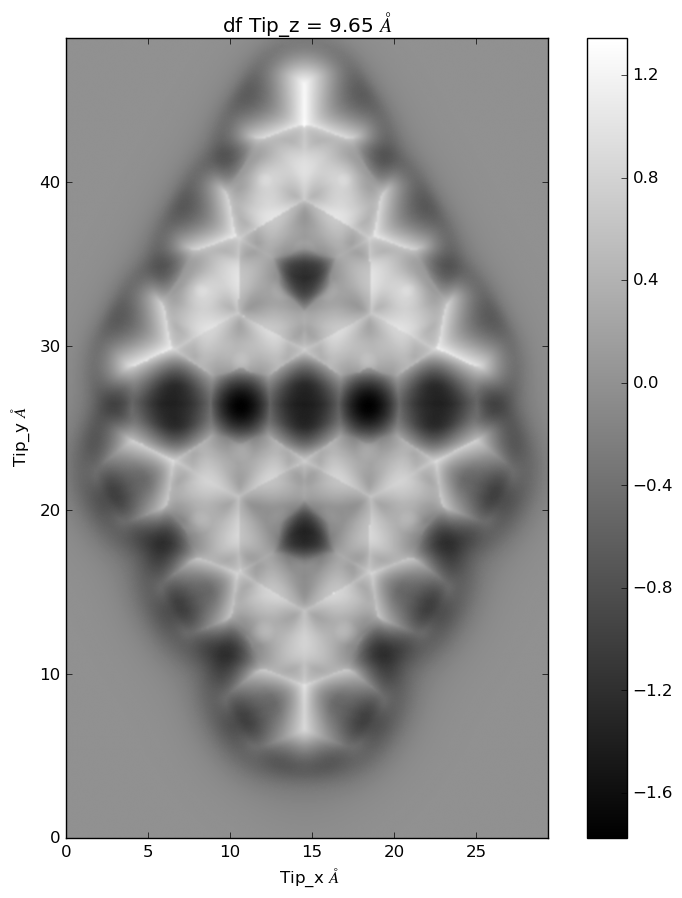

Supplement: File 9 — Datasets A0=5A k=0.5. [file Beilstein_J_Nanotechnol-07-937-s009.zip › S9/A0=5A/k=0.5/results/df_0127.png]

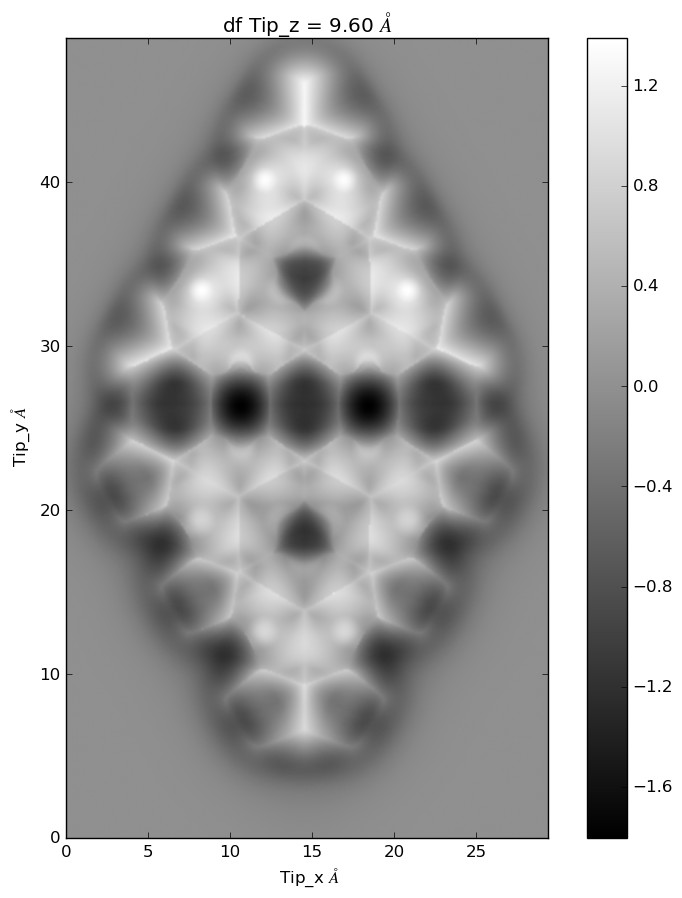

Supplement: File 9 — Datasets A0=5A k=0.5. [file Beilstein_J_Nanotechnol-07-937-s009.zip › S9/A0=5A/k=0.5/results/df_0128.png]

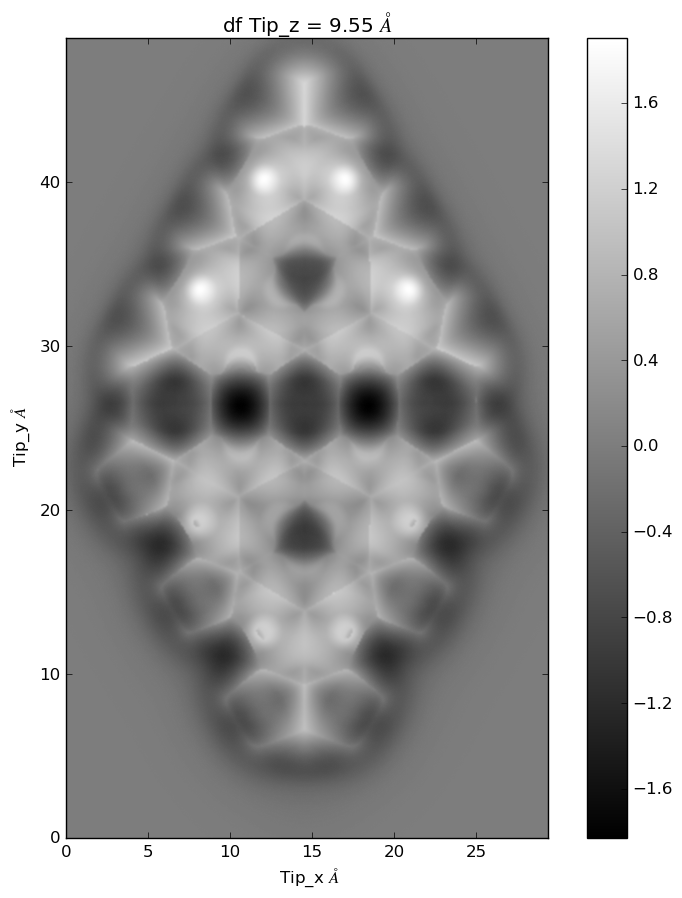

Supplement: File 9 — Datasets A0=5A k=0.5. [file Beilstein_J_Nanotechnol-07-937-s009.zip › S9/A0=5A/k=0.5/results/df_0129.png]

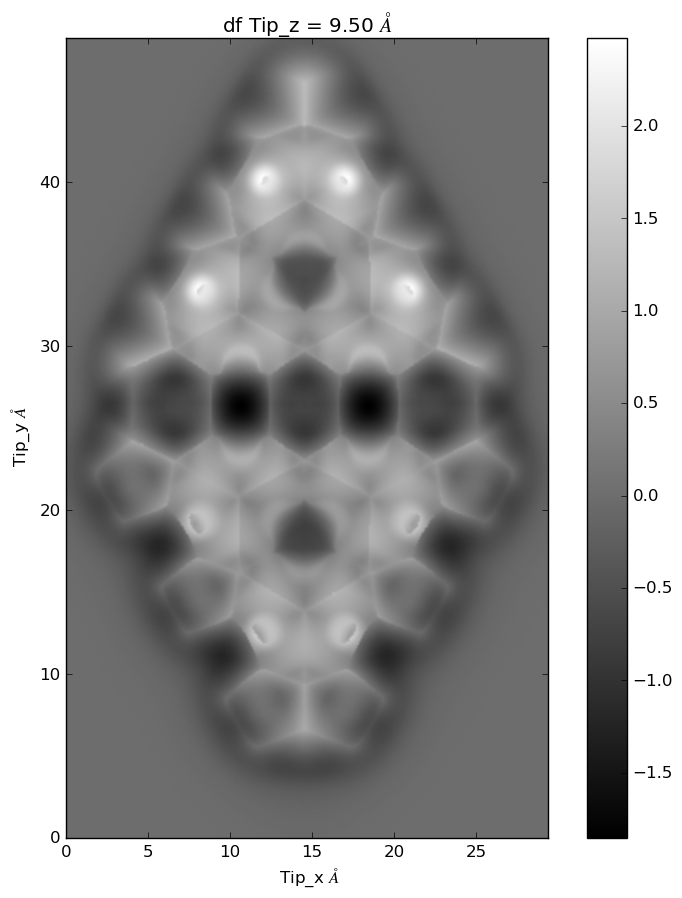

Supplement: File 9 — Datasets A0=5A k=0.5. [file Beilstein_J_Nanotechnol-07-937-s009.zip › S9/A0=5A/k=0.5/results/df_0130.png]

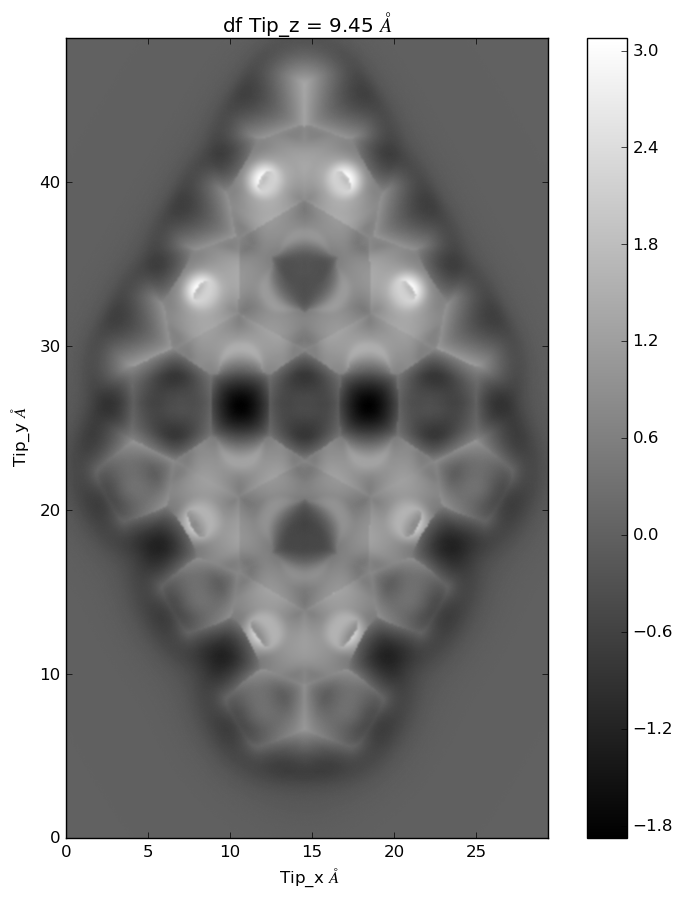

Supplement: File 9 — Datasets A0=5A k=0.5. [file Beilstein_J_Nanotechnol-07-937-s009.zip › S9/A0=5A/k=0.5/results/df_0131.png]

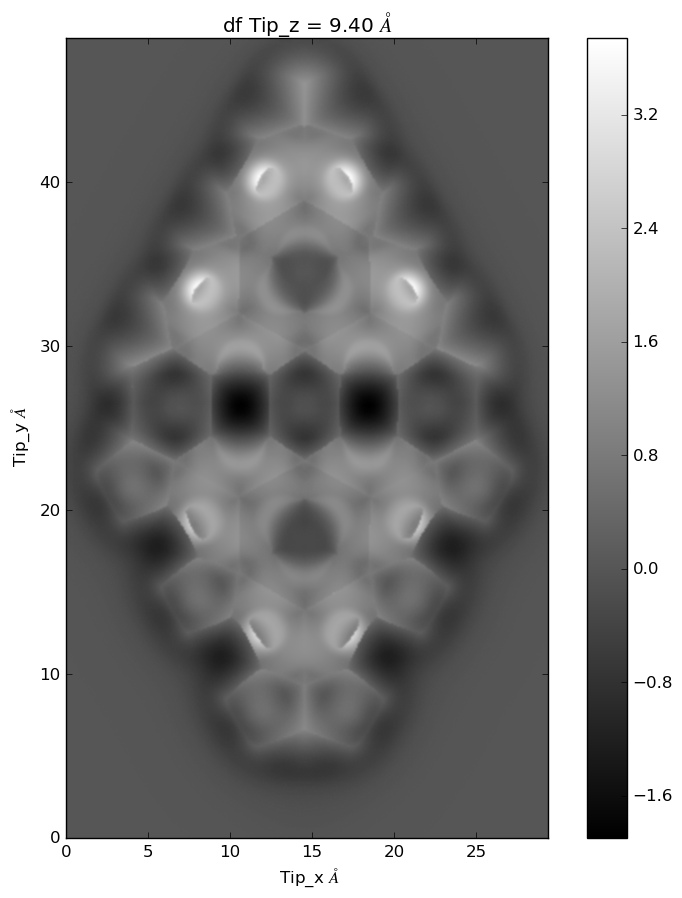

Supplement: File 9 — Datasets A0=5A k=0.5. [file Beilstein_J_Nanotechnol-07-937-s009.zip › S9/A0=5A/k=0.5/results/df_0132.png]

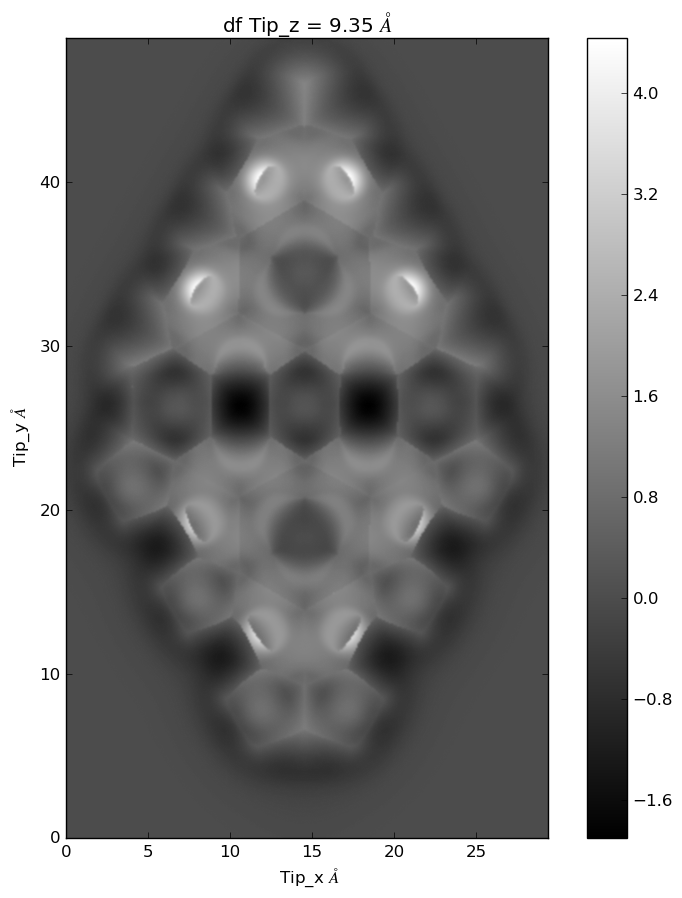

Supplement: File 9 — Datasets A0=5A k=0.5. [file Beilstein_J_Nanotechnol-07-937-s009.zip › S9/A0=5A/k=0.5/results/df_0133.png]

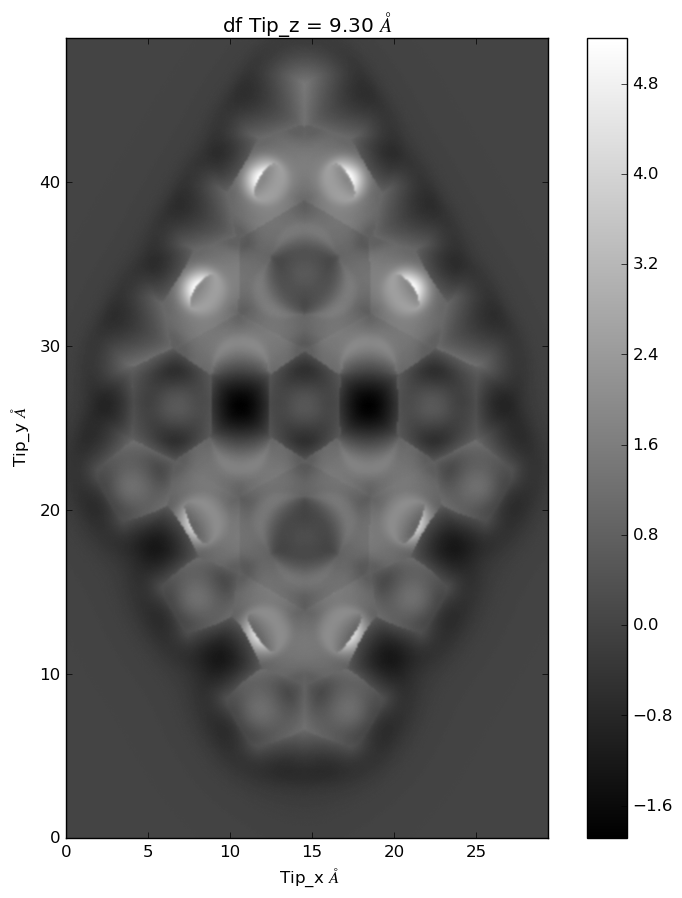

Supplement: File 9 — Datasets A0=5A k=0.5. [file Beilstein_J_Nanotechnol-07-937-s009.zip › S9/A0=5A/k=0.5/results/df_0134.png]

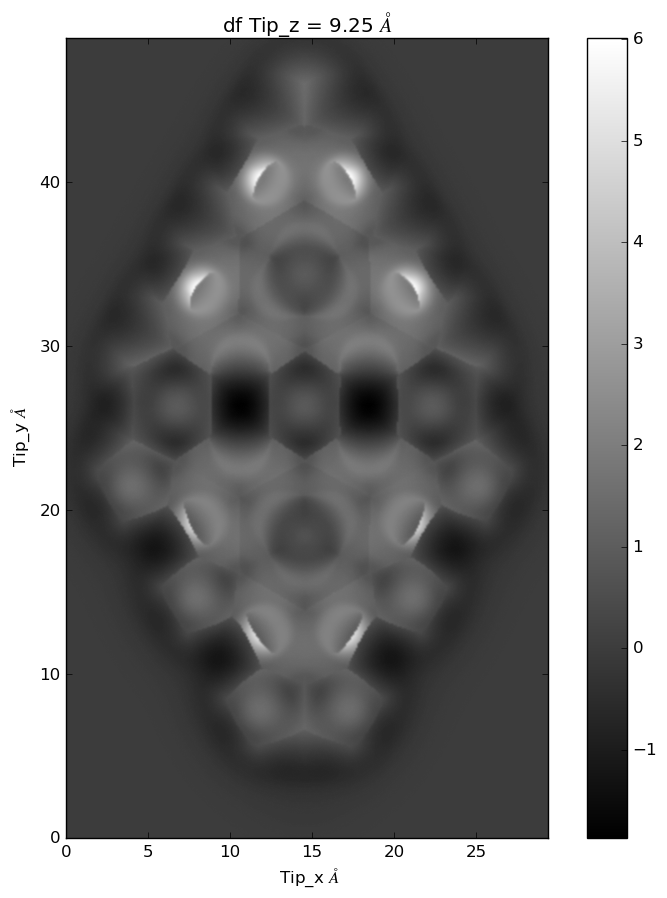

Supplement: File 9 — Datasets A0=5A k=0.5. [file Beilstein_J_Nanotechnol-07-937-s009.zip › S9/A0=5A/k=0.5/results/df_0135.png]

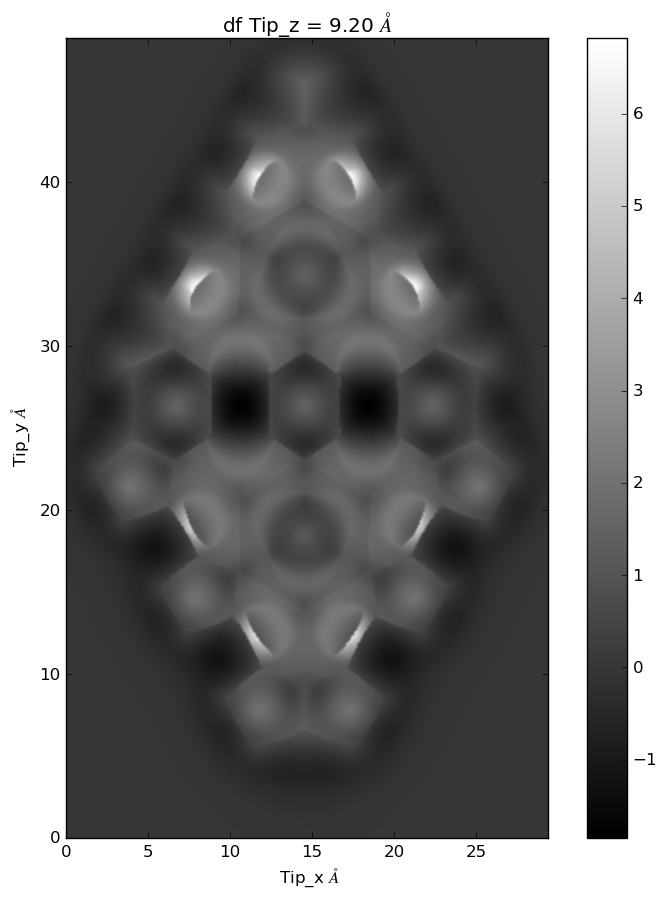

Supplement: File 9 — Datasets A0=5A k=0.5. [file Beilstein_J_Nanotechnol-07-937-s009.zip › S9/A0=5A/k=0.5/results/df_0136.png]

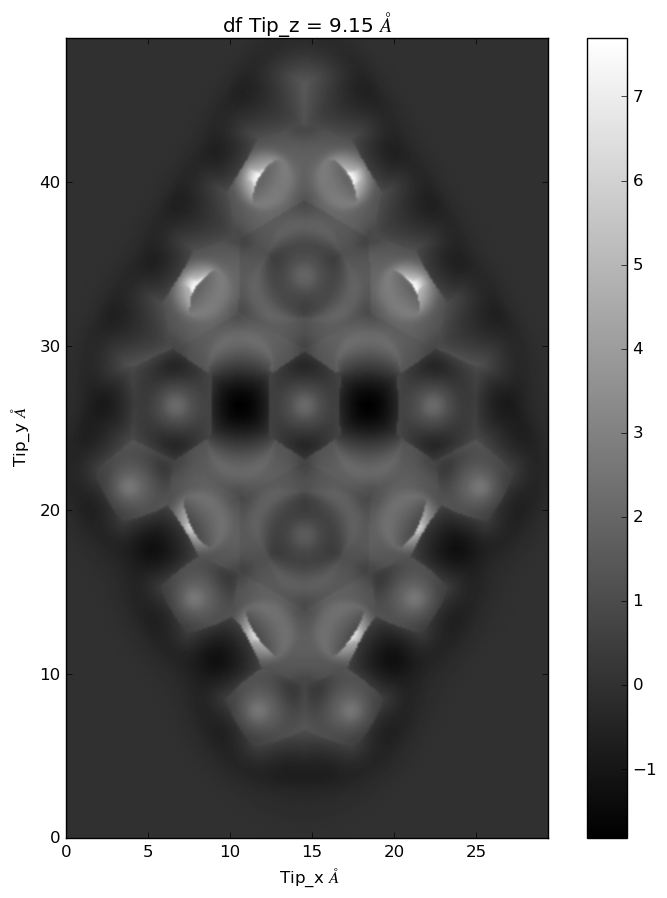

Supplement: File 9 — Datasets A0=5A k=0.5. [file Beilstein_J_Nanotechnol-07-937-s009.zip › S9/A0=5A/k=0.5/results/df_0137.png]

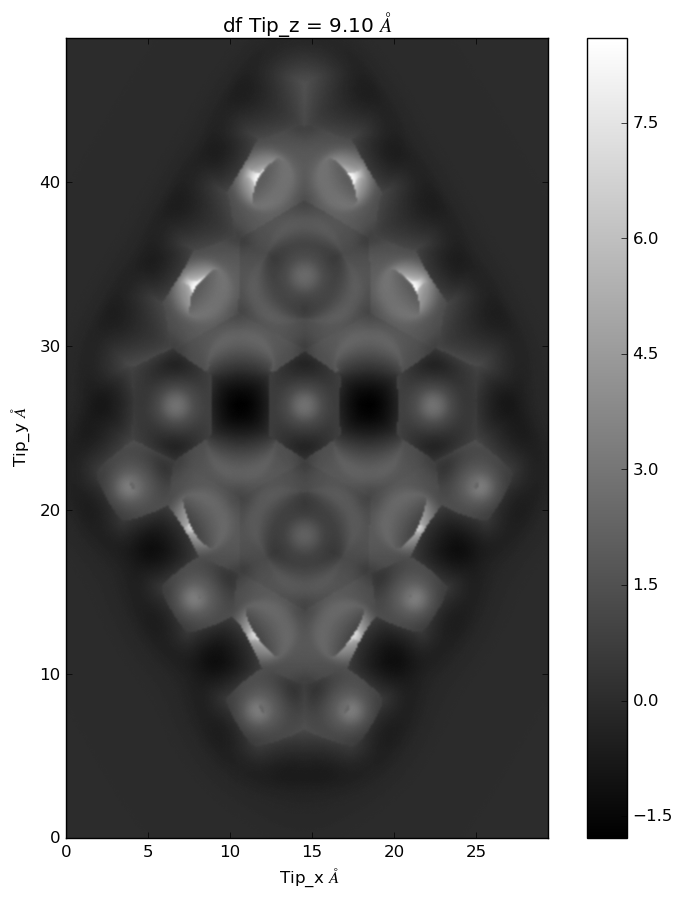

Supplement: File 9 — Datasets A0=5A k=0.5. [file Beilstein_J_Nanotechnol-07-937-s009.zip › S9/A0=5A/k=0.5/results/df_0138.png]

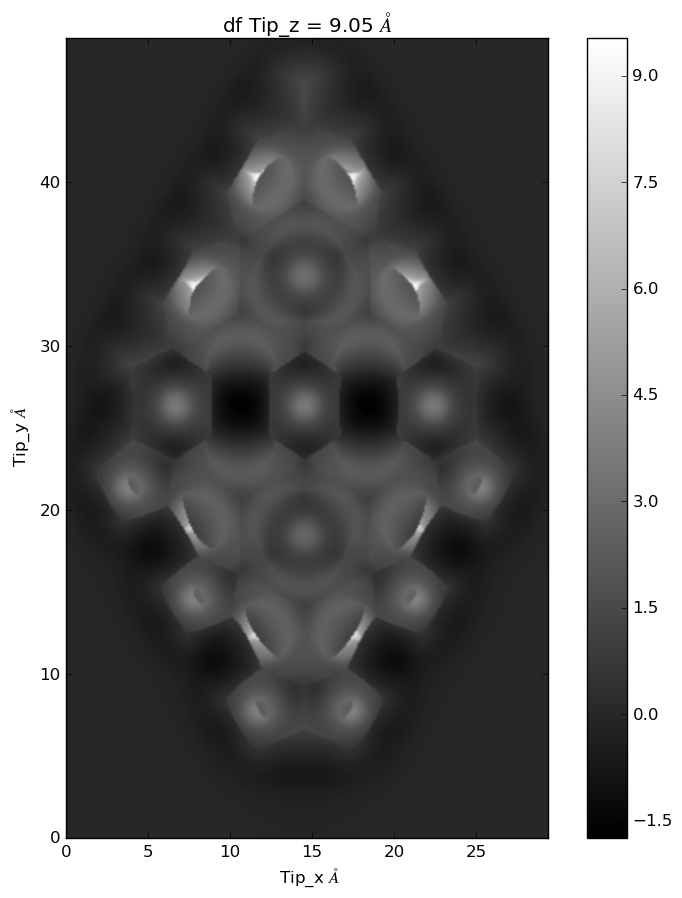

Supplement: File 9 — Datasets A0=5A k=0.5. [file Beilstein_J_Nanotechnol-07-937-s009.zip › S9/A0=5A/k=0.5/results/df_0139.png]

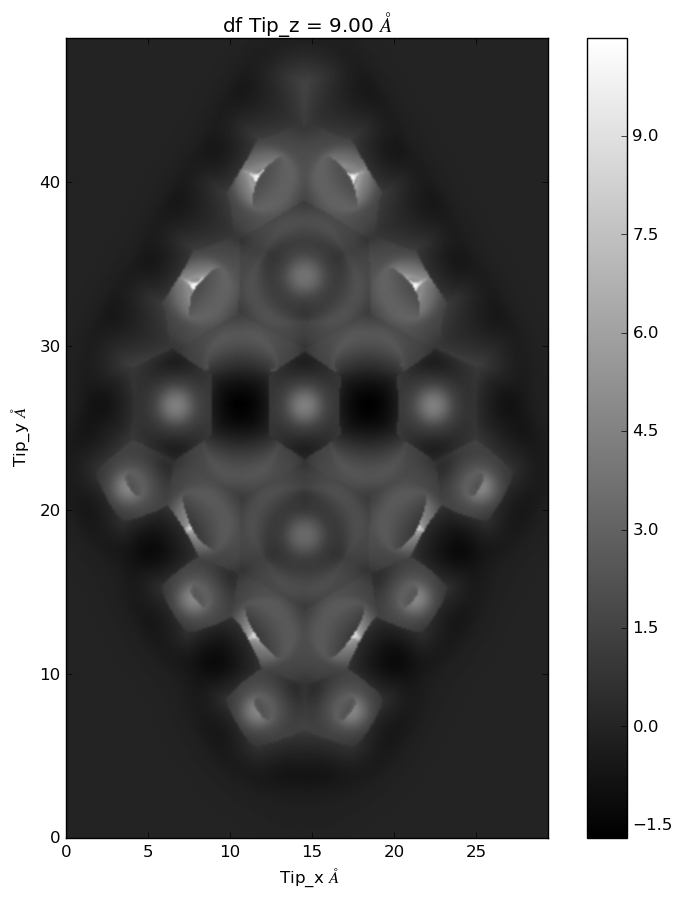

Supplement: File 9 — Datasets A0=5A k=0.5. [file Beilstein_J_Nanotechnol-07-937-s009.zip › S9/A0=5A/k=0.5/results/df_0140.png]

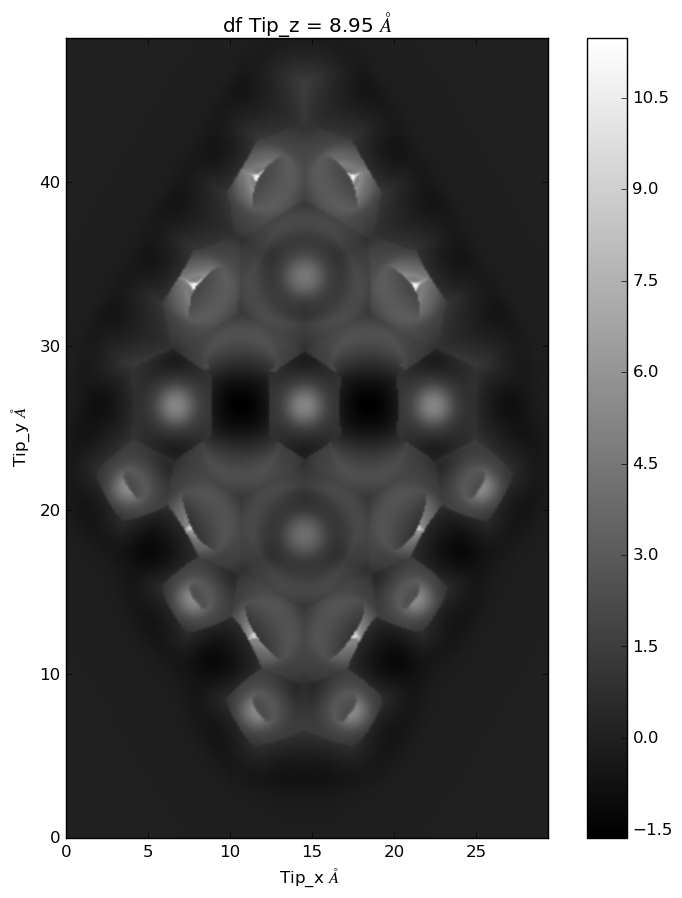

Supplement: File 9 — Datasets A0=5A k=0.5. [file Beilstein_J_Nanotechnol-07-937-s009.zip › S9/A0=5A/k=0.5/results/df_0141.png]

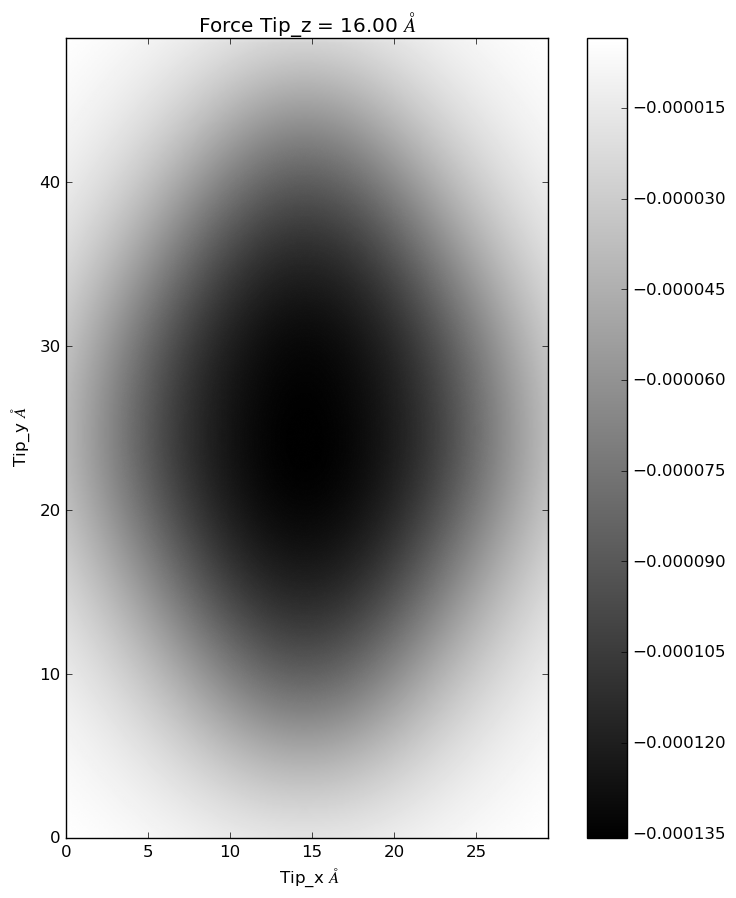

Supplement: File 9 — Datasets A0=5A k=0.5. [file Beilstein_J_Nanotechnol-07-937-s009.zip › S9/A0=5A/k=0.5/results/Force_0000.png]

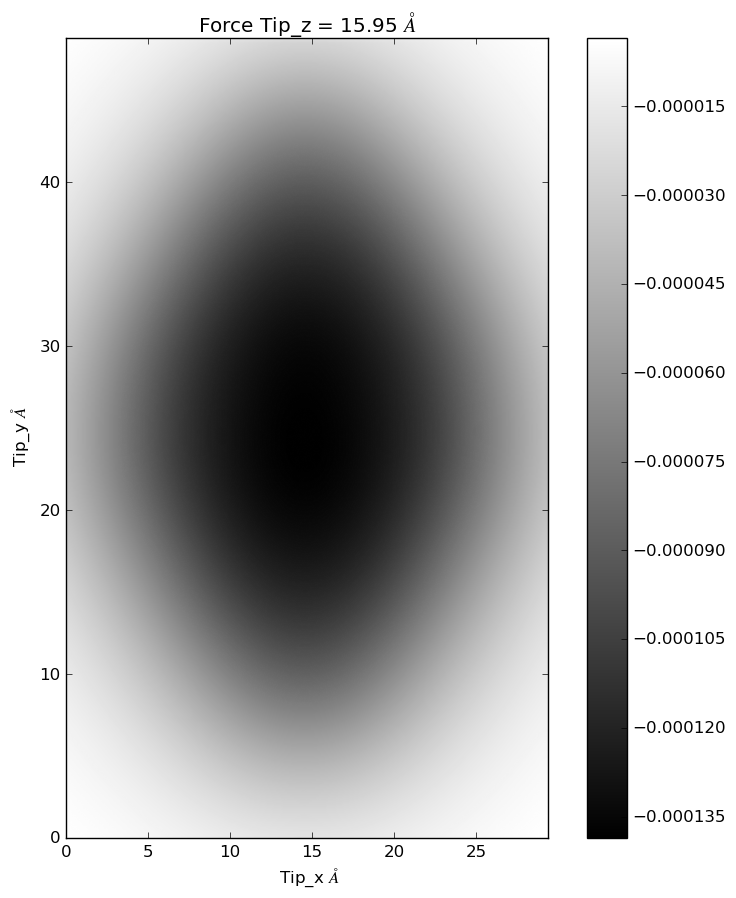

Supplement: File 9 — Datasets A0=5A k=0.5. [file Beilstein_J_Nanotechnol-07-937-s009.zip › S9/A0=5A/k=0.5/results/Force_0001.png]

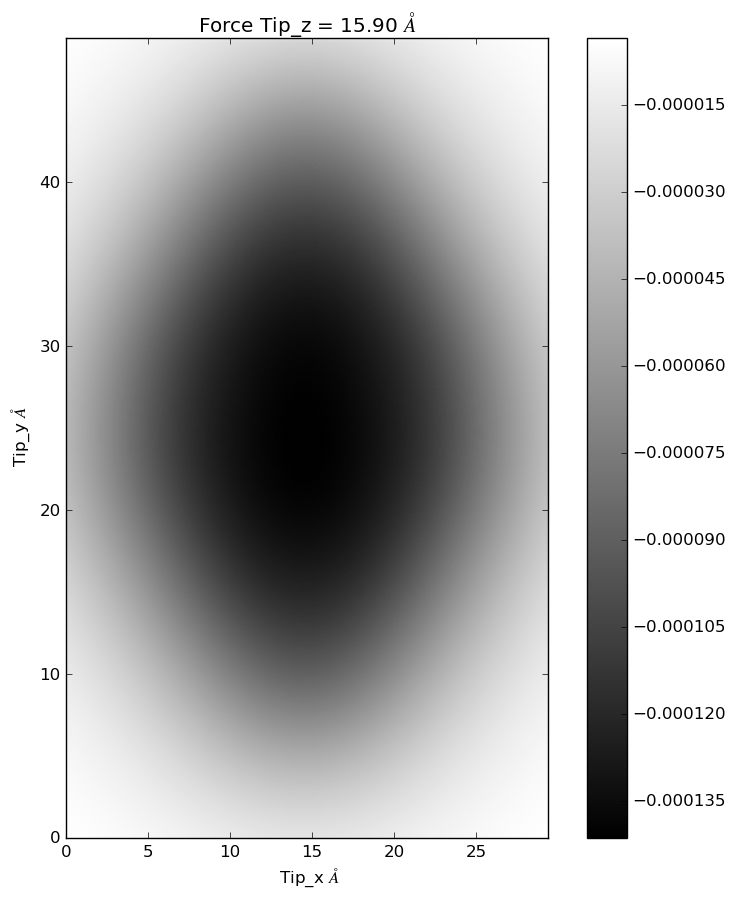

Supplement: File 9 — Datasets A0=5A k=0.5. [file Beilstein_J_Nanotechnol-07-937-s009.zip › S9/A0=5A/k=0.5/results/Force_0002.png]

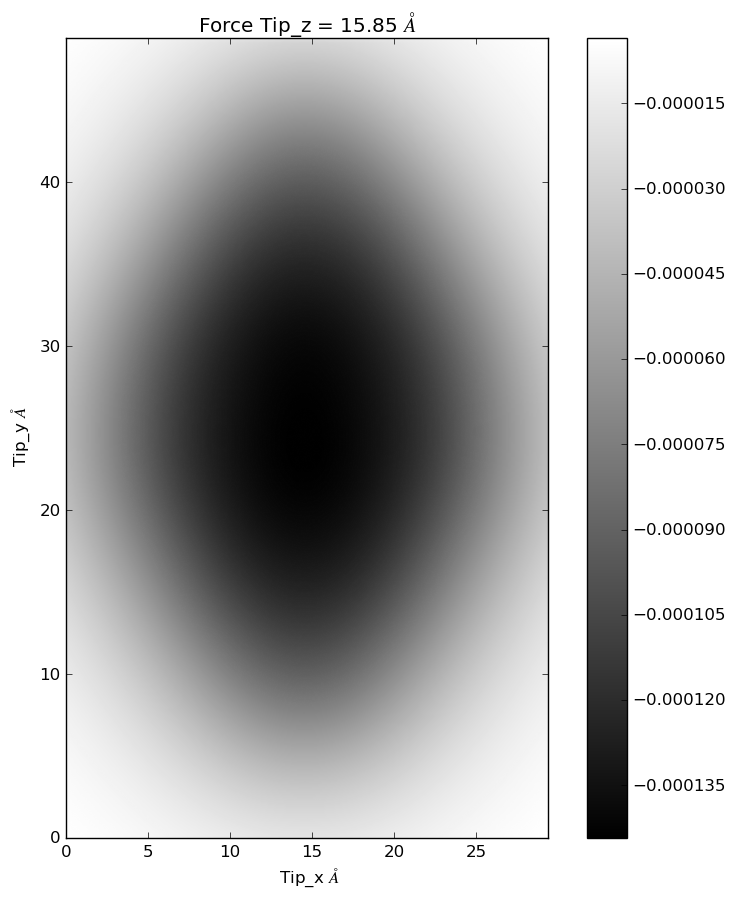

Supplement: File 9 — Datasets A0=5A k=0.5. [file Beilstein_J_Nanotechnol-07-937-s009.zip › S9/A0=5A/k=0.5/results/Force_0003.png]

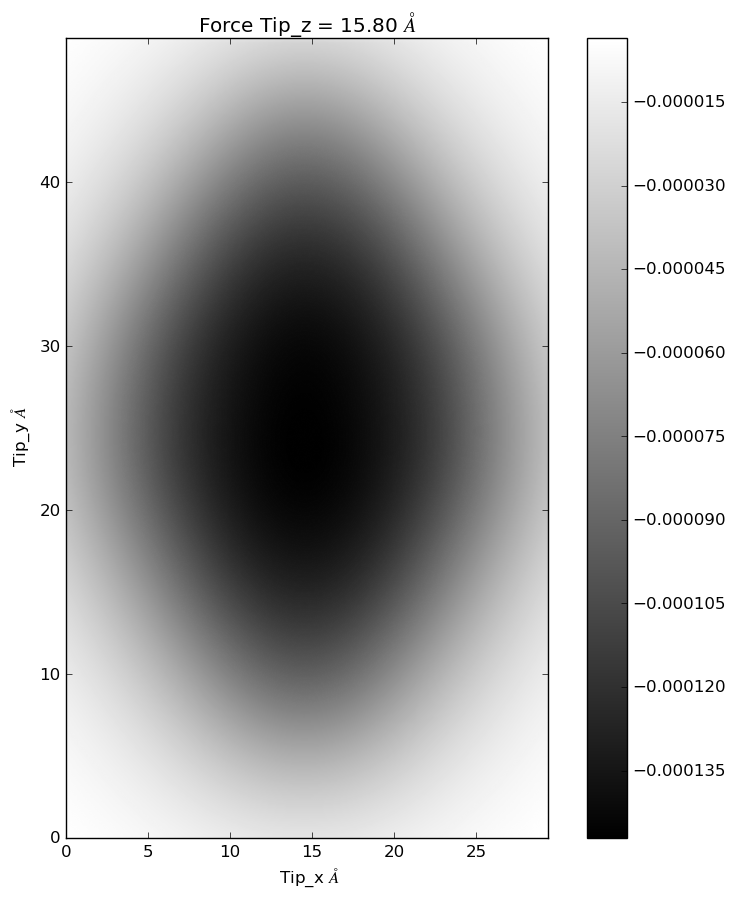

Supplement: File 9 — Datasets A0=5A k=0.5. [file Beilstein_J_Nanotechnol-07-937-s009.zip › S9/A0=5A/k=0.5/results/Force_0004.png]

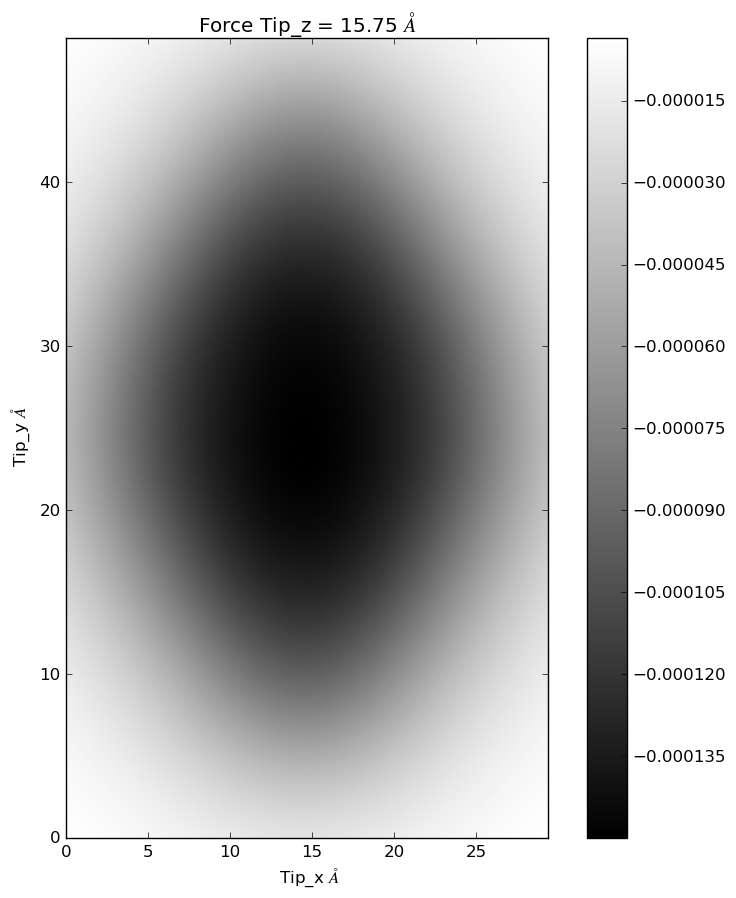

Supplement: File 9 — Datasets A0=5A k=0.5. [file Beilstein_J_Nanotechnol-07-937-s009.zip › S9/A0=5A/k=0.5/results/Force_0005.png]

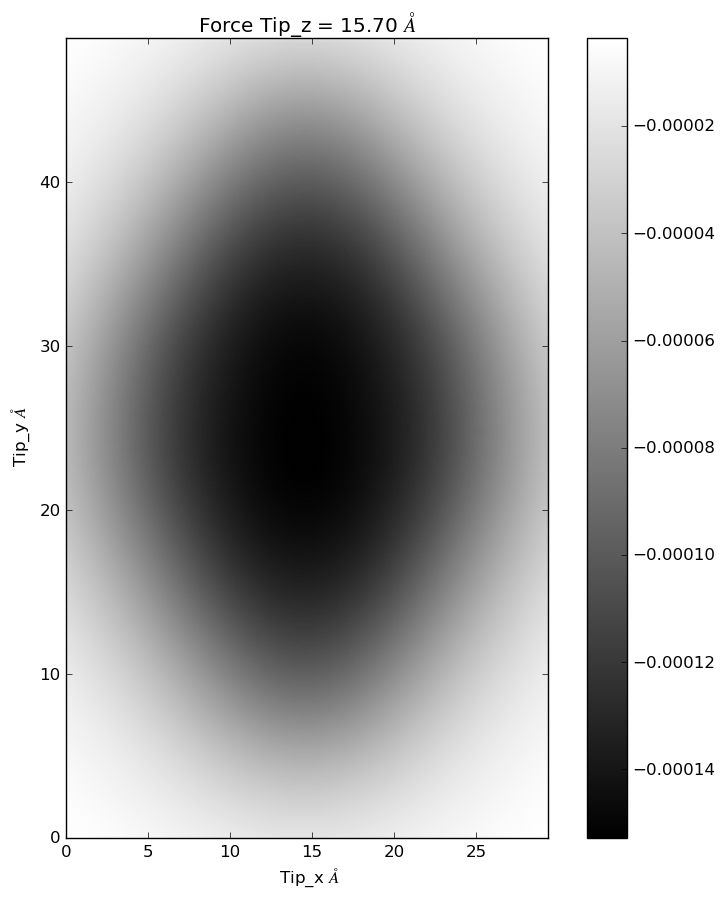

Supplement: File 9 — Datasets A0=5A k=0.5. [file Beilstein_J_Nanotechnol-07-937-s009.zip › S9/A0=5A/k=0.5/results/Force_0006.png]

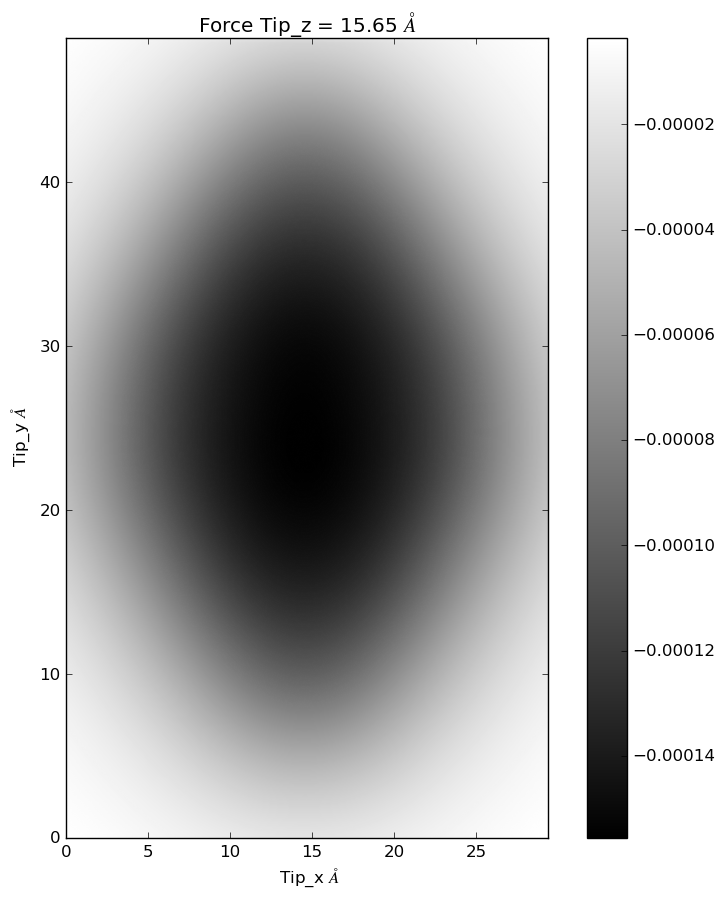

Supplement: File 9 — Datasets A0=5A k=0.5. [file Beilstein_J_Nanotechnol-07-937-s009.zip › S9/A0=5A/k=0.5/results/Force_0007.png]

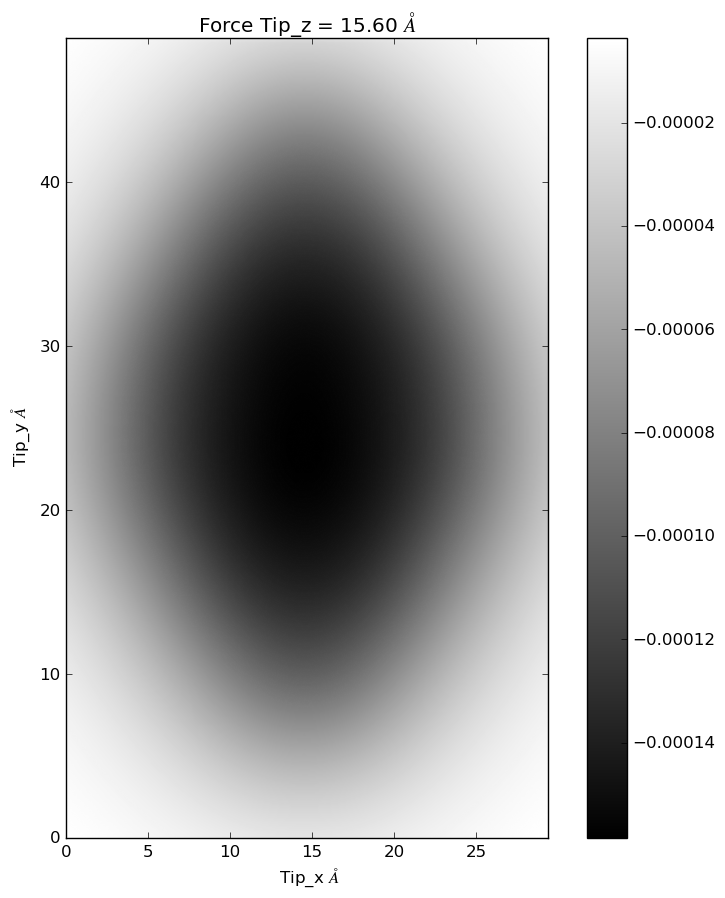

Supplement: File 9 — Datasets A0=5A k=0.5. [file Beilstein_J_Nanotechnol-07-937-s009.zip › S9/A0=5A/k=0.5/results/Force_0008.png]

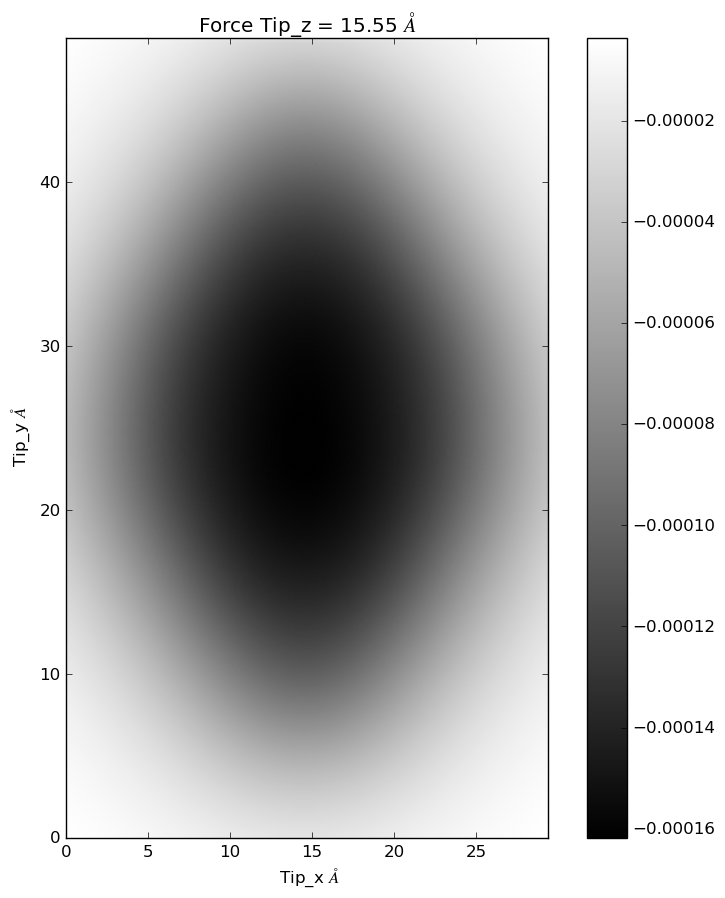

Supplement: File 9 — Datasets A0=5A k=0.5. [file Beilstein_J_Nanotechnol-07-937-s009.zip › S9/A0=5A/k=0.5/results/Force_0009.png]

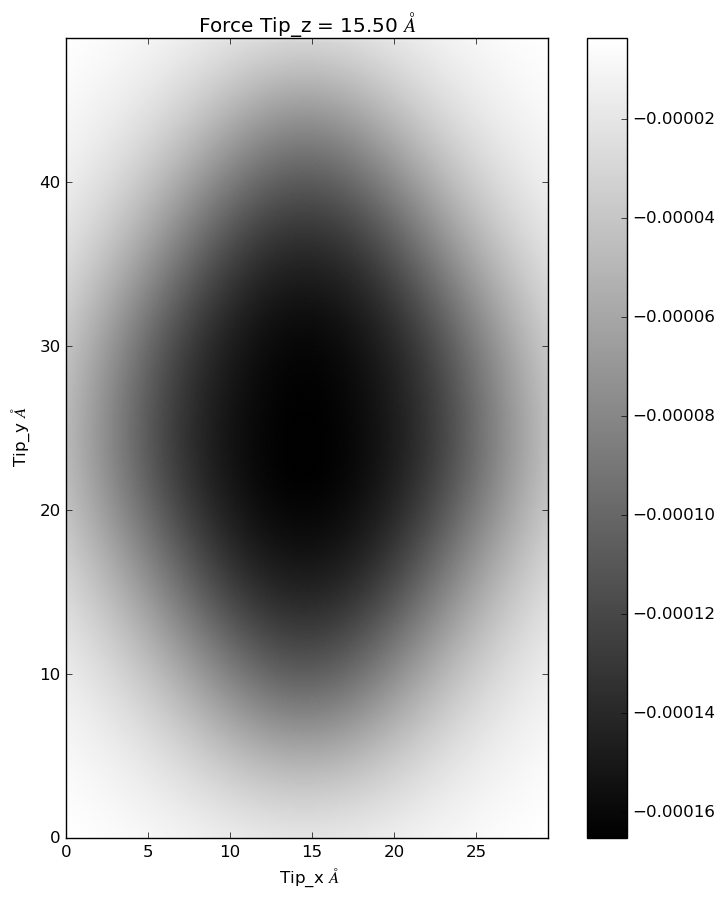

Supplement: File 9 — Datasets A0=5A k=0.5. [file Beilstein_J_Nanotechnol-07-937-s009.zip › S9/A0=5A/k=0.5/results/Force_0010.png]

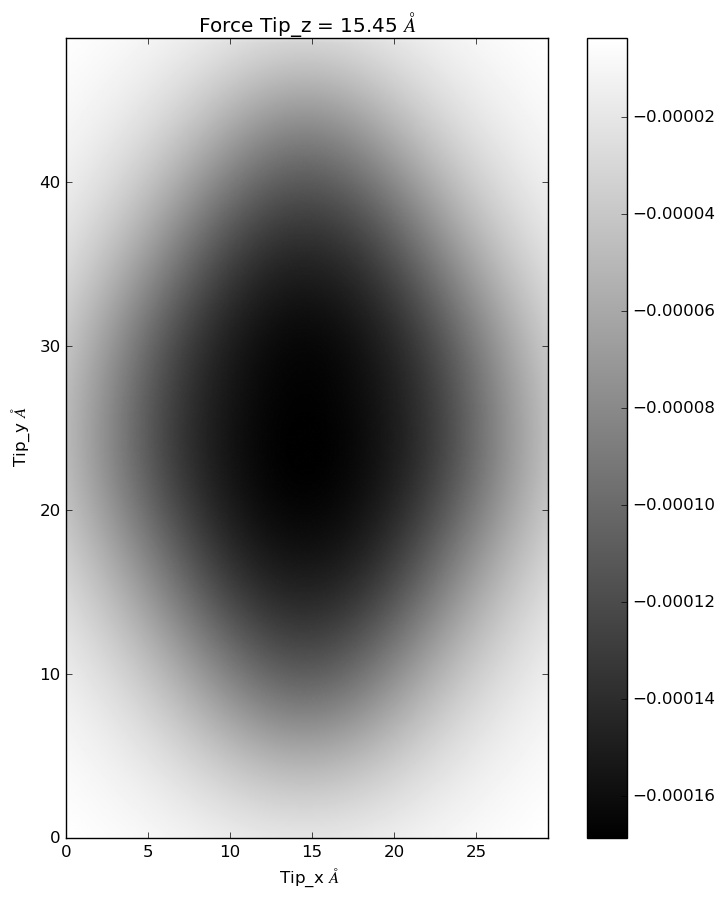

Supplement: File 9 — Datasets A0=5A k=0.5. [file Beilstein_J_Nanotechnol-07-937-s009.zip › S9/A0=5A/k=0.5/results/Force_0011.png]

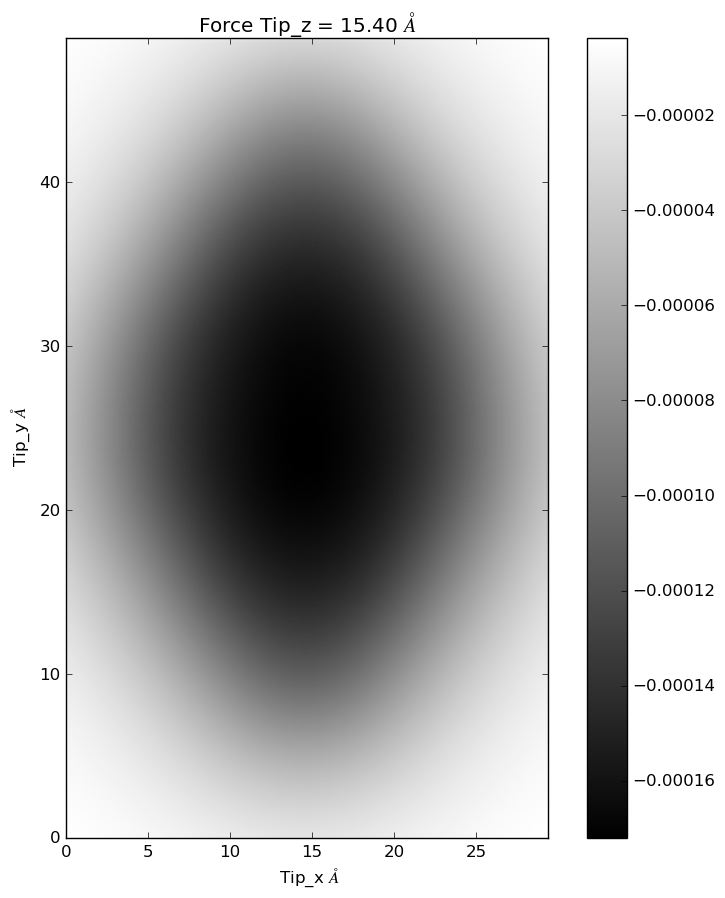

Supplement: File 9 — Datasets A0=5A k=0.5. [file Beilstein_J_Nanotechnol-07-937-s009.zip › S9/A0=5A/k=0.5/results/Force_0012.png]

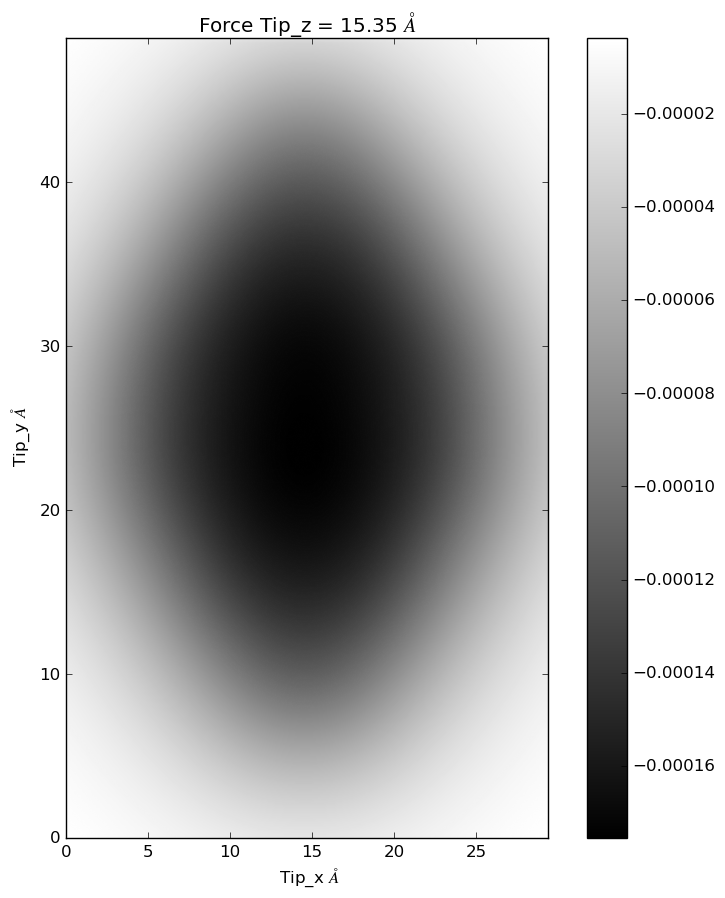

Supplement: File 9 — Datasets A0=5A k=0.5. [file Beilstein_J_Nanotechnol-07-937-s009.zip › S9/A0=5A/k=0.5/results/Force_0013.png]

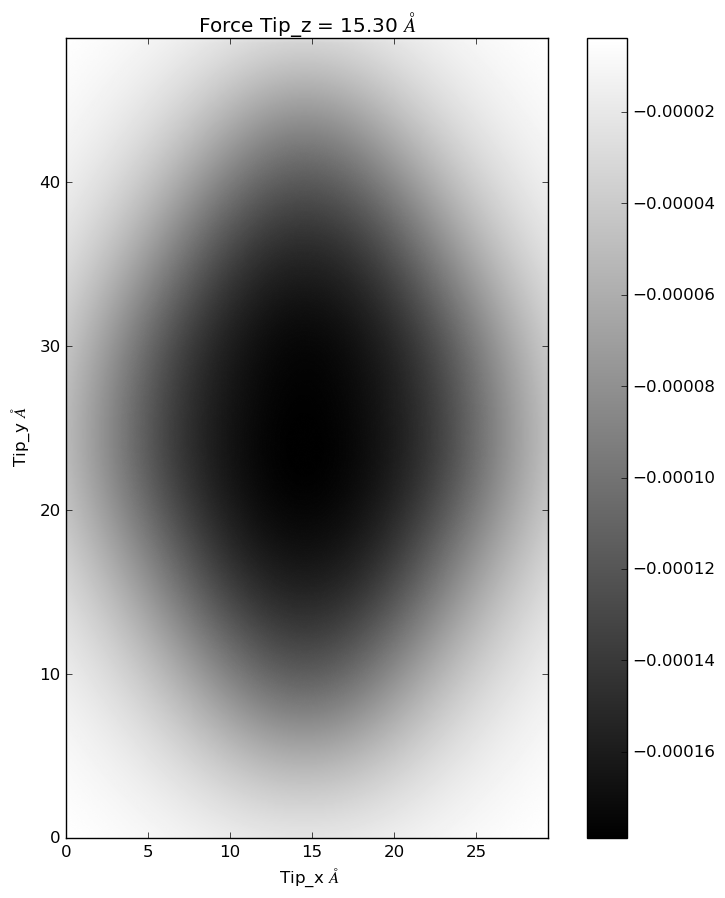

Supplement: File 9 — Datasets A0=5A k=0.5. [file Beilstein_J_Nanotechnol-07-937-s009.zip › S9/A0=5A/k=0.5/results/Force_0014.png]

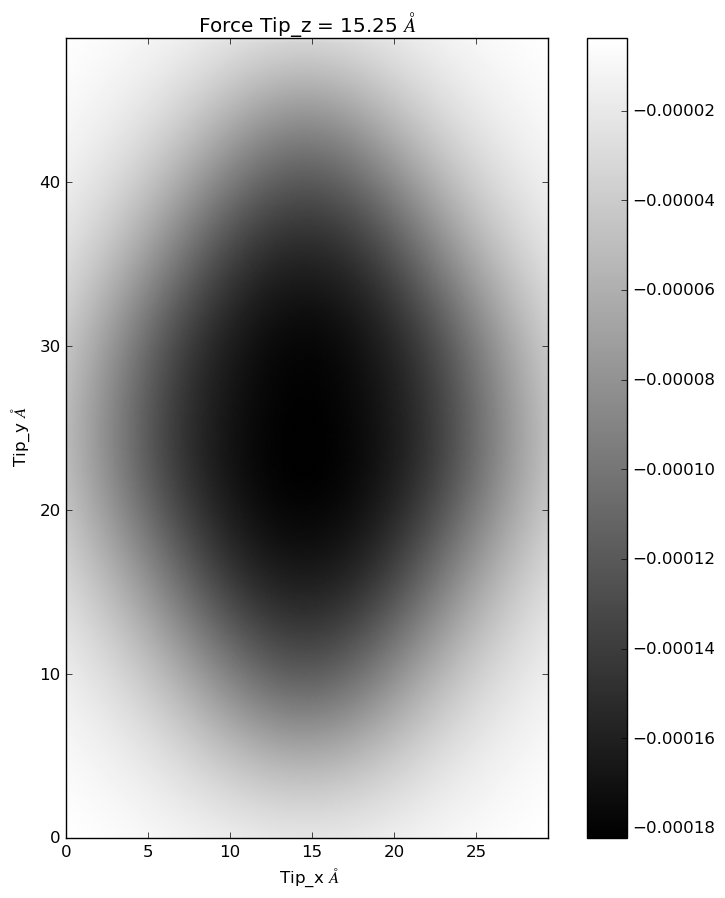

Supplement: File 9 — Datasets A0=5A k=0.5. [file Beilstein_J_Nanotechnol-07-937-s009.zip › S9/A0=5A/k=0.5/results/Force_0015.png]

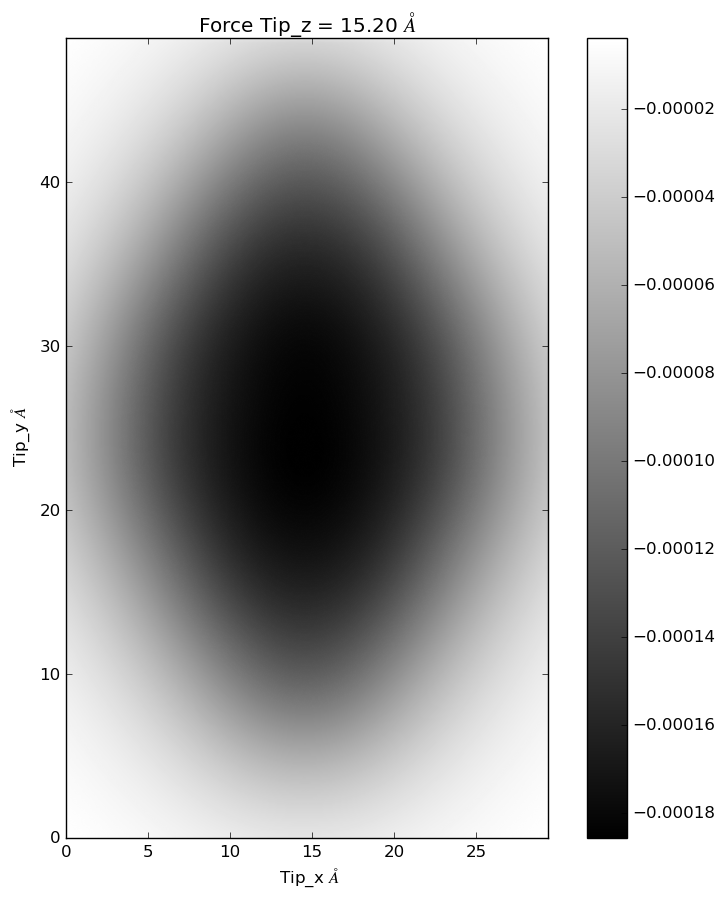

Supplement: File 9 — Datasets A0=5A k=0.5. [file Beilstein_J_Nanotechnol-07-937-s009.zip › S9/A0=5A/k=0.5/results/Force_0016.png]

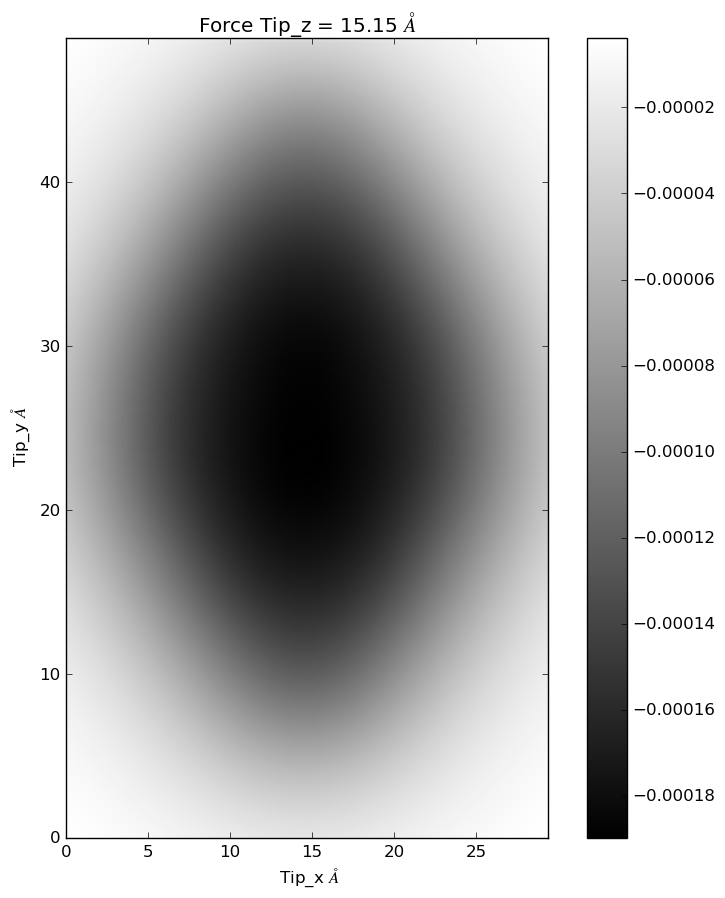

Supplement: File 9 — Datasets A0=5A k=0.5. [file Beilstein_J_Nanotechnol-07-937-s009.zip › S9/A0=5A/k=0.5/results/Force_0017.png]

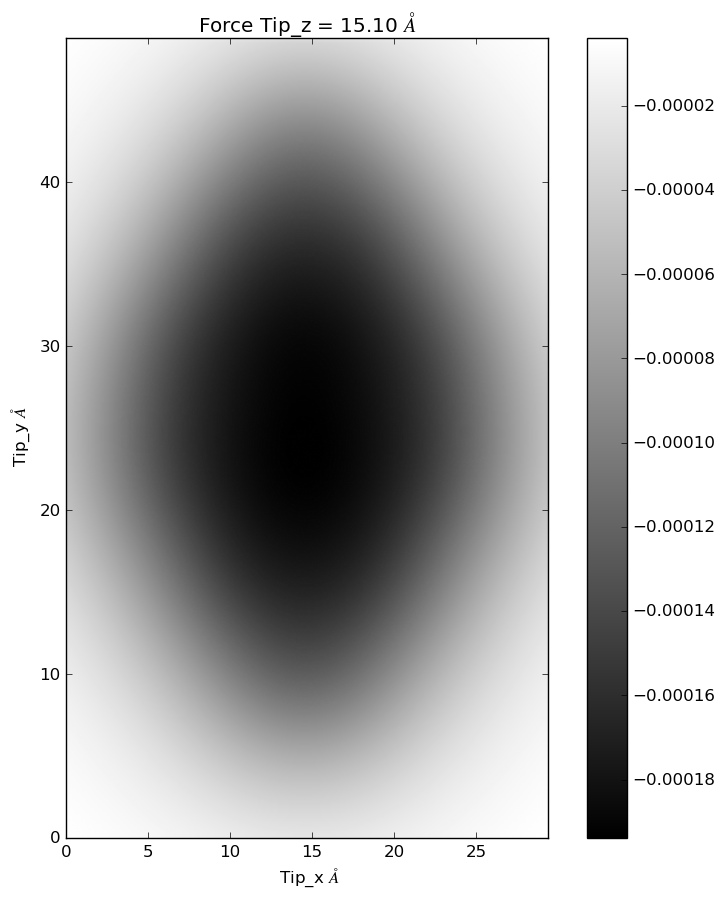

Supplement: File 9 — Datasets A0=5A k=0.5. [file Beilstein_J_Nanotechnol-07-937-s009.zip › S9/A0=5A/k=0.5/results/Force_0018.png]

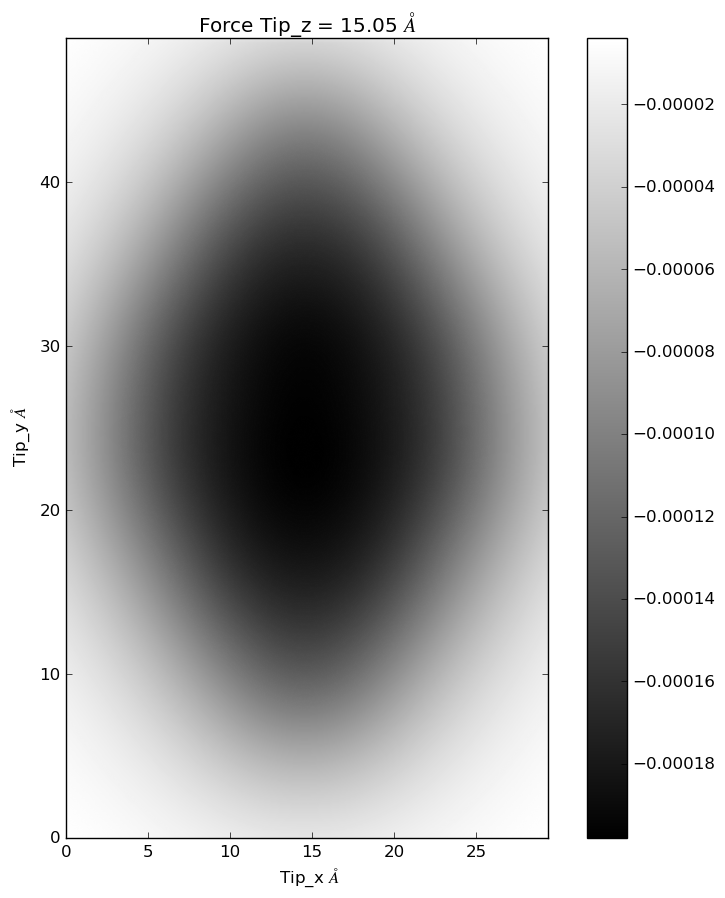

Supplement: File 9 — Datasets A0=5A k=0.5. [file Beilstein_J_Nanotechnol-07-937-s009.zip › S9/A0=5A/k=0.5/results/Force_0019.png]

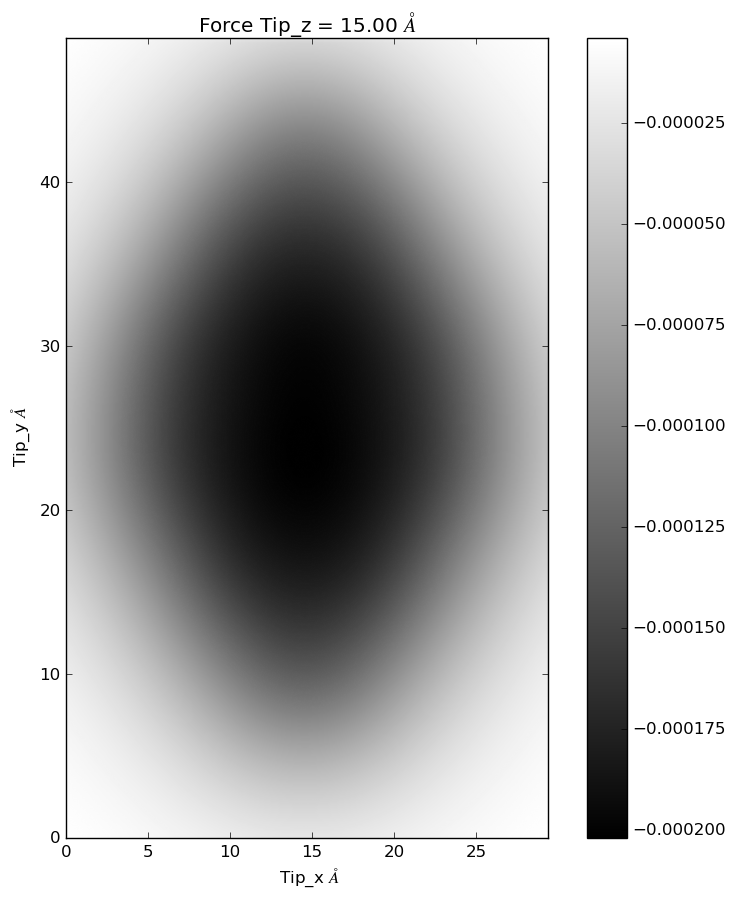

Supplement: File 9 — Datasets A0=5A k=0.5. [file Beilstein_J_Nanotechnol-07-937-s009.zip › S9/A0=5A/k=0.5/results/Force_0020.png]

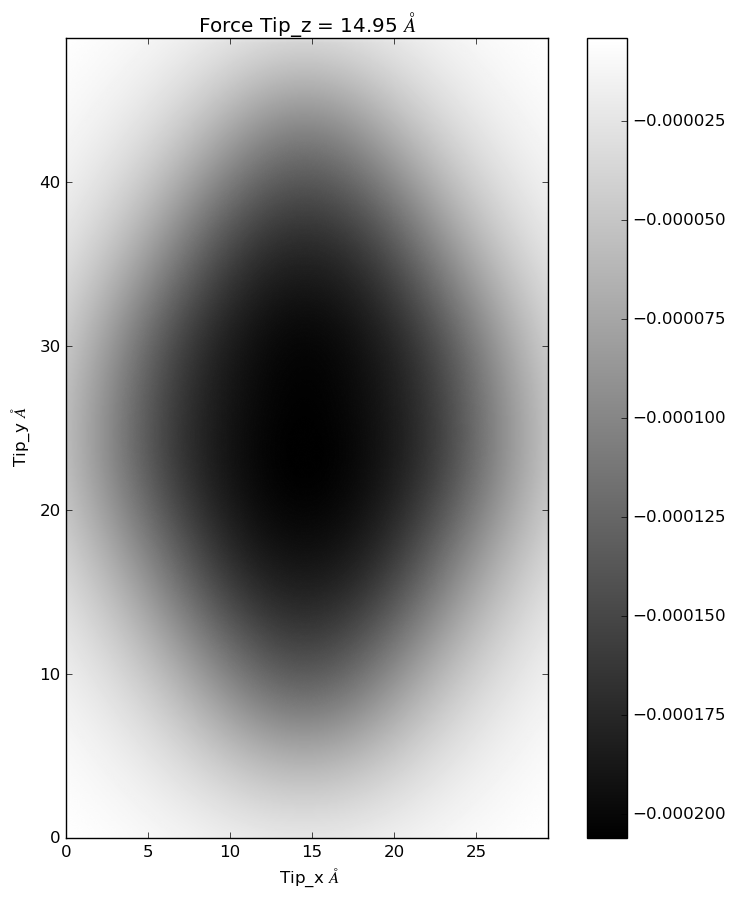

Supplement: File 9 — Datasets A0=5A k=0.5. [file Beilstein_J_Nanotechnol-07-937-s009.zip › S9/A0=5A/k=0.5/results/Force_0021.png]

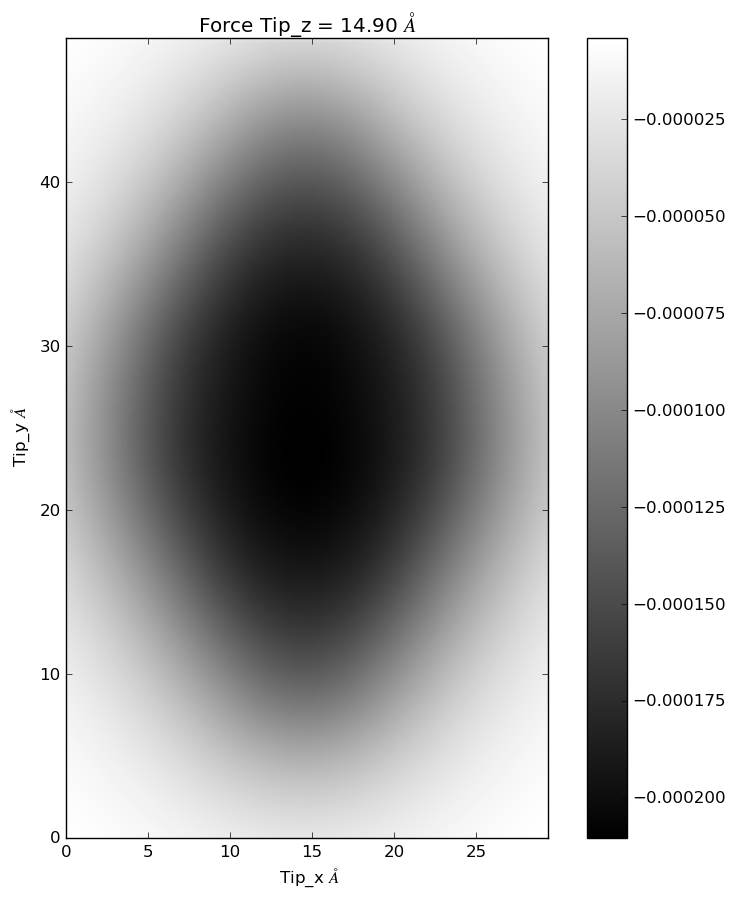

Supplement: File 9 — Datasets A0=5A k=0.5. [file Beilstein_J_Nanotechnol-07-937-s009.zip › S9/A0=5A/k=0.5/results/Force_0022.png]

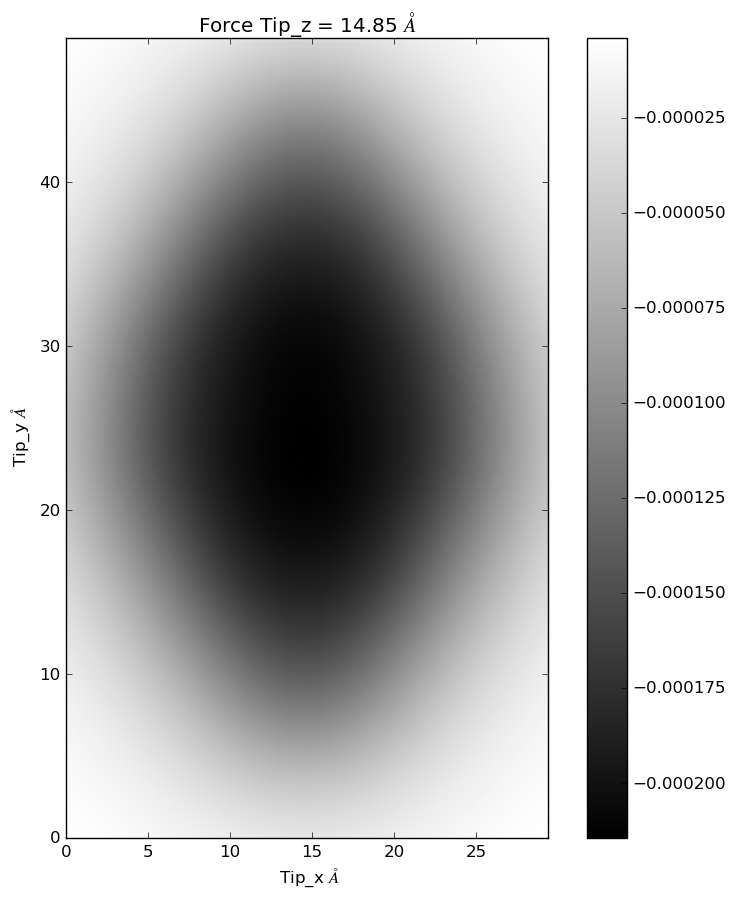

Supplement: File 9 — Datasets A0=5A k=0.5. [file Beilstein_J_Nanotechnol-07-937-s009.zip › S9/A0=5A/k=0.5/results/Force_0023.png]

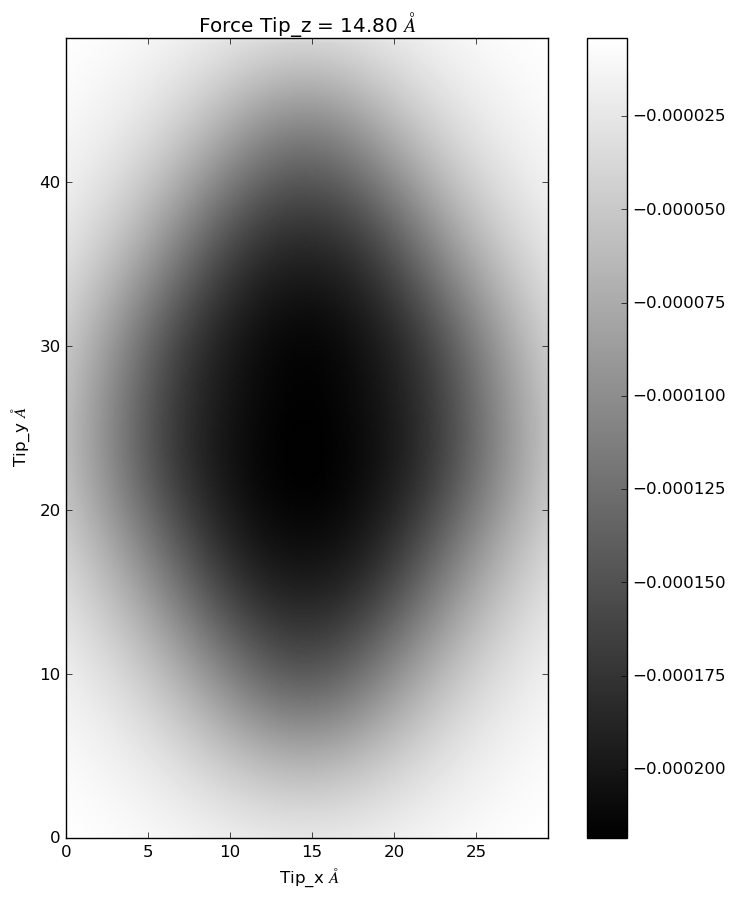

Supplement: File 9 — Datasets A0=5A k=0.5. [file Beilstein_J_Nanotechnol-07-937-s009.zip › S9/A0=5A/k=0.5/results/Force_0024.png]

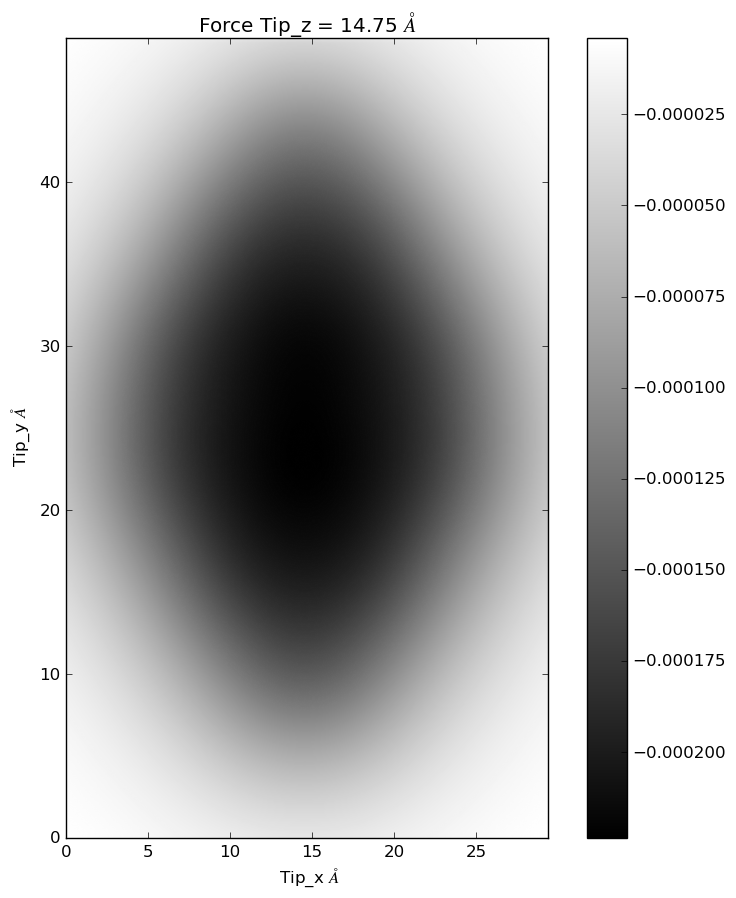

Supplement: File 9 — Datasets A0=5A k=0.5. [file Beilstein_J_Nanotechnol-07-937-s009.zip › S9/A0=5A/k=0.5/results/Force_0025.png]

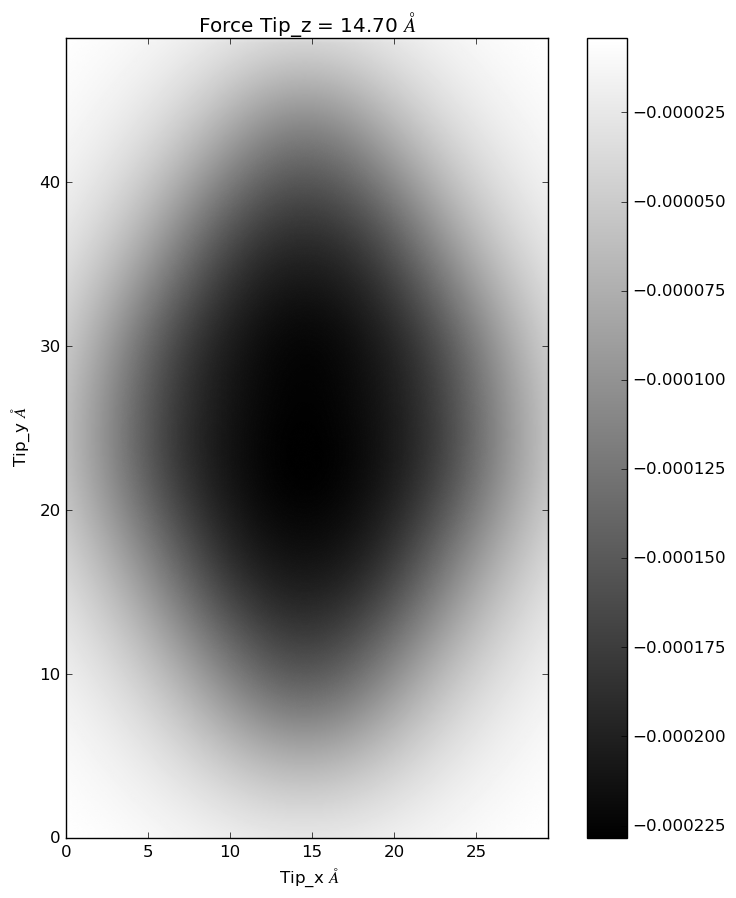

Supplement: File 9 — Datasets A0=5A k=0.5. [file Beilstein_J_Nanotechnol-07-937-s009.zip › S9/A0=5A/k=0.5/results/Force_0026.png]

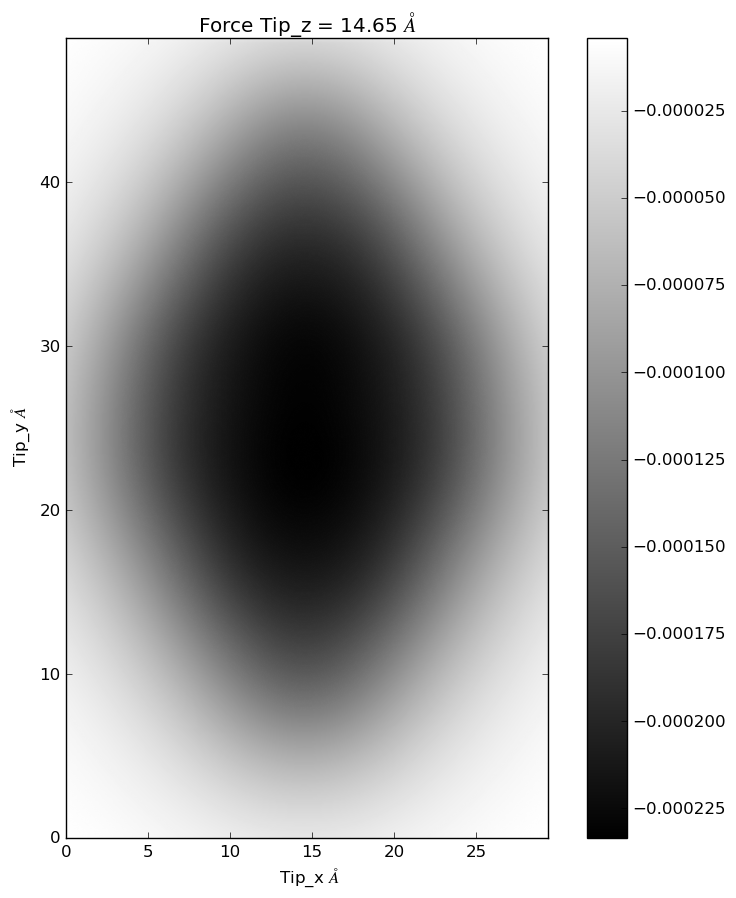

Supplement: File 9 — Datasets A0=5A k=0.5. [file Beilstein_J_Nanotechnol-07-937-s009.zip › S9/A0=5A/k=0.5/results/Force_0027.png]

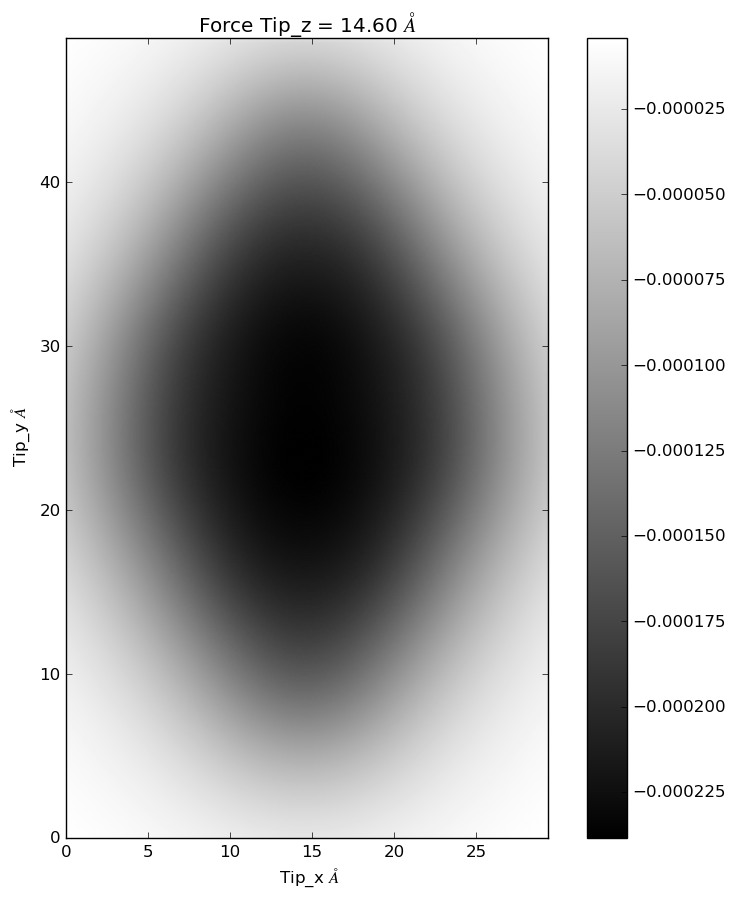

Supplement: File 9 — Datasets A0=5A k=0.5. [file Beilstein_J_Nanotechnol-07-937-s009.zip › S9/A0=5A/k=0.5/results/Force_0028.png]

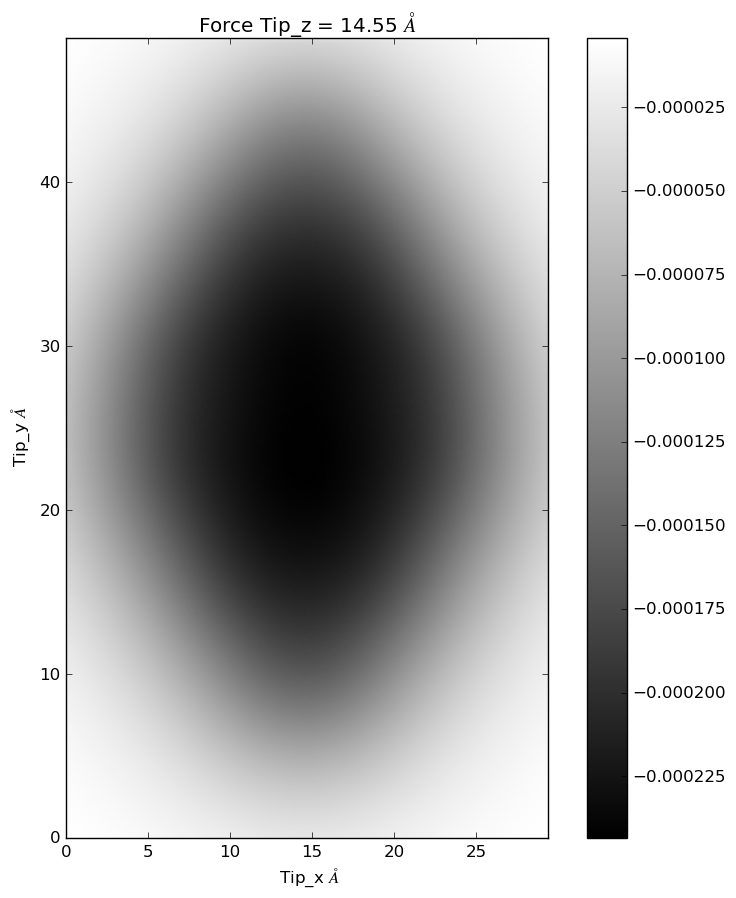

Supplement: File 9 — Datasets A0=5A k=0.5. [file Beilstein_J_Nanotechnol-07-937-s009.zip › S9/A0=5A/k=0.5/results/Force_0029.png]

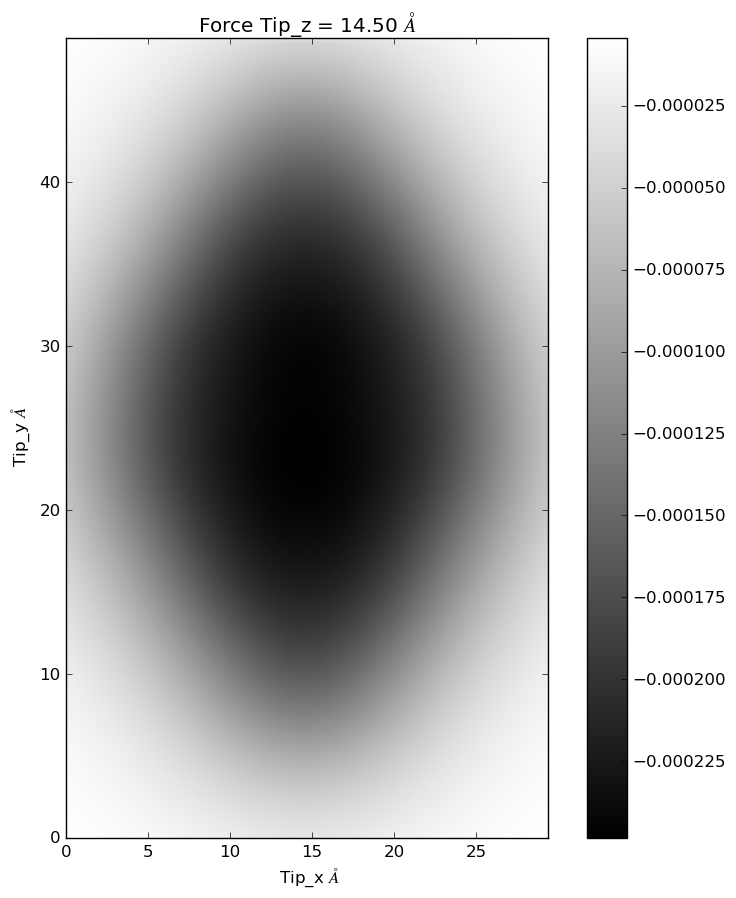

Supplement: File 9 — Datasets A0=5A k=0.5. [file Beilstein_J_Nanotechnol-07-937-s009.zip › S9/A0=5A/k=0.5/results/Force_0030.png]

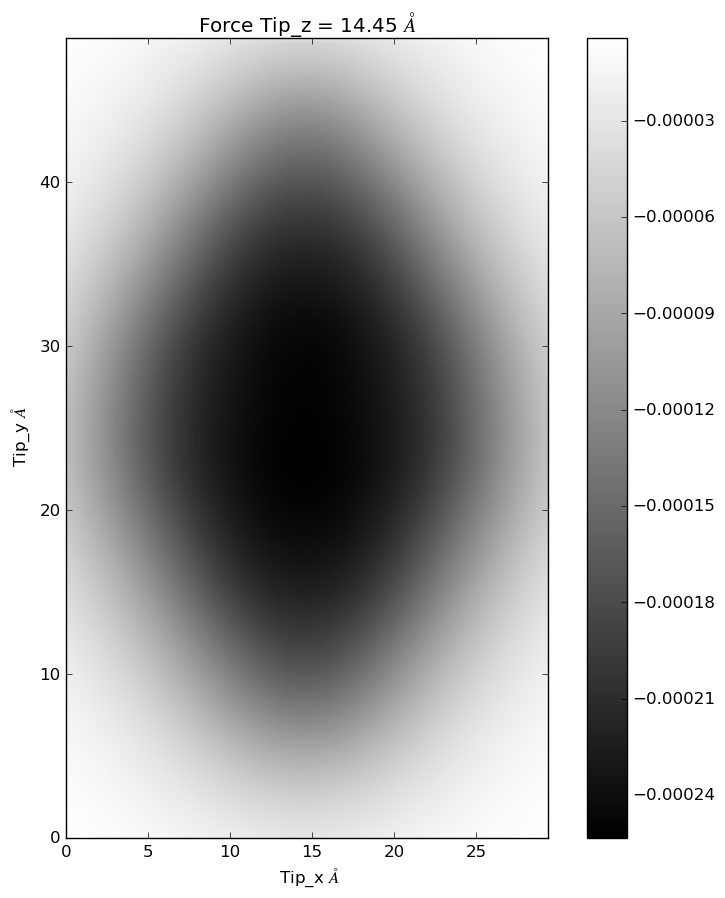

Supplement: File 9 — Datasets A0=5A k=0.5. [file Beilstein_J_Nanotechnol-07-937-s009.zip › S9/A0=5A/k=0.5/results/Force_0031.png]

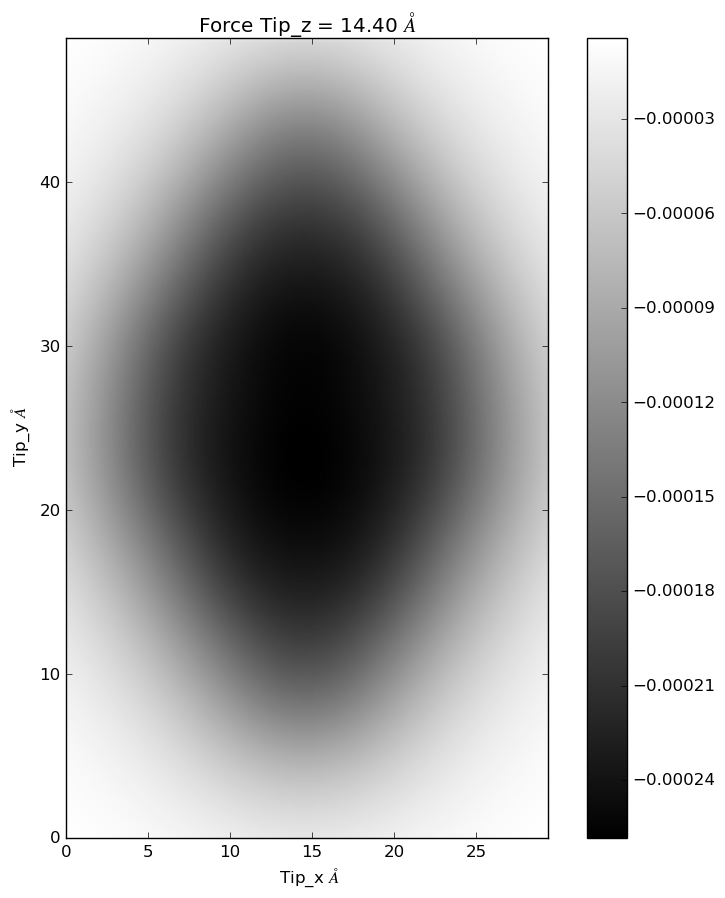

Supplement: File 9 — Datasets A0=5A k=0.5. [file Beilstein_J_Nanotechnol-07-937-s009.zip › S9/A0=5A/k=0.5/results/Force_0032.png]

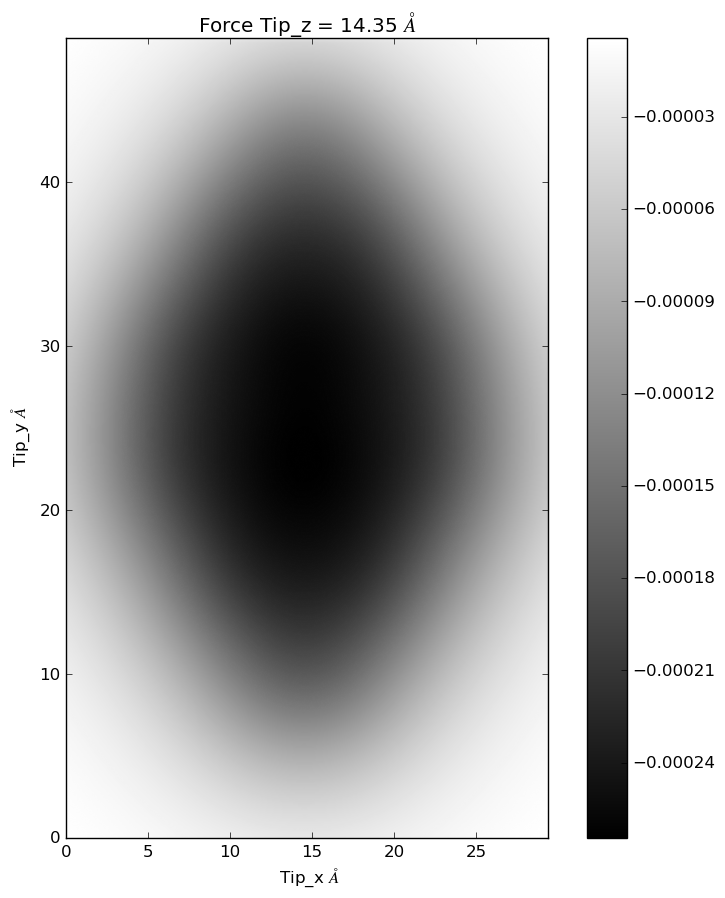

Supplement: File 9 — Datasets A0=5A k=0.5. [file Beilstein_J_Nanotechnol-07-937-s009.zip › S9/A0=5A/k=0.5/results/Force_0033.png]

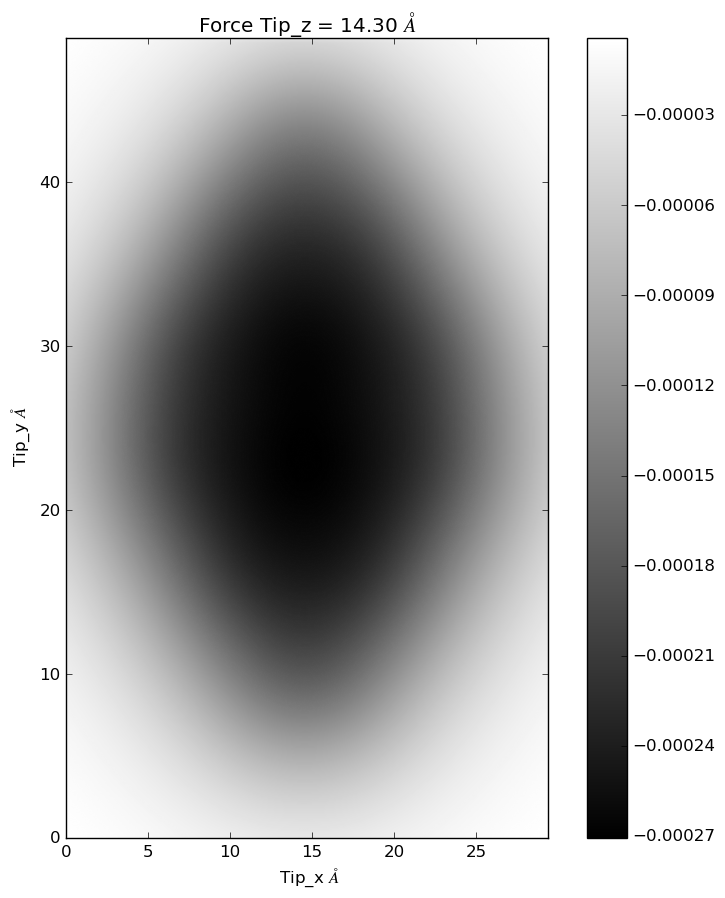

Supplement: File 9 — Datasets A0=5A k=0.5. [file Beilstein_J_Nanotechnol-07-937-s009.zip › S9/A0=5A/k=0.5/results/Force_0034.png]

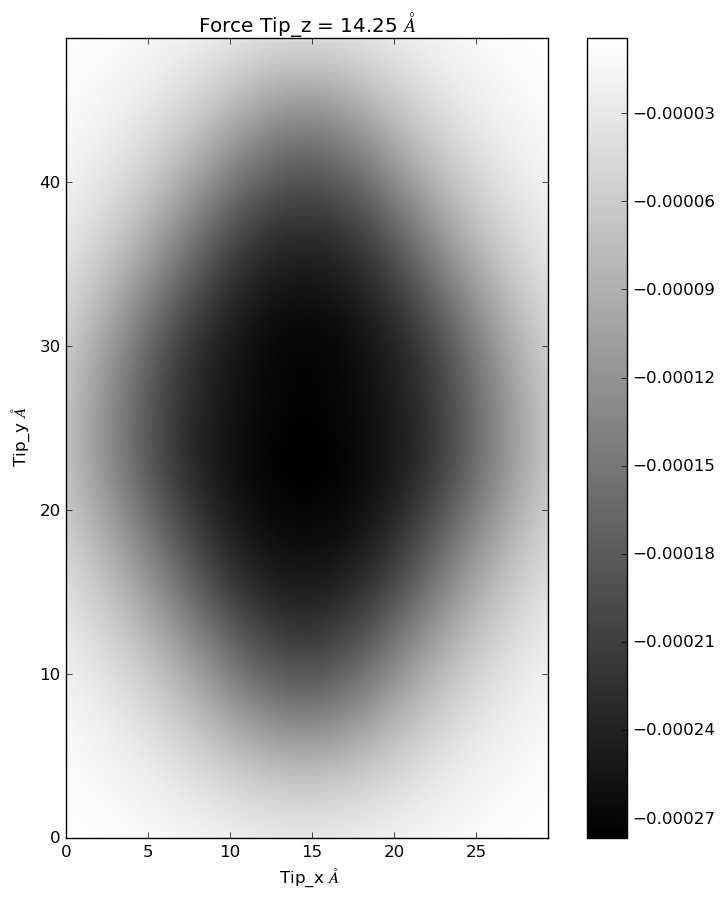

Supplement: File 9 — Datasets A0=5A k=0.5. [file Beilstein_J_Nanotechnol-07-937-s009.zip › S9/A0=5A/k=0.5/results/Force_0035.png]

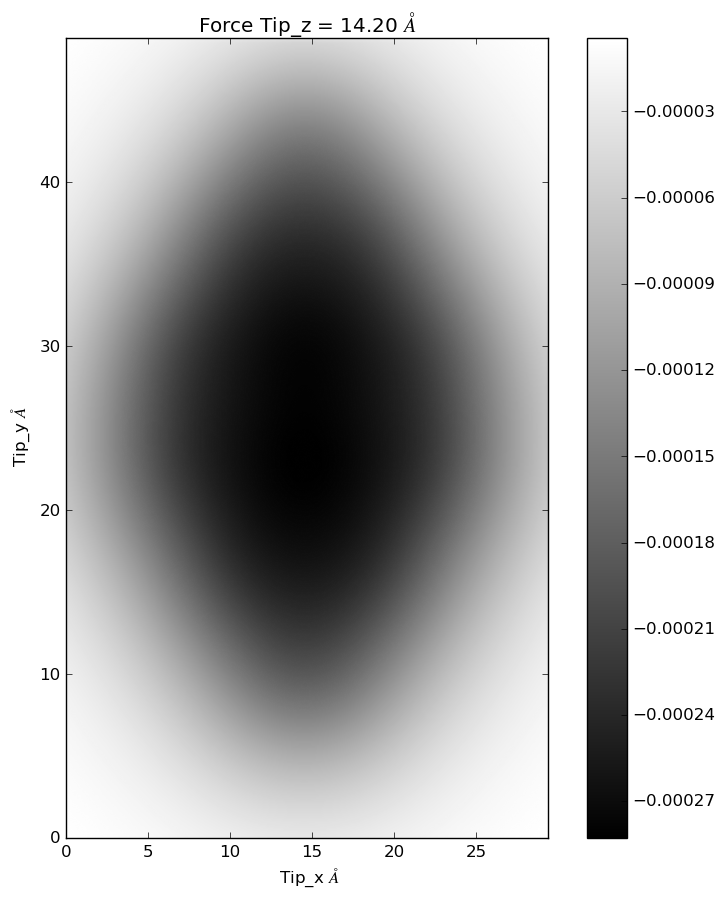

Supplement: File 9 — Datasets A0=5A k=0.5. [file Beilstein_J_Nanotechnol-07-937-s009.zip › S9/A0=5A/k=0.5/results/Force_0036.png]

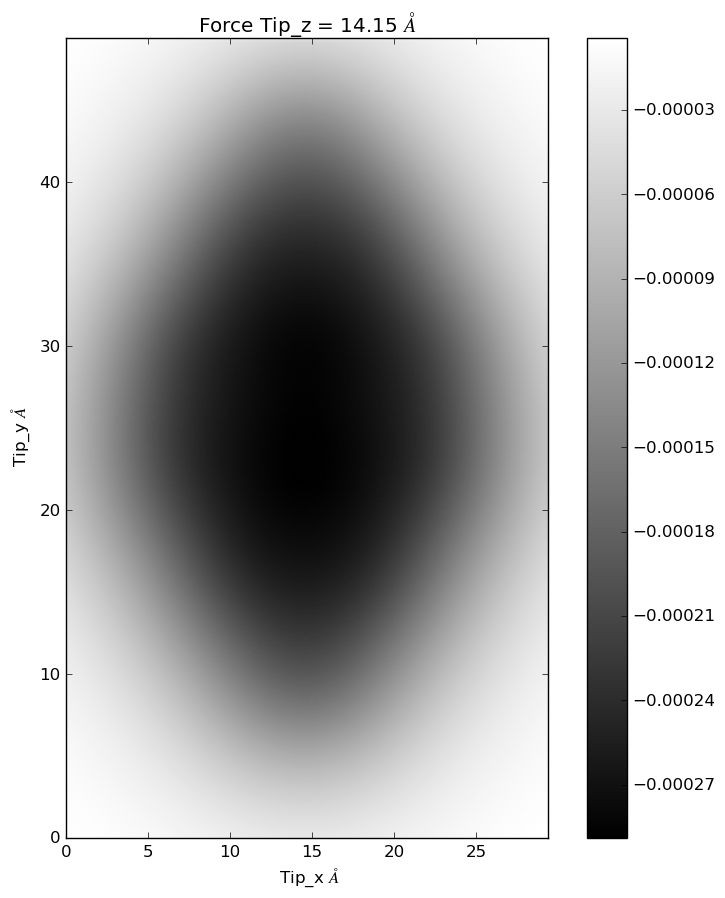

Supplement: File 9 — Datasets A0=5A k=0.5. [file Beilstein_J_Nanotechnol-07-937-s009.zip › S9/A0=5A/k=0.5/results/Force_0037.png]

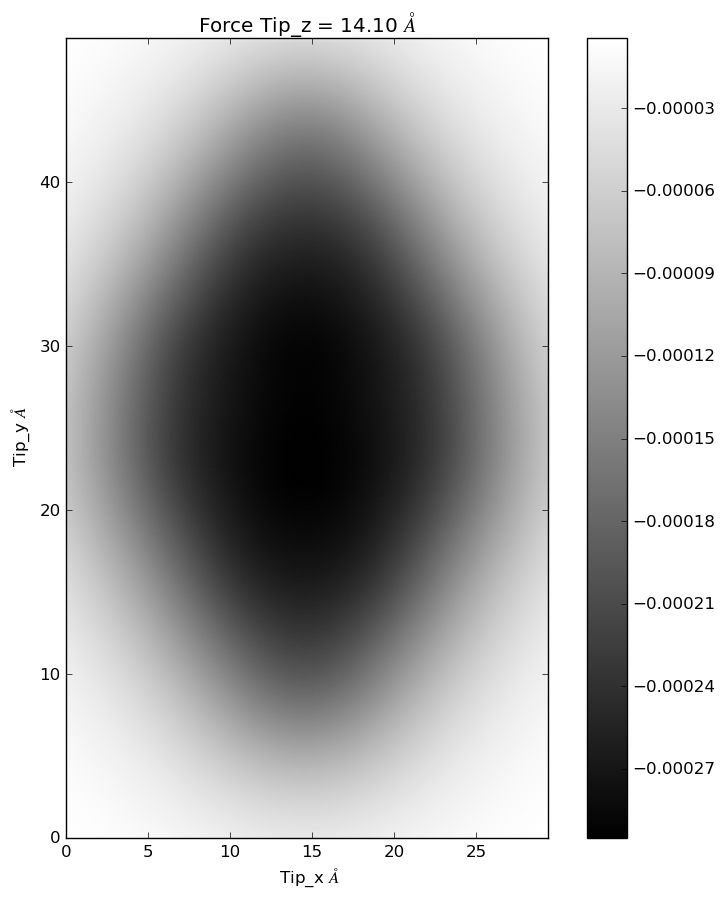

Supplement: File 9 — Datasets A0=5A k=0.5. [file Beilstein_J_Nanotechnol-07-937-s009.zip › S9/A0=5A/k=0.5/results/Force_0038.png]

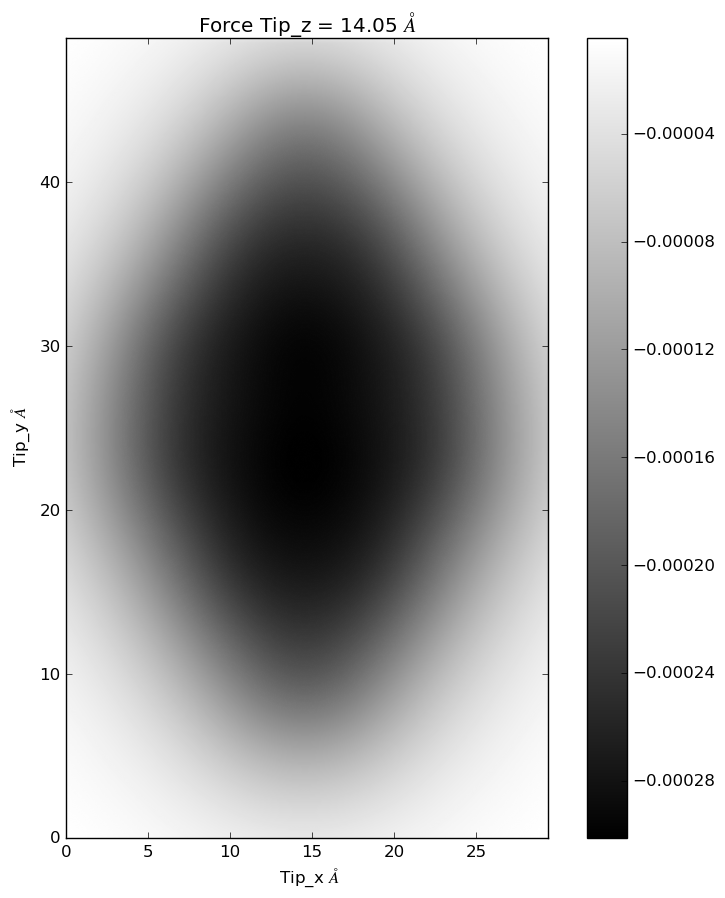

Supplement: File 9 — Datasets A0=5A k=0.5. [file Beilstein_J_Nanotechnol-07-937-s009.zip › S9/A0=5A/k=0.5/results/Force_0039.png]

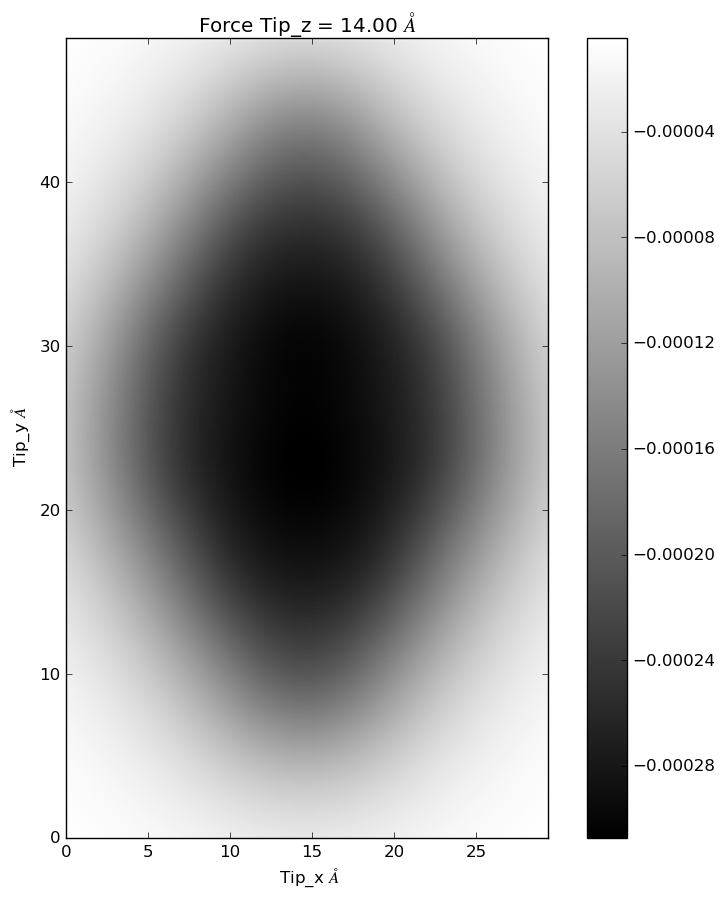

Supplement: File 9 — Datasets A0=5A k=0.5. [file Beilstein_J_Nanotechnol-07-937-s009.zip › S9/A0=5A/k=0.5/results/Force_0040.png]

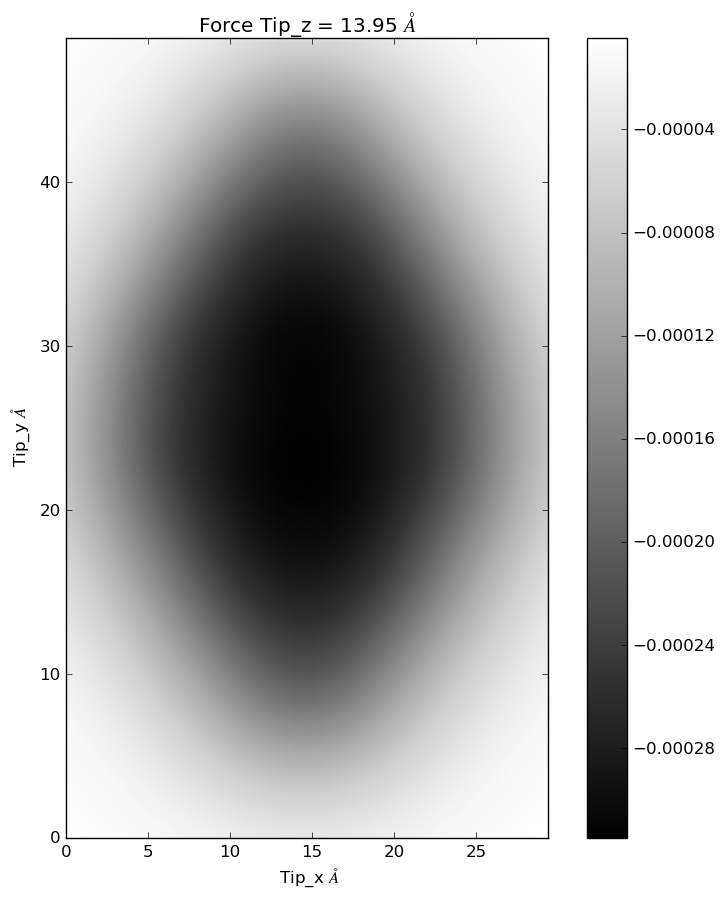

Supplement: File 9 — Datasets A0=5A k=0.5. [file Beilstein_J_Nanotechnol-07-937-s009.zip › S9/A0=5A/k=0.5/results/Force_0041.png]

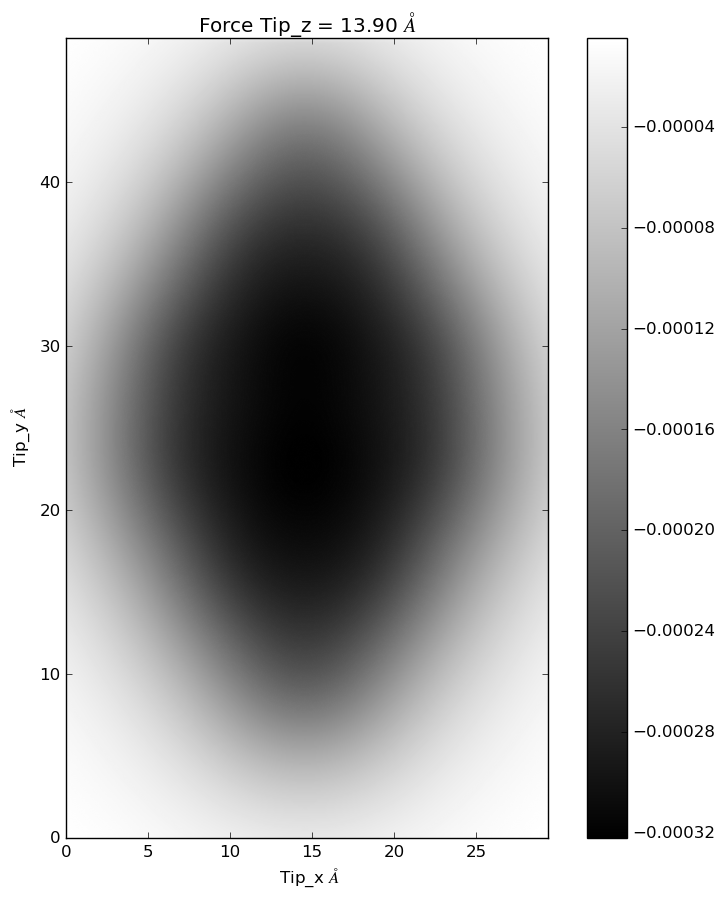

Supplement: File 9 — Datasets A0=5A k=0.5. [file Beilstein_J_Nanotechnol-07-937-s009.zip › S9/A0=5A/k=0.5/results/Force_0042.png]

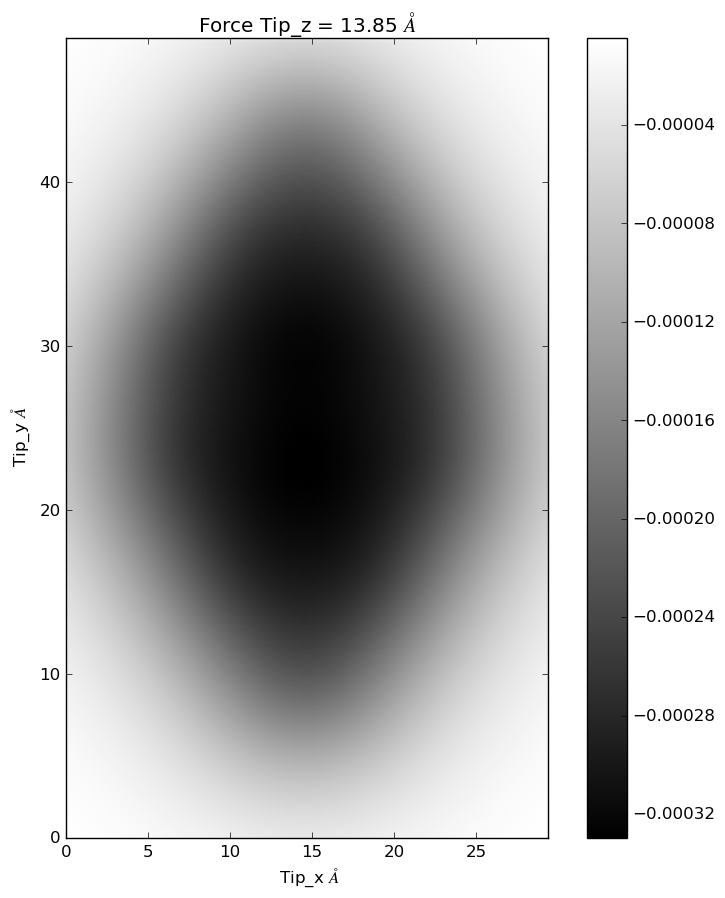

Supplement: File 9 — Datasets A0=5A k=0.5. [file Beilstein_J_Nanotechnol-07-937-s009.zip › S9/A0=5A/k=0.5/results/Force_0043.png]

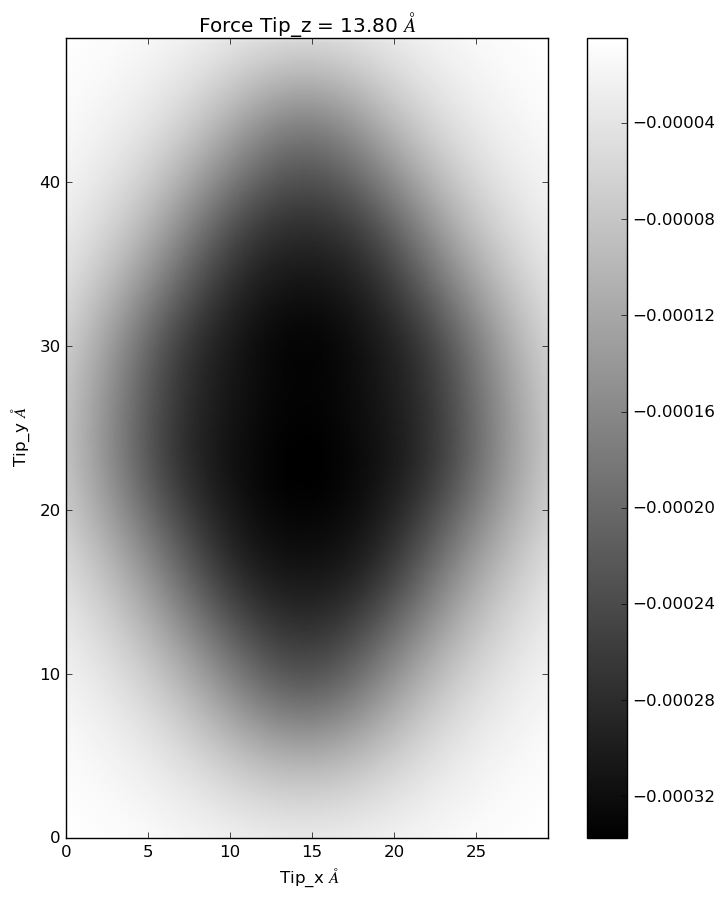

Supplement: File 9 — Datasets A0=5A k=0.5. [file Beilstein_J_Nanotechnol-07-937-s009.zip › S9/A0=5A/k=0.5/results/Force_0044.png]

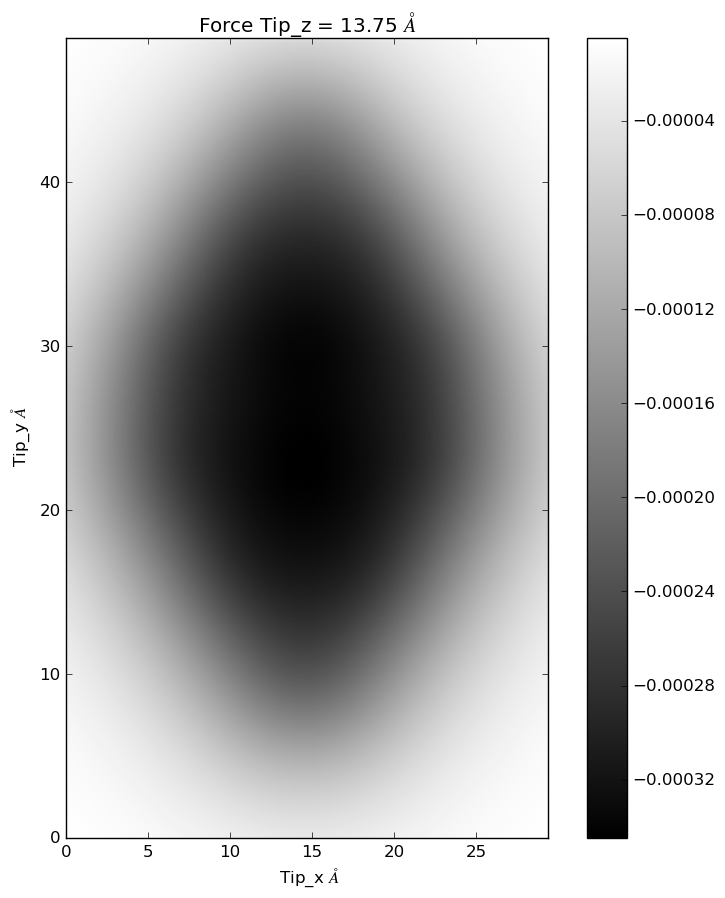

Supplement: File 9 — Datasets A0=5A k=0.5. [file Beilstein_J_Nanotechnol-07-937-s009.zip › S9/A0=5A/k=0.5/results/Force_0045.png]

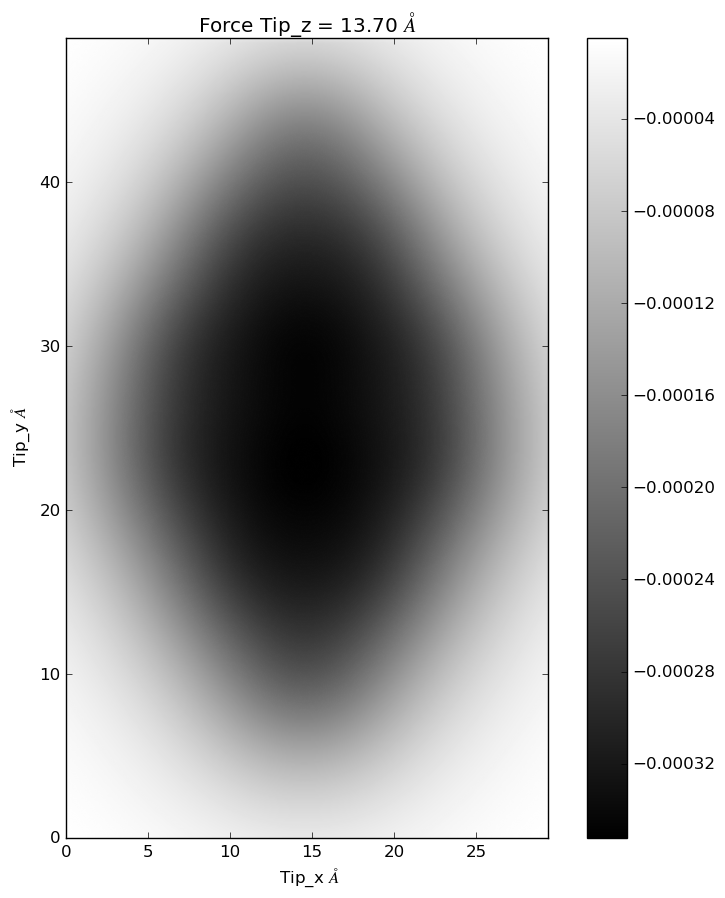

Supplement: File 9 — Datasets A0=5A k=0.5. [file Beilstein_J_Nanotechnol-07-937-s009.zip › S9/A0=5A/k=0.5/results/Force_0046.png]

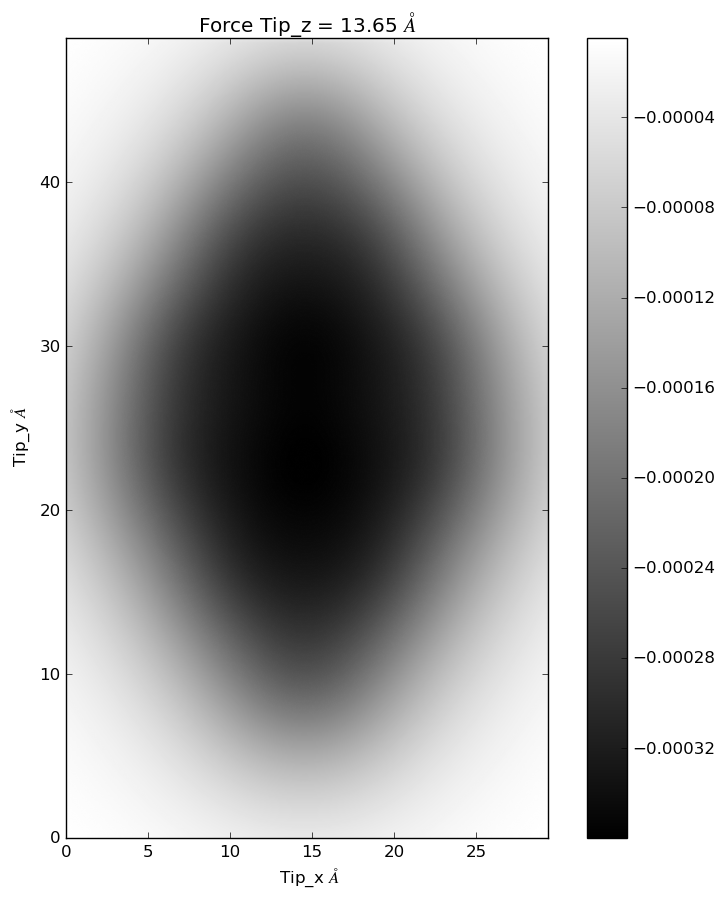

Supplement: File 9 — Datasets A0=5A k=0.5. [file Beilstein_J_Nanotechnol-07-937-s009.zip › S9/A0=5A/k=0.5/results/Force_0047.png]

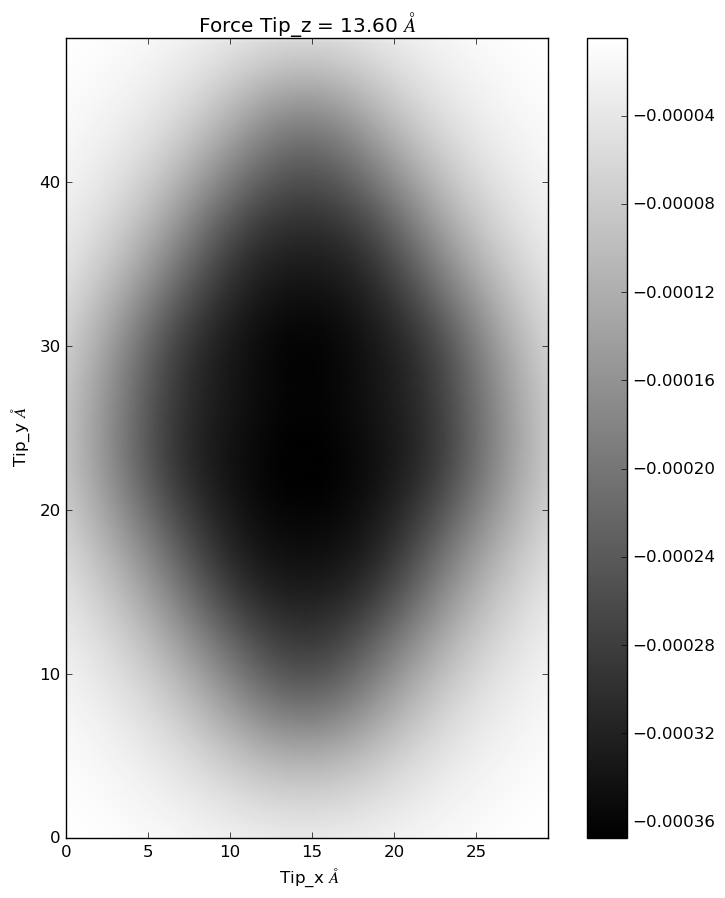

Supplement: File 9 — Datasets A0=5A k=0.5. [file Beilstein_J_Nanotechnol-07-937-s009.zip › S9/A0=5A/k=0.5/results/Force_0048.png]

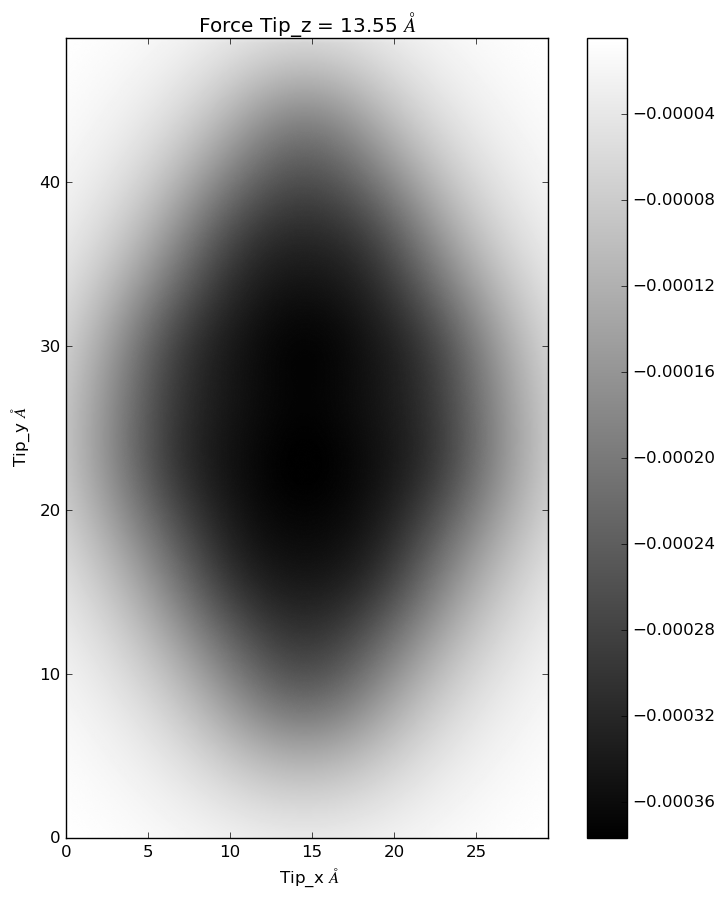

Supplement: File 9 — Datasets A0=5A k=0.5. [file Beilstein_J_Nanotechnol-07-937-s009.zip › S9/A0=5A/k=0.5/results/Force_0049.png]

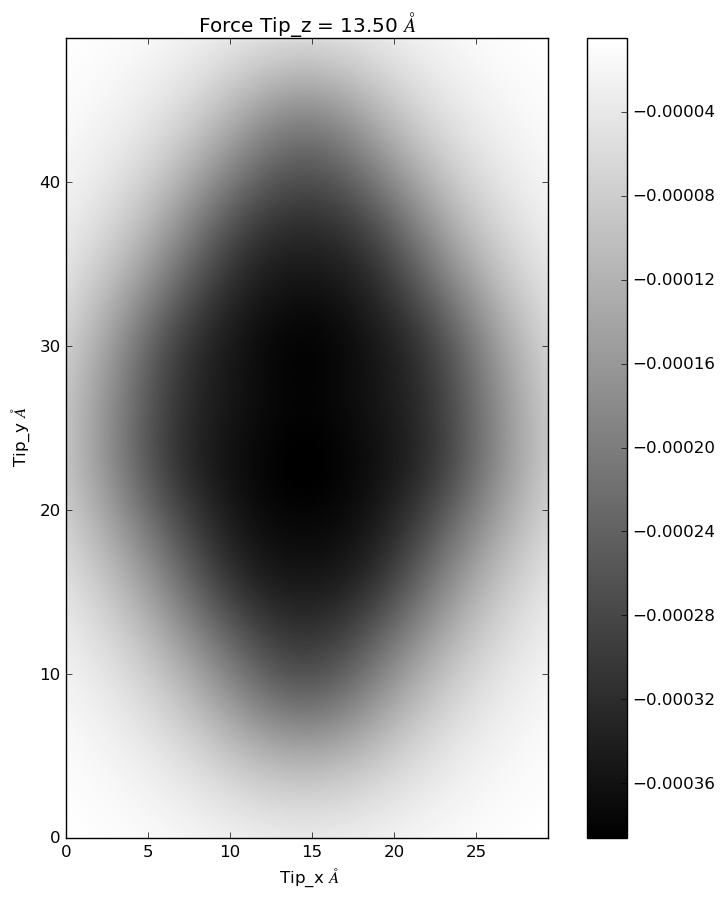

Supplement: File 9 — Datasets A0=5A k=0.5. [file Beilstein_J_Nanotechnol-07-937-s009.zip › S9/A0=5A/k=0.5/results/Force_0050.png]

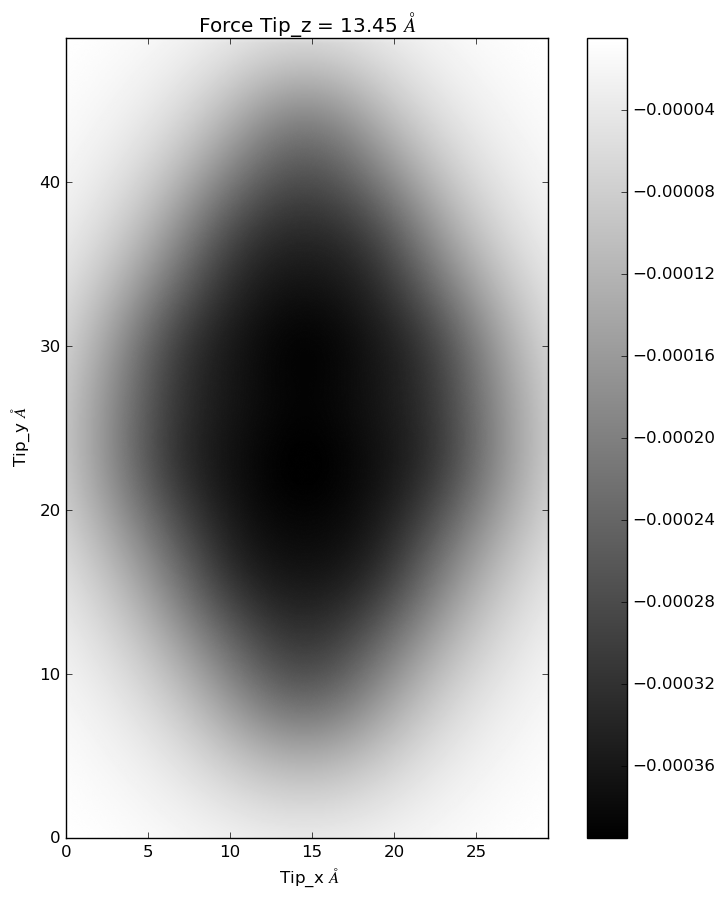

Supplement: File 9 — Datasets A0=5A k=0.5. [file Beilstein_J_Nanotechnol-07-937-s009.zip › S9/A0=5A/k=0.5/results/Force_0051.png]

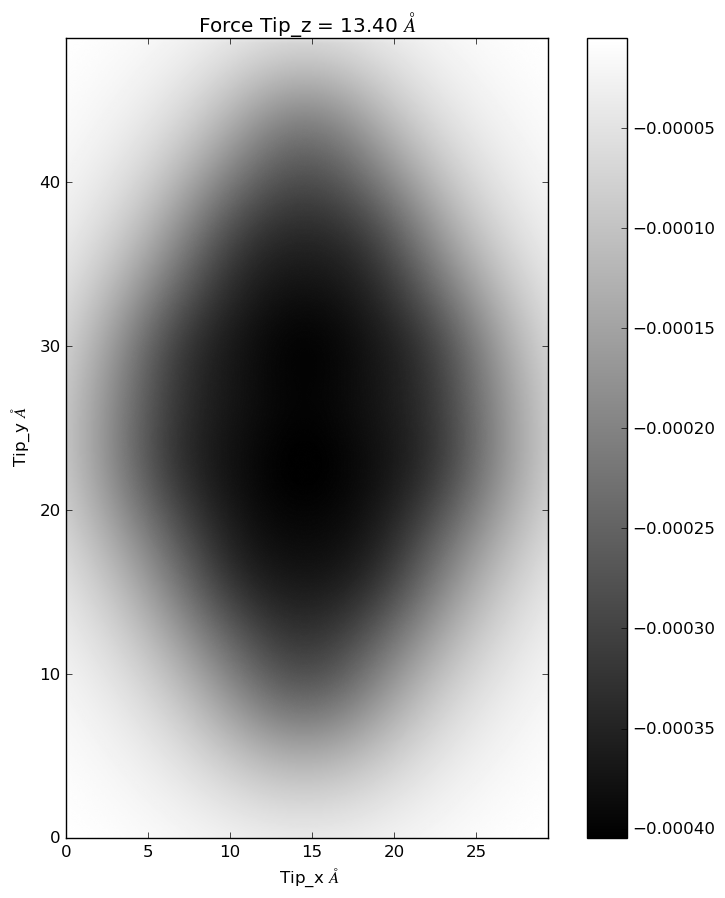

Supplement: File 9 — Datasets A0=5A k=0.5. [file Beilstein_J_Nanotechnol-07-937-s009.zip › S9/A0=5A/k=0.5/results/Force_0052.png]

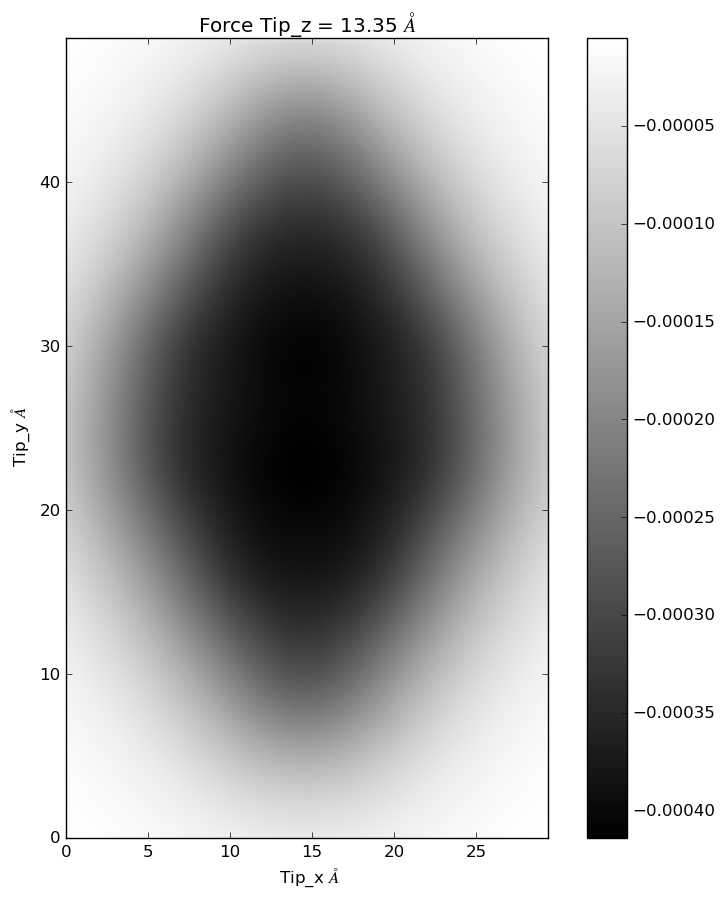

Supplement: File 9 — Datasets A0=5A k=0.5. [file Beilstein_J_Nanotechnol-07-937-s009.zip › S9/A0=5A/k=0.5/results/Force_0053.png]

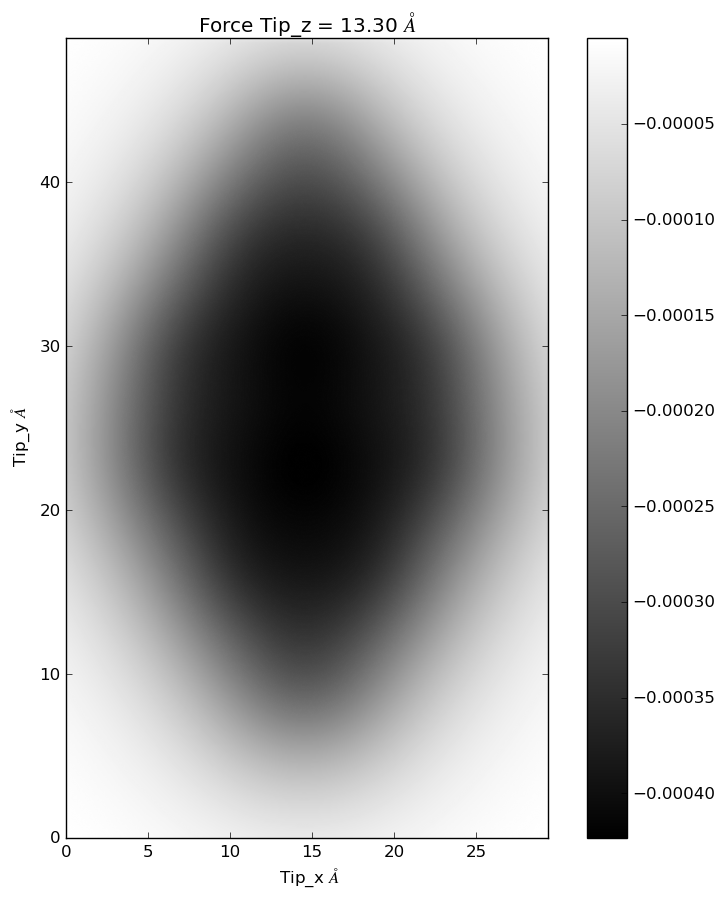

Supplement: File 9 — Datasets A0=5A k=0.5. [file Beilstein_J_Nanotechnol-07-937-s009.zip › S9/A0=5A/k=0.5/results/Force_0054.png]

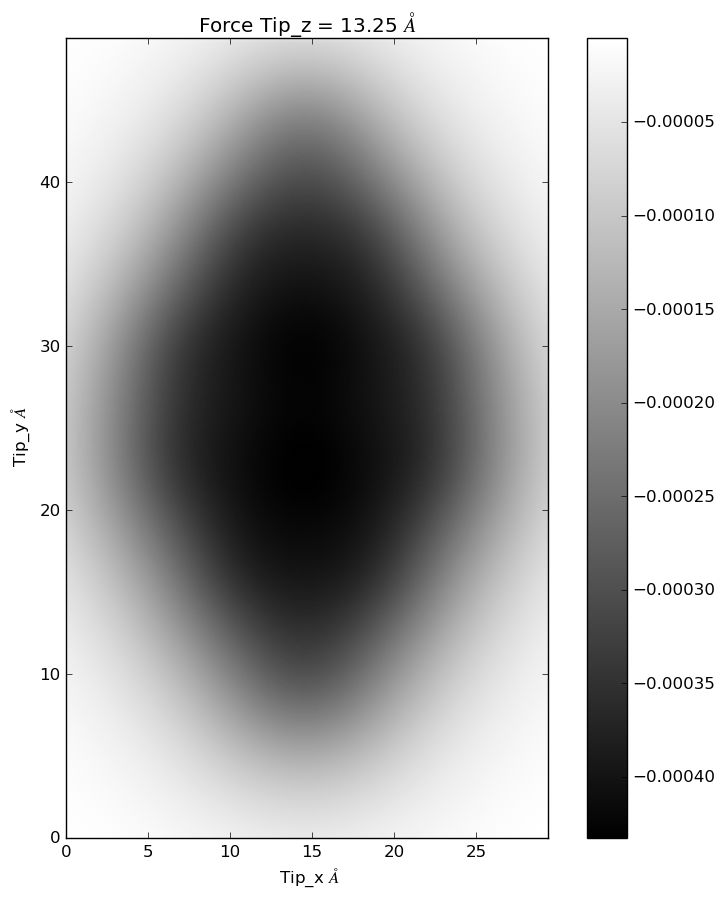

Supplement: File 9 — Datasets A0=5A k=0.5. [file Beilstein_J_Nanotechnol-07-937-s009.zip › S9/A0=5A/k=0.5/results/Force_0055.png]

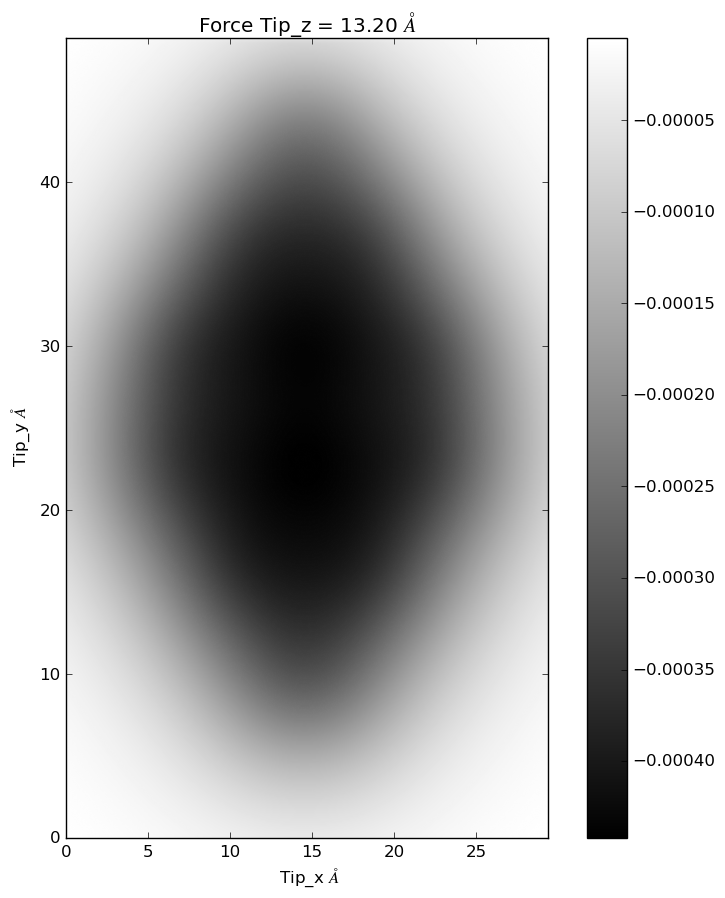

Supplement: File 9 — Datasets A0=5A k=0.5. [file Beilstein_J_Nanotechnol-07-937-s009.zip › S9/A0=5A/k=0.5/results/Force_0056.png]
